# Synthesis of Unsymmetrical Urea Derivatives via PhI(OAc)_2_ and Application in Late-Stage Drug Functionalization

**DOI:** 10.3390/molecules29235669

**Published:** 2024-11-29

**Authors:** Subban Kathiravan, Prakriti Dhillon, Tianshu Zhang, Ian A. Nicholls

**Affiliations:** Bioorganic & Biophysical Chemistry Laboratory, Linnaeus University Centre for Biomaterials Chemistry, Department of Chemistry & Biomedical Sciences, Linnaeus University, SE-39182 Kalmar, Sweden; prakriti.dhillon@lnu.se (P.D.); zhangtianshu1@hotmail.com (T.Z.); ian.nicholls@lnu.se (I.A.N.)

**Keywords:** hypervalent iodine, urea, amides, amines, late-stage functionalization, drugs

## Abstract

Unsymmetrical urea derivatives are essential structural motifs in a wide array of biologically significant compounds. Despite the well-established methods for synthesizing symmetrical ureas, efficient strategies for the synthesis of unsymmetrical urea derivatives remain limited. In this study, we present a novel approach for the synthesis of unsymmetrical urea derivatives through the coupling of amides and amines. Utilizing hypervalent iodine reagent PhI(OAc)_2_ as a coupling mediator, this method circumvents the need for metal catalysts, high temperatures, and inert atmosphere. The reaction proceeds under mild conditions and demonstrates broad substrate scope, including various primary and secondary amines and primary benzamides. This protocol not only offers a practical and versatile route for synthesizing unsymmetrical ureas but also shows significant potential for the late-stage functionalization of complex molecules in drug development.

## 1. Introduction

Urea derivatives are integral to a diverse range of biologically active compounds, pharmaceuticals, agrochemicals, and materials science [1,2,3,4,5,6,7,8,9]. Among these, unsymmetrical urea derivatives stand out due to their unique structural and functional properties [10]. They are prevalent in enzyme inhibitors, antiviral agents, and selective receptor modulators [11,12,13]. Accordingly, the synthesis of this class of compounds is of significance in synthetic organic chemistry, driven by the ongoing need to develop novel compounds with improved biological activities and functionalities.

Historically, the synthesis of urea derivatives has relied heavily on the reaction of amines with isocyanates [14,15]. While this approach is effective, the preparation of unsymmetrical ureas often requires additional steps and the handling of potentially hazardous reagents. Methods such as the direct carbonylation of amines and the use of phosgene or its derivatives, although effective, pose substantial challenges due to their toxicity and stringent reaction conditions [16].

The pressing need for more efficient, safer, and environmentally benign methodologies to synthesize unsymmetrical urea derivatives has motivated a number of recent studies. Rousseaux reported a metal-free synthesis of unsymmetrical ureas and carbamates from CO_2_ and amines via isocyanate intermediates [17], though the use of low temperature to generate the isocyanate intermediate limits its large-scale application. Transition metal-catalyzed synthesis of unsymmetrical ureas has been demonstrated, such as by Nowrouzi et al., who reported a palladium-catalyzed method using chromium hexacarbonyl as a convenient and safer alternative for introducing the carbonyl group [18]. Stahl and co-workers reported a copper-catalyzed benzylic C-H isocyanation and amine coupling sequence that enabled the high-throughput synthesis of pharmaceutically relevant ureas [19]. Furthermore, iron-catalyzed urea synthesis through dehydrogenative coupling of methanol and amines was reported by Bernskoetter [20].

**Scheme 1:**
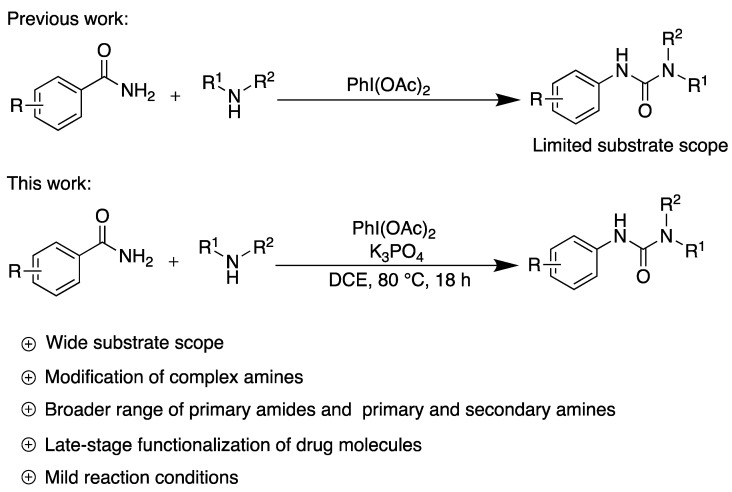
I(III)-mediated urea synthesis [27,28].

In recent years, hypervalent iodine reagents have emerged as versatile tools in organic synthesis [21]. These reagents, particularly iodine(III) compounds like iodobenzene diacetate (PhI(OAc)_2_), have gained popularity due to their mild oxidative properties, ease of handling, and low toxicity. Hypervalent iodine reagents have been successfully employed in a variety of oxidative transformations, including C-H activation, oxidative coupling, and amination reactions [22,23,24,25,26]. Their ability to mediate bond formation under mild conditions makes them attractive for the synthesis of complex organic molecules, including urea derivatives. The use of PhI(OAc)_2_ as an oxidant and coupling mediator in the synthesis of unsymmetrical ureas represents a novel and promising approach by avoiding the use of toxic transition metal catalysts and reagents. Recently, Reboul [27] and Patureau [28] described the use of PhI(OAc)_2_ for the metal-free synthesis of unsymmetrical urea derivatives through the coupling of amines with amides. We have now exploited this development to explore the scope of this reaction through the synthesis of a wide range of primary amides and primary and secondary amines (Scheme 1). This versatility is crucial for the late-stage functionalization of complex molecules with urea moieties, particularly in pharmaceutical synthesis where functional group compatibility and the use of mild reaction conditions are paramount. The ability to introduce unsymmetrical urea functionalities into advanced intermediates or drug candidates without extensive protection–deprotection sequences or harsh conditions represents a significant advancement in this field.

## 2. Results and Discussion

### 2.1. Survey of Substrate Scope: Scope of Primary Amines

To optimize the reaction conditions, we explored various parameters, including solvents, temperature, and base selection (see Supplementary Materials Tables S1 and S2). Only inorganic bases were used, as organic bases have the potential to react with the intermediate isocyanate leading to side reactions, as reported in a previous study [29].

A comparison of solvent effects revealed that DCE provided the highest yields, while MeOH and other tested solvents resulted in lower conversions. Additionally, lowering the reaction temperature led to diminished yields, which is consistent with prior reports [27,28]. Our study demonstrates that the reaction can proceed efficiently at 80 °C, though without the need for excessive amounts of amine (e.g., 17.5 equivalents, as used in [27]).

Under the optimized reaction conditions, isolated yields were below 50% in several cases. The formation of the isocyanate appears to be the limiting step, as unreacted starting materials were observed. Increasing the amount of amine may help overcome this limitation and improve urea formation, as suggested by our comparative studies.

**Scheme 2:**
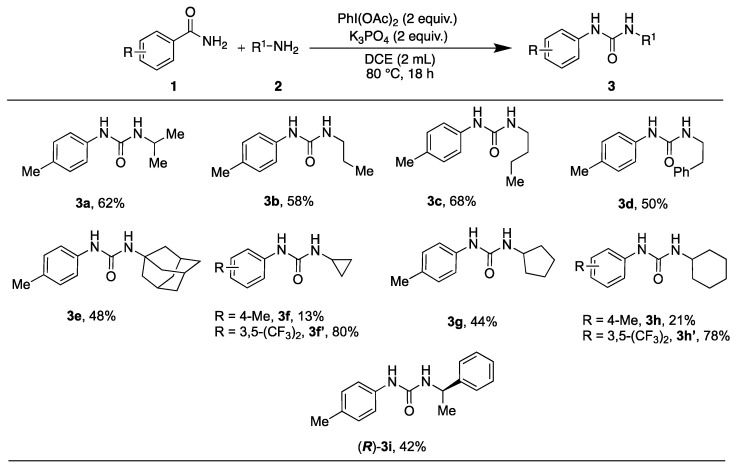
Scope of primary amines.

Initially, the use of aliphatic primary amines in the synthesis of unsymmetrical ureas was investigated (Scheme 2). The results indicated that primary amines with alkyl substituents, such as isopropylamine (**2a**), propylamine (**2b**), and butylamine (**2c**), yielded the corresponding urea derivatives (**3a**–**3c**) in relatively high yields (58–68%). However, a lower yield (50%) was observed for 2-phenylethylamine (**3d**). Even with the sterically demanding adamantylamine (**2e**), commonly used to treat dyskinesia, the expected product (**3e**) was isolated in a similar yield (48%). Cyclopropylamine (**2f**), cyclopentylamine (**2g**), and cyclohexylamine (**2h**) gave varied yields (**3f**–**3h**, 13–44%), likely due to the steric impact of these cyclic structures. Notably, the yields for cyclopropylamine (**2f**) and cyclohexylamine (**2h**) significantly improved when using the more reactive benzamide (**1’**), resulting in yields of 80% and 78%, respectively (**3f’** and **3h’**). The *R*-enantiomer of 1-phenylethylamine (**2i**) behaved as anticipated, giving the corresponding urea in 42% yield.

### 2.2. Survey of Substrate Scope: Scope of Secondary Amines

**Scheme 3:**
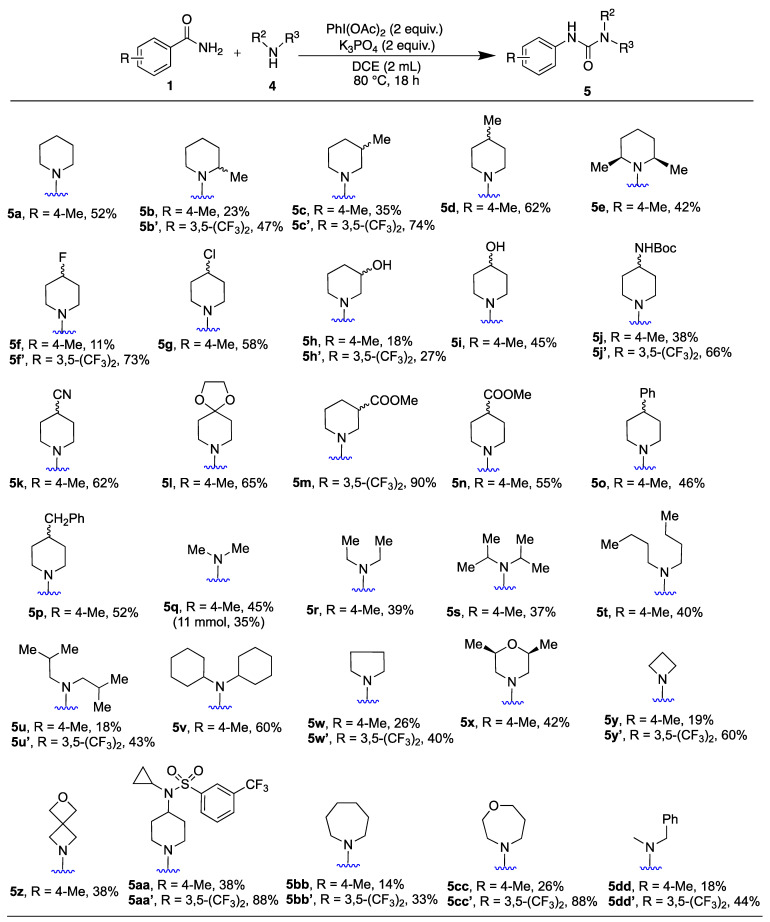
Scope of secondary amines.

Following the use of primary amines, we expanded our study to include a variety of secondary amines for the synthesis of unsymmetrical ureas (Scheme 3). Overall, the yields were generally lower compared to the primary amines, with significant variability depending on both the secondary amine and the benzamide substituents. Although this protocol typically gave satisfactory results, there were cases with lower yields. Thin-layer chromatographic analysis revealed minimal reactivity in these instances, with unreacted amides persisting after the standard reaction time.

Given the prevalence of piperidine moieties in bioactive compounds and their significance in medicinal chemistry, we initially tested various C2, C3, and C4 substituted derivatives, for instance, piperidine (**4a**) gave a 52% yield, while 2-methylpiperidine (**4b**) and 3-methylpiperidine (**4c**) gave lower yields (**5b**, 23% and **5c** 35%, respectively). The yields for **5b’** and **5c’** increased to 47% and 74% with the more reactive benzamide (**1’**). 4-methylpiperidine (**4d**) was well tolerated (**5d**, 62%); additionally, the sterically hindered 2,6-dimethylpiperidine (**4e**) yielded **5e** in 42%, indicating the influence of steric and electronic factors on the reaction outcome. We also found that piperidines with polar functional groups (**4f**–**4n**) were generally compatible. For instance, fluoro (**4f**), chloro (**4g**), hydroxyl (**4h** and **4i**), carbamate (**4j**), cyano (**4r**), ketal (**4l**), and ester (**4m** and **4n**) functionalities yielded products in moderate yields. Enhanced yields were obtained using benzamide **1’**, with fluoro (**5f′**, 73%), 3-OH (**5h’**, 27%), NHBoc (**5j’**, 66%), and 3-COOMe (**5m**, 90%) substituted piperidines. Different reactivity patterns were observed among piperidines with similar functional group substitutions, such as 3-OH (**4h**) versus 4-OH (**4i**), and 3-COOMe (**4m**) versus 4-COOMe (**4n**). However, the 3-COOMe (**4m**) substituted piperidine, which did not yield the product with benzamide **1**, gave a remarkable 90% yield with benzamide **1’**, underscoring the crucial role of the amide’s substituents in determining reactivity with the functional group in piperidine. Phenyl (**4o**) and benzyl (**4p**) substituted piperidines yielded products **5o** and **5p** in moderate yields (46% and 52%). Next, we screened various acyclic dialkyl secondary amines (**4q**–**4v**) and obtained moderate yields for the corresponding unsymmetrical ureas (**5q**–**5v**). Specifically, product **5u** was isolated in an 18% yield, with unreacted starting material present in the reaction mixture. To improve the yields, we examined other benzamide substituents. Notably, using a 3,5-bistrifluoromethyl substituted benzamide (**1′**) significantly increased the yield of product **5u′** to 43%, a trend observed with other amine substrates as well. This strategy of incorporating trifluoromethyl substituted amides shows potential in medicinal chemistry [30]. It is worth noting that gaseous dimethylamine (**4q**) was used as a 40% aqueous solution, demonstrating the suitability of water in this reaction system and its scalability for gram-scale reactions. Dicyclohexylamine (**4v**) provided a higher product yield (**5v**, 60%). Pyrrolidine with 4-methyl substituted benzamide (**1**) produced **5w** in a 26% yield, whereas the 3,5-bistrifluoromethyl benzamide (**1′**) yielded **5w′** at 40%. Both reactions showed unreacted starting material, and attempts to isolate pure starting materials resulted in only trace amounts, with the remainder decomposing. The 2,6-dimethylmorpholine (**4x**) moiety, significant in medicinal chemistry, yielded **5x** at 42%. Incorporating small heterocycles, a common approach in medicinal chemistry, was further explored with azetidine (**4y**) and 2-oxa-6-azaspiro[3,3]heptane (**4z**), a spirocyclic bioisostere of morpholine. The highly functionalized piperidine (**4aa**) was successfully used, demonstrating the potential for late-stage modification of medicinally relevant compounds through this unsymmetrical urea synthesis. Other important heterocycles, such as azepane (**4bb**) and 1,4-oxazepane (**4cc**), known for their pharmacological properties, were also included, as was *N*-methyl benzylamine (**4dd**); in each case, the corresponding urea was obtained, with significantly improved yields observed with benzamide **1′** (**5dd′**).

### 2.3. Survey of Substrate Scope: Scope of Benzamides

**Scheme 4:**
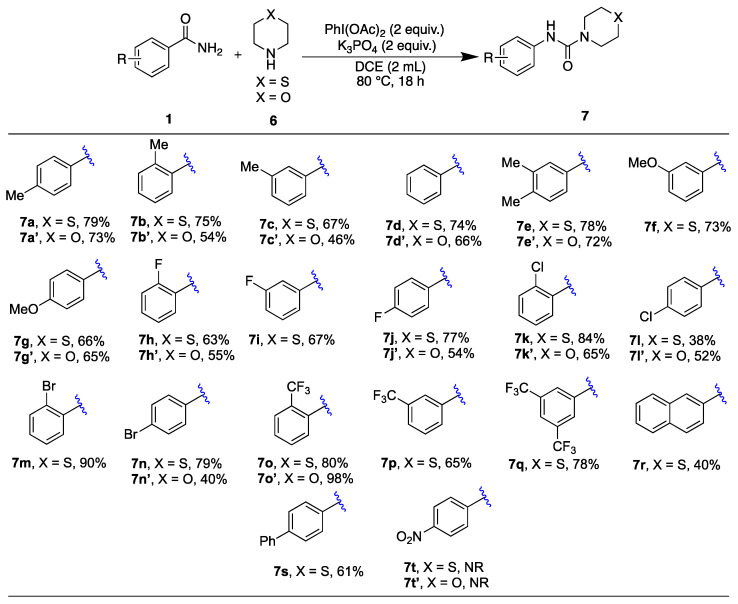
Scope of benzamides.

We turned our attention to examining the reaction scope and application with various benzamides (**1a**–**1t**) used with thiomorpholine (**6**) and morpholine (**6’**) (Scheme 4). We tested benzamides with different substituents (*ortho*, *meta*, and *para)*, including methyl (**1a**–**1c**), unsubstituted (**1d**), 3,4-dimethyl (**1e**), methoxy (**1f**–**1g**), halo (**1h**–**1n**), trifluoromethyl (**1o**–**1q**), naphthyl (**1r**), phenyl (**1s**) and nitro (**1t**) groups. Among these, the benzamide bearing a strongly electron-withdrawing trifluoromethyl group (**1o**) with morpholine (**6′**) yielded the product with a remarkable efficiency (**7o′**). However, the *para*-nitro derivative (**1t**) did not react, which we attribute to the benzamide’s insolubility.

### 2.4. Survey of Substrate Scope: Scope of Late-Stage Functionalization

**Scheme 5:**
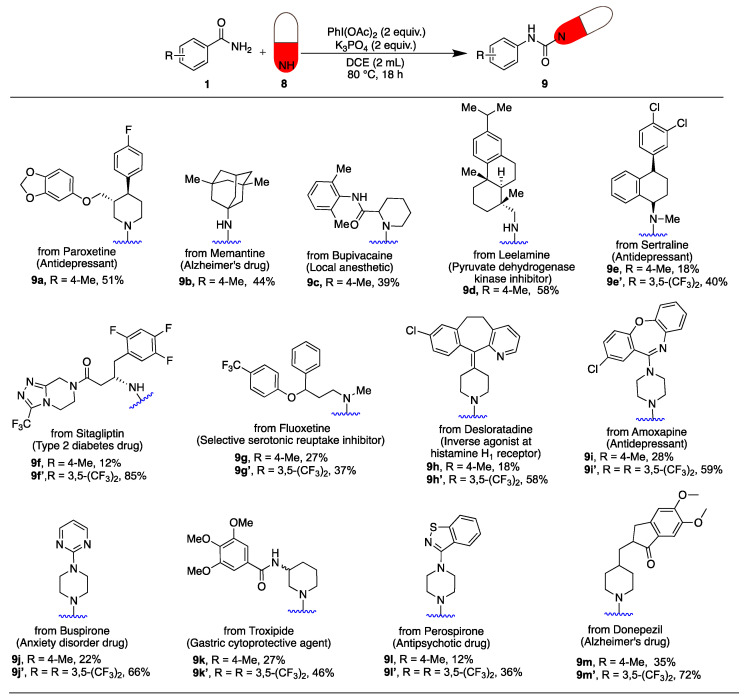
Late-stage functionalization of drugs.

Late-stage functionalization is crucial in medicinal chemistry for modifying drug scaffolds to enhance their properties [31,32,33]. It enables the introduction of functional and structural diversity, which is important for exploring new chemical spaces in drug development. To illustrate the applicability of our unsymmetrical urea synthesis in late-stage functionalization, we applied our method to a range of drugs (Scheme 5): Paroxetine (**8a**), Memantine (**8b**), Bupivacaine (**8c**), Leelamine (**8d**), Sertraline (**8e**), Sitagliptin (**8f**), Fluoxetine (**8g**), Desloratadine (**8h**), Amoxapine (**8i**), Buspirone (**8j**), Troxipide (**8k**), Perospirone (**8l**), and Donepezil (**8m**). All the drugs showed good reactivity by furnishing the products (**9a**–**9m**) in low to moderate yields.

### 2.5. Proposed Mechanism

**Scheme 6:**
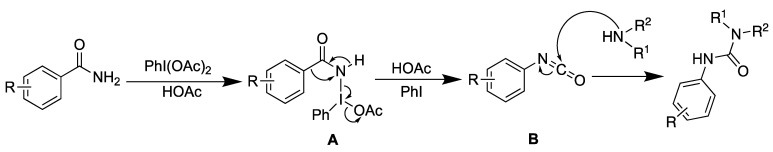
Proposed mechanism.

Based on the previous reports [27,28] and the observed reactivity, we propose the following mechanism (Scheme 6): Initially, benzamide undergoes addition with PhI(OAc)_2_, forming an iodonium intermediate (**A**) and releasing acetic acid, which was confirmed by LC-MS analysis (See Supplementary Materials). The need for an excess of amine arises from its role in neutralizing the acetic acid. Following this, the iodonium intermediate (**A**) undergoes a Hofmann rearrangement to form an isocyanate intermediate (**B**), which then reacts with the amines to produce the unsymmetrical urea.

## 3. Materials and Methods

All catalytic reactions were carried out under aerobic conditions using standard procedures. Solvents and reagents were sourced from Merck (Stockholm, Sweden) and Chemtronica (Stockholm, Sweden). Moisture control measures were not implemented throughout the experiments. All glassware was dried overnight at 120 °C and further flame-dried, as necessary. Silica gel (Carlo Erba, 60 Å) was used for column chromatography. Thin-layer chromatography (TLC) was performed on silica gel-precoated aluminum sheets with a fluorescence indicator at 254 nm. Preparative TLC was conducted using plates from Aldrich (Analtech, UV254 20 × 20 cm, 500 µm). Yields represent isolated products, with purity verified by 1H NMR spectroscopy. NMR spectra were recorded on a Bruker Ascend 400 instrument at frequencies of 400 MHz (1H NMR), 101 MHz (13C NMR), and 376 MHz (19F NMR). Chemical shifts (δ) are given in ppm, with spectra referenced to residual non-deuterated solvent signals. High-resolution mass spectra (HRMS) were obtained at the Lund University Kemi Centrum Mass Spectrometry facility using a Waters XEVO-G2 QTOF instrument. The parameters for ESI+ were as follows: capillary voltage 3 kV, cone voltage 35V, extractor 4, source temperature 120 °C, desolvation temperature 300 °C, cone gas flow 50, desolvation gas flow 400. Continuum resolution mode was applied, with an *m/z* range of 100–1200, and manual lock mass correction using Leucine Enkephalin (*m*/*z* 556.2771). All starting materials were obtained from Aldrich or Chemtronica and used without further purification. The solvents were neither dried nor stored under an inert atmosphere.

General Procedure: In a 10 mL screw cap reaction tube, PhI(OAc)_2_ (2 equiv.), K_3_PO_4_ (2 equiv.), benzamide (1 equiv.) and 1,2-dichloroethane (12 M) were combined with the appropriate amine (2 equiv.) under ambient conditions. If the amine was solid, it was added at this step, whereas liquid amines were dissolved in 1,2-dichloroethane (12 M) before being added. The reaction mixture was stirred at 80 °C for 18 h, using an aluminum block heated by an oil bath. The reaction mixture was then allowed to cool to room temperature, followed by the addition of silica gel. The crude product was then purified using either column chromatography or preparative thin-layer chromatography, with an elution gradient of petroleum ether and acetone.

### Spectral Data

**1-isopropyl-3-(*p*-tolyl)urea** (**3a**): General procedure was followed using **1a** (50 mg, 0.37 mmol, 1.0 equiv.), **2b** (63 μL, 2 equiv.), PhI(OAc)_2_ (238 mg, 2 equiv.), K_3_PO_4_ (157 mg, 2 equiv.), 1,2-DCE (2 mL), at 80 °C for 18 h. Column chromatography was performed using 85/15 *v/v* petroleum ether/acetone as eluent and yielded **3a** (44 mg, 62%). Beige solid. mp—150–152 °C. **FTIR (cm^−1^):** 3314, 2964, 2920, 2871, 1685, 1632, 1592, 1543, 1505, 1235, 1173, 1067, 816, 776. **^1^H NMR (400 MHz, CDCl_3_) δ** 1H NMR (400 MHz, Chloroform-d) δ 7.31 (s, 1H), 7.13 (d, *J* = 8.4 Hz, 2H), 7.02 (d, *J* = 8.2 Hz, 2H), 5.38 (d, *J* = 7.8 Hz, 1H), 3.94 (dq, *J* = 13.1, 6.6 Hz, 1H), 2.26 (s, 3H), 1.10 (d, J = 6.6 Hz, 6H). **^13^C NMR (101 MHz, CDCl_3_) δ** 156.0, 136.4, 132.7, 129.5, 120.7, 41.9, 23.2, 20.7. **HRMS (ESI):** Exact mass calculated for C_11_H_17_N_2_O [M + H]^+^: 193.1336, found: 193.1337.

**1-propyl-3-(*p*-tolyl)urea** (**3b**): General procedure was followed using **1a** (50 mg, 0.37 mmol, 1.0 equiv.), **2a** (61 μL, 2 equiv.), PhI(OAc)_2_ (238 mg, 2 equiv.), K_3_PO_4_ (157 mg, 2 equiv.), 1,2-DCE (2 mL), at 80 °C for 18 h. Column chromatography was performed using 85/15 *v/v* petroleum ether/acetone as eluent and yielded **3b** (41 mg, 58%). Green solid. mp—97–99 °C. **FTIR (cm^−1^):** 3310, 2959, 2920, 2869, 1638, 1585, 1561, 1508, 1459, 1306, 1233, 1107, 810. **^1^H NMR (400 MHz, CDCl_3_) δ** 7.40 (s, 1H), 7.12 (d, *J* = 8.3 Hz, 2H), 7.02 (d, *J* = 8.3 Hz, 2H), 5.60 (t, *J* = 5.8 Hz, 1H), 3.15–3.05 (m, 2H), 2.26 (s, 3H), 1.43 (q, *J* = 7.3 Hz, 2H), 0.85 (t, *J* = 7.4 Hz, 3H). **^13^C NMR (101 MHz, CDCl_3_) δ** 156.8, 136.4, 132.7, 129.5, 120.8, 41.9, 23.3, 20.7, 11.3. **HRMS (ESI):** Exact mass calculated for C_11_H_17_N_2_O [M + H]^+^: 193.1336, found: 193.1337.

**1-butyl-3-(*p*-tolyl)urea** (**3c**): General procedure was followed using **1a** (50 mg, 0.37 mmol, 1.0 equiv.), **2c** (73 μL, 2 equiv.), PhI(OAc)_2_ (238 mg, 2 equiv.), K_3_PO_4_ (157 mg, 2 equiv.), 1,2-DCE (2 mL), at 80 °C for 18 h. Column chromatography was performed using 85/15 *v/v* petroleum ether/acetone as eluent and yielded **3c** (52 mg, 68%). Brown solid. mp—103–105 °C. **FTIR (cm^−1^):** 3312, 2959, 2931, 2857, 1625, 1585, 1556, 1282, 1226, 1107, 816, 776. **^1^H NMR (400 MHz, CDCl_3_) δ** 7.46 (s, 1H), 7.13 (d, *J* = 8.4 Hz, 2H), 7.01 (d, *J* = 8.3 Hz, 2H), 5.64 (t, *J* = 5.7 Hz, 1H), 3.13 (td, *J* = 7.1, 5.6 Hz, 2H), 2.26 (s, 3H), 1.43–1.33 (m, 2H), 1.26 (dq, *J* = 13.9, 7.1 Hz, 2H), 0.86 (t, *J* = 7.3 Hz, 3H). **^13^C NMR (101 MHz, CDCl_3_) δ** 156.9, 136.4, 132.6, 129.5, 120.8, 39.9, 32.2, 20.7, 20.0, 13.7. **HRMS (ESI):** Exact mass calculated for C_12_H_19_N_2_O [M + H]^+^: 207.1492, found: 207.1494.

**1-phenethyl-3-(*p*-tolyl)urea** (**3d**): General procedure was followed using **1a** (50 mg, 37 mmol, 1.0 equiv.), **2d** (93 μL, 2 equiv.), PhI(OAc)_2_ (238 mg, 2 equiv.), K_3_PO_4_ (157 mg, 2 equiv.), 1,2-DCE (2 mL), at 80 °C for 18 h. Column chromatography was performed using 85/15 *v/v* petroleum ether/acetone as eluent and yielded **3d** (47 mg, 50%). Yellow solid. mp—150–152 °C. **FTIR (cm^−1^)**: 3322, 3057, 3026, 2920, 1631, 1592, 1561, 1450, 1406, 1306, 1227, 1079, 811, 757, 736, 701. **^1^H NMR (400 MHz, CDCl_3_) δ** 7.29 (d, *J* = 6.9 Hz, 1H), 7.26 (s, 1H), 7.24–7.20 (m, 1H), 7.20–7.15 (m, 2H), 7.10–7.03 (m, 4H), 6.27 (s, 1H), 4.79 (s, 1H), 3.48 (td, *J* = 6.9, 5.8 Hz, 2H), 2.81 (t, *J* = 6.9 Hz, 2H), 2.30 (s, 3H). **^13^C NMR (101 MHz, CDCl_3_) δ** 156.0, 139.1, 135.4, 134.2, 129.9, 128.8, 128.6, 126.4, 122.3, 41.5, 36.2, 20.8. **HRMS (ESI):** Exact mass calculated for C_16_H_19_N_2_O [M + H]^+^: 255.1492, found: 255.1494.

**1-((3*s*,5*s*,7*s*)-adamantan-1-yl)-3-(p-tolyl)urea** (**3e**): General procedure was followed using **1a** (50 mg, 0.37 mmol, 1.0 equiv.), **2e** (225 mg, 0.74 mmol, 2 equiv.), PhI(OAc)_2_ (238 mg, 2 equiv.), K_3_PO_4_ (157 mg, 2 equiv.), 1,2-DCE (2 mL), at 80 °C for 18 h. Column chromatography was performed using 85/15 *v/v* petroleum ether/acetone as eluent and yielded **3e** (39 mg, 48%). Brown solid. mp—231–233 °C. **FTIR (cm^−1^):** 3321, 2906, 2851, 1643, 1587, 1550, 1512, 1450, 1357, 1290, 1228, 1098, 807. **^1^H NMR (400 MHz, CDCl_3_) δ** ^1^H NMR (400 MHz, Chloroform-d) δ 7.13 (d, J = 8.5 Hz, 2H), 7.09 (d, J = 8.5 Hz, 2H), 6.12 (s, 1H), 4.53 (s, 1H), 2.30 (s, 3H), 2.06 (d, J = 2.6 Hz, 4H), 1.98 (d, J = 2.9 Hz, 6H), 1.67 (t, J = 3.2 Hz, 8H). **^13^C NMR (101 MHz, CDCl_3_) δ** 154.7, 136.1, 133.5, 129.8, 121.7, 51.2, 42.2, 36.4, 29.5, 20.8. **HRMS (ESI):** Exact mass calculated for C_18_H_25_N_2_O [M + H]^+^: 285.1962, found: 285.1961.

**1-cyclopropyl-3-(*p*-tolyl)urea** (**3f**): General procedure was followed using **1a** (50 mg, 0.37 mmol, 1.0 equiv.), **2f** (51 μL, 2 equiv.), PhI(OAc)_2_ (238 mg, 2 equiv.), K_3_PO_4_ (157 mg, 2 equiv.), 1,2-DCE (2 mL), at 80 °C for 18 h. Column chromatography was performed using 85/15 *v/v* petroleum ether/acetone as eluent and yielded **3f** (10 mg, 13%). Yellow solid. mp—151–153 °C. **FTIR (cm^−1^):** 3301, 3090, 2984, 2915, 1632, 1592, 1556, 1503, 1302, 1282, 1235, 1018, 816, 701. **^1^H NMR (400 MHz, CDCl_3_) δ** 7.30–7.23 (m, 3H), 7.11 (d, *J* = 8.3 Hz, 2H), 6.81 (s, 1H), 4.95 (s, 1H), 2.58 (ttd, *J* = 6.8, 3.6, 1.4 Hz, 1H), 2.30 (s, 3H), 0.82 (td, *J* = 6.8, 4.7 Hz, 2H), 0.67–0.59 (m, 2H). **^13^C NMR (101 MHz, CDCl_3_) δ** 156.6, 135.7, 133.3, 129.6, 120.6, 22.6, 20.7, 7.5. **HRMS (ESI):** Exact mass calculated for C_11_H_15_N_2_O [M + H]^+^: 191.1179, found: 191.1180.

**1-(3,5-bis(trifluoromethyl)phenyl)-3-cyclopropylurea** (**3f′**): General procedure was followed using **1′** (95 mg, 0.37 mmol, 1.0 equiv.), **2f** (51 μL, 2 equiv.), PhI(OAc)_2_ (238 mg, 2 equiv.), K_3_PO_4_ (157 mg, 2 equiv.), 1,2-DCE (2 mL), at 80 °C for 18 h. Column chromatography was performed using 85/15 *v/v* petroleum ether/acetone as eluent and yielded **3f’** (93 mg, 80%). Colorless solid. mp—175–177 °C. **FTIR (cm^−1^):** 3316, 1660, 1552, 1472, 1437, 1384, 1264, 1123, 1068, 1021, 942, 875, 678. **^1^H NMR (400 MHz, CDCl_3_) δ** 7.94 (s, 2H), 7.53 (s, 1H), 7.24 (s, 1H), 5.10 (s, 1H), 2.63 (ttd, J = 6.7, 3.6, 1.2 Hz, 1H), 0.92 (td, J = 6.8, 4.8 Hz, 2H), 0.75–0.69 (m, 2H). **^13^C NMR (101 MHz, CDCl_3_) δ** 155.5, 140.0, 132.8, 132.4, 124.5, 121.8, 118.9, 116.3, 22.6, 7.7. **^19^F NMR (376 MHz, CDCl_3_)** δ −63.0. **HRMS (ESI):** Exact mass calculated for C_12_H_11_F_6_N_2_O [M + H]^+^: 313.0771, found: 313.0776.

**1-cyclopentyl-3-(*p*-tolyl)urea** (**3g)**: General procedure was followed using **1a** (50 mg, 0.37 mmol, 1.0 equiv.), **2g** (73 μL, 2 equiv.), PhI(OAc)_2_ (238 mg, 2 equiv.), K_3_PO_4_ (157 mg, 2 equiv.), 1,2-DCE (2 mL), at 80 °C for 18 h. Column chromatography was performed using 85/15 *v/v* petroleum ether/acetone as eluent and yielded **3g** (35 mg, 44%). Brown solid. mp—186–188 °C. **FTIR (cm^−1^):** 3301, 2953, 2864, 1627, 1585, 1556, 1508, 1282, 1233, 1042, 938, 816, 778. **^1^H NMR (400 MHz, CDCl_3_) δ** 7.17–7.12 (m, 2H), 7.05 (d, *J* = 2.1 Hz, 2H), 7.04 (d, *J* = 1.8 Hz, 1H), 5.29 (d, *J* = 7.3 Hz, 1H), 4.08 (h, *J* = 6.9 Hz, 1H), 2.27 (s, 3H), 1.98–1.87 (m, 2H), 1.65–1.47 (m, 4H), 1.38–1.28 (m, 2H). **^13^C NMR (101 MHz, CDCl_3_) δ** 156.1, 136.1, 133.2, 129.7, 121.2, 51.9, 33.4, 23.5, 20.7. **HRMS (ESI):** Exact mass calculated for C_13_H_19_N_2_O [M + H]^+^: 219.1492, found: 219.1494.

**1-cyclohexyl-3-(*p*-tolyl)urea** (**3h**): General procedure was followed using **1a** (50 mg, 0.37 mmol, 1.0 equiv.), **2h** (84 μL, 2 equiv.), PhI(OAc)_2_ (238 mg, 2 equiv.), K_3_PO_4_ (157 mg, 2 equiv.), 1,2-DCE (2 mL), at 80 °C for 18 h. Column chromatography was performed using 85/15 *v/v* petroleum ether/acetone as eluent and yielded **3h** (18 mg, 21%). Brown solid. mp—181–183 °C. **FTIR (cm^−1^):** 3330, 3028, 2926, 2849, 1625, 1590, 1561, 1505, 1441, 1310, 1226, 1044, 889, 818, 776. **^1^H NMR (400 MHz, CDCl_3_) δ** 7.17–7.08 (m, 5H), 6.35 (s, 1H), 4.71 (d, *J* = 8.1 Hz, 1H), 3.65 (dddd, *J* = 14.6, 10.5, 7.9, 3.9 Hz, 1H), 2.31 (s, 3H), 1.94 (dd, *J* = 12.5, 3.9 Hz, 3H), 1.71–1.55 (m, 6H), 1.41–1.28 (m, 4H), 1.19–1.03 (m, 4H). **^13^C NMR (101 MHz, CDCl_3_) δ** 155.4, 135.8, 133.8, 129.9, 122.0, 49.0, 33.6, 25.5, 24.9, 20.8. **HRMS (ESI):** Exact mass calculated for C_14_H_21_N_2_O [M + H]^+^: 233.1649, found: 233.1650.

**1-(3,5-bis(trifluoromethyl)phenyl)-3-cyclohexylurea** (**3h′**): General procedure was followed using **1′** (95 mg, 0.37 mmol, 1.0 equiv.), **2h** (84 μL, 2 equiv.), PhI(OAc)_2_ (238 mg, 2 equiv.), K_3_PO_4_ (157 mg, 2 equiv.), 1,2-DCE (2 mL), at 80 °C for 18 h. Column chromatography was performed using 85/15 *v/v* petroleum ether/acetone as eluent and yielded **3h′** (105 mg, 78%). Brown solid. mp—179–181 °C. **FTIR (cm^−1^):** 3342, 2936, 2854, 1656, 1543, 1474, 1439, 1386, 1269, 1127, 1041, 935, 871, 674. **^1^H NMR (400 MHz, CDCl_3_) δ** 7.86 (d, *J* = 1.6 Hz, 2H), 7.47 (s, 1H), 6.82 (s, 1H), 4.75 (d, *J* = 7.9 Hz, 1H), 3.66 (dtd, *J* = 10.7, 7.1, 3.8 Hz, 1H), 2.03–1.94 (m, 3H), 1.72 (dt, *J* = 13.4, 3.9 Hz, 4H), 1.68–1.55 (m, 5H), 1.44–1.31 (m, 5H), 1.21–1.09 (m, 4H). **^13^C NMR (101 MHz, CDCl_3_) δ** 153.7, 151.6, 145.8, 140.7, 132.3, 132.0, 123.5, 120.9, 118.4, 116.3, 49.3, 33.5, 29.7, 25.4, 24.8, 1.0. **HRMS (ESI):** Exact mass calculated for C_15_H_17_F_6_N_2_O [M + H]^+^: 355.1240, found: 355.1241.

**(*R*)-1-(1-phenylethyl)-3-(*p*-tolyl)urea** (**3i**): General procedure was followed using **1a** (50 mg, 0.37 mmol, 1.0 equiv.), **2i** (95 μL, 2 equiv.), PhI(OAc)_2_ (238 mg, 2 equiv.), K_3_PO_4_ (157 mg, 2 equiv.), 1,2-DCE (2 mL), at 80 °C for 18 h. Column chromatography was performed using 85/15 *v/v* petroleum ether/acetone as eluent and yielded **3i** (39 mg, 42%). Brown solid. mp—147–149 °C. **FTIR (cm^−1^):** 3290, 3090, 3026, 2977, 2917, 1627, 1585, 1561, 1517, 1448, 1310, 1228, 1018, 829, 741. **^1^H NMR (400 MHz, CDCl_3_) δ** 7.28–7.24 (m, 1H), 7.23 (d, *J* = 2.7 Hz, 3H), 7.21 (dt, *J* = 2.7, 1.4 Hz, 1H), 7.19 (s, 1H), 7.10 (s, 1H), 7.09–7.05 (m, 2H), 6.99 (d, *J* = 8.4 Hz, 2H), 5.67 (d, *J* = 7.6 Hz, 1H), 4.89 (p, *J* = 7.0 Hz, 1H), 2.25 (s, 3H), 1.33 (d, *J* = 6.9 Hz, 3H). **^13^C NMR (101 MHz, CDCl_3_) δ** 155.8, 144.2, 136.1, 132.9, 129.6, 128.6, 127.0, 125.9, 120.8, 49.7, 22.9, 20.7. **HRMS (ESI):** Exact mass calculated for C_16_H_18_N_2_O [M + H]^+^: C_16_H_19_N_2_O [M + H]^+^: 255.1492, found: 255.1493.

**N-(*p*-tolyl)piperidine-1-carboxamide** (**5a**): General procedure was followed using **1a** (50 mg, 0.37 mmol, 1.0 equiv.), **4a** (63 mg, 0.74 mmol, 2 equiv.), PhI(OAc)_2_ (238 mg, 2 equiv.), K_3_PO_4_ (157 mg, 2 equiv.), 1,2-DCE (2 mL), at 80 °C for 18 h. Column chromatography was performed using 85/15 *v/v* petroleum ether/acetone as eluent and yielded **5a** (42 mg, 52%). Yellow solid. mp—149–150 °C. FTIR (cm^−1^): 3274, 3124, 2936, 2847, 1627, 1594, 1508, 1421, 1240, 1070, 1019, 805, 747. **^1^H NMR (400 MHz, CDCl_3_) δ** 7.27–7.23 (m, 2H), 7.09 (d, *J* = 8.3 Hz, 2H), 6.39 (s, 1H), 3.45 (dd, *J* = 6.2, 4.1 Hz, 4H), 2.31 (s, 3H), 1.71–1.57 (m, 6H). **^13^C NMR (101 MHz, CDCl_3_) δ** 155.2, 136.7, 132.3, 129.3, 120.1, 45.2, 25.7, 24.4, 20.7. **HRMS (ESI):** Exact mass calculated for C_13_H_18_N_2_O [M+Na]^+^: 241.1312, found: 241.1318.

**2-methyl-*N*-(*p*-tolyl)piperidine-1-carboxamide** (**5b**): General procedure was followed using **1a** (50 mg, 0.37 mmol, 1.0 equiv.), **4b** (73 mg, 0.74 mmol, 2 equiv.), PhI(OAc)_2_ (238 mg, 2 equiv.), K_3_PO_4_ (157 mg, 2 equiv.), 1,2-DCE (2 mL), at 80 °C for 18 h. Column chromatography was performed using 85/15 *v/v* petroleum ether/acetone as eluent and yielded **5b** (20 mg, 23%). Brown solid. mp—153–154 °C. **FTIR (cm^−1^):** 3311, 2925, 2854, 1627, 1589, 1512, 1417, 1373, 1234, 1176, 1052, 811, 789. **^1^H NMR (400 MHz, CDCl_3_) δ** 7.16 (d, *J* = 8.4 Hz, 2H), 7.00 (d, *J* = 8.3 Hz, 2H), 6.23 (s, 1H), 4.29 (pd, *J* = 7.0, 1.9 Hz, 1H), 3.84–3.76 (m, 1H), 2.90 (td, *J* = 13.0, 3.0 Hz, 1H), 2.21 (s, 3H), 1.72–1.59 (m, 3H), 1.59–1.48 (m, 3H), 1.48–1.34 (m, 1H), 1.15 (d, *J* = 6.9 Hz, 3H). **^13^C NMR (101 MHz, CDCl_3_) δ** 155.1, 136.7, 132.3, 129.3, 120.1, 46.6, 39.0, 30.2, 25.6, 20.7, 18.5, 15.7. **HRMS (ESI):** Exact mass calculated for C_14_H_21_N_2_O [M + H]^+^: 233.1649, found: 233.1651.

**N-(3,5-bis(trifluoromethyl)phenyl)-2-methylpiperidine-1-carboxamide** (**5b′**): General procedure was followed using **1′** (95 mg, 0.37 mmol, 1.0 equiv.), **4b** (73 mg, 0.74 mmol, 2 equiv.), PhI(OAc)_2_ (238 mg, 2 equiv.), K_3_PO_4_ (157 mg, 2 equiv.), 1,2-DCE (2 mL), at 80 °C for 18 h. Column chromatography was performed using 85/15 *v/v* petroleum ether/acetone as eluent and yielded **5b′** (61 mg, 47%). Brown solid. mp—179–181 °C. **FTIR (cm^−1^):** 3342, 2936, 2854, 1656, 1543, 1474, 1439, 1386, 1269, 1127, 1041, 935, 871, 674. **^1^H NMR (400 MHz, CDCl_3_) δ** 7.87–7.83 (m, 2H), 7.45 (s, 1H), 6.99 (s, 1H), 4.42 (qd, J = 9.5, 6.1, 3.8 Hz, 1H), 3.91 (dd, J = 13.5, 2.5 Hz, 1H), 3.02 (td, J = 13.1, 3.0 Hz, 1H), 1.78–1.56 (m, 5H), 1.55–1.40 (m, 1H), 1.24 (d, J = 6.9 Hz, 3H). **^13^C NMR (101 MHz, CDCl_3_) δ** 154.1, 141.0, 137.4, 132.3, 132.0, 131.7, 131.4, 130.2, 127.4, 127.3, 124.6, 121.9, 119.4, 119.3, 115.8, 115.7, 115.6, 46.9, 39.2, 30.1, 25.5, 18.4, 15.8. **HRMS (ESI):** Exact mass calculated for C_15_H_17_F_6_N_2_O [M + H]^+^: 355.1240, found: 355.1250.

**3-methyl-N-(*p*-tolyl)piperidine-1-carboxamide** (**5c**): General procedure was followed using **1a** (50 mg, 0.37 mmol, 1.0 equiv.), **4c** (73 mg, 0.74 mmol, 2 equiv.), PhI(OAc)_2_ (238 mg, 2 equiv.), K_3_PO_4_ (157 mg, 2 equiv.), 1,2-DCE (2 mL), at 80 °C for 18 h. Column chromatography was performed using 85/15 *v/v* petroleum ether/acetone as eluent and yielded **5c** (30 mg, 35%). Yellow solid. mp—107–109 °C. **FTIR (cm^−1^):** 3338, 3285, 2920, 2847, 1631, 1589, 1508, 1408, 1234, 1035, 962, 807, 747. **^1^H NMR (400 MHz, CDCl_3_) δ** 7.25 (d, *J* = 8.5 Hz, 2H), 7.10 (d, *J* = 8.3 Hz, 2H), 6.35 (s, 1H), 4.00–3.90 (m, 2H), 2.86 (ddd, *J* = 13.2, 11.8, 3.0 Hz, 1H), 2.52 (dd, *J* = 13.1, 10.7 Hz, 1H), 1.86 (dtd, *J* = 12.8, 3.7, 1.9 Hz, 1H), 1.78–1.48 (m, 5H), 1.20–1.07 (m, 1H), 0.95 (d, *J* = 6.7 Hz, 3H). **^13^C NMR (101 MHz, CDCl_3_) δ** 155.1, 136.6, 132.3, 129.3, 120.0, 51.8, 44.7, 32.9, 31.0, 25.1, 20.7, 19.0. **HRMS (ESI):** Exact mass calculated for C_14_H_20_N_2_O [M + Na]^+^: 255.1468, found: 255.1479.

***N*-(3,5-bis(trifluoromethyl)phenyl)-3-methylpiperidine-1-carboxamide** (**5c’**): General procedure was followed using **1′** (95 mg, 0.37 mmol, 1.0 equiv.), **4c** (73 mg, 0.74 mmol, 2 equiv.), PhI(OAc)_2_ (238 mg, 2 equiv.), K_3_PO_4_ (157 mg, 2 equiv.), 1,2-DCE (2 mL), at 80 °C for 18 h. Column chromatography was performed using 85/15 *v/v* petroleum ether/acetone as eluent and yielded **5c’** (97 mg, 74%). Yellow solid. mp—108–110 °C. **FTIR (cm^−1^):** 3267, 2974, 2869, 1638, 1534, 1441, 1368, 1269, 1121, 1085, 966, 882, 838, 677. **^1^H NMR (400 MHz, CDCl_3_) δ** 7.80 (s, 2H), 7.65 (s, 1H), 7.40–7.37 (m, 1H), 4.08–3.95 (m, 2H), 2.89–2.79 (m, 1H), 2.51 (dd, J = 13.1, 10.7 Hz, 1H), 1.84 (dd, J = 13.3, 3.8 Hz, 1H), 1.69 (dt, J = 13.5, 3.5 Hz, 1H), 1.60 (dt, J = 6.7, 3.9 Hz, 1H), 1.55–1.43 (m, 1H), 1.18–1.07 (m, 1H), 0.89 (d, J = 6.6 Hz, 3H). **^13^C NMR (101 MHz, CDCl_3_) δ** 154.1, 141.0, 137.4, 132.3, 132.0, 131.7, 131.4, 130.2, 127.4, 127.3, 124.6, 121.9, 119.4, 119.3, 115.8, 115.7, 115.6, 46.9, 39.2, 30.1, 25.5, 18.4, 15.8. **HRMS (ESI):** Exact mass calculated for C_15_H_17_F_6_N_2_O [M + H]^+^: 355.1240, found: 355.1249.

**4-methyl-N-(*p*-tolyl)piperidine-1-carboxamide** (**5d**): General procedure was followed using **1a** (50 mg, 0.37 mmol, 1.0 equiv.), **4d** (73 mg, 0.74 mmol, 2 equiv.), PhI(OAc)_2_ (238 mg, 2 equiv.), K_3_PO_4_ (157 mg, 2 equiv.), 1,2-DCE (2 mL), at 80 °C for 18 h. Column chromatography was performed using 85/15 *v/v* petroleum ether/acetone as eluent and yielded **5d** (53 mg, 62%). Yellow solid. mp—112–113 °C. **FTIR (cm^−1^):** 3350, 2911, 2860, 1634, 1589, 1510, 1417, 1306, 1236, 1077, 964, 805, 745. **^1^H NMR (400 MHz, CDCl_3_) δ** 7.25 (d, *J* = 8.5 Hz, 2H), 7.09 (d, *J* = 8.2 Hz, 2H), 6.40 (s, 1H), 4.04 (d, *J* = 13.2 Hz, 2H), 2.86 (ddd, *J* = 13.2, 12.2, 2.7 Hz, 2H), 2.31 (s, 3H), 1.75–1.66 (m, 2H), 1.59 (ddd, *J* = 11.2, 8.8, 5.2 Hz, 1H), 1.20 (dddd, *J* = 15.4, 12.9, 9.6, 4.2 Hz, 3H), 0.99 (d, *J* = 6.5 Hz, 3H). **^13^C NMR (101 MHz, CDCl_3_) δ** 155.2, 136.6, 132.3, 129.3, 120.1, 44.6, 33.9, 30.9, 21.8, 20.7. **HRMS (ESI):** Exact mass calculated for C_14_H_20_N_2_O [M + Na]^+^: 255.1468, found: 255.1476.

**2,6-dimethyl-N-(*p*-tolyl)piperidine-1-carboxamide** (**5e**): General procedure was followed using **1a** (50 mg, 0.37 mmol, 1.0 equiv.), **4e** (84 mg, 0.74 mmol, 2 equiv.), PhI(OAc)_2_ (238 mg, 2 equiv.), K_3_PO_4_ (157 mg, 2 equiv.), 1,2-DCE (2 mL), at 80 °C for 18 h. Column chromatography was performed using 85/15 *v/v* petroleum ether/acetone as eluent and yielded **5e** (39 mg, 42%). Orange solid. mp—149–151 °C. **FTIR (cm^−1^):** 3334, 3031, 2991, 2925, 2588, 1629, 1589, 1508, 1399, 1245, 1068, 805, 787. **^1^H NMR (400 MHz, Chloroform-d) δ** 7.21–7.17 (m, 2H), 7.00 (d, J = 8.4 Hz, 2H), 6.27 (s, 1H), 4.27–4.19 (m, 2H), 2.21 (s, 3H), 1.79–1.60 (m, 4H), 1.60–1.52 (m, 3H), 1.44 (dt, J = 12.3, 3.6 Hz, 1H), 1.22 (s, 3H), 1.20 (s, 3H). **^13^C NMR (101 MHz, CDCl3) δ** 154.9, 136.7, 132.4, 129.5, 129.3, 120.3, 45.6, 30.3, 20.8, 20.7, 13.7. **HRMS (ESI):** Exact mass calculated for C_15_H_23_N_2_O [M + H]^+^: 247.1805, found: 247.1807.

**4-fluoro-N-(*p*-tolyl)piperidine-1-carboxamide** (**5f**): General procedure was followed using **1a** (50 mg, 0.37 mmol, 1.0 equiv.), **4f** (116 mg, 0.74 mmol, 5 equiv.), PhI(OAc)_2_ (238 mg, 2 equiv.), K_3_PO_4_ (157 mg, 2 equiv.), 1,2-DCE (2 mL), at 80 °C for 18 h. Column chromatography was performed using 85/15 *v/v* petroleum ether/acetone as eluent and yielded **5f** (10 mg, 11%). Brown solid. mp—132–134 °C. **FTIR (cm^−1^):** 3318, 2953, 2920, 2854, 1733, 1634, 1594, 1508, 1419, 1222, 1015, 911, 805, 747. **^1^H NMR (400 MHz, CDCl_3_) δ** 7.15 (d, *J* = 8.4 Hz, 2H), 7.02 (d, *J* = 8.4 Hz, 2H), 6.25 (s, 1H), 4.90–4.71 (m, 1H), 3.48 (dd, *J* = 6.4, 5.2 Hz, 5H), 2.23 (s, 4H), 1.92–1.77 (m, 6H). **^13^C NMR (101 MHz, CDCl_3_) δ** 155.0, 136.3, 132.8, 129.4, 120.2, 88.5, 86.8, 40.4 (*J* = 6.0 Hz), 31.1 (*J* = 20.2 Hz), 20.7. **^19^F NMR (376 MHz, CDCl_3_) δ** −182.9, −182.9, −183.0, −183.0, −183.0, −183.0, −183.1, −183.1, −183.1, −183.2, −183.2, −183.2. **HRMS (ESI):** Exact mass calculated for C_13_H_17_FN_2_O [M+Na]^+^: 259.1223, found: 259.1228.

**N-(3,5-bis(trifluoromethyl)phenyl)-4-fluoropiperidine-1-carboxamide** (**5f′**): General procedure was followed using **1′** (95 mg, 0.37 mmol, 1.0 equiv.), **4f** (116 mg, 0.74 mmol, 5 equiv.), PhI(OAc)_2_ (238 mg, 2 equiv.), K_3_PO_4_ (157 mg, 2 equiv.), 1,2-DCE (2 mL), at 80 °C for 18 h. Column chromatography was performed using 85/15 *v/v* petroleum ether/acetone as eluent and yielded **5f’** (97 mg, 73%). Yellow solid. mp—140–142 °C. **FTIR (cm^−1^):** 3378, 3241, 2958, 1642, 1539, 1444, 1368, 1269, 1169, 1119, 1021, 886, 838, 754, 701, 680. **^1^H NMR (400 MHz, CDCl_3_) δ** 7.86 (d, J = 1.6 Hz, 2H), 7.50 (s, 1H), 6.89 (s, 1H), 5.00–4.82 (m, 1H), 3.65 (dt, J = 13.6, 5.0 Hz, 2H), 3.56 (dtd, J = 13.5, 6.6, 6.0, 2.2 Hz, 2H), 1.99–1.83 (m, 4H). **^13^C NMR (101 MHz, CDCl_3_) δ** 153.9, 140.6, 124.5, 121.8, 119.4, 119.3, 116.2, 116.1, 87.9, 86.2, 40.3 (*J* = 5.0 Hz), 31.0 (*J* = 20.2 Hz). **^19^F NMR (376 MHz, CDCl_3_) δ** −63.0, −184.0, −184.1, −184.1, −184.1, −184.1, −184.1, −184.2, −184.2, −184.2, −184.3. **HRMS (ESI):** Exact mass calculated for C_14_H_14_F_7_N_2_O [M + H]^+^: 359.0989, found: 359.1001.

**4-chloro-N-(*p*-tolyl)piperidine-1-carboxamide** (**5g**): General procedure was followed using **1a** (56 mg, 23 mmol, 1.0 equiv.), **4g** (88 mg, 0.74 mmol, 2 equiv.), PhI(OAc)_2_ (238 mg, 2 equiv.), K_3_PO_4_ (157 mg, 2 equiv.), 1,2-DCE (2 mL), at 80 °C for 18 h. Column chromatography was performed using 85/15 *v/v* petroleum ether/acetone as eluent and yielded **5g** (55 mg, 58%). Yellow solid. mp—185–186 °C. **FTIR (cm^−1^):** 3314, 2958, 2914, 1634, 1594, 1510, 1415, 1245, 1041, 1008, 802, 749. **^1^H NMR (400 MHz, CDCl CDCl_3_) δ** 7.23 (d, *J* = 8.4 Hz, 2H), 7.10 (d, *J* = 8.3 Hz, 2H), 6.48 (s, 1H), 4.28 (tt, *J* = 7.4, 3.7 Hz, 1H), 3.74 (ddd, *J* = 13.5, 7.7, 3.6 Hz, 2H), 3.41 (ddd, *J* = 13.6, 7.4, 3.7 Hz, 2H), 2.32 (s, 3H), 2.11 (ddt, *J* = 14.5, 7.5, 3.7 Hz, 2H), 1.89 (dtd, *J* = 14.2, 7.3, 3.5 Hz, 2H). **^13^C NMR (101 MHz, CDCl_3_) δ** 155.1, 136.3, 132.8, 129.4, 120.4, 56.5, 41.6, 34.7, 20.7. **HRMS (ESI):** Exact mass calculated for C_13_H_17_CIN_2_O [M + Na]^+^: 275.0927, found: 275.0933.

**3-hydroxy-N-(*p*-tolyl)piperidine-1-carboxamide** (**5h**): General procedure was followed using **1a** (50 mg, 0.37 mmol, 1.0 equiv.), **4h** (75 mg, 0.74 mmol, 2 equiv.), PhI(OAc)_2_ (238 mg, 2 equiv.), K_3_PO_4_ (157 mg, 2 equiv.), 1,2-DCE (2 mL), at 80 °C for 18 h. Column chromatography was performed using 85/15 *v/v* petroleum ether/acetone as eluent and yielded **5h** (20 mg, 18%). Brown solid. mp—130–132 °C. **^1^H NMR (400 MHz, CDCl_3_) δ** 7.13 (d, J = 8.4 Hz, 2H), 7.01 (d, J = 8.3 Hz, 2H), 6.43 (s, 1H), 3.79 (dt, J = 6.0, 3.4 Hz, 1H), 3.51 (dd, J = 13.4, 3.1 Hz, 1H), 3.37–3.27 (m, 4H), 2.29 (s, 1H), 2.22 (s, 3H), 1.83–1.76 (m, 2H), 1.58 (td, J = 9.2, 8.5, 4.2 Hz, 2H), 1.51–1.42 (m, 1H). **^13^C NMR (101 MHz, CDCl_3_)** δ 156.1, 136.5, 132.5, 129.3, 127.4, 120.2, 65.8, 51.1, 44.9, 32.1, 21.9, 20.7. **HRMS (ESI):** Exact mass calculated for C_13_H_19_N_2_O_2_ [M + H]^+^: 235.1442, found: 235.1441.

***N*-(3,5-bis(trifluoromethyl)phenyl)-3-hydroxypiperidine-1-carboxamide** (**5h′**): General procedure was followed using **1′** (95 mg, 0.37 mmol, 1.0 equiv.), **4h** (75 mg, 0.74 mmol, 2 equiv.), PhI(OAc)_2_ (238 mg, 2 equiv.), K_3_PO_4_ (157 mg, 2 equiv.), 1,2-DCE (2 mL), at 80 °C for 18 h. Column chromatography was performed using 85/15 *v/v* petroleum ether/acetone as eluent and yielded **5h’** (36 mg, 27%). Yellow solid. mp—188–190 °C. **FTIR (cm^−1^):** 3342, 2932, 2866, 2441, 1626, 1565, 1479, 1444, 1394, 1272, 1128, 999, 928, 883, 698, 678. **^1^H NMR (400 MHz, CDCl_3_) δ** 7.94 (s, 2H), 7.41–7.39 (m, 1H), 3.80 (dd, *J* = 13.1, 3.8 Hz, 1H), 3.60 (ddd, *J* = 7.7, 4.7, 2.5 Hz, 2H), 3.21 (p, *J* = 1.6 Hz, 1H), 3.19–3.10 (m, 1H), 3.00 (dd, *J* = 13.1, 8.0 Hz, 1H), 1.94–1.82 (m, 1H), 1.75 (dq, *J* = 6.2, 3.2, 2.8 Hz, 1H), 1.49–1.39 (m, 2H), 1.18 (s, 1H). **^13^C NMR (101 MHz, CDCl_3_)** δ 155.6, 142.3, 131.9, 131.6, 131.3, 130.9, 124.8, 122.1, 119.2, 114.5, 114.4, 65.5, 50.4, 44.1, 32.2, 22.3. **HRMS (ESI):** Exact mass calculated for C_14_H_15_F_6_N_2_O_2_ [M + H]^+^: 357.1033, found: 357.1032.

**4-hydroxy-*N*-(*p*-tolyl)piperidine-1-carboxamide** (**5i**): General procedure was followed using **1a** (50 mg, 0.37 mmol, 1.0 equiv.), **4i** (75 mg, 0.74 mmol, 2 equiv.), PhI(OAc)_2_ (238 mg, 2 equiv.), K_3_PO_4_ (157 mg, 2 equiv.), 1,2-DCE (2 mL), at 80 °C for 18 h. Column chromatography was performed using 85/15 *v/v* petroleum ether/acetone as eluent and yielded **5i** (38 mg, 45%). Yellow solid. mp—128–130 °C. **FTIR (cm^−1^):** 3307, 3024, 2931, 2865, 1634, 1596, 1528, 1419, 1357, 1309, 1242, 1116, 1050, 970, 809, 743. **^1^H NMR (400 MHz, CDCl_3_) δ** 7.22 (d, *J* = 8.4 Hz, 2H), 7.09 (d, *J* = 8.3 Hz, 2H), 6.50 (s, 1H), 3.94–3.79 (m, 3H), 3.16 (ddd, *J* = 13.1, 9.3, 3.4 Hz, 2H), 2.31 (s, 4H), 1.91 (dqd, *J* = 13.1, 3.9, 2.1 Hz, 2H), 1.56 (dtd, *J* = 12.9, 8.8, 3.9 Hz, 2H). **^13^C NMR (101 MHz, CDCl_3_) δ** 155.3, 136.4, 132.7, 129.3, 120.3, 67.2, 41.7, 33.9, 20.7. **HRMS (ESI):** Exact mass calculated for C_13_H_18_N_2_O_2_ [M+Na]^+^: 257.1261, found: 257.1270.

***tert*-butyl (1-(*p*-tolylcarbamoyl)piperidin-4-yl)carbamate** (**5j**): General procedure was followed using **1a** (56 mg, 23 mmol, 1.0 equiv.), **4j** (148 mg, 0.74 mmol, 2 equiv.), PhI(OAc)_2_ (238 mg, 2 equiv.), K_3_PO_4_ (157 mg, 2 equiv.), 1,2-DCE (2 mL), at 80 °C for 18 h. Column chromatography was performed using 85/15 *v/v* petroleum ether/acetone as eluent and yielded **5j** (46 mg, 38%). Colorless solid. mp—205–207 °C. **FTIR (cm^−1^):** 3349, 2980, 2925, 2888, 1682, 1629, 1516, 1417, 1318, 1225, 1152, 1041, 1021, 807, 743. **^1^H NMR (400 MHz, CDCl_3_) δ** 7.23 (d, *J* = 8.4 Hz, 2H), 7.09 (d, *J* = 8.4 Hz, 2H), 6.49 (s, 1H), 4.53 (s, 1H), 4.00 (d, *J* = 13.4 Hz, 2H), 3.65 (s, 1H), 2.98 (ddd, *J* = 14.0, 11.6, 2.8 Hz, 2H), 2.30 (s, 4H), 2.03–1.93 (m, 2H), 1.47 (s, 11H), 1.44–1.31 (m, 3H), 0.93–0.85 (m, 1H). **^13^C NMR (101 MHz, CDCl_3_) δ** 155.1, 155.1, 136.4, 132.6, 129.3, 120.3, 79.5, 47.8, 43.2, 32.3, 28.4, 20.7. **HRMS (ESI):** Exact mass calculated for C_18_H_27_N_3_O_3_ [M+Na]^+^: 356.1950, found: 356.1959.

***tert*-butyl(1-((3,5-bis(trifluoromethyl)phenyl)carbamoyl)piperidin-4-yl)carbamate** (**5j’**): General procedure was followed using **1′** (95 mg, 23 mmol, 1.0 equiv.), **4j** (148 mg, 0.74 mmol, 2 equiv.), PhI(OAc)_2_ (238 mg, 2 equiv.), K_3_PO_4_ (157 mg, 2 equiv.), 1,2-DCE (2 mL), at 80 °C for 18 h. Column chromatography was performed using 85/15 *v/v* petroleum ether/acetone as eluent and yielded **5j’** (110 mg, 66%). Colorless solid. mp—225–227 °C. **FTIR (cm^−1^):** 3380, 3208, 1660, 1572, 1539, 1472, 1318, 1276, 1227, 1123, 1048, 871, 698. **^1^H NMR (400 MHz, DMSO-*d_6_*) δ** 9.23 (s, 1H), 8.27 (s, 2H), 6.94 (d, J = 7.9 Hz, 1H), 4.10 (d, J = 13.9 Hz, 2H), 3.37 (d, J = 9.5 Hz, 3H), 2.99 (ddd, J = 14.0, 11.6, 2.7 Hz, 2H), 1.87–1.78 (m, 2H), 1.45 (d, J = 3.2 Hz, 9H). **^13^C NMR (101 MHz, DMSO-*d*_6_) δ** 155.2, 154.4, 143.3, 130.9, 130.6, 122.5, 119.0, 78.0, 47.6, 43.1, 32.2, 28.7. **HRMS (ESI):** Exact mass calculated for C_19_H_24_F_6_N_3_O_3_ [M + H]^+^: 456.1717, found: 456.1712.

**4-cyano-*N*-(p-tolyl)piperidine-1-carboxamide** (**5k**): General procedure was followed using **1a** (50 mg, 0.37 mmol, 1.0 equiv.), **4k** (81 mg, 0.74 mmol, 2 equiv.), PhI(OAc)_2_ (238 mg, 2 equiv.), K_3_PO_4_ (157 mg, 2 equiv.), 1,2-DCE (2 mL), at 80 °C for 18 h. Column chromatography was performed using 85/15 *v/v* petroleum ether/acetone as eluent and yielded **5k** (55 mg, 62%). Yellow solid. mp—103–109 °C. **FTIR (cm^−1^):** 3314, 2953, 2925, 2869, 2237, 1629, 1589, 1512, 1413, 1284, 1240, 1017, 975, 800, 747. **^1^H NMR (400 MHz, CDCl_3_) δ** 7.22 (d, *J* = 8.4 Hz, 2H), 7.12 (d, *J* = 8.0 Hz, 2H), 6.37 (s, 1H), 3.68 (dd, *J* = 10.1, 4.4 Hz, 2H), 3.51–3.40 (m, 2H), 2.90 (q, *J* = 3.8 Hz, 1H), 2.32 (s, 3H), 2.04–1.83 (m, 5H), 1.64 (s, 1H). **^13^C NMR (101 MHz, CDCl_3_) δ** 155.0, 136.0, 136.0, 133.1, 129.4, 129.3, 127.4, 120.8, 120.4, 120.4, 42.4, 28.3, 26.2, 20.7. **HRMS (ESI):** Exact mass calculated for C_14_H_17_N_3_O [M + Na]^+^: 266.1269, found: 266.1274.

***N*-(*p*-tolyl)-1,4-dioxa-8-azaspiro[4,5]decane-8-carboxamide** (**5l**): General procedure was followed using **1a** (50 mg, 0.37 mmol, 1.0 equiv.), **4l** (138 mg, 0.74 mmol, 2 equiv.), PhI(OAc)_2_ (238 mg, 2 equiv.), K_3_PO_4_ (157 mg, 2 equiv.), 1,2-DCE (2 mL), at 80 °C for 18 h. Column chromatography was performed using 85/15 *v/v* petroleum ether/acetone as eluent and yielded **5l** (66 mg, 65%). Colorless solid. mp—158–160 °C. **FTIR (cm^−1^):** 3316, 2964, 2880, 1638, 1594, 1521, 1479, 1309, 1225, 1101, 1032, 944, 902, 805, 742. **^1^H NMR (400 MHz, CDCl_3_) δ** 7.21 (d, *J* = 8.4 Hz, 2H), 7.09–7.04 (m, 2H), 6.50 (s, 1H), 3.97 (s, 4H), 3.58–3.52 (m, 4H), 2.28 (s, 3H), 1.75–1.69 (m, 4H). **^13^C NMR (101 MHz, CDCl_3_) δ** 155.0, 136.5, 132.6, 129.3, 120.3, 106.9, 64.4, 42.5, 34.9, 20.7. **HRMS (ESI):** Exact mass calculated for C_15_H_21_N_2_O_3_ [M + H]^+^: 277.1547, found: 277.1546.

**Methyl-1-((3,5-bis(trifluoromethyl)phenyl)carbamoyl)piperidine-3-carboxylate** (**5m**): General procedure was followed using **1’** (95 mg, 23 mmol, 1.0 equiv.), **4m** (105 mg, 0.74 mmol, 2 equiv.), PhI(OAc)_2_ (238 mg, 2 equiv.), K_3_PO_4_ (157 mg, 2 equiv.), 1,2-DCE (2 mL), at 80 °C for 18 h. Column chromatography was performed using 85/15 *v/v* petroleum ether/acetone as eluent and yielded **5m** (133 mg, 90%). Yellow solid. mp—82–84 °C. **FTIR (cm^−1^):** 3338, 2958, 1726, 1642, 1556, 1472, 1441, 1373, 1269, 1165, 1110, 1035, 984, 937, 879, 681. **^1^H NMR (400 MHz, CDCl_3_) δ** 8.15 (s, 1H), 7.81 (s, 2H), 7.43 (s, 1H), 3.76 (s, 4H), 3.73–3.68 (m, 1H), 3.64 (dd, J = 14.1, 3.5 Hz, 1H), 3.30 (td, J = 9.0, 8.4, 4.0 Hz, 1H), 2.71–2.65 (m, 1H), 2.14–2.04 (m, 1H), 1.95 (ddt, J = 13.6, 9.0, 4.5 Hz, 1H), 1.58 (dtt, J = 17.4, 8.8, 3.9 Hz, 2H). **^13^C NMR (101 MHz, CDCl_3_) δ** 174.6, 154.6, 141.4, 132.3, 131.9, 131.6, 131.3, 124.6, 121.9, 119.2, 119.1, 119.0, 115.4, 115.3, 52.3, 46.6, 44.2, 40.6, 26.5, 23.7. **HRMS (ESI):** Exact mass calculated for C_16_H_17_F_6_N_2_O [M + H]^+^: 399.1138, found: 399.1140.

**Methyl-1-(*p*-tolylcarbamoyl)piperidine-4-carboxylate** (**5n**): General procedure was followed using **1a** (56 mg, 23 mmol, 1.0 equiv.), **4n** (105 mg, 0.74 mmol, 2 equiv.), PhI(OAc)_2_ (238 mg, 2 equiv.), K_3_PO_4_ (157 mg, 2 equiv.), 1,2-DCE (2 mL), at 80 °C for 18 h. Column chromatography was performed using 85/15 *v/v* petroleum ether/acetone as eluent and yielded **5n** (57 mg, 55%). Yellow solid. mp—110–112 °C. **FTIR (cm^−1^):** 3294, 2949, 2854, 1726, 1638, 1594, 1512, 1413, 1225, 1030, 970, 802,747. **^1^H NMR (400 MHz, CDCl_3_) δ** 7.25–7.21 (m, 2H), 7.11–7.07 (m, 2H), 6.52 (s, 1H), 4.03–3.96 (m, 2H), 3.72 (s, 3H), 2.99 (ddd, *J* = 13.8, 11.2, 3.0 Hz, 2H), 2.53 (tt, *J* = 10.8, 4.0 Hz, 1H), 2.30 (s, 3H), 1.95 (dt, *J* = 12.8, 4.0 Hz, 2H), 1.78–1.68 (m, 2H). **^13^C NMR (101 MHz, CDCl_3_) δ** 174.7, 155.2, 136.4, 132.6, 129.3, 120.3, 51.8, 43.6, 40.8, 27.8, 20.7. **HRMS (ESI):** Exact mass calculated for C_15_H_20_N_2_O_3_ [M+Na]^+^: 299.1367, found: 299.1370.

**4-phenyl-*N*-(*p*-tolyl)piperidine-1-carboxamide** (**5o**): General procedure was followed using **1a** (50 mg, 0.37 mmol, 1.0 equiv.), **4o** (119 mg, 0.74 mmol, 2 equiv.), PhI(OAc)_2_ (238 mg, 2 equiv.), K_3_PO_4_ (157 mg, 2 equiv.), 1,2-DCE (2 mL), at 80 °C for 18 h. Column chromatography was performed using 85/15 *v/v* petroleum ether/acetone as eluent and yielded **5o** (50 mg, 46%). Yellow solid. mp—154–155 °C. **^1^H NMR (400 MHz, CDCl_3_)** δ 7.35 (t, *J* = 7.4 Hz, 3H), 7.31–7.20 (m, 8H), 7.12 (d, *J* = 8.3 Hz, 2H), 6.48 (s, 1H), 4.24 (dt, *J* = 13.3, 2.3 Hz, 2H), 3.00 (td, *J* = 13.0, 2.6 Hz, 2H), 2.74 (ddd, *J* = 12.1, 8.5, 3.6 Hz, 1H), 2.32 (s, 4H), 1.98–1.90 (m, 3H), 1.82–1.69 (m, 3H). **^13^C NMR (101 MHz, CDCl_3_)** δ 154.2, 144.3, 135.4, 131.5, 128.3, 127.5, 125.7, 119.2, 44.0, 41.6, 32.0, 19.7. **HRMS (ESI):** Exact mass calculated for C_19_H_22_N_2_O [M+Na]^+^: 317.1625, found: 317.1629.

**4-benzyl-*N*-(*p*-tolyl)piperidine-1-carboxamide** (**5p**): General procedure was followed using **1a** (50 mg, 0.37 mmol, 1.0 equiv.), **4p** (130 mg, 0.74 mmol, 2 equiv.), PhI(OAc)_2_ (238 mg, 2 equiv.), K_3_PO_4_ (157 mg, 2 equiv.), 1,2-DCE (2 mL), at 80 °C for 18 h. Column chromatography was performed using 85/15 *v/v* petroleum ether/acetone as eluent and yielded **5p** (59 mg, 52%). Yellow solid. mp—148–150 °C. **FTIR (cm^−1^):** 3305, 3024, 2914, 2843, 1629, 1587, 1508, 1417, 1287, 1240, 1052, 906, 802, 745. **^1^H NMR (400 MHz, CDCl_3_)** δ 7.20 (dd, *J* = 8.0, 6.6 Hz, 2H), 7.17–7.09 (m, 4H), 7.05 (d, *J* = 6.7 Hz, 2H), 6.97 (d, *J* = 8.3 Hz, 2H), 6.38 (s, 1H), 3.94 (d, *J* = 13.3 Hz, 2H), 2.68 (td, *J* = 12.8, 2.5 Hz, 2H), 2.46 (d, *J* = 6.9 Hz, 2H), 2.20 (s, 3H), 1.68–1.55 (m, 3H), 1.13 (qd, *J* = 12.6, 4.0 Hz, 2H). **^13^C NMR** (101 MHz, CDCl_3_) δ 155.2, 140.0, 136.7, 132.3, 129.3, 129.1, 128.3, 126.0, 120.2, 44.6, 43.0, 38.1, 31.9, 20.7. **HRMS (ESI):** Exact mass calculated for C_20_H_24_N_2_O [M+Na]^+^: 331.1786, found: 331.1789.

**1,1-dimethyl-3-(*p*-tolyl)urea** (**5q**): General procedure was followed using **1a** (50 mg, 0.37 mmol, 1.0 equiv.), **4q** (66 mg, 74 μL, 1.48 mmol, 4 equiv.), PhI(OAc)_2_ (238 mg, 2 equiv.), K_3_PO_4_ (157 mg, 2 equiv.), 1,2-DCE (2 mL), at 80 °C for 18 h. Column chromatography was performed using 85/15 *v/v* petroleum ether/acetone as eluent and yielded **5q** (30 mg, 45%). Yellow solid. mp—152–154 °C. **FTIR (cm^−1^):** 3336, 3020, 2914, 1640, 1594, 1508, 1483, 1362, 1295, 1234, 1183, 1072, 1028, 814, 749. **^1^H NMR (400 MHz, CDCl_3_)** δ 7.21–7.15 (m, 2H), 7.03–6.98 (m, 2H), 6.22 (s, 1H), 2.93 (s, 6H), 2.22 (s, 3H). **^13^C NMR (101 MHz, CDCl_3_)** δ 155.9, 136.6, 132.4, 129.3, 120.0, 36.4, 20.7. **HRMS (ESI):** Exact mass calculated for C_10_H_14_N_2_O [M+Na]^+^: 201.1004, found: 201.1008.

**1,1-dimethyl-3-(*p*-tolyl)urea** (**5q**): General procedure was followed using **1a** (1.5 g, 0.37 mmol, 1.0 equiv.), **4q** (3 mL, 4 equiv.), PhI(OAc)_2_ (7.144g, 2 equiv.), K_3_PO_4_ (4.7g, 2 equiv.), 1,2-DCE (2 mL), at 80 °C for 18 h. Column chromatography was performed using 85/15 *v/v* petroleum ether/acetone as eluent and yielded **5q** (687 mg, 35%). Yellow solid. mp—152–154 °C. **FTIR (cm^−1^):** 3336, 3020, 2914, 1640, 1594, 1508, 1483, 1362, 1295, 1234, 1183, 1072, 1028, 814, 749. **^1^H NMR (400 MHz, CDCl_3_)** δ 7.21–7.15 (m, 2H), 7.03–6.98 (m, 2H), 6.22 (s, 1H), 2.93 (s, 6H), 2.22 (s, 3H). **^13^C NMR** (101 MHz, CDCl_3_) δ 155.9, 136.6, 132.4, 129.3, 120.0, 36.4, 20.7. **HRMS (ESI):** Exact mass calculated for C_10_H_14_N_2_O [M+Na]^+^: 201.1004, found: 201.1008.

**1,1-diethyl-3-(*p*-tolyl)urea** (**5r**): General procedure was followed using **1a** (50 mg, 0.37 mmol, 1.0 equiv.), **4r** (54 mg, 0.74 mmol, 2 equiv.), PhI(OAc)_2_ (238 mg, 2 equiv.), K_3_PO_4_ (157 mg, 2 equiv.), 1,2-DCE (2 mL), at 80 °C for 18 h. Column chromatography was performed using 85/15 *v/v* petroleum ether/acetone as eluent and yielded **5r** (29 mg, 39%). Brown solid. mp—50–51 °C. **FTIR (cm^−1^):** 3331, 2975, 2931, 2872, 1629, 1594, 1510, 1483, 1413, 1287, 1236, 1158, 1074, 807, 780, 749. **^1^H NMR (400 MHz, CDCl_3_) δ** 7.22–7.16 (m, 3H), 7.04–6.97 (m, 2H), 6.15 (s, 1H), 3.29 (q, *J* = 7.2 Hz, 5H), 2.21 (s, 4H), 1.14 (t, *J* = 7.1 Hz, 7H). **^13^C NMR (101 MHz, CDCl_3_) δ** 154.7, 136.7, 132.2, 129.3, 120.0, 41.6, 20.7, 13.9. **HRMS (ESI):** Exact mass calculated for C_12_H_18_N_2_O [M+Na]^+^: 229.1317, found: 229.1322.

**1,1-diisopropyl-3-(*p*-tolyl)urea** (**5s**): General procedure was followed using **1a** (50 mg, 0.37 mmol, 1.0 equiv.), **4s** (75 mg, 0.74 mmol, 2 equiv.), PhI(OAc)_2_ (238 mg, 2 equiv.), K_3_PO_4_ (157 mg, 2 equiv.), 1,2-DCE (2 mL), at 80 °C for 18 h. Column chromatography was performed using 85/15 *v/v* petroleum ether/acetone as eluent and yielded **5s** (32 mg, 37%). Yellow solid. mp—136–138 °C. **FTIR (cm^−1^):** 3318, 2964, 2931, 2865, 1627, 1594, 1508, 1424, 1324, 1236, 1141, 1052, 800, 747. **^1^H NMR (400 MHz, CDCl_3_) δ** 7.18 (d, *J* = 8.6 Hz, 2H), 7.00 (d, *J* = 8.3 Hz, 2H), 6.06 (s, 1H), 3.90 (hept, *J* = 6.9 Hz, 2H), 2.21 (s, 3H), 1.25 (s, 6H), 1.23 (s, 6H). **^13^C NMR (101 MHz, CDCl_3_) δ** 154.8, 136.7, 132.1, 129.3, 119.8, 45.4, 21.5, 20.7. **HRMS (ESI):** Exact mass calculated for C_14_H_23_N_2_O [M + H]^+^: 235.1805, found: 235.1807.

**1,1-dibutyl-3-(*p*-tolyl)urea** (**5t**): General procedure was followed using **1a** (50 mg, 0.37 mmol, 1.0 equiv.), **4t** (96 mg, 0.74 mmol, 2 equiv.), PhI(OAc)_2_ (238 mg, 2 equiv.), K_3_PO_4_ (157 mg, 2 equiv.), 1,2-DCE (2 mL), at 80 °C for 18 h. Column chromatography was performed using 85/15 *v/v* petroleum ether/acetone as eluent and yielded **5t** (15 mg, 40%). Yellow solid. mp—76–78 °C. **FTIR (cm^−1^):** 3321, 2959, 2931, 2851, 1634, 1592, 1512, 1483, 1417, 1310, 1286, 1220, 1104, 936, 814, 750. **^1^H NMR (400 MHz, CDCl_3_) δ** 7.21–7.16 (m, 3H), 7.03–6.98 (m, 2H), 6.12 (s, 1H), 3.25–3.17 (m, 4H), 2.22 (s, 3H), 1.52 (tt, *J* = 7.6, 6.4 Hz, 5H), 1.29 (dq, *J* = 14.6, 7.3 Hz, 5H), 0.89 (t, *J* = 7.3 Hz, 7H). ^13^C NMR (101 MHz, CDCl_3_) δ 154.0, 135.6, 131.2, 128.2, 118.8, 46.4, 29.8, 19.6, 19.2, 12.8. **HRMS (ESI):** Exact mass calculated for C_16_H_27_N_2_O [M + H]^+^: 263.2118, found: 263.2120.

**1,1-diisobutyl-3-(*p*-tolyl)urea** (**5u**): General procedure was followed using **1a** (50 mg, 0.37 mmol, 1.0 equiv.), **4u** (96 mg, 0.74 mmol, 2 equiv.), PhI(OAc)_2_ (238 mg, 2 equiv.), K_3_PO_4_ (157 mg, 2 equiv.), 1,2-DCE (2 mL), at 80 °C for 18 h. Column chromatography was performed using 85/15 *v/v* petroleum ether/acetone as eluent and yielded **5u** (17 mg, 18%). Yellow solid. mp—93–95 °C. **FTIR (cm^−1^):** 3323, 2957, 2920, 2864, 1638, 1594, 1512, 1483, 1403, 1379, 1306, 1288, 1231, 1166, 1100, 1056, 810, 756. **^1^H NMR (400 MHz, CDCl_3_) δ** 7.25 (d, *J* = 7.7 Hz, 3H), 7.08 (d, *J* = 8.3 Hz, 2H), 6.22 (s, 1H), 3.14 (d, *J* = 7.5 Hz, 4H), 2.29 (s, 3H), 2.03 (dq, *J* = 13.8, 6.9 Hz, 3H), 0.94 (d, *J* = 6.7 Hz, 12H). **^13^C NMR (101 MHz, CDCl_3_) δ** 155.5, 136.6, 132.2, 129.3, 119.8, 56.0, 27.8, 20.7, 20.3. **HRMS (ESI):** Exact mass calculated for C_16_H_26_N_2_O [M + H]^+^: 263.2118, found: 263.2120.

**3-(3,5-bis(trifluoromethyl)phenyl)-1,1-diisobutylurea** (**5u′**): General procedure was followed using **1′** (95 mg, 0.37 mmol, 1.0 equiv.), **4u** (96 mg, 0.74 mmol, 2 equiv.), PhI(OAc)_2_ (238 mg, 2 equiv.), K_3_PO_4_ (157 mg, 2 equiv.), 1,2-DCE (2 mL), at 80 °C for 18 h. Column chromatography was performed using 85/15 *v/v* petroleum ether/acetone as eluent and yielded **5u’** (60 mg, 43%). Yellow solid. mp—124–126 °C. **FTIR (cm^−1^):** 3316, 2969, 2876, 1638, 1534, 1468, 1444, 1373, 1271, 1119, 997, 948, 877, 838, 700, 681. **^1^H NMR (400 MHz, CDCl3) δ** 7.88–7.83 (m, 2H), 7.45 (s, 1H), 6.85 (s, 1H), 3.18 (d, *J* = 7.5 Hz, 4H), 2.03 (dq, *J* = 13.9, 7.0 Hz, 2H), 0.95 (d, *J* = 6.7 Hz, 12H). **^13^C NMR (101 MHz, CDCl_3_) δ** 154.7, 140.8, 132.4, 132.1, 131.7, 131.4, 127.3, 124.5, 121.8, 119.3, 115.8, 115.7, 55.9, 27.6, 20.1. **^19^F NMR (376 MHz, CDCl_3_)** δ −63.0. **HRMS (ESI):** Exact mass calculated for C_17_H_23_F_6_N_2_O [M + H]^+^: 385.1710, found: 385.1713.

**1,1-dicyclohexyl-3-(*p*-tolyl)urea** (**5v**): General procedure was followed using **1a** (50 mg, 0.37 mmol, 1.0 equiv.), **4v** (134 mg, 0.74 mmol, 2 equiv.), PhI(OAc)_2_ (238 mg, 2 equiv.), K_3_PO_4_ (157 mg, 2 equiv.), 1,2-DCE (2 mL), at 80 °C for 18 h. Column chromatography was performed using 85/15 *v/v* petroleum ether/acetone as eluent and yielded **5v** (70 mg, 60%). Brown solid. mp—157–159 °C. FTIR (cm^−1^): 3318, 2920, 2847, 1629, 1594, 1510, 1357, 1311, 1236, 1150, 1001, 813, 816, 745. **^1^H NMR (400 MHz, CDCl3) δ** 7.17 (d, *J* = 8.4 Hz, 2H), 6.99 (d, *J* = 8.3 Hz, 2H), 6.13 (s, 1H), 3.39 (tt, *J* = 10.8, 5.0 Hz, 2H), 2.21 (s, 3H), 1.77 (d, *J* = 3.4 Hz, 2H), 1.74 (dd, *J* = 6.9, 2.9 Hz, 4H), 1.68 (dt, *J* = 8.1, 3.6 Hz, 7H), 1.63–1.56 (m, 3H), 1.33–1.22 (m, 5H), 1.08 (tt, *J* = 13.2, 3.4 Hz, 2H). **^13^C NMR (101 MHz, CDCl_3_) δ** 155.0, 136.8, 132.0, 129.3, 119.7, 55.4, 31.9, 26.4, 25.5, 20.7. **HRMS (ESI):** Exact mass calculated for C_20_H_31_N_2_O [M + H]^+^: 315.2431, found: 315.2439.

***N*-(*p*-tolyl)pyrrolidine-1-carboxamide** (**5w**): General procedure was followed using **1a** (50 mg, 0.37 mmol, 1.0 equiv.), **4w** (53 mg, 0.74 mmol, 2 equiv.), PhI(OAc)_2_ (238 mg, 2 equiv.), K_3_PO_4_ (157 mg, 2 equiv.), 1,2-DCE (2 mL), at 80 °C for 18 h. Column chromatography was performed using 85/15 *v/v* petroleum ether/acetone as eluent and yielded **5w** (16 mg, 26%). Yellow solid. mp—147–149 °C. **FTIR (cm^−1^):** 3316, 3035, 2971, 2869, 1640, 1589, 1508, 1406, 1375, 1287, 1242, 1114, 1032, 805, 752. **^1^H NMR (400 MHz, CDCl_3_) δ** 7.31 (d, *J* = 8.4 Hz, 2H), 7.10 (d, *J* = 8.3 Hz, 2H), 6.16 (s, 1H), 3.49–3.45 (m, 4H), 2.31 (s, 3H), 1.99–1.95 (m, 4H). **^13^C NMR (101 MHz, CDCl_3_) δ** 153.1, 135.5, 131.2, 128.3, 118.7, 44.7, 24.5, 19.7. **HRMS (ESI):** Exact mass calculated for C_12_H_16_N_2_O [M+Na]^+^: 227.1155, found: 227.1165.

***N*-(3,5-bis(trifluoromethyl)phenyl)pyrrolidine-1-carboxamide** (**5w’**): General procedure was followed using **1’** (95 mg, 0.37 mmol, 1.0 equiv.), **4w**(53 mg, 0.74 mmol, 2 equiv.), PhI(OAc)_2_ (238 mg, 2 equiv.), K_3_PO_4_ (157 mg, 2 equiv.), 1,2-DCE (2 mL), at 80 °C for 18 h. Column chromatography was performed using 85/15 *v/v* petroleum ether/acetone as eluent and yielded **5w’** (48 mg, 40%). Pale yellow solid. mp—145–147 °C. **FTIR (cm^−1^):** 3267, 2958, 2876, 1722, 1649, 1541, 1472, 1441, 1362, 1273, 1163, 1123, 1080, 875, 701, 679. **^1^H NMR (400 MHz, CDCl_3_) δ** 7.85 (d, J = 1.6 Hz, 2H), 7.39 (s, 1H), 6.67 (s, 1H), 3.45–3.37 (m, 4H), 1.95–1.87 (m, 4H). **^13^C NMR (101 MHz, CDCl_3_) δ** 13C NMR (101 MHz, CDCl3) δ 152.2, 139.7, 131.4, 131.1, 130.7, 130.4, 123.5, 120.8, 118.0, 117.9, 44.9, 24.5. ^19^F NMR (376 MHz, CDCl3) δ −63.08. **HRMS (ESI):** Exact mass calculated for C_13_H_13_F_6_N_2_O_2_ [M + H]^+^: 327.0927, found: 327.0930.

**2,6-dimethyl-*N*-(*p*-tolyl)morpholine-4-carboxamide** (**5x**): General procedure was followed using **1a** (50 mg, 0.37 mmol, 1.0 equiv.), **4x** (85 mg, 0.74 mmol, 2 equiv.), PhI(OAc)_2_ (238 mg, 2 equiv.), K_3_PO_4_ (157 mg, 2 equiv.), 1,2-DCE (2 mL), at 80 °C for 18 h. Column chromatography was performed using 85/15 *v/v* petroleum ether/acetone as eluent and yielded **5x** (39 mg, 42%). Orange solid. mp—144–145 °C. **FTIR (cm^−1^):** 3238, 3112, 3042, 2971, 2808, 1623, 1594, 1512, 1413, 1247, 1167, 1081, 1052, 816, 725. **^1^H NMR (400 MHz, CDCl_3_) δ** 7.14 (d, *J* = 8.4 Hz, 2H), 7.00 (d, *J* = 8.2 Hz, 2H), 6.41 (s, 1H), 3.76 (dd, *J* = 13.2, 2.1 Hz, 2H), 3.51 (dtd, *J* = 12.5, 6.2, 2.5 Hz, 2H), 2.50 (dd, *J* = 12.8, 10.6 Hz, 2H), 2.22 (s, 3H), 2.09 (s, 1H), 1.12 (d, *J* = 6.3 Hz, 6H). **^13^C NMR (101 MHz, CDCl_3_) δ** 155.0, 136.2, 132.8, 129.3, 120.4, 71.6, 49.4, 30.9, 20.7, 18.7. **HRMS (ESI):** Exact mass calculated for C_14_H_21_N_2_O_2_ [M + H]^+^: 249.1598, found: 249.1600.

**N-(*p*-tolyl)azetidine-1-carboxamide** (**5y**): General procedure was followed using **1a** (50 mg, 0.37 mmol, 1.0 equiv.), **4y** (42 mg, 50 μL, 0.74 mmol, 2 equiv.), PhI(OAc)_2_ (238 mg, 2 equiv.), K_3_PO_4_ (157 mg, 2 equiv.), 1,2-DCE (2 mL), at 80 °C for 18 h. Column chromatography using 85/15 *v*/*v* petroleum ether/acetone yielded **5y** (14 mg, 19%). Brown solid. mp—211–212 °C. **FTIR (cm^−1^):** 3227, 3112, 3009, 2960, 2887, 1642, 1596, 1510, 1402, 1364, 1284, 1245, 1099, 800. **^1^H NMR (400 MHz, CDCl_3_) δ** 7.23–7.16 (m, 3H), 7.00 (d, *J* = 8.4 H3, 2H), 5.85 (s, 1H), 3.98 (t, *J* = 7.5 Hz, 4H), 2.22 (s, 3H), 2.21–2.16 (m, 2H). **^13^C NMR (101 MHz, CDCl_3_) δ** 156.5, 136.0, 132.4, 129.4, 119.5, 49.2, 20.7, 15.1. **HRMS (ESI):** Exact mass calculated for C_11_H_15_N_2_O [M + H]^+^: 191.1179, found: 191.1180.

***N*-(3,5-bis(trifluoromethyl)phenyl)azetidine-1-carboxamide** (**5y′**): General procedure was followed using **1′** (95 mg, 0.37 mmol, 1.0 equiv.), **4y** (42 mg, 50 μL, 0.74 mmol, 2 equiv.), PhI(OAc)_2_ (238 mg, 2 equiv.), K_3_PO_4_ (157 mg, 2 equiv.), 1,2-DCE (2 mL), at 80 °C for 18 h. Column chromatography was performed using 85/15 *v/v* petroleum ether/acetone as eluent and yielded **5y′** (97 mg, 60%). Colorless solid. mp—161–163 °C. **FTIR (cm^−1^):** 3278, 2958, 2887, 1654, 1545, 1441, 1362, 1271, 1165, 1119, 1001, 970, 937, 882, 701, 680. **^1^H NMR (400 MHz, CDCl_3_) δ** 7.91 (d, J = 0.9 Hz, 2H), 7.49–7.44 (m, 1H), 6.65 (s, 1H), 4.14–4.08 (m, 4H), 2.39–2.28 (m, 2H). **^13^C NMR (101 MHz, CDCl_3_) δ** 155.3, 140.4, 132.5, 132.2, 131.9, 131.6, 124.5, 121.8, 118.6, 115.9, 115.8, 49.3, 15.1. **HRMS (ESI):** Exact mass calculated for C_12_H_11_F_6_N_2_O [M + H]^+^: 313.0771, found: 313.0772.

***N*-(*p*-tolyl)-2-oxa-6-azaspiro[3,3]heptane-6-carboxamide** (**5z**): General procedure was followed using **1a** (50 mg, 0.37 mmol, 1.0 equiv.), **4z** (73 mg, 0.74 mmol, 2 equiv.), PhI(OAc)_2_ (238 mg, 2 equiv.), K_3_PO_4_ (157 mg, 2 equiv.), 1,2-DCE (2 mL), at 80 °C for 18 h. Column chromatography was performed using 85/15 *v/v* petroleum ether/acetone as eluent and yielded **5z** (33 mg, 38%). Colorless solid. mp—173–175 °C. **FTIR (cm^−1^):** 3287, 2928, 2862, 1647, 1596, 1532, 1508, 1406, 1368, 1333, 1244, 1087, 969, 812, 670. **^1^H NMR (400 MHz, CDCl_3_) δ** 7.31–7.20 (m, 3H), 7.08 (d, *J* = 8.3 Hz, 2H), 6.18 (s, 1H), 4.77 (s, 4H), 4.15 (s, 4H), 2.29 (s, 3H). **^13^C NMR (101 MHz, CDCl_3_) δ** 156.57, 135.73, 133.00, 129.47, 129.28, 127.42, 119.98, 80.89, 58.89, 37.67, 21.50, 20.77. **HRMS (ESI):** Exact mass calculated for C_13_H_17_N_2_O_2_ [M + H]^+^: 233.1285, found: 233.1287.

**4-((*N*-cyclopropyl-3-(trifluoromethyl)phenyl)sulfonamido)-*N*-(p-tolyl)piperidine-1-carboxamide** (**5aa**): General procedure was followed using **1a** (77 mg, 0.50 mmol, 2.0 equiv.), **4aa** (100 mg, 0.28 mmol, 1 equiv.), PhI(OAc)_2_ (180 mg, 2 equiv.), K_3_PO_4_ (120 mg, 2 equiv.), 1,2-DCE (2 mL), at 80 °C for 18 h. Column chromatography was performed using 85/15 *v/v* petroleum ether/acetone as eluent and yielded **5aa** (67 mg, 38%). Colorless solid. mp—155–157 °C. **FTIR (cm^−1^):** 3342, 1641, 1598, 1513, 1481, 1423, 1326, 1241, 1162, 1124, 1070, 975, 928, 881, 811, 696, 647. **^1^H NMR (400 MHz, CDCl_3_) δ** 8.05 (d, *J* = 1.9 Hz, 1H), 7.98 (dt, *J* = 7.9, 1.5 Hz, 1H), 7.78 (d, *J* = 7.8 Hz, 1H), 7.61 (t, *J* = 7.9 Hz, 1H), 7.12 (d, *J* = 8.4 Hz, 2H), 6.97 (d, *J* = 8.4 Hz, 2H), 6.47 (s, 1H), 4.04 (d, *J* = 13.5 Hz, 2H), 3.95 (tt, *J* = 12.1, 3.8 Hz, 1H), 2.74 (td, *J* = 13.0, 2.4 Hz, 2H), 2.19 (s, 3H), 1.86 (dp, *J* = 11.8, 4.3, 3.7 Hz, 1H), 1.79 (dd, *J* = 12.4, 4.1 Hz, 2H), 1.52 (ddd, *J* = 11.8, 4.2, 2.0 Hz, 2H), 0.88–0.81 (m, 2H), 0.81–0.74 (m, 1H), 0.74–0.64 (m, 2H). **^13^C NMR (101 MHz, CDCl_3_) δ** 153.9, 139.9, 135.2, 131.7, 130.9, 130.6, 129.4, 128.9, 128.3, 128.3, 123.4, 123.3, 119.3, 57.7, 43.1, 29.8, 25.1, 19.7, 6.6. **HRMS (ESI):** Exact mass calculated for C_23_H_27_F_3_N_3_O_3_S [M + H]^+^: 482.1720, found: 482.1723.

***N*-(3,5-bis(trifluoromethyl)phenyl)-4-((*N*-cyclopropyl-3-(trifluoromethyl)phenyl)sulfonamido)piperidine-1-carboxamide** (**5aa′**): General procedure was followed using **1’** (95 mg, 0.37 mmol, 1.0 equiv.), **4aa** (100 mg, 0.28 mmol, 1 equiv.), PhI(OAc)_2_ (180 mg, 2 equiv.), K_3_PO_4_ (120 mg, 2 equiv.), 1,2-DCE (2 mL), at 80 °C for 18 h. Column chromatography was performed using 85/15 *v/v* petroleum ether/acetone as eluent and yielded **5aa’** (200 mg, 88%). Yellow solid. mp—158–160 °C. **FTIR (cm^−1^):** 3318, 2953, 1042, 1545, 1450, 1375, 1324, 1273, 1161, 1121, 1068, 944, 873, 805, 733, 698. **^1^H NMR (400 MHz, CDCl_3_) δ** 8.13 (s, 1H), 8.06 (d, J = 7.9 Hz, 1H), 7.88 (dd, J = 7.8, 0.9 Hz, 1H), 7.83 (s, 2H), 7.72 (t, J = 7.9 Hz, 1H), 7.67 (s, 1H), 7.42 (d, J = 1.6 Hz, 1H), 4.24 (dd, J = 11.1, 2.5 Hz, 2H), 4.10–4.00 (m, 1H), 2.92–2.81 (m, 2H), 1.97 (dq, J = 7.0, 3.5 Hz, 1H), 1.89 (dd, J = 12.5, 4.1 Hz, 2H), 1.69–1.60 (m, 2H), 0.90 (dt, J = 7.2, 3.2 Hz, 2H), 0.81–0.74 (m, 2H). **^13^C NMR (101 MHz, CDCl_3_) δ** 154.1, 140.9, 140.7, 132.0, 131.8, 131.6, 131.5, 130.4, 130.0, 129.5, 129.4, 124.5, 124.3, 124.2, 124.2, 121.8, 120.5, 119.5, 115.8, 112.0, 58.7, 44.0, 30.9, 30.8, 28.4, 26.2, 7.5. **^19^F NMR (376 MHz, CDCl_3_)** δ -62.9, -63.1. **HRMS (ESI):** Exact mass calculated for C_24_H_23_F_9_N_3_O_3_S [M + H]^+^: 604.1311, found: 604.1317.

***N*-(*p*-tolyl)azepane-1-carboxamide** (**5bb**): General procedure was followed using **1a** (50 mg, 0.37 mmol, 1.0 equiv.), **4bb** (73 mg, 0.74 mmol, 2 equiv.), PhI(OAc)_2_ (238 mg, 2 equiv.), K_3_PO_4_ (157 mg, 2 equiv.), 1,2-DCE (2 mL), at 80 °C for 18 h. Column chromatography was performed using 85/15 *v/v* petroleum ether/acetone as eluent and yielded **5bb** (12 mg, 14%). Brown solid. mp—123–125 °C. **FTIR (cm^−1^):** 3280, 2931, 2854, 1629, 1524, 1510, 1474, 1413, 1289, 1236, 1189, 1099, 902, 809, 749. **^1^H NMR (400 MHz, CDCl_3_) δ** 7.23–7.17 (m, 3H), 7.01 (d, *J* = 8.3 Hz, 2H), 6.15 (s, 1H), 3.46–3.40 (m, 4H), 2.22 (s, 3H), 1.71 (dq, *J* = 7.7, 2.5 Hz, 5H), 1.54 (dt, *J* = 7.3, 2.7 Hz, 5H). **^13^C NMR (101 MHz, CDCl_3_) δ** 155.2, 136.7, 132.2, 129.3, 119.9, 46.6, 28.6, 27.2, 20.7. **HRMS (ESI):** Exact mass calculated for C_14_H_20_N_2_O [M+Na]^+^: 255.1468, found: 255.1476.

***N*-(3,5-bis(trifluoromethyl)phenyl)azepane-1-carboxamide** (**5bb′**): General procedure was followed using **1’** (95 mg, 0.37 mmol, 1.0 equiv.), **4bb** (73 mg, 0.74 mmol, 2 equiv.), PhI(OAc)_2_ (238 mg, 2 equiv.), K_3_PO_4_ (157 mg, 2 equiv.), 1,2-DCE (2 mL), at 80 °C for 18 h. Column chromatography was performed using 85/15 *v/v* petroleum ether/acetone as eluent and yielded **5bb’** (33 mg, 33%). Yellow solid. mp—158–160 °C. **FTIR (cm^−1^):** 3320, 2931, 2854, 1642, 1539, 1439, 1368, 1269, 1167, 1121, 997, 967, 885, 701, 681. **^1^H NMR (400 MHz, CDCl_3_) δ** 8.24 (s, 2H), 7.81 (s, 1H), 7.13 (s, 1H), 3.90–3.79 (m, 4H), 2.20–2.07 (m, 4H), 2.01–1.90 (m, 4H). **^13^C NMR (101 MHz, CDCl_3_) δ** 154.4, 140.9, 132.4, 132.1, 131.8, 131.4, 127.3, 124.6, 121.9, 119.3, 119.3, 115.8, 115.8, 115.8, 46.7, 28.4, 27.1. **HRMS (ESI):** Exact mass calculated for C_15_H_17_F_6_N_2_O [M + H]^+^: 355.1240, found: 355.1249.

***N*-(*p*-tolyl)-1,4-oxazepane-4-carboxamide** (**5cc**): General procedure was followed using **1a** (50 mg, 0.37 mmol, 1.0 equiv.), **4cc** (74 mg, 0.74 mmol, 2 equiv.), PhI(OAc)_2_ (238 mg, 2 equiv.), K_3_PO_4_ (157 mg, 2 equiv.), 1,2-DCE (2 mL), at 80 °C for 18 h. Column chromatography was performed using 85/15 *v/v* petroleum ether/acetone as eluent and yielded **5cc** (22 mg, 26%). Yellow Solid. mp—112–114 °C. **FTIR (cm^−1^):** 3327, 2948, 2926, 2853, 1632, 1590, 1512, 1408, 1290, 1120, 1051, 927, 803, 750. **^1^H NMR (400 MHz, CDCl_3_) δ** 7.30–7.19 (m, 2H), 7.15–7.02 (m, 2H), 6.32 (s, 1H), 3.84–3.72 (m, 4H), 3.70–3.59 (m, 4H), 2.29 (s, 3H), 1.99 (tt, *J* = 8.1, 5.3 Hz, 2H). **^13^C NMR (101 MHz, CDCl_3_) δ** 155.2, 136.3, 132.7, 129.3, 120.3, 71.1, 70.3, 49.2, 45.2, 30.1, 20.7. **HRMS (ESI):** Exact mass calculated for C_13_H_19_N_2_O_2_ [M + H]^+^: 235.1442, found: 235.1443.

***N*-(3,5-bis(trifluoromethyl)phenyl)-1,4-oxazepane-4-carboxamide** (**5cc′**): General procedure was followed using **1’** (95 mg, 0.37 mmol, 1.0 equiv.), **4cc** (74 mg, 0.74 mmol, 2 equiv.), PhI(OAc)_2_ (238 mg, 2 equiv.), K_3_PO_4_ (157 mg, 2 equiv.), 1,2-DCE (2 mL), at 80 °C for 18 h. Column chromatography was performed using 85/15 *v/v* petroleum ether/acetone as eluent and yielded **5cc’** (115 mg, 88%). Colorless Solid. mp—109–111 °C. **FTIR (cm^−1^):** 3258, 2958, 2858, 1638, 1528, 1490, 1446, 1364, 1273, 1114, 1035, 990, 878, 750, 680. **^1^H NMR (400 MHz, CDCl_3_) δ** 7.78 (d, J = 1.6 Hz, 2H), 7.43 (s, 1H), 7.29 (s, 1H), 3.78 (q, J = 5.8, 5.1 Hz, 4H), 3.72–3.64 (m, 4H), 2.02–1.93 (m, 2H). **^13^C NMR (101 MHz, CDCl_3_) δ** 154.7, 140.6, 132.2, 131.9, 131.6, 131.2, 127.2, 124.5, 121.8, 119.9, 119.8, 119.0, 116.2, 116.1, 116.0, 70.5, 70.2, 49.2, 45.3, 30.0. **HRMS (ESI):** Exact mass calculated for C_14_H_15_F_6_N_2_O_2_ [M + H]^+^: 357.1033, found: 357.1039.

**1-benzyl-1-methyl-3-(*p*-tolyl)urea** (**5dd**): General procedure was followed using **1a** (50 mg, 0.37 mmol, 1.0 equiv.), **4dd** (90 mg, 1.15 mmol, 5 equiv.), PhI(OAc)_2_ (238 mg, 2 equiv.), K_3_PO_4_ (157 mg, 2 equiv.), 1,2-DCE (2 mL), at 80 °C for 18 h. Column chromatography was performed using 85/15 *v/v* petroleum ether/acetone as eluent and yielded **5dd** (17 mg, 18%). Yellow solid. mp—113–115 °C. **FTIR (cm^−1^):** 3311, 3064, 3026, 2916, 2865, 1629, 1594, 1510, 1373, 1245, 1021, 807, 741. **^1^H NMR (400 MHz, CDCl_3_) δ** 7.31–7.26 (m, 2H), 7.22 (d, *J* = 6.9 Hz, 3H), 7.15 (d, *J* = 8.4 Hz, 2H), 7.00 (d, *J* = 8.3 Hz, 2H), 6.22 (s, 1H), 4.50 (s, 2H), 2.94 (s, 3H), 2.21 (s, 3H). **^13^C NMR (101 MHz, CDCl_3_) δ** 154.8, 136.5, 135.4, 131.5, 128.3, 127.8, 126.5, 126.2, 119.0, 51.3, 33.7, 19.7. **HRMS (ESI):** Exact mass calculated for C_16_H_19_N_2_O [M + H]^+^: 255.1492, found: 255.1494.

**1-benzyl-3-(3,5-bis(trifluoromethyl)phenyl)-1-methylurea** (**5dd′**): General procedure was followed using **1′** (95 mg, 0.37 mmol, 1.0 equiv.), **4dd** (90 mg, 1.15 mmol, 5 equiv.), PhI(OAc)_2_ (238 mg, 2 equiv.), K_3_PO_4_ (157 mg, 2 equiv.), 1,2-DCE (2 mL), at 80 °C for 18 h. Column chromatography was performed using 85/15 *v/v* petroleum ether/acetone as eluent and yielded **5dd’** (61 mg, 44%). Yellow liquid. mp—113–115 °C. **FTIR (cm^−1^):** 2922, 2854, 1715, 1651, 1556, 1468, 1368, 1276, 1172, 1127, 1019, 878, 749, 697. **^1^H NMR (400 MHz, CDCl_3_) δ** 7.85 (d, J = 1.6 Hz, 2H), 7.49 (s, 1H), 7.38 (t, J = 7.2 Hz, 2H), 7.35–7.31 (m, 1H), 7.31–7.26 (m, 2H), 6.75 (s, 1H), 4.60 (s, 2H), 3.06 (s, 3H). **^13^C NMR (101 MHz, CDCl_3_) δ** 154.9, 140.6, 136.7, 132.5, 132.2, 131.8, 131.5, 129.0, 127.9, 127.2, 124.5, 121.8, 119.3, 116.2, 116.1, 116.0, 52.4, 34.8. **HRMS (ESI):** Exact mass calculated for C_17_H_15_F_6_N_2_O [M + H]^+^: 377.1084, found: 377.1080.

***N*-(*p*-tolyl)thiomorpholine-4-carboxamide** (**7a**): General procedure was followed using **1d** (50 mg, 0.37 mmol, 1.0 equiv.), **6a** (76 mg, 0.74 mmol, 2 equiv.), PhI(OAc)_2_ (238 mg, 2 equiv.), K_3_PO_4_ (157 mg, 2 equiv.), 1,2-DCE (2 mL), at 80 °C for 18 h. Column chromatography was performed using 85/15 *v/v* petroleum ether/acetone as eluent and yielded **7d** (69 mg, 79%). Orange solid. mp—179–181 °C. **FTIR (cm^−1^):** 3320, 2958, 2905, 1738, 1629, 1594, 1508, 1413, 1306, 1240, 1015, 942, 805, 743. **^1^H NMR (400 MHz, CDCl_3_) δ** 7.22 (d, *J* = 8.4 Hz, 2H), 7.10 (d, *J* = 8.3 Hz, 2H), 6.45 (s, 1H), 3.81–3.75 (m, 4H), 2.68–2.62 (m, 4H), 2.32 (s, 3H). **^13^C NMR (101 MHz, CDCl_3_) δ** 154.9, 136.2, 132.9, 129.4, 120.5, 47.0, 27.0, 20.7. **HRMS (ESI):** Exact mass calculated for C_12_H_16_N_2_OS [M+Na]^+^: 259.0876, found: 259.0886.

***N*-(*p*-tolyl)morpholine-4-carboxamide (7a’):** General procedure was followed using **1d** (50 mg, 0.37 mmol, 1.0 equiv.), **6b** (64 mg, 0.74 mmol, 2 equiv.), PhI(OAc)_2_ (238 mg, 2 equiv.), K_3_PO_4_ (157 mg, 2 equiv.), 1,2-DCE (2 mL), at 80 °C for 18 h. Column chromatography was performed using 85/15 *v/v* petroleum ether/acetone as eluent and yielded **7d′** (60 mg, 73%). Brown solid. mp—161–162 °C. FTIR (cm^−1^): 3320, 3035, 2958, 2909, 1631, 1594, 1510, 1472, 1417, 1309, 1289, 1236, 1207, 1017, 946, 807, 747. **^1^H NMR (400 MHz, CDCl_3_)** δ 7.27–7.22 (m, 2H), 6.88–6.83 (m, 2H), 6.41 (s, 1H), 3.80 (s, 4H), 3.75–3.69 (m, 4H), 3.48–3.43 (m, 4H). **^13^C NMR (101 MHz, CDCl_3_)** δ 156.0, 155.7, 131.6, 122.6, 114.1, 66.5, 55.5, 44.2. **HRMS (ESI):** Exact mass calculated for C_12_H_17_N_2_O_2_ [M + H]^+^: 221.1285, found: 221.1289.

***N*-(*o*-tolyl)thiomorpholine-4-carboxamide** (**7b**): General procedure was followed using **1b** (50 mg, 0.37 mmol, 1.0 equiv.), **6a** (76 mg, 0.74 mmol, 2 equiv.), PhI(OAc)_2_ (238 mg, 2 equiv.), K_3_PO_4_ (157 mg, 2 equiv.), 1,2-DCE (2 mL), at 80 °C for 18 h. Column chromatography was performed using 85/15 *v/v* petroleum ether/acetone as eluent and yielded **7b** (60 mg, 75%). Orange solid. mp—190–191 °C. **FTIR (cm^−1^):** 3307, 2949, 2905, 1627, 1583, 1505, 1452, 1395, 1302, 1245, 1012, 942, 743. **^1^H NMR (400 MHz, CDCl_3_) δ** 7.51 (d, *J* = 8.1 Hz, 1H), 7.19 (t, *J* = 7.3 Hz, 2H), 7.06 (td, *J* = 7.4, 1.3 Hz, 1H), 6.22 (s, 1H), 3.82–3.73 (m, 4H), 2.70–2.61 (m, 4H), 2.25 (s, 3H). **^13^C NMR (101 MHz, CDCl_3_) δ** 155.0, 136.8, 130.4, 129.7, 126.7, 124.5, 123.5, 47.1, 27.0, 17.8. **HRMS (ESI):** Exact mass calculated for C_12_H_17_N_2_OS [M + H]^+^: 237.1057, found: 237.1059.

***N*-(*o*-tolyl)morpholine-4-carboxamide (7b’):** General procedure was followed using **1b** (50 mg, 0.37 mmol, 1.0 equiv.), **6a** (64 mg, 0.74 mmol, 2 equiv.), PhI(OAc)_2_ (238 mg, 2 equiv.), K_3_PO_4_ (157 mg, 2 equiv.), 1,2-DCE (2 mL), at 80 °C for 18 h. Column chromatography was performed using 85/15 *v/v* petroleum ether/acetone as eluent and yielded **7b’** (44 mg, 54%). Pink solid. mp—145–147 °C. **FTIR (cm^−1^):** 3287, 2958, 2916, 2847, 1629, 1578, 1508, 1455, 1375, 1253, 1112, 1039, 990, 858, 741. **^1^H NMR (400 MHz, CDCl_3_)** δ7.59 (dd, *J* = 8.0, 1.3 Hz, 1H), 7.23–7.17 (m, 2H), 7.06 (td, *J* = 7.5, 1.3 Hz, 1H), 6.22 (s, 1H), 3.78–3.70 (m, 5H), 3.51–3.44 (m, 5H), 2.26 (s, 3H). **^13^C NMR (101 MHz, CDCl_3_)** δ 155.5, 136.7, 130.4, 129.3, 126.7, 124.4, 123.1, 66.5, 44.3, 17.8. **HRMS (ESI):** Exact mass calculated for C_12_H_16_N_2_O_2_ [M + H]^+^: 221.1285, found: 221.1287.

***N*-(*m*-tolyl)thiomorpholine-4-carboxamide** (**7c**): General procedure was followed using **1c** (50 mg, 0.37 mmol, 1.0 equiv.), **6a** (76 mg, 0.74 mmol, 2 equiv.), PhI(OAc)_2_ (238 mg, 2 equiv.), K_3_PO_4_ (157 mg, 2 equiv.), 1,2-DCE (2 mL), at 80 °C for 18 h. Column chromatography was performed using 85/15 *v/v* petroleum ether/acetone as eluent and yielded **7c** (58 mg, 67%). Yellow solid. mp—116–117 °C. **FTIR (cm^−1^):** 3300, 2960, 2909, 1631, 1583, 1530, 1421, 1291, 1209, 1039, 940, 776. **^1^H NMR (400 MHz, CDCl_3_) δ** 7.24–7.12 (m, 3H), 7.10 (dt, *J* = 8.2, 1.5 Hz, 1H), 6.87 (d, *J* = 7.4 Hz, 1H), 6.62 (s, 1H), 3.82–3.73 (m, 4H), 2.67–2.58 (m, 4H), 2.32 (s, 4H). **^13^C NMR (101 MHz, CDCl_3_) δ** 154.8, 138.8, 138.7, 128.7, 128.6, 124.2, 121.1, 117.4, 47.0, 27.0, 21.4. **HRMS (ESI):** Exact mass calculated for C_12_H_17_N_2_OS [M + H]^+^: 237.1057, found: 237.1058.

***N*-(*m*-tolyl)morpholine-4-carboxamide (7c’):** General procedure was followed using **1c** (50 mg, 0.37 mmol, 1.0 equiv.), **6b** (64 mg, 0.74 mmol, 2 equiv.), PhI(OAc)_2_ (238 mg, 2 equiv.), K_3_PO_4_ (157 mg, 2 equiv.), 1,2-DCE (2 mL), at 80 °C for 18 h. Column chromatography was performed using 85/15 *v/v* petroleum ether/acetone as eluent and yielded **7c’** (37 mg, 46%). Yellow solid. mp—126–127 °C. **FTIR (cm^−1^):** 3285, 2964, 2909, 2843, 1631, 1605, 1523, 1483, 1452, 1421, 1298, 1245, 1108, 1015, 982, 864, 776. **^1^H NMR (400 MHz, CDCl_3_)** δ 7.24 (d, *J* = 2.0 Hz, 1H), 7.19 (t, *J* = 7.7 Hz, 1H), 7.12 (dt, *J* = 8.2, 1.6 Hz, 1H), 6.89 (d, *J* = 7.4 Hz, 1H), 6.46 (s, 1H), 3.77–3.69 (m, 4H), 3.52–3.42 (m, 4H), 2.34 (s, 3H). **^13^C NMR (101 MHz, CDCl_3_)** δ 155.2, 138.8, 138.6, 128.7, 124.2, 120.9, 117.2, 66.5, 44.2, 21.5. **HRMS (ESI):** Exact mass calculated for C_12_H_17_N_2_O_2_ [M + H]^+^: 221.1285, found: 221.1287.

***N*-phenylthiomorpholine-4-carboxamide** (**7d**): General procedure was followed using **1a** (45 mg, 0.37 mmol, 1.0 equiv.), **6a** (76 mg, 0.74 mmol, 2 equiv.), PhI(OAc)_2_ (238 mg, 2 equiv.), K_3_PO_4_ (157 mg, 2 equiv.), 1,2-DCE (2 mL), at 80 °C for 18 h. Column chromatography was performed using 85/15 *v/v* petroleum ether/acetone as eluent and yielded **7a** (56 mg, 74%). Brown solid. mp—167–168 °C. **FTIR (cm^−1^):** 3298, 2953, 2902, 1627, 1589, 1521, 1441, 1399, 1309, 1216, 1015, 942, 745. **^1^H NMR (400 MHz, CDCl_3_) δ** 7.38–7.24 (m, 4H), 7.10–7.02 (m, 1H), 6.69 (s, 1H), 3.81–3.72 (m, 4H), 2.66–2.58 (m, 4H). **^13^C NMR (101 MHz, CDCl_3_) δ** 154.8, 138.9, 128.8, 123.3, 120.4, 46.9, 27.0. **HRMS (ESI):** Exact mass calculated for C_11_H_15_N_2_OS [M + H]^+^: 233.0900, found: 233.0901.

***N*-phenylmorpholine-4-carboxamide (7d’):** General procedure was followed using **1a** (45 mg, 0.37 mmol, 1.0 equiv.), **6b** (64 mg, 0.74 mmol, 2 equiv.), PhI(OAc)_2_ (238 mg, 2 equiv.), K_3_PO_4_ (157 mg, 2 equiv.), 1,2-DCE (2 mL), at 80 °C for 18 h. Column chromatography was performed using 85/15 *v/v* petroleum ether/acetone as eluent and yielded **7a’** (54 mg, 66%). Colorless solid. mp—156–158 °C. **FTIR (cm^−1^):** 3250, 3124, 3046, 2953, 2854, 1627, 1592, 1523, 1497, 1439, 1402, 1298, 1242, 1108, 1074, 988, 853, 738. **^1^H NMR (400 MHz, CDCl_3_)** δ 7.39–7.33 (m, 2H), 7.33–7.25 (m, 2H), 7.11–7.03 (m, 1H), 6.63 (s, 1H), 3.74–3.67 (m, 4H), 3.49–3.43 (m, 4H). **^13^C NMR (101 MHz, CDCl_3_)** δ 155.3, 138.8, 128.9, 123.3, 120.3, 66.5, 44.2. **HRMS (ESI):** Exact mass calculated for C_11_H_15_N_2_O_2_ [M + H]^+^: 207.1129, found: 207.1130.

***N*-(3,4-dimethylphenyl)thiomorpholine-4-carboxamide** (**7e**): General procedure was followed using **1e** (56 mg, 0.37 mmol, 1.0 equiv.), **6a** (76 mg, 0.74 mmol, 2 equiv.), PhI(OAc)_2_ (238 mg, 2 equiv.), K_3_PO_4_ (157 mg, 2 equiv.), 1,2-DCE (2 mL), at 80 °C for 18 h. Column chromatography was performed using 85/15 *v/v* petroleum ether/acetone as eluent and yielded **7e** (77 mg, 78%). Yellow solid. mp—173–174 °C. **FTIR (cm-1):** 3316, 2909, 1771, 1629, 1587, 1519, 1417, 1247, 1211, 1019, 942, 814, 745. **^1^H NMR (400 MHz, CDCl_3_) δ** 7.06 (d, *J* = 1.8 Hz, 1H), 6.95 (d, *J* = 1.7 Hz, 2H), 6.31 (s, 1H), 3.72–3.64 (m, 4H), 2.60–2.51 (m, 4H), 2.13 (d, *J* = 6.8 Hz, 6H). **^13^C NMR (101 MHz, CDCl_3_) δ** 154.9, 137.1, 136.4, 131.6, 129.8, 121.9, 117.9, 47.0, 27.0, 19.9, 19.0. **HRMS (ESI):** Exact mass calculated for C_13_H_19_N_2_OS [M + H]^+^: 251.1213, found: 251.1214.

***N*-(3,4-dimethylphenyl)morpholine-4-carboxamide (7e’):** General procedure was followed using **1e** (56 mg, 0.37 mmol, 1.0 equiv.), **6b** (64 mg, 0.74 mmol, 2 equiv.), PhI(OAc)_2_ (238 mg, 2 equiv.), K_3_PO_4_ (157 mg, 2 equiv.), 1,2-DCE (2 mL), at 80 °C for 18 h. Column chromatography was performed using 85/15 *v/v* petroleum ether/acetone as eluent and yielded **7e’** (62 mg, 72%). Yellow solid. mp—164–165 °C. FTIR (cm-1): 3289, 2964, 2914, 2891, 2852, 1771, 1634, 1594, 1505, 1406, 1242, 1108, 1066, 988, 802. **^1^H NMR (400 MHz, CDCl_3_)** δ 7.27–7.22 (m, 2H), 6.87–6.82 (m, 2H), 6.46 (s, 1H), 3.79 (s, 4H), 3.74–3.69 (m, 4H), 3.48–3.43 (m, 4H). **^13^C NMR (101 MHz, CDCl_3_)** δ 156.0, 155.7, 131.7, 122.6, 114.1, 66.5, 55.5, 44.2. **HRMS (ESI):** Exact mass calculated for C_13_H_19_N_2_O_2_ [M + H]^+^: 235.1442, found: 235.1443.

***N*-(3-methoxyphenyl)thiomorpholine-4-carboxamide** (**7f**): General procedure was followed using **1f** (56 mg, 0.37 mmol, 1.0 equiv.), **6a** (76 mg, 0.74 mmol, 2 equiv.), PhI(OAc)_2_ (238 mg, 2 equiv.), K_3_PO_4_ (157 mg, 2 equiv.), 1,2-DCE (2 mL), at 80 °C for 18 h. Column chromatography was performed using 85/15 *v/v* petroleum ether/acetone as eluent and yielded **7f** (68 mg, 73%). Brown solid. mp—110–112 °C. **FTIR (cm^−1^):** 3322, 3075, 3002, 2964, 2898, 1638, 1528, 1225, 1154, 1041, 946, 749. **^1^H NMR (400 MHz, CDCl_3_) δ** 7.07 (t, *J* = 8.1 Hz, 1H), 6.98 (t, *J* = 2.3 Hz, 1H), 6.78–6.73 (m, 2H), 6.53–6.48 (m, 1H), 3.68 (s, 3H), 3.67–3.62 (m, 4H), 2.54–2.48 (m, 4H). **^13^C NMR (101 MHz, CDCl_3_) δ** 159.0, 153.7, 139.2, 128.4, 111.5, 107.9, 105.1, 54.2, 45.9, 26.0. **HRMS (ESI):** Exact mass calculated for C_12_H_17_N_2_O_2_S [M + H]^+^: 253.1006, found: 253.1007.

***N*-(4-methoxyphenyl)thiomorpholine-4-carboxamide** (**7g**): General procedure was followed using **1g** (56 mg, 0.37 mmol, 1.0 equiv.), **6a** (76 mg, 0.74 mmol, 2 equiv.), PhI(OAc)_2_ (238 mg, 2 equiv.), K_3_PO_4_ (157 mg, 2 equiv.), 1,2-DCE (2 mL), at 80 °C for 18 h. Column chromatography was performed using 85/15 *v/v* petroleum ether/acetone as eluent and yielded **7g** (60 mg, 66%). Brown solid. mp—133–134 °C. FTIR (cm-1): 3292, 2958, 2909, 2888, 1605, 1508, 1410, 1384, 1218, 1194, 1032, 946, 820, 749. **^1^H NMR (400 MHz, CDCl_3_) δ** 7.21 (d, *J* = 9.0 Hz, 2H), 6.81 (d, *J* = 9.0 Hz, 2H), 6.68 (s, 1H), 3.77 (s, 3H), 3.75–3.71 (m, 4H), 2.62–2.57 (m, 4H). **^13^C NMR (101 MHz, CDCl_3_) δ** 161.2, 156.0, 155.3, 131.9, 122.8, 114.0, 55.5, 46.9, 27.0. **HRMS (ESI):** Exact mass calculated for C_12_H_17_N_2_O_2_S [M + H]^+^: 253.1006, found: 253.1008.

***N*-(4-methoxyphenyl)morpholine-4-carboxamide (7g’):** General procedure was followed using **1g** (56 mg, 0.37 mmol, 1.0 equiv.), **6b** (64 mg, 0.74 mmol, 2 equiv.), PhI(OAc)_2_ (238 mg, 2 equiv.), K_3_PO_4_ (157 mg, 2 equiv.), 1,2-DCE (2 mL), at 80 °C for 18 h. Column chromatography was performed using 85/15 *v/v* petroleum ether/acetone as eluent and yielded **7g’** (57 mg, 65%). Pink solid. mp—117–119 °C. FTIR (cm^−1^): 3261, 2960, 2902, 2836, 1627, 1594, 1510, 1417, 1390, 1291, 1222, 1103, 1032, 984, 741. **^1^H NMR (400 MHz, CDCl_3_)** δ 7.27–7.22 (m, 2H), 6.88–6.83 (m, 2H), 6.41 (s, 1H), 3.80 (s, 4H), 3.75–3.69 (m, 4H), 3.48–3.43 (m, 4H). **^13^C NMR (101 MHz, CDCl_3_)** δ 156.0, 155.7, 131.6, 122.6, 114.1, 66.5, 55.5, 44.2. **HRMS (ESI):** Exact mass calculated for C_12_H_17_N_2_O_3_ [M + H]^+^: 237.1234, found: 237.1237.

***N*-(2-fluorophenyl)thiomorpholine-4-carboxamide** (**7h**): General procedure was followed using **1h** (52 mg, 0.37 mmol, 1.0 equiv.), **6a** (76 mg, 0.74 mmol, 2 equiv.), PhI(OAc)_2_ (238 mg, 2 equiv.), K_3_PO_4_ (157 mg, 2 equiv.), 1,2-DCE (2 mL), at 80 °C for 18 h. Column chromatography was performed using 85/15 *v/v* petroleum ether/acetone as eluent and yielded **7h** (52 mg, 63%). Brown solid. mp—118–119 °C. **FTIR (cm^−1^):** 3311, 2958, 2905, 1631, 1585, 1494, 1455, 1395, 1251, 1189, 1099, 946, 745. **^1^H NMR (400 MHz, CDCl_3_)** δ 8.01 (td, *J* = 8.2, 1.7 Hz, 1H), 7.14–7.03 (m, 2H), 6.99 (dddd, *J* = 8.5, 7.1, 5.3, 1.7 Hz, 1H), 6.62 (s, 1H), 3.85–3.78 (m, 4H), 2.72–2.65 (m, 4H). **^13^C NMR (101 MHz, CDCl_3_)** δ 153.9, 151.5, 127.3 (*J* = 11.2 Hz), 124.5, 123.2 (*J* = 10 Hz), 121.8, 114.7 (*J* = 23.7 Hz), 47.0, 27.0. **^19^F NMR (376 MHz, CDCl_3_)** δ −132.1, −132.1, −132.1, −132.1, −132.2, −132.2, −132.2, −132.2. **HRMS (ESI):** Exact mass calculated for C_11_H_14_FN_2_OS [M + H]^+^: 241.0806, found: 241.0808.

***N*-(2-fluorophenyl)morpholine-4-carboxamide (7h’):** General procedure was followed using **1h** (52 mg, 0.37 mmol, 1.0 equiv.), **6b** (64 mg, 0.74 mmol, 2 equiv.), PhI(OAc)_2_ (238 mg, 2 equiv.), K_3_PO_4_ (157 mg, 2 equiv.), 1,2-DCE (2 mL), at 80 °C for 18 h. Column chromatography was performed using 85/15 *v/v* petroleum ether/acetone as eluent and yielded **7h’** (44 mg, 55%). Yellow solid. mp—93–95 °C. **FTIR (cm^−1^):** 3367, 3053, 2980, 2858, 1645, 1600, 1519, 1490, 1439, 1384, 1262, 1105, 1063, 990, 745. **^1^H NMR (400 MHz, CDCl_3_)** δ 8.06 (td, *J* = 8.2, 1.7 Hz, 1H), 7.19–6.93 (m, 3H), 6.72–6.59 (m, 1H), 3.84–3.68 (m, 4H), 3.58–3.44 (m, 4H). **^13^C NMR (101 MHz, CDCl_3_)** δ 154.4, 153.9, 151.5, 134.0, 133.9, 132.3, 132.2, 127.2 (*J* = 11.2 Hz), 124.5, 123.2 (*J* = 10 Hz), 121.7, 114.7 (*J* = 23.7 Hz), 66.4, 44.2. **^19^F NMR (376 MHz, CDCl_3_)** δ −132.39. **HRMS (ESI):** Exact mass calculated for C_11_H_14_FN_2_O_2_ [M + H]^+^: 225.1034, found: 225.1033.

***N*-(3-fluorophenyl)thiomorpholine-4-carboxamide** (**7i**): General procedure was followed using **1i** (52 mg, 0.37 mmol, 1.0 equiv.), **6a** (76 mg, 0.74 mmol, 2 equiv.), PhI(OAc)_2_ (238 mg, 2 equiv.), K_3_PO_4_ (157 mg, 2 equiv.), 1,2-DCE (2 mL), at 80 °C for 18 h. Column chromatography using 85/15 *v*/*v* petroleum ether/acetone yielded **7i** (63 mg, 67%). Red solid. mp—128–130 °C. **FTIR (cm^−1^):** 3209, 3129, 3059, 2913, 1727, 1597, 1531, 1440, 1297, 1139, 1035, 955, 834. **^1^H NMR (400 MHz, CDCl_3_) δ** 7.20–7.07 (m, 2H), 6.95–6.90 (m, 1H), 6.78 (s, 1H), 6.64 (tdd, *J* = 8.3, 2.6, 0.9 Hz, 1H), 3.72–3.62 (m, 4H), 2.58–2.50 (m, 4H). **^13^C NMR (101 MHz, CDCl_3_) δ** 164.2, 161.8, 154.4, 140.7 (*J* = 13.7 Hz), 129.9 (*J* = 12.5 Hz), 115.4 (*J* = 3.75 Hz), 109.9 (*J* = 26.2 Hz), 107.6 (*J* = 31.2 Hz), 46.9, 27.0. **^19^F NMR (376 MHz, CDCl_3_) δ** −111.9, −112.0, −112.0, −112.0, −112.0, −112.0, −112.0, −112.0. **HRMS (ESI):** Exact mass calculated for C_11_H_14_FN_2_OS [M + H]^+^: 241.0806, found: 248.0807.

***N*-(4-fluorophenyl)thiomorpholine-4-carboxamide** (**7j**): General procedure was followed using **1j** (52 mg, 0.37 mmol, 1.0 equiv.), **6a** (76 mg, 0.74 mmol, 2 equiv.), PhI(OAc)_2_ (238 mg, 2 equiv.), K_3_PO_4_ (157 mg, 2 equiv.), 1,2-DCE (2 mL), at 80 °C for 18 h. Column chromatography was performed using 85/15 *v/v* petroleum ether/acetone as eluent and yielded **7j** (63 mg, 77%). Brown solid. mp—167–169 °C. **FTIR (cm^−1^):** 3201, 3126, 3042, 2900, 1616, 1532, 1505, 1417, 1302, 1205, 1024, 942, 811, 743. **^1^H NMR (400 MHz, CDCl_3_) δ** 7.28 (dd, *J* = 9.1, 4.7 Hz, 2H), 6.99 (t, *J* = 8.6 Hz, 2H), 6.50 (s, 1H), 3.82–3.74 (m, 4H), 2.70–2.62 (m, 4H). **^13^C NMR (101 MHz, CDCl_3_) δ** 160.2, 157.8, 154.8, 134.7 (*J* = 3.7 Hz), 122.3 (*J* = 8.7 Hz), 115.6 (*J* = 28.7 Hz), 46.9, 27.0. **^19^F NMR (376 MHz, CDCl_3_) δ** −119.5, −119.5, −119.5, −119.6, −119.6. **HRMS (ESI):** Exact mass calculated for C_11_H_14_FN_2_OS [M + H]^+^: 241.0806, found: 241.0807.

***N*-(4-fluorophenyl)morpholine-4-carboxamide (7j’):** General procedure was followed using **1j** (52 mg, 0.37 mmol, 1.0 equiv.), **6b** (64 mg, 0.74 mmol, 2 equiv.), PhI(OAc)_2_ (238 mg, 2 equiv.), K_3_PO_4_ (157 mg, 2 equiv.), 1,2-DCE (2 mL), at 80 °C for 18 h. Column chromatography was performed using 85/15 *v/v* petroleum ether/acetone as eluent and yielded **7j’** (44 mg, 54%). Brown solid. mp—177–179 °C. **FTIR (cm^−1^):** 3254, 3141,3053, 2980, 2920, 2854, 1631, 1609, 1528, 1508, 1419, 1302, 1245, 1205, 1110, 1068, 990, 822, 741. **^1^H NMR (400 MHz, CDCl_3_)** δ 7.35–7.27 (m, 3H), 7.01 (dd, *J* = 9.0, 8.3 Hz, 2H), 6.39 (s, 1H), 3.78–3.73 (m, 4H), 3.51–3.46 (m, 4H). **^13^C NMR (101 MHz, CDCl_3_)** δ 160.2, 157.8, 155.2, 134.6 (*J* = 3.7 Hz), 134.5, 122.2 (*J* = 10 Hz), 122.1, 115.6 (*J* = 27 Hz), 115.4, 66.4, 44.2. **^19^F NMR (376 MHz, CDCl_3_)** δ −119.5, −119.5. **HRMS (ESI):** Exact mass calculated for C_11_H_14_FN_2_O_2_ [M + H]^+^: 225.1034, found: 225.1036.

***N*-(2-chlorophenyl)thiomorpholine-4-carboxamide** (**7k**): General procedure was followed using **1k** (58 mg, 0.37 mmol, 1.0 equiv.), **6a** (76 mg, 0.74 mmol, 2 equiv.), PhI(OAc)_2_ (238 mg, 2 equiv.), K_3_PO_4_ (157 mg, 2 equiv.), 1,2-DCE (2 mL), at 80 °C for 18 h. Column chromatography was performed using 85/15 *v/v* petroleum ether/acetone as eluent and yielded **7k** (79 mg, 84%). Brown solid. mp—115–116 °C. **FTIR (cm^−1^):** 3338, 2905, 1629, 1576, 1512, 1439, 1395, 1295, 1054, 944, 745. **^1^H NMR (400 MHz, CDCl_3_) δ** 8.14 (dd, *J* = 8.3, 1.6 Hz, 1H), 7.35 (dd, *J* = 8.0, 1.5 Hz, 1H), 7.30–7.22 (m, 1H), 6.98 (td, *J* = 7.6, 1.6 Hz, 2H), 3.88–3.79 (m, 4H), 2.75–2.66 (m, 4H). **^13^C NMR (101 MHz, CDCl_3_) δ** 153.6, 135.6, 128.8, 127.7, 123.4, 122.5, 121.2, 47.1, 27.0. **HRMS (ESI):** Exact mass calculated for C_11_H_14_ClN_2_OS [M + H]^+^: 257.0510, found: 257.0512.

***N*-(2-chlorophenyl)morpholine-4-carboxamide (7k’):** General procedure was followed using **1k** (58 mg, 0.37 mmol, 1.0 equiv.), **6b** (64 mg, 0.74 mmol, 2 equiv.), PhI(OAc)_2_ (238 mg, 2 equiv.), K_3_PO_4_ (157 mg, 2 equiv.), 1,2-DCE (2 mL), at 80 °C for 18 h. Column chromatography was performed using 85/15 *v/v* petroleum ether/acetone as eluent and yielded **7k’** (50 mg, 65%). Colorless solid. mp—128–130 °C. FTIR (cm^−1^): 3212, 2909, 2887, 2854, 1629, 1572, 1510, 1439, 1368, 1245, 1114, 1050, 1019, 993, 898, 747. **^1^H NMR (400 MHz, CDCl_3_)** δ 8.21 (dd, *J* = 8.3, 1.6 Hz, 1H), 7.35 (dd, *J* = 8.0, 1.5 Hz, 1H), 7.27 (ddd, *J* = 8.6, 7.4, 1.6 Hz, 1H), 7.06–7.00 (m, 1H), 6.98 (dd, *J* = 7.7, 1.6 Hz, 1H), 3.84–3.73 (m, 4H), 3.59–3.48 (m, 4H). **^13^C NMR (101 MHz, CDCl_3_)** δ 154.2, 135.5, 128.7, 127.7, 123.3, 122.3, 120.9, 66.4, 44.1. **HRMS (ESI):** Exact mass calculated for C_11_H_14_ClN_2_O_2_ [M + H]^+^: 241.0739, found: 241.0741.

***N*-(4-chlorophenyl)thiomorpholine-4-carboxamide** (**7l**): General procedure was followed using **1l** (58 mg, 0.37 mmol, 1.0 equiv.), **6a** (76 mg, 0.74 mmol, 2 equiv.), PhI(OAc)_2_ (238 mg, 2 equiv.), K_3_PO_4_ (157 mg, 2 equiv.), 1,2-DCE (2 mL), at 80 °C for 18 h. Column chromatography was performed using 85/15 *v/v* petroleum ether/acetone as eluent and yielded **7l** (37 mg, 38%). Brown solid. mp—208–210 °C. **FTIR (cm^−1^):** 3298, 2958, 2894, 1631, 1512, 1587, 1490, 1413, 1298, 1240, 1220, 1194, 1085, 1008, 937, 816, 742. **^1^H NMR (400 MHz, CDCl_3_) δ** 7.28 (d, J = 9.0 Hz, 2H), 7.24 (d, J = 9.0 Hz, 2H), 6.37 (s, 1H), 3.80–3.77 (m, 4H), 2.68–2.65 (m, 4H). **^13^C NMR (101 MHz, CDCl_3_)** δ 154.33, 137.4, 128.9, 128.3, 121.3, 47.0, 27.1. **HRMS (ESI):** Exact mass calculated for C_11_H_13_ClN_2_OS [M+Na]^+^: 279.0330, found: 279.0328.

***N*-(4-chlorophenyl)morpholine-4-carboxamide (7l′):** General procedure was followed using **1l** (56 mg, 0.37 mmol, 1.0 equiv.), **6b** (64 mg, 0.74 mmol, 2 equiv.), PhI(OAc)_2_ (238 mg, 2 equiv.), K_3_PO_4_ (157 mg, 2 equiv.), 1,2-DCE (2 mL), at 80 °C for 18 h. Column chromatography was performed using 85/15 *v/v* petroleum ether/acetone as eluent and yielded **7l’** (39 mg, 52%). Beige solid. mp—200–202 °C. **FTIR (cm^−1^):** 3283, 3104, 3055, 2971, 2887, 2847, 1629, 1589, 1508, 1494, 1417, 1242, 1108, 1077, 988, 891, 822. **^1^H NMR (400 MHz, CDCl_3_)** δ 7.33 (d, *J* = 8.8 Hz, 2H), 7.30–7.24 (m, 3H), 6.41 (s, 1H), 3.79–3.71 (m, 4H), 3.49 (t, *J* = 4.9 Hz, 4H). **^13^C NMR (101 MHz, CDCl_3_)** δ 154.8, 137.3, 128.9, 128.3, 121.2, 66.4, 44.2. **HRMS (ESI):** Exact mass calculated for C_11_H_14_ClN_2_O_2_ [M + H]^+^: 241.0739, found: 241.0741.

***N*-(2-bromophenyl)thiomorpholine-4-carboxamide** (**7m**): General procedure was followed using **1m** (75 mg, 0.37 mmol, 1.0 equiv.), **6a** (76 mg, 0.74 mmol, 2 equiv.), PhI(OAc)_2_ (238 mg, 2 equiv.), K_3_PO_4_ (157 mg, 2 equiv.), 1,2-DCE (2 mL), at 80 °C for 18 h. Column chromatography was performed using 85/15 *v/v* petroleum ether/acetone as eluent and yielded **7m** (100 mg, 90%). Light brown solid. mp—122–123 °C. **FTIR (cm^−1^):** 3335, 2960, 1568, 1627, 1507, 1043, 1015, 949, 744. **^1^H NMR (400 MHz, CDCl_3_) δ** 8.03 (dd, *J* = 8.3, 1.6 Hz, 1H), 7.42 (dd, *J* = 8.0, 1.5 Hz, 1H), 7.25–7.16 (m, 1H), 6.91 (s, 1H), 6.82 (td, *J* = 7.7, 1.6 Hz, 1H), 3.79–3.72 (m, 4H), 2.64–2.57 (m, 4H). **^13^C NMR (101 MHz, CDCl_3_) δ** 153.6, 136.6, 131.9, 128.3, 123.9, 121.5, 113.5, 47.1, 27.0. **HRMS (ESI):** Exact mass calculated for C_11_H_14_BrN_2_OS [M + H]^+^: 301.0005, found: 301.0007.

***N*-(4-bromophenyl)thiomorpholine-4-carboxamide** (**7n**): General procedure was followed using **1n** (75 mg, 0.37 mmol, 1.0 equiv.), **6a** (76 mg, 0.74 mmol, 2 equiv.), PhI(OAc)_2_ (238 mg, 2 equiv.), K_3_PO_4_ (157 mg, 2 equiv.), 1,2-DCE (2 mL), at 80 °C for 18 h. Column chromatography was performed using 85/15 *v/v* petroleum ether/acetone as eluent and yielded **7n** (88 mg, 79%). Brown solid. mp—212–214 °C. **FTIR (cm^−1^):** 3316, 2958, 2902, 1631, 1583, 1510, 1486, 1410, 1306, 1218, 1068, 1004, 940, 798, 746. **^1^H NMR (400 MHz, CDCl_3_) δ** 7.32 (d, J = 8.3 Hz, 2H), 7.22–7.14 (m, 2H), 6.25 (s, 1H), 3.77–3.65 (m, 4H), 2.66–2.53 (m, 4H). **^13^C NMR (101 MHz, CDCl_3_)** δ 153.1, 136.9, 130.8, 120.5, 114.8, 46.0, 26.0. **HRMS (ESI):** Exact mass calculated for C_11_H_14_BrN_2_OS [M + H]^+^: 301.0005, found: 301.0004.

***N*-(4-bromophenyl)morpholine-4-carboxamide (7n′):** General procedure was followed using **1n** (75 mg, 0.37 mmol, 1.0 equiv.), **6b** (64 mg, 0.74 mmol, 2 equiv.), PhI(OAc)_2_ (238 mg, 2 equiv.), K_3_PO_4_ (157 mg, 2 equiv.), 1,2-DCE (2 mL), at 80 °C for 18 h. Column chromatography was performed using 85/15 *v/v* petroleum ether/acetone as eluent and yielded **7n′** (29 mg, 40%). Colorless solid. mp—195–197 °C. **FTIR (cm^−1^):** 3289, 2969, 2887, 2847, 1631, 1585, 1510, 1488, 1413, 1300, 1242, 1110, 1063, 993, 818, 738. **^1^H NMR (400 MHz, CDCl_3_)** δ 7.32 (d, J = 8.3 Hz, 2H), 7.24–7.03 (m, 3H), 6.25 (s, 1H), 3.84–3.59 (m, 4H), 2.76–2.46 (m, 4H). **^13^C NMR (101 MHz, CDCl_3_) δ** 154.7, 137.8, 131.8, 121.5, 115.8, 66.4, 44.2. **HRMS (ESI):** Exact mass calculated for C_11_H_14_BrN_2_O_2_ [M + H]^+^: 285.0234, found: 285.0236.

***N*-(thiomorpholinyl)-2-Trifluoromethylbenzamide** (**7o**): General procedure was followed using **1o** (70 mg, 0.37 mmol, 1.0 equiv.), **6a** (76 mg, 0.74 mmol, 2 equiv.), PhI(OAc)_2_ (238 mg, 2 equiv.), K_3_PO_4_ (157 mg, 2 equiv.), 1,2-DCE (2 mL), at 80 °C for 18 h. Column chromatography was performed using 85/15 *v/v* petroleum ether/acetone as eluent and yielded **7o** (81 mg, 80%). Brown solid. mp—115–116 °C. **FTIR (cm^−1^):** 3265, 2960, 2909, 1627, 1583, 1516, 1395, 1311, 1247, 1163, 1052, 949, 749. **^1^H NMR (400 MHz, CDCl_3_)** δ 8.03 (d, *J* = 8.3 Hz, 1H), 7.63–7.49 (m, 2H), 7.22–7.13 (m, 1H), 6.77 (s, 1H), 3.85–3.77 (m, 4H), 2.73–2.63 (m, 4H). **^13^C NMR (101 MHz, CDCl_3_) δ** 153.8, 136.7, 136.6, 132.8, 128.5, 126.0, 125.9, 125.8, 124.3, 123.3, 123.1, 120.1, 119.8, 119.5, 119.2, 47.1, 26.9. ^19^F NMR (376 MHz, CDCl_3_) δ −60.5. **HRMS (ESI):** Exact mass calculated for C_12_H_14_F_3_N_2_OS [M + H]^+^: 291.0774, found: 291.0776.

***N*-(2-(trifluoromethyl)phenyl)morpholine-4-carboxamide** (**7o’**): General procedure was followed using **1o** (70 mg, 0.37 mmol, 1.0 equiv.), **6b** (64 mg, 0.74 mmol, 2 equiv.), PhI(OAc)_2_ (238 mg, 2 equiv.), K_3_PO_4_ (157 mg, 2 equiv.), 1,2-DCE (2 mL), at 80 °C for 18 h. Column chromatography was performed using 85/15 *v/v* petroleum ether/acetone as eluent and yielded **7o’** (72 mg, 98%). Brown solid. mp—115–116 °C. **FTIR (cm^−1^):** 3265, 2960, 2909, 1627, 1583, 1516, 1395, 1311, 1247, 1163, 1052, 947, 749. **^1^H NMR (400 MHz, CDCl_3_)** δ 8.11 (d, *J* = 8.3 Hz, 1H), 7.62–7.51 (m, 2H), 7.17 (t, *J* = 7.7 Hz, 1H), 6.80 (s, 1H), 3.82–3.73 (m, 4H), 3.55–3.46 (m, 4H). **^13^C NMR (101 MHz, CDCl_3_)** δ 154.3, 136.6, 136.6, 136.6, 136.6, 132.8, 126.0, 125.9, 125.8, 123.9, 123.2, 123.1, 119.8, 119.5, 119.2, 118.9, 66.4, 44.1. **^19^F NMR (376 MHz, CDCl_3_)** δ −60.58. **HRMS (ESI):** Exact mass calculated for C_12_H_14_F_3_N_2_O_2_ [M + H]^+^: 275.1002, found: 275.1005.

***N*-(3-(trifluoromethyl)phenyl)thiomorpholine-4-carboxamide** (**7p**): General procedure was followed using **1p** (70 mg, 0.37 mmol, 1.0 equiv.), **6a** (76 mg, 0.74 mmol, 2 equiv.), PhI(OAc)_2_ (238 mg, 2 equiv.), K_3_PO_4_ (157 mg, 2 equiv.), 1,2-DCE (2 mL), at 80 °C for 18 h. Column chromatography was performed using 85/15 *v/v* petroleum ether/acetone as eluent and yielded **7p** (70 mg, 65%). Brown solid. mp—127–129 °C. **FTIR (cm^−1^):** 3303, 3048, 2964, 2916, 1638, 1594, 1521, 1435, 1101, 1061, 973, 787. **^1^H NMR (400 MHz, CDCl_3_) δ** 7.51 (d, *J* = 2.1 Hz, 1H), 7.44 (dd, *J* = 8.1, 2.2 Hz, 1H), 7.28 (t, *J* = 7.9 Hz, 1H), 7.23–7.16 (m, 1H), 6.74 (s, 1H), 3.75–3.66 (m, 4H), 2.61–2.51 (m, 4H). **^13^C NMR (101 MHz, CDCl_3_) δ** 154.3, 139.5, 131.6, 131.3, 130.9, 130.6, 129.3, 123.3, 123.2, 119.8, 119.7, 116.8, 116.7, 47.0, 27.1. **^19^F NMR (376 MHz, CDCl_3_) δ** −62.6. **HRMS (ESI):** Exact mass calculated for C_12_H_14_F_3_N_2_OS [M + H]^+^: 291.0774, found: 291.0776.

***N*-(3,5-bis(trifluoromethyl)phenyl)thiomorpholine-4-carboxamide** (**7q**): General procedure was followed using **1’** (95 mg, 0.37 mmol, 1.0 equiv.), **6a** (76 mg, 0.74 mmol, 2 equiv.), PhI(OAc)_2_ (238 mg, 2 equiv.), K_3_PO_4_ (157 mg, 2 equiv.), 1,2-DCE (2 mL), at 80 °C for 18 h. Column chromatography was performed using 85/15 *v/v* petroleum ether/acetone as eluent and yielded **7q** (103 mg, 78%). Brwon solid. mp—140–141 °C. **FTIR (cm-1):** 3327, 2912, 1638, 1538, 1446, 1367, 1268, 1167, 1116, 973, 884. **^1^H NMR (400 MHz, CDCl_3_) δ** 7.61 (s, 2H), 7.34 (d, *J* = 5.6 Hz, 2H), 3.80–3.65 (m, 4H), 2.62–2.50 (m, 4H). **^13^C NMR (101 MHz, CDCl_3_) δ** 153.1, 139.4, 131.3, 130.9, 130.6, 130.3, 126.1, 123.4, 120.7, 118.9, 118.8, 118.8, 118.7, 118.0, 115.3, 115.3, 115.2, 115.2, 115.2, 45.9, 26.1. **^19^F NMR (376 MHz, CDCl_3_) δ** −63.1. **HRMS (ESI):** Exact mass calculated for C_13_H_13_F_6_N_2_OS [M + H]^+^: 359.0648, found: 359.0650.

***N*-(naphthalen-2-yl)thiomorpholine-4-carboxamide** (**7r**): General procedure was followed using **1r** (64 mg, 0.37 mmol, 1.0 equiv.), **6a** (76 mg, 0.74 mmol, 2 equiv.), PhI(OAc)_2_ (238 mg, 2 equiv.), K_3_PO_4_ (157 mg, 2 equiv.), 1,2-DCE (2 mL), at 80 °C for 18 h. Column chromatography was performed using 85/15 *v/v* petroleum ether/acetone as eluent and yielded **7r** (40 mg, 40%). Brown solid. mp—187–188 °C. **FTIR (cm^−1^):** 3298, 3057, 2961, 2911, 1628, 1599, 1535, 1392, 1016, 943, 786, 759. **^1^H NMR (400 MHz, CDCl_3_) δ** 7.75 (d, *J* = 13.4 Hz, 2H), 7.60 (d, *J* = 8.1 Hz, 1H), 7.40 (m, 4H), 6.63 (s, 1H), 3.76–3.58 (m, 4H), 2.52 (s, 4H). **^13^C NMR (101 MHz, CDCl_3_) δ** 155.7, 134.2, 133.9, 128.6, 128.4, 126.1, 125.9, 125.7, 125.5, 121.4, 121.3, 47.1, 27.0. **HRMS (ESI):** Exact mass calculated for C_15_H_17_N_2_O_2_S [M + H]^+^: 273.1057, found: 273.1058.

***N*-([1,1’-biphenyl]-4-yl)thiomorpholine-4-carboxamide** (**7s**): General procedure was followed using **1s** (64 mg, 0.37 mmol, 1.0 equiv.), **6a** (76 mg, 0.74 mmol, 2 equiv.), PhI(OAc)_2_ (238 mg, 2 equiv.), K_3_PO_4_ (157 mg, 2 equiv.), 1,2-DCE (2 mL), at 80 °C for 18 h. Column chromatography was performed using 85/15 *v/v* petroleum ether/acetone as eluent and yielded **7s** (40 mg, 40%). Beige solid. mp—202–204 °C. **FTIR (cm^−1^):** 3318, 2958, 2909, 1627, 1516, 1399, 1306, 1245, 1220, 1196, 1169, 1085, 942, 811, 760, 693. **^1^H NMR (400 MHz, CDCl_3_) δ** 7.58–7.50 (m, 5H), 7.41 (ddd, *J* = 8.0, 4.4, 2.0 Hz, 4H), 7.34–7.28 (m, 1H), 6.53 (s, 1H), 3.82–3.77 (m, 4H), 2.70–2.64 (m, 4H). **^13^C NMR (101 MHz, CDCl_3_) δ** 154.61, 140.59, 138.19, 136.18, 128.74, 127.53, 126.94, 126.77, 120.40, 47.05, 27.10. **HRMS (ESI):** Exact mass calculated for C_17_H_19_N_2_OS [M + H]^+^: 299.1213, found: 299.1210.

**(3*R*,4*S*)-3-((benzo[*d*][1,3]dioxol-5-yloxy)methyl)-4-(4-fluorophenyl)-*N*-(*p*-tolyl)piperidine-1-carboxamide** (**9a**): General procedure was followed using **1a** (50 mg, 0.37 mmol, 1.0 equiv.), **8a** (182 mg, 0.55 mmol, 1.5 equiv.), PhI(OAc)_2_ (238 mg, 2 equiv.), K_3_PO_4_ (157 mg, 2 equiv.), 1,2-DCE (2 mL), at 80 °C for 18 h. Column chromatography was performed using 85/15 *v/v* petroleum ether/acetone as eluent and yielded **9a** (87 mg, 51%). Brown oily liquid. **FTIR (cm^−1^):** 3301, 2915, 1632, 1592, 1505, 1483, 1217, 1175, 1031, 929, 810, 723. **^1^H NMR (400 MHz, CDCl_3_) δ** 7.32–7.26 (m, 2H), 7.19–7.08 (m, 4H), 7.01 (t, *J* = 8.7 Hz, 2H), 6.70–6.63 (m, 2H), 6.38 (d, *J* = 2.5 Hz, 1H), 6.17 (dd, *J* = 8.5, 2.5 Hz, 1H), 5.90 (s, 2H), 4.43–4.21 (m, 2H), 3.64 (dd, *J* = 9.5, 2.9 Hz, 1H), 3.50 (dd, *J* = 9.5, 6.7 Hz, 1H), 2.98 (dd, *J* = 13.5, 11.0 Hz, 2H), 2.79–2.68 (m, 1H), 2.32 (s, 3H), 2.19 (s, 2H), 2.13 (tq, *J* = 7.7, 4.0 Hz, 1H), 2.08 (s, 1H), 1.92–1.72 (m, 2H), 1.38–1.26 (m, 1H), 0.95–0.85 (m, 3H). **^13^C NMR (101 MHz, CDCl_3_) δ** 162.8, 160.4, 155.3, 154.1, 148.2, 141.8, 138.7 (*J* = 3.7 Hz), 136.5, 132.6, 129.4, 128.8 (*J* = 8.7 Hz), 120.3, 115.7 (*J* = 26.2 Hz), 107.9, 105.6, 101.1, 98.0, 68.7, 47.9, 44.9, 44.0, 41.9, 33.8, 30.9, 20.7. **^19^F NMR (376 MHz, CDCl_3_) δ** −115.8, −115.8, −115.8, −115.9, −115.9. **HRMS (ESI):** Exact mass calculated for C_27_H_28_FN_2_O_4_ [M + H]^+^: 463.2028, found: 463.2030.

**1-((1*R*,3*R*,5*S*)-3,5-dimethyladamantan-1-yl)-3-(*p*-tolyl)urea** (**9b**): General procedure was followed using **1a** (50 mg, 0.37 mmol, 1.0 equiv.), **8b** (132 mg, 2 equiv.), PhI(OAc)_2_ (238 mg, 2 equiv.), K_3_PO_4_ (157 mg, 2 equiv.), 1,2-DCE (2 mL), at 80 °C for 18 h. Column chromatography was performed using 85/15 *v/v* petroleum ether/acetone as eluent and yielded **9b** (50 mg, 44%). Brown solid. mp—152–154 °C. **FTIR (cm^−1^):** 3323, 2891, 2838, 1643, 1594, 1556, 1512, 1452, 1310, 1295, 1224, 1031, 816. **^1^H NMR (400 MHz, CDCl3) δ** 7.15 (d, *J* = 8.4 Hz, 2H), 7.06 (d, *J* = 8.3 Hz, 2H), 6.74 (s, 1H), 4.95 (s, 1H), 2.28 (s, 3H), 2.13–2.07 (m, 1H), 1.80 (d, *J* = 3.2 Hz, 2H), 1.66–1.54 (m, 5H), 1.34 (dt, *J* = 12.1, 2.5 Hz, 3H), 1.30–1.23 (m, 3H), 1.16–1.08 (m, 3H), 0.82 (s, 7H). **^13^C NMR (101 MHz, CDCl_3_) δ** 155.2, 136.4, 132.8, 129.6, 121.0, 52.7, 50.6, 48.2, 42.7, 40.7, 32.4, 30.2, 30.1, 20.7. **HRMS (ESI):** Exact mass calculated for C_20_H_29_N_2_O [M + H]^+^: 313.2275, found: 313.2274.

***N*2-(2,6-dimethylphenyl)-*N*1-(p-tolyl)piperidine-1,2-dicarboxamide** (**9c**): General procedure was followed using **1a** (50 mg, 0.37 mmol, 1.0 equiv.), **8c** (171 mg, 0,74 mmol, 2 equiv.), PhI(OAc)_2_ (238 mg, 2 equiv.), K_3_PO_4_ (157 mg, 2 equiv.), 1,2-DCE (2 mL), at 80 °C for 18 h. Column chromatography was performed using 85/15 *v/v* petroleum ether/acetone as eluent and yielded **9c** (52 mg, 39%). Colorless solid. mp—218–220 °C. **FTIR (cm^−1^):** 3219, 2935, 2857, 1638, 1592, 1525, 1512, 1446, 1406, 1379, 1344, 1297, 1224, 1036, 821, 767. **^1^H NMR (400 MHz, CDCl_3_) δ** 7.91 (s, 1H), 7.16 (d, *J* = 8.2 Hz, 3H), 7.04–6.98 (m, 8H), 6.47 (s, 1H), 5.07–5.03 (m, 1H), 3.70 (dt, *J* = 13.5, 3.6 Hz, 2H), 3.23 (td, *J* = 12.6, 2.6 Hz, 1H), 2.29 (dd, *J* = 8.9, 5.7 Hz, 2H), 2.23 (s, 3H), 2.17 (d, *J* = 2.1 Hz, 1H), 2.13 (s, 6H). **^13^C NMR (101 MHz, CDCl_3_) δ** 170.1, 156.5, 135.8, 135.0, 133.8, 133.4, 129.6, 129.5, 128.3, 128.1, 127.0, 126.0, 120.6, 53.7, 43.5, 25.4, 25.0, 20.7, 20.3, 18.8, 18.6, 18.4, 14.0. **HRMS (ESI):** Exact mass calculated for C_22_H_28_N_3_O_2_ [M + H]^+^: 366.2177, found: 366.2178.

**1-(((1*R*,4a*S*,10a*R*)-7-isopropyl-1,4*a*-dimethyl-1,2,3,4,4*a*,9,10,10*a*-octahydrophenanthren-1-yl)methyl)-3-(*p*-tolyl)urea (9d**): General procedure was followed using **1a** (50 mg, 0.37 mmol, 1.0 equiv.), **8d** (211 mg, 0.74 mmol, 2 equiv.), PhI(OAc)_2_ (238 mg, 2 equiv.), K_3_PO_4_ (157 mg, 2 equiv.), 1,2-DCE (2 mL), at 80 °C for 18 h. Column chromatography was performed using 85/15 *v/v* petroleum ether/acetone as eluent and yielded **9d** (89 mg, 58%). Colorless solid. mp—209–211 °C. **FTIR (cm^−1^):** 3325, 2948, 2913, 1687, 1641, 1592, 1512, 1308, 1295, 1235, 1082, 1036, 815, 667. **^1^H NMR (400 MHz, CDCl_3_) δ** 7.12 (dd, *J* = 12.3, 8.3 Hz, 3H), 7.01 (d, *J* = 8.1 Hz, 2H), 6.97 (dd, *J* = 8.1, 2.1 Hz, 1H), 6.85–6.81 (m, 2H), 5.14 (t, *J* = 6.4 Hz, 1H), 3.08 (t, *J* = 6.4 Hz, 2H), 2.88–2.68 (m, 4H), 2.25 (s, 3H), 1.86–1.76 (m, 2H), 1.76–1.52 (m, 5H), 1.44–1.25 (m, 5H), 1.21 (s, 4H), 1.19 (s, 3H), 1.17 (s, 3H), 0.87 (s, 3H). **^13^C NMR (101 MHz, CDCl_3_) δ** 156.5, 147.2, 145.5, 136.0, 134.8, 133.4, 129.7, 126.8, 124.2, 123.7, 121.5, 50.6, 45.4, 38.4, 37.5, 37.4, 36.1, 33.4, 30.2, 25.2, 24.0, 20.8, 18.9, 18.6, 18.5. **HRMS (ESI):** Exact mass calculated for C_28_H_39_N_2_O [M + H]^+^: 419.3057, found: 419.3056.

**1-((1*S*,4*S*)-4-(3,4-dichlorophenyl)-1,2,3,4-tetrahydronaphthalen-1-yl)-1-methyl-3-(*p*-tolyl)urea** (**9e**): General procedure was followed using **1a** (44 mg, 0.32 mmol, 2.0 equiv.), **8e** (50 mg, 0.16 mmol, 1 equiv.), PhI(OAc)_2_ (238 mg, 2 equiv.), K_3_PO_4_ (157 mg, 2 equiv.), 1,2-DCE (2 mL), at 80 °C for 18 h. Column chromatography was performed using 85/15 *v/v* petroleum ether/acetone as eluent and yielded **9e** (32 mg, 18%). Orange solid. mp—151–153 °C. **FTIR (cm^−1^):** 3290, 3028, 2942, 2855, 1632, 1590, 1512, 1408, 1290, 1120, 1051, 927, 803, 750. **^1^H NMR (400 MHz, CDCl_3_) δ** 7.36–7.30 (m, 3H), 7.30–7.24 (m, 2H), 7.22–7.17 (m, 1H), 7.13–7.07 (m, 3H), 6.97 (dd, *J* = 7.6, 1.2 Hz, 1H), 6.85 (dd, *J* = 8.3, 2.1 Hz, 1H), 6.40 (s, 1H), 5.70 (dd, *J* = 10.9, 6.1 Hz, 1H), 4.20 (dd, *J* = 5.7, 2.9 Hz, 1H), 2.78 (s, 3H), 2.30 (s, 3H), 2.07–1.96 (m, 1H), 1.81 (dt, *J* = 5.7, 3.8 Hz, 1H), 1.73 (ddd, *J* = 13.3, 10.7, 2.8 Hz, 1H), 0.92–0.79 (m, 2H). **^13^C NMR (101 MHz, CDCl_3_) δ** 156.1, 147.1, 138.2, 136.7, 136.4, 132.7, 132.3, 130.8, 130.6, 130.1, 129.4, 128.0, 127.5, 127.4, 120.1, 54.1, 43.0, 30.4, 30.1, 22.6, 22.2, 20.7. **HRMS (ESI):** Exact mass calculated for C_25_H_25_Cl_2_N_2_O [M + H]^+^: 439.1339, found: 439.1341.

**3-(3,5-bis(trifluoromethyl)phenyl)-1-((1*S*,4*S*)-4-(3,4-dichlorophenyl)-1,2,3,4-tetrahydronaphthalen-1-yl)-1-methylurea** (**9e’**): General procedure was followed using **1′** (95 mg, 0.32 mmol, 2.0 equiv.), **8e** (46 mg, 0.16 mmol, 1 equiv.), PhI(OAc)_2_ (238 mg, 2 equiv.), K_3_PO_4_ (157 mg, 2 equiv.), 1,2-DCE (2 mL), at 80 °C for 18 h. Column chromatography was performed using 85/15 *v/v* petroleum ether/acetone as eluent and yielded **9e’** (84 mg, 40%). Orange sticky solid. **FTIR (cm^−1^):** 3320, 2936, 1642, 1539, 1466, 1446, 1368, 1273, 1119, 1028, 953, 874, 731, 699, 679. **^1^H NMR (400 MHz, CDCl_3_) δ** 7.98 (s, 2H), 7.53 (s, 1H), 7.35 (d, J = 8.3 Hz, 1H), 7.31–7.20 (m, 4H), 7.10 (d, J = 2.1 Hz, 1H), 7.00 (d, J = 7.3 Hz, 1H), 6.86 (dd, J = 8.4, 2.1 Hz, 2H), 2.84 (s, 3H), 2.32 (tdd, J = 13.4, 5.7, 2.9 Hz, 1H), 2.17 (s, 4H), 2.05 (dt, J = 13.7, 2.7 Hz, 1H), 1.87–1.68 (m, 2H). **^13^C NMR (101 MHz, CDCl_3_) δ** 155.3, 146.9, 140.6, 138.3, 135.9, 132.6, 132.4, 132.3, 131.9, 131.6, 131.0, 130.6, 130.2, 130.2, 127.9, 127.7, 127.6, 127.2, 124.5, 121.8, 119.4, 116.3, 42.9, 30.9, 30.5, 30.0, 22.2. ^19^F NMR (376 MHz, CDCl_3_) δ −62.99. **HRMS (ESI):** Exact mass calculated for C_26_H_21_Cl_2_F_6_N_2_O [M + H]^+^: 561.0930, found: 561.0933.

**(*S*)-1-(4-oxo-4-(3-(trifluoromethyl)-5,6-dihydro-[1,2,4]triazolo[4,3-a]pyrazin-7(8*H*)-yl)-1-(2,4,5-trifluorophenyl)butan-2-yl)-3-(*p*-tolyl)urea (9f)**: General procedure was followed using **1a** (30 mg, 0.22 mmol, 1.0 equiv.), **8f** (90 mg, 0.22 mmol, 1 equiv.), PhI(OAc)_2_ (141 mg, 2 equiv.), K_3_PO_4_ (93 mg, 2 equiv.), 1,2-DCE (2 mL), at 80 °C for 18 h. Column chromatography was performed using 100/4 *v*/*v* dichloromethane/methanol as eluent and yielded **9f** (30 mg, 12%). Brown solid. mp—122–124 °C. FTIR (cm^−1^): 3325, 1654, 1512, 1419, 1268, 1250, 1138, 1013, 725. ^1^H NMR (400 MHz, DMSO-*d*_6_) δ 8.44 (d, *J* = 23.1 Hz, 1H), 7.55–7.39 (m, 2H), 7.21 (d, *J* = 8.7 Hz, 2H), 7.03 (d, *J* = 8.1 Hz, 2H), 6.24 (dd, *J* = 21.9, 9.0 Hz, 1H), 5.07 (d, *J* = 7.2 Hz, 1H), 4.96 (s, 1H), 4.31 (td, *J* = 18.1, 14.8, 9.5 Hz, 3H), 4.14 (d, *J* = 5.8 Hz, 1H), 4.05 (t, *J* = 6.0 Hz, 2H), 2.93 (t, *J* = 6.7 Hz, 2H), 2.86–2.76 (m, 2H), 2.25 (s, 3H), 2.15 (s, 2H). ^13^C NMR (101 MHz, DMSO-*d*_6_) δ 170.2, 170.1, 155.1, 151.4, 138.2, 130.2, 129.4, 120.2, 118.1, 106.2, 79.7, 79.4, 79.1, 47.0, 44.0, 42.4, 41.5, 38.7, 37.8, 31.1, 20.7. ^19^F NMR (376 MHz, DMSO-*d*_6_) δ −61.87, −61.88, −118.50, −118.51, −118.53, −118.55, −118.56, −118.57, −137.30, −137.31, −137.33, −137.35, −137.36, −137.39, −144.04, −144.05, −144.07, −144.08, −144.10. HRMS (ESI): Exact mass calculated for C_24_H_23_F_6_N_6_O_2_ [M + H]^+^: 541.1782, found: 541.1781.

**(*S*)-1-(3,5-bis(trifluoromethyl)phenyl)-3-(4-oxo-4-(3-(trifluoromethyl)-5,6-dihydro-[1,2,4]triazolo[4,3-a]pyrazin-7(8*H*)-yl)-1-(2,4,5-trifluorophenyl)butan-2-yl)urea (9f′):** General procedure was followed using **1’** (95 mg, 0.22 mmol, 1.0 equiv.), **8f** (226 mg, 0.22 mmol, 1 equiv.), PhI(OAc)_2_ (141 mg, 2 equiv.), K_3_PO_4_ (93 mg, 2 equiv.), 1,2-DCE (2 mL), at 80 °C for 18 h. Column chromatography was performed using 100/4 *v*/*v* dichloromethane/methanol as eluent and yielded **9f’** (207 mg, 85%). Brown solid. mp—122–124 °C. FTIR (cm^−1^): 3325, 1654, 1512, 1419, 1268, 1250, 1138, 1013, 725. ^1^H NMR (400 MHz, DMSO-*d*_6_) δ 8.54 (s, 1H), 8.04 (s, 2H), 7.82 (s, 1H), 7.41 (s, 1H), 7.35 (d, J = 9.2 Hz, 1H), 7.16–7.06 (m, 2H), 6.87 (td, J = 9.5, 6.4 Hz, 2H), 6.08 (d, J = 17.8 Hz, 1H), 5.09 (t, J = 15.1 Hz, 1H), 5.00–4.88 (m, 1H), 4.78 (d, J = 18.0 Hz, 1H), 4.51 (s, 1H), 4.41–4.08 (m, 7H), 3.90 (ddd, J = 13.9, 8.8, 4.5 Hz, 2H), 3.25 (dd, J = 13.6, 8.4 Hz, 1H), 3.05 (dd, J = 13.9, 6.8 Hz, 1H), 3.00–2.85 (m, 2H), 2.82–2.70 (m, 2H). ^13^C NMR (101 MHz, DMSO-*d*_6_) δ 170.0, 156.0, 155.9, 154.7, 151.1, 141.4, 132.0, 131.7, 129.0, 128.0, 124.6, 121.9, 119.2 (*J* = 10 Hz), 118.1, 117.7, 116.8, 114.3, 109.9, 105.7, 105.4, 105.2, 103.6, 47.4, 43.8, 41.5, 38.8, 35.3, 32.6, 30.9. ^19^F NMR (376 MHz, DMSO-*d*_6_) δ −63.14, −119.76, −119.78, −119.81, −119.83, −134.88, −135.21, −135.25, −135.27, −142.49, −142.52, −142.54, −142.57. HRMS (ESI): Exact mass calculated for C_25_H_19_F_12_N_6_O_2_ [M + H]^+^: 663.1372, found: 663.1373.

**1-methyl-1-(3-phenyl-3-(4-(trifluoromethyl)phenoxy)propyl)-3-(*p*-tolyl)urea** (**9g**): General procedure was followed using **1a** (50 mg, 0.37 mmol, 1.0 equiv.), **8g** (121 mg, 0.74 mmol, 2 equiv.), PhI(OAc)_2_ (238 mg, 2 equiv.), K_3_PO_4_ (157 mg, 2 equiv.), 1,2-DCE (2 mL), at 80 °C for 18 h. Column chromatography was performed using 85/15 *v/v* petroleum ether/acetone as eluent and yielded **9g** (26 mg, 27%). Yellow oily liquid. **FTIR (cm^−1^):** 3321, 2922, 1641, 1610, 1512, 1317, 1239, 1153, 1107, 1064, 832, 810, 707. **^1^H NMR (400 MHz, CDCl_3_)** δ 7.36–7.31 (m, 3H), 7.26 (d, *J* = 4.4 Hz, 5H), 7.22–7.17 (m, 2H), 6.99–6.90 (m, 4H), 6.83 (d, *J* = 8.3 Hz, 2H), 6.20 (s, 1H), 5.20 (dd, *J* = 8.5, 4.3 Hz, 1H), 3.59–3.42 (m, 2H), 2.92 (s, 3H), 2.19 (s, 4H), 2.17–2.08 (m, 2H), 0.89–0.75 (m, 2H). **^13^C NMR (101 MHz, CDCl_3_)** δ 160.0, 155.5, 140.3, 136.2, 132.5, 129.2, 128.9, 128.0, 126.9, 125.7, 123.3, 123.0, 122.9, 120.0, 115.8, 77.6, 45.9, 37.1, 34.7, 23.8, 22.6, 20.7. **^19^F NMR (376 MHz, CDCl_3_)** δ -61.6. **HRMS (ESI):** Exact mass calculated for C_25_H_26_F_3_N_2_O_2_ [M + H]^+^: 443.1941, found: 443.1941.

**3-(3,5-bis(trifluoromethyl)phenyl)-1-methyl-1-(3-phenyl-3-(4-(trifluoromethyl)phenoxy)propyl)urea** (**9g′**): General procedure was followed using **1′** (95 mg, 0.37 mmol, 1.0 equiv.), **8g** (0,19 mg, 0.74 mmol, 1.5 equiv.), PhI(OAc)_2_ (238 mg, 2 equiv.), K_3_PO_4_ (157 mg, 2 equiv.), 1,2-DCE (2 mL), at 80 °C for 18 h. Column chromatography was performed using 85/15 *v/v* petroleum ether/acetone as eluent and yielded **9g’** (208 mg, 37%). Yellow oily liquid. FTIR (cm^−1^): 3318, 2936, 1649, 1616, 1539, 1472, 1364, 1324, 1273, 1108, 1066, 833, 697, 680. **^1^H NMR (400 MHz, CDCl_3_)** δ 7.68 (s, 2H), 7.43 (s, 1H), 7.41–7.37 (m, 2H), 7.36–7.31 (m, 4H), 7.30–7.25 (m, 1H), 6.93–6.85 (m, 3H), 5.30 (dd, J = 8.4, 4.3 Hz, 1H), 3.69 (dt, J = 15.0, 7.5 Hz, 1H), 3.61–3.52 (m, 1H), 3.05 (s, 3H), 2.30–2.19 (m, 2H). **^13^C NMR (101 MHz, CDCl_3_)** δ 159.5, 154.7, 140.6, 139.9, 132.4, 132.0, 131.7, 131.4, 129.0, 128.3, 127.0, 126.9, 125.7, 124.5, 123.7, 123.4, 122.7, 121.8, 119.0, 119.0, 115.8, 45.9, 36.8, 34.6, 30.9. **^19^F NMR (376 MHz, CDCl_3_)** δ −61.84, −63.06. **HRMS (ESI):** Exact mass calculated for C_26_H_22_F_9_N_2_O_2_ [M + H]^+^: 565.1533, found: 565.1534.

**4-(8-chloro-5,6-dihydro-11*H*-benzo[5,6]cyclohepta[1,2-*b*]pyridin-11-ylidene)-*N*-(p-tolyl)piperidine-1-carboxamide (9h**): General procedure was followed using **1a** (50 mg, 0.37 mmol, 1.0 equiv.), **8h** (172 mg, 0.74 mmol, 2 equiv.), PhI(OAc)_2_ (238 mg, 2 equiv.), K_3_PO_4_ (157 mg, 2 equiv.), 1,2-DCE (2 mL), at 80 °C for 18 h. Column chromatography was performed using 85/15 *v/v* petroleum ether/acetone as eluent and yielded **9h** (29 mg, 18%). Beige solid. mp—203–205 °C. **FTIR (cm^−1^):** 3301, 2915, 1709, 1630, 1592, 1514, 1477, 1417, 1239, 1080, 993, 810. **^1^H NMR (400 MHz, CDCl_3_) δ** 8.34 (dd, *J* = 4.8, 1.7 Hz, 1H), 7.37 (dd, *J* = 7.7, 1.7 Hz, 1H), 7.20–7.08 (m, 4H), 7.08–7.03 (m, 3H), 7.03–6.97 (m, 2H), 6.27 (s, 1H), 3.74–3.64 (m, 2H), 3.30 (dtd, *J* = 16.1, 8.7, 7.7, 3.9 Hz, 2H), 3.17 (ddt, *J* = 12.8, 9.2, 3.8 Hz, 2H), 2.85–2.68 (m, 2H), 2.55 (ddd, *J* = 14.2, 9.1, 4.5 Hz, 1H), 2.40 (ddd, *J* = 13.9, 9.1, 4.6 Hz, 1H), 2.37–2.26 (m, 3H), 2.21 (s, 3H). **^13^C NMR (101 MHz, CDCl_3_) δ** 156.9, 155.0, 146.7, 139.5, 137.6, 136.9, 136.4, 134.5, 133.3, 133.0, 132.6, 130.5, 129.4, 129.2, 129.0, 127.4, 126.2, 122.3, 120.1, 60.4, 44.9, 44.8, 31.7, 31.5, 30.5, 30.3, 20.7, 14.2. **HRMS (ESI):** Exact mass calculated for C_27_H_27_ClN_3_O [M + H]^+^: 444.1843, found: 444.1847.

***N*-(3,5-bis(trifluoromethyl)phenyl)-4-(8-chloro-5,6-dihydro-11*H*-benzo[5,6]cyclohepta[1,2-b]pyridin-11-ylidene)piperidine-1-carboxamide (9h′):** General procedure was followed using **1’** (95 mg, 0.37 mmol, 1.0 equiv.), **8h** (172 mg, 0.74 mmol, 2 equiv.), PhI(OAc)_2_ (238 mg, 2 equiv.), K_3_PO_4_ (157 mg, 2 equiv.), 1,2-DCE (2 mL), at 80 °C for 18 h. Column chromatography was performed using 85/15 *v/v* petroleum ether/acetone as eluent and yielded **9h′** (121 mg, 58%). Brown sticky solid. FTIR (cm^−1^): 3314, 1645, 1550, 1439, 1368, 1273, 1220, 1167, 1121, 990, 880, 827, 726, 699, 680. ^1^H NMR (400 MHz, CDCl_3_) δ 8.40 (dd, J = 4.9, 1.7 Hz, 1H), 7.81 (d, J = 1.6 Hz, 2H), 7.49 (dd, J = 7.7, 1.7 Hz, 1H), 7.42 (d, J = 3.4 Hz, 2H), 7.18–7.07 (m, 4H), 3.80 (dq, J = 11.2, 5.3, 4.8 Hz, 2H), 3.43–3.22 (m, 4H), 2.92–2.76 (m, 2H), 2.58 (ddd, J = 13.9, 9.1, 4.5 Hz, 1H), 2.50–2.29 (m, 3H). ^13^C NMR (101 MHz, CDCl_3_) δ 156.6, 154.3, 146.2, 141.0, 139.4, 138.0, 137.1, 136.6, 134.2, 133.7, 133.1, 132.2, 131.9, 131.6, 131.3, 130.4, 129.1, 126.2, 124.5, 122.6, 121.8, 119.4, 115.7, 115.6, 44.8, 44.7, 31.6, 31.3, 30.9, 30.4, 30.2. HRMS (ESI): Exact mass calculated for C_28_H_23_ClF_6_N_3_O [M + H]^+^: 566.1429, found: 566.1425.

**4-(2-chlorodibenzo[*b*,*f*][1,4]oxazepin-11-yl)-*N*-(*p*-tolyl)piperazine-1-carboxamide** (**9i**): General procedure was followed using **1a** (85 mg, 0.62 mmol, 2.0 equiv.), **8i** (100 mg, 0.31 mmol, 1 equiv.), PhI(OAc)_2_ (256 mg, 2 equiv.), K_3_PO_4_ (135 mg, 2 equiv.), 1,2-DCE (2 mL), at 80 °C for 18 h. Column chromatography was performed using 85/15 *v/v* petroleum ether/acetone as eluent and yielded **9i** (46 mg, 28%). Orange solid. Mp—205–207 °C. **FTIR (cm^−1^):** 3292, 2917, 2849, 1638, 1585, 1508, 1459, 1397, 1233, 1100, 1013, 987, 807, 770. **^1^H NMR (400 MHz, CDCl_3_) δ** 7.34 (dd, *J* = 8.6, 2.6 Hz, 1H), 7.25 (d, *J* = 2.6 Hz, 1H), 7.16 (dd, *J* = 12.9, 4.5 Hz, 3H), 7.13–7.06 (m, 3H), 7.06–6.98 (m, 6H), 6.94 (td, *J* = 7.5, 1.8 Hz, 2H), 6.35 (s, 1H), 3.61–3.42 (m, 8H), 2.22 (s, 3H). **^13^C NMR (101 MHz, CDCl_3_) δ** 158.3, 157.6, 154.3, 150.7, 138.8, 135.0, 132.0, 131.7, 129.4, 128.6, 128.4, 127.8, 126.0, 124.8, 123.9, 123.7, 121.8, 119.4, 119.1, 46.1, 42.7, 19.7. **HRMS (ESI):** Exact mass calculated for C_25_H_24_ClN_4_O_2_ [M + H]^+^: 447.1583, found: 447.1588.

***N*-(3,5-bis(trifluoromethyl)phenyl)-4-(2-chlorodibenzo[*b*,*f*][1,4]oxazepin-11-yl)piperazine-1-carboxamide** (**9i′**): General procedure was followed using **1′** (95 mg, 0.62 mmol, 2.0 equiv.), **8i** (174 mg, 0.31 mmol, 2 equiv.), PhI(OAc)_2_ (256 mg, 2 equiv.), K_3_PO_4_ (135 mg, 2 equiv.), 1,2-DCE (2 mL), at 80 °C for 18 h. Column chromatography was performed using 85/15 *v/v* petroleum ether/acetone as eluent and yielded **9i’** (210 mg, 59%). Yellow sticky solid. **FTIR (cm^−1^):** 3320, 1654, 1598, 1552, 1468, 1360, 1273, 1169, 1123, 1015, 878, 827, 775, 727, 702, 679. **^1^H NMR (400 MHz, CDCl_3_) δ** 7.99 (s, 1H), 7.89 (s, 1H), 7.53–7.46 (m, 2H), 7.44–7.39 (m, 1H), 7.34 (dd, J = 17.1, 2.6 Hz, 1H), 7.20 (dd, J = 8.6, 1.9 Hz, 1H), 7.18–7.07 (m, 3H), 7.03 (dt, J = 7.7, 1.8 Hz, 1H), 5.11 (s, 1H), 3.82 (s, 1H), 3.63 (d, J = 35.3 Hz, 3H). **^13^C NMR (101 MHz, CDCl_3_) δ** 159.3, 159.1, 158.7, 156.1, 154.6, 152.5, 151.8, 151.7, 140.7, 140.4, 139.7, 139.5, 133.0, 132.9, 132.1, 131.8, 130.5, 130.5, 128.8, 128.4, 127.1, 127.0, 126.0, 125.9, 125.2, 125.1, 124.6, 124.5, 122.8, 122.6, 120.3, 120.2, 119.6, 119.4, 119.3, 62.6, 47.2, 44.3, 43.7. **HRMS (ESI):** Exact mass calculated for C_26_H_20_ClF_6_N_4_O_2_ [M + H]^+^: 569.1174, found: 569.1178.

**4-(pyrimidin-2-yl)-*N*-(*p*-tolyl)piperazine-1-carboxamide** (**9j**): General procedure was followed using **1a** (50 mg, 0.37 mmol, 1.0 equiv.), **8j** (91 mg, 0.74 mmol, 5 equiv.), PhI(OAc)_2_ (238 mg, 2 equiv.), K_3_PO_4_ (157 mg, 2 equiv.), 1,2-DCE (2 mL), at 80 °C for 18 h. Column chromatography was performed using 85/15 *v/v* petroleum ether/acetone as eluent and yielded **9j** (24 mg, 22%). Yellow solid. mp—167–169 °C. **FTIR (cm^−1^):** 3305, 2902, 2862, 1632, 1581, 1512, 1470, 1412, 1355, 1306, 1288, 1239, 1080, 978, 792. **^1^H NMR (400 MHz, CDCl_3_) δ** 8.26 (d, *J* = 4.7 Hz, 2H), 7.20–7.14 (m, 2H), 7.02 (d, *J* = 8.3 Hz, 2H), 6.47 (t, *J* = 4.7 Hz, 1H), 6.37 (s, 1H), 3.86–3.78 (m, 4H), 3.55–3.46 (m, 4H), 2.22 (s, 3H). **^13^C NMR (101 MHz, CDCl_3_) δ** 160.4, 156.7, 154.3, 135.1, 131.8, 128.4, 119.3, 109.3, 42.7, 42.3, 19.7. **HRMS (ESI):** Exact mass calculated for C_16_H_19_N_5_O [M+Na]^+^: 320.1482, found: 320.1490.

***N*-(3,5-bis(trifluoromethyl)phenyl)-4-(pyrimidin-2-yl)piperazine-1-carboxamide** (**9j′**): General procedure was followed using **1’** (95 mg, 0.37 mmol, 1.0 equiv.), **8j** (91 mg, 0.74 mmol, 1.5 equiv.), PhI(OAc)_2_ (238 mg, 2 equiv.), K_3_PO_4_ (157 mg, 2 equiv.), 1,2-DCE (2 mL), at 80 °C for 18 h. Column chromatography was performed using 85/15 *v/v* petroleum ether/acetone as eluent and yielded **9j’** (83 mg, 66%). Yellow solid. mp—159–161 °C. **FTIR (cm^−1^):** 3327, 2925, 2858, 1642, 1583, 1550, 1466, 1439, 1373, 1271, 1240, 1165, 1116, 997, 979, 944, 886, 796, 701, 680. **^1^H NMR (400 MHz, CDCl_3_) δ** 8.39 (d, J = 4.8 Hz, 2H), 7.98–7.95 (m, 2H), 7.53 (s, 1H), 6.71 (s, 1H), 6.67 (t, J = 4.8 Hz, 1H), 5.11 (s, 2H), 4.01–3.88 (m, 4H), 1.25 (s, 4H). **^13^C NMR (101 MHz, CDCl_3_) δ** 159.2, 158.0, 152.0, 140.1, 132.7, 132.4, 132.0, 131.7, 124.5, 121.8, 119.1, 116.5, 111.3, 61.1, 44.3. **^19^F NMR (376 MHz, CDCl_3_)** δ −63.04. **HRMS (ESI):** Exact mass calculated for C_17_H_16_F_6_N_5_O [M + H]^+^: 420.1254, found: 420.1264.

***N*-(*p*-tolyl)-3-(3,4,5-trimethoxybenzamido)piperidine-1-carboxamide** (**9k**): General procedure was followed using **1a** (91 mg, 0.68 mmol, 2.0 equiv.), **8k** (100 mg, 0.34 mmol, 1 equiv.), PhI(OAc)_2_ (238 mg, 2 equiv.), K_3_PO_4_ (157 mg, 2 equiv.), 1,2-DCE (2 mL), at 80 °C for 18 h. Column chromatography was performed using 85/15 *v/v* petroleum ether/acetone as eluent and yielded **9k** (42 mg, 27%). Colorless solid. mp—129–131 °C. **FTIR (cm^−1^):** 3312, 2937, 1627, 1579, 1523, 1499, 1408, 1337, 1233, 1126, 1084, 989, 838, 807. **^1^H NMR (400 MHz, CDCl_3_) δ** 7.20–7.15 (m, 3H), 6.97 (d, *J* = 7.4 Hz, 4H), 6.85 (s, 1H), 4.03–3.97 (m, 1H), 3.83–3.79 (m, 2H), 3.79 (s, 3H), 3.74 (s, 6H), 3.59–3.47 (m, 1H), 3.41–3.27 (m, 2H), 2.21 (s, 3H), 1.84–1.63 (m, 2H), 1.56–1.43 (m, 1H), 0.88–0.75 (m, 1H). **^13^C NMR (101 MHz, CDCl_3_) δ** 174.5, 167.3, 156.8, 153.1, 140.8, 136.2, 132.9, 129.4, 129.2, 120.3, 104.3, 60.8, 56.1, 48.8, 47.0, 46.2, 28.9, 22.2, 20.7. **HRMS (ESI):** Exact mass calculated for C_23_H_29_N_3_O_5_ [M+Na]^+^: 450.2000, found: 450.2005.

***N*-(3,5-bis(trifluoromethyl)phenyl)-3-(3,4,5-trimethoxybenzamido)piperidine-1-carboxamide** (**9k’**): General procedure was followed using **1’** (95 mg, 0.68 mmol, 2.0 equiv.), **8k** (100 mg, 0.34 mmol, 1 equiv.), PhI(OAc)_2_ (238 mg, 2 equiv.), K_3_PO_4_ (157 mg, 2 equiv.), 1,2-DCE (2 mL), at 80 °C for 18 h. Column chromatography was performed using 85/15 *v/v* petroleum ether/acetone as eluent and yielded **9k’** (203 mg, 46%). Beige solid. mp—163–165 °C. **FTIR (cm^−1^):** 3312, 2937, 1627, 1579, 1523, 1499, 1408, 1337, 1233, 1126, 1084, 989, 838, 807. **^1^H NMR (400 MHz, CDCl_3_) δ** 1H NMR (400 MHz, CDCl3) δ 7.95 (s, 2H), 7.70 (s, 1H), 7.45 (s, 1H), 6.94 (s, 2H), 6.47 (d, J = 5.1 Hz, 1H), 4.02 (s, 1H), 3.85 (s, 3H), 3.84 (s, 6H), 3.71–3.50 (m, 4H), 2.06–1.88 (m, 3H), 1.82 (dd, J = 8.8, 4.3 Hz, 2H), 1.76–1.62 (m, 1H). **^13^C NMR (101 MHz, CDCl_3_) δ** 168.0, 155.3, 153.2, 141.2, 141.1, 132.3, 131.9, 131.6, 131.3, 129.0, 127.3, 124.6, 121.8, 119.1, 119.0, 115.7, 115.7, 115.6, 104.3, 60.8, 56.1, 48.9, 47.3, 45.2, 30.9, 29.3, 22.5. ^19^F NMR (376 MHz, CDCl_3_) δ -63.02. **HRMS (ESI):** Exact mass calculated for C_24_H_26_F_6_N_3_O_5_ [M + H]^+^: 550.1772, found: 550.1780.

**4-(benzo[*d*]isothiazol-3-yl)-*N*-(p-tolyl)piperazine-1-carboxamide** (**9l**): General procedure was followed using **1a** (50 mg, 0.37 mmol, 1.0 equiv.), **8l** (121 mg, 0.55 mmol, 1.5 equiv.), PhI(OAc)_2_ (238 mg, 2 equiv.), K_3_PO_4_ (157 mg, 2 equiv.), 1,2-DCE (2 mL), at 80 °C for 18 h. Column chromatography was performed using 85/15 *v/v* petroleum ether/acetone as eluent and yielded **9l** (16 mg, 12%). Yellow solid. mp—172–174 °C. **FTIR (cm^−1^):** 3354, 2842, 1636, 1592, 1517, 1481, 1412, 1370, 1235, 1018, 996, 807, 730. **^1^H NMR (400 MHz, CDCl_3_) δ** 7.84 (dt, *J* = 8.2, 1.0 Hz, 1H), 7.76 (dd, *J* = 8.1, 1.0 Hz, 1H), 7.42 (ddd, *J* = 8.2, 6.9, 1.1 Hz, 1H), 7.32 (ddd, *J* = 8.0, 6.9, 1.0 Hz, 1H), 7.21–7.16 (m, 2H), 7.04 (d, *J* = 8.3 Hz, 2H), 6.33 (s, 1H), 3.70–3.62 (m, 4H), 3.57–3.49 (m, 4H), 2.23 (s, 3H). **^13^C NMR (101 MHz, CDCl_3_) δ** 163.4, 155.3, 152.8, 136.1, 132.9, 129.4, 127.8, 127.7, 124.1, 123.6, 120.6, 120.3, 49.7, 43.8, 20.7. **HRMS (ESI):** Exact mass calculated for C_19_H_20_N_4_OS [M+Na]^+^: 375.1251, found: 375.1255.

**4-(benzo[*d*]isothiazol-3-yl)-*N*-(3,5-bis(trifluoromethyl)phenyl)piperazine-1-carboxamide** (**9l′**): General procedure was followed using **1’** (95 mg, 0.37 mmol, 1.0 equiv.), **8l** (121 mg, 0.55 mmol, 1.5 equiv.), PhI(OAc)_2_ (238 mg, 2 equiv.), K_3_PO_4_ (157 mg, 2 equiv.), 1,2-DCE (2 mL), at 80 °C for 18 h. Column chromatography was performed using 85/15 *v/v* petroleum ether/acetone as eluent and yielded **9l′** (63 mg, 36%). Yellow sticky solid. **FTIR (cm^−1^):** 3309, 1654, 1561, 1505, 1472, 1439, 1357, 1273, 1167, 1116, 1000, 880, 729, 700, 678. **^1^H NMR (400 MHz, CDCl_3_) δ** 7.99 (d, J = 8.3 Hz, 1H), 7.97 (s, 2H), 7.80 (d, J = 8.2 Hz, 1H), 7.51–7.46 (m, 2H), 7.38–7.33 (m, 1H), 7.24 (s, 1H), 5.27 (s, 2H), 4.14 (dd, J = 7.2, 6.0 Hz, 2H), 3.86 (t, J = 6.6 Hz, 2H). **^13^C NMR (101 MHz, CDCl_3_) δ** 159.2, 153.2, 152.5, 140.3, 132.5, 132.2, 131.9, 131.5, 128.0, 126.9, 124.5, 124.4, 123.5, 121.8, 120.5, 119.3, 119.3, 116.3, 116.3, 116.3, 64.1, 48.4, 44.0, 30.9. ^19^F NMR (376 MHz, CDCl_3_) δ -63.08. **HRMS (ESI):** Exact mass calculated for C_20_H_17_F_6_N_4_OS [M + H]^+^: 475.1022, found: 475.1024.

**4-((5,6-dimethoxy-1-oxo-2,3-dihydro-1*H*-inden-2-yl)methyl)-*N*-(*p*-tolyl)piperidine-1-carboxamide** (**9m**): General procedure was followed using **1a** (50 mg, 0.37 mmol, 1.0 equiv.), **8m** (214 mg, 2 equiv.), PhI(OAc)_2_ (238 mg, 2 equiv.), K_3_PO_4_ (157 mg, 2 equiv.), 1,2-DCE (2 mL), at 80 °C for 18 h. Column chromatography was performed using 85/15 *v/v* petroleum ether/acetone as eluent and yielded **9m** (55 mg, 35%). Yellow solid. mp—160–162 °C. **FTIR (cm^−1^):** 3330, 2913, 2842, 1683, 1638, 1590, 1497, 1308, 1259, 1239, 1111, 1031, 962, 807, 723. **^1^H NMR (400 MHz, CDCl_3_) δ** 7.24 (d, *J* = 8.4 Hz, 2H), 7.16 (s, 1H), 7.07 (d, *J* = 8.3 Hz, 2H), 6.87 (s, 1H), 6.50 (s, 1H), 4.14–4.05 (m, 2H), 3.97 (s, 3H), 3.90 (s, 3H), 3.27 (dd, *J* = 17.6, 8.2 Hz, 1H), 2.93–2.82 (m, 2H), 2.71 (dt, *J* = 12.8, 3.7 Hz, 2H), 2.28 (s, 3H), 2.17 (s, 1H), 1.97–1.88 (m, 1H), 1.85–1.69 (m, 3H), 1.42–1.32 (m, 1H), 1.27 (tdt, *J* = 9.5, 4.1, 1.8 Hz, 2H). **^13^C NMR (101 MHz, CDCl_3_) δ** 207.4, 155.6, 155.2, 149.5, 148.7, 136.6, 132.4, 129.3, 129.1, 120.1, 107.3, 104.3, 56.2, 56.1, 45.1, 44.5, 38.6, 34.5, 33.4, 32.6, 31.6, 20.7. **HRMS (ESI):** Exact mass calculated for C_25_H_31_N_2_O_4_ [M + H]^+^: 423.2279, found: 423.2279.

***N*-(3,5-bis(trifluoromethyl)phenyl)-4-((5,6-dimethoxy-1-oxo-2,3-dihydro-1*H*-inden-2-yl)methyl)piperidine-1-carboxamide** (**9m’**): General procedure was followed using **1’** (50 mg, 0.37 mmol, 1.0 equiv.), **8m** (214 mg, 2 equiv.), PhI(OAc)_2_ (238 mg, 2 equiv.), K_3_PO_4_ (157 mg, 2 equiv.), 1,2-DCE (2 mL), at 80 °C for 18 h. Column chromatography was performed using 85/15 *v/v* petroleum ether/acetone as eluent and yielded **9m’** (144 mg, 72%). Colorless solid. mp—202–204 °C. **FTIR (cm^−1^):** 3300, 1684, 1638, 1539, 1497, 1472, 1439, 1375, 1304, 1276, 1169, 1121, 1068, 1024, 861, 836, 703, 680. **^1^H NMR (400 MHz, CDCl_3_) δ** 7.92 (s, 2H), 7.74 (s, 1H), 7.43 (s, 1H), 7.13 (s, 1H), 6.88 (s, 1H), 4.25–4.15 (m, 2H), 3.97 (s, 3H), 3.88 (s, 3H), 3.28 (dd, J = 17.6, 8.0 Hz, 1H), 2.91 (td, J = 13.3, 12.7, 2.4 Hz, 2H), 2.77–2.67 (m, 2H), 1.95–1.72 (m, 4H), 1.43–1.16 (m, 3H). **^13^C NMR (101 MHz, CDCl_3_) δ** 207.8, 155.8, 154.4, 149.6, 149.0, 141.3, 132.2, 131.9, 131.6, 131.2, 128.9, 124.6, 121.9, 119.4, 119.3, 115.5, 107.4, 104.2, 56.2, 56.0, 45.0, 44.5, 44.5, 38.5, 34.3, 33.3, 32.6, 31.6. **^19^F NMR (376 MHz, CDCl_3_) δ** −62.99. **HRMS (ESI):** Exact mass calculated for C_26_H_27_F_6_N_2_O_4_ [M + H]^+^: 545.1870, found: 545.1873.

## 4. Conclusions

In summary, we have developed a highly efficient method for synthesizing unsymmetrical ureas from benzamides and primary and secondary amines via a hypervalent iodine-mediated Hofmann-type rearrangement. This method exhibits broad functional group tolerance on both coupling partners and demonstrates compatibility with water, making it a valuable tool for generating a wide variety of unsymmetrical ureas. Additionally, this protocol can be effectively applied to the late-stage modification of various important drug structures, underscoring its potential utility in drug development.

## Data Availability

The data associated with this article can be obtained from Supplementary Materials.

## Supplementary Materials

The following supporting information can be downloaded at: https://www.mdpi.com/article/10.3390/molecules29235669/s1, Optimization Table S1: NMR spectra (^1^H NMR, ^13^C NMR, & ^19^F NMR), LC-MS study of crude reaction mixture revealing acetic acid release. Table S2: Optimization of base.
