# Supplementary material for: Synthesis of Unsymmetrical Urea Derivatives via PhI(OAc)2 and Application in Late-Stage Drug Functionalization
Source: Molecules. 2024 Nov 29;29(23):5669. doi: 10.3390/molecules29235669 (PMC11643609; doi:10.3390/molecules29235669)
Supplement: Supplementary file 1 [file molecules-29-05669-s001.zip › molecules-3337595-supplementary.pdf]

Table of contents

1. Optimization table 2
2. NMR spectra 5
3. LC-MS spectra 254
4. Crude NMR 255

Table SI-1: Optimization of solvent

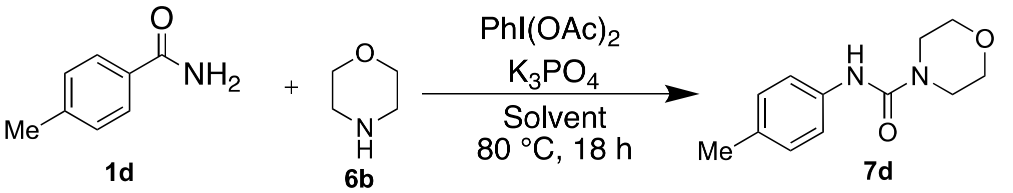

S. No.	Base	Yield (%)
1	Acetonitrile	69
2	Methanol	12
3	DCE	73

Reaction conditions: 1d (50 mg, 0.37 mmol, 1.0 equiv.), **6b** (64 mg, 0.74 mmol, 2 equiv.), $PhI(OAc)_2$ (238 mg, 2 equiv.), K_3PO_4 (157 mg, 2 equiv.), 1,2-DCE (2 mL), at 80 °C for 18 h.

Table SI-2: Optimization of base

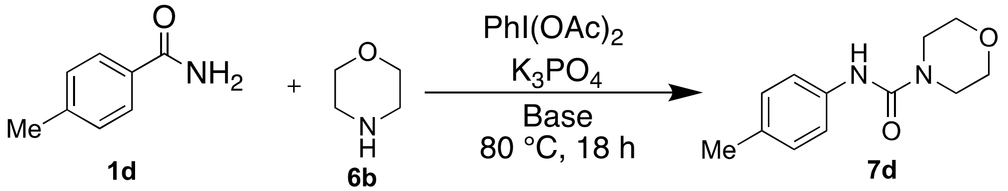

S. No.	Base	Yield (%)
1	K_3PO_4	73
2	K_2HPO_4	46
3	Cs_2CO_3	70
4	K_2CO_3	50
5	LiOAc	49
6	NaOAc	55
7	KOAc	56

Reaction conditions: **1d** (50 mg, 0.37 mmol, 1.0 equiv.), **6b** (64 mg, 0.74 mmol, 2 equiv.), $PhI(OAc)_2$ (238 mg, 2 equiv.), K_3PO_4 (157 mg, 2 equiv.), 1,2-DCE (2 mL), at 80 °C for 18 h.

NMR spectra

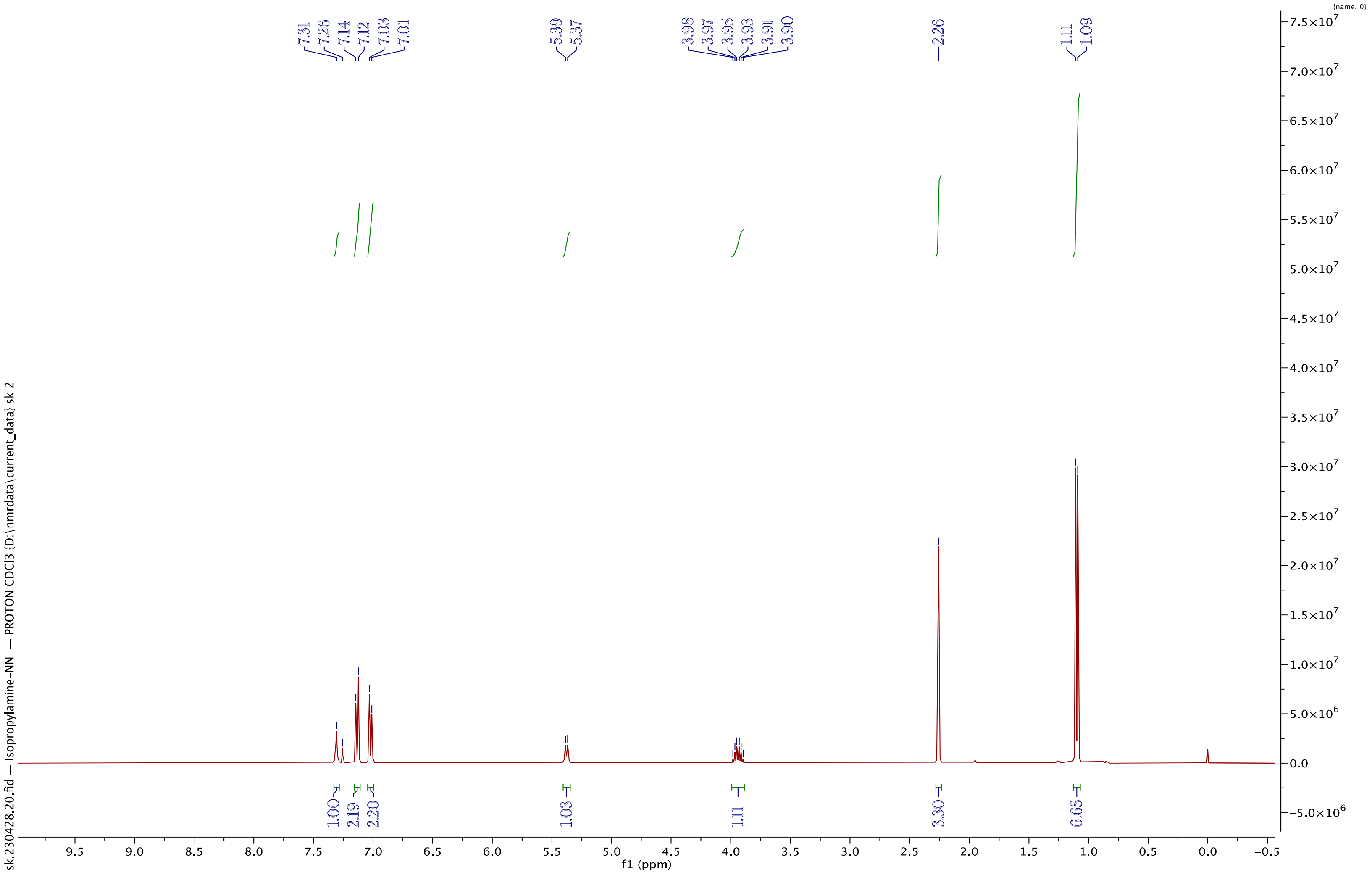

^{1}H NMR spectra of **3a** (400 MHz, RT, $CDCl_3$)

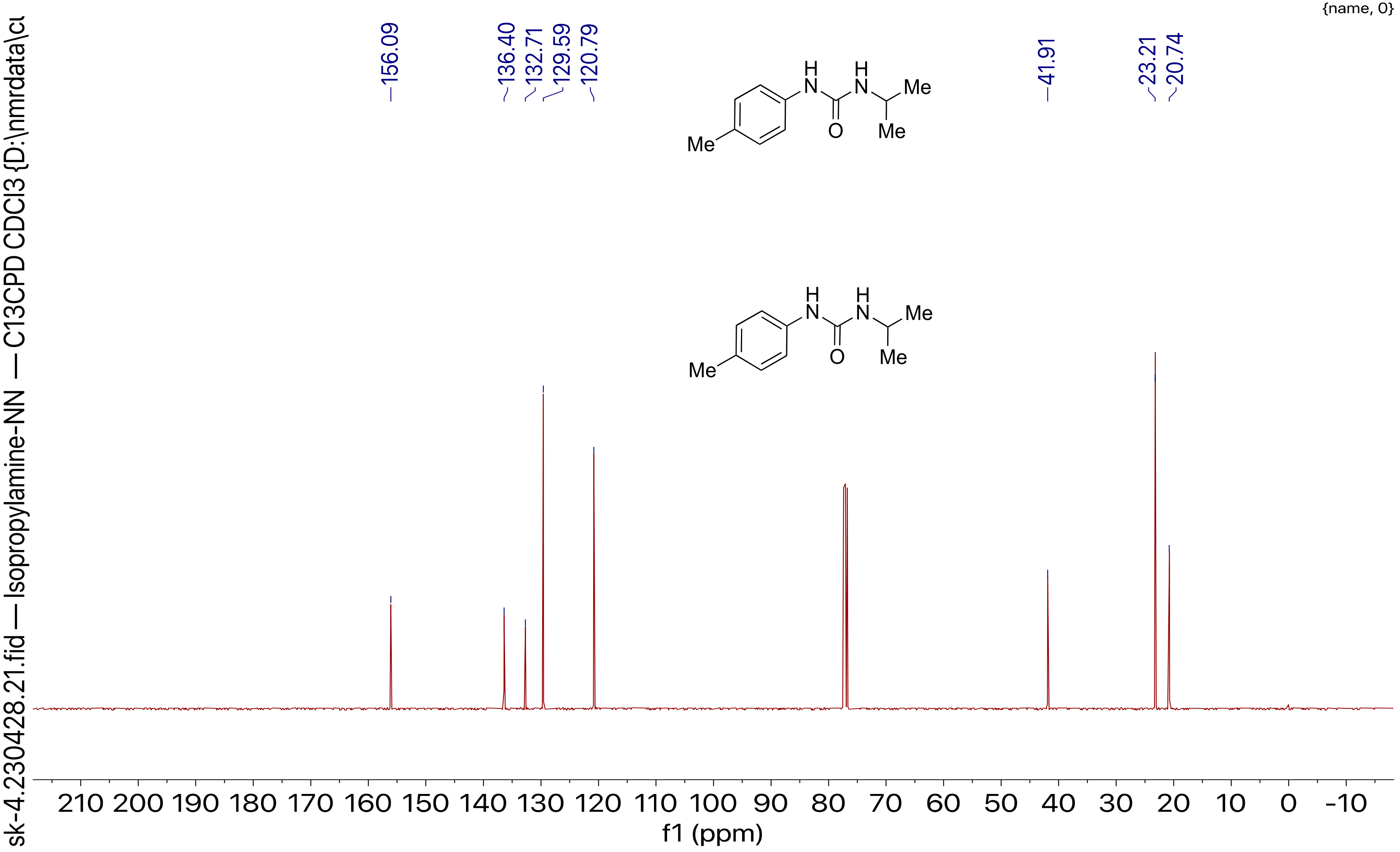

^{13}C NMR spectra of **3a** (101 MHz, RT, $CDCl_3$)

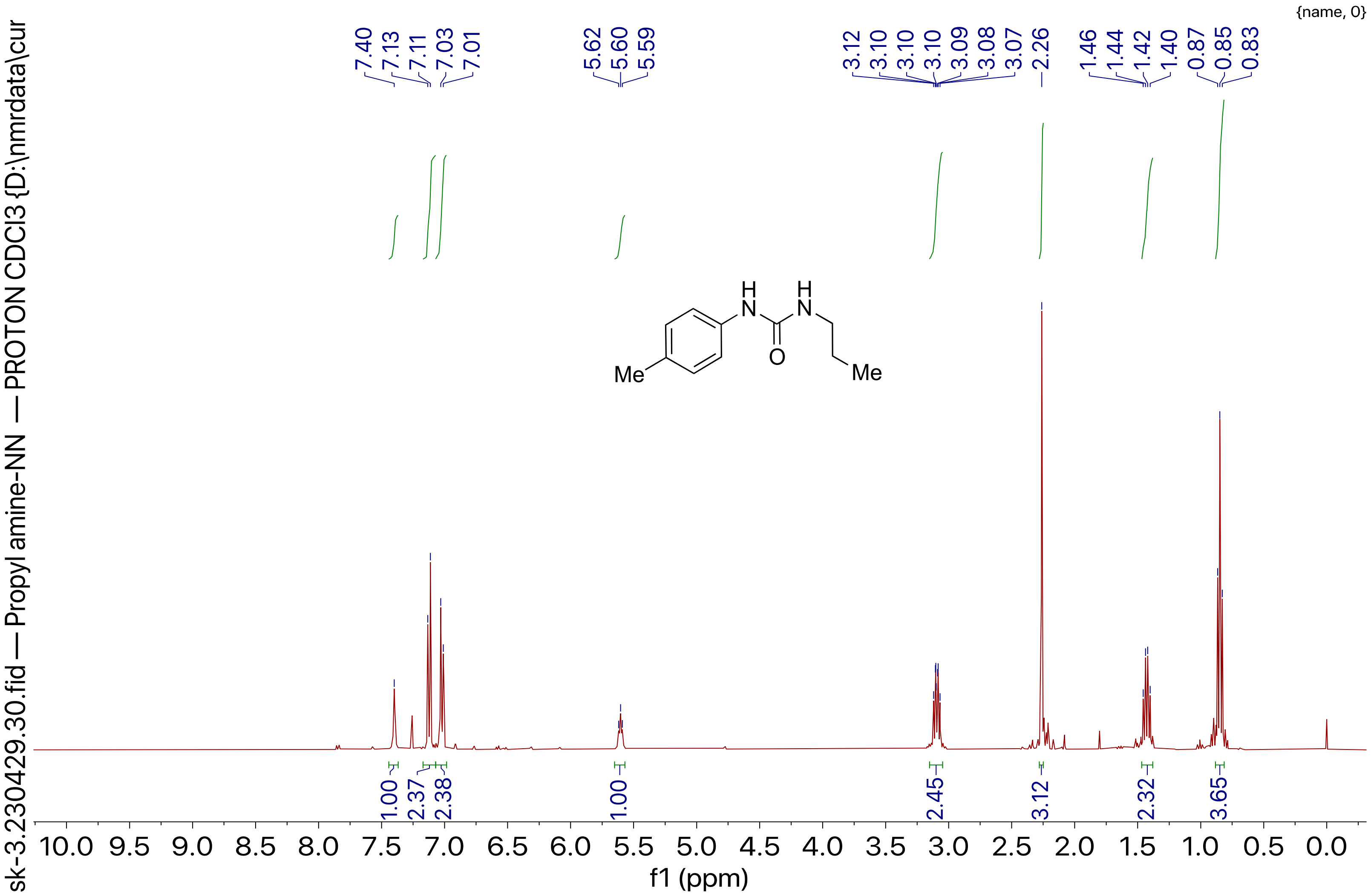

^{1}H NMR spectra of **3b** (400 MHz, RT, $CDCl_3$)

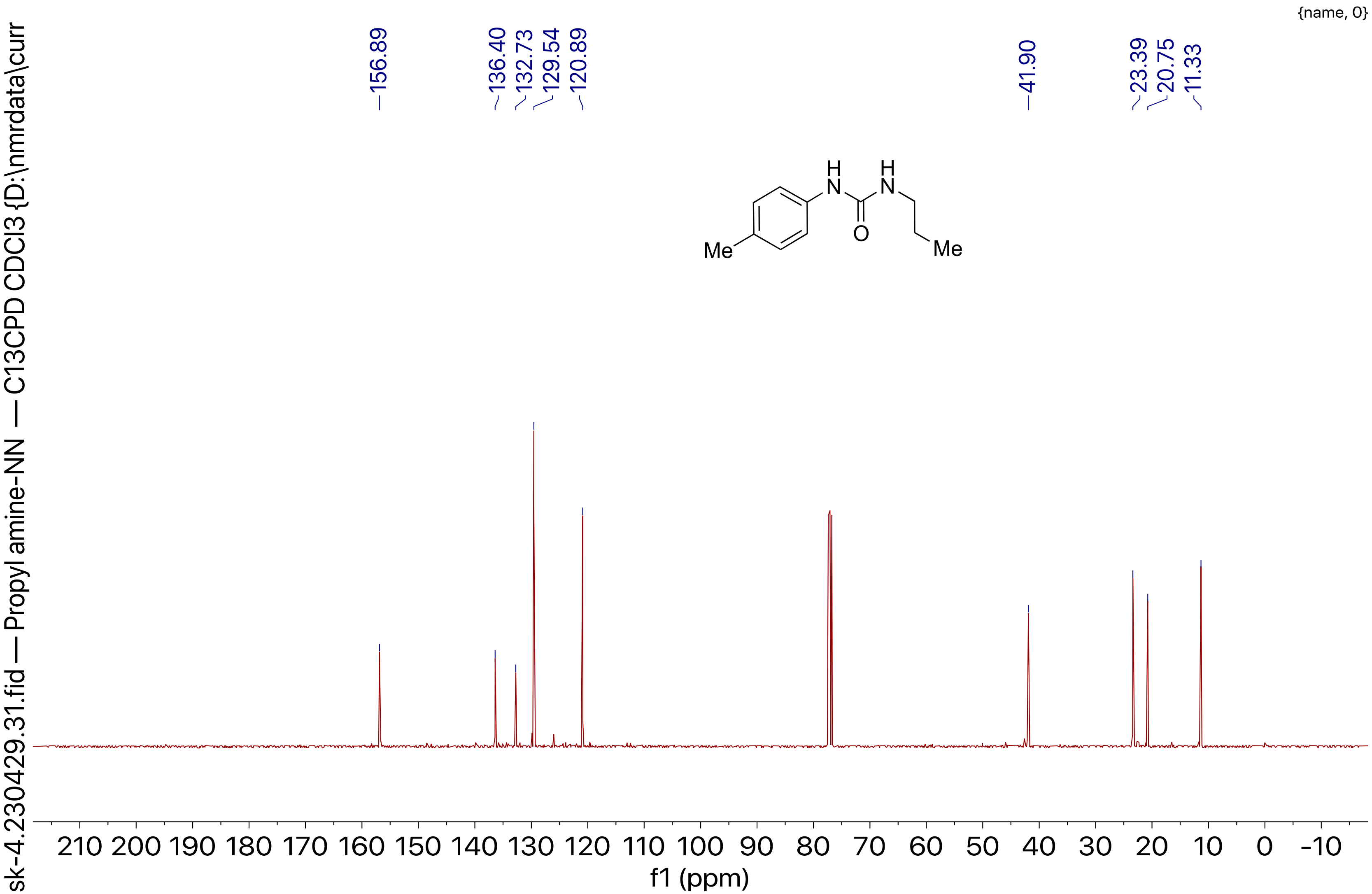

^{13}C NMR spectra of **3b** (101 MHz, RT, $CDCl_3$)

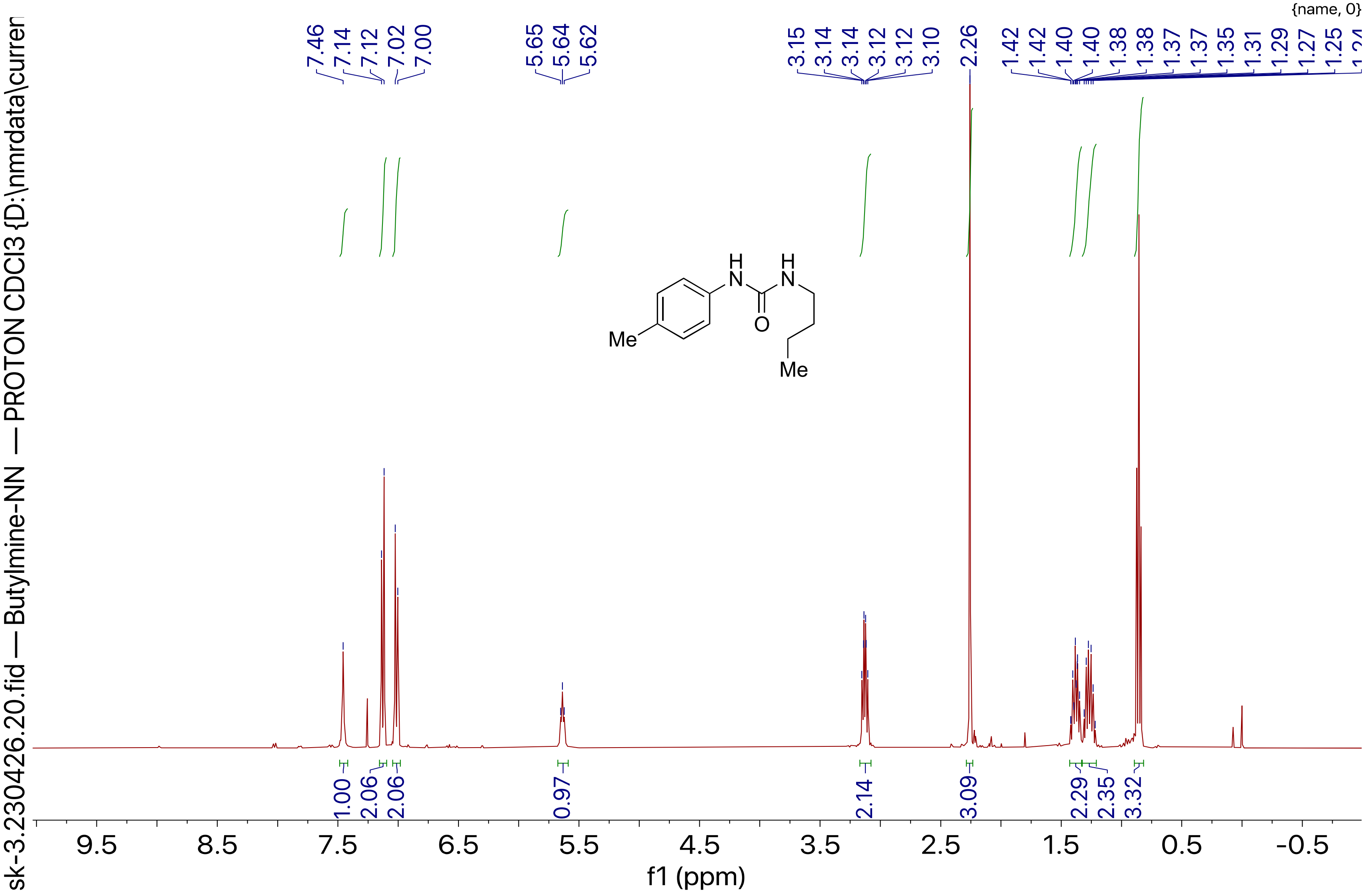

^{1}H NMR spectra of **3c** (400 MHz, RT, $CDCl_3$)

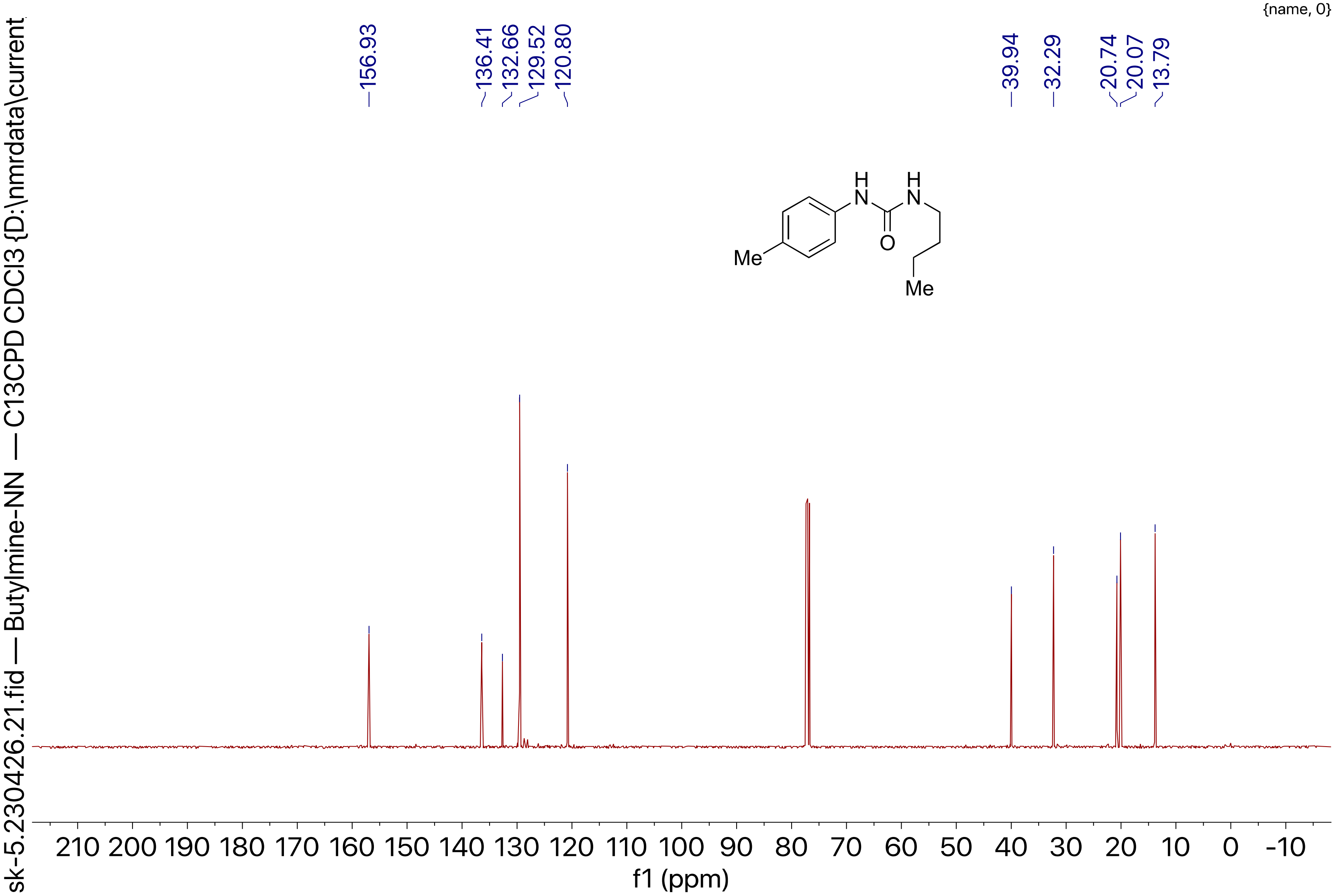

^{13}C NMR spectra of **3c** (101 MHz, RT, $CDCl_3$)

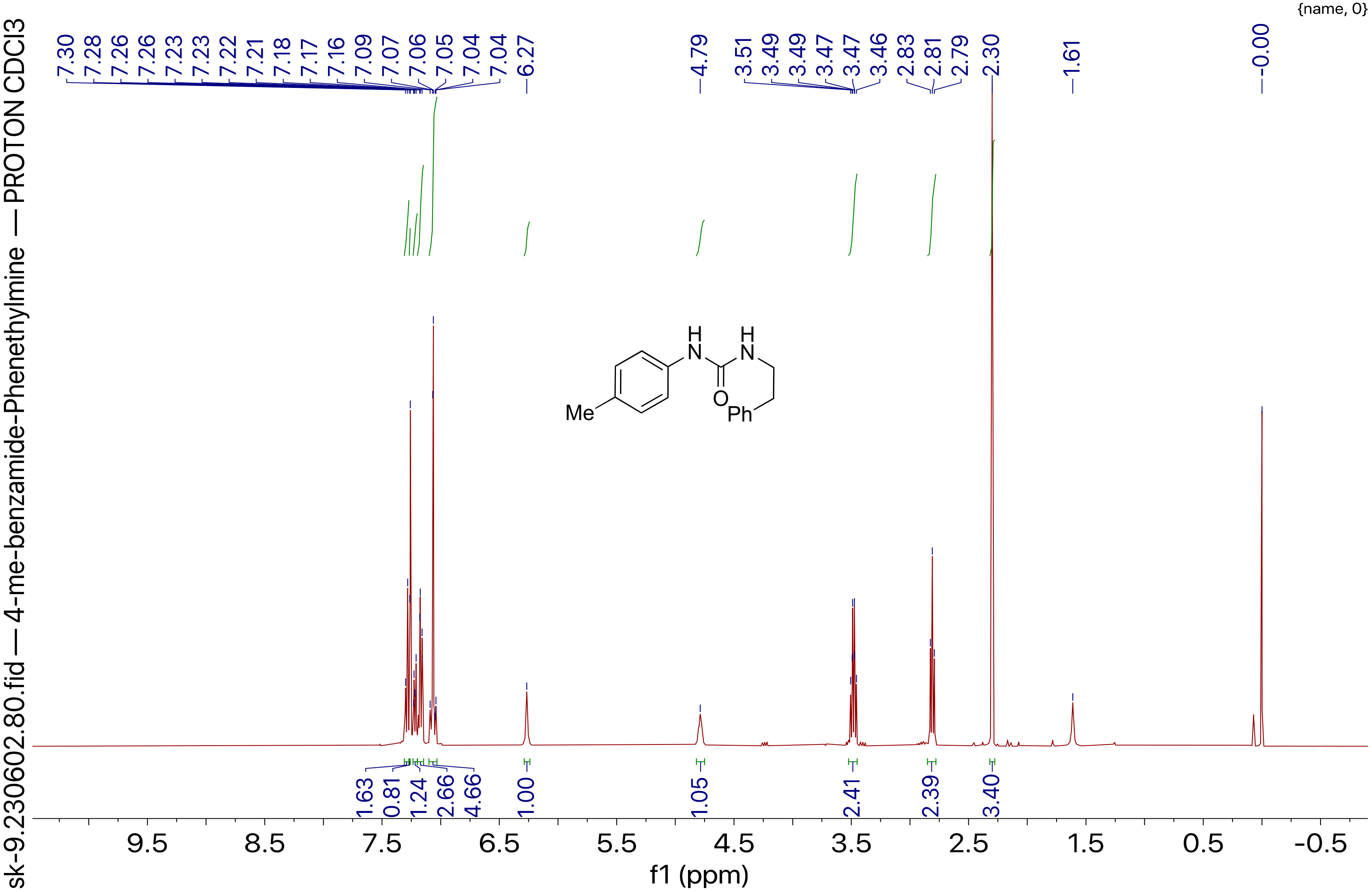

^{1}H NMR spectra of **3d** (400 MHz, RT, $CDCl_3$)

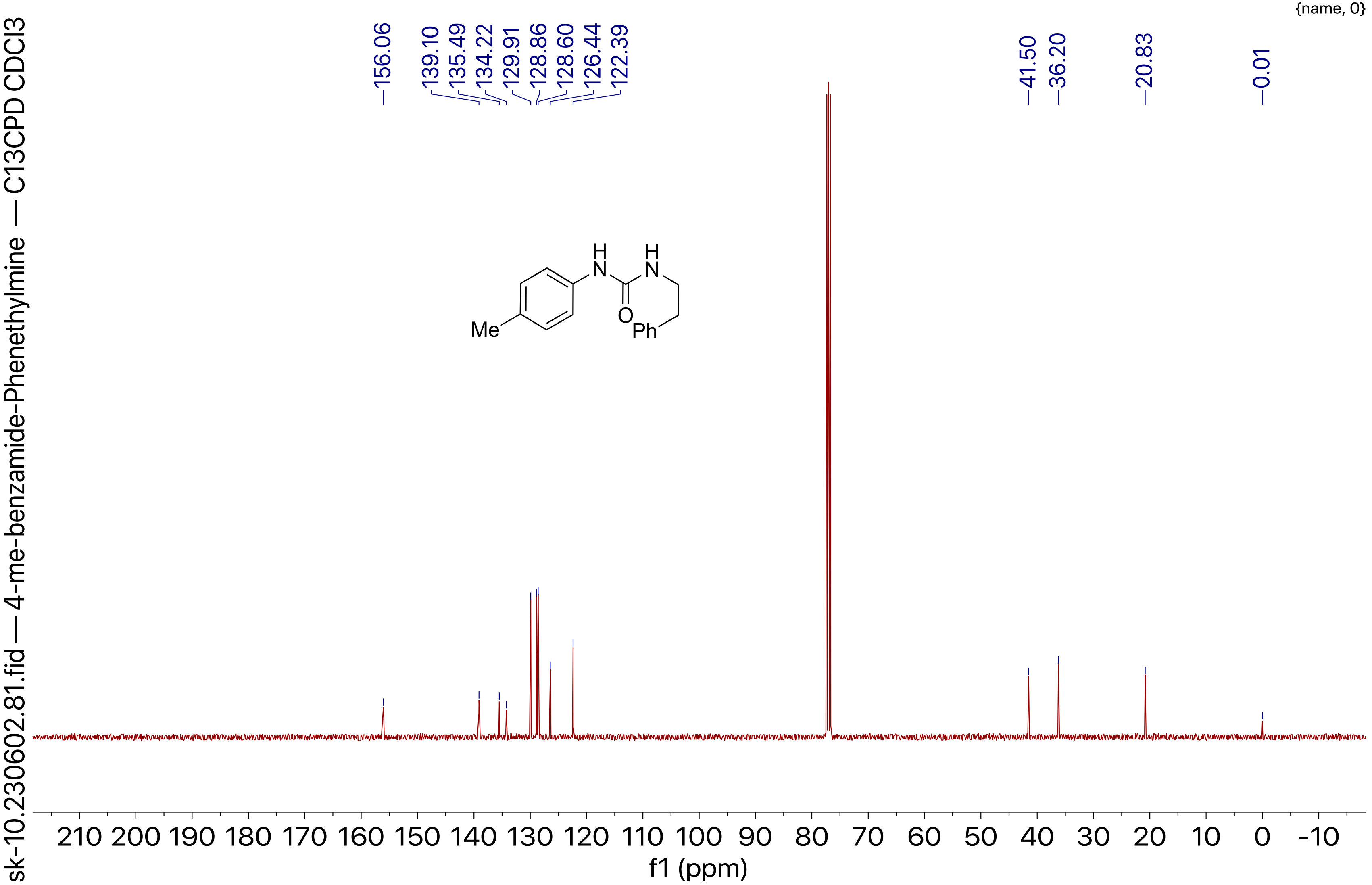

^{13}C NMR spectra of **3d** (101 MHz, RT, $CDCl_3$)

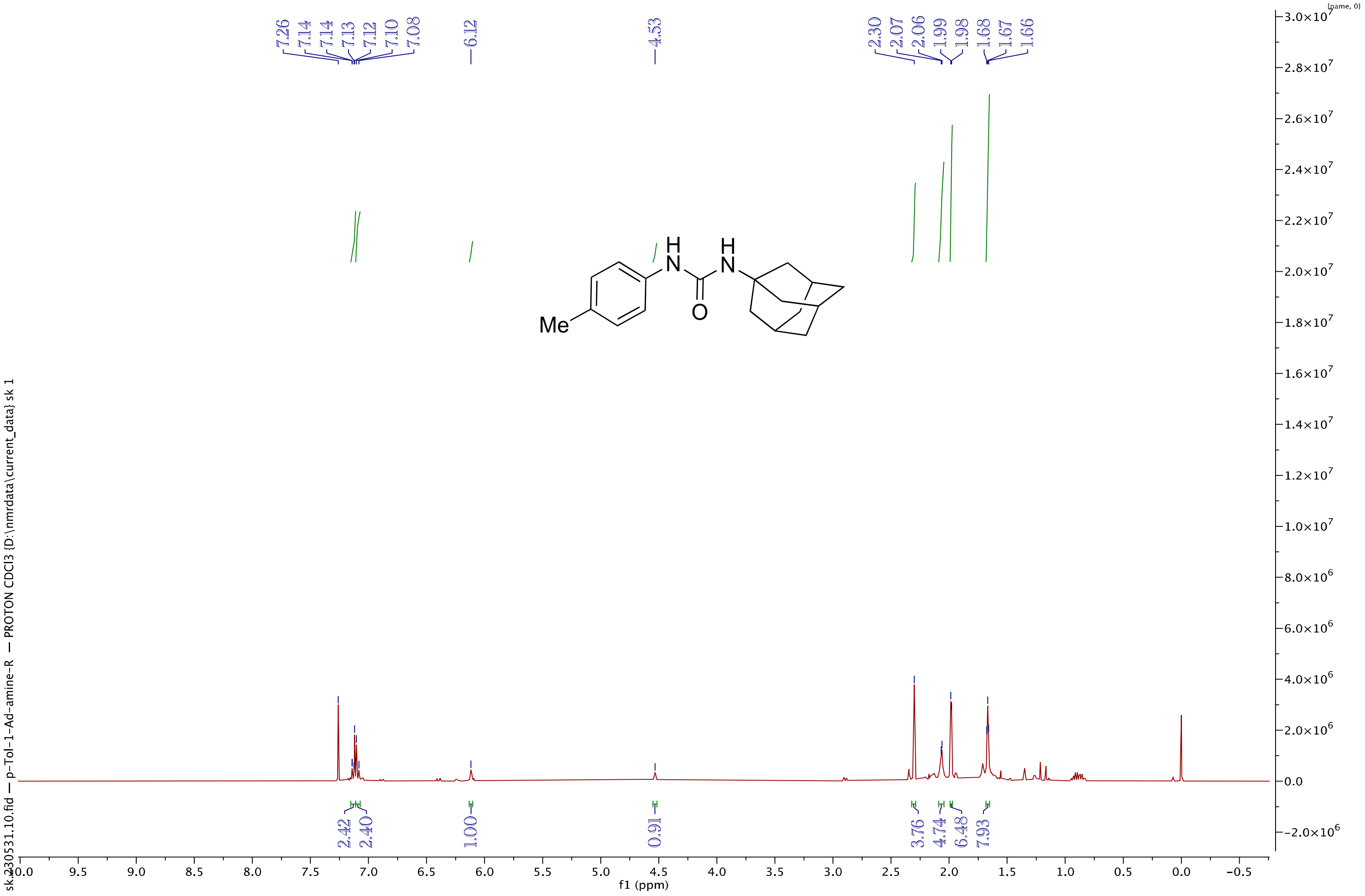

^{1}H NMR spectra of **3e** (400 MHz, RT, $CDCl_3$)

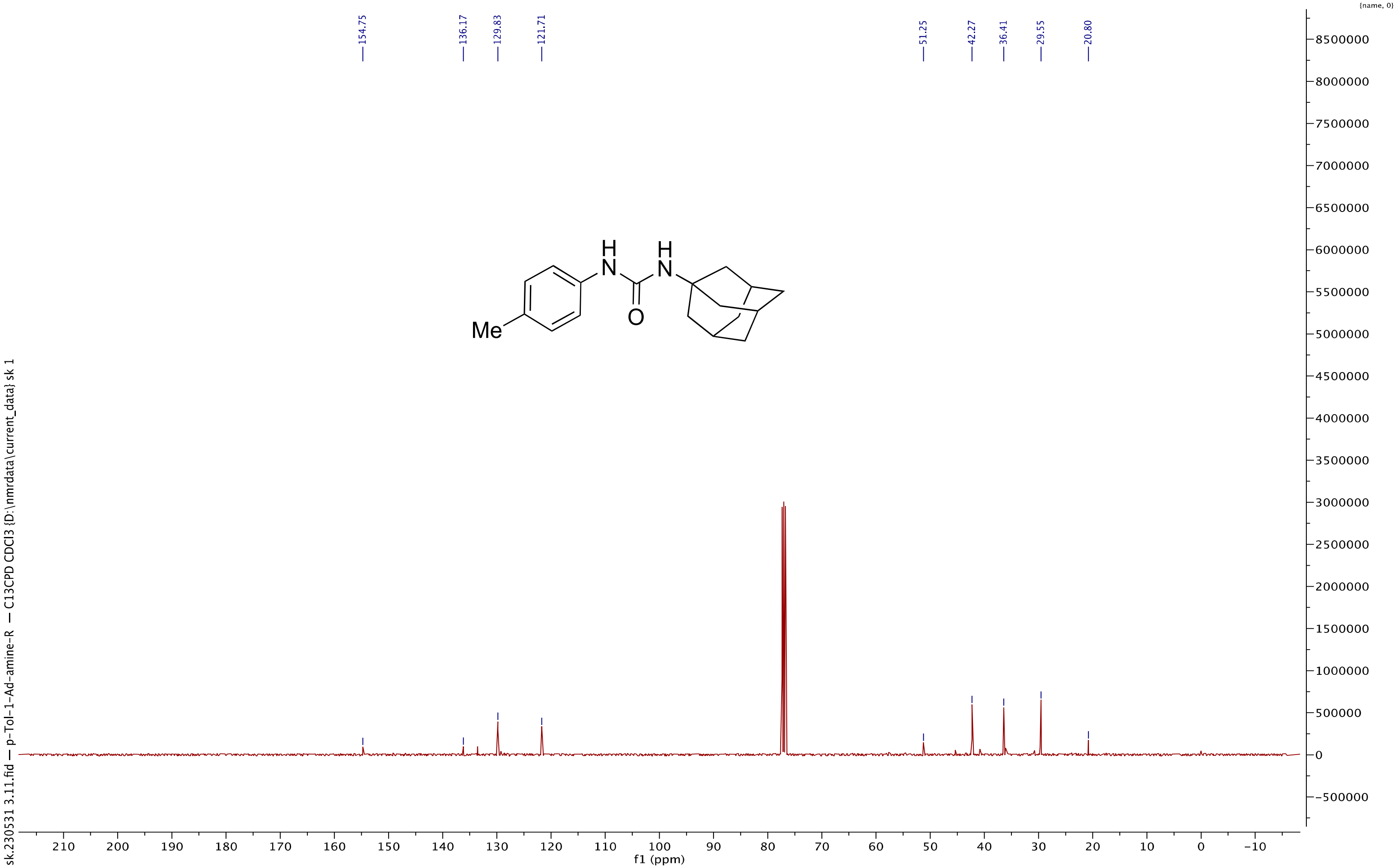

^{13}C NMR spectra of **3e** (101 MHz, RT, $CDCl_3$)

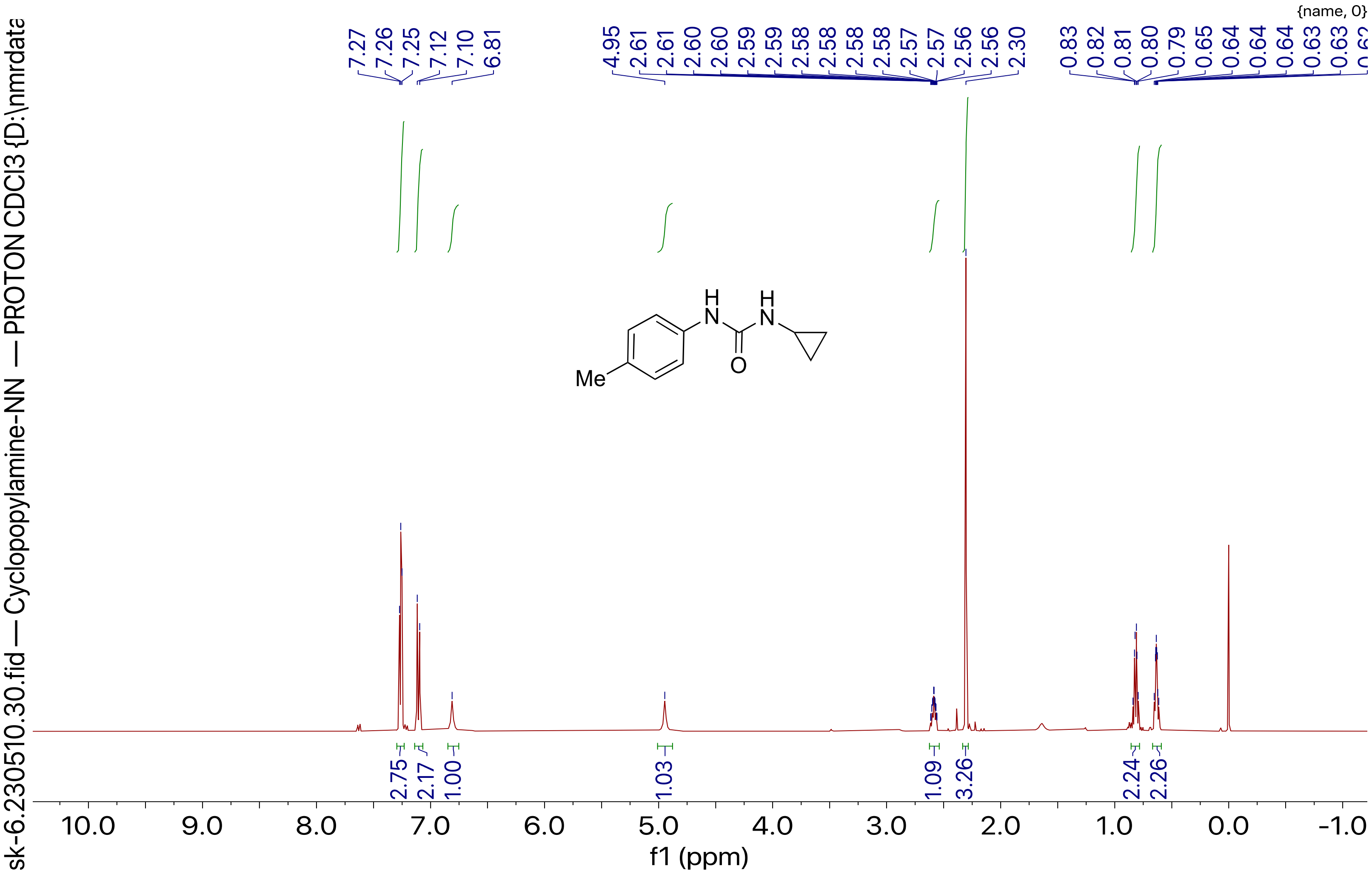

^{1}H NMR spectra of **3f** (400 MHz, RT, $CDCl_3$)

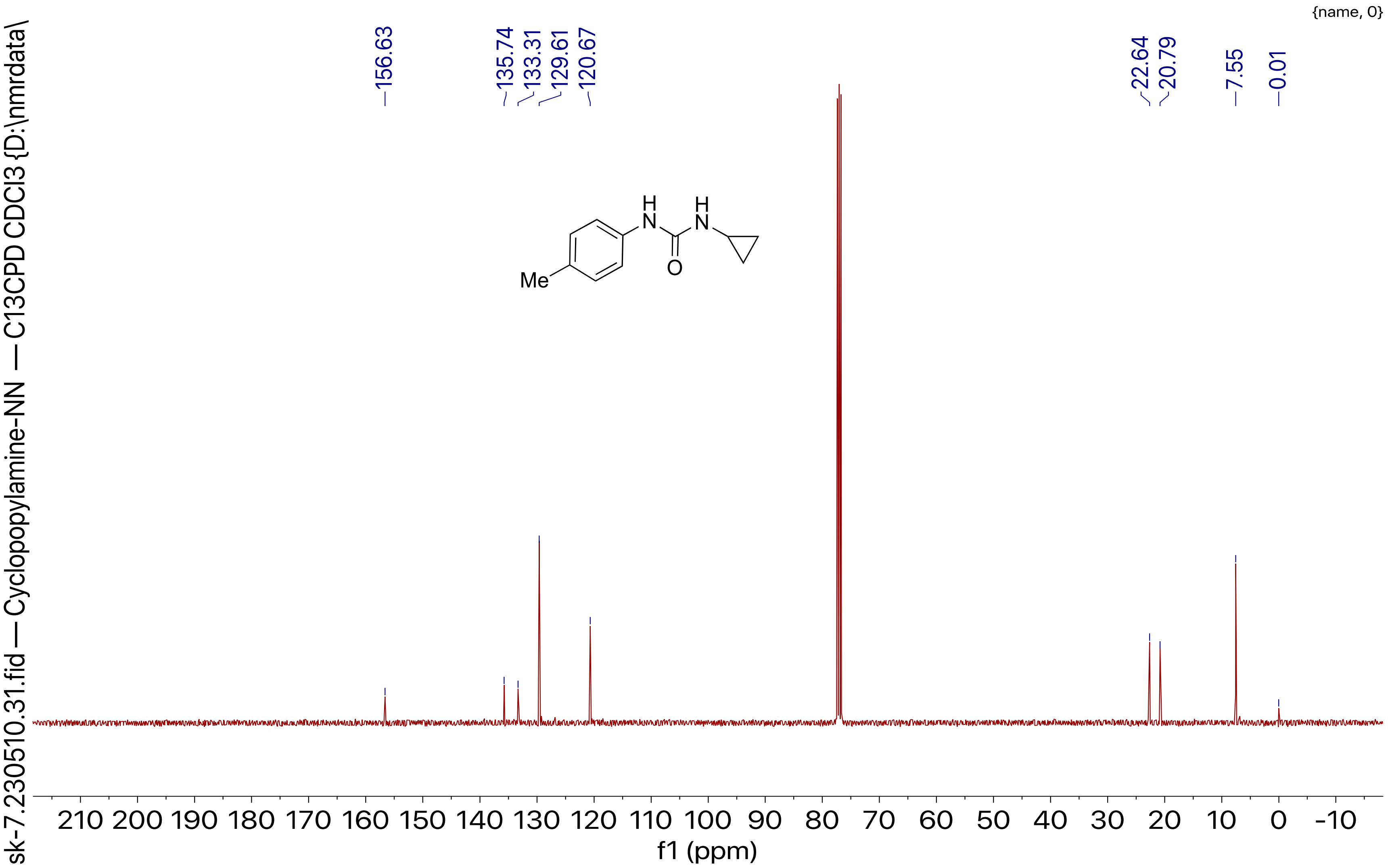

^{13}C NMR spectra of **3f** (101 MHz, RT, $CDCl_3$)

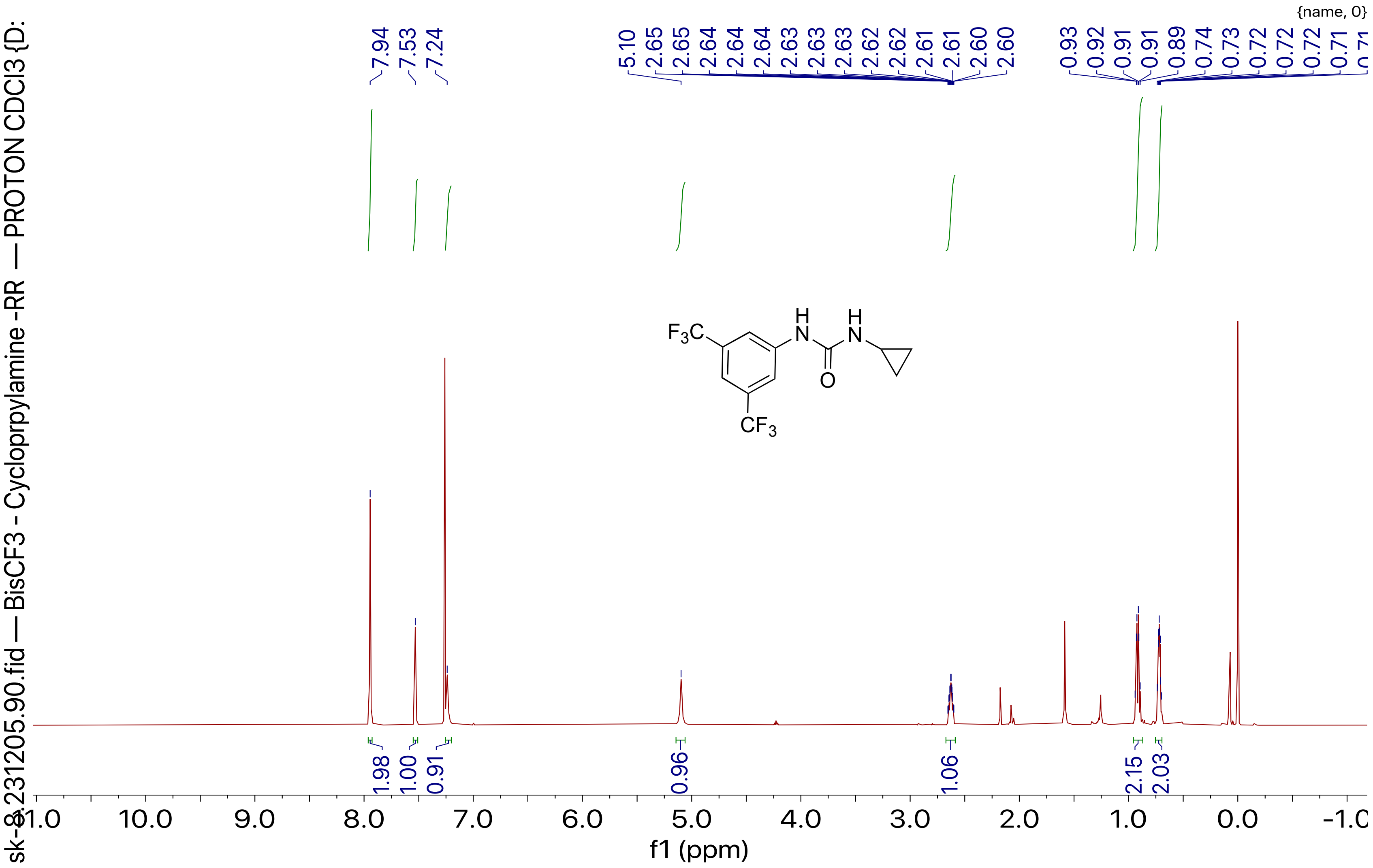

^{1}H NMR spectra of **3f'** (400 MHz, RT, $CDCl_3$)

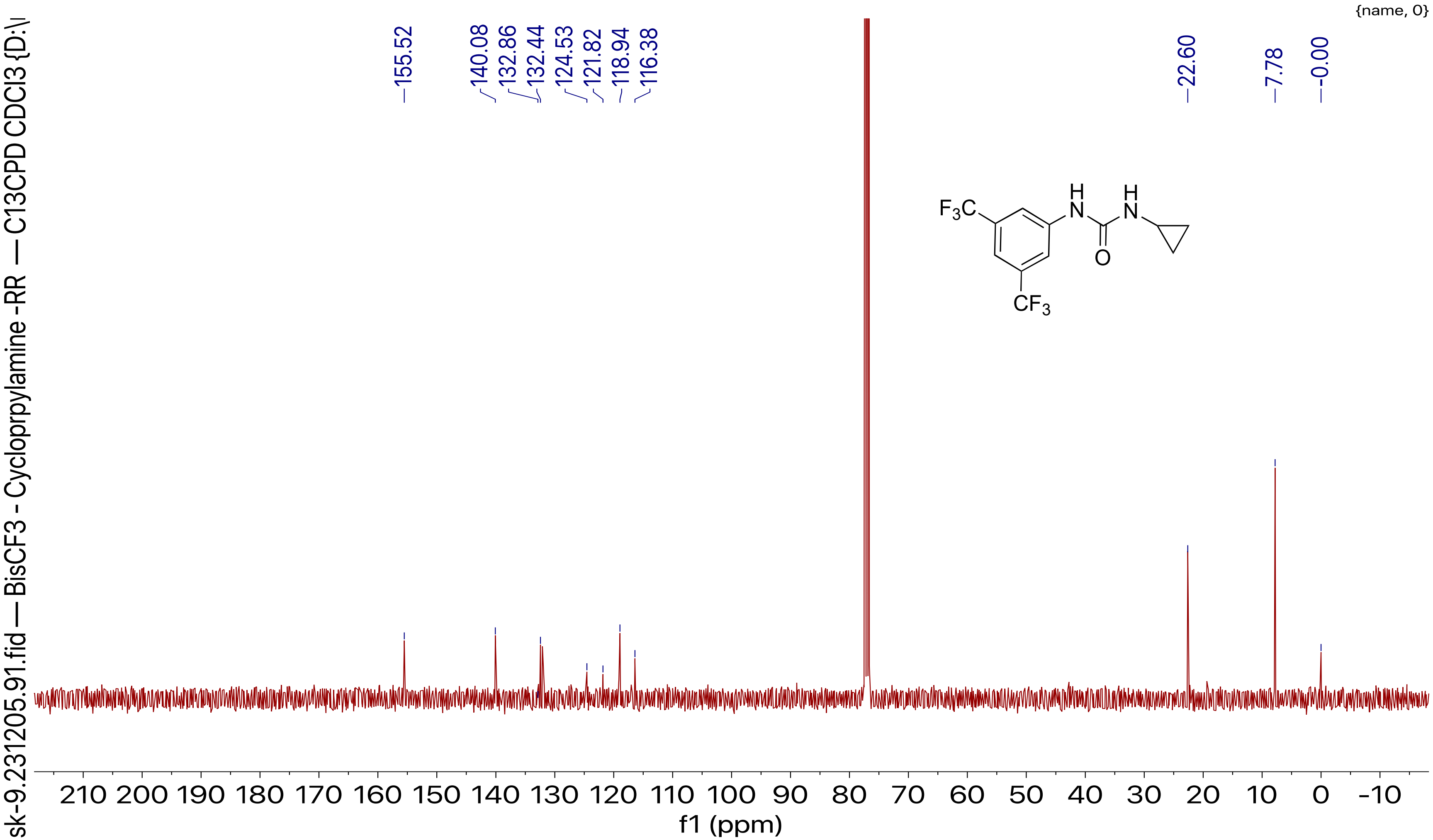

^{13}C NMR spectra of **3f’** (101 MHz, RT, $CDCl_3$)

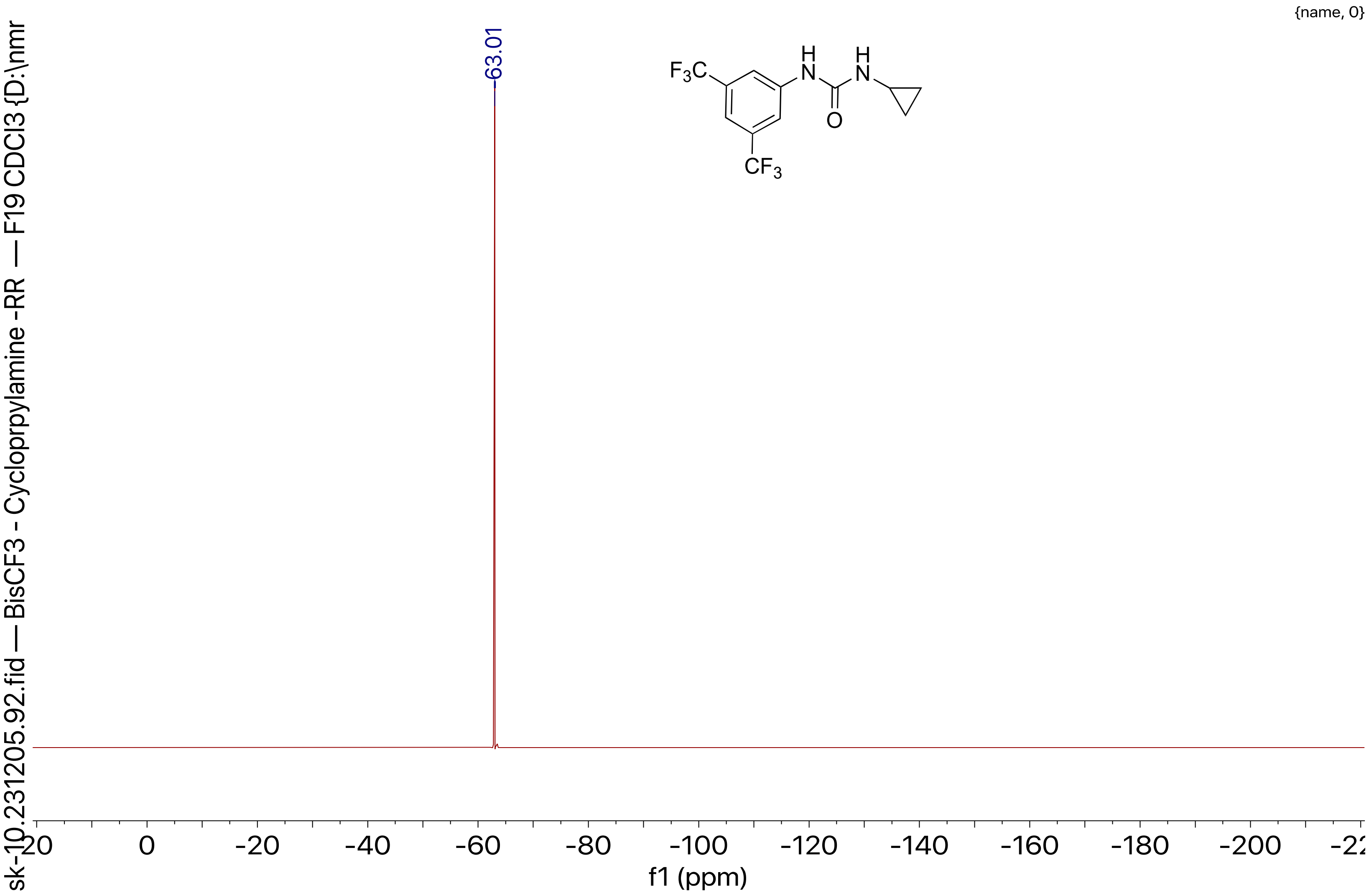

^{19}F NMR spectra of **3f’** (376 MHz, RT, $CDCl_3$)

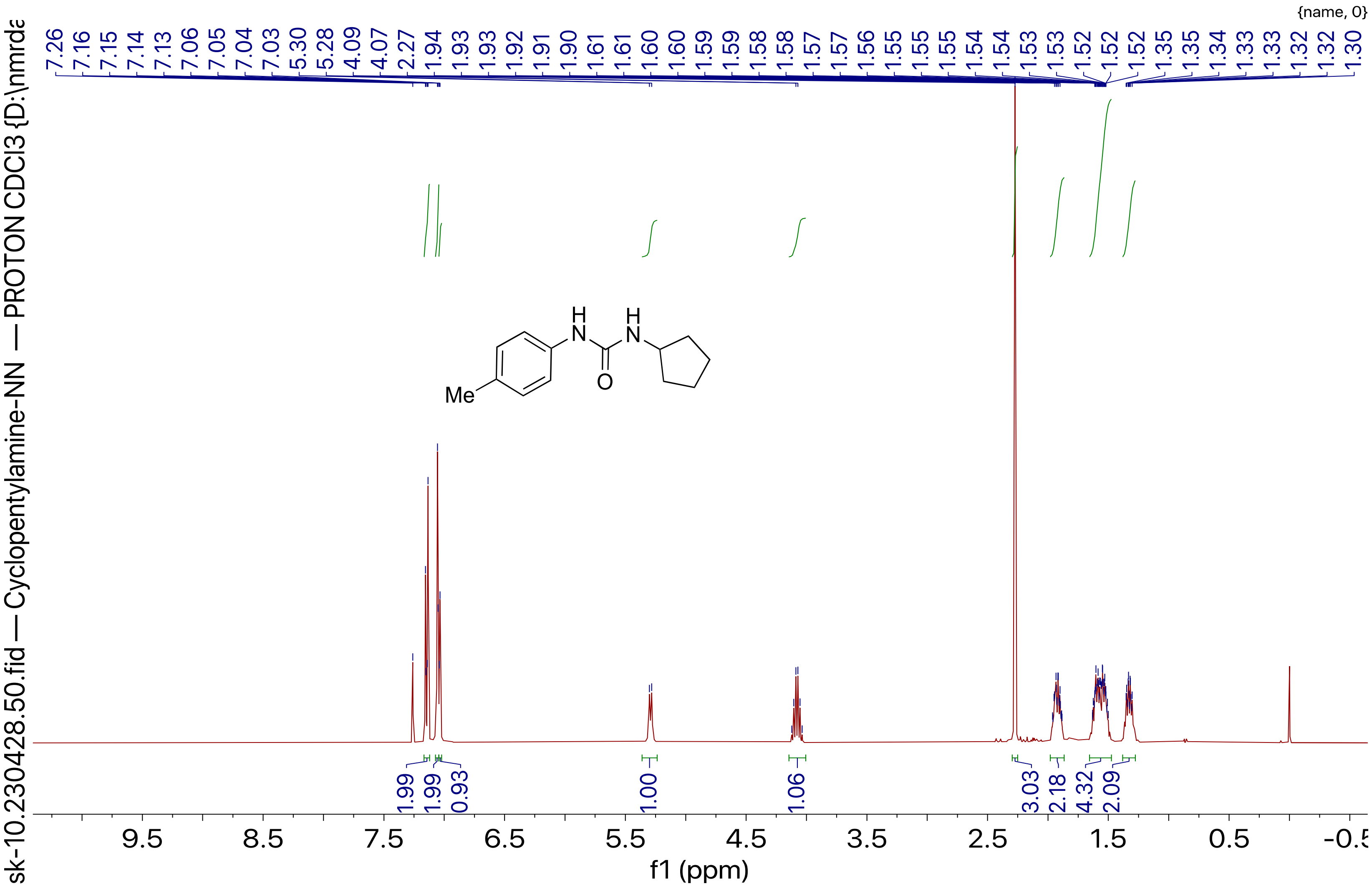

^{1}H NMR spectra of **3g** (400 MHz, RT, $CDCl_3$)

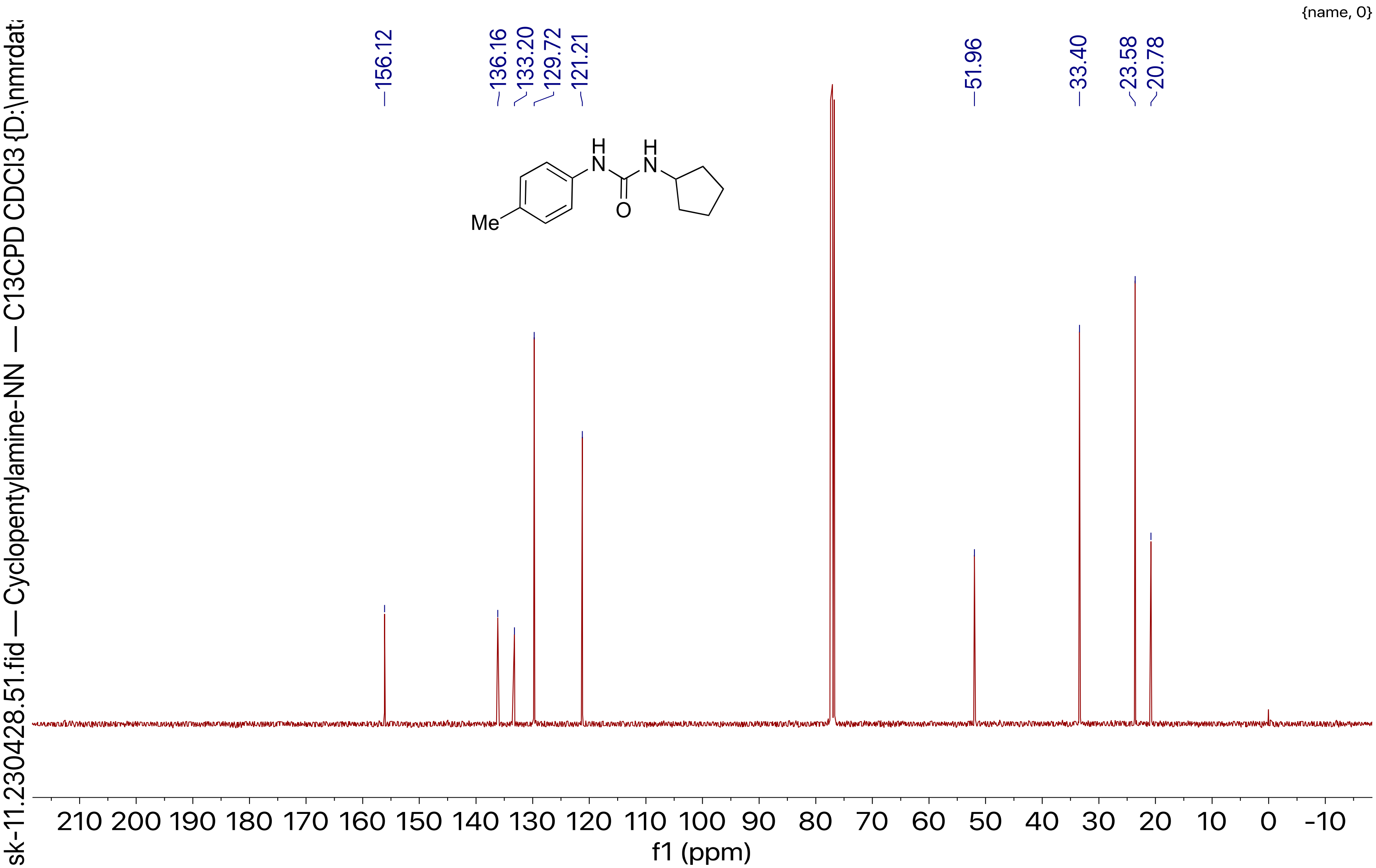

^{13}C NMR spectra of **3g** (101 MHz, RT, $CDCl_3$)

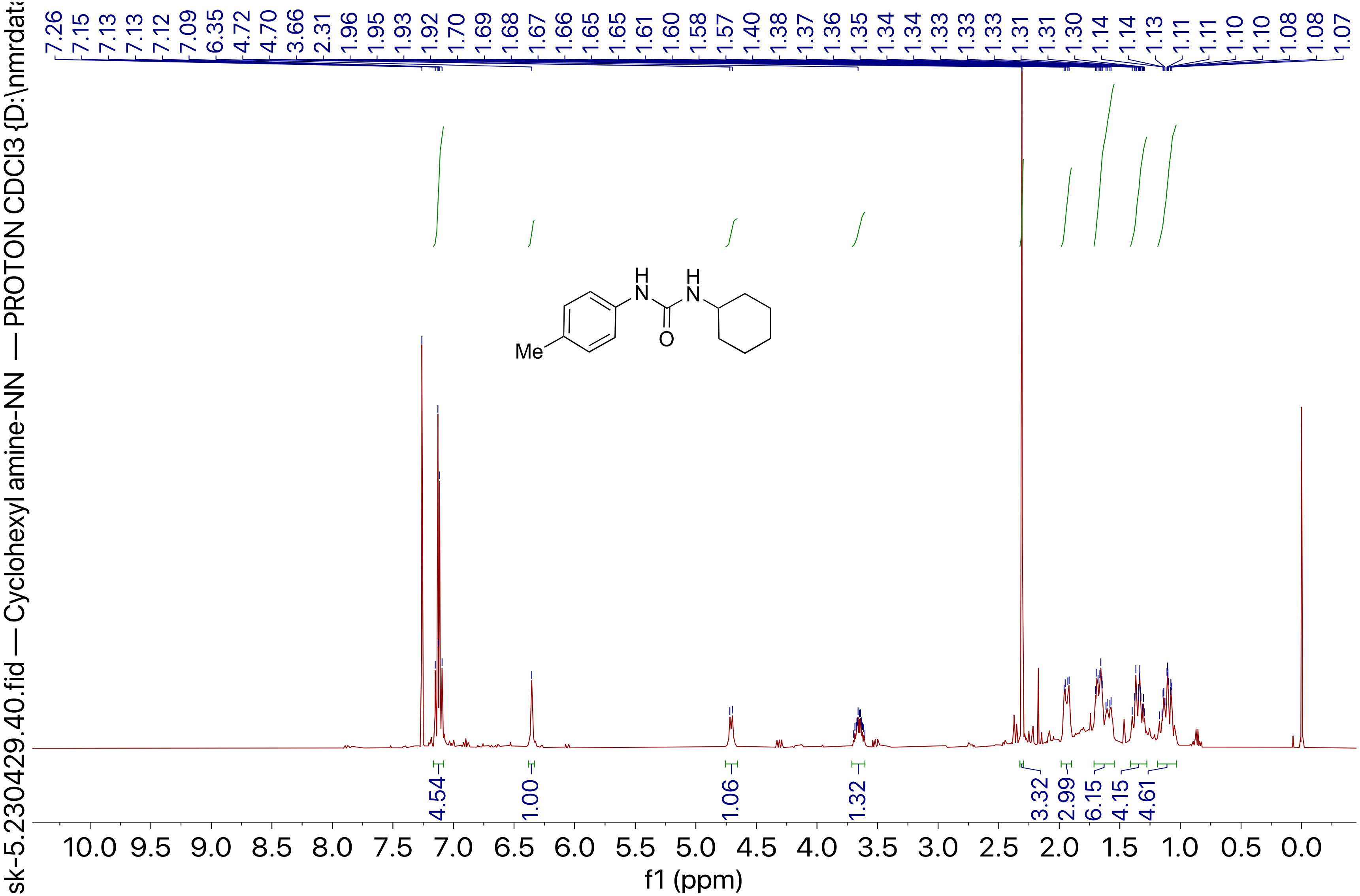

^{1}H NMR spectra of **3h** (400 MHz, RT, $CDCl_3$)

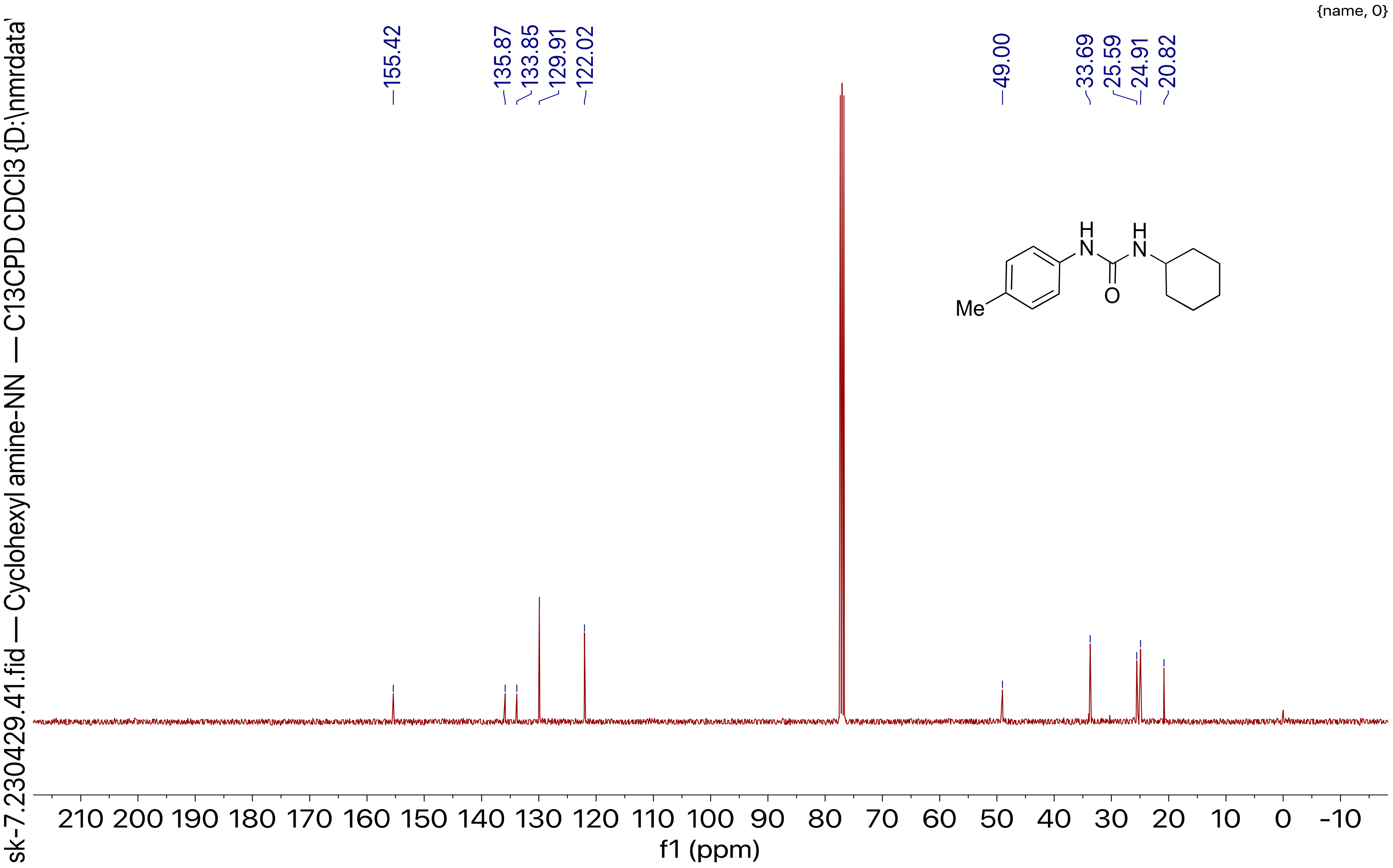

^{13}C NMR spectra of **3h** (101 MHz, RT, $CDCl_3$)

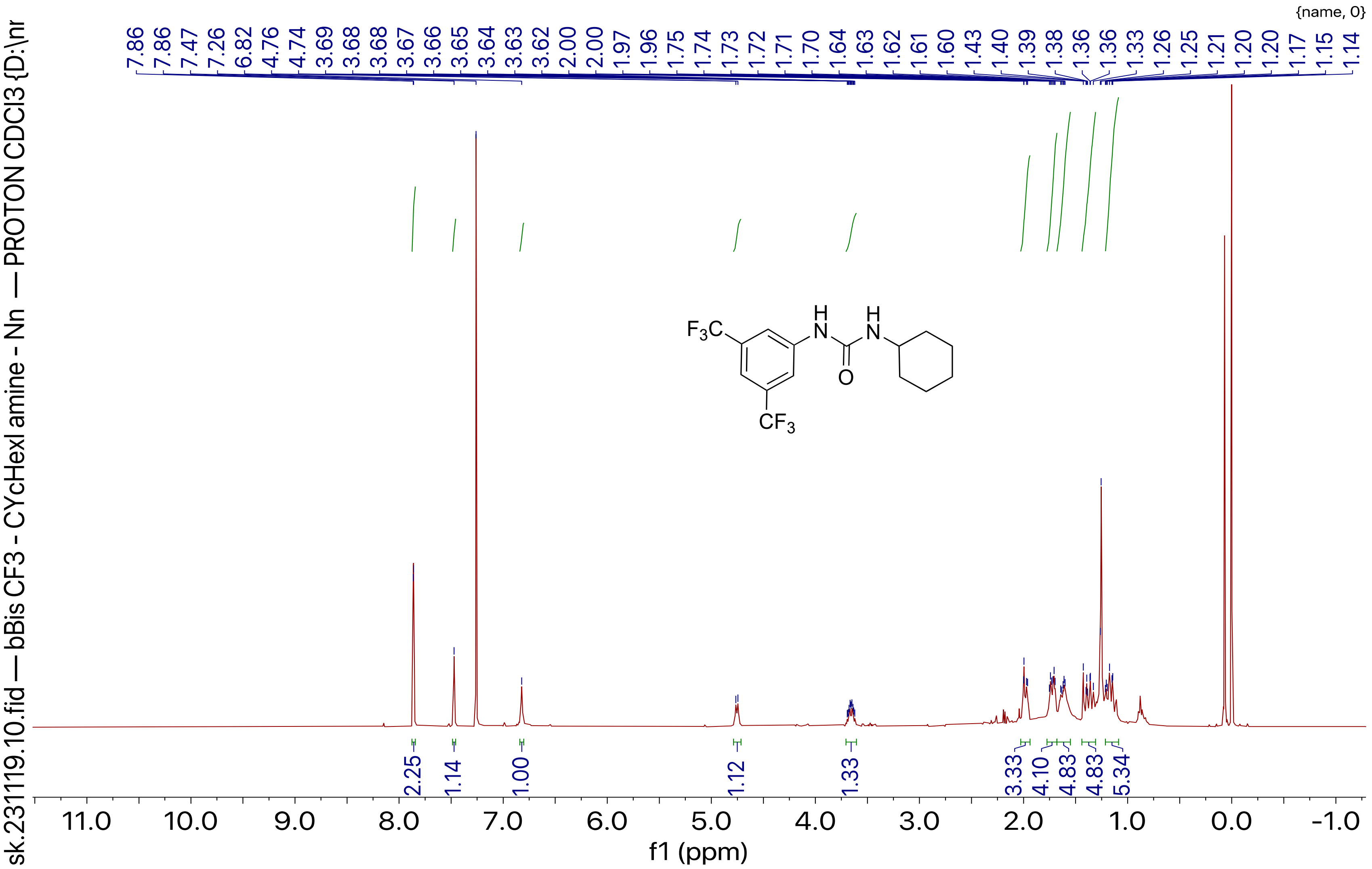

^{1}H NMR spectra of **3h'** (400 MHz, RT, $CDCl_3$)

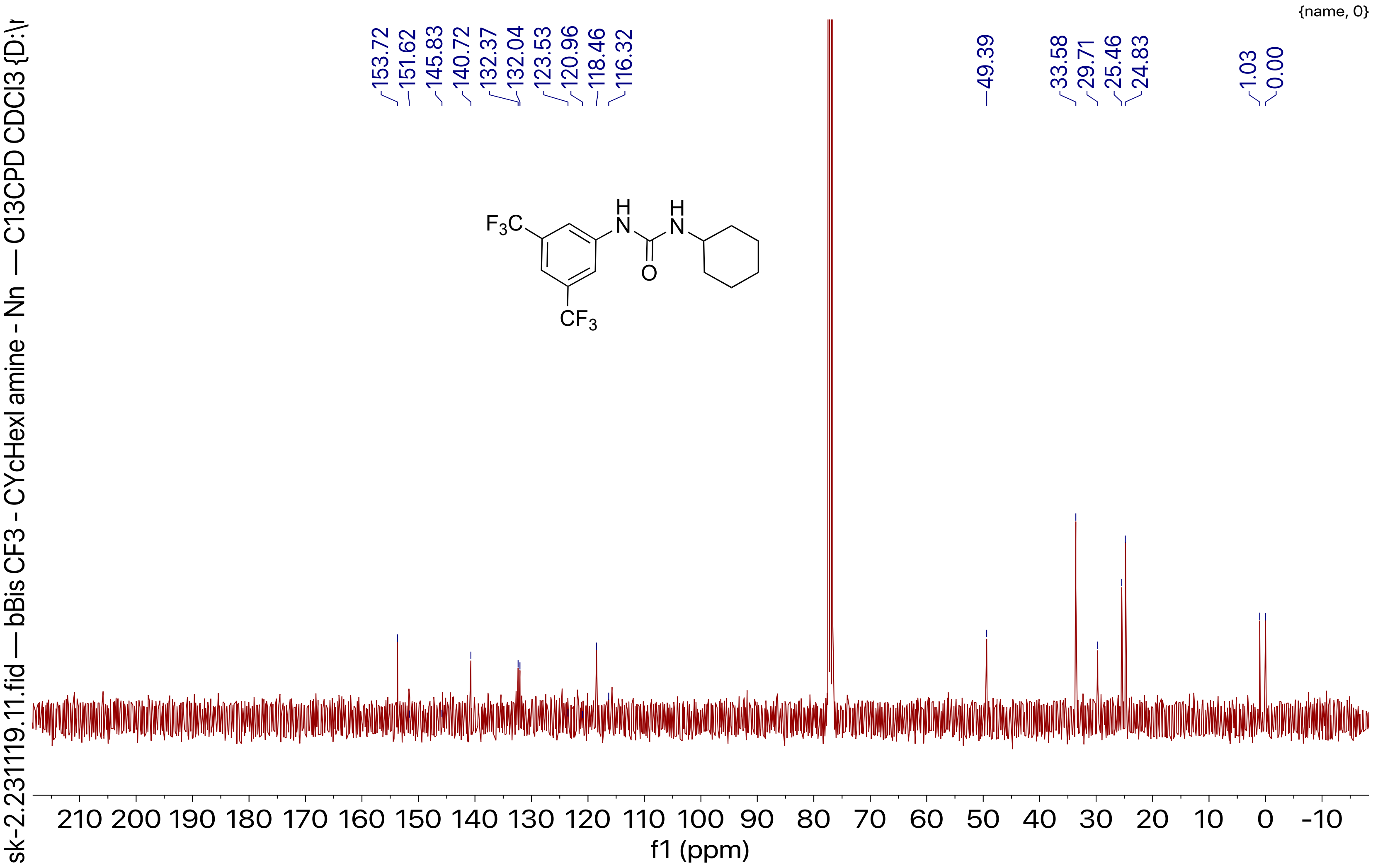

^{13}C NMR spectra of **3h'** (101 MHz, RT, $CDCl_3$)

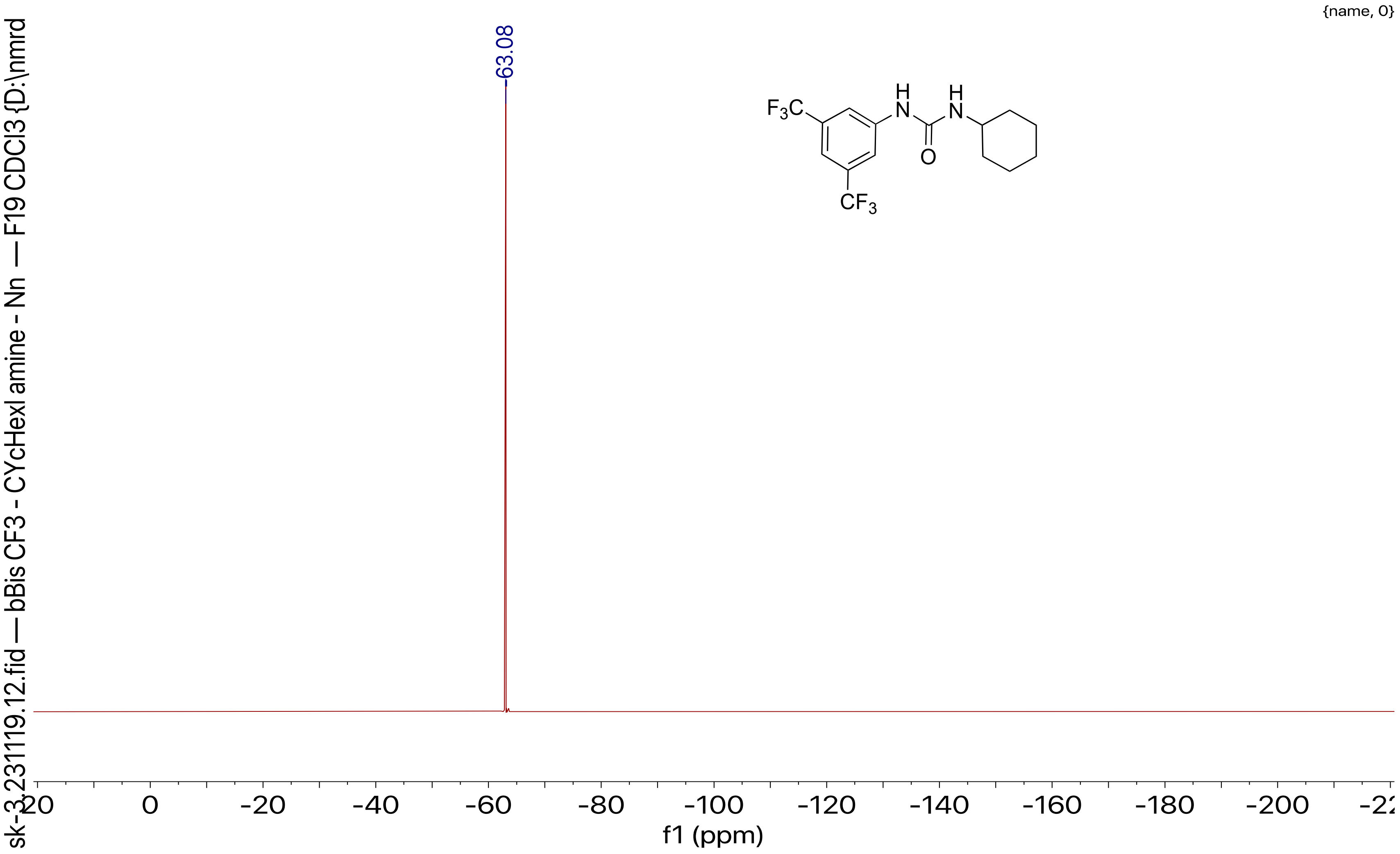

^{19}F NMR spectra of **3h’** (376 MHz, RT, $CDCl_3$)

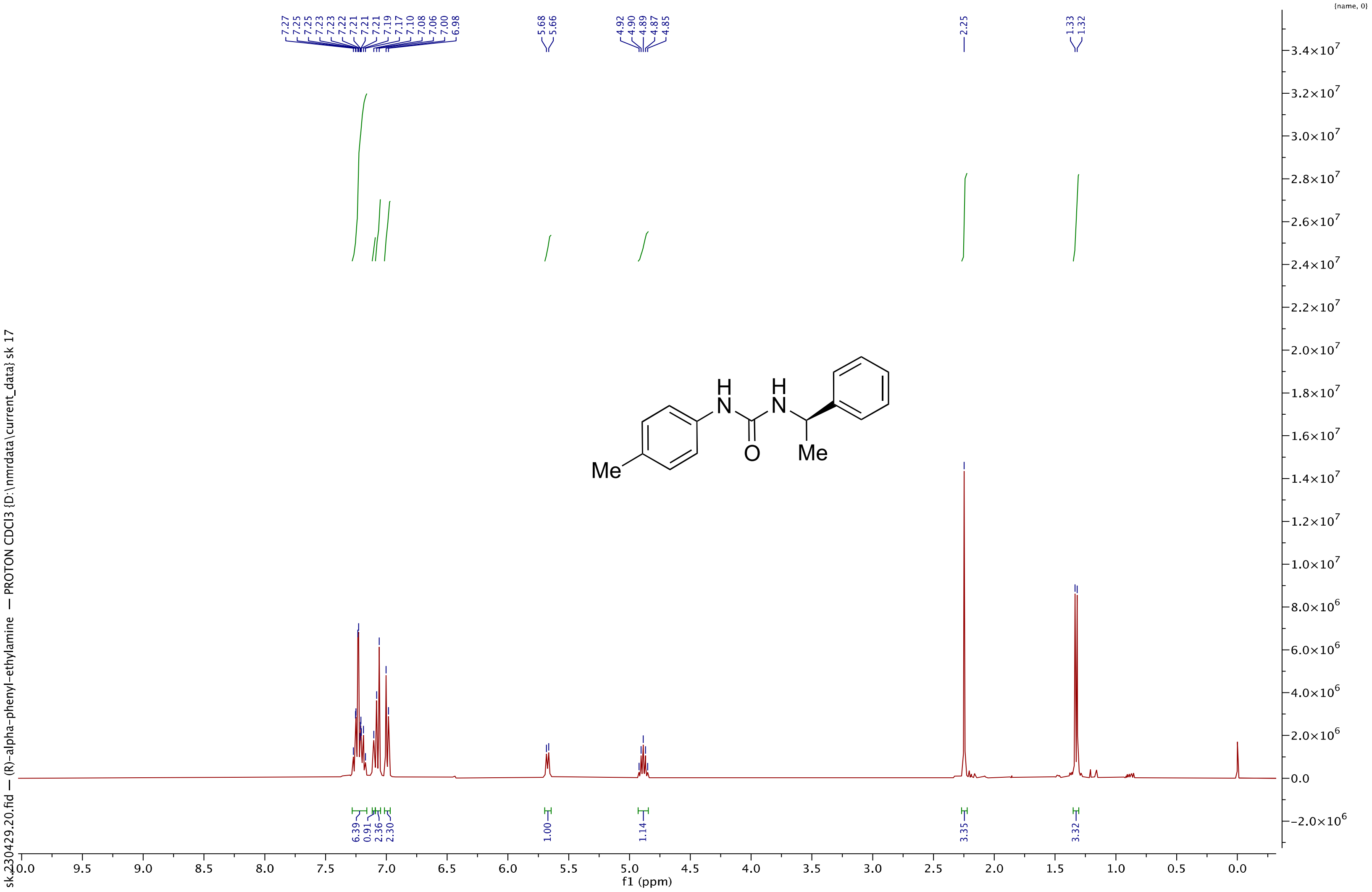

^{1}H NMR spectra of **3i** (400 MHz, RT, $CDCl_3$)

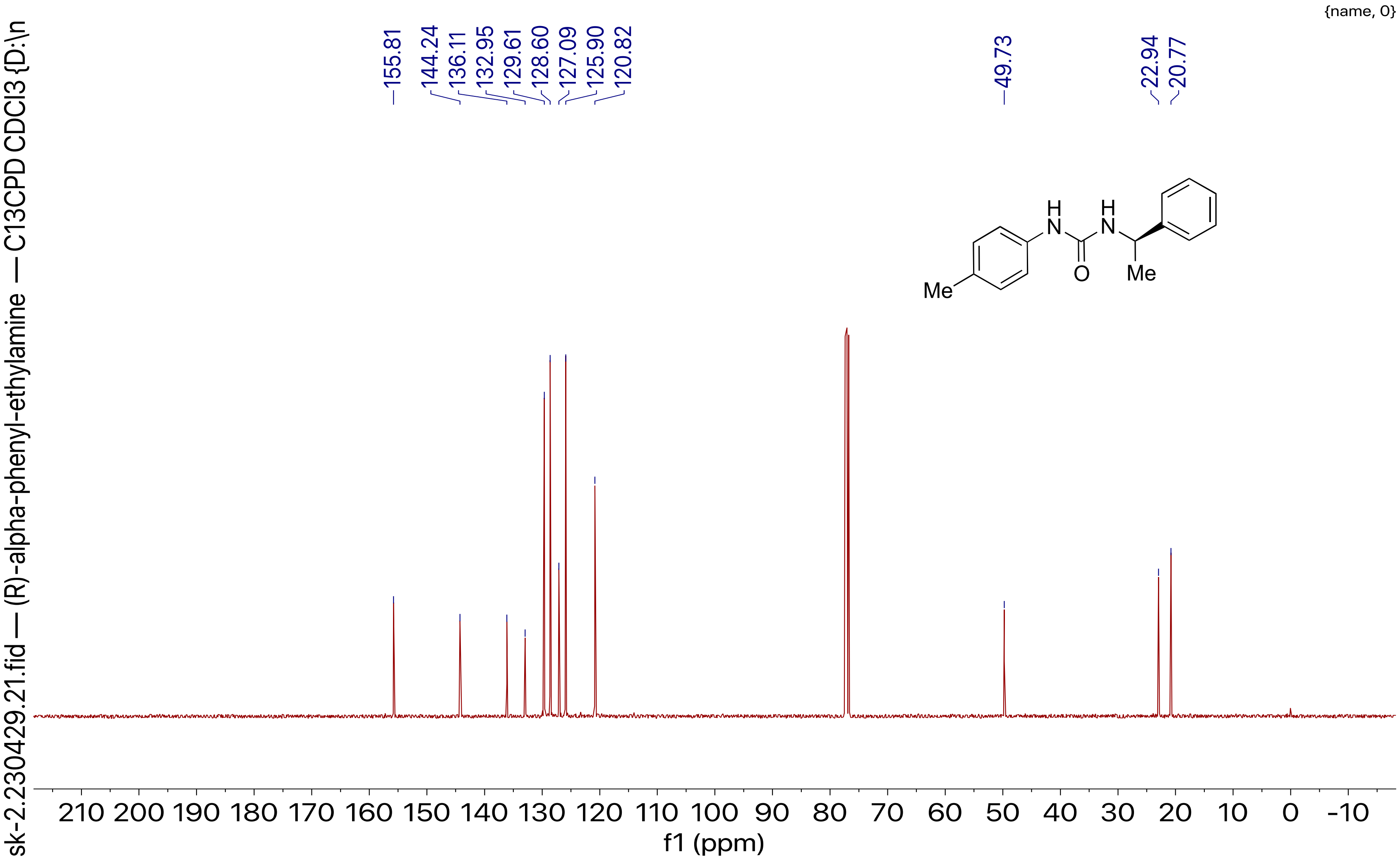

^{13}C NMR spectra of **3i** (101 MHz, RT, $CDCl_3$)

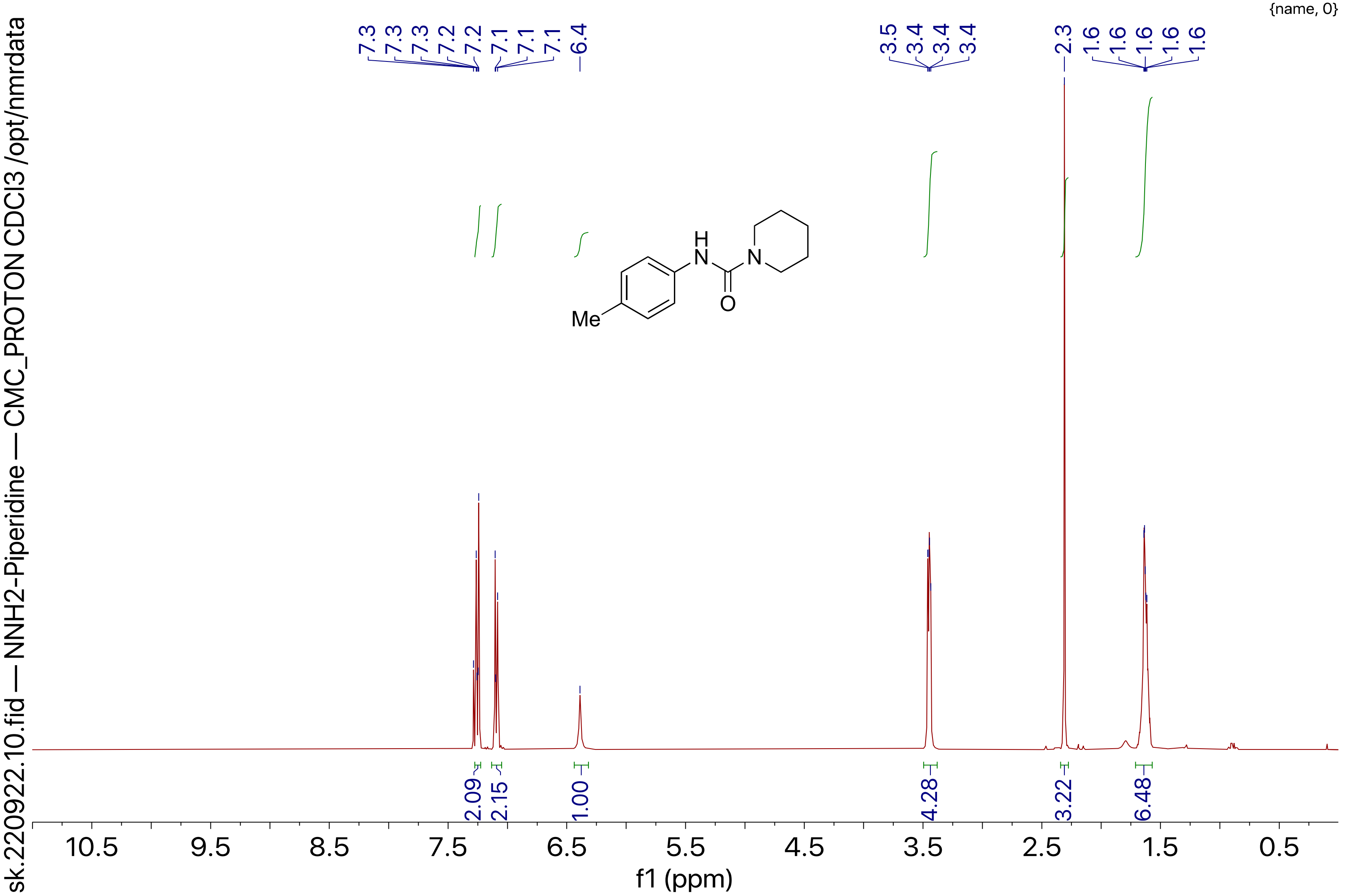

^{1}H NMR spectra of **5a** (400 MHz, RT, $CDCl_3$)

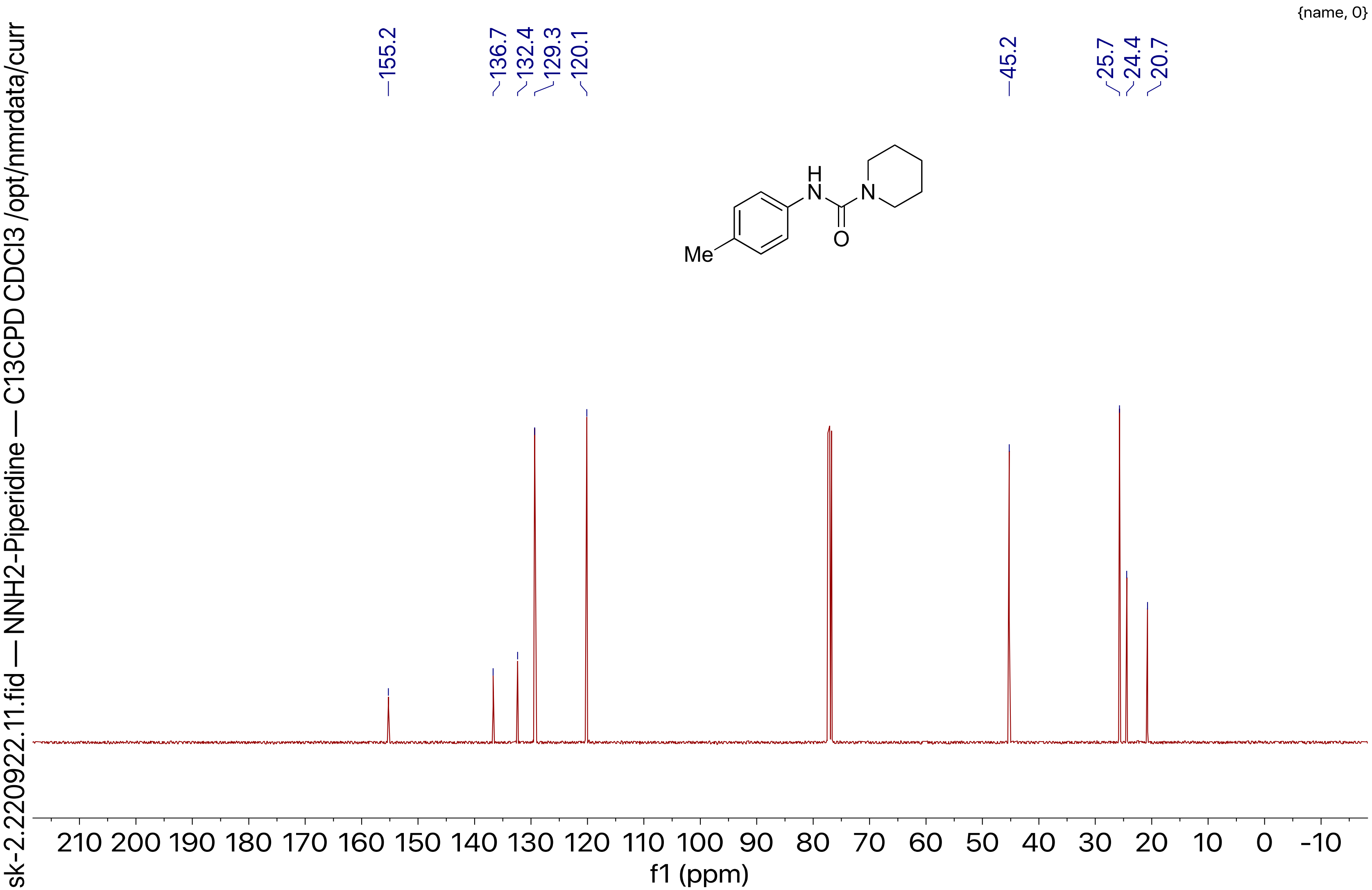

^{13}C NMR spectra of **5a** (101 MHz, RT, $CDCl_3$)

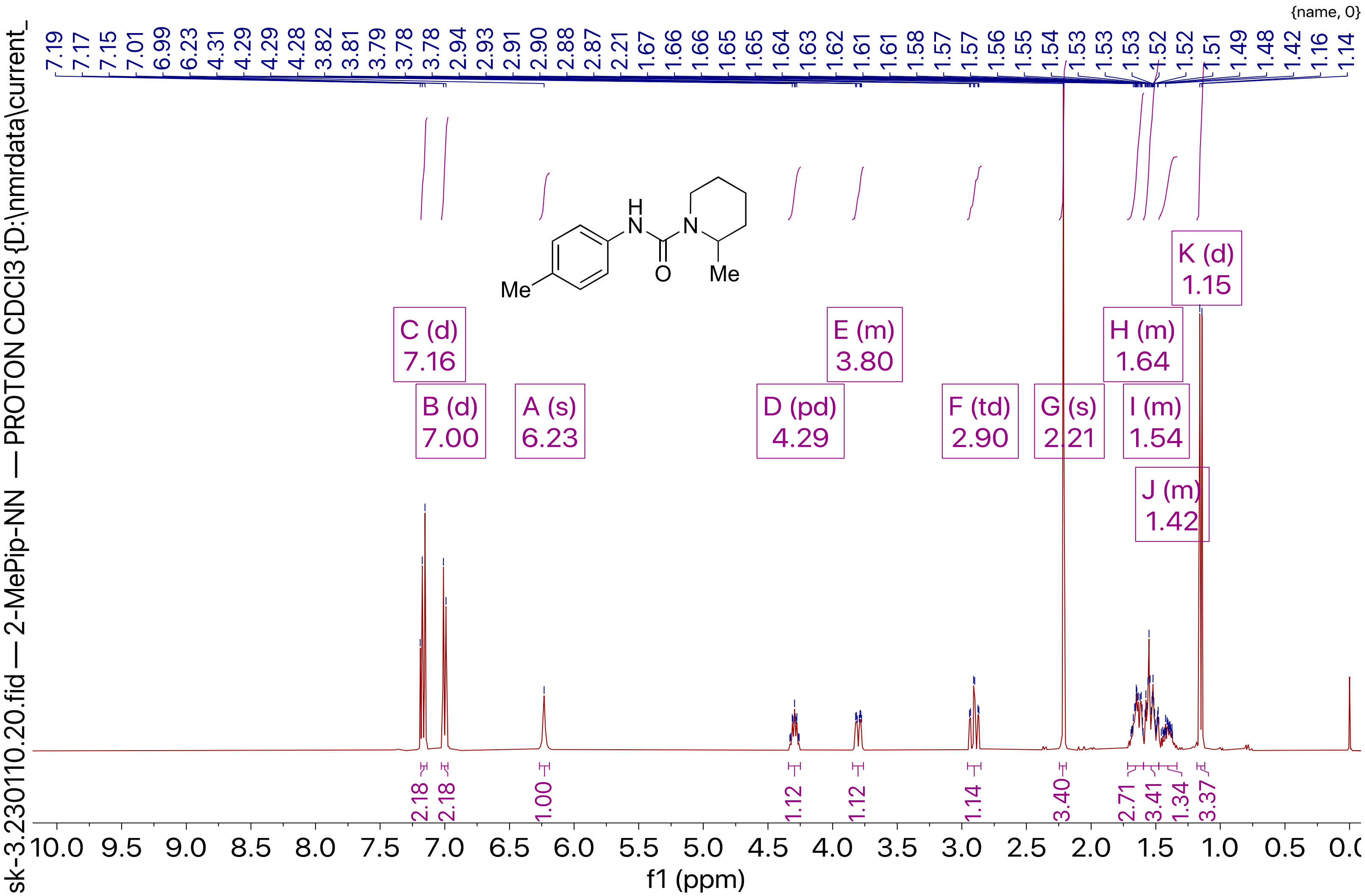

^{1}H NMR spectra of **5b** (400 MHz, RT, $CDCl_3$)

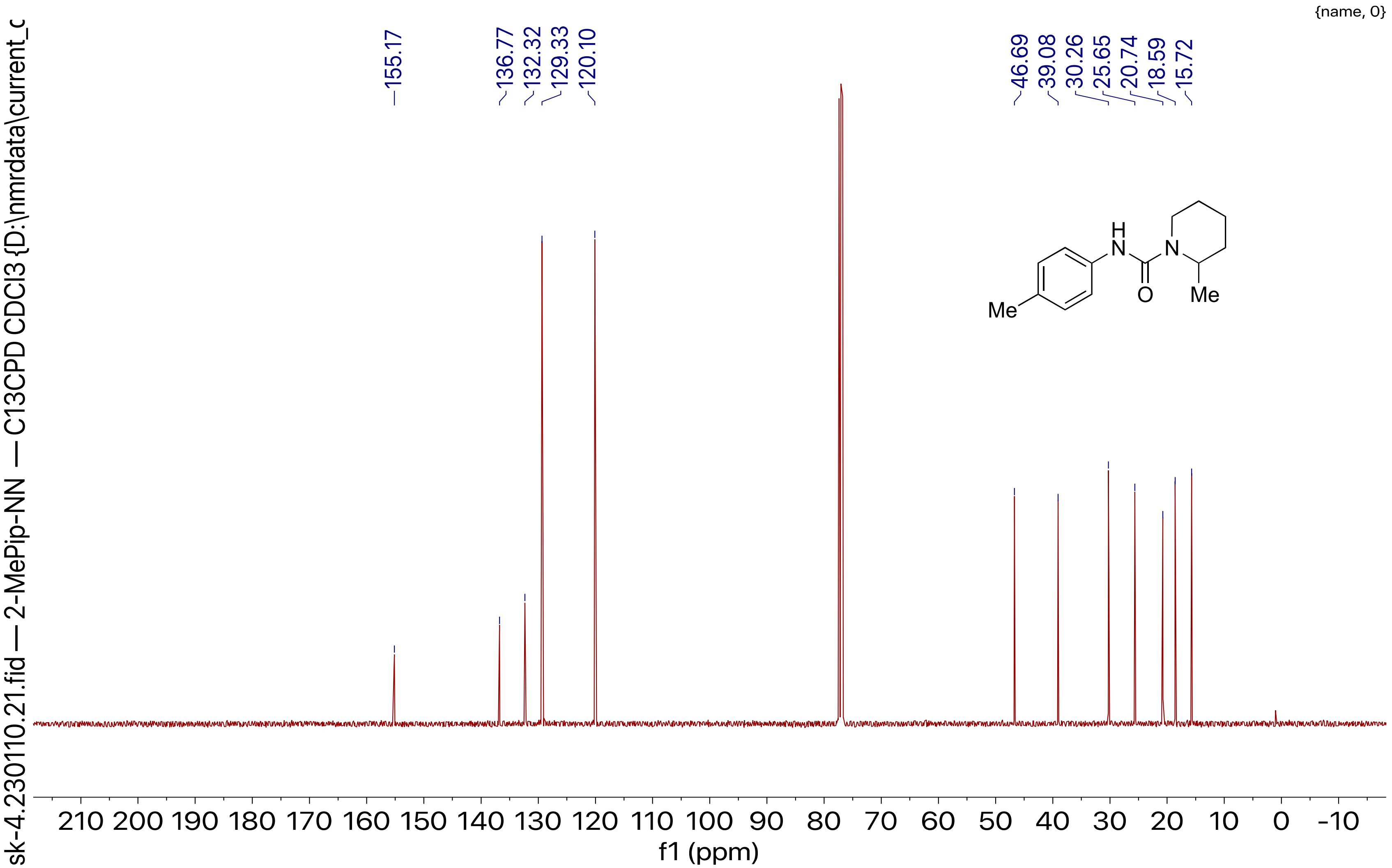

^{13}C NMR spectra of **5b** (100 MHz, RT, $CDCl_3$)

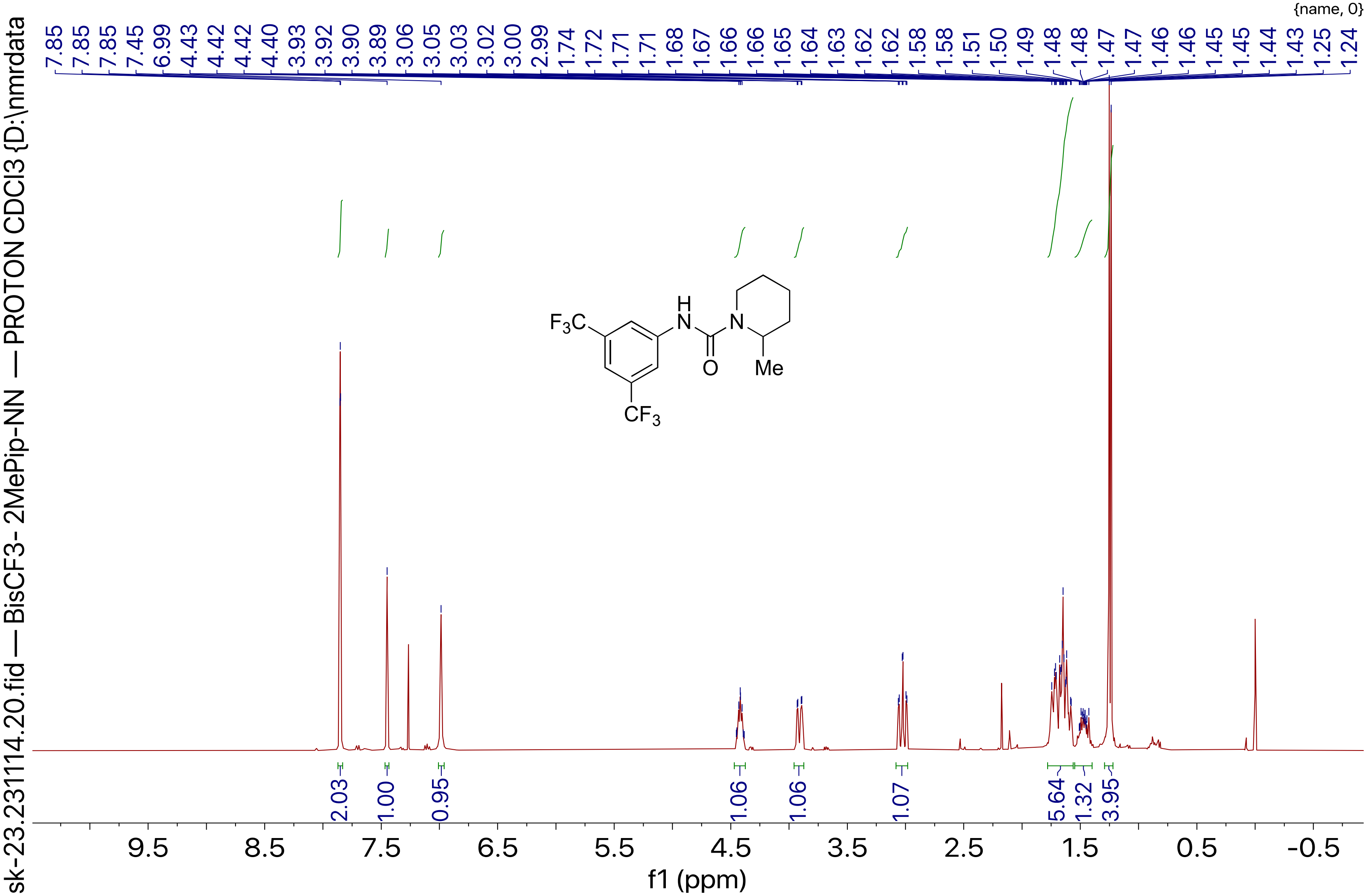

^{1}H NMR spectra of **5b'** (400 MHz, RT, $CDCl_3$)

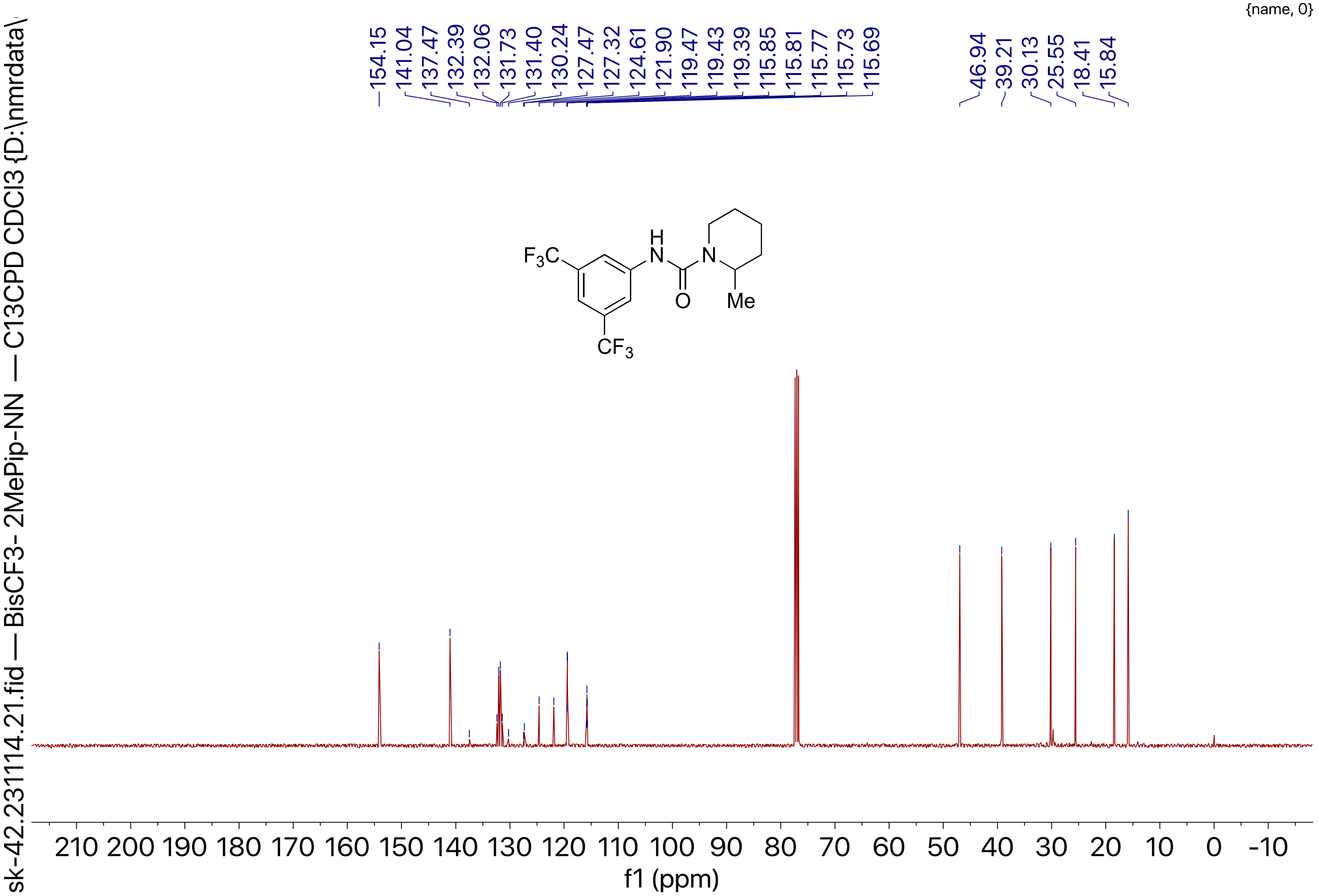

^{13}C NMR spectra of **5b’** (101 MHz, RT, $CDCl_3$)

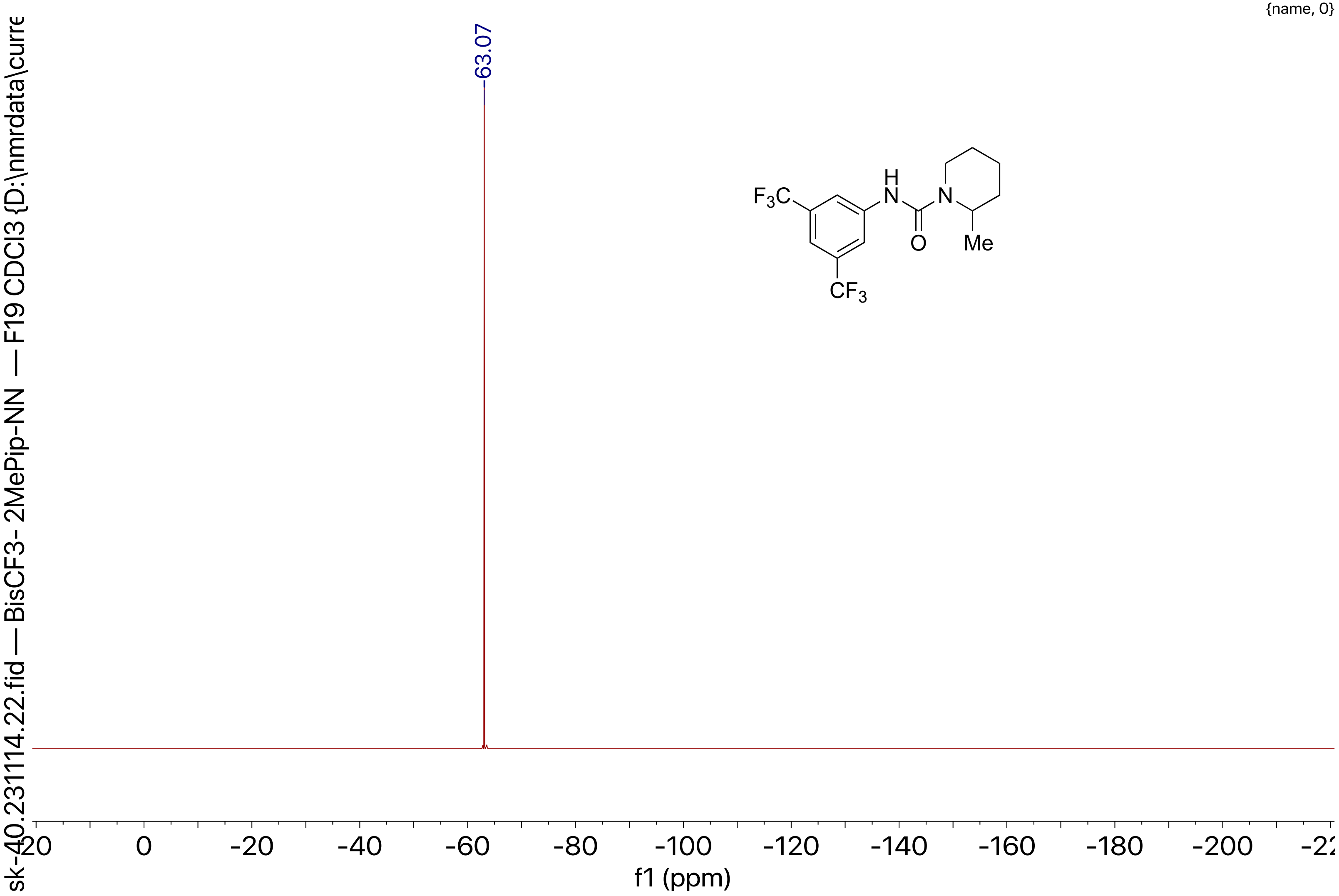

^{19}F NMR spectra of **5b'** (376 MHz, RT, $CDCl_3$)

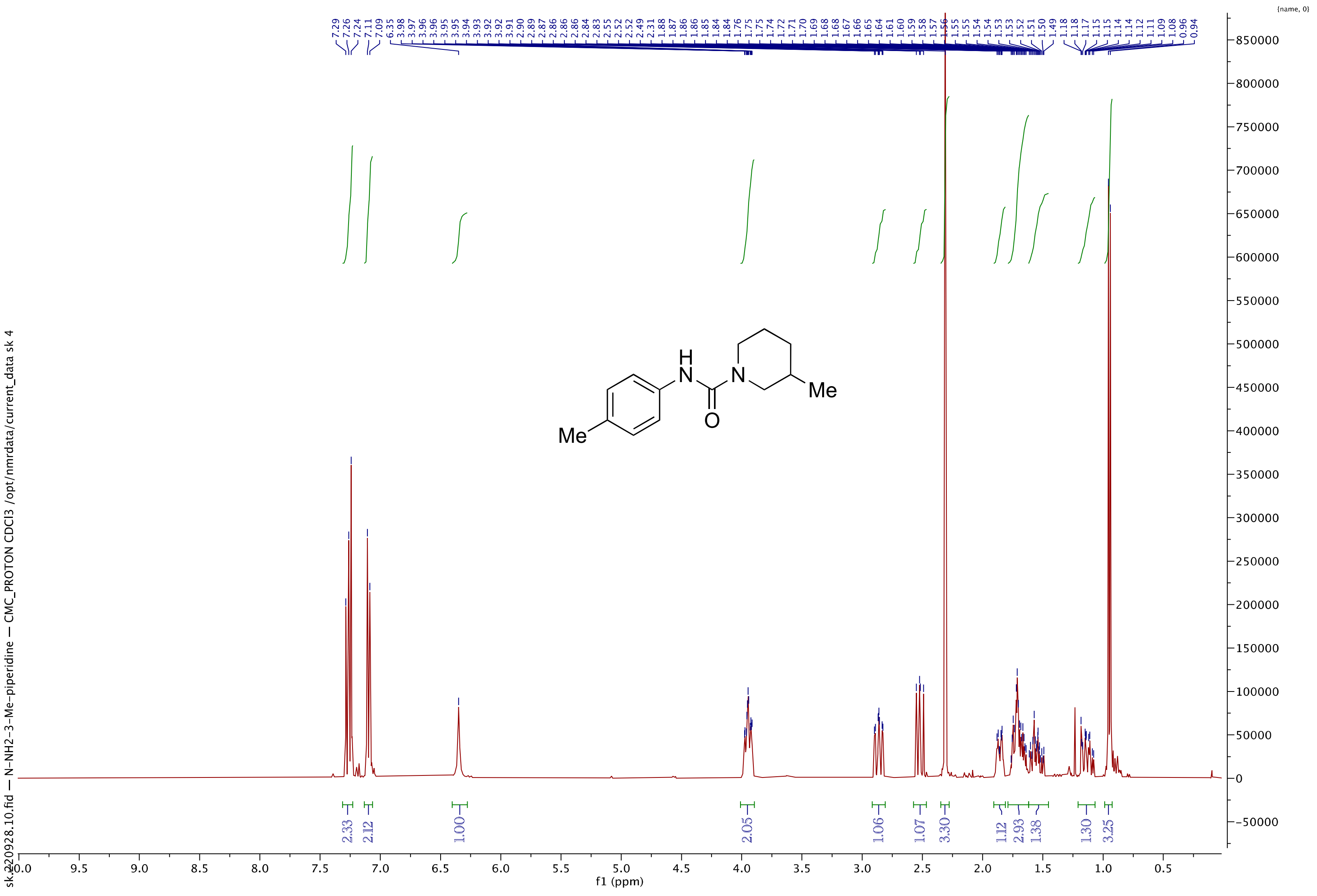

^{1}H NMR spectra of **5c** (400 MHz, RT, $CDCl_3$)

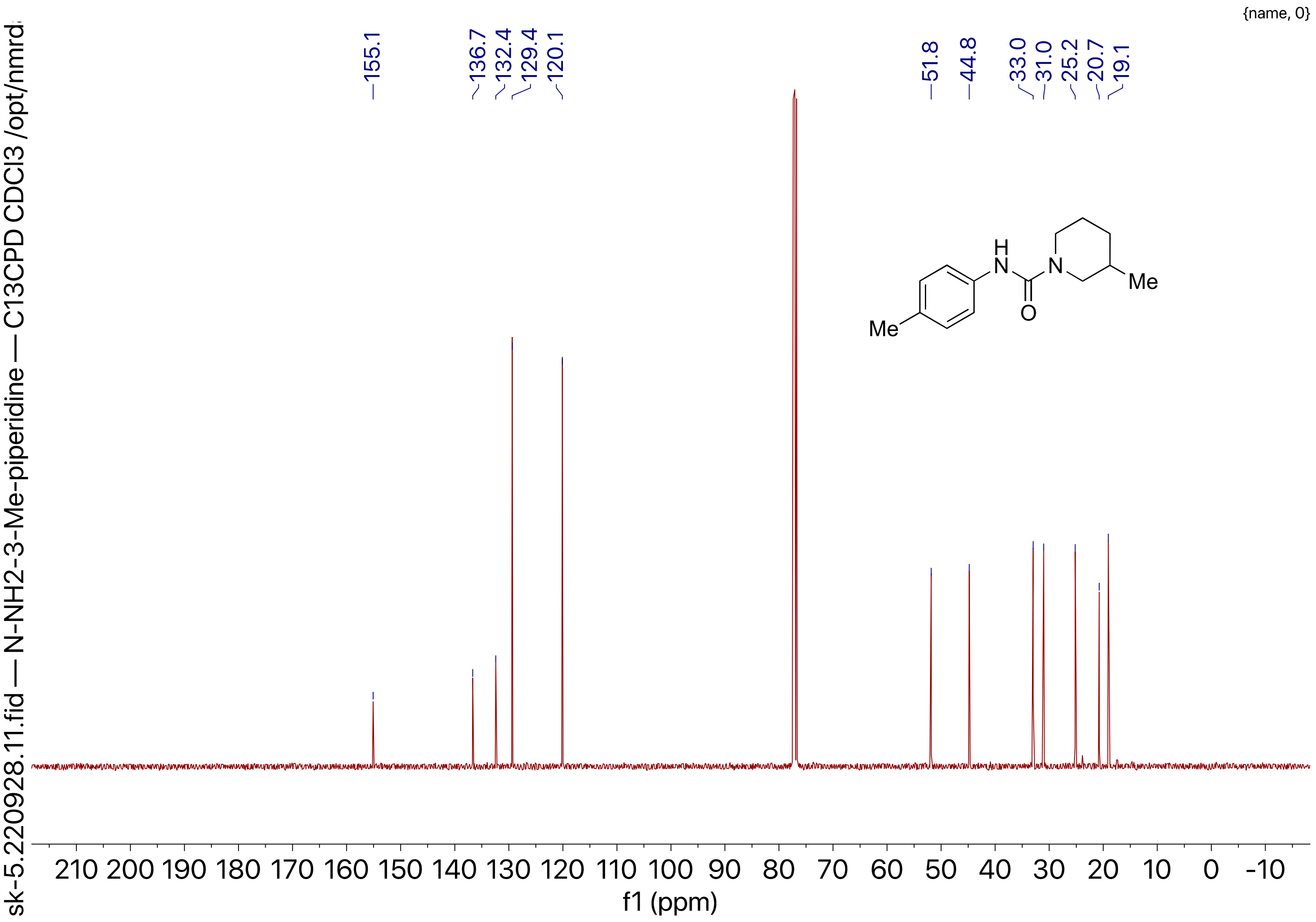

^{13}C NMR spectra of **5c** (101 MHz, RT, $CDCl_3$)

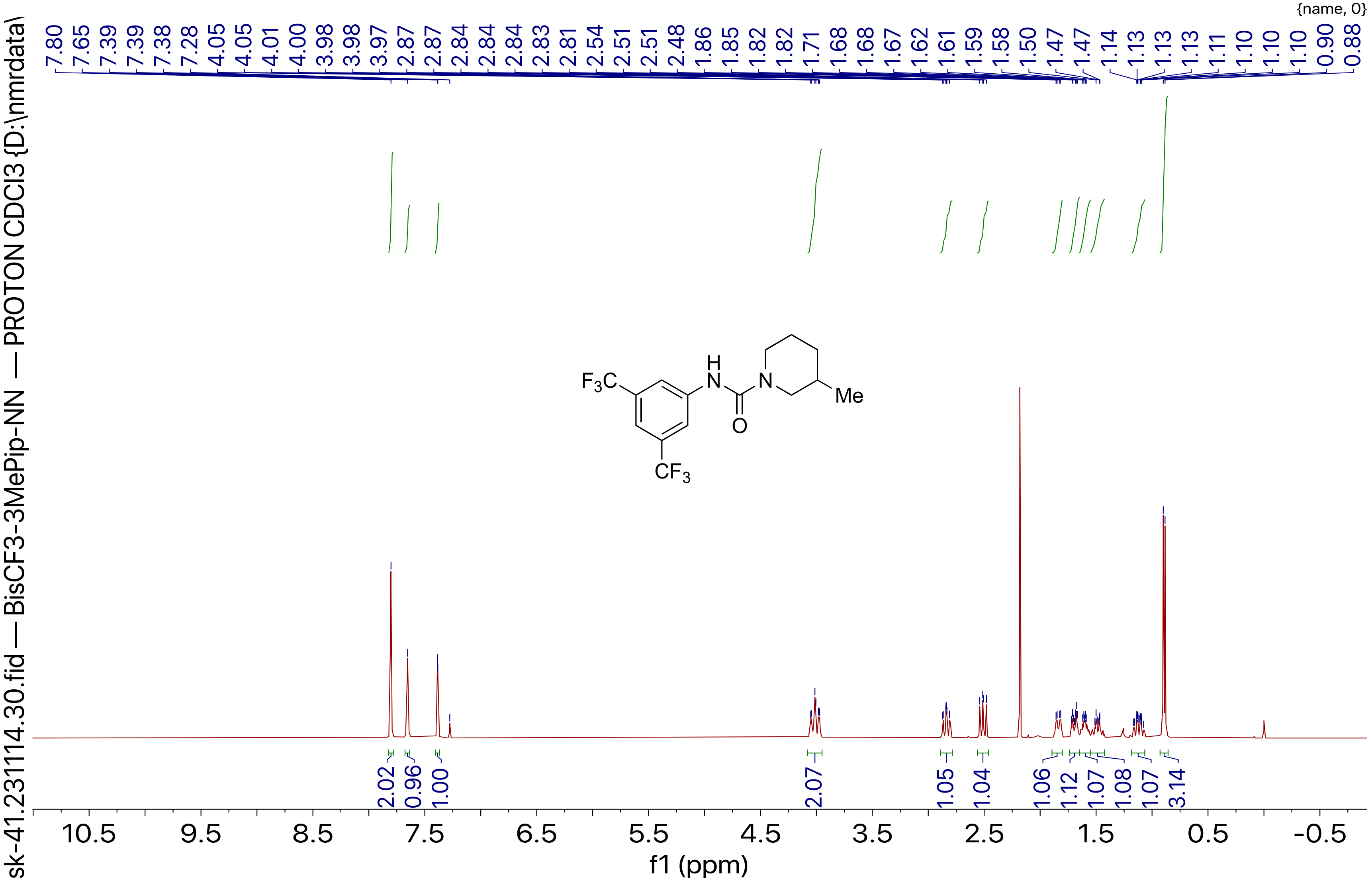

^{1}H NMR spectra of **5c’** (400 MHz, RT, $CDCl_3$)

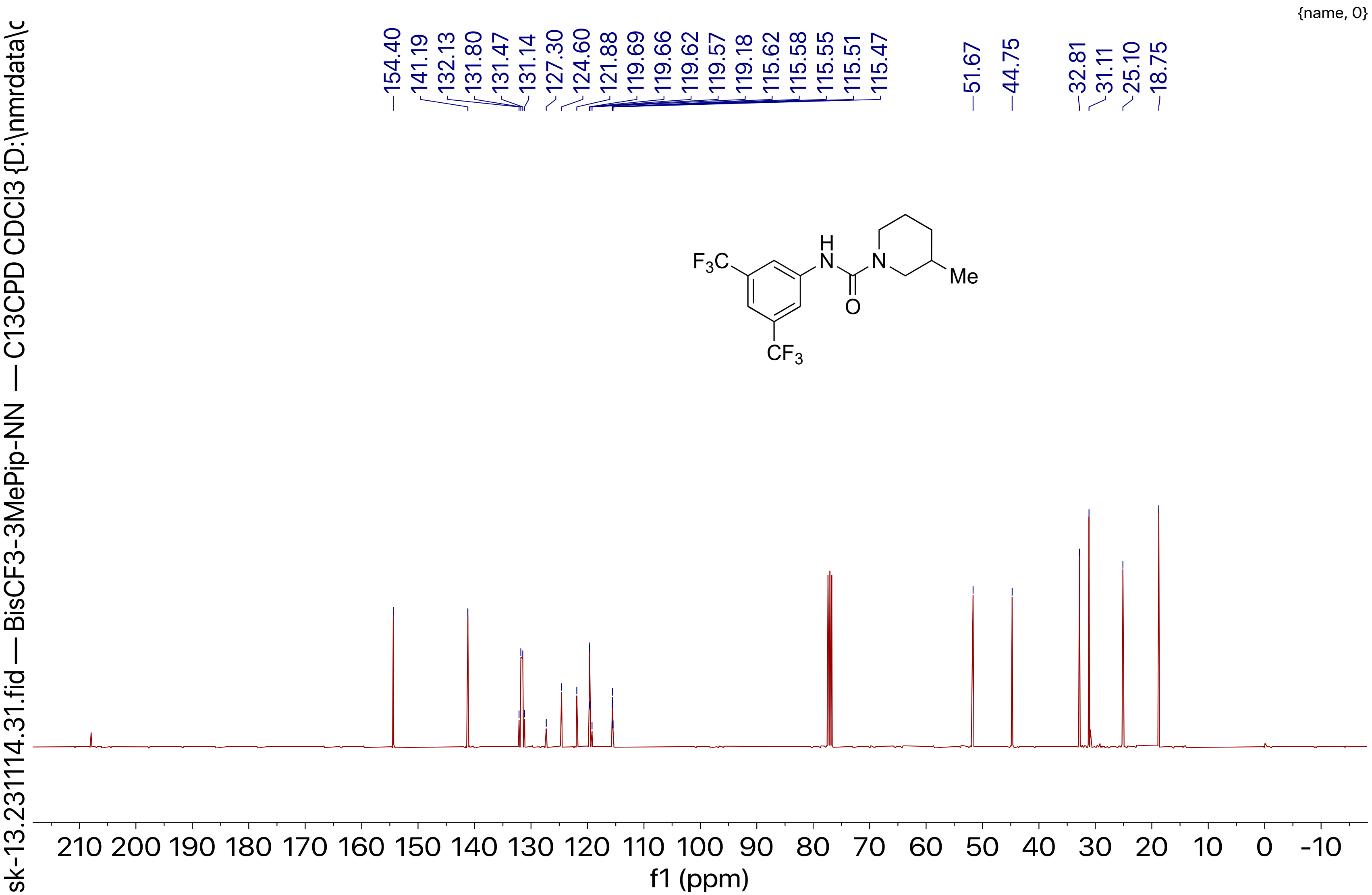

^{13}C NMR spectra of **5c’** (101 MHz, RT, $CDCl_3$)

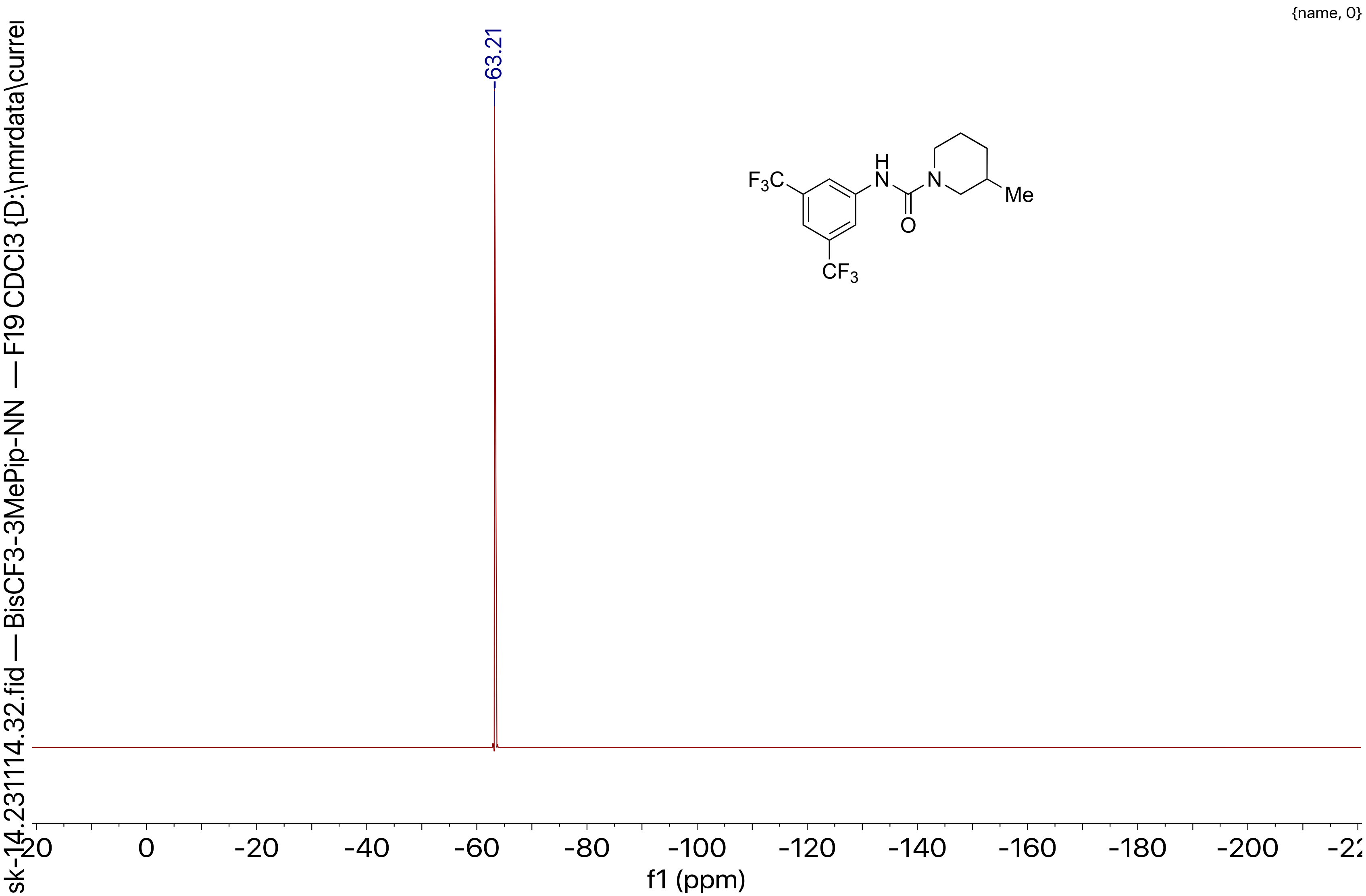

^{19}F NMR spectra of **5c’** (376 MHz, RT, $CDCl_3$)

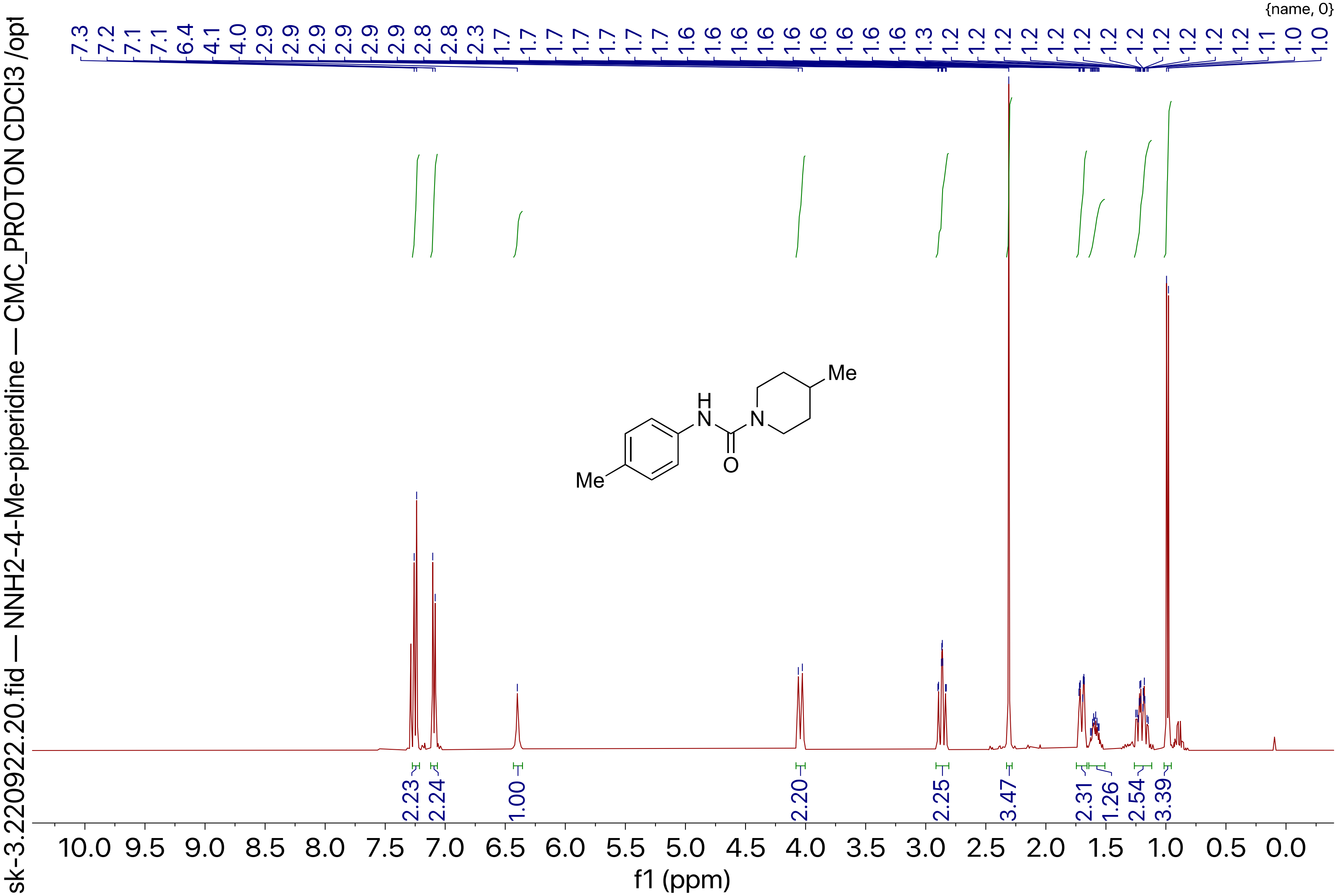

^1H NMR spectra of **5d** (400 MHz, RT, $CDCl_3$)

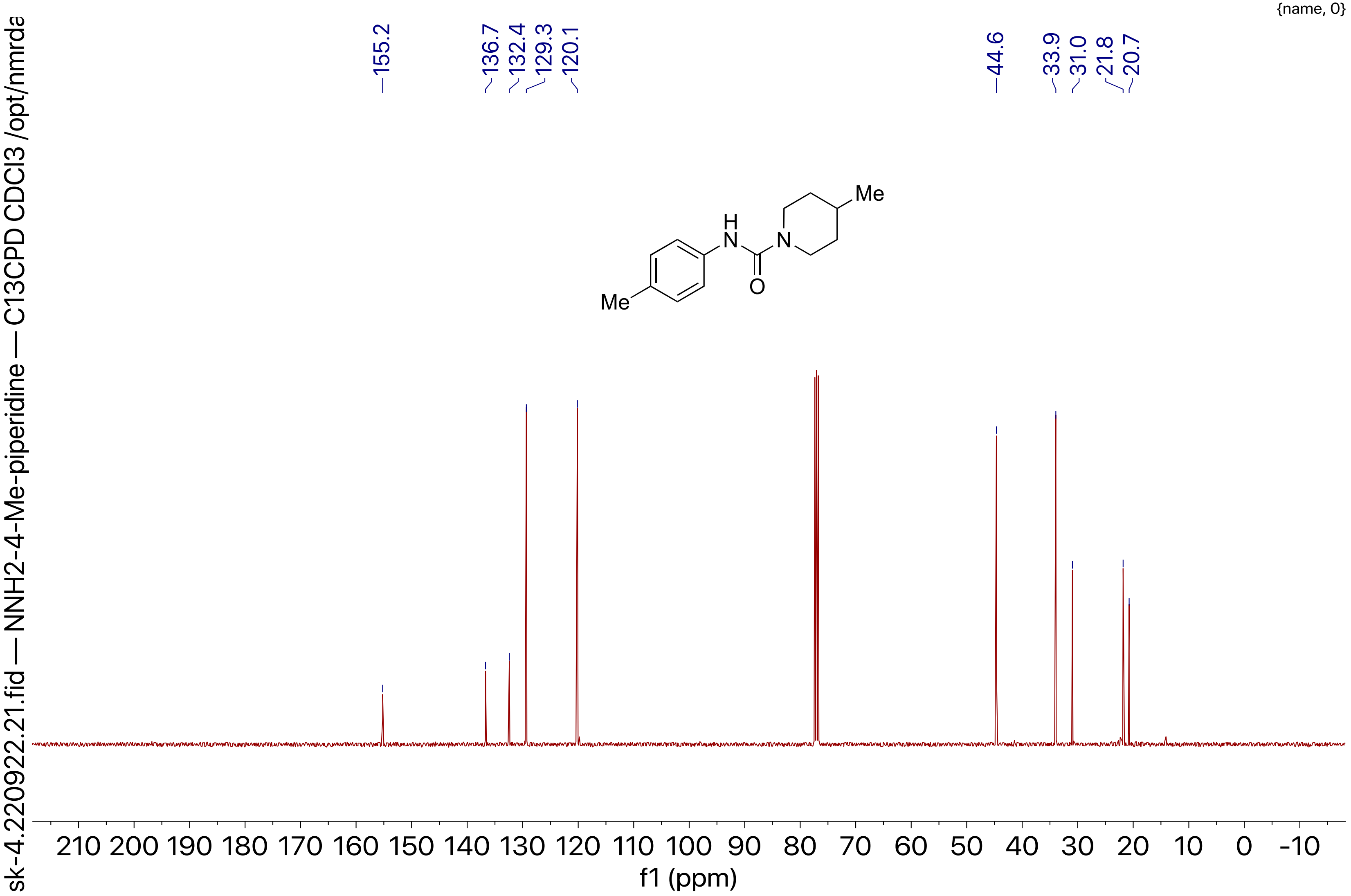

^{13}C NMR spectra of **5d** (100 MHz, RT, $CDCl_3$)

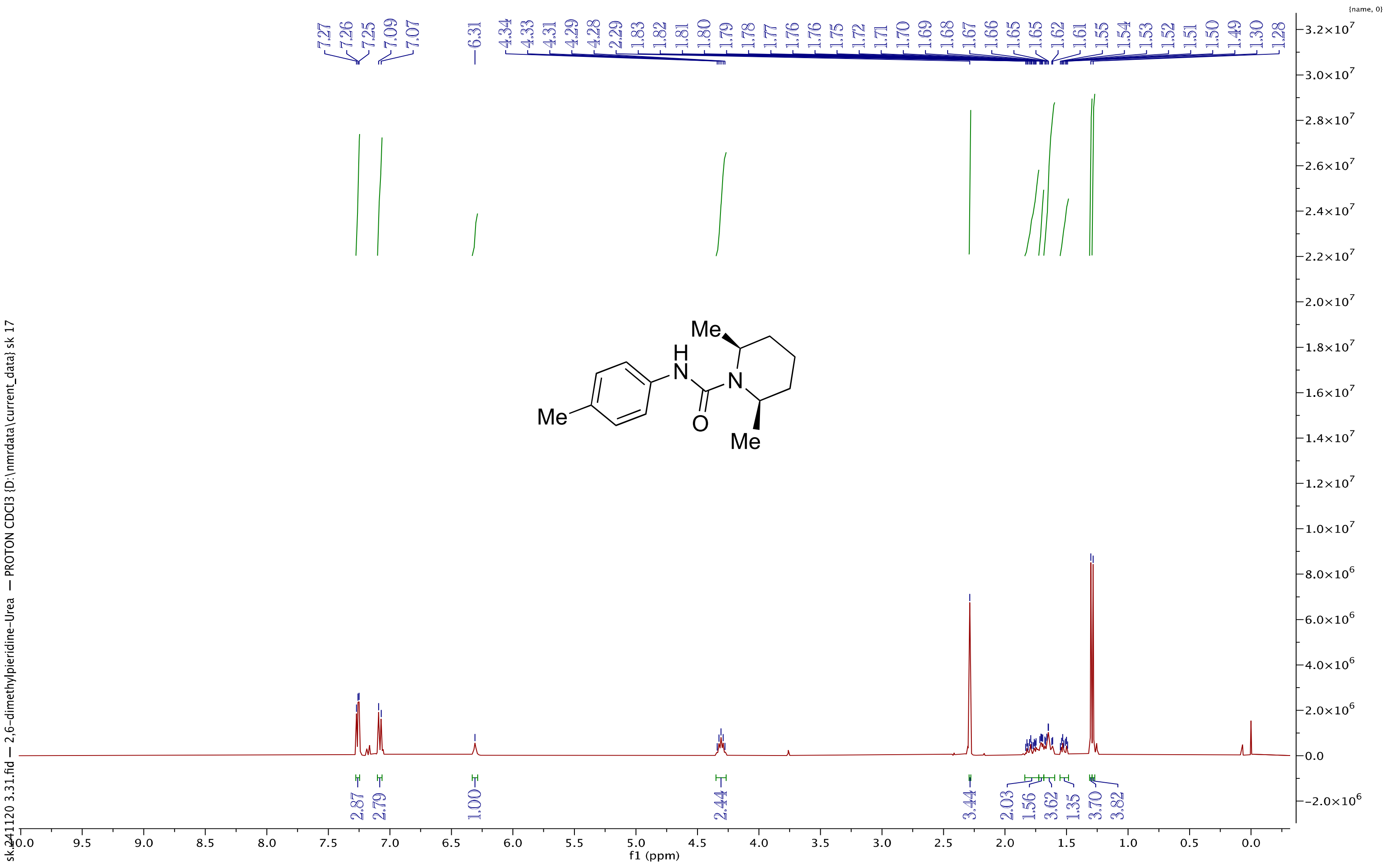

^{1}H NMR spectra of **5e** (400 MHz, RT, $CDCl_3$)

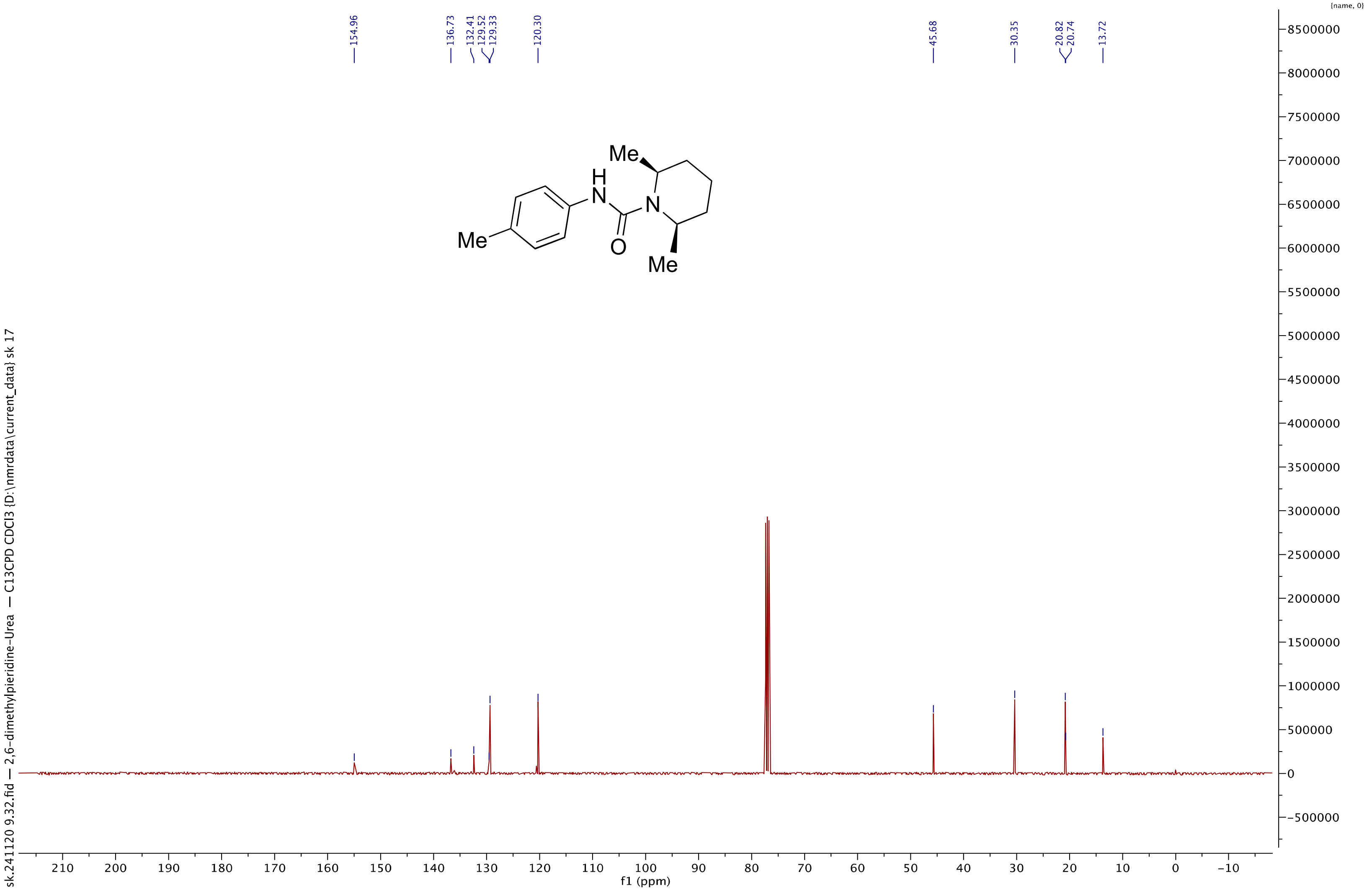

^{13}C NMR spectra of **5e** (100 MHz, RT, $CDCl_3$)

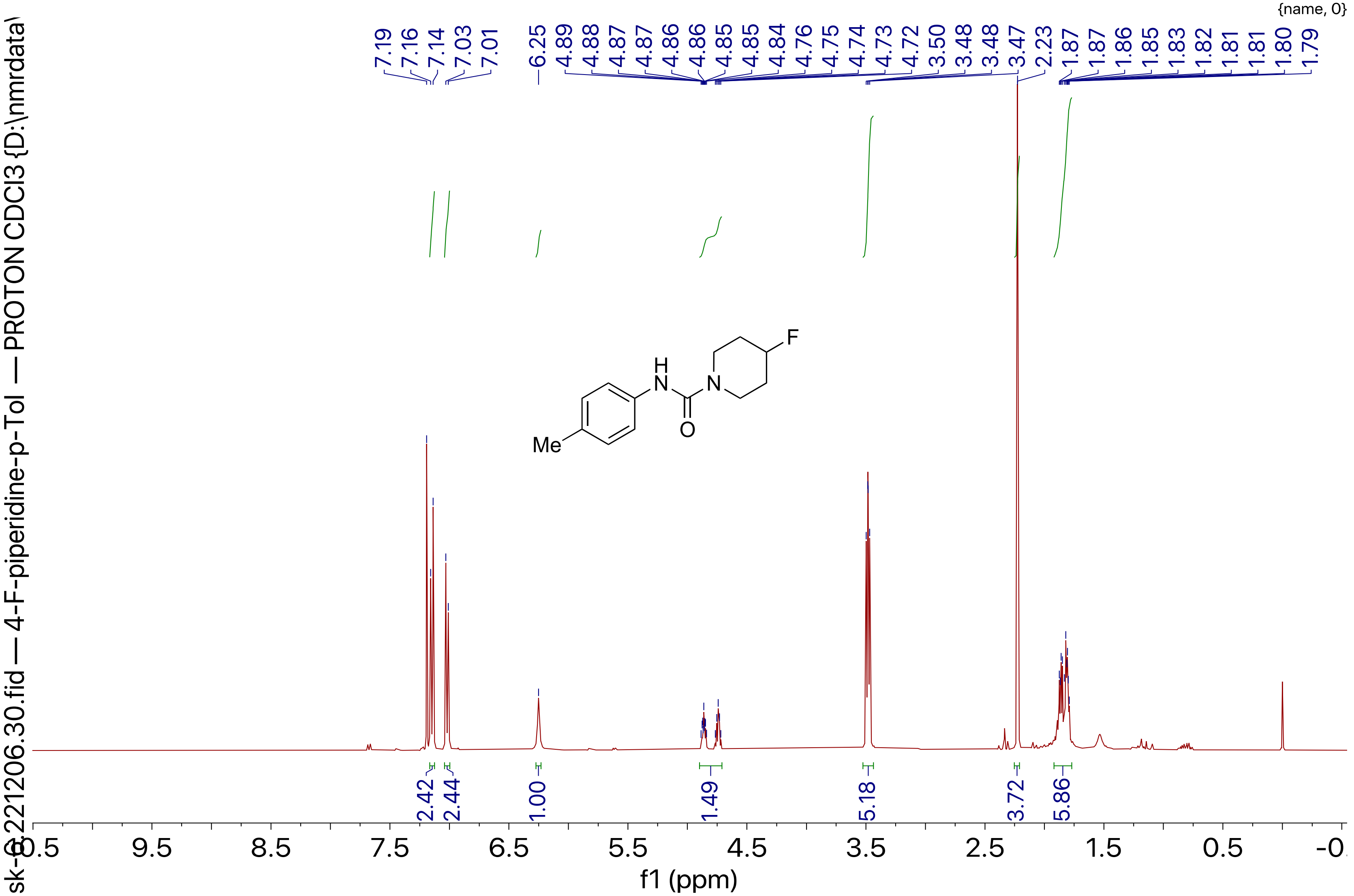

^{1}H NMR spectra of **5f** (400 MHz, RT, $CDCl_3$)

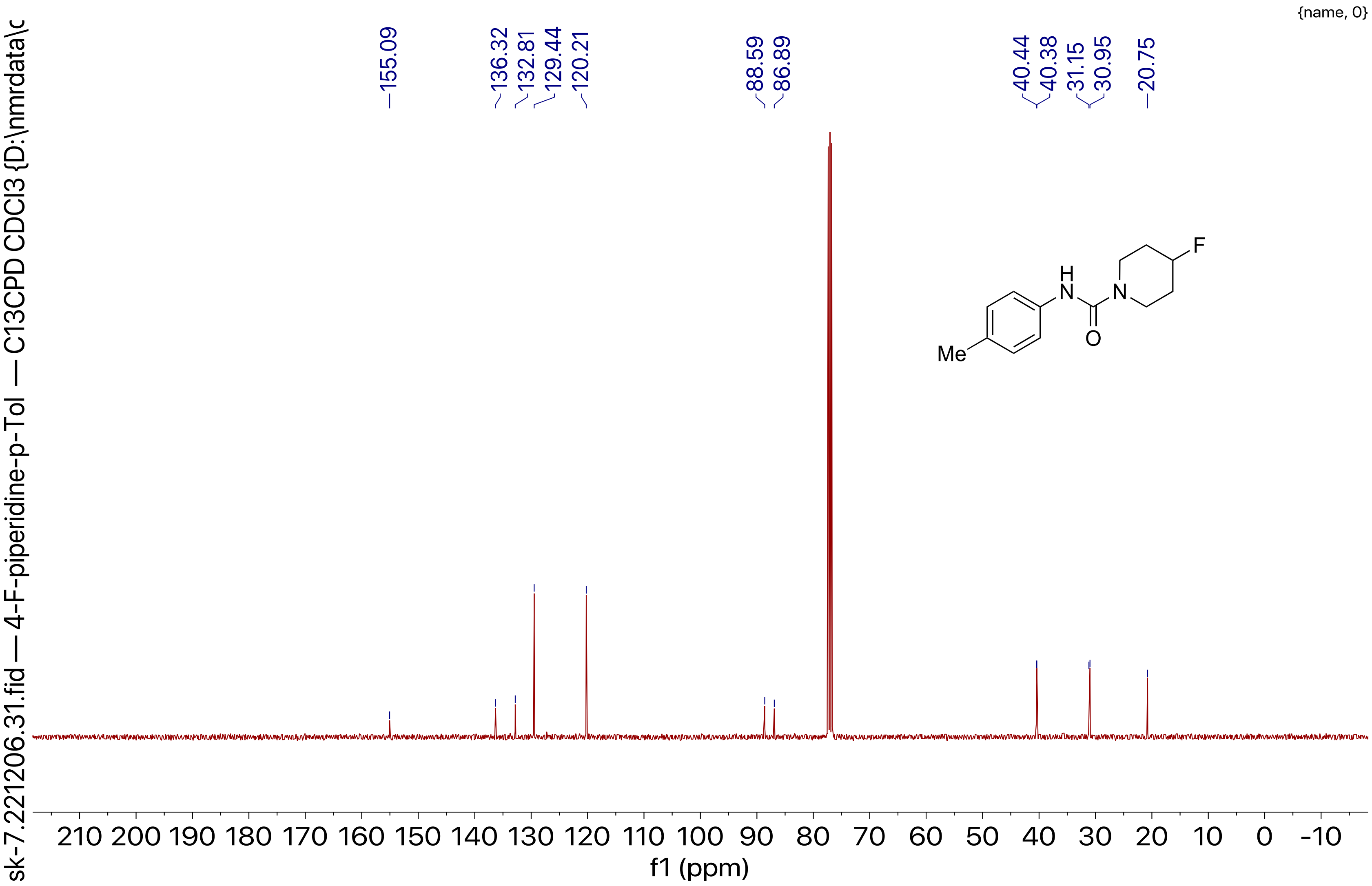

^{13}C NMR spectra of **5f** (101 MHz, RT, $CDCl_3$)

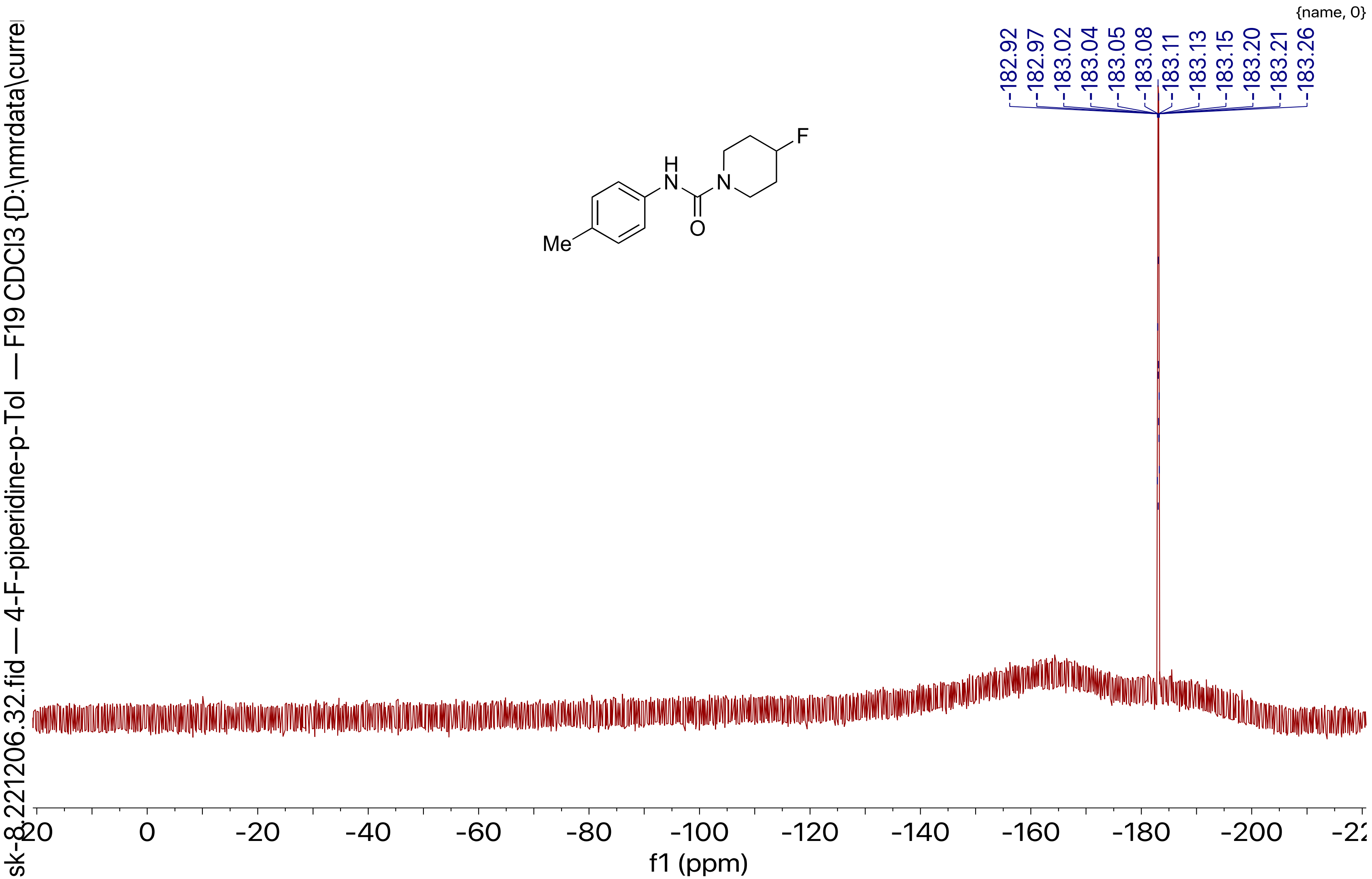

^{19}F NMR spectra of **5f** (376 MHz, RT, $CDCl_3$)

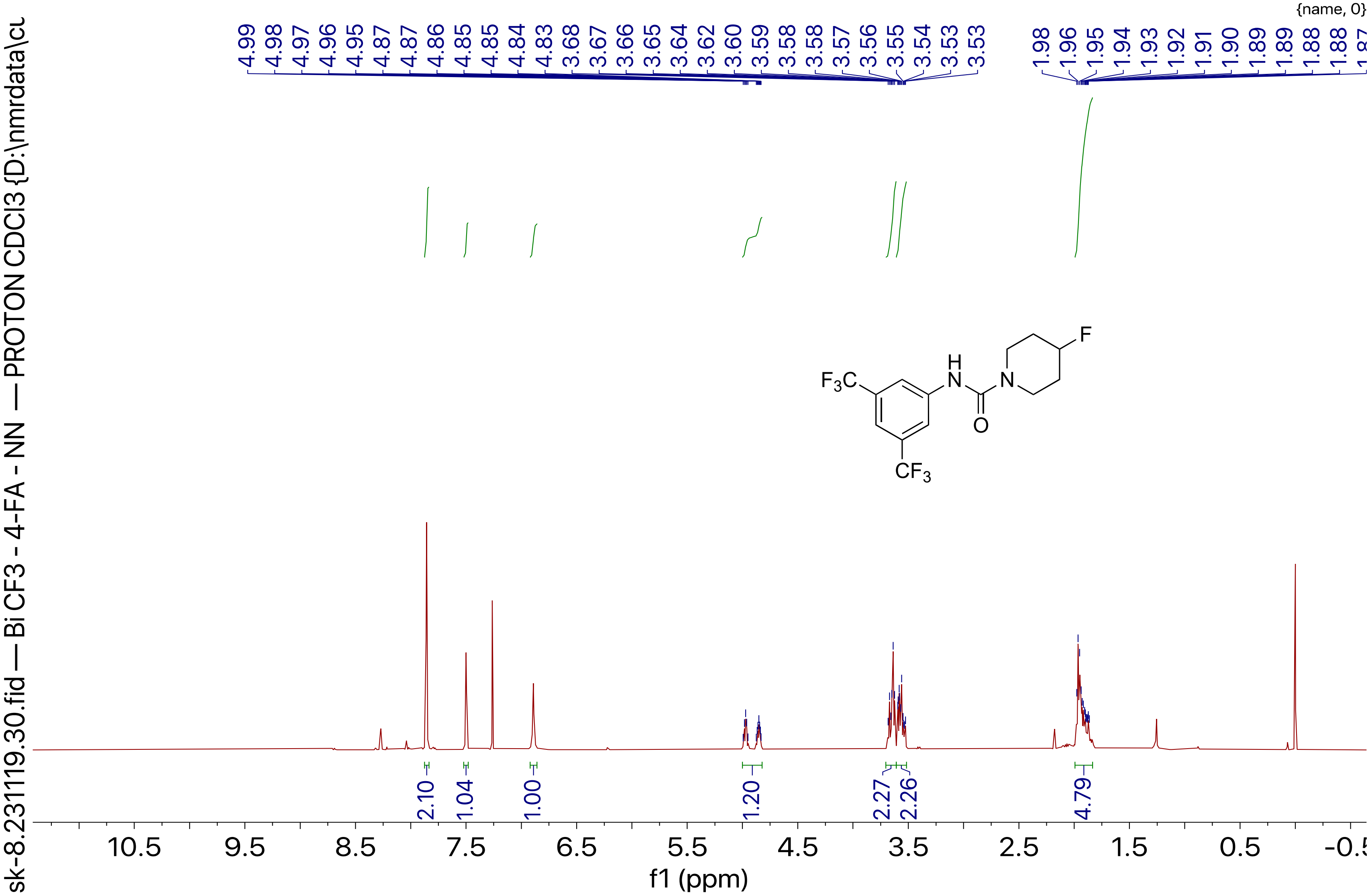

^{1}H NMR spectra of **5f’** (400 MHz, RT, $CDCl_3$)

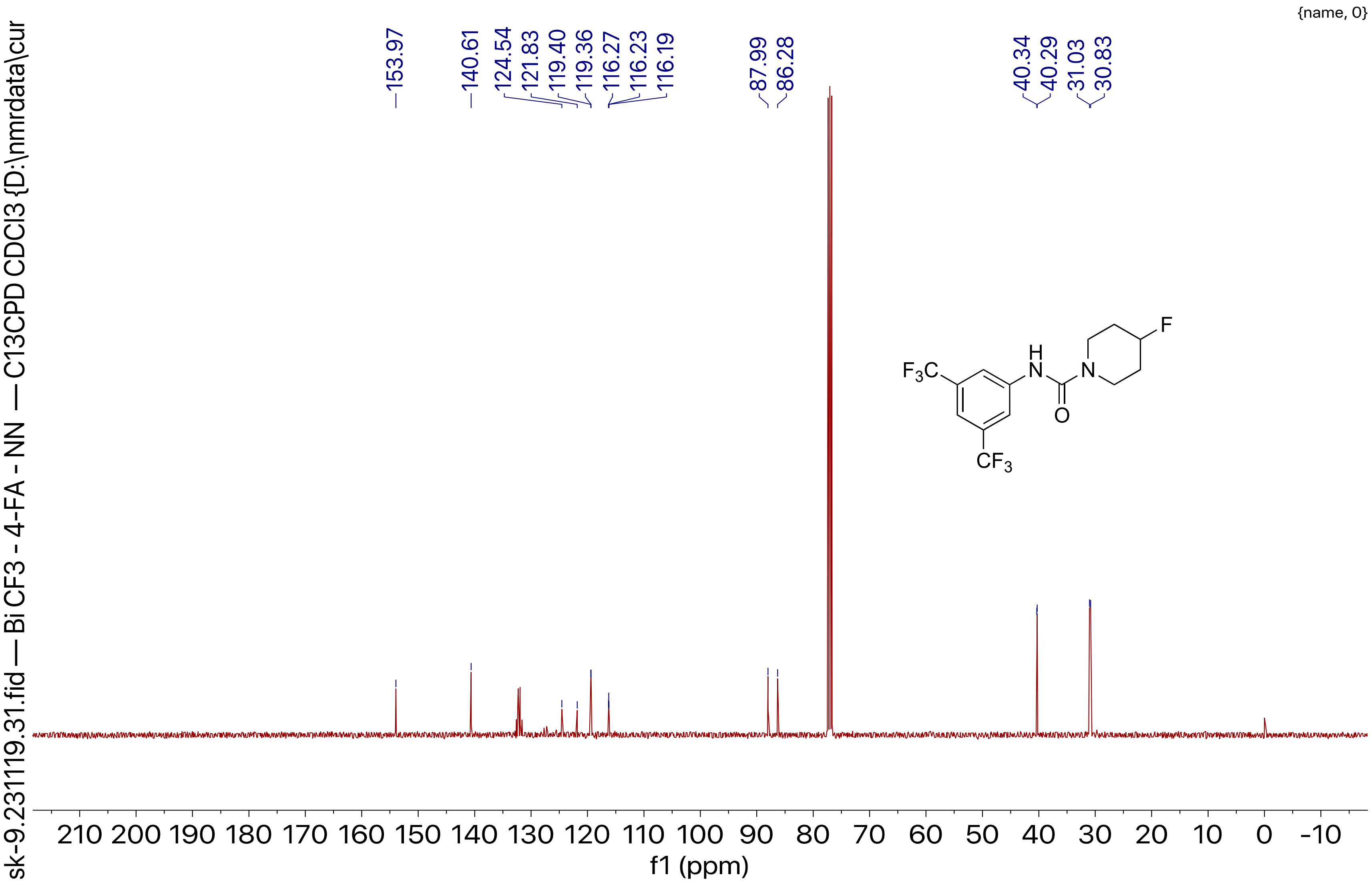

^{13}C NMR spectra of **5f’** (101 MHz, RT, $CDCl_3$)

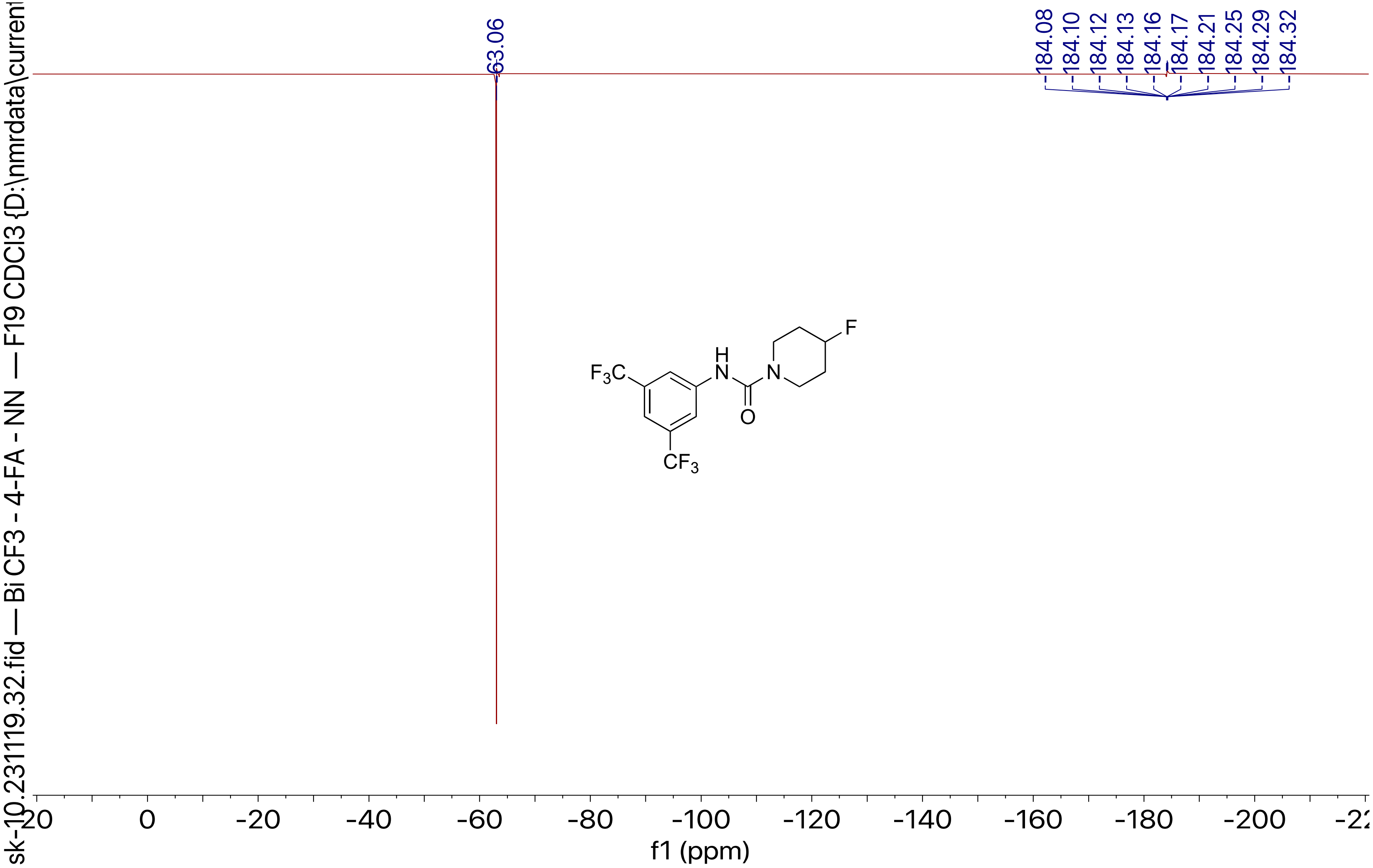

^{19}F NMR spectra of **5f'** (376 MHz, RT, $CDCl_3$)

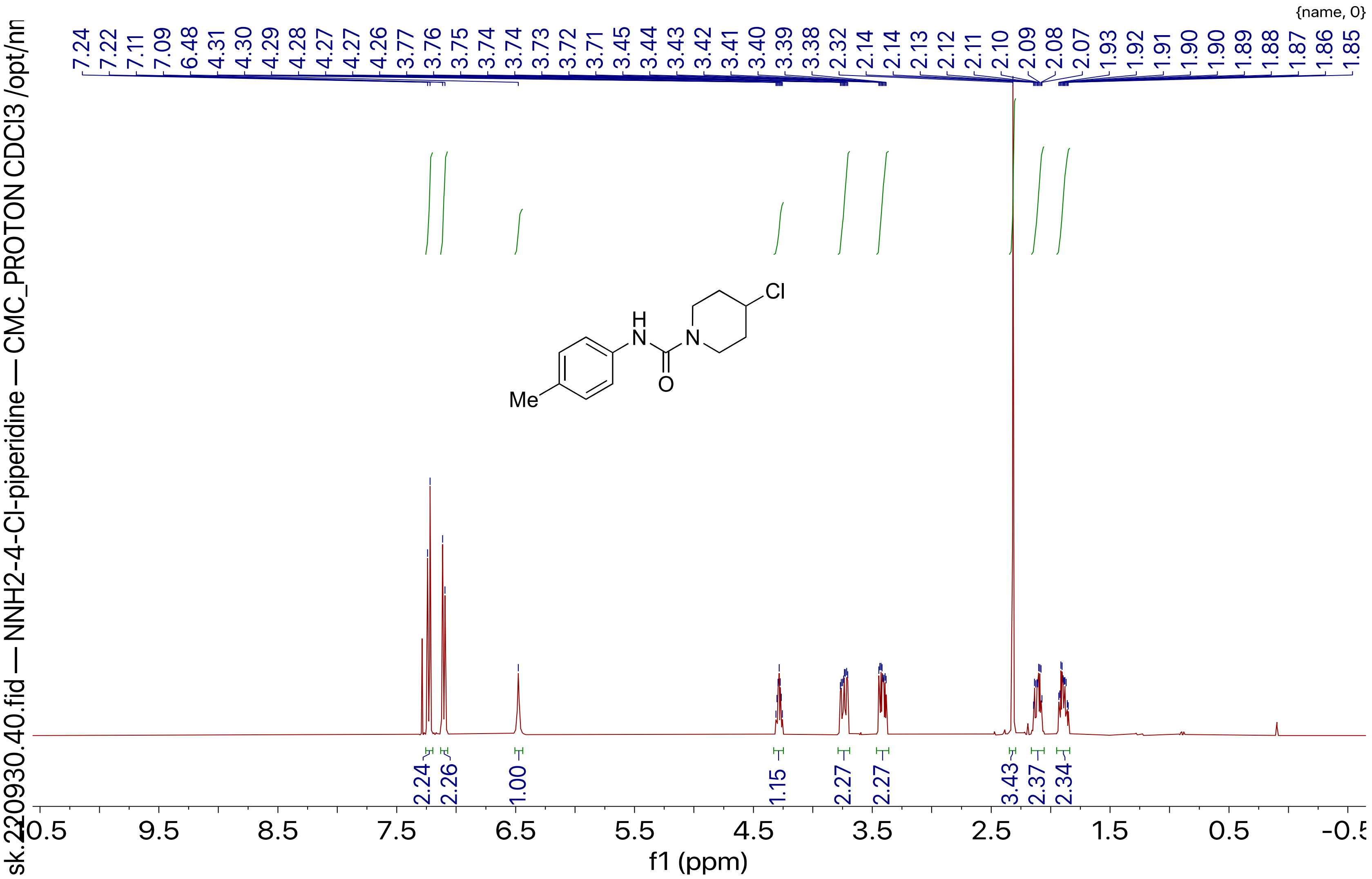

^{1}H NMR spectra of **5g** (400 MHz, RT, $CDCl_3$)

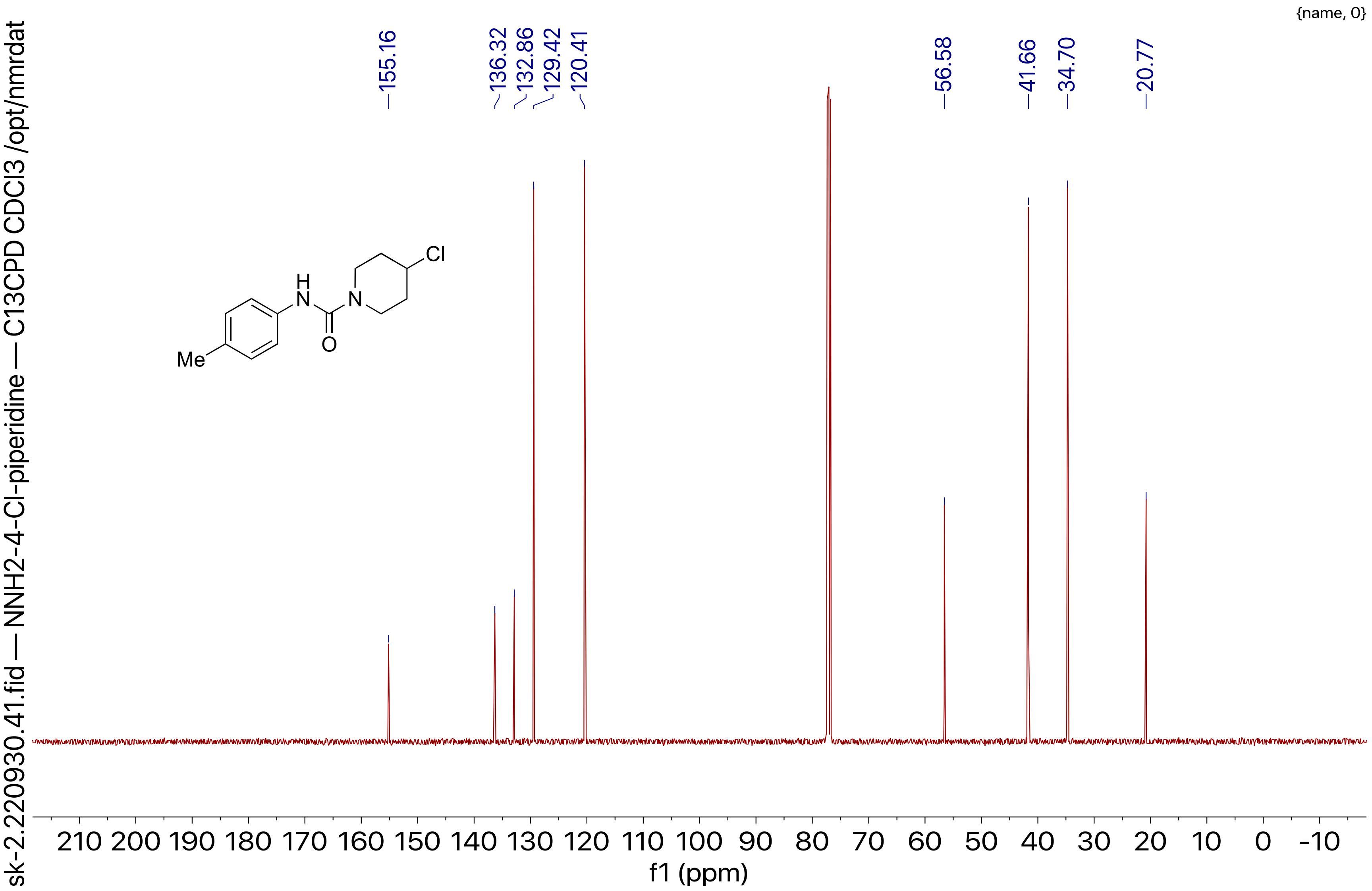

^{13}C NMR spectra of **5g** (100 MHz, RT, $CDCl_3$)

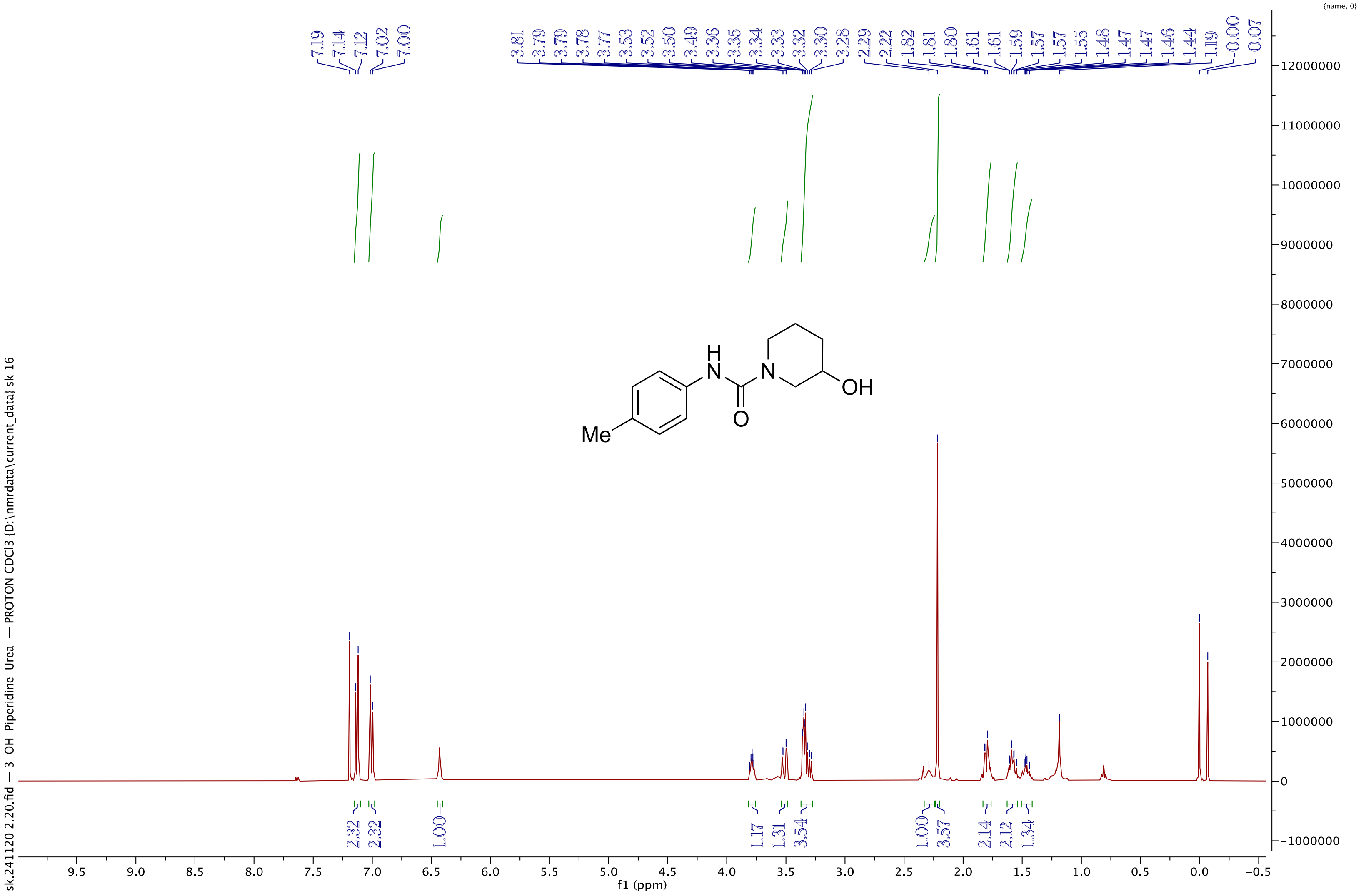

^{1}H NMR spectra of **5h** (400 MHz, RT, $CDCl_3$)

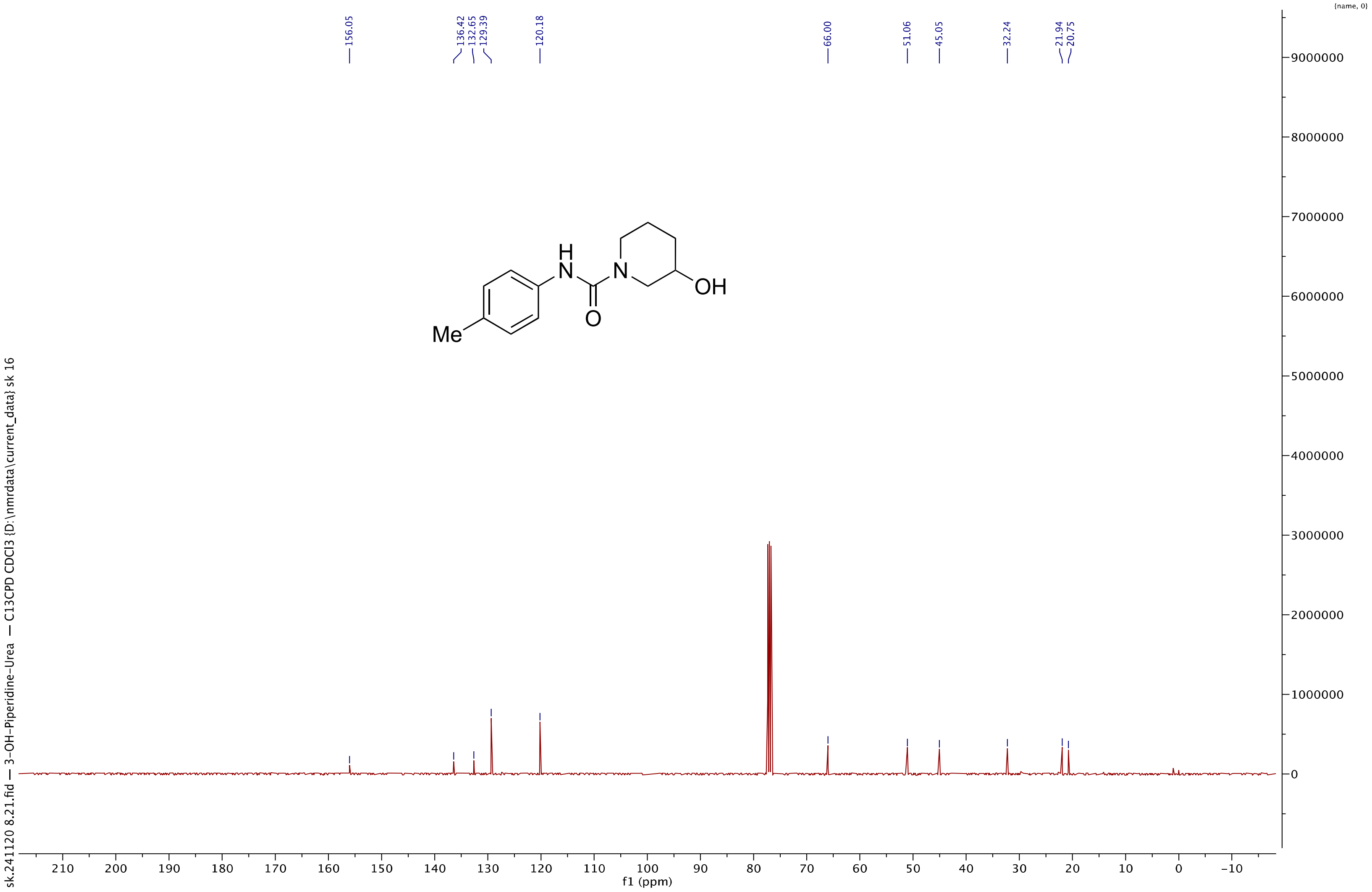

^{13}C NMR spectra of **5h** (101 MHz, RT, $CDCl_3$)

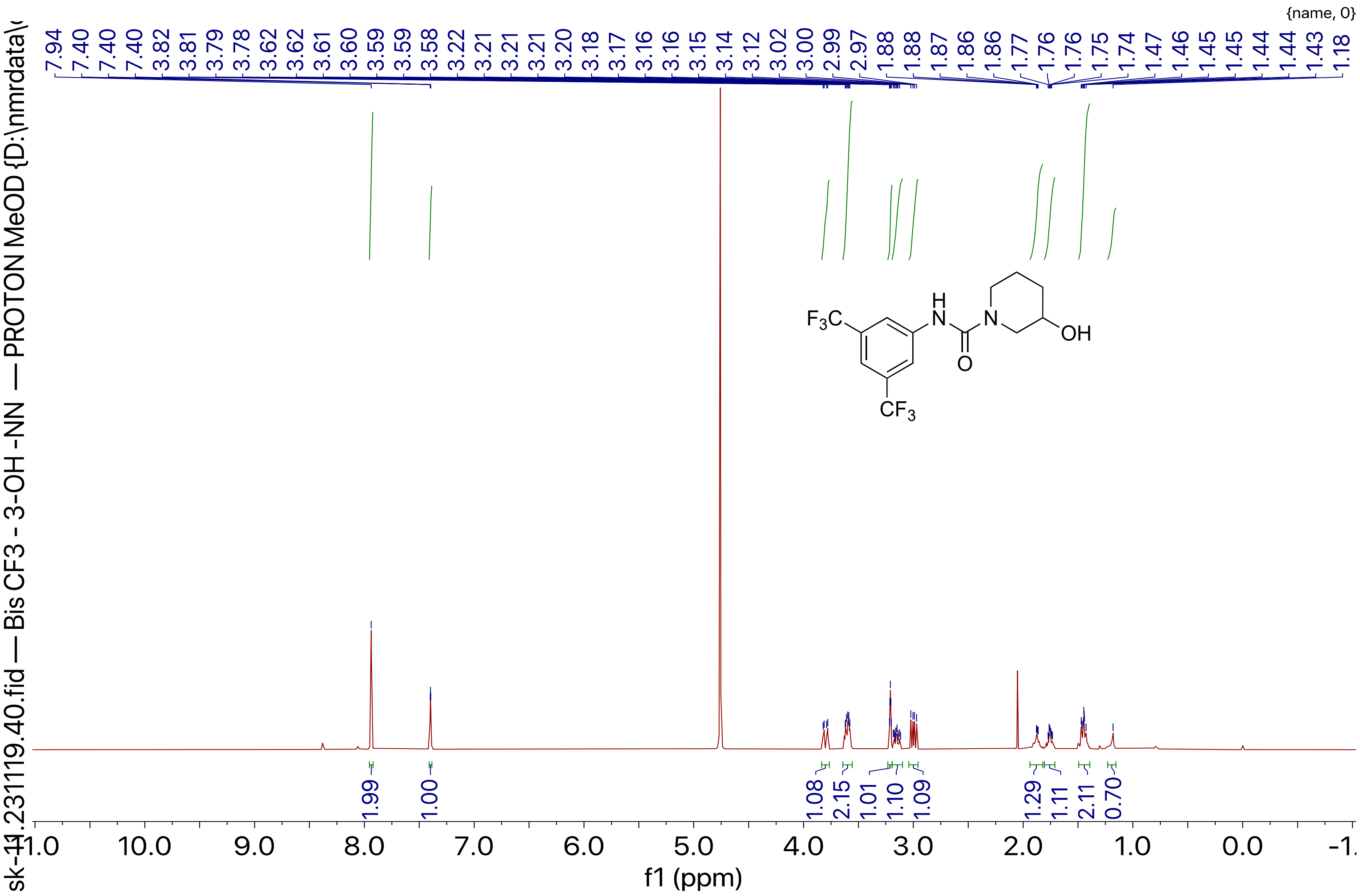

^{1}H NMR spectra of **5h’** (400 MHz, RT, CD_3OD)

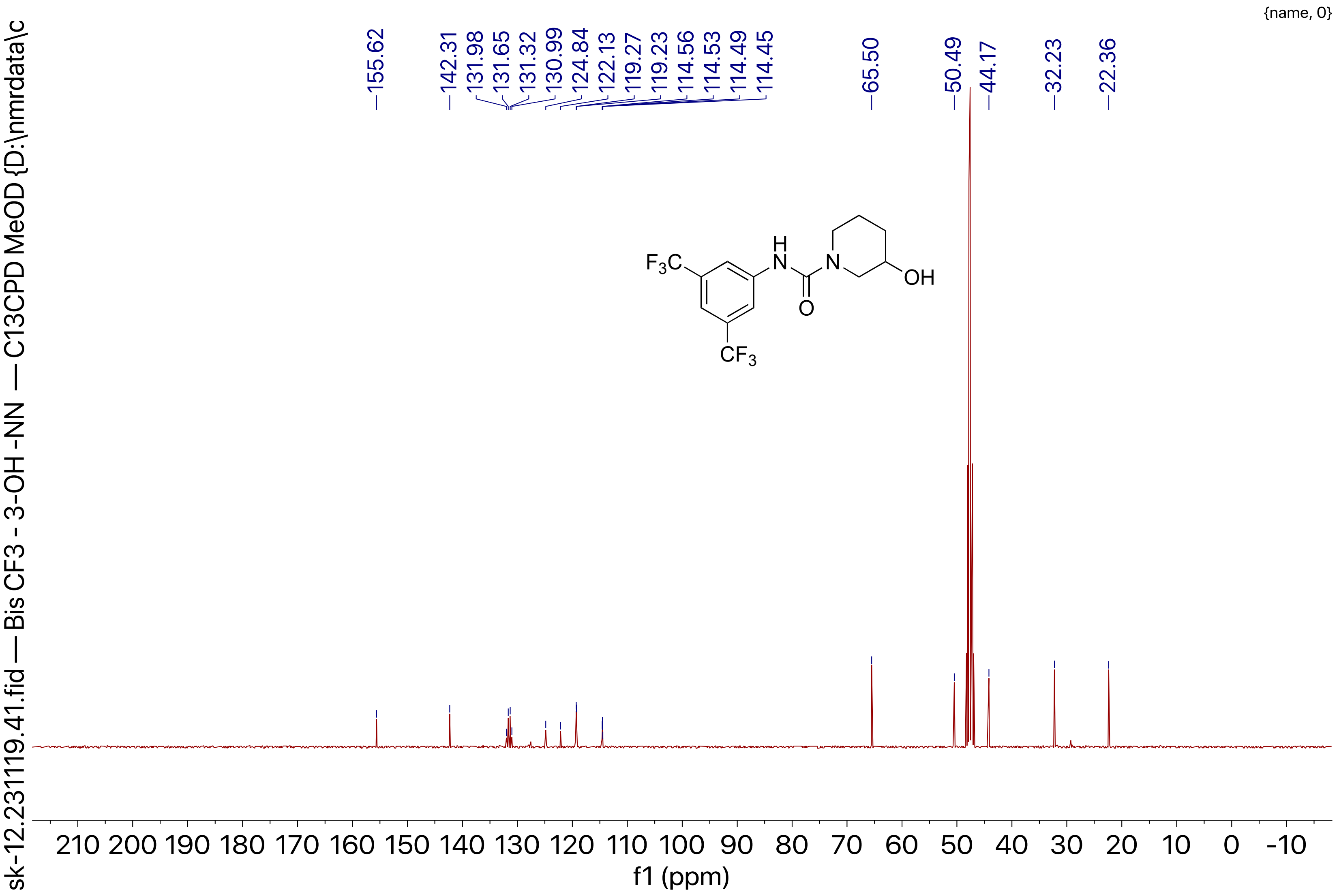

^{13}C NMR spectra of **5h'** (101 MHz, RT, CD_3OD)

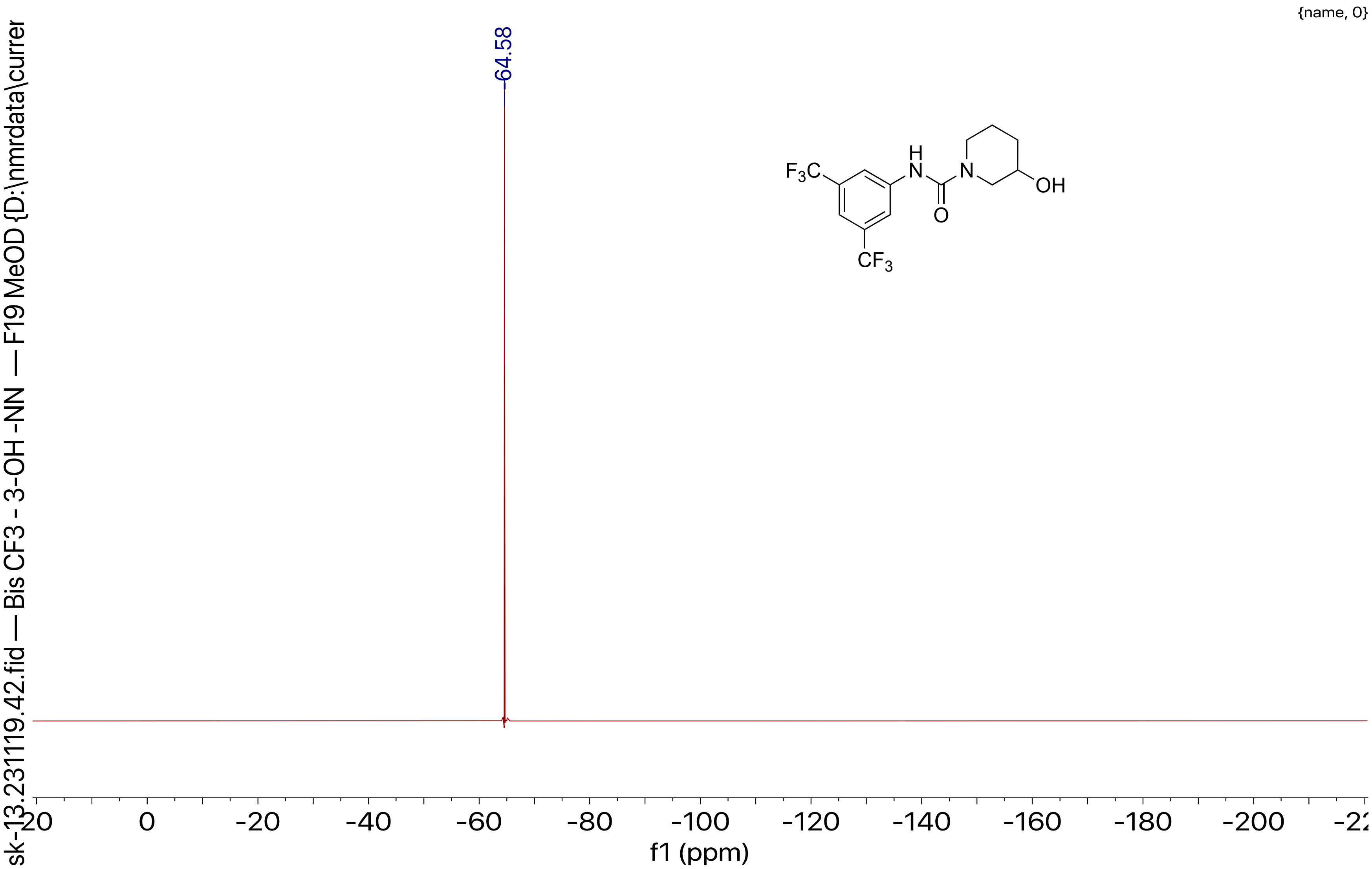

^{19}F NMR spectra of **5h’** (376 MHz, RT, CD_3OD)

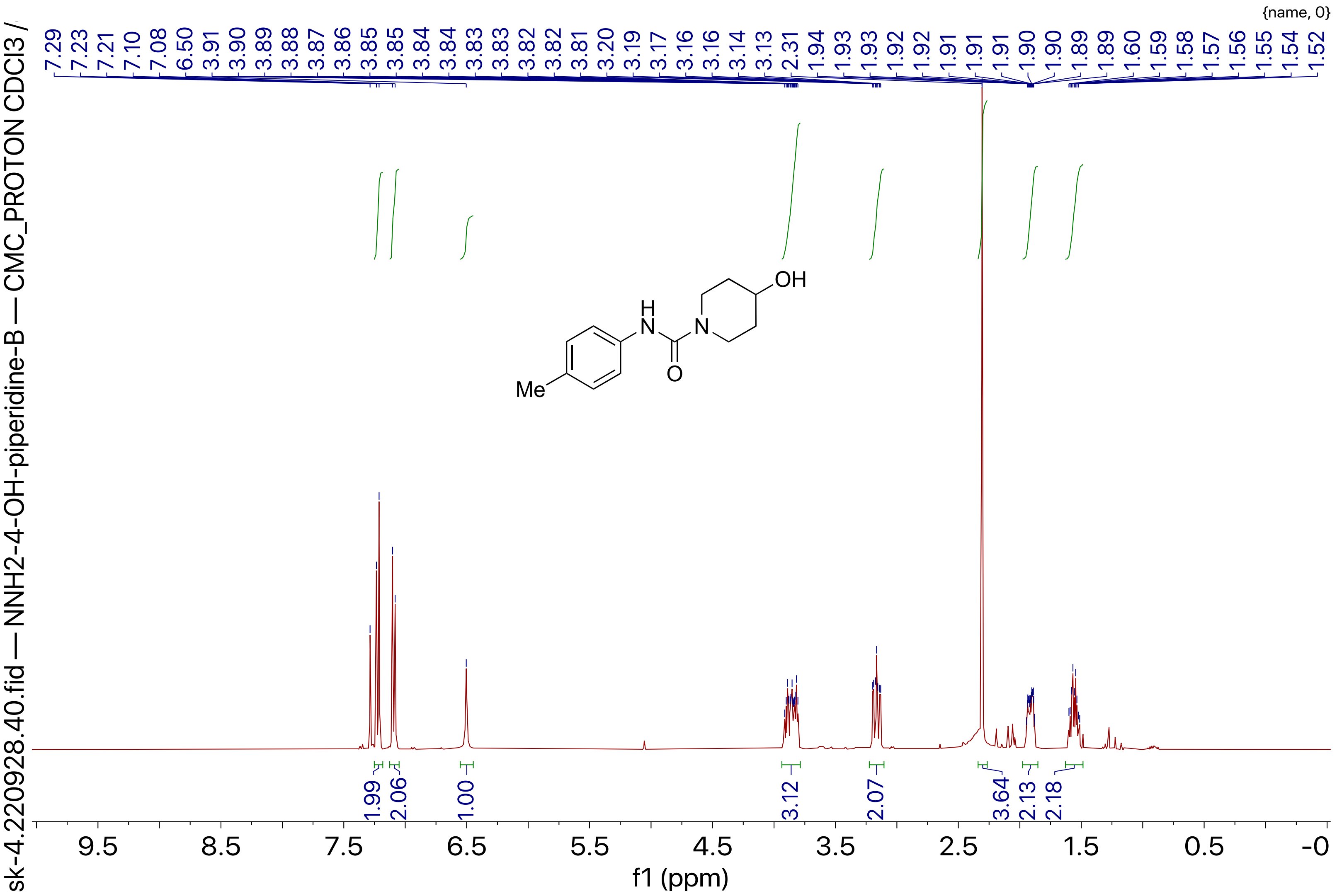

^{1}H NMR spectra of **5i** (400 MHz, RT, $CDCl_3$)

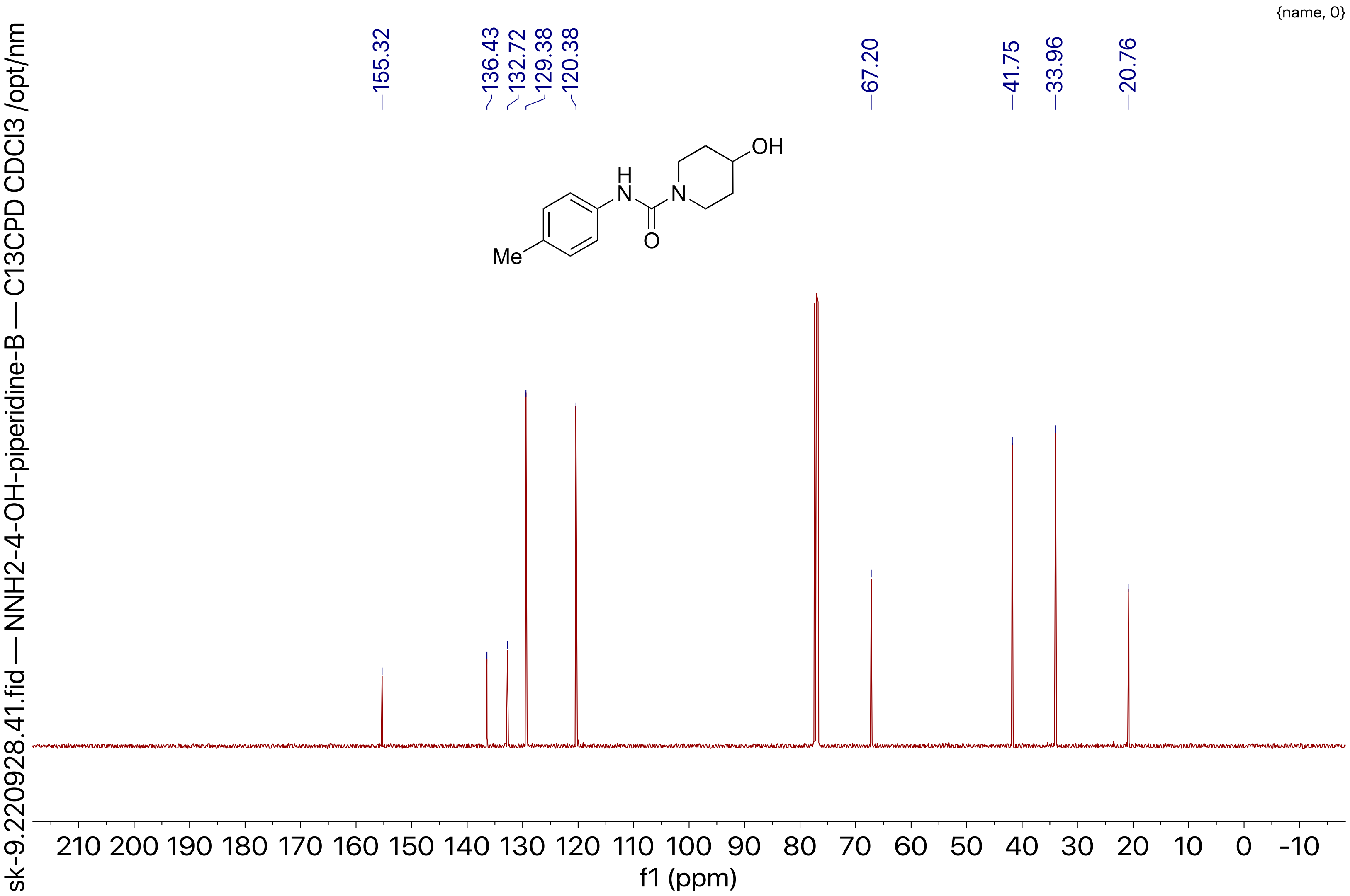

^{13}C NMR spectra of **5i** (101 MHz, RT, $CDCl_3$)

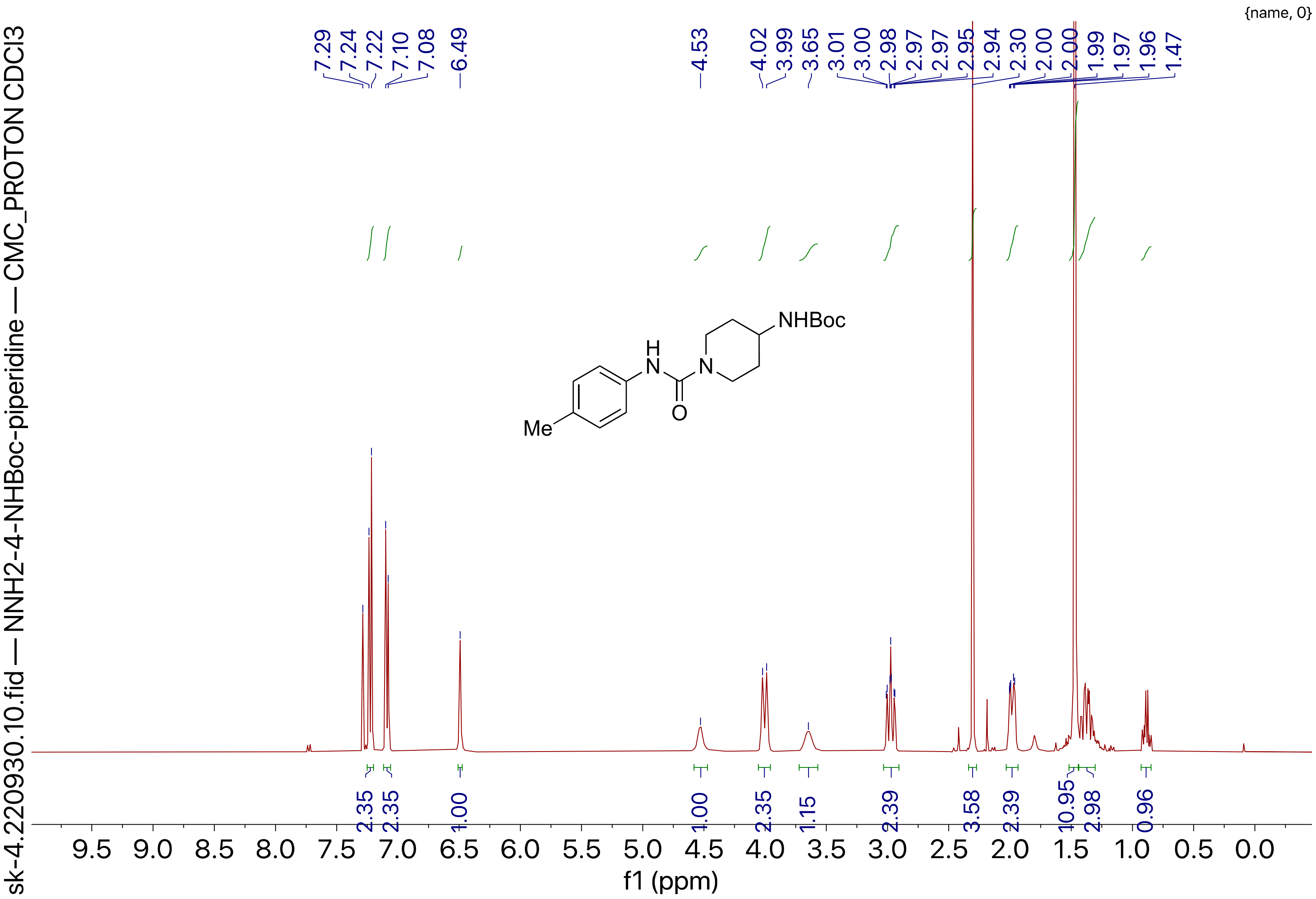

^{1}H NMR spectra of **5j** (400 MHz, RT, $CDCl_3$)

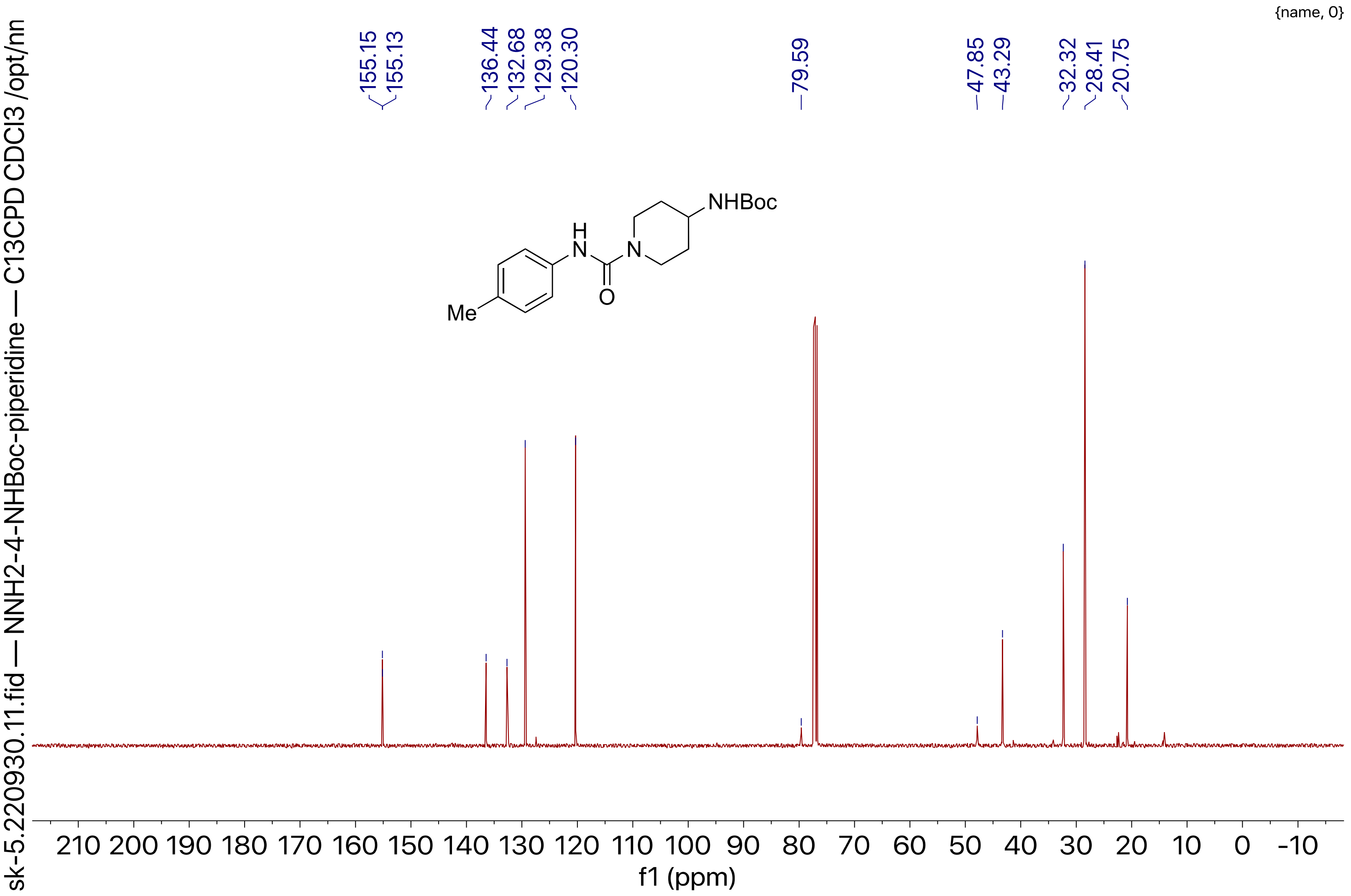

^{13}C NMR spectra of **5j** (101 MHz, RT, $CDCl_3$)

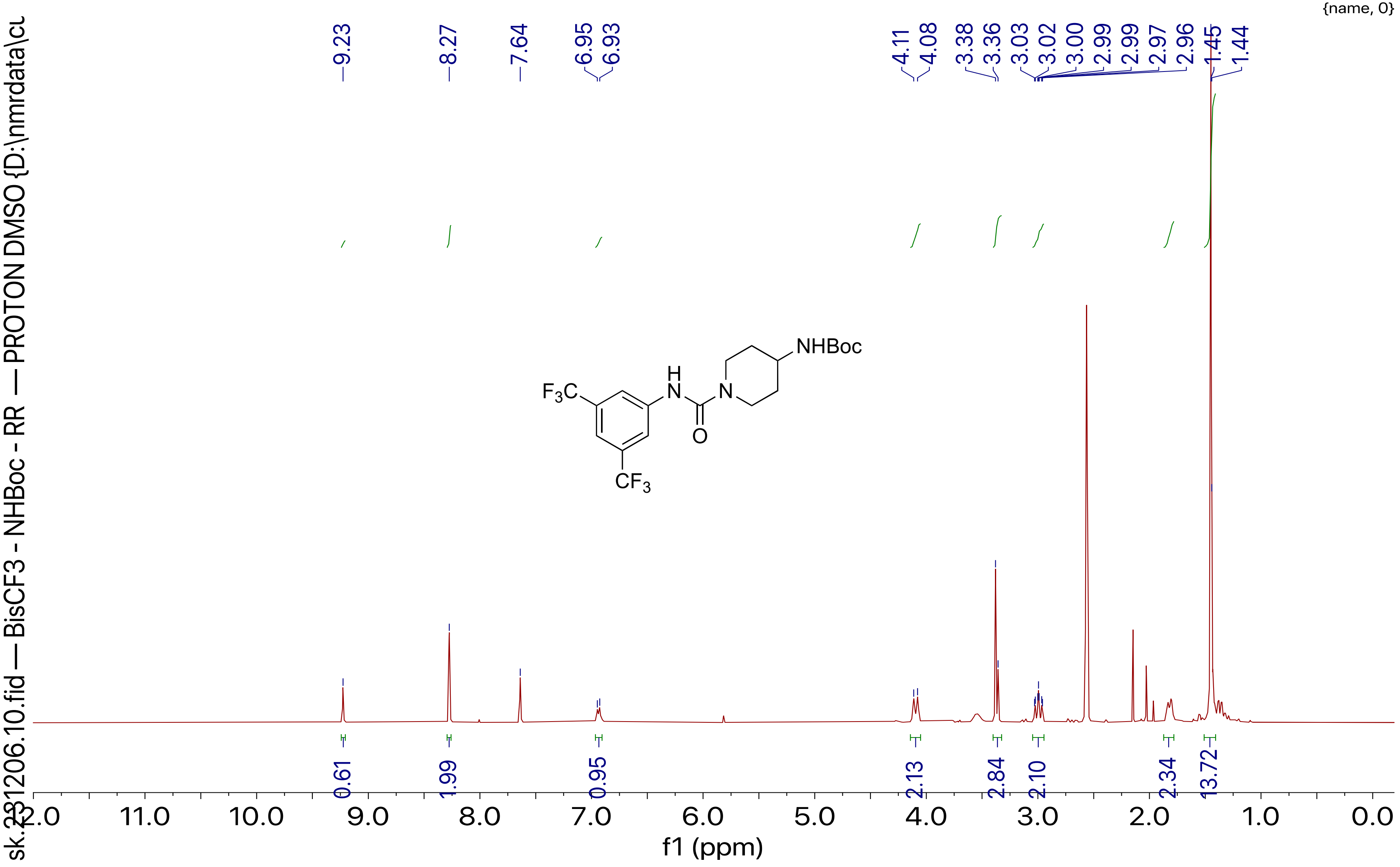

^{1}H NMR spectra of **5j’** (400 MHz, RT, $CDCl_3$)

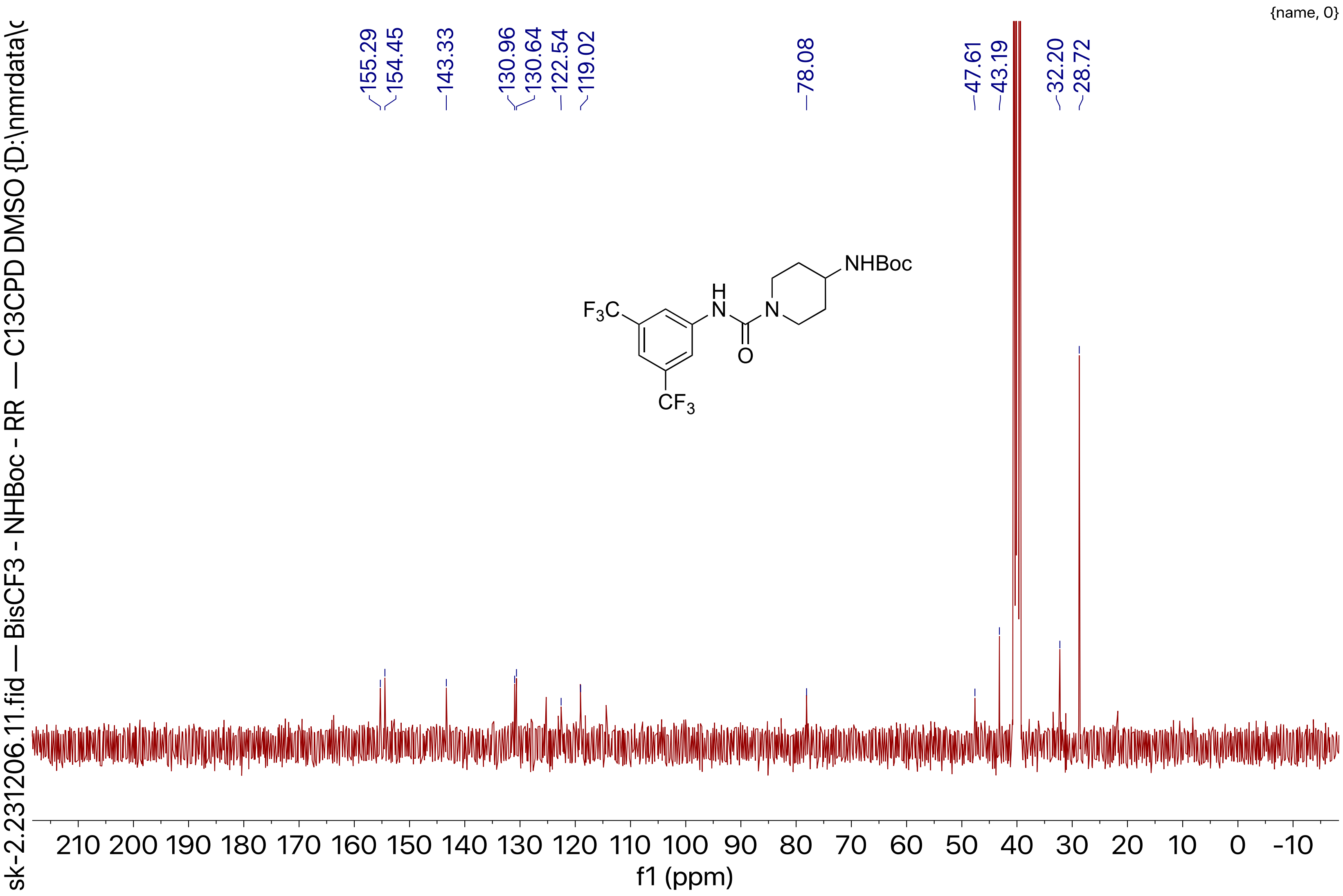

^{13}C NMR spectra of **5j'** (101 MHz, RT, $CDCl_3$)

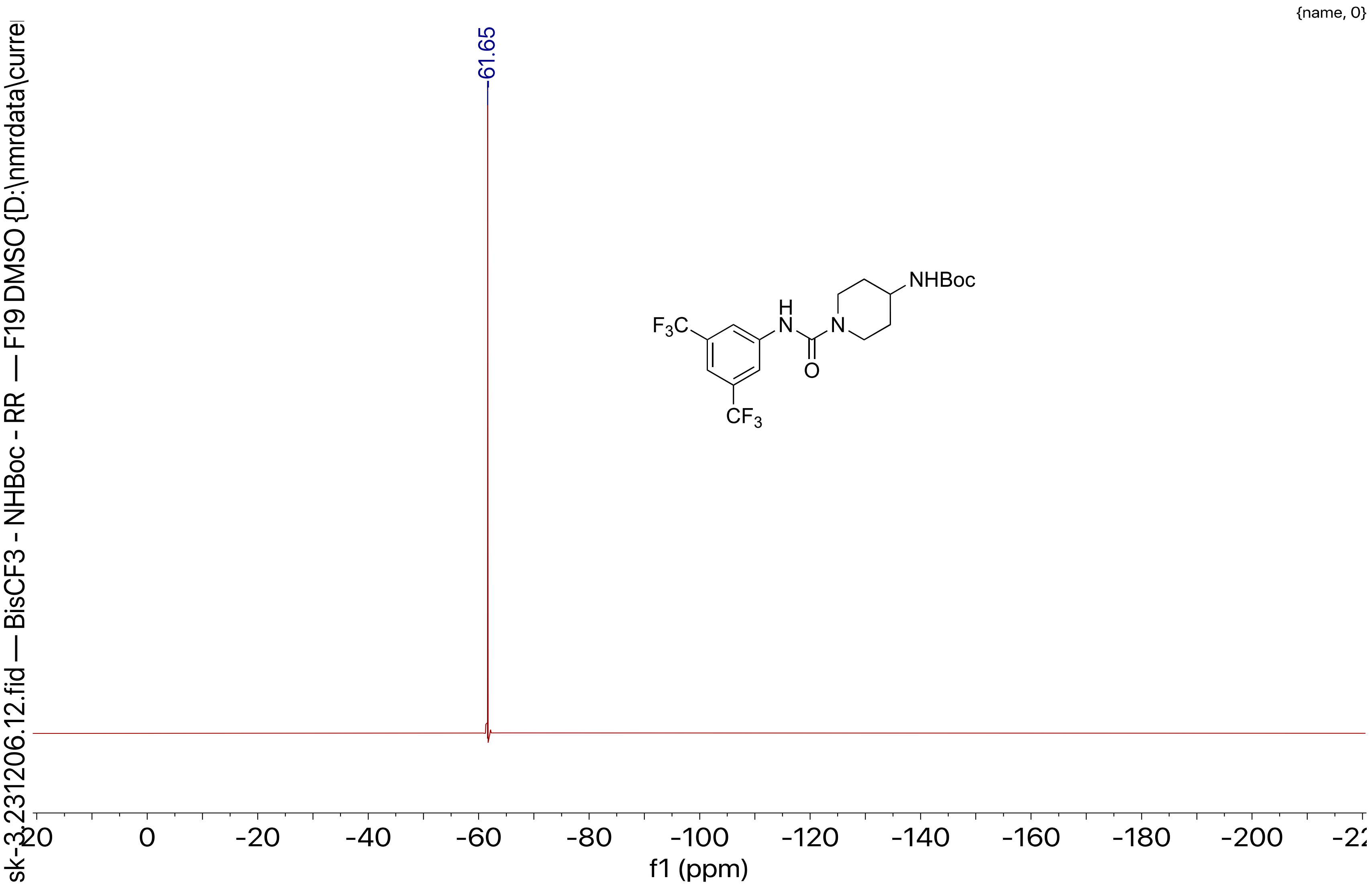

^{19}F NMR spectra of **5j’** (376 MHz, RT, $CDCl_3$)

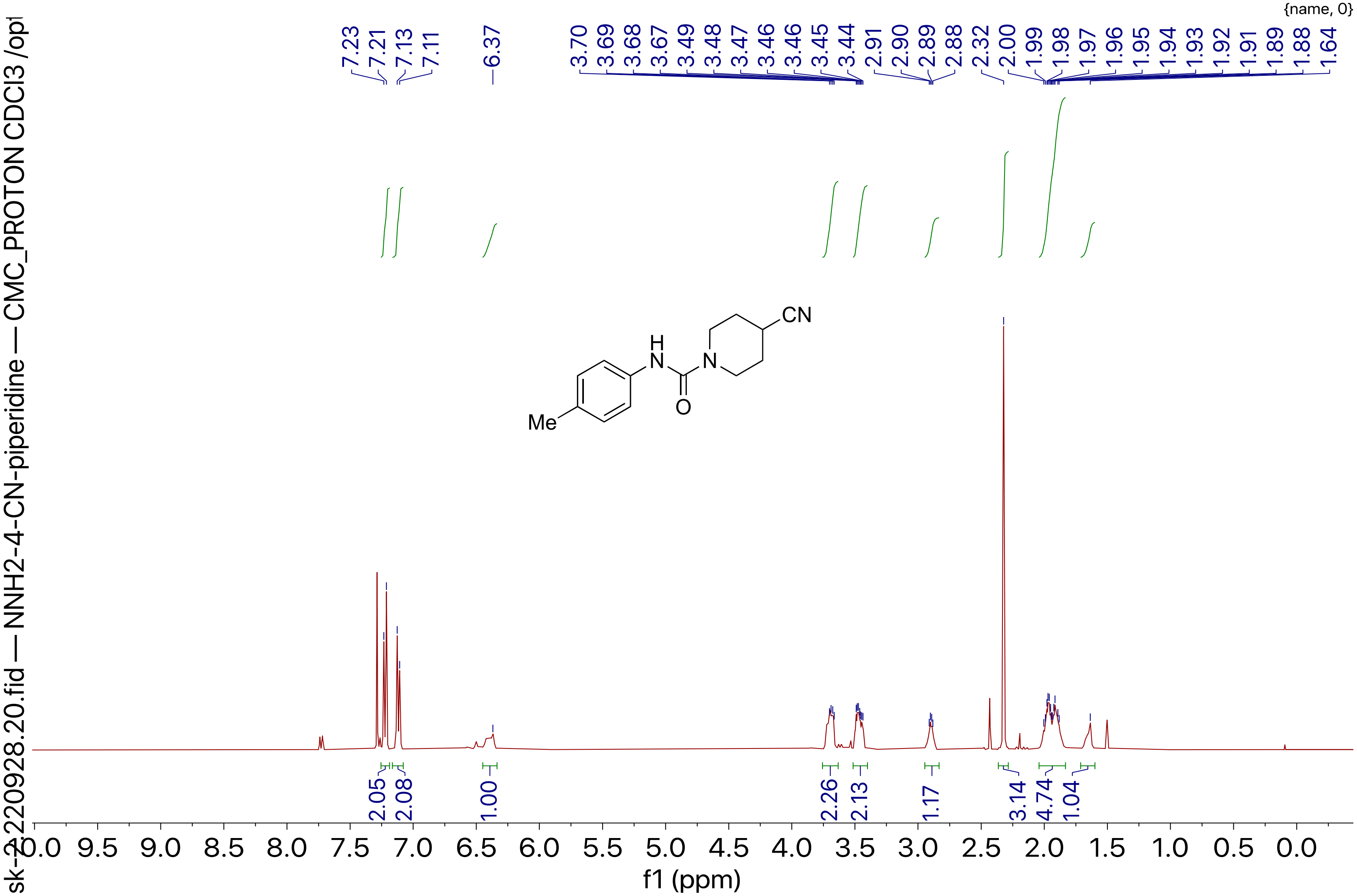

^{1}H NMR spectra of **5k** (400 MHz, RT, $CDCl_3$)

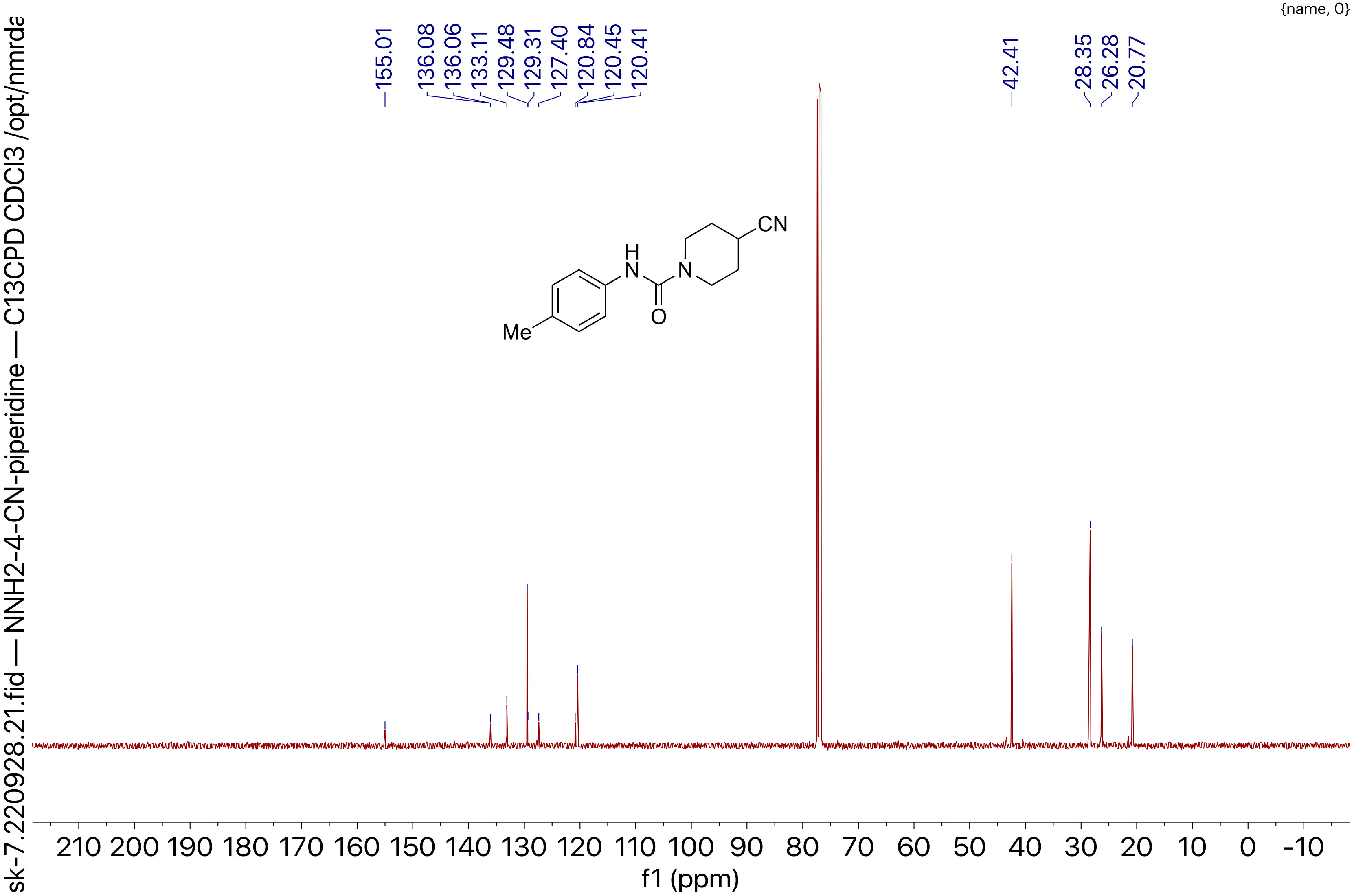

^{13}C NMR spectra of **5k** (100 MHz, RT, $CDCl_3$)

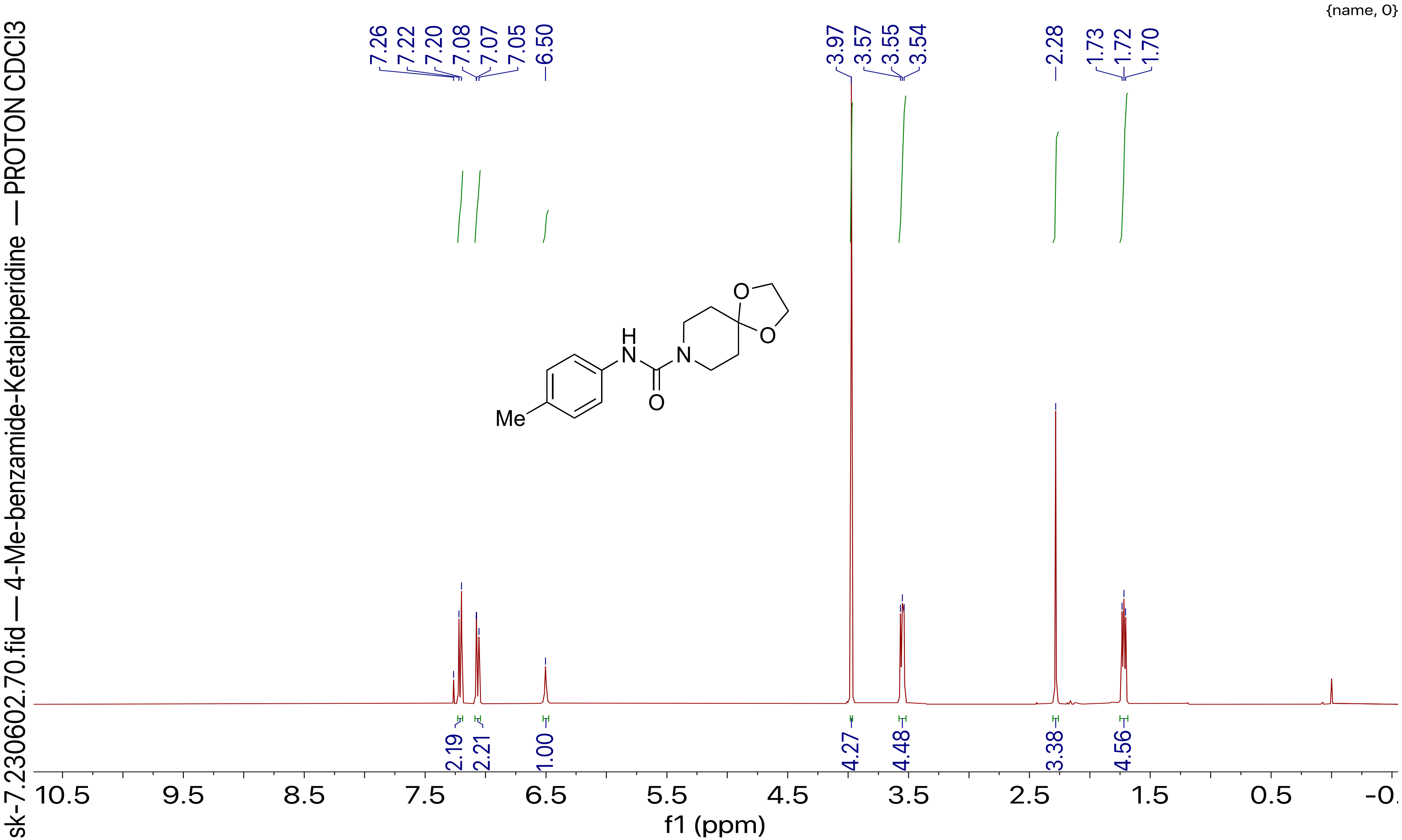

^{1}H NMR spectra of **5l** (400 MHz, RT, $CDCl_3$)

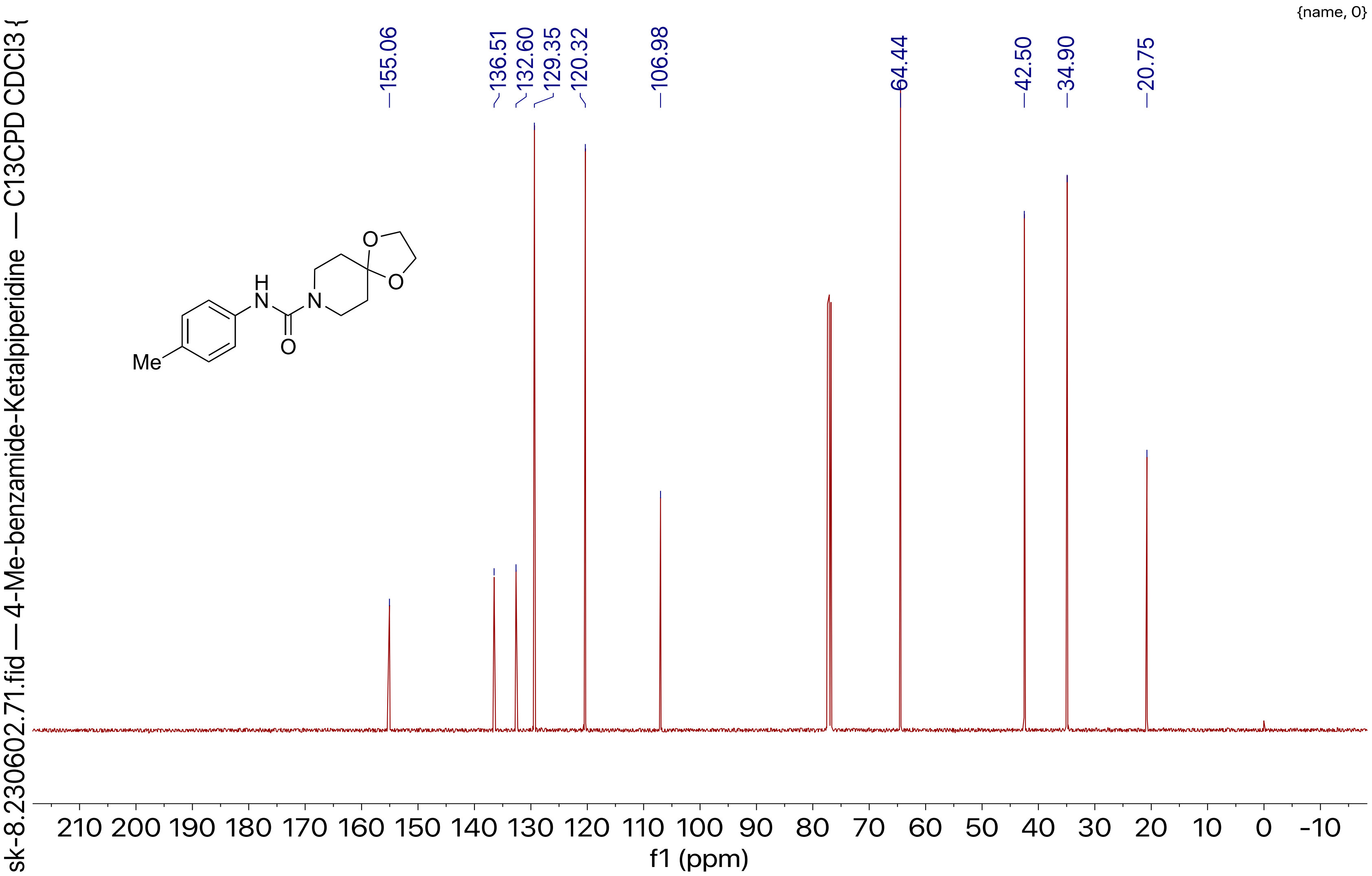

^{13}C NMR spectra of **5l** (101 MHz, RT, $CDCl_3$)

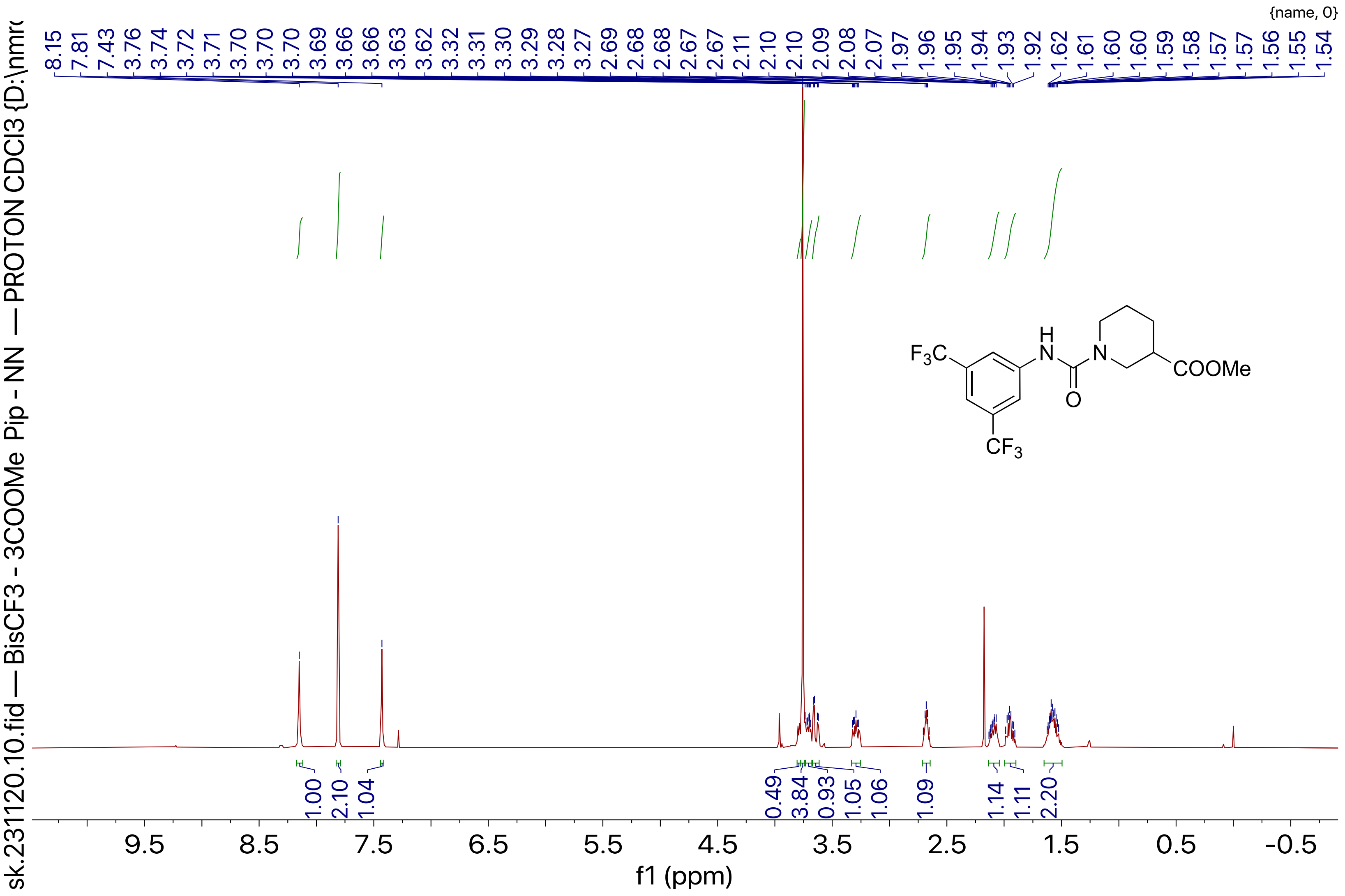

^{1}H NMR spectra of **5m** (400 MHz, RT, $CDCl_3$)

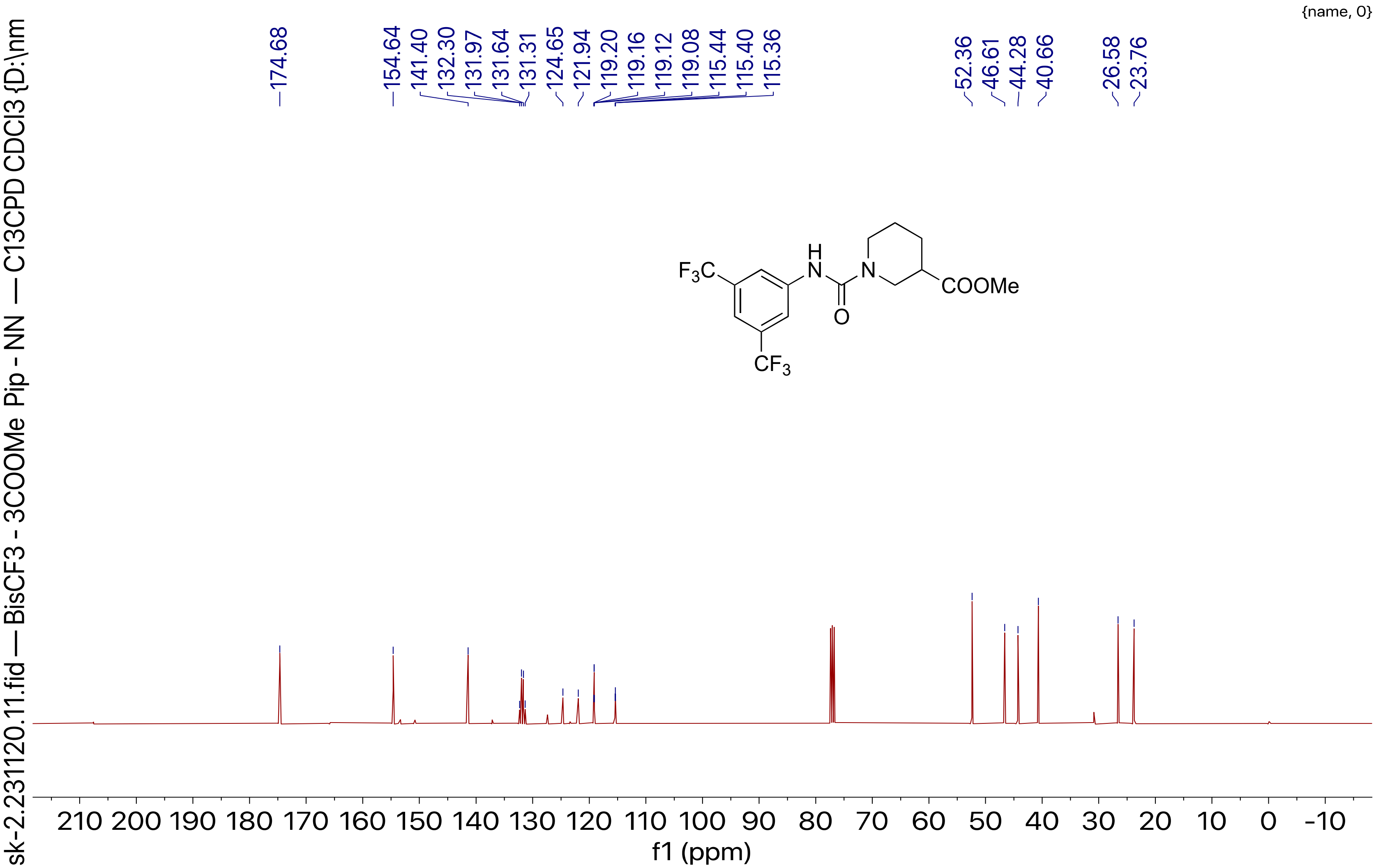

^{13}C NMR spectra of **5m** (101 MHz, RT, $CDCl_3$)

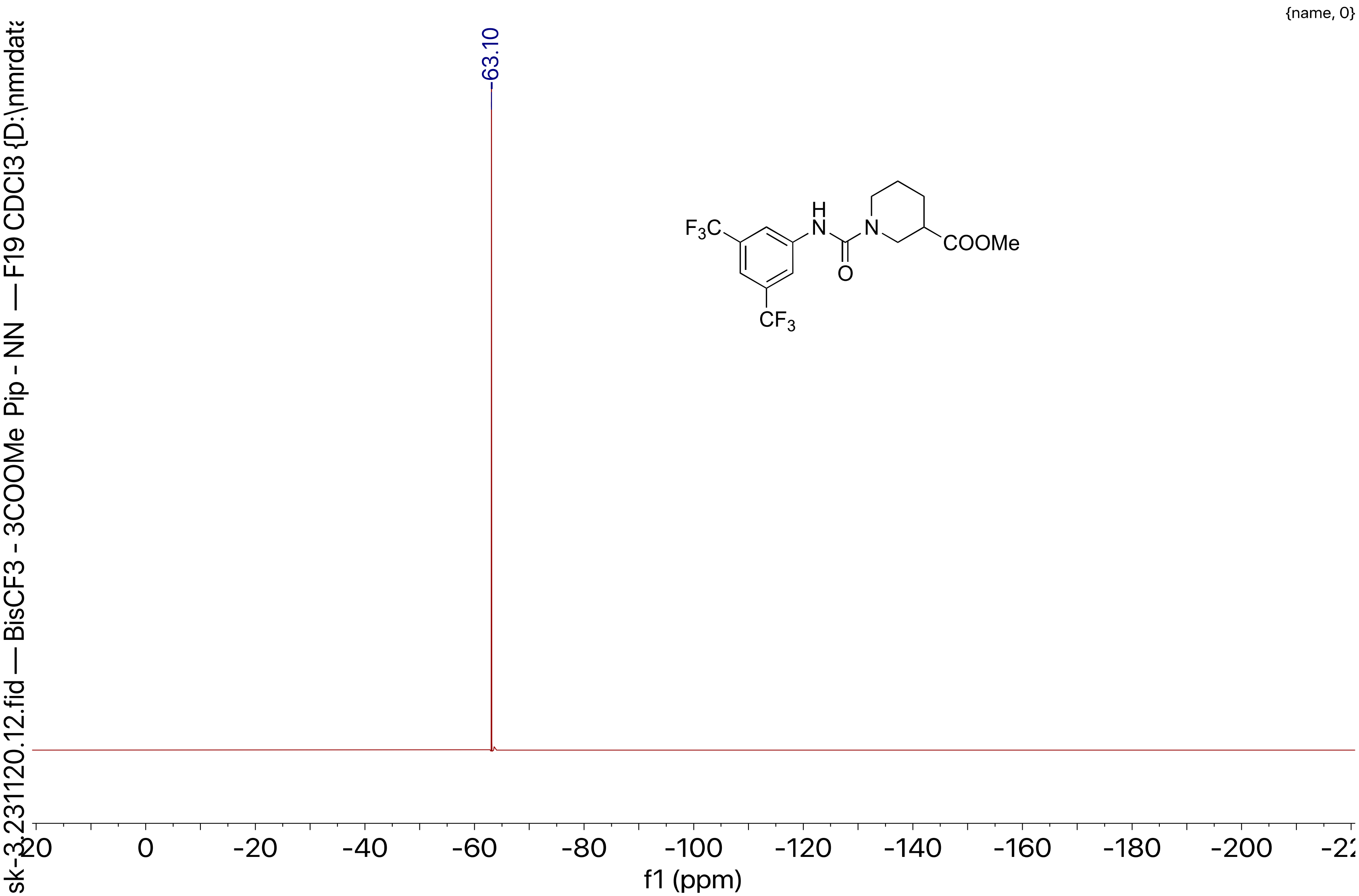

^{19}F NMR spectra of **5m** (376 MHz, RT, $CDCl_3$)

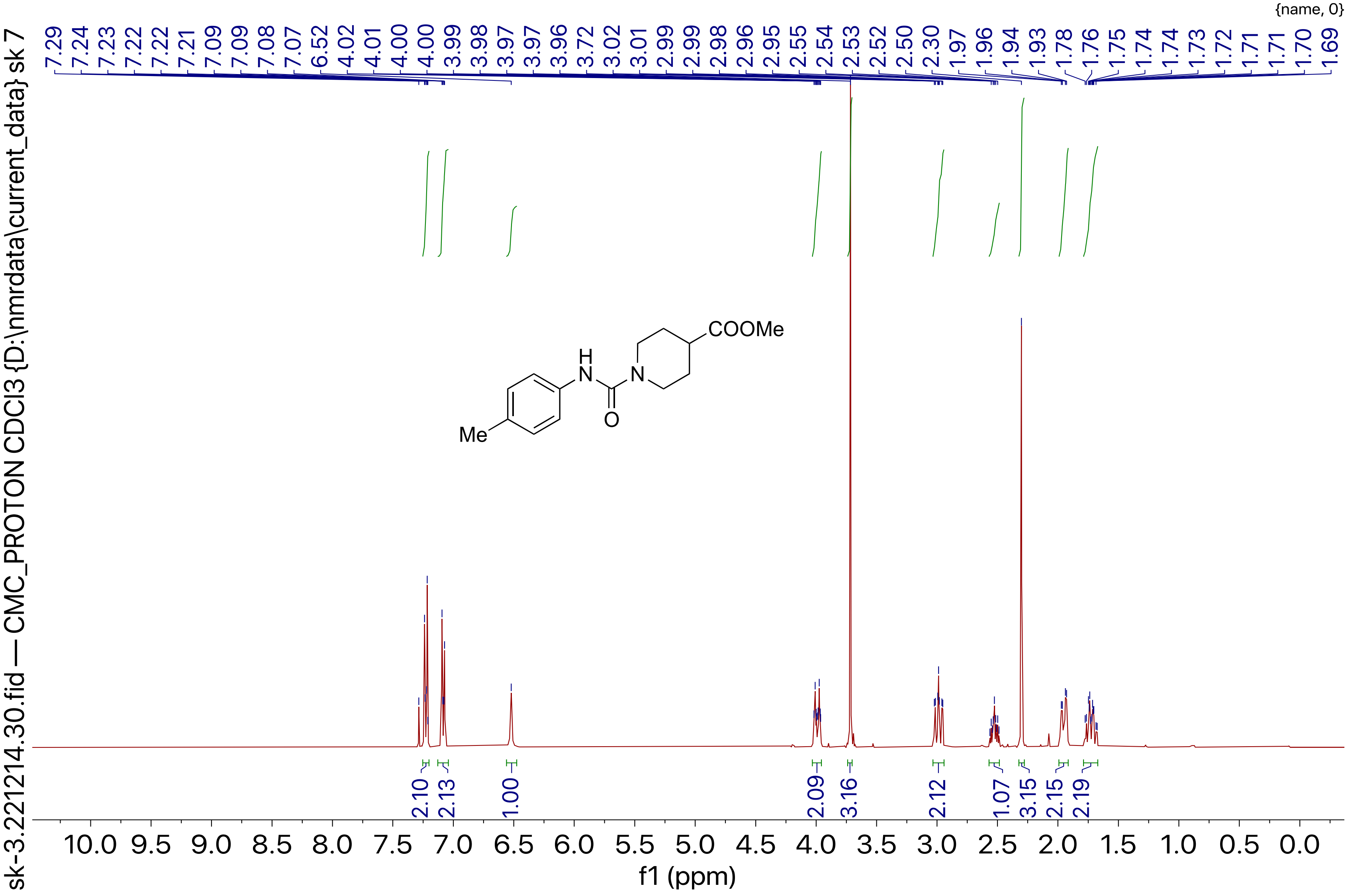
sk-3.221214.30.fid — CMC_PROTON CDCl3 {D:\nmrdata\current_data} sk 7
{name, 0}
7.29 7.24 7.23 7.22 7.22 7.21 7.09 7.09 7.08 7.07 6.52 4.02 4.01 4.00 4.00 3.99 3.98 3.97 3.97 3.96 3.72 3.02 3.01 2.99 2.99 2.98 2.96 2.95 2.55 2.54 2.53 2.52 2.50 2.30 1.97 1.96 1.94 1.93 1.78 1.76 1.75 1.74 1.74 1.73 1.72 1.71 1.71 1.70 1.69
COOMe
H
N
N
O
Me
2.10 2.13 1.00 2.09 3.16 2.12 1.07 3.15 2.15 2.19
10.0 9.5 9.0 8.5 8.0 7.5 7.0 6.5 6.0 5.5 5.0 4.5 4.0 3.5 3.0 2.5 2.0 1.5 1.0 0.5 0.0
f1 (ppm)

^{1}H NMR spectra of **5n** (400 MHz, RT, $CDCl_3$)

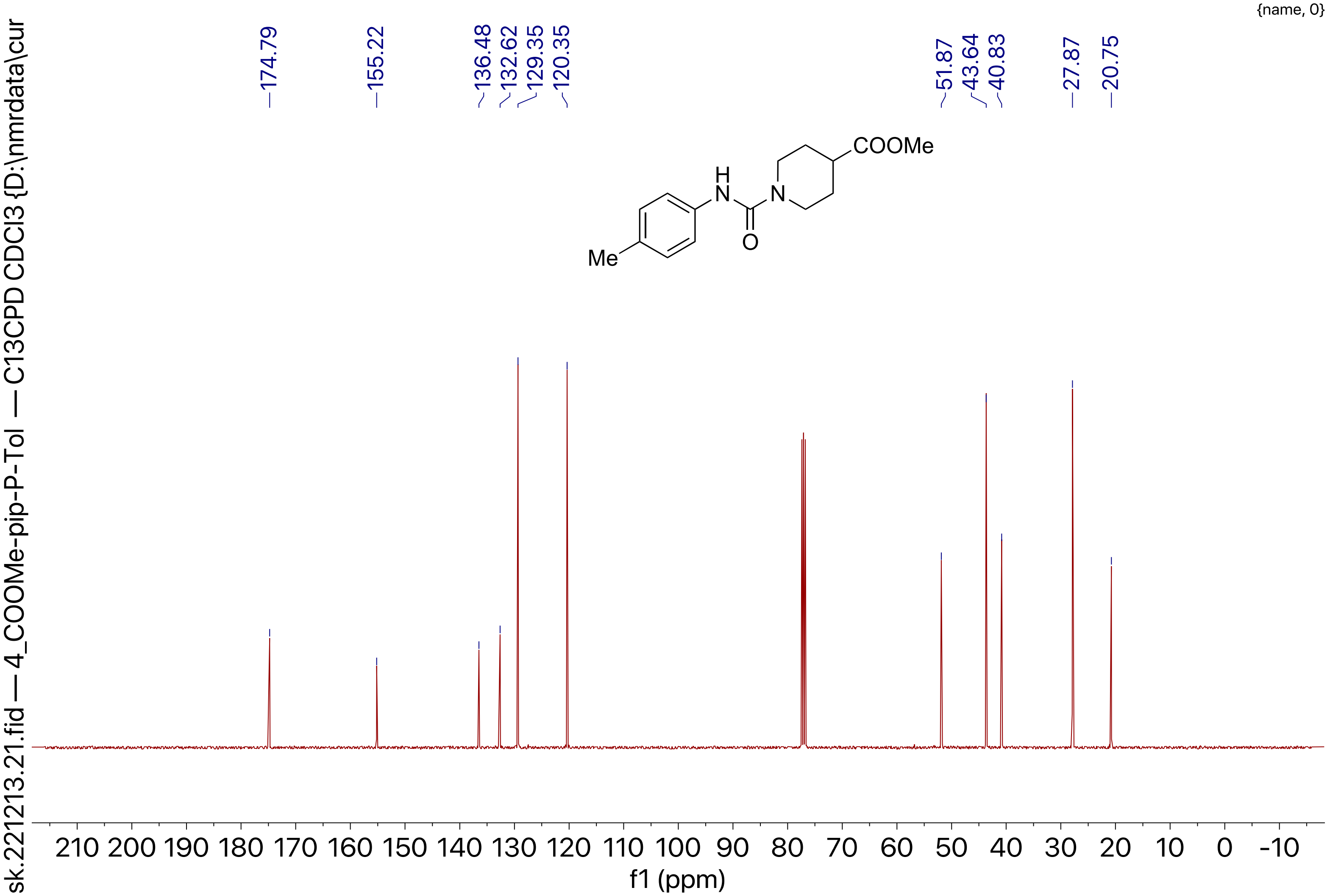

^{13}C NMR spectra of **5n** (101 MHz, RT, $CDCl_3$)

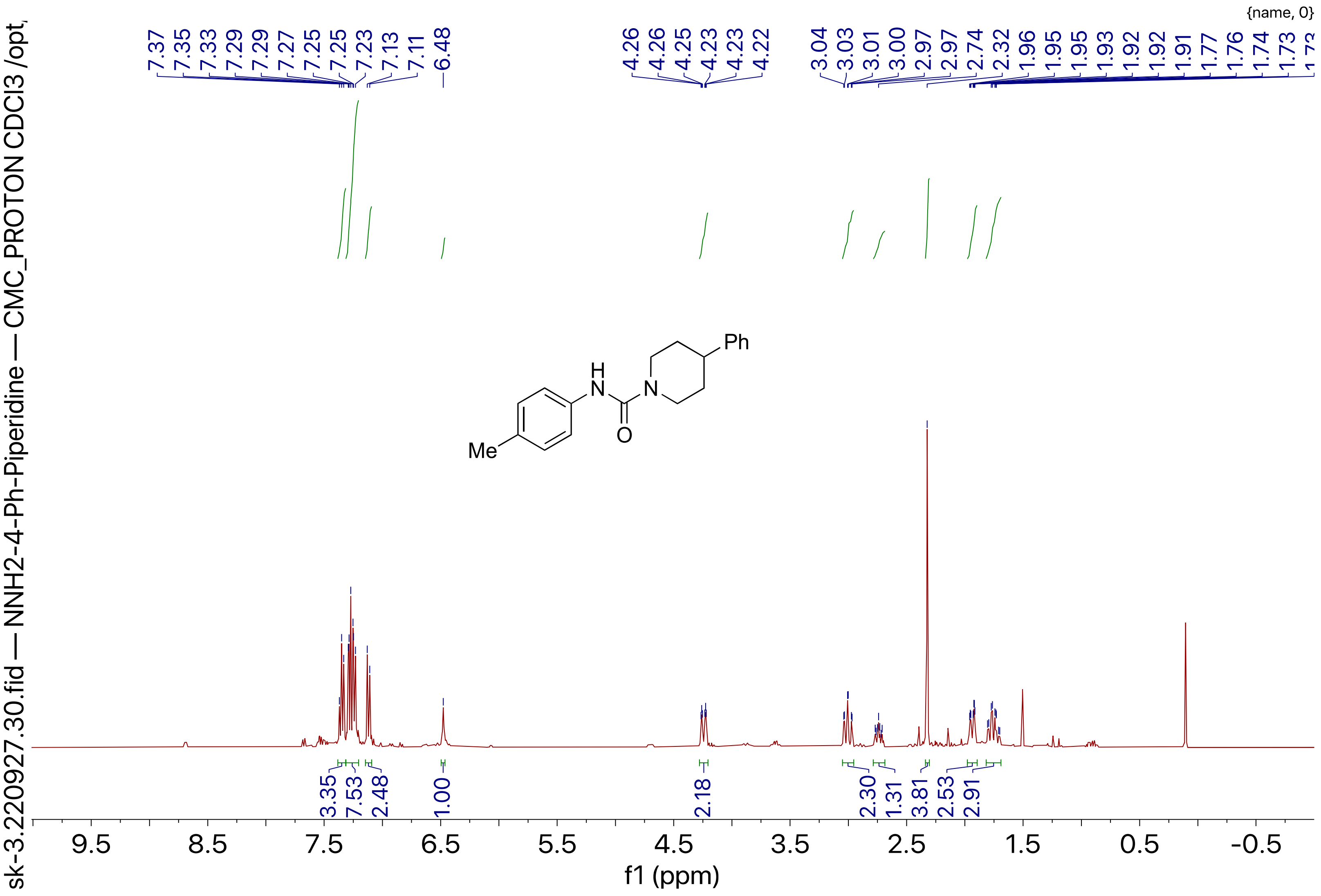

^{1}H NMR spectra of **5o** (400 MHz, RT, $CDCl_3$)

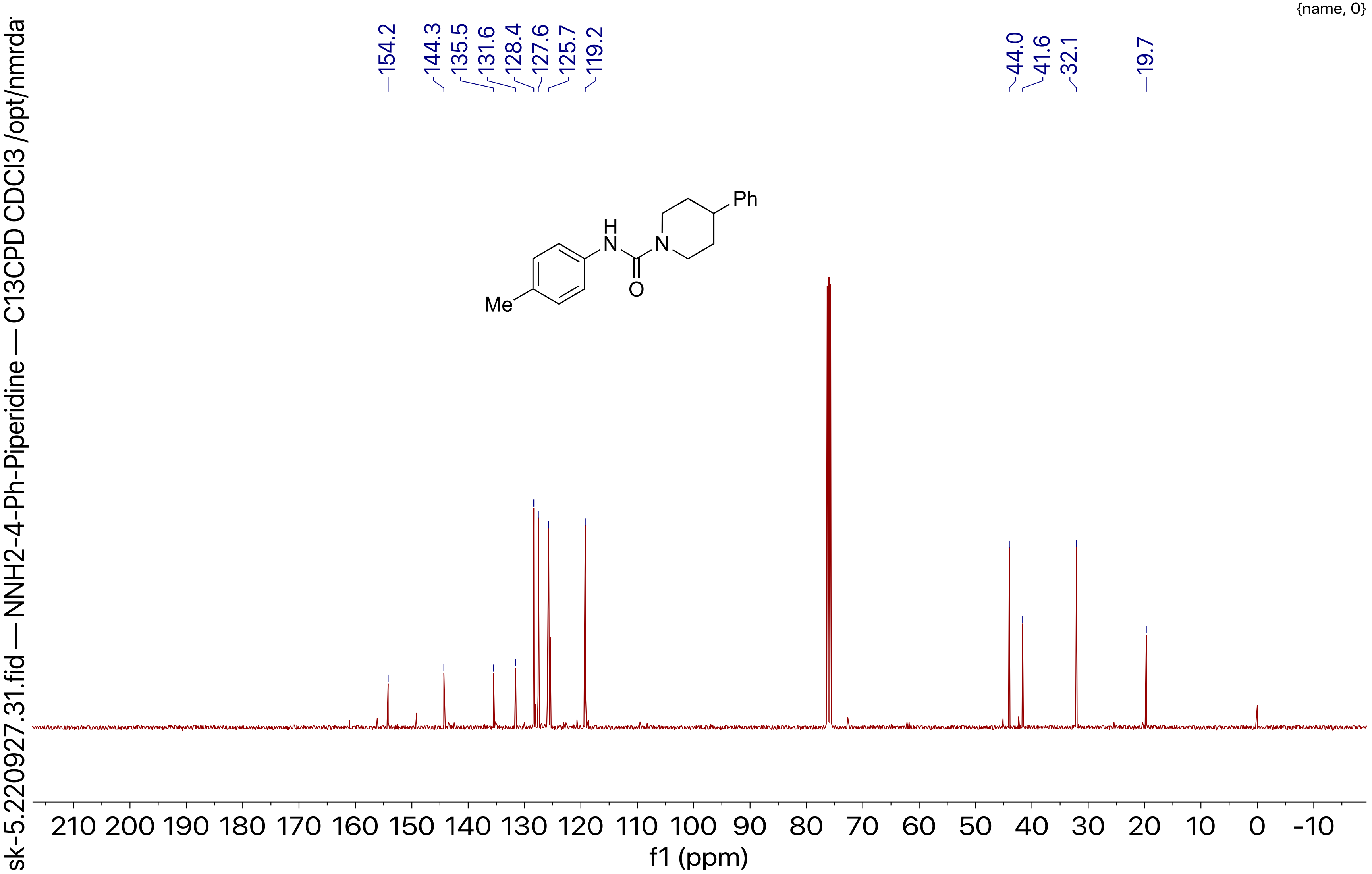

^{13}C NMR spectra of **5o** (101 MHz, RT, $CDCl_3$)

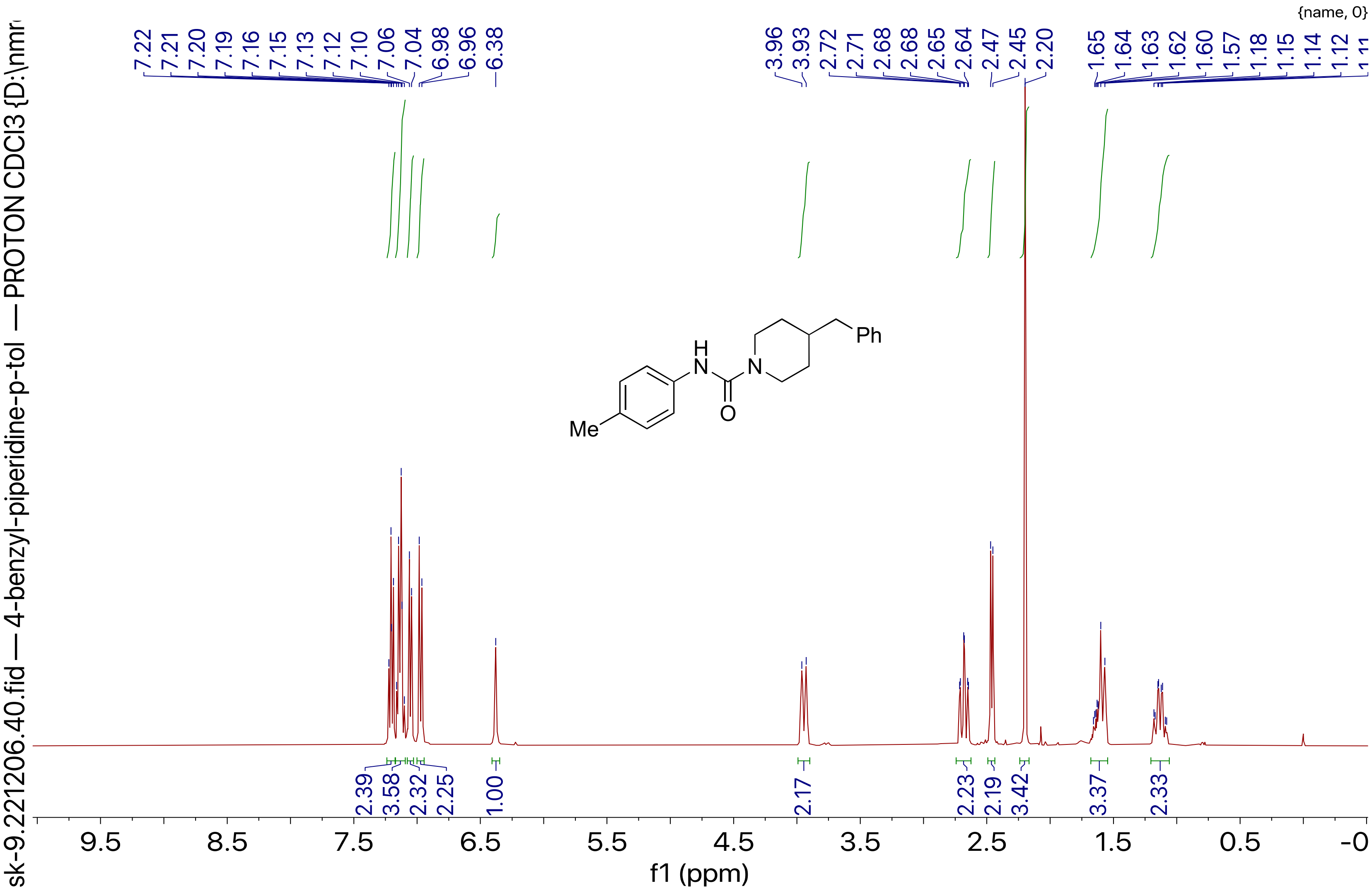

^{1}H NMR spectra of **5p** (400 MHz, RT, $CDCl_3$)

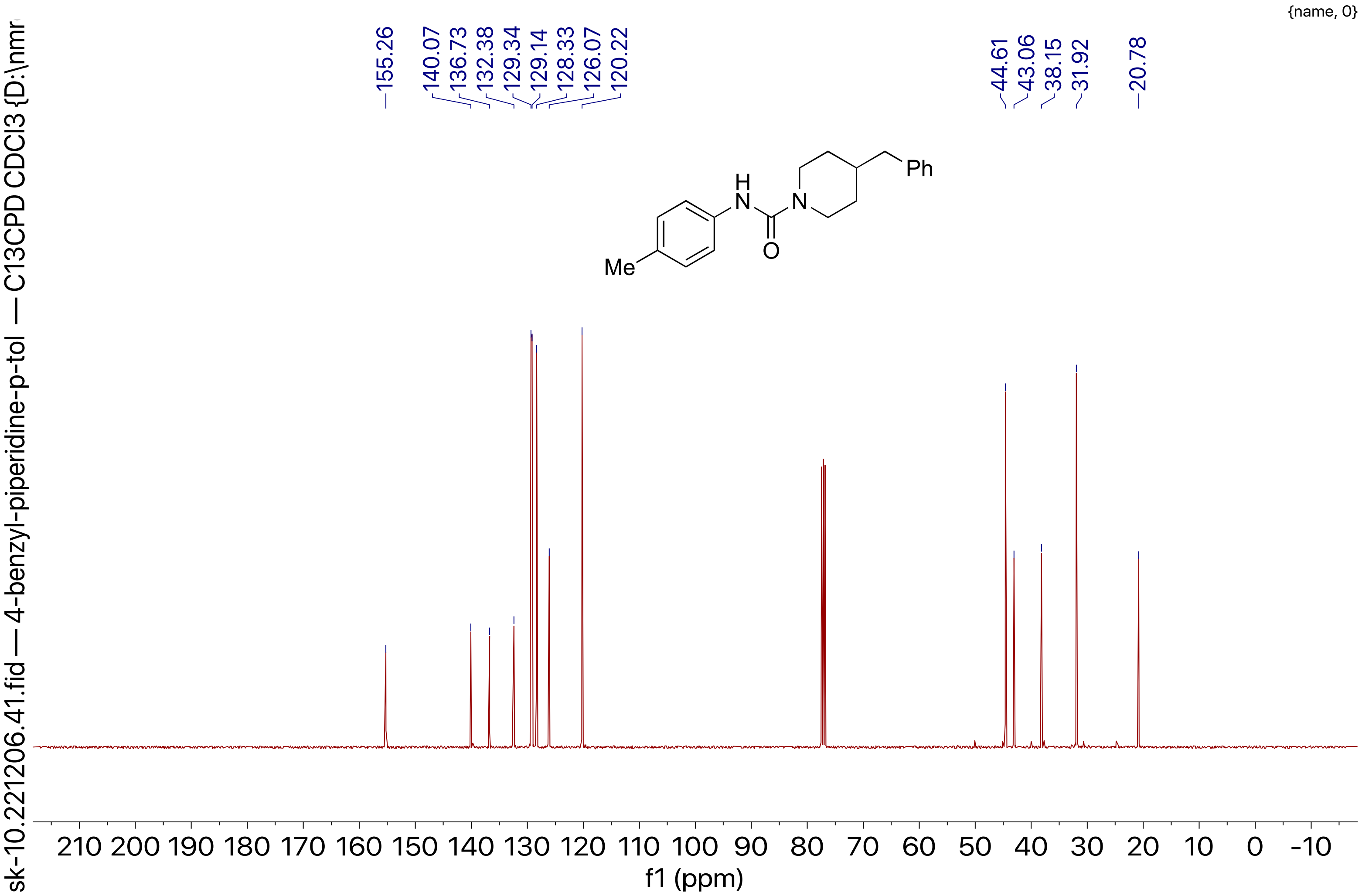

^{13}C NMR spectra of **5p** (101 MHz, RT, $CDCl_3$)

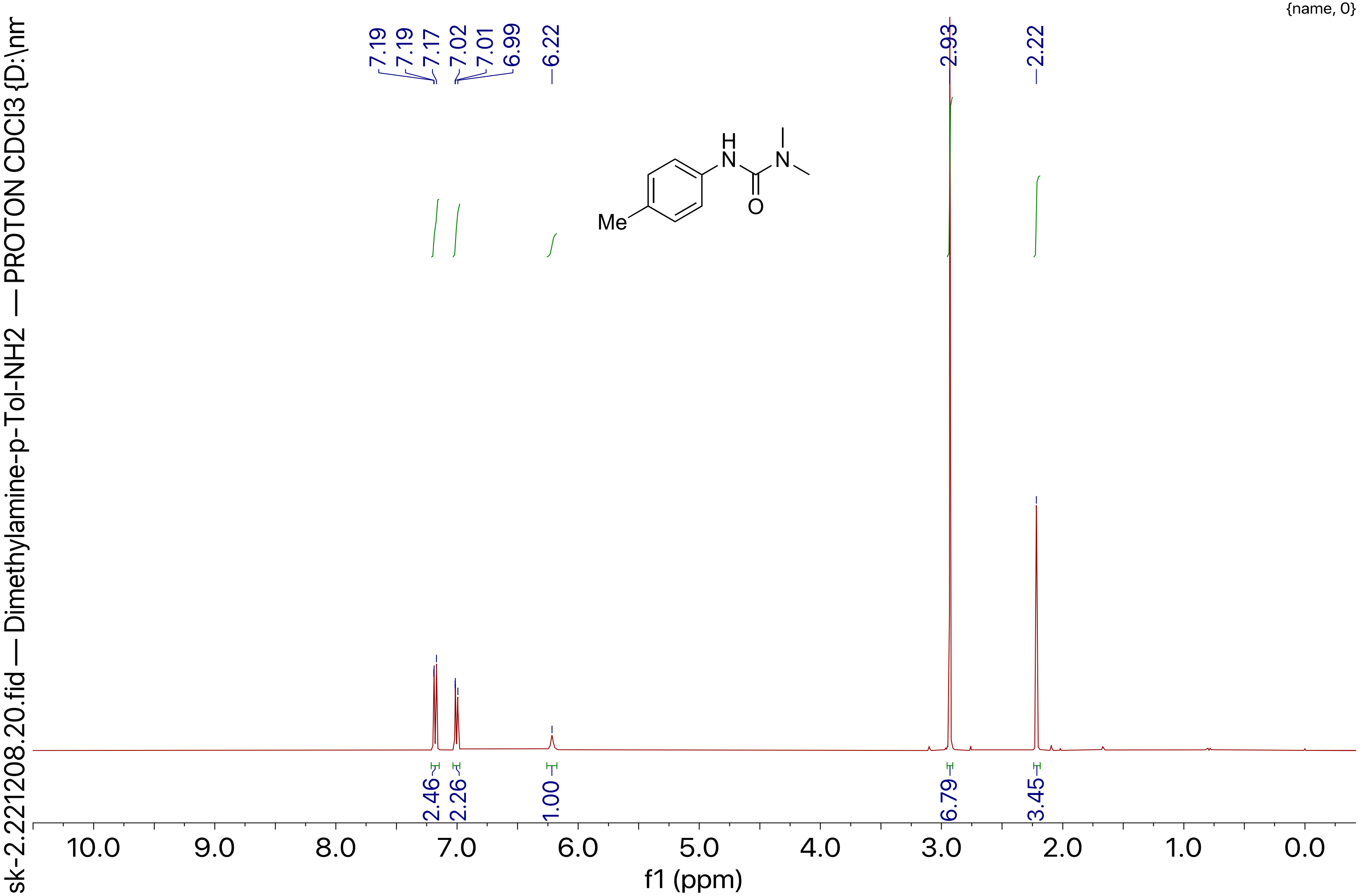

¹H NMR spectra of **5q** (400 MHz, RT, $CDCl_3$)

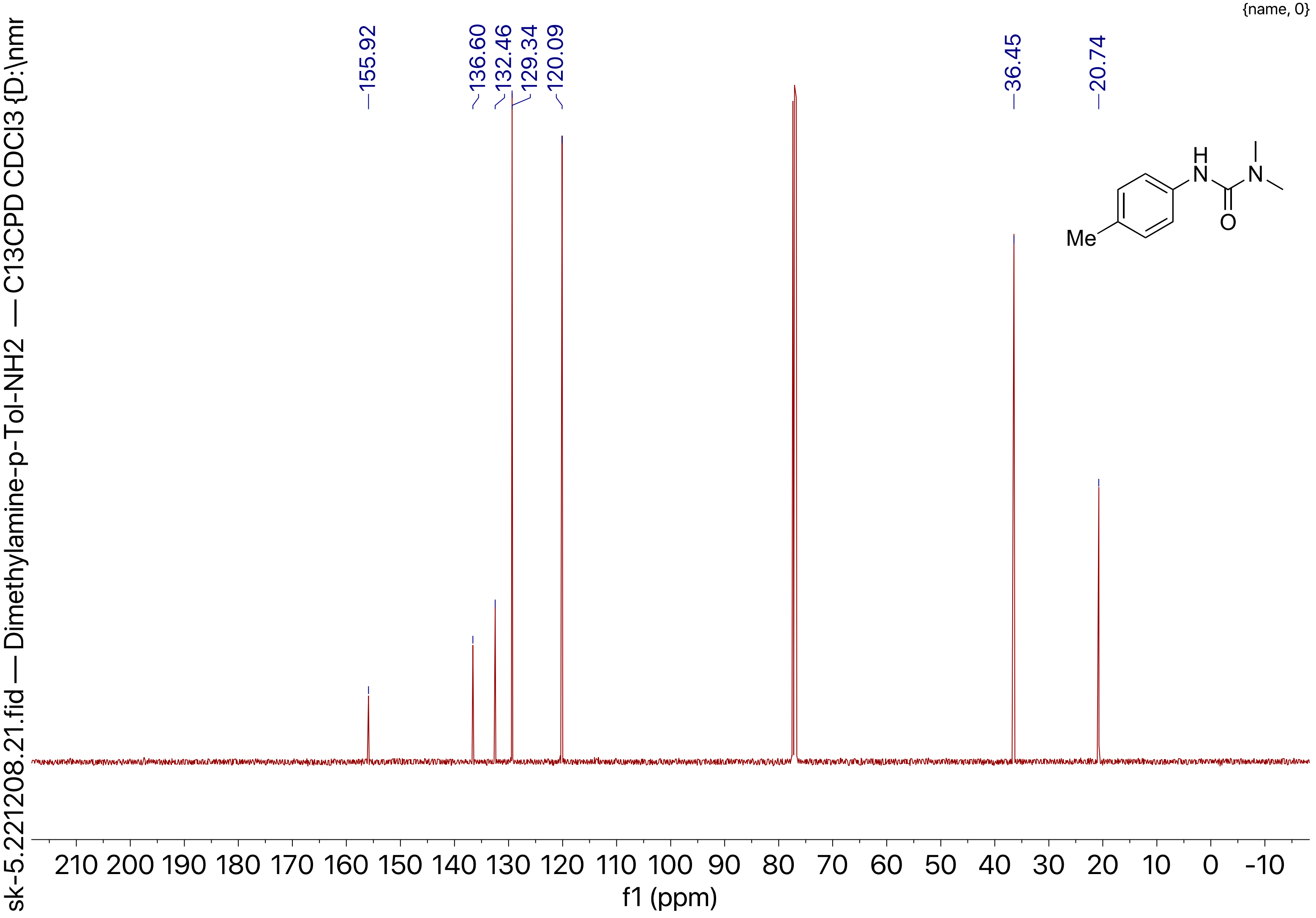

^{13}C NMR spectra of **5q** (101 MHz, RT, $CDCl_3$)

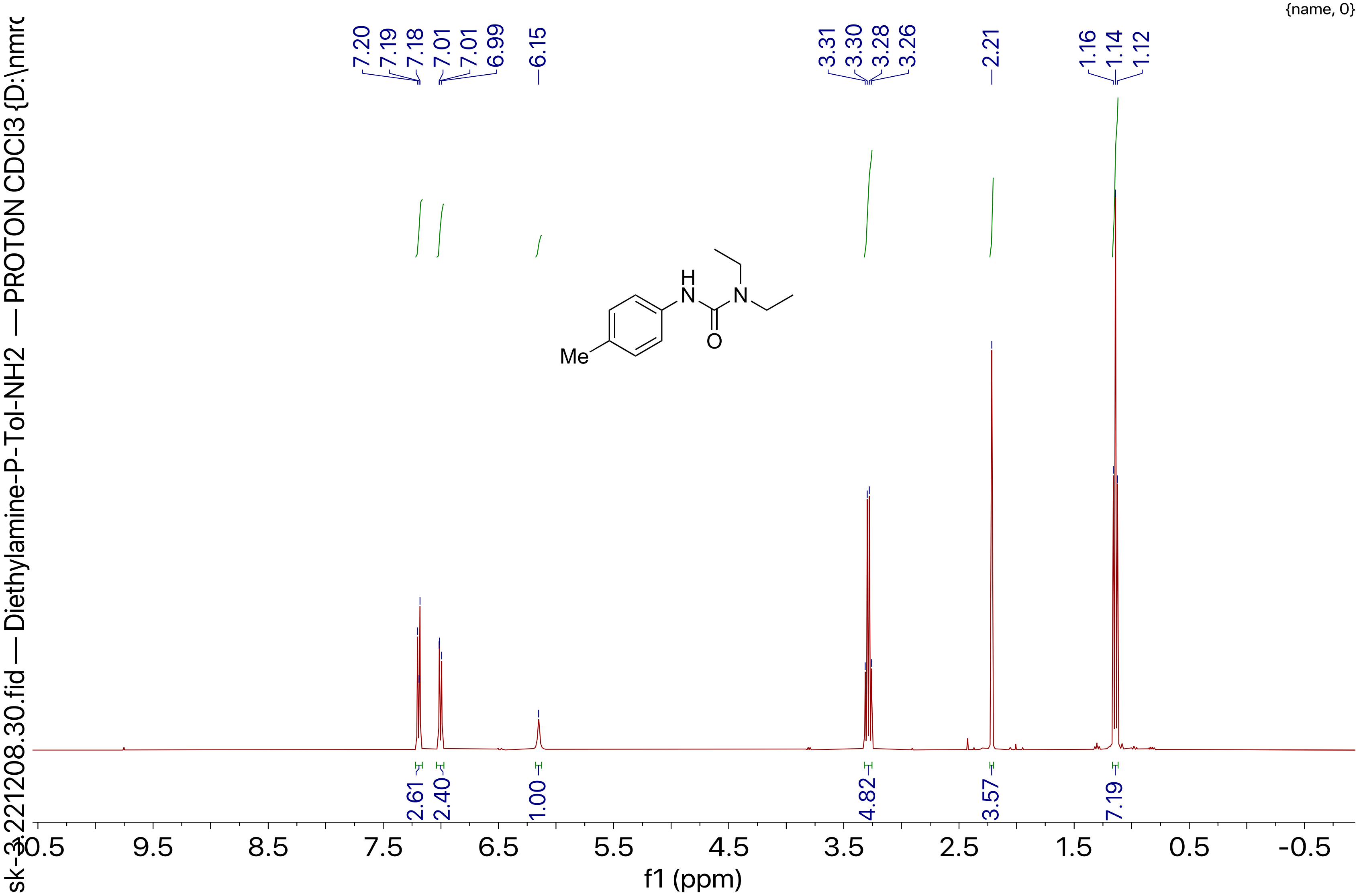

^{1}H NMR spectra of **5r** (400 MHz, RT, $CDCl_3$)

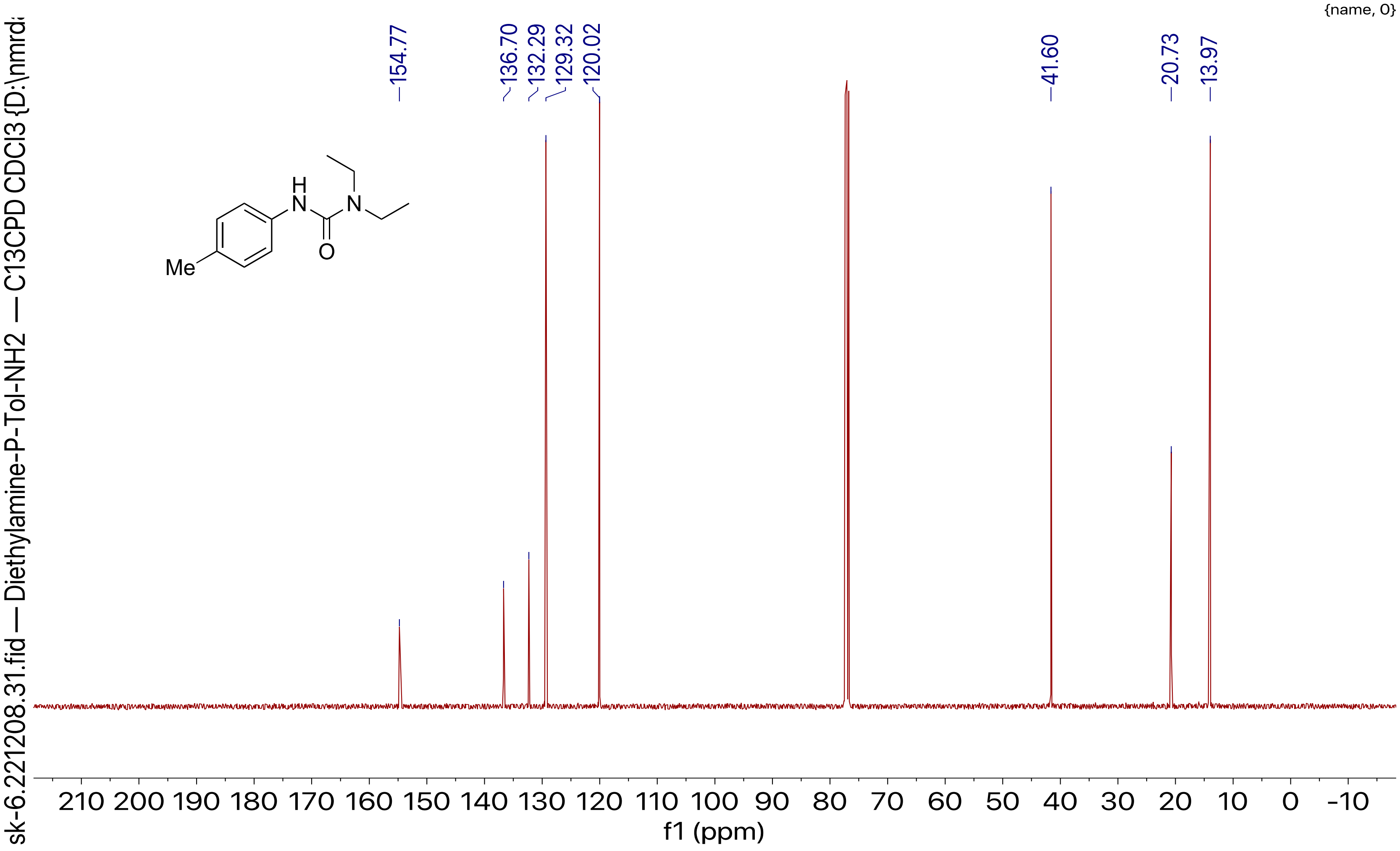

^{13}C NMR spectra of **5r** (101 MHz, RT, $CDCl_3$)

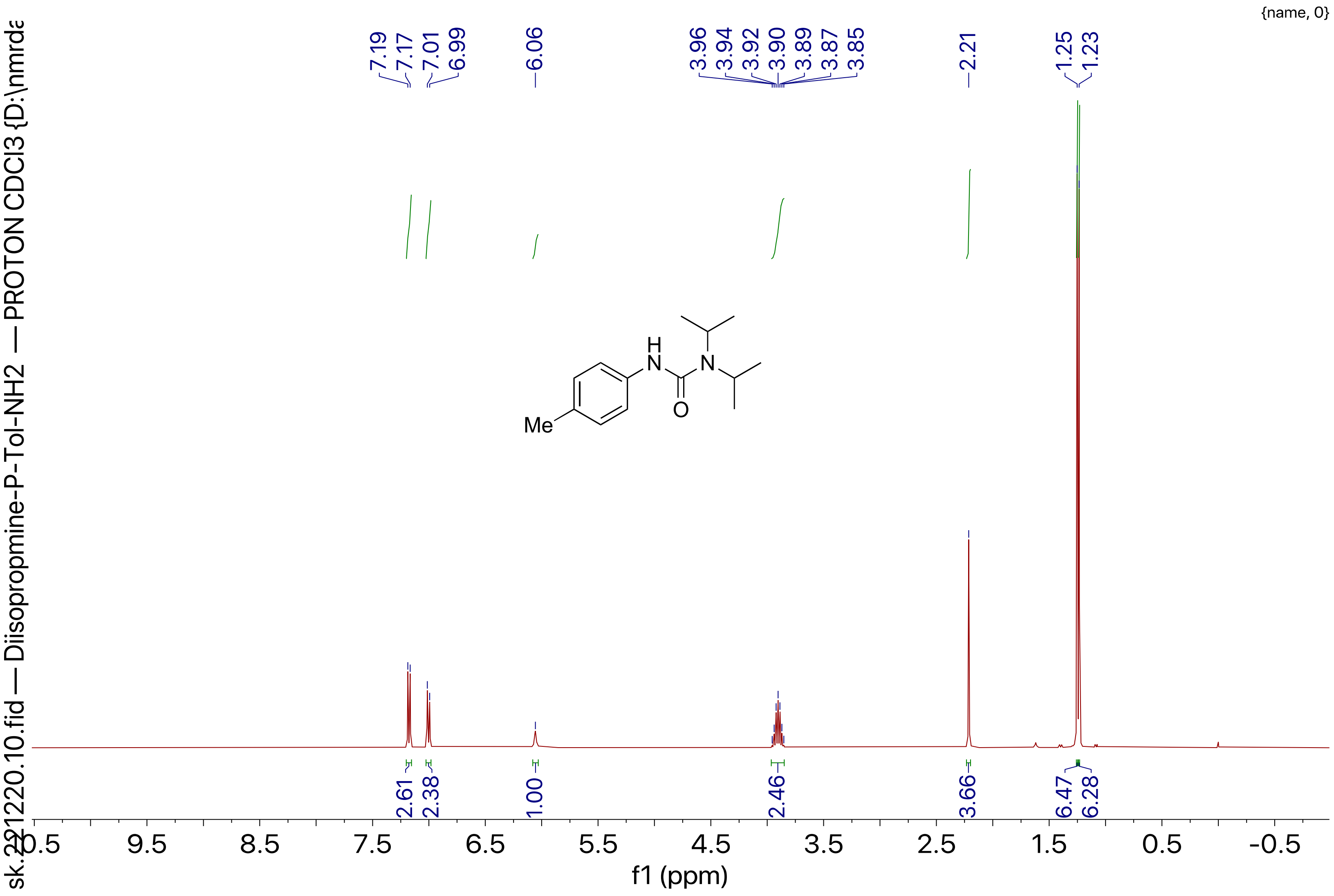

^{1}H NMR spectra of **5s** (400 MHz, RT, $CDCl_3$)

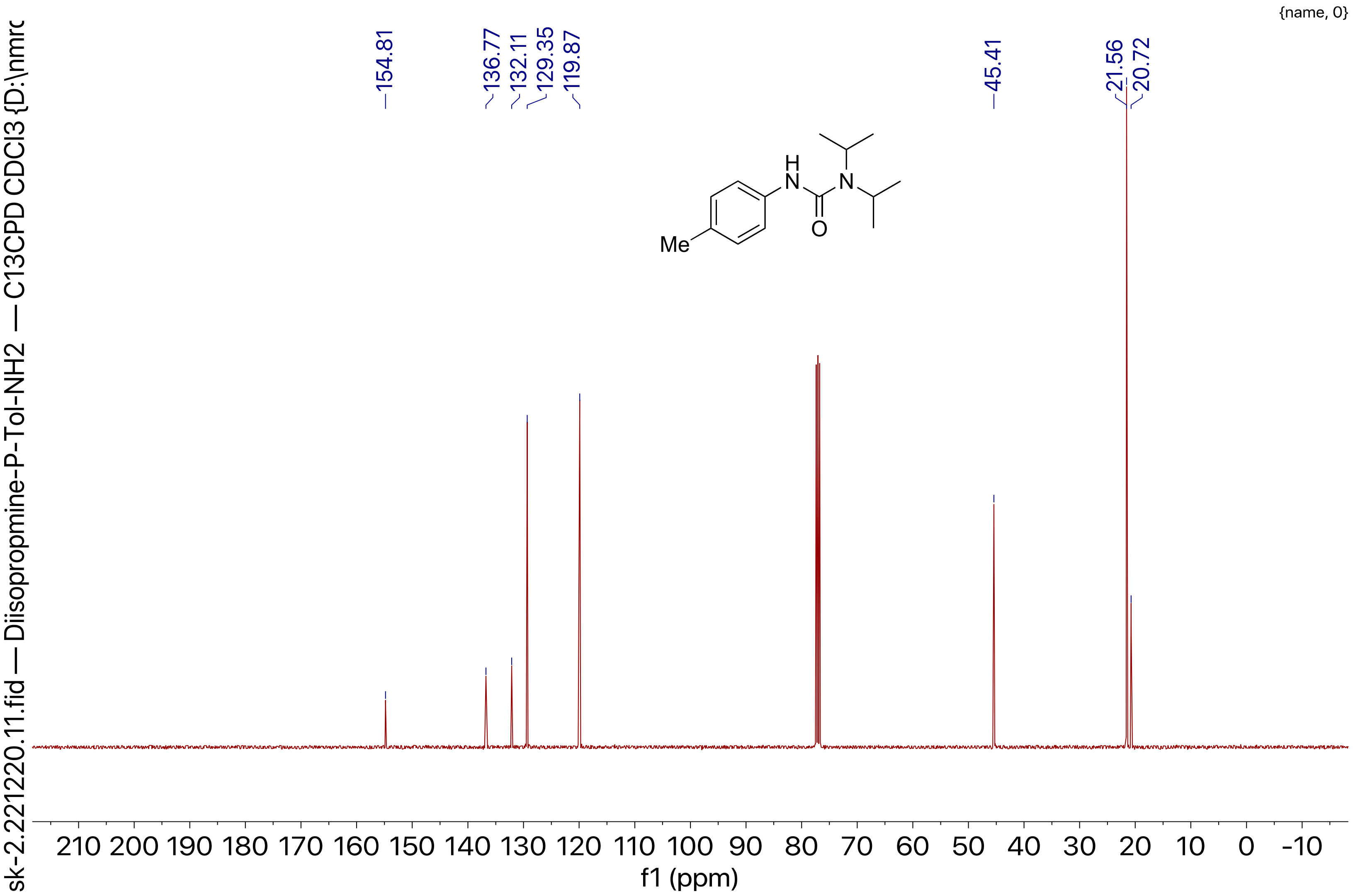

^{13}C NMR spectra of **5s** (101 MHz, RT, $CDCl_3$)

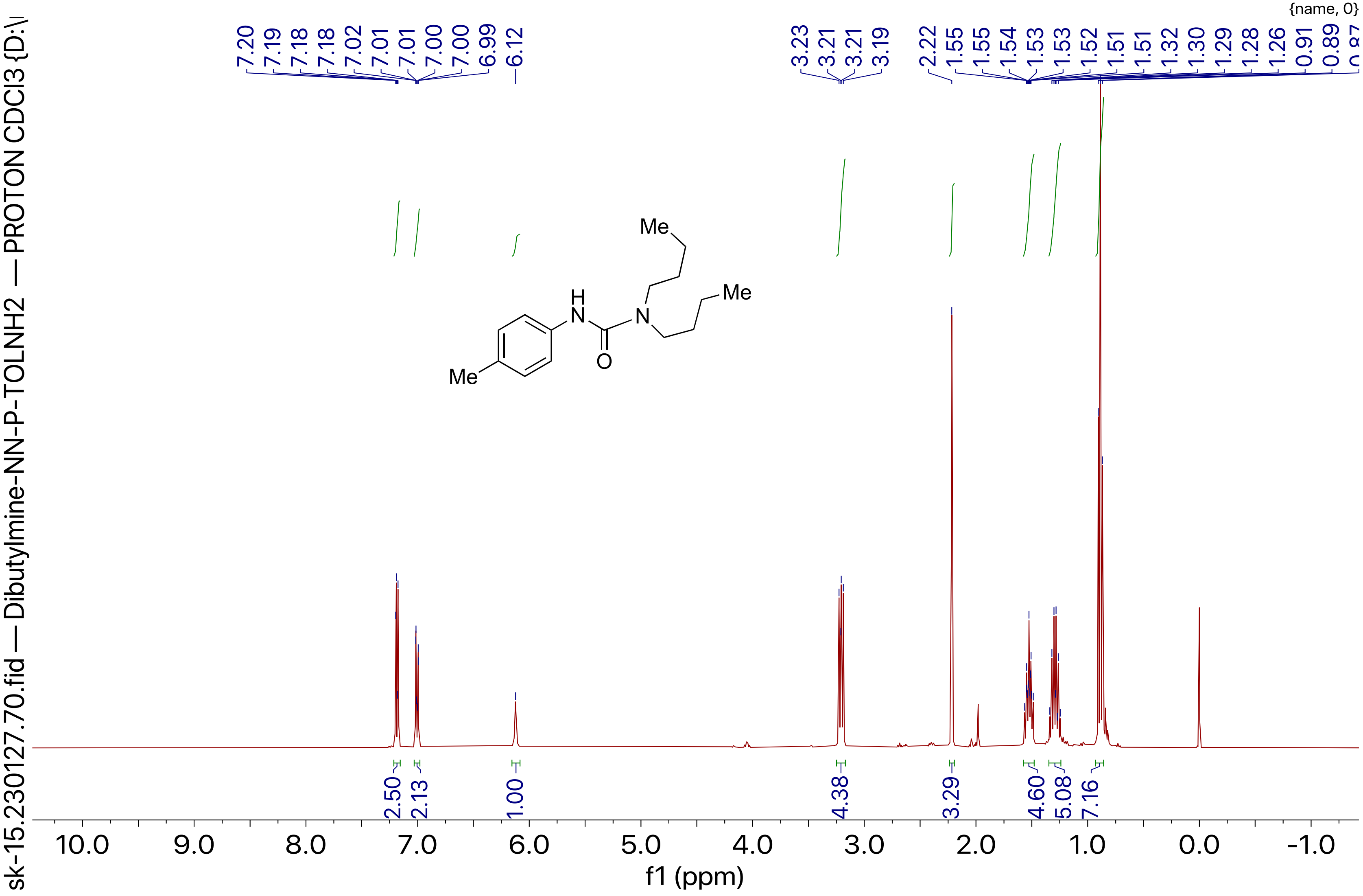

^{1}H NMR spectra of **5t** (400 MHz, RT, $CDCl_3$)

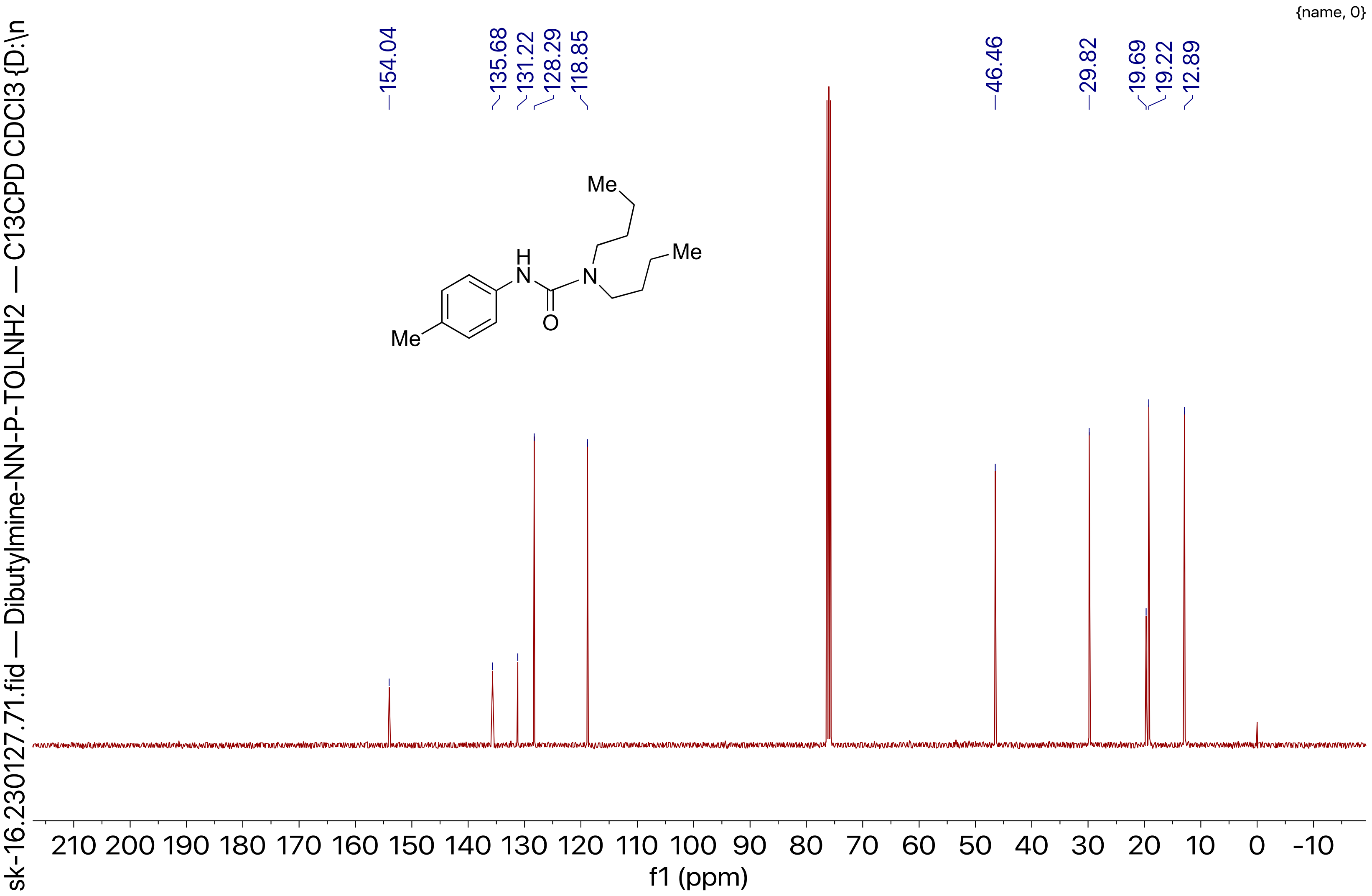

^{13}C NMR spectra of **5t** (101 MHz, RT, $CDCl_3$)

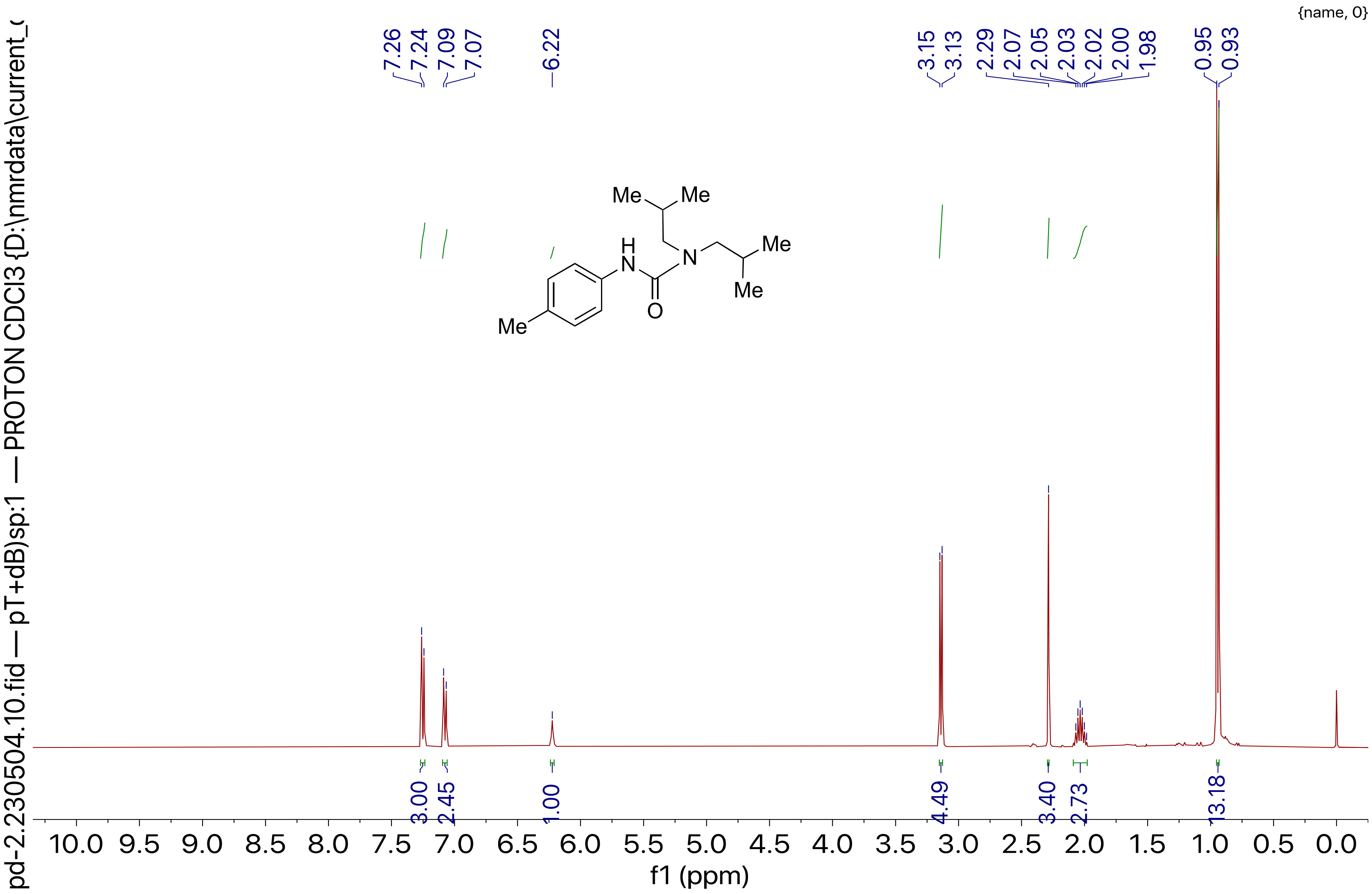

^{1}H NMR spectra of **5u** (400 MHz, RT, $CDCl_3$)

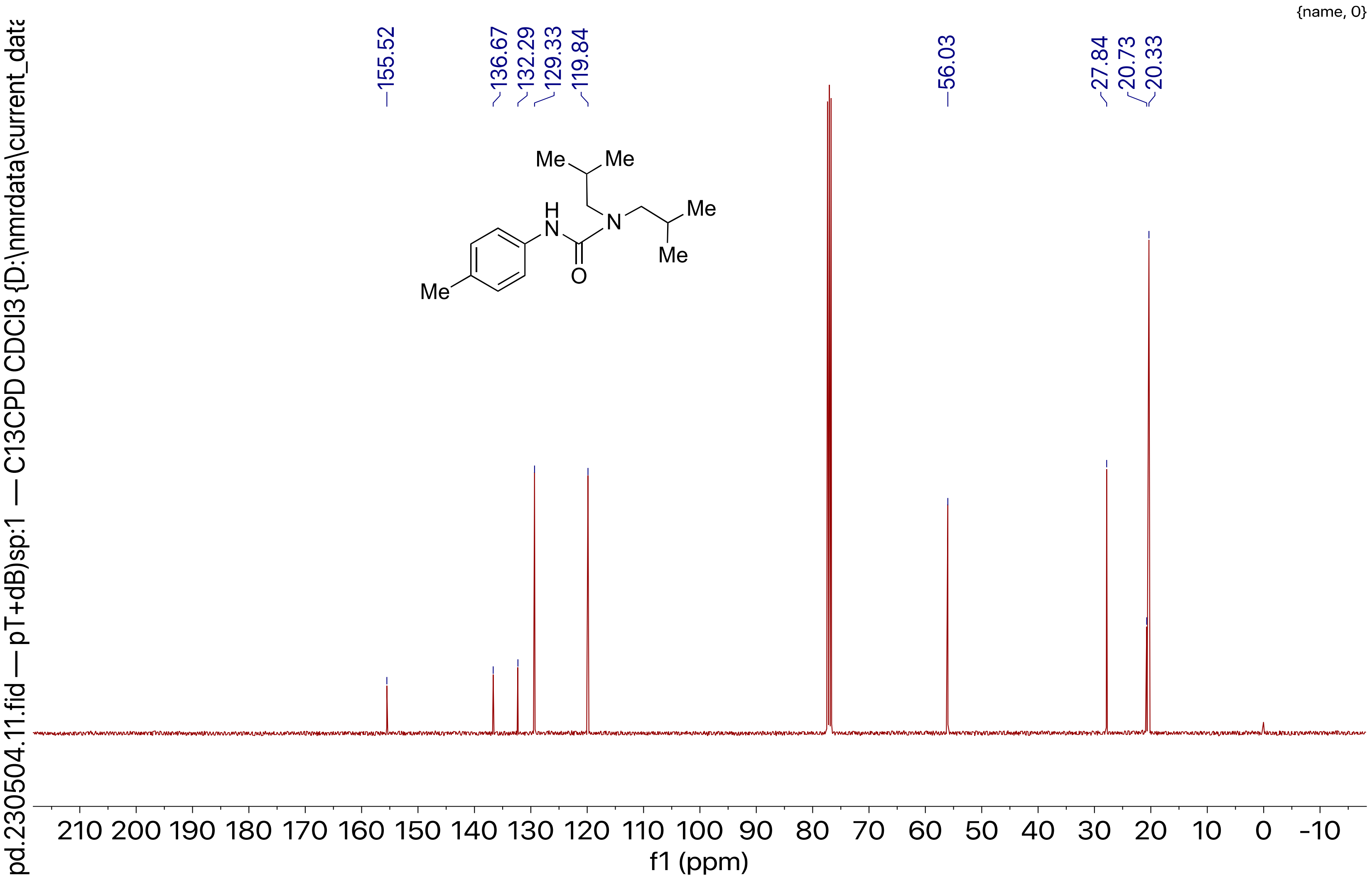

^{13}C NMR spectra of **5u** (101 MHz, RT, $CDCl_3$)

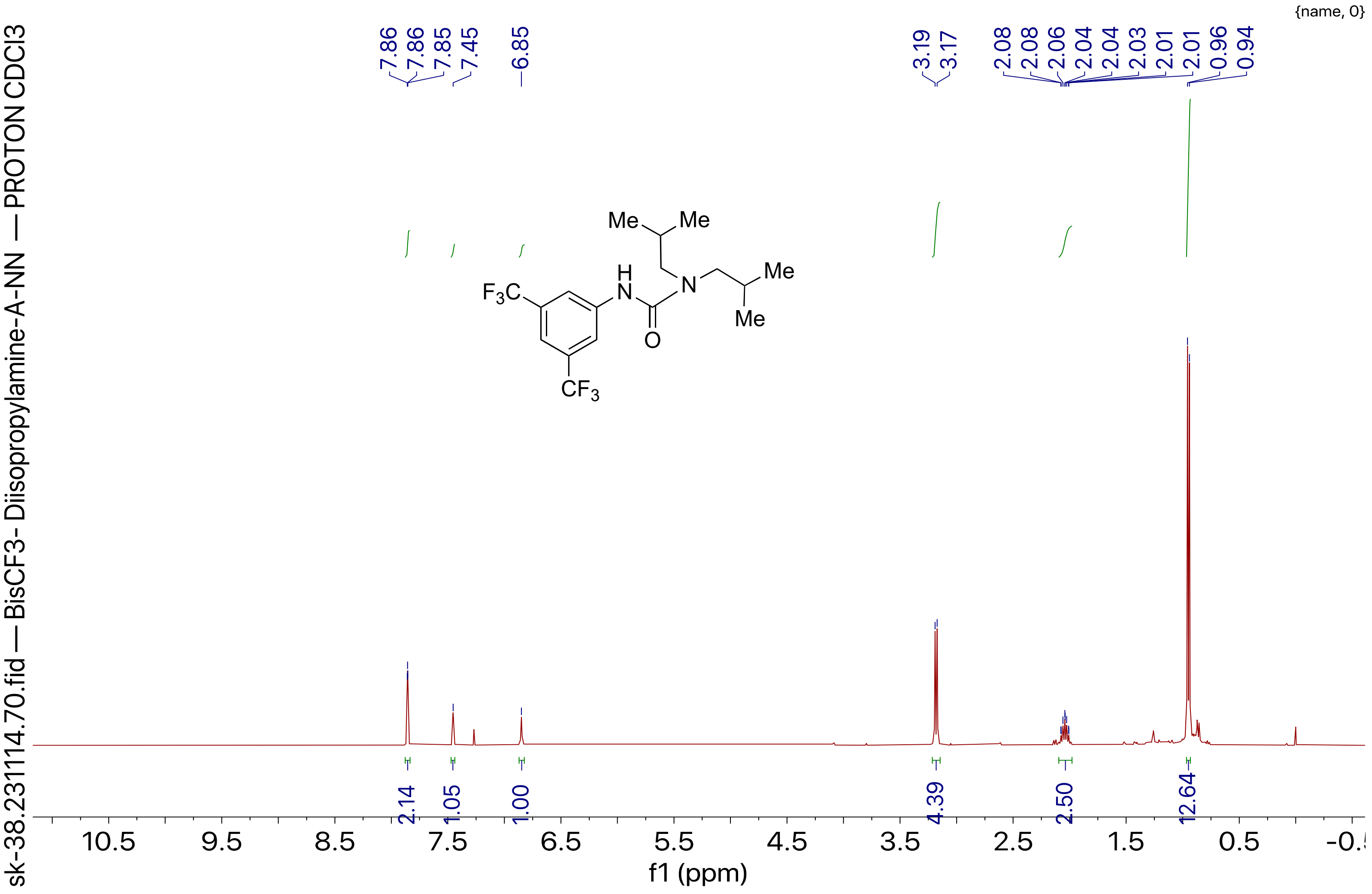

^{1}H NMR spectra of **5u'** (400 MHz, RT, $CDCl_3$)

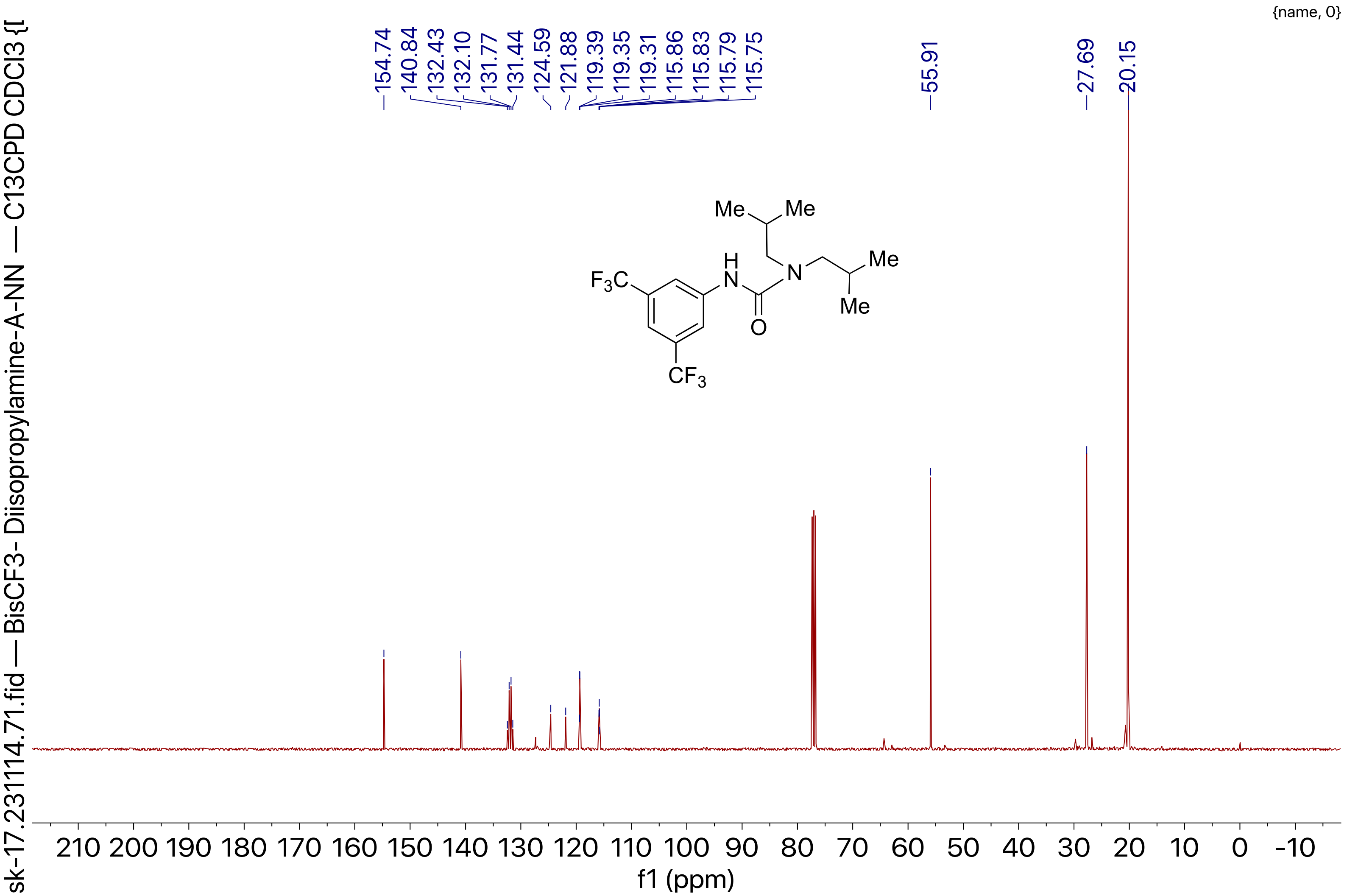

^{13}C NMR spectra of **5u’** (101 MHz, RT, $CDCl_3$)

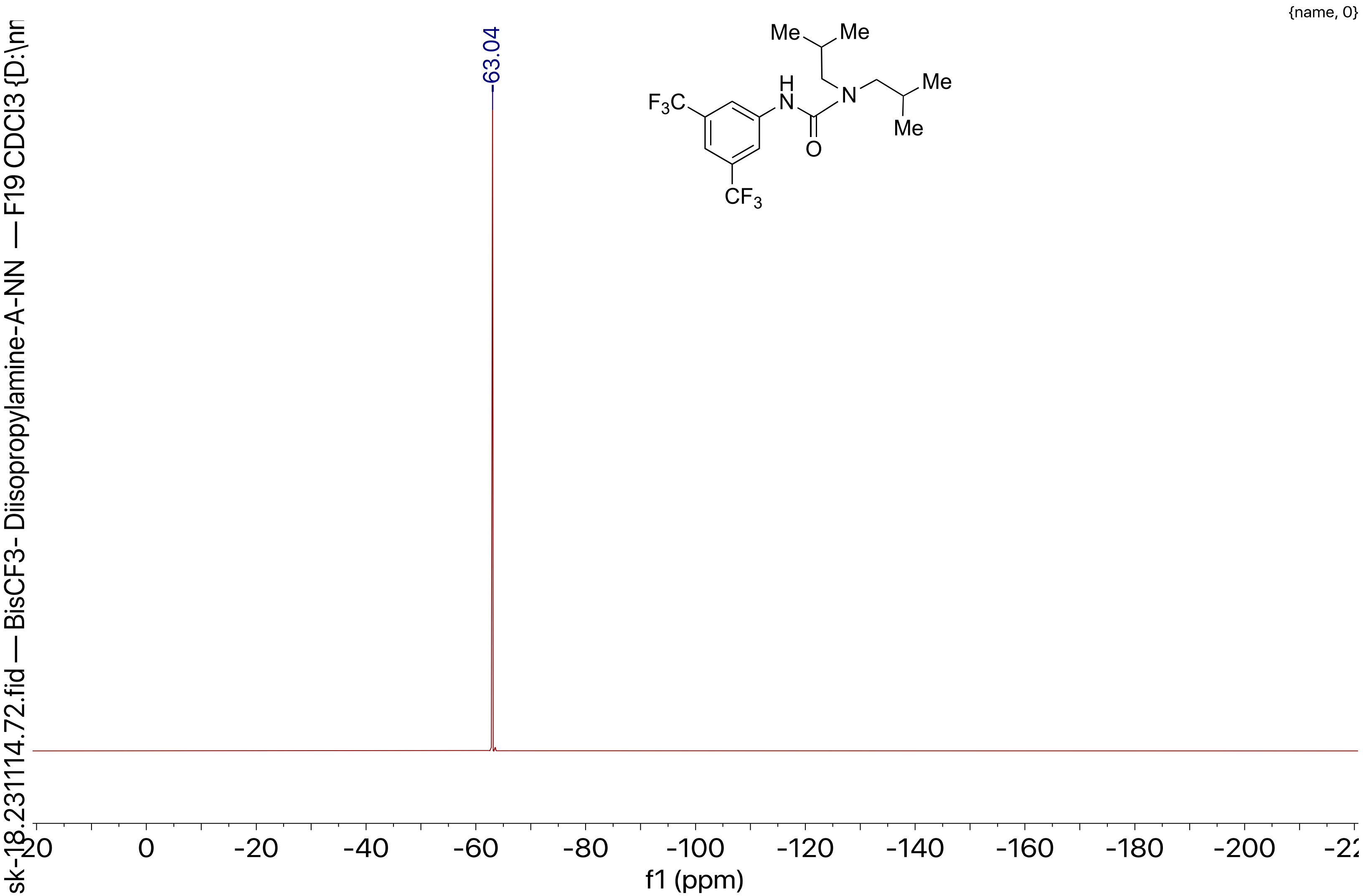

^{19}F NMR spectra of **5u'** (376 MHz, RT, $CDCl_3$)

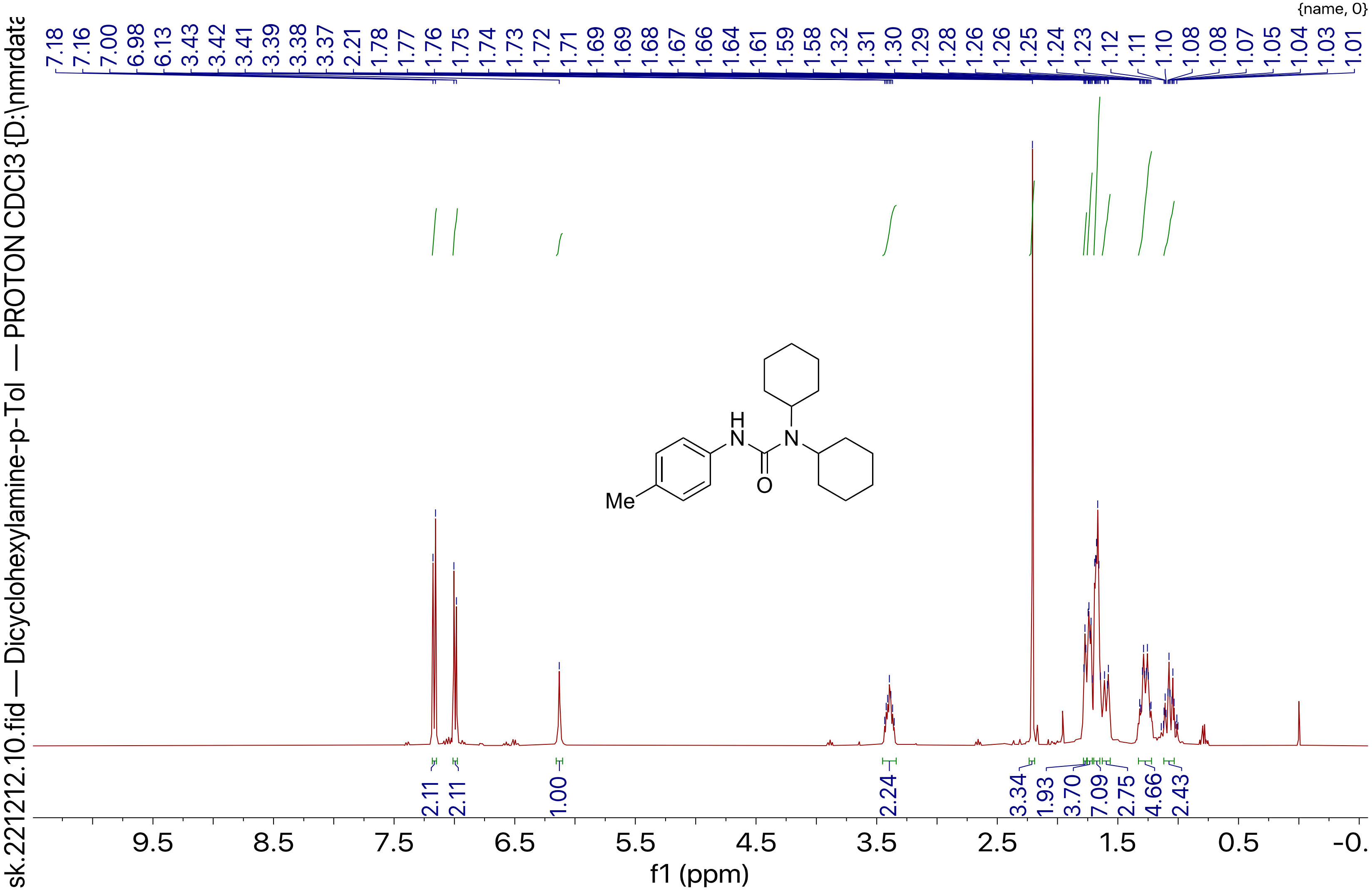

^{1}H NMR spectra of **5v** (400 MHz, RT, $CDCl_3$)

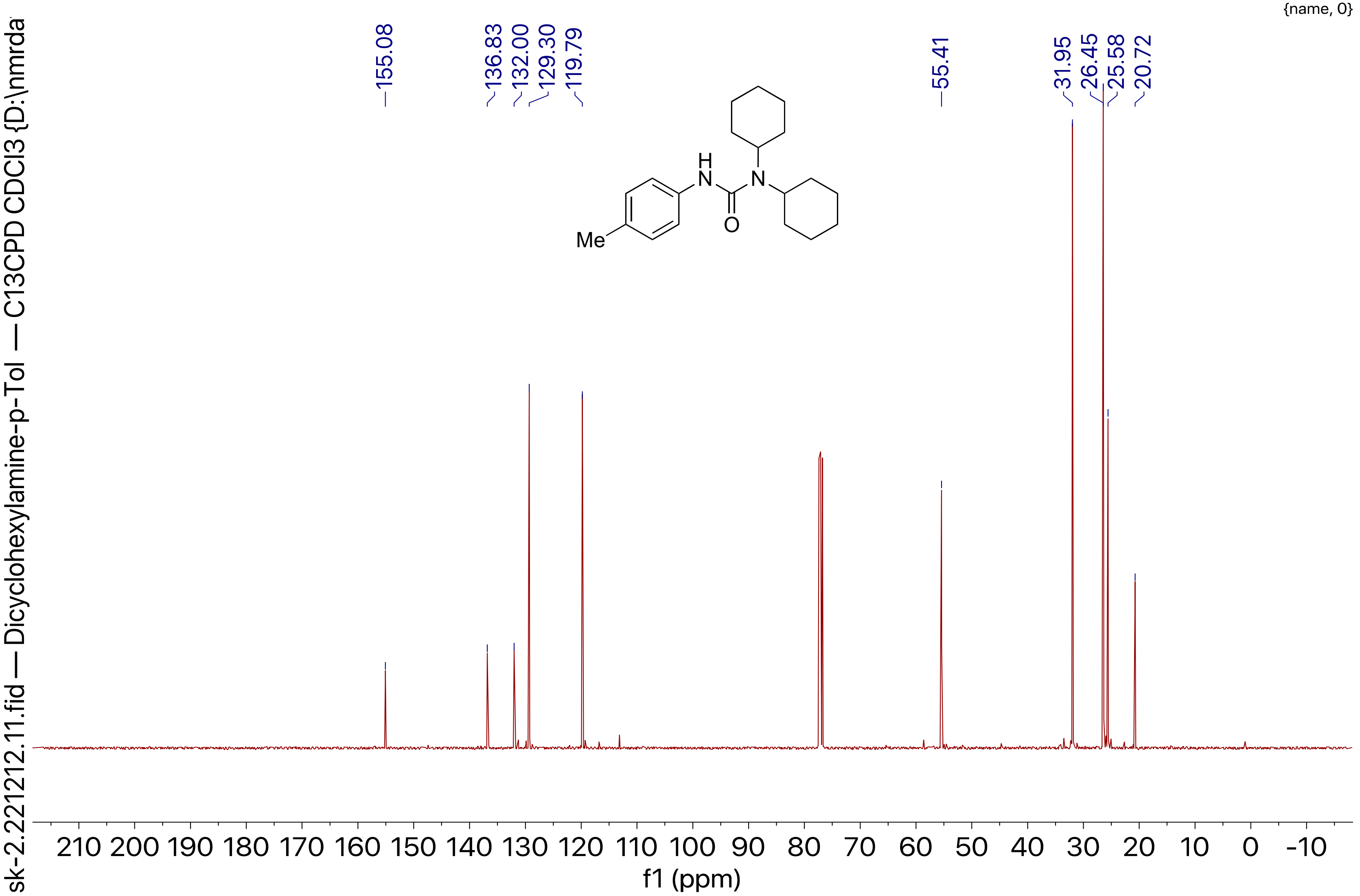

^{13}C NMR spectra of **5v** (101 MHz, RT, $CDCl_3$)

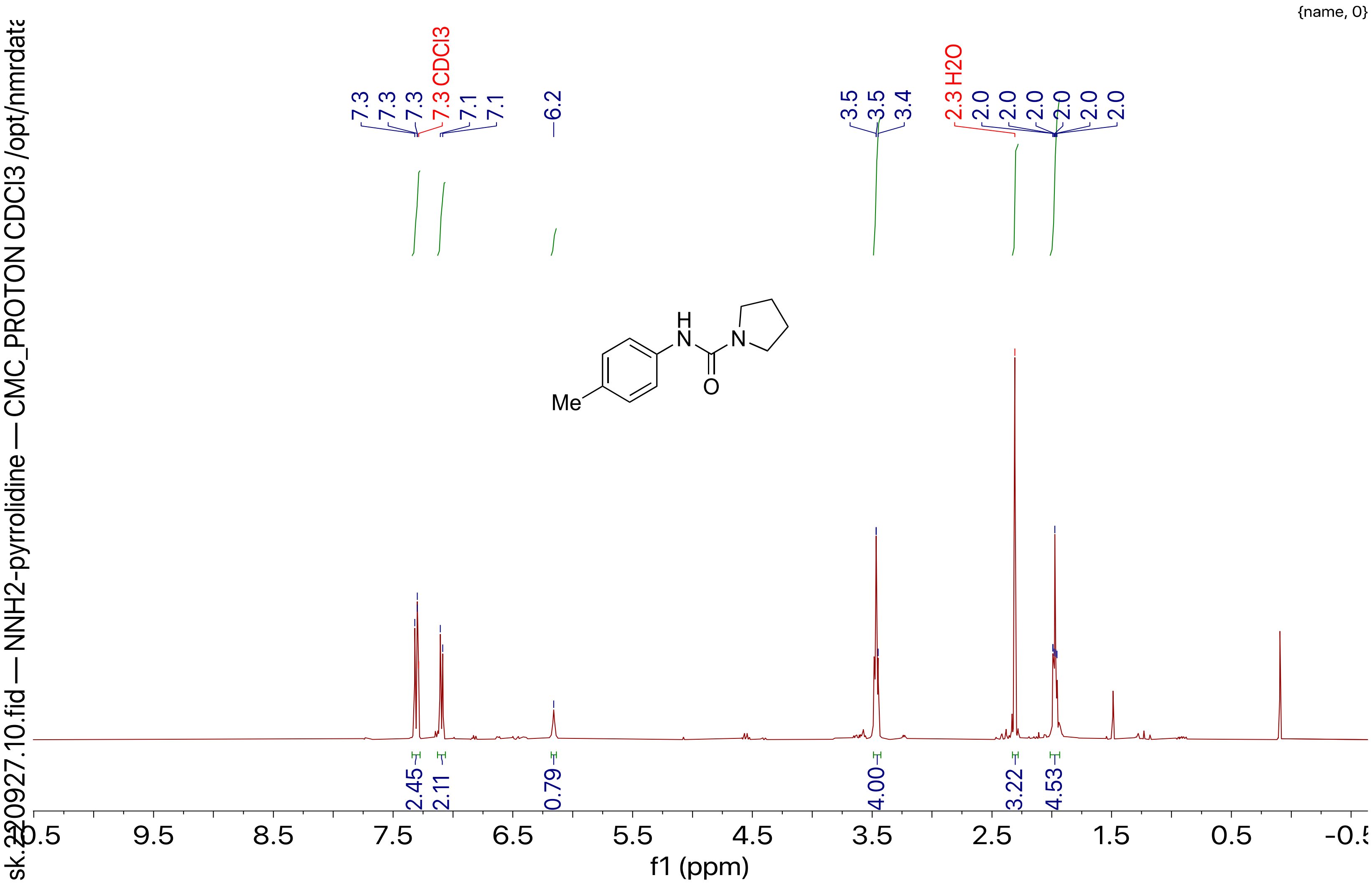

^{1}H NMR spectra of **5w** (400 MHz, RT, $CDCl_3$)

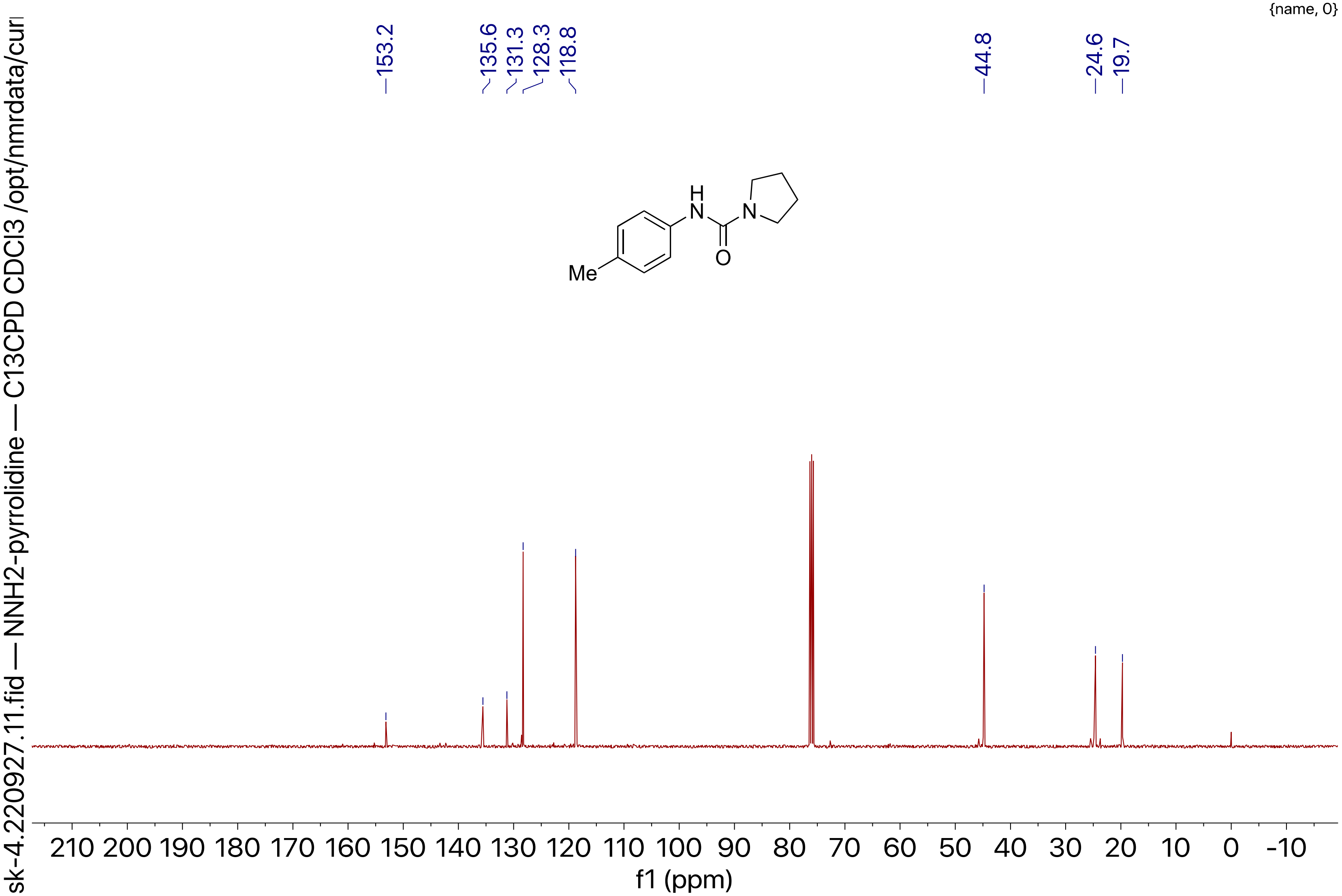

^{13}C NMR spectra of **5w** (101 MHz, RT, $CDCl_3$)

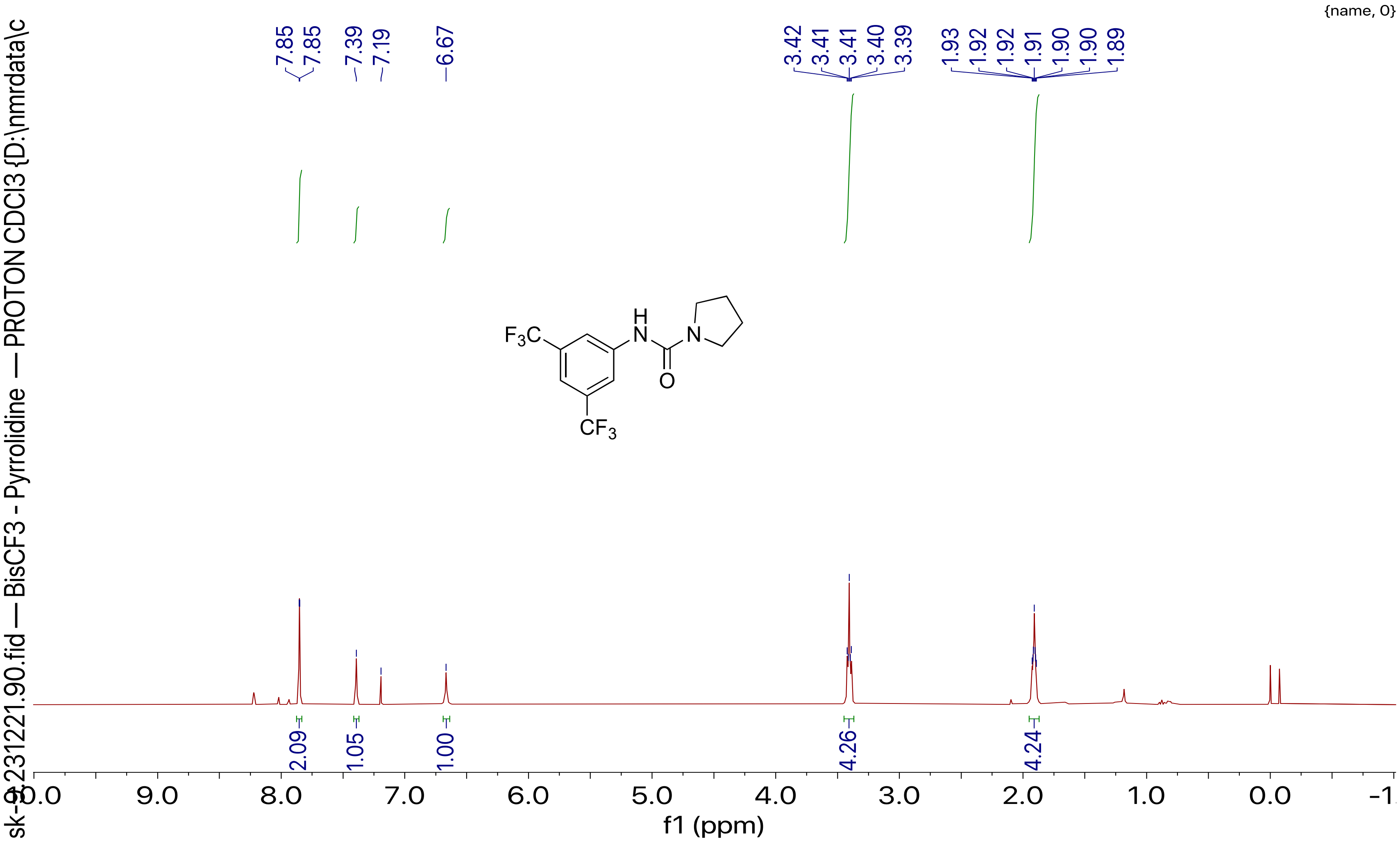

^{1}H NMR spectra of **5w'** (400 MHz, RT, $CDCl_3$)

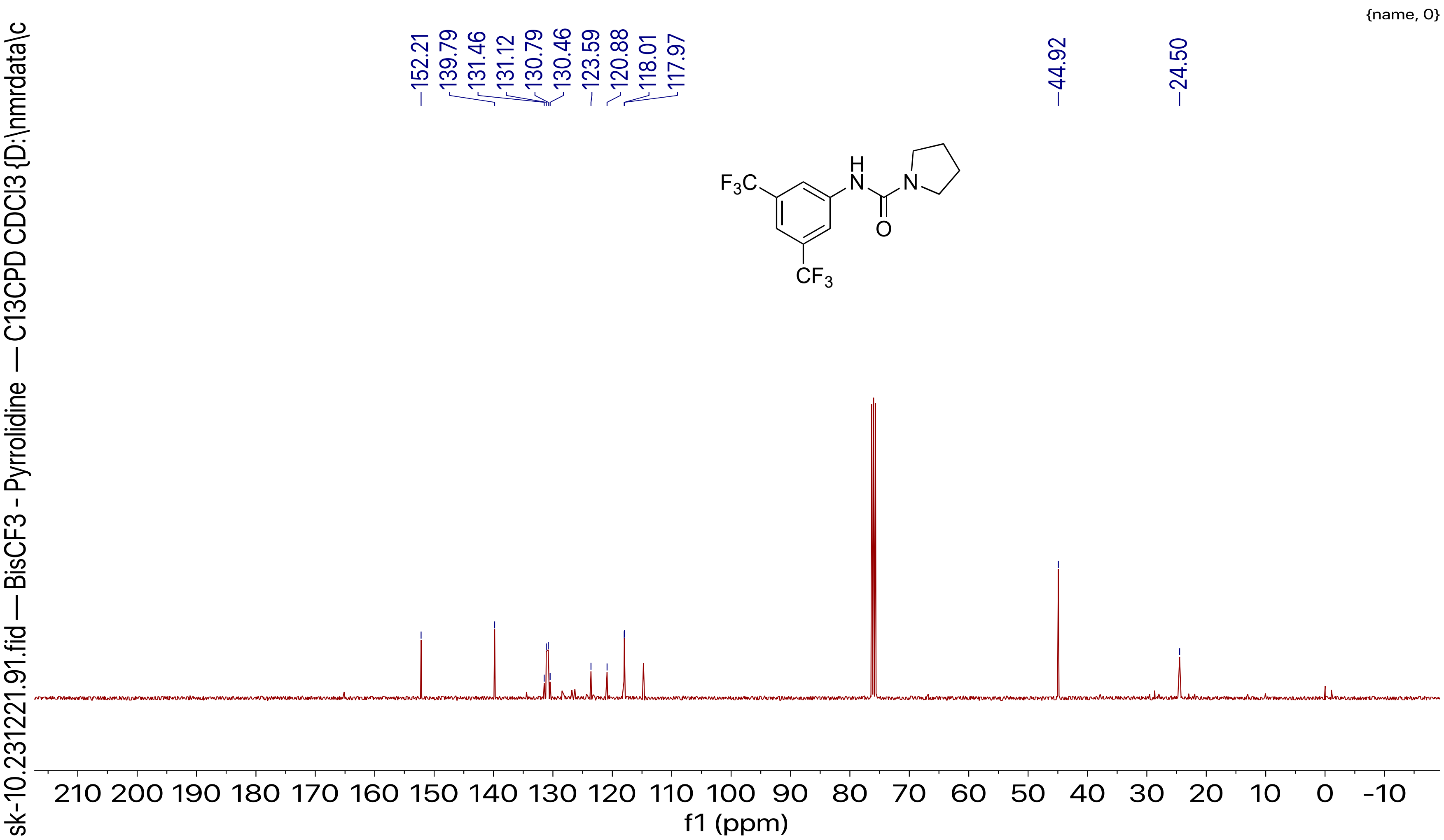

^{13}C NMR spectra of **5w’** ($CDCl_3$, 101 MHz, RT)

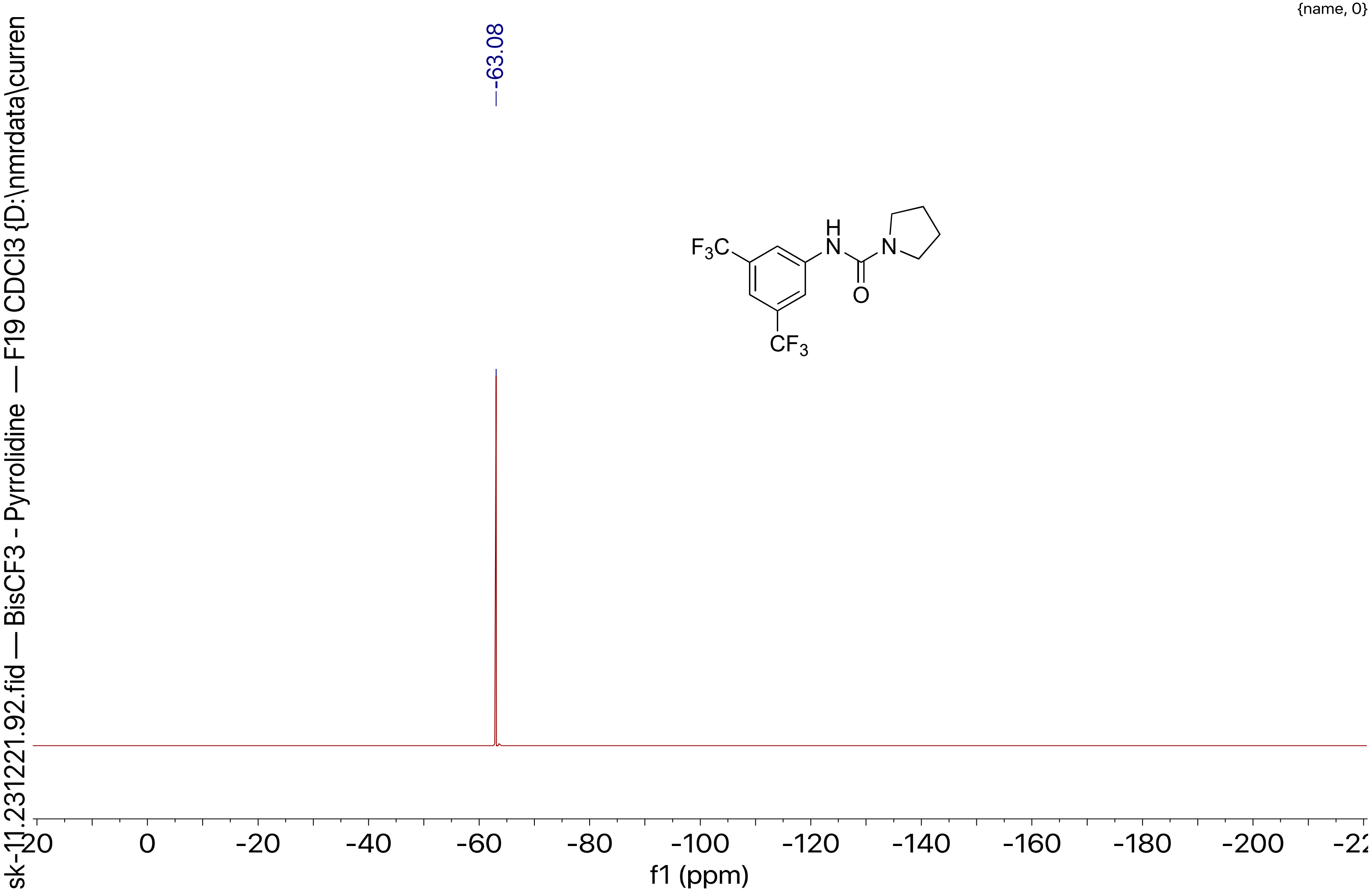

^{19}F NMR spectra of **5w'** ($CDCl_3$, 376 MHz, RT)

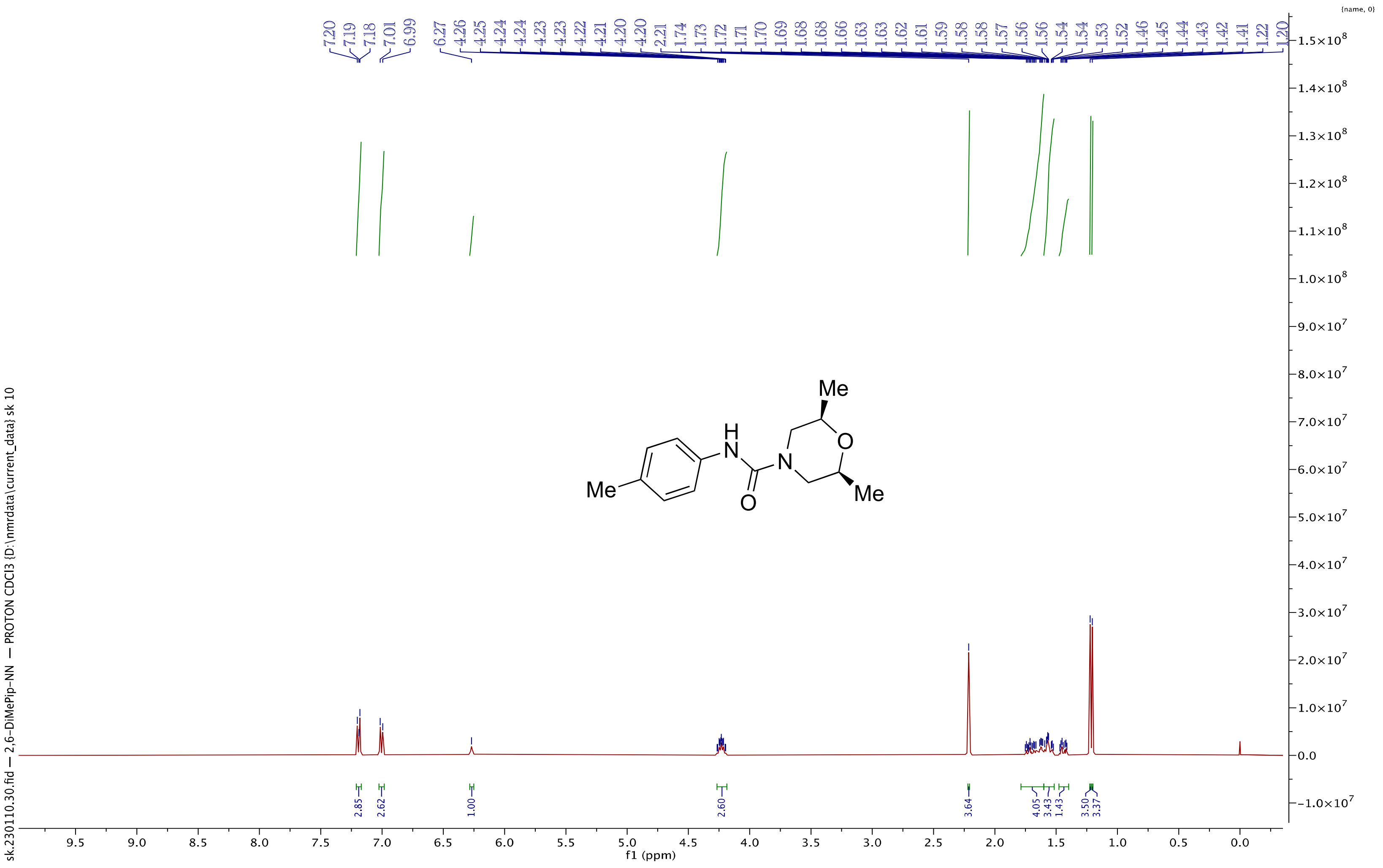

^{1}H NMR spectra of **5x** (400 MHz, RT, $CDCl_3$)

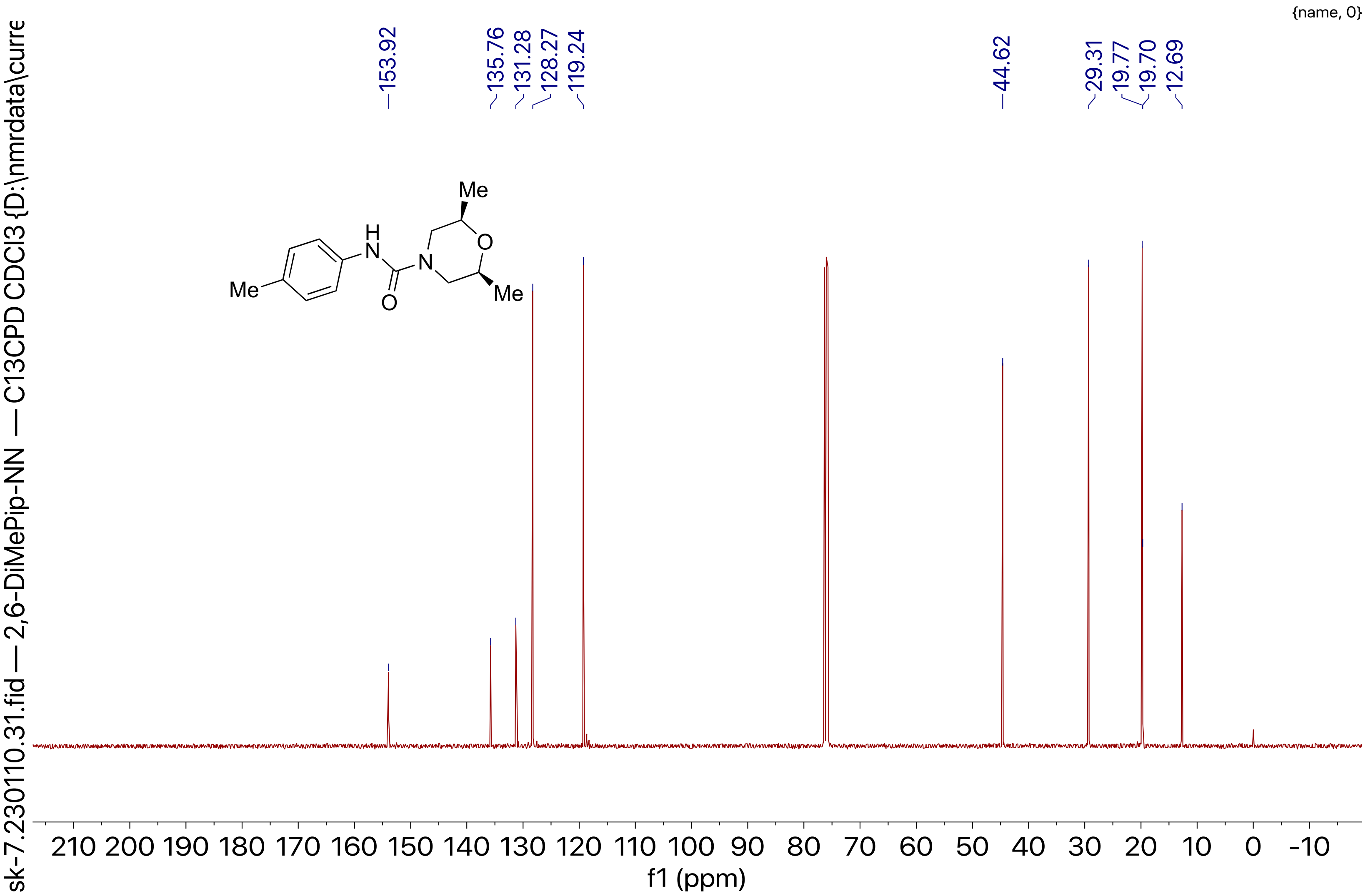

^{13}C NMR spectra of **5x** (101 MHz, RT, $CDCl_3$)

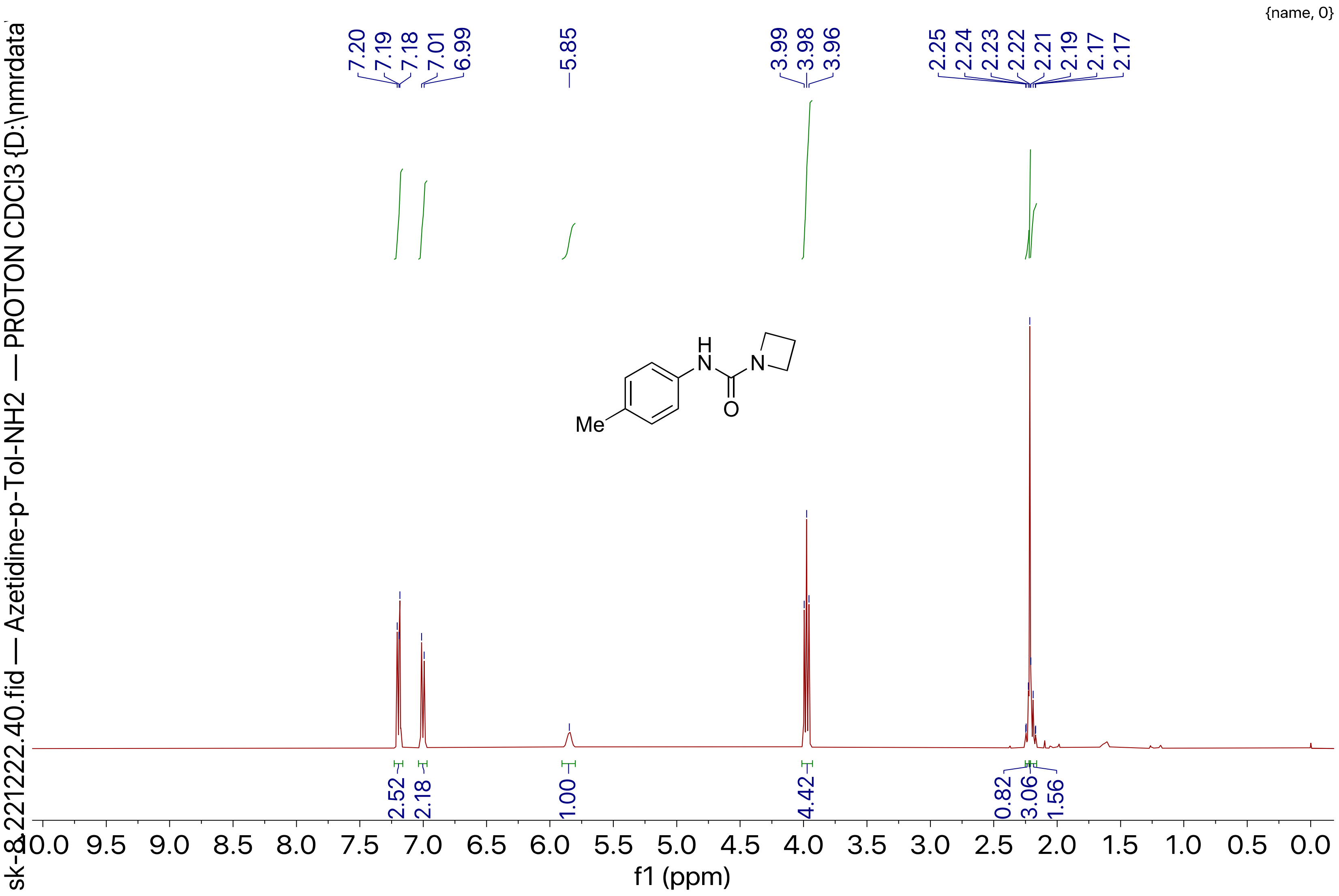

^{1}H NMR spectra of **5y** (400 MHz, RT, $CDCl_3$)

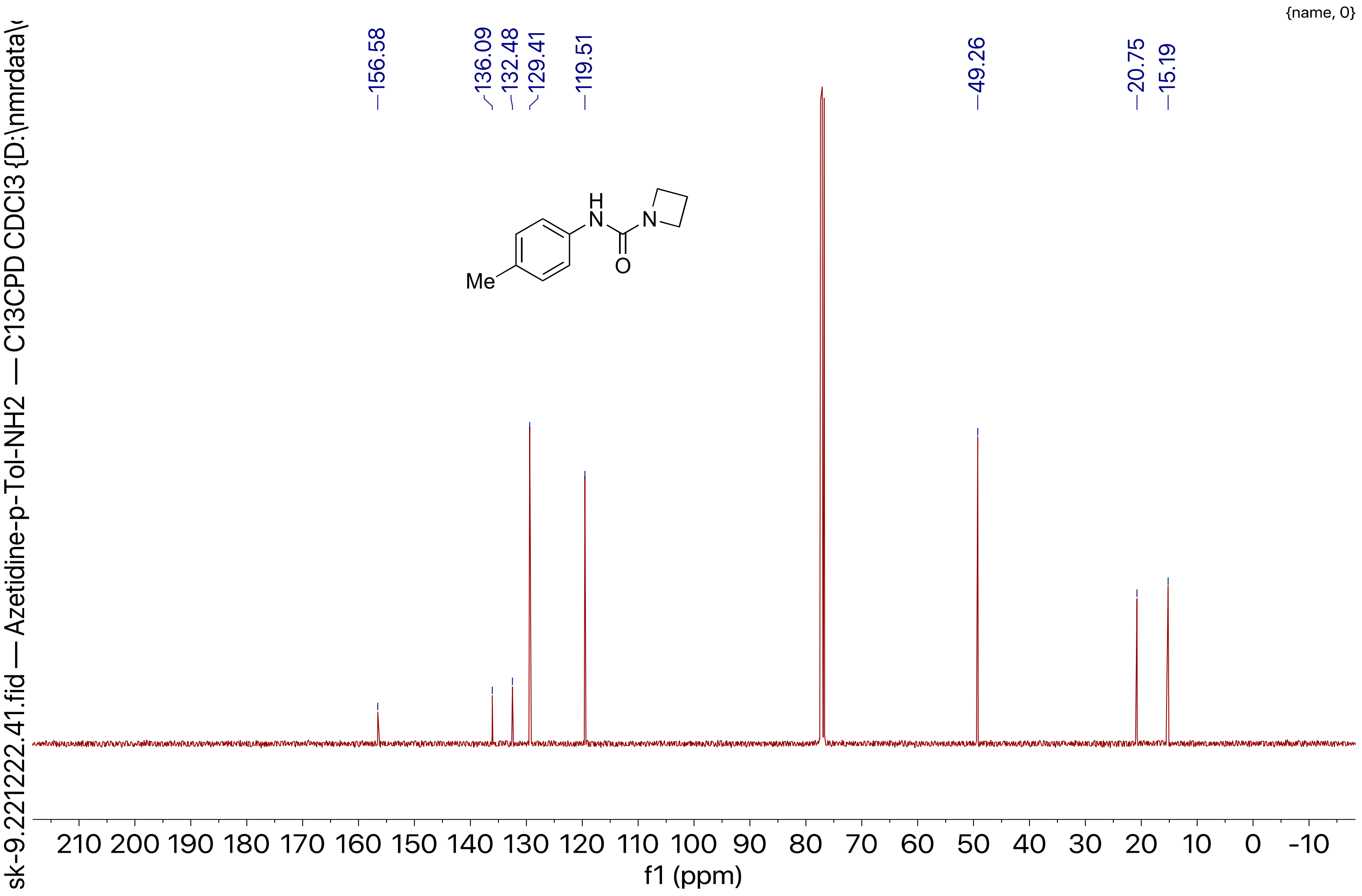

^{13}C NMR spectra of **5y** (100 MHz, RT, $CDCl_3$)

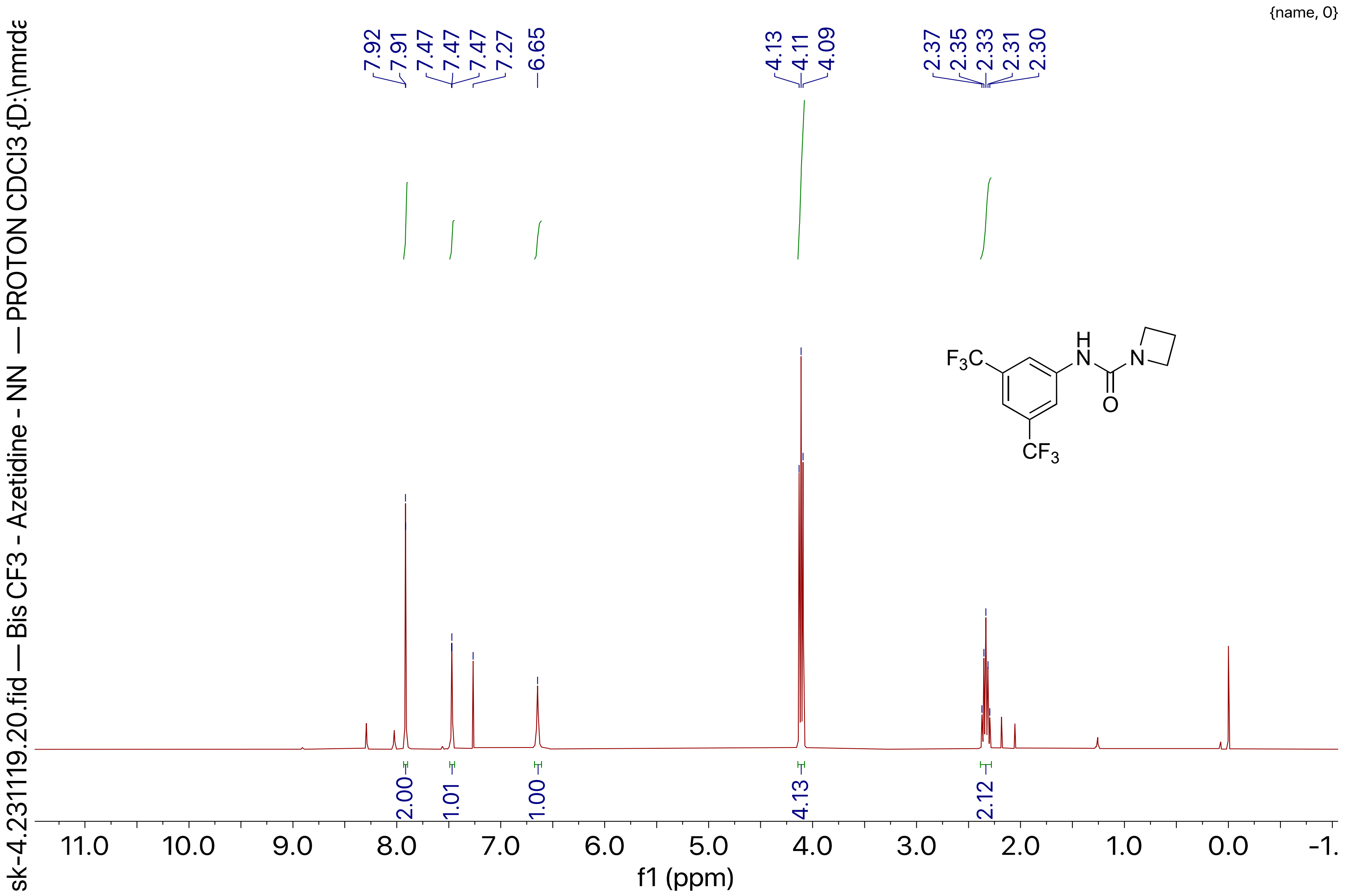

^{1}H NMR spectra of **5y’** (400 MHz, RT, $CDCl_3$)

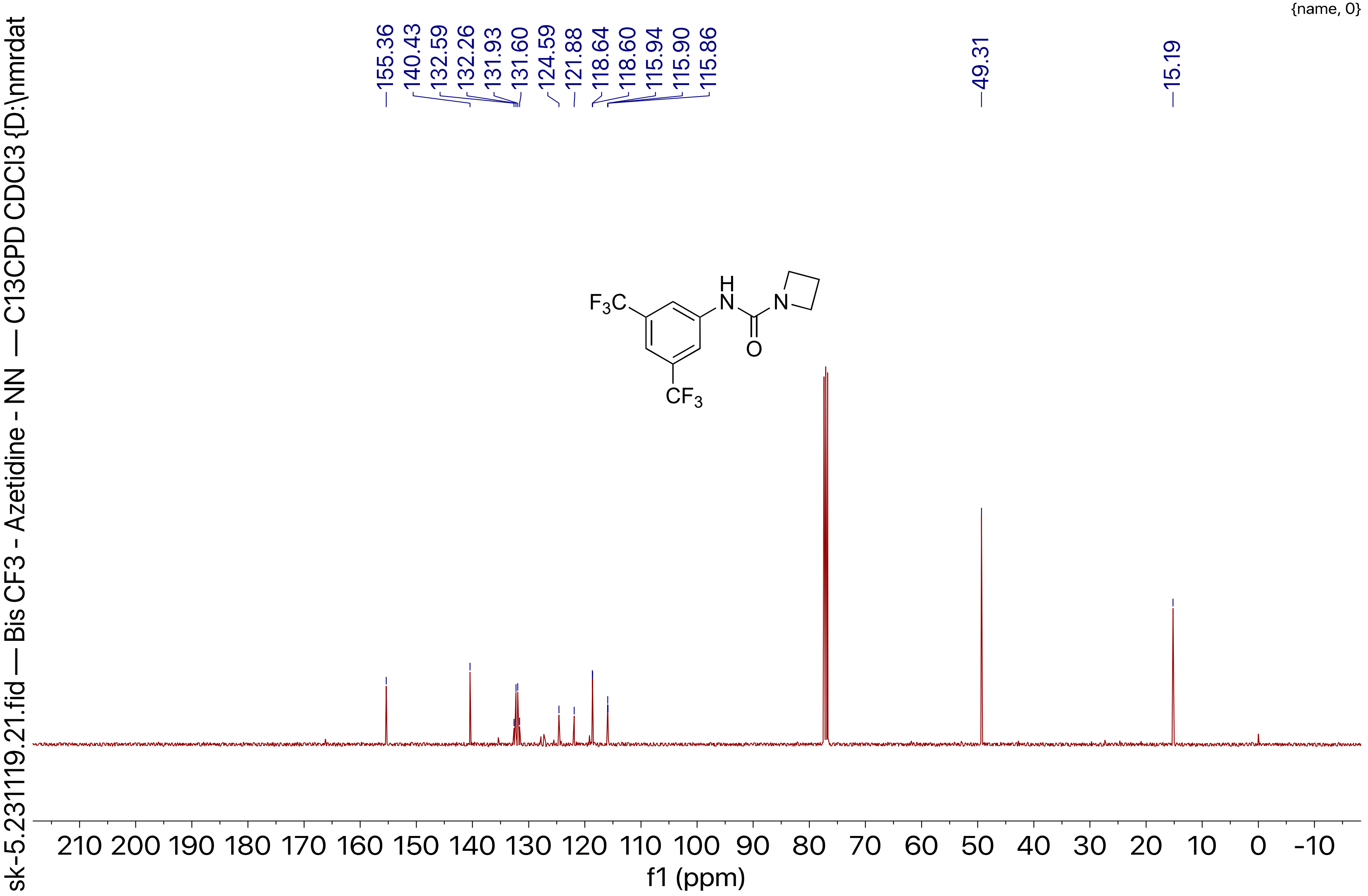

^{13}C NMR spectra of **5y’** (101 MHz, RT, $CDCl_3$)

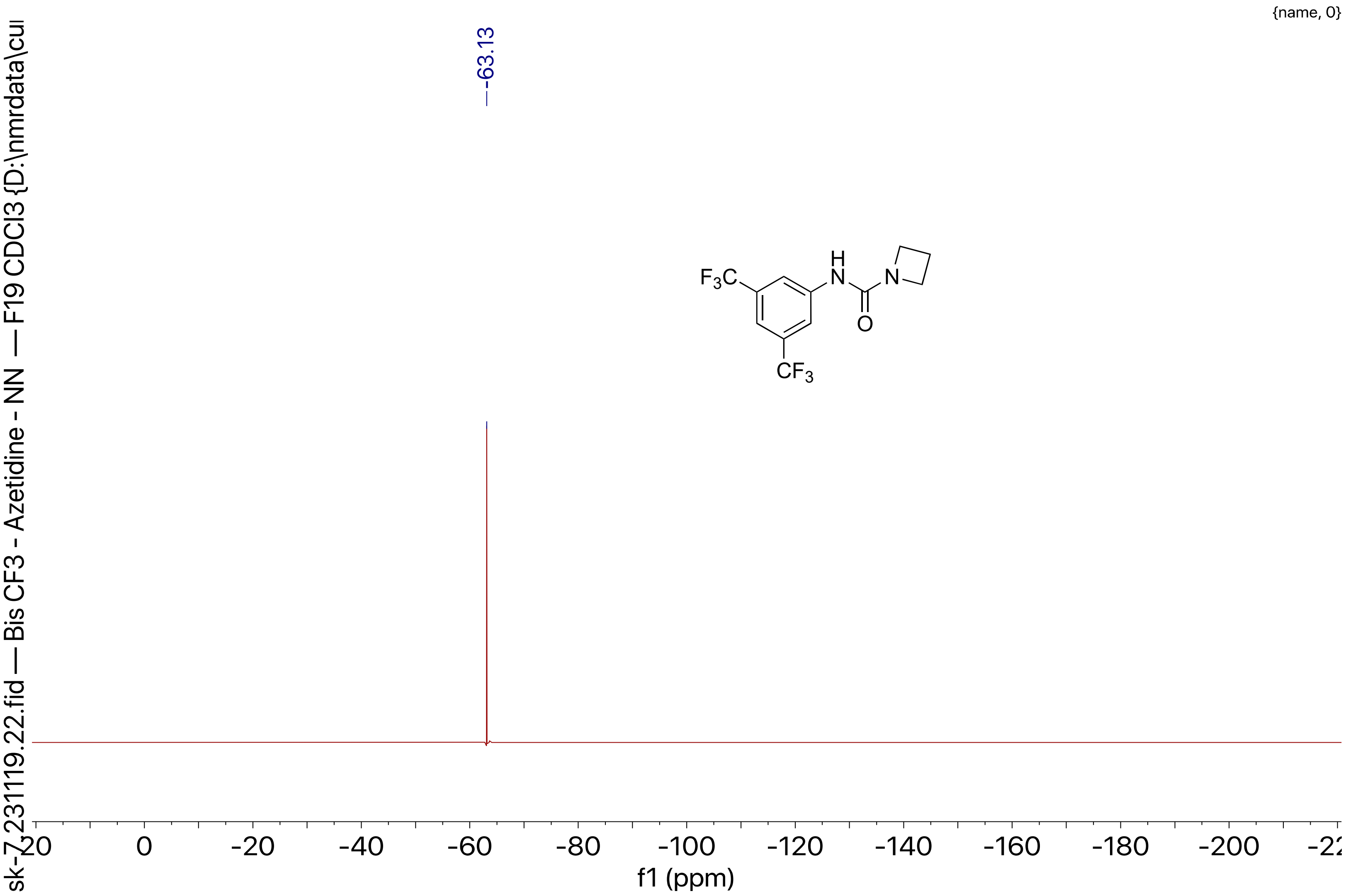

^{19}C NMR spectra of **5y'** (376 MHz, RT, $CDCl_3$)

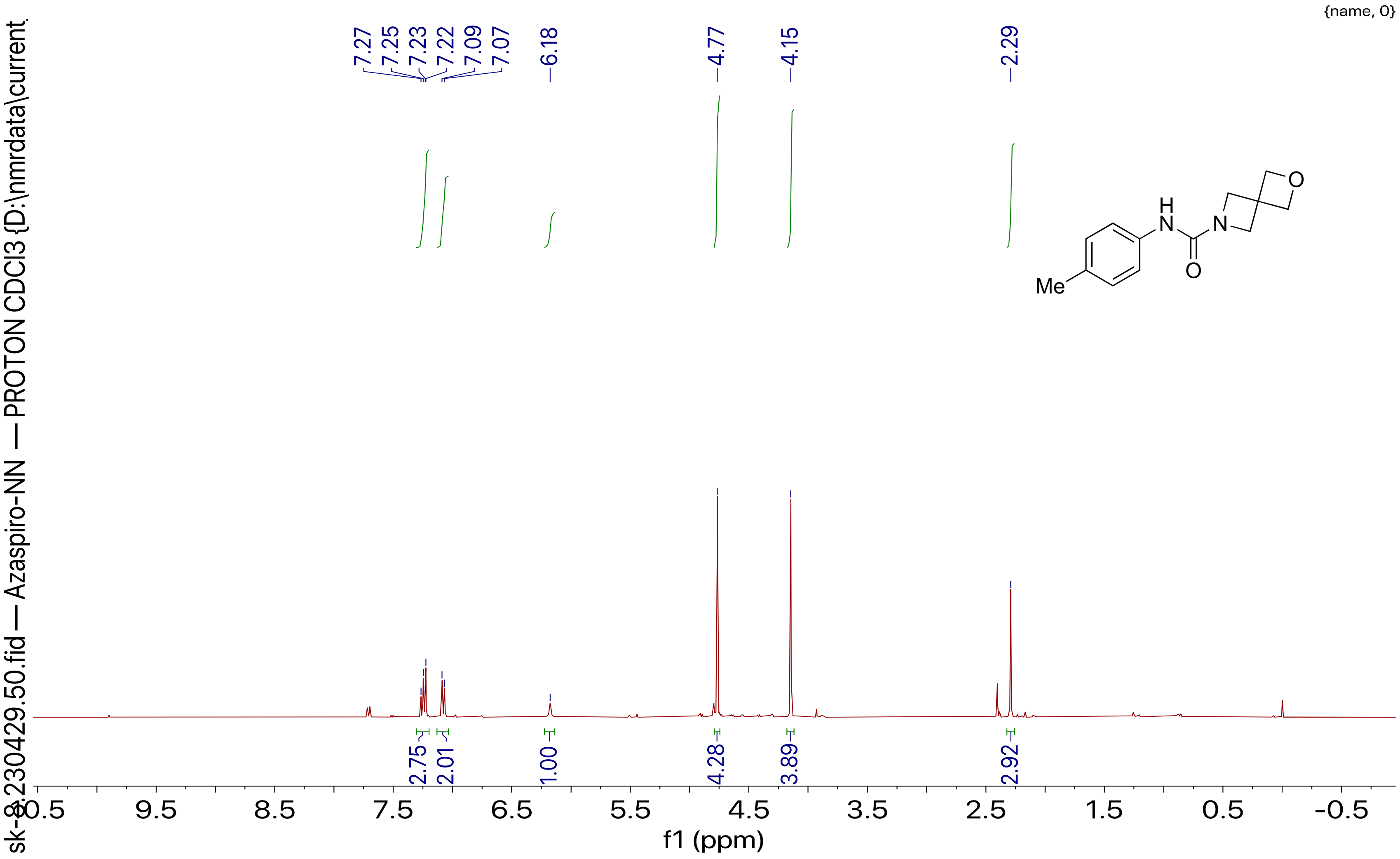

^{1}H NMR spectra of **5z** (400 MHz, RT, $CDCl_3$)

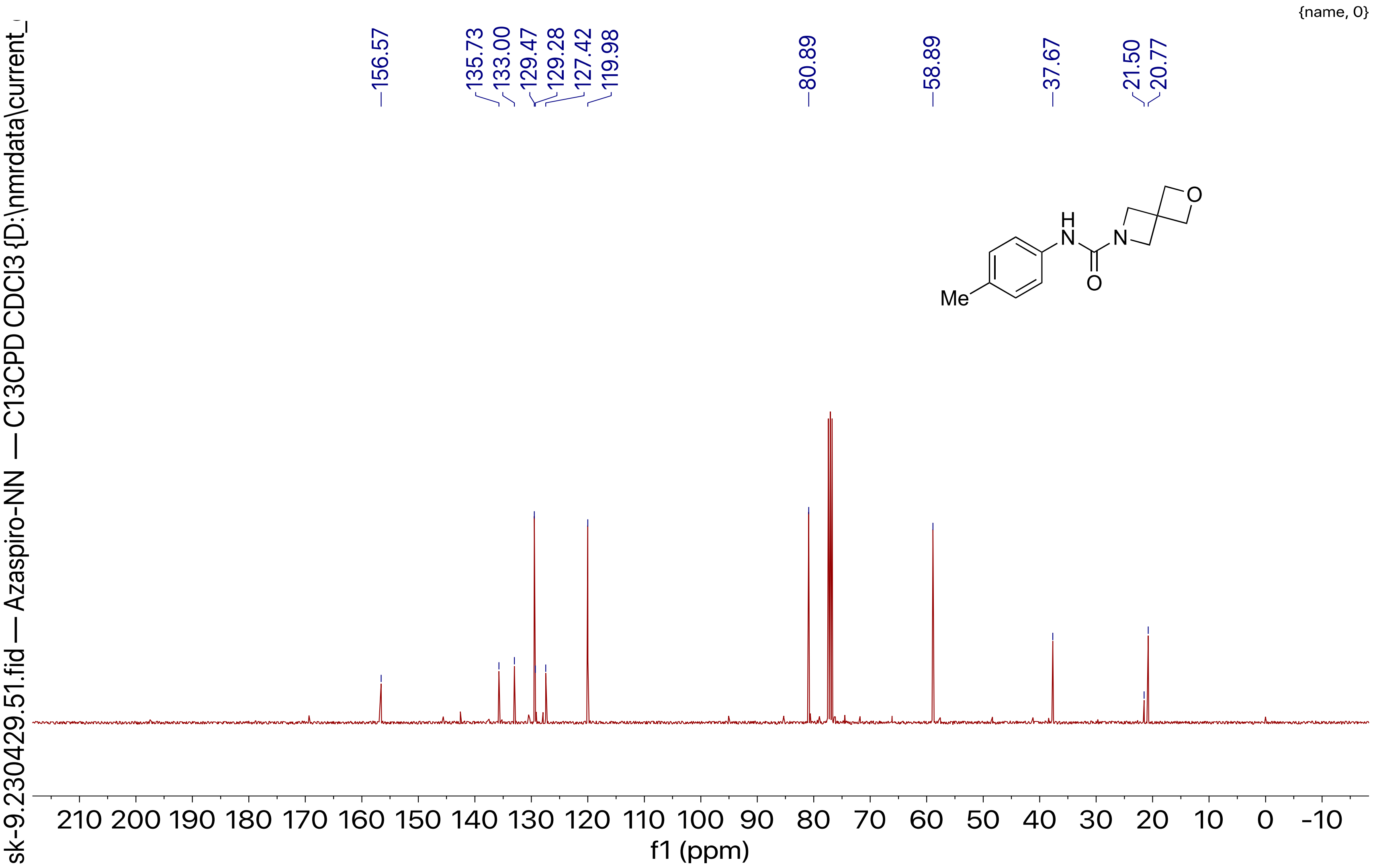

^{13}C NMR spectra of **5z** (101 MHz, RT, $CDCl_3$)

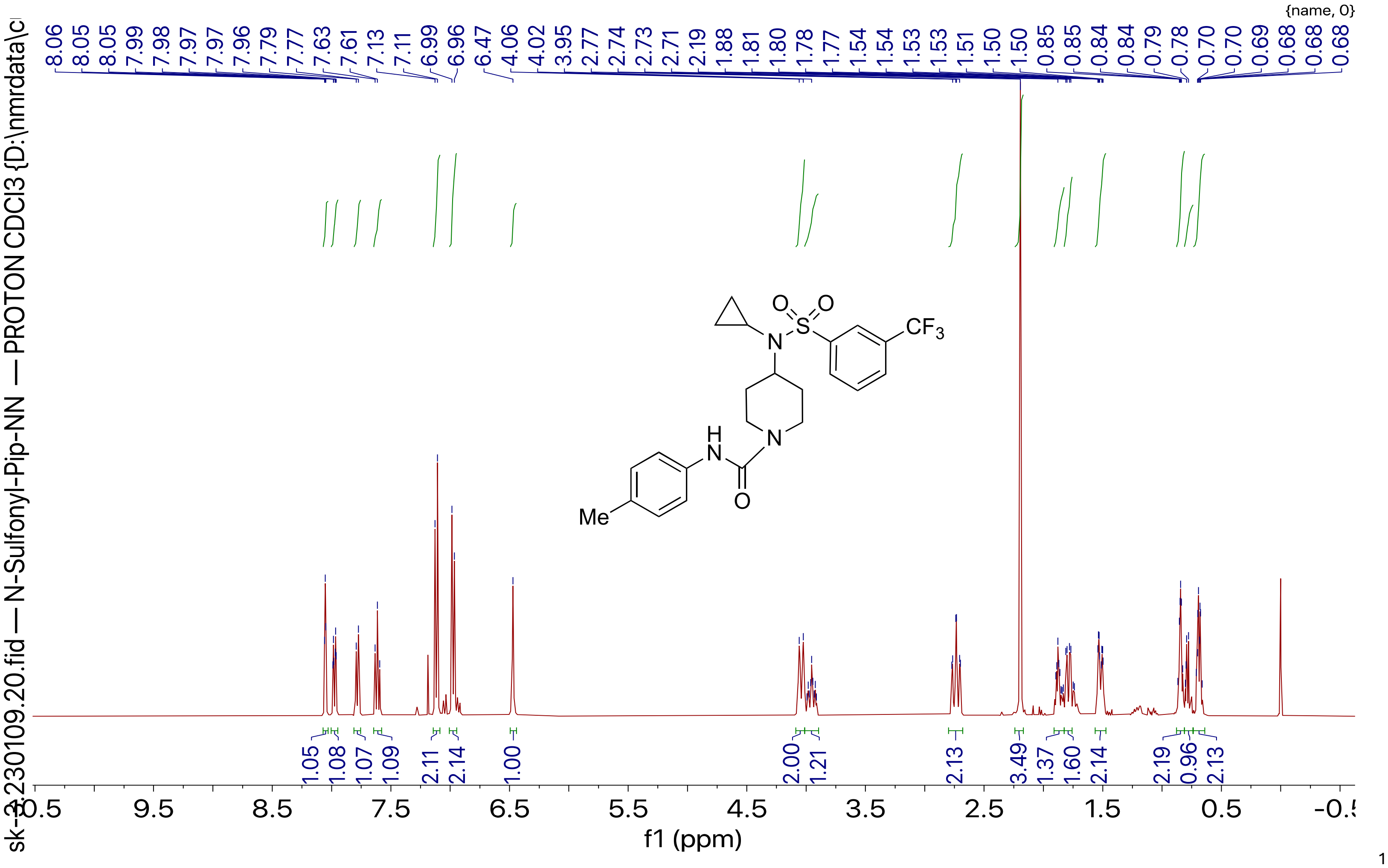

H NMR spectra of **5aa** (400 MHz, RT, $CDCl_3$)

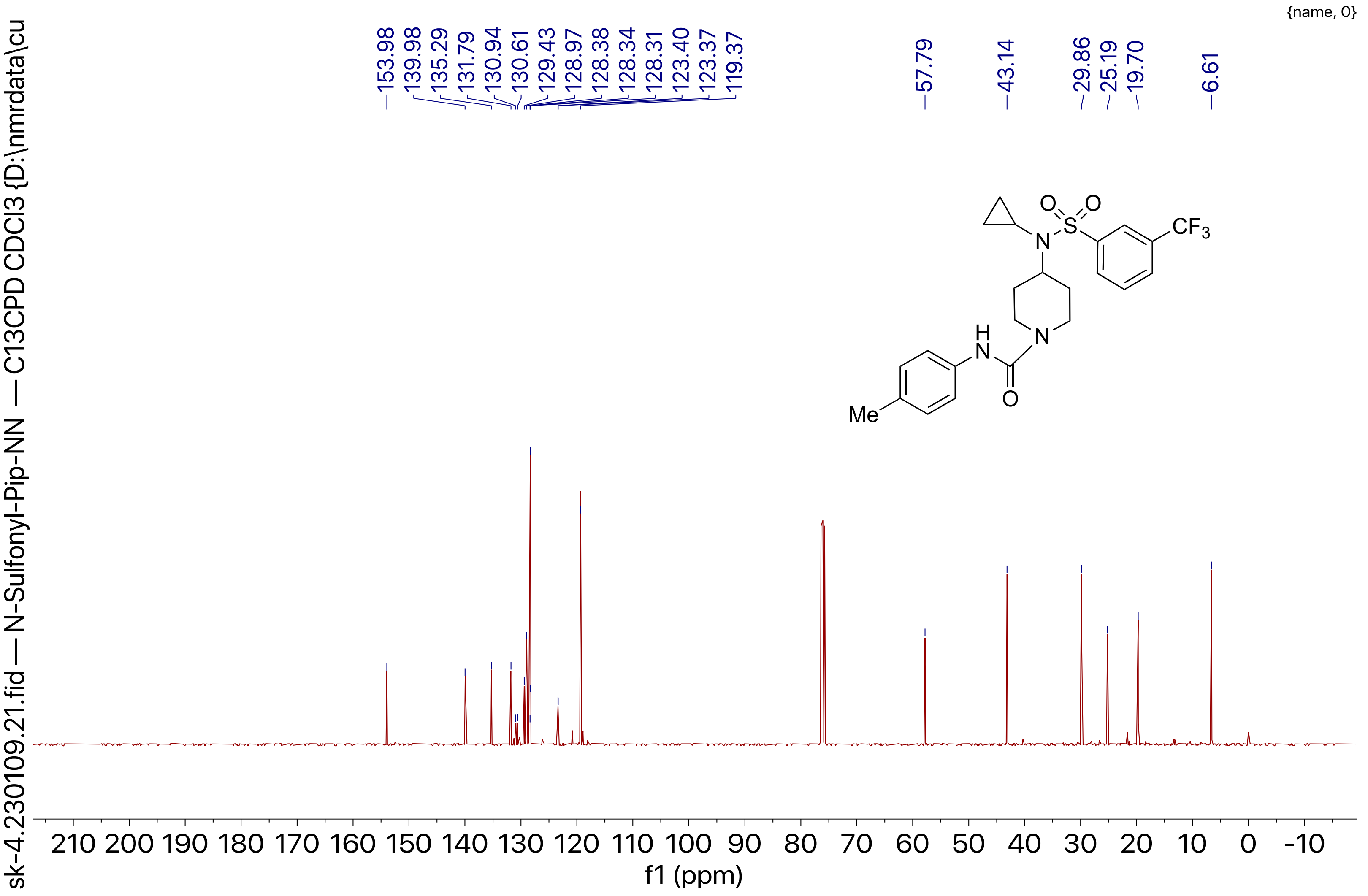

^{13}C NMR spectra of **5aa** (101 MHz, RT, $CDCl_3$)

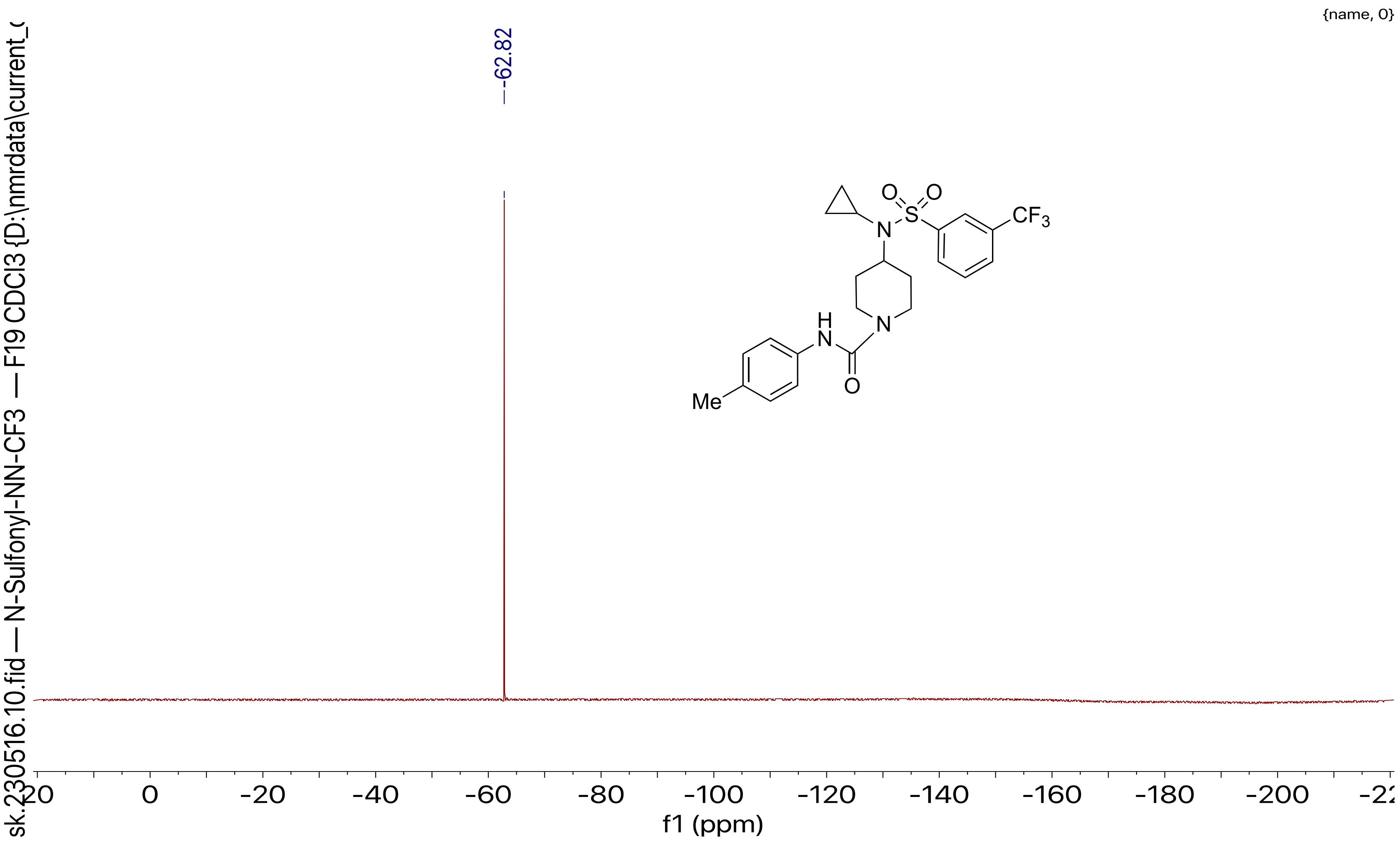

^{19}F NMR spectra of **5aa** (376 MHz, RT, $CDCl_3$)

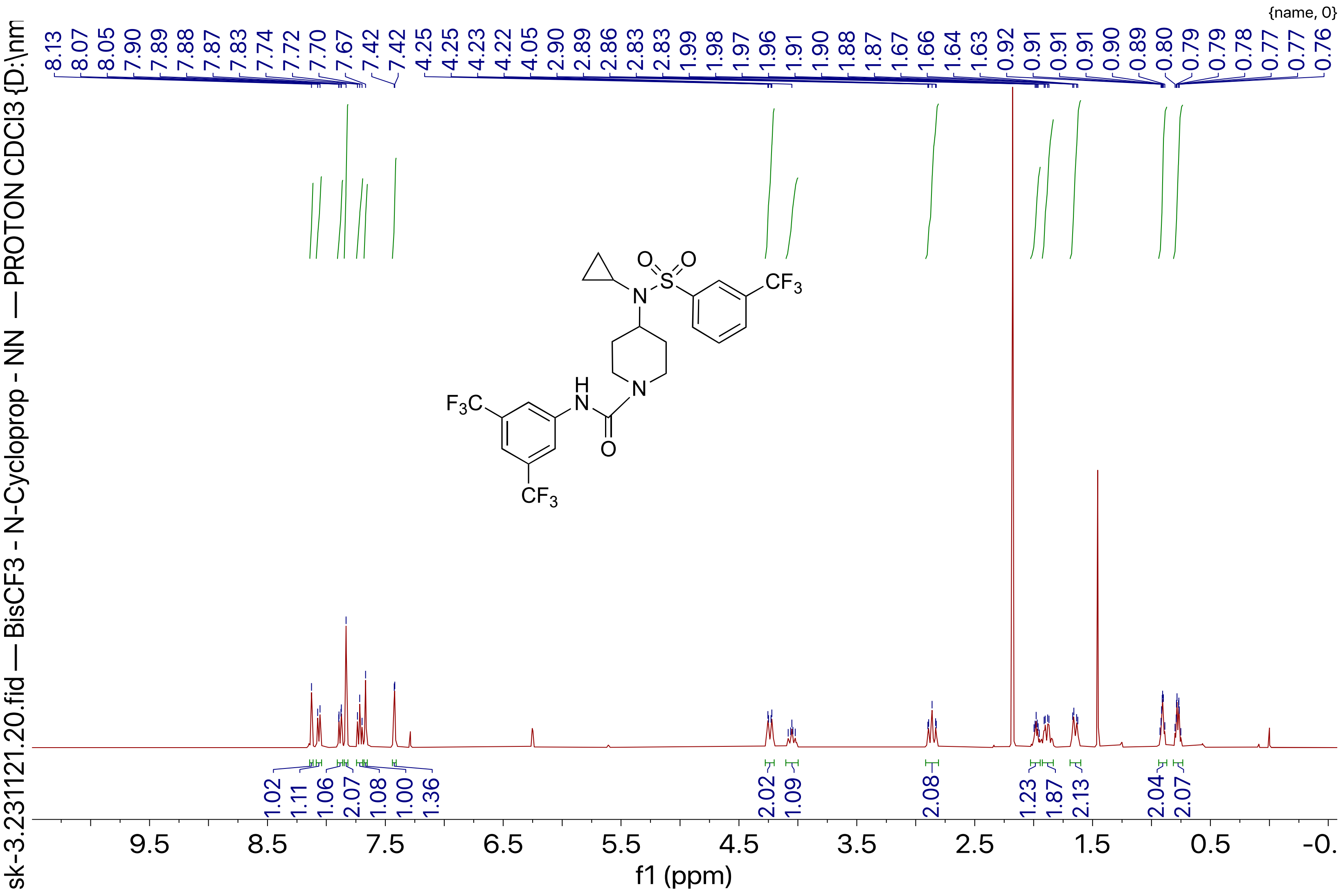

^{1}H NMR spectra of **5aa'** (400 MHz, RT, $CDCl_3$)

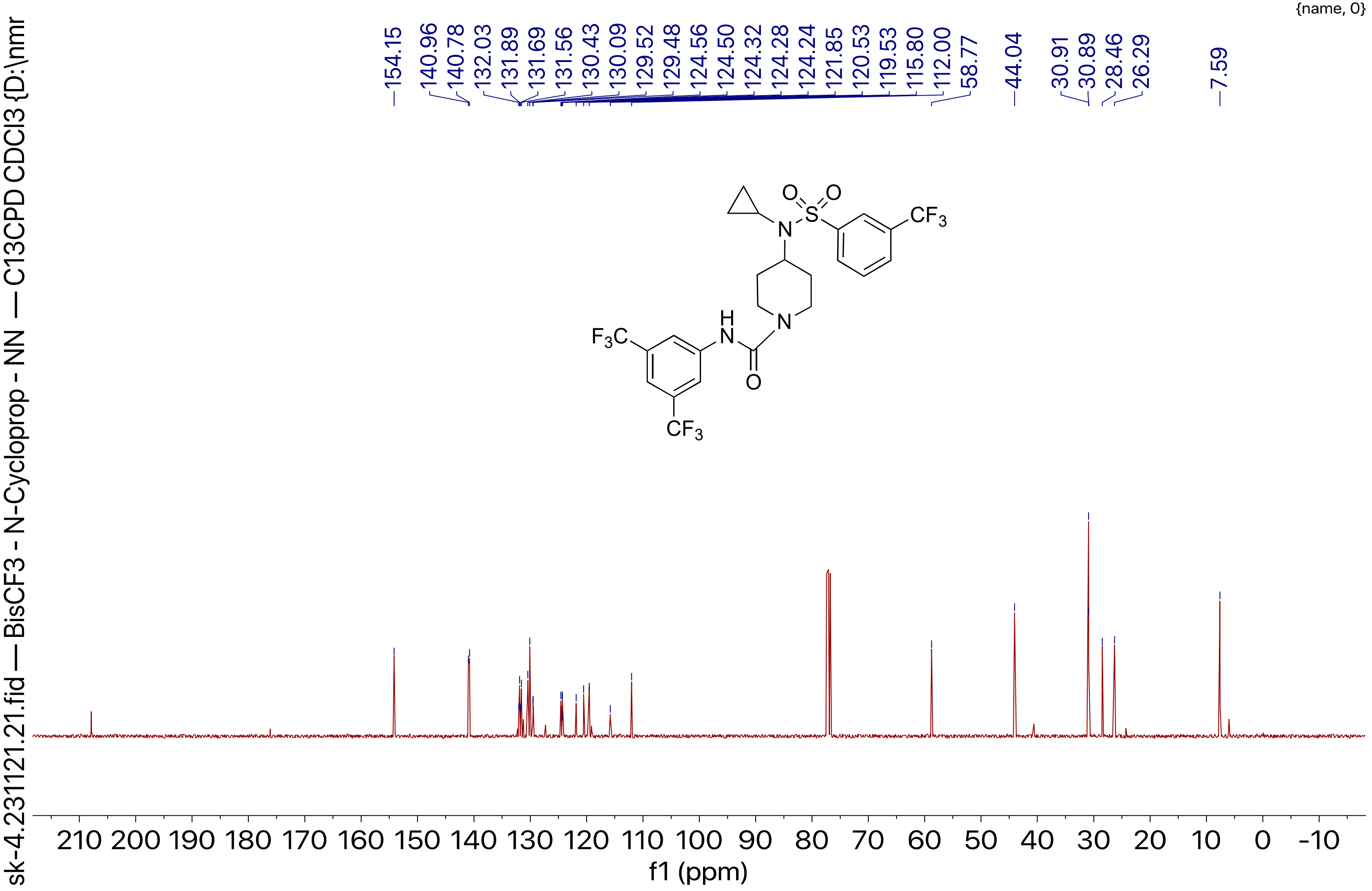

^{13}C NMR spectra of **5aa’** (101 MHz, RT, $CDCl_3$)

{name, 0}

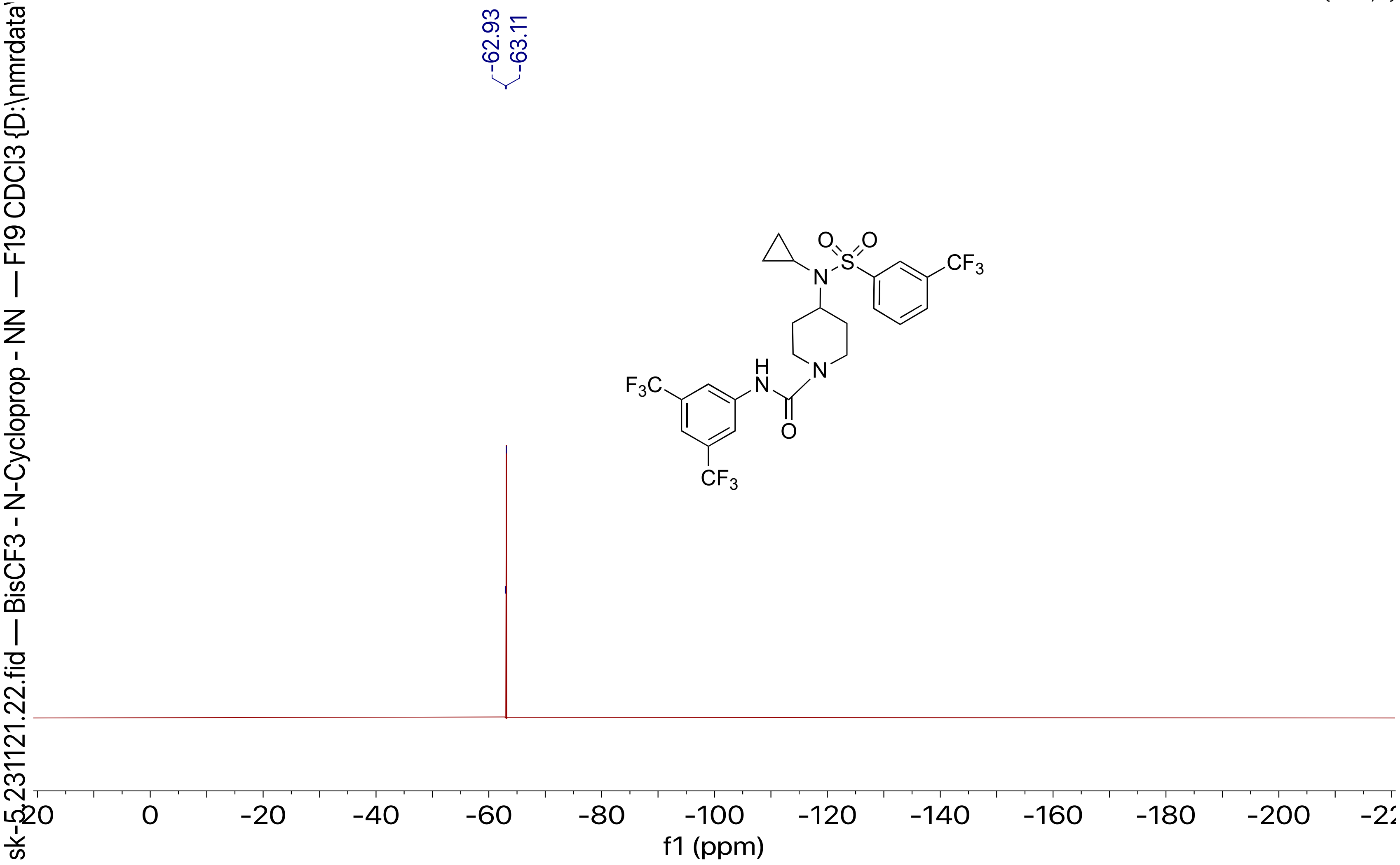

^{19}F NMR spectra of **5aa'** (376 MHz, RT, $CDCl_3$)

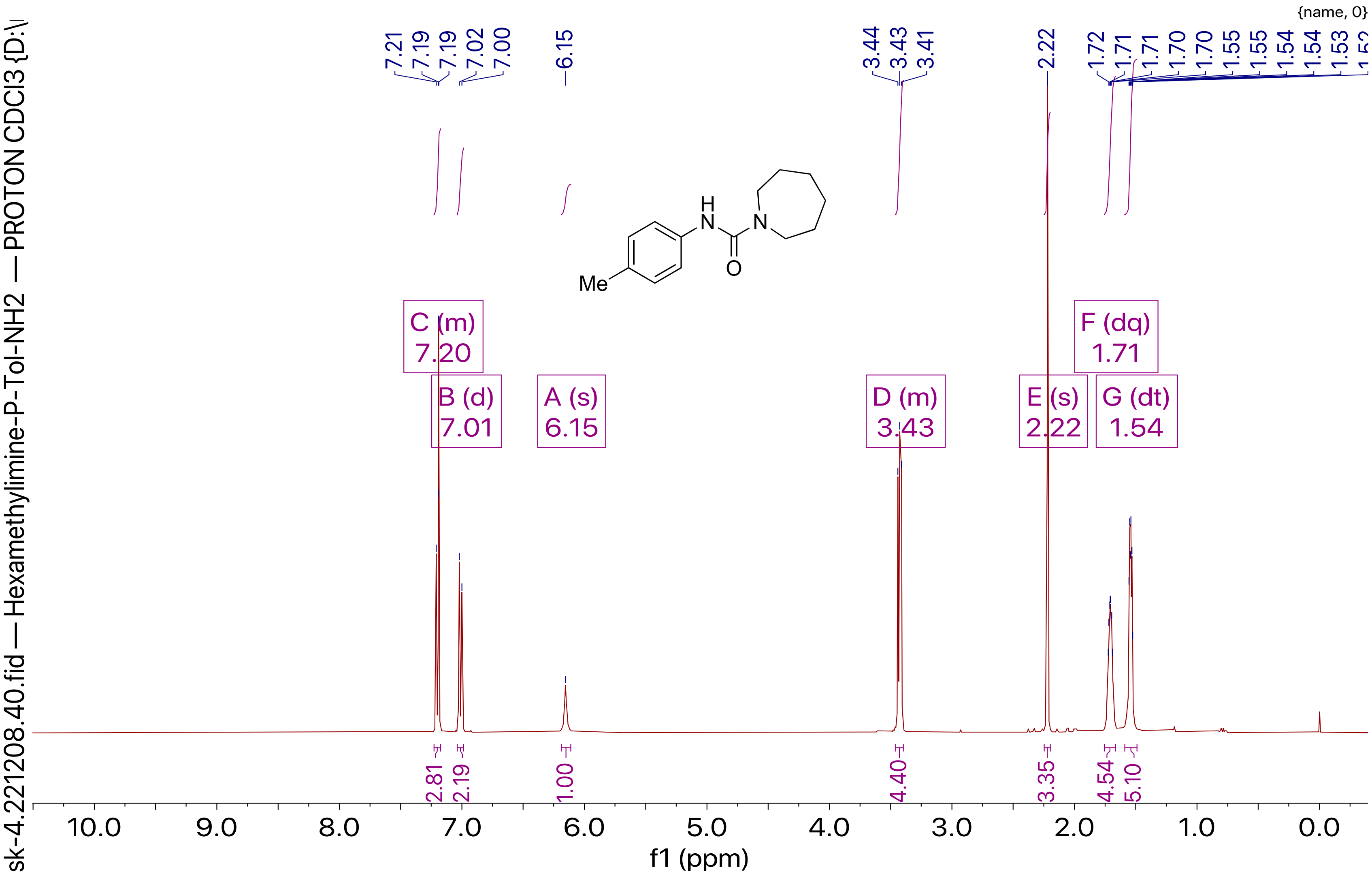

^{1}H NMR spectra of **5bb** (400 MHz, RT, $CDCl_3$)

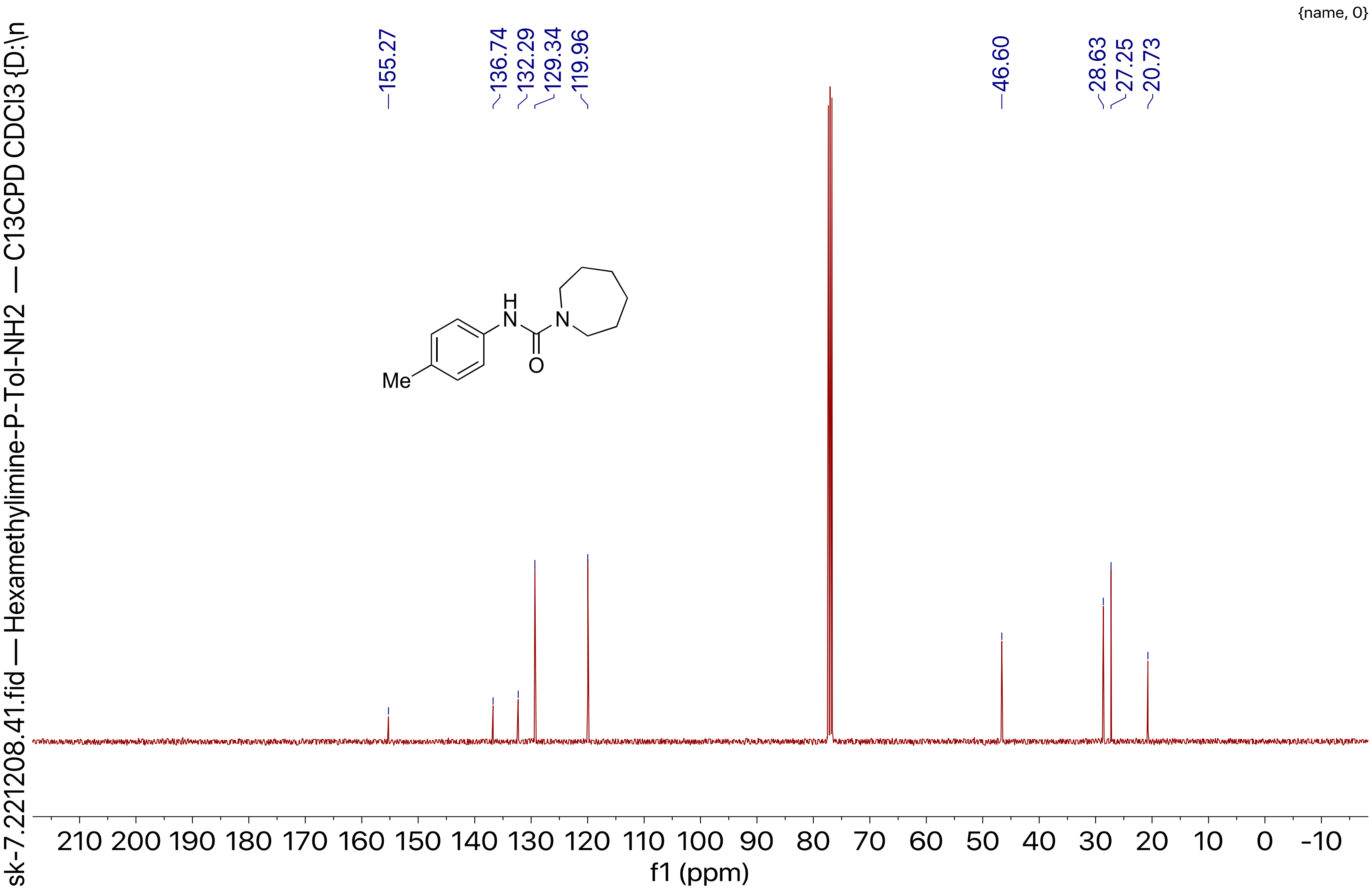

^{13}C NMR spectra of **5bb** (101 MHz, RT, $CDCl_3$)

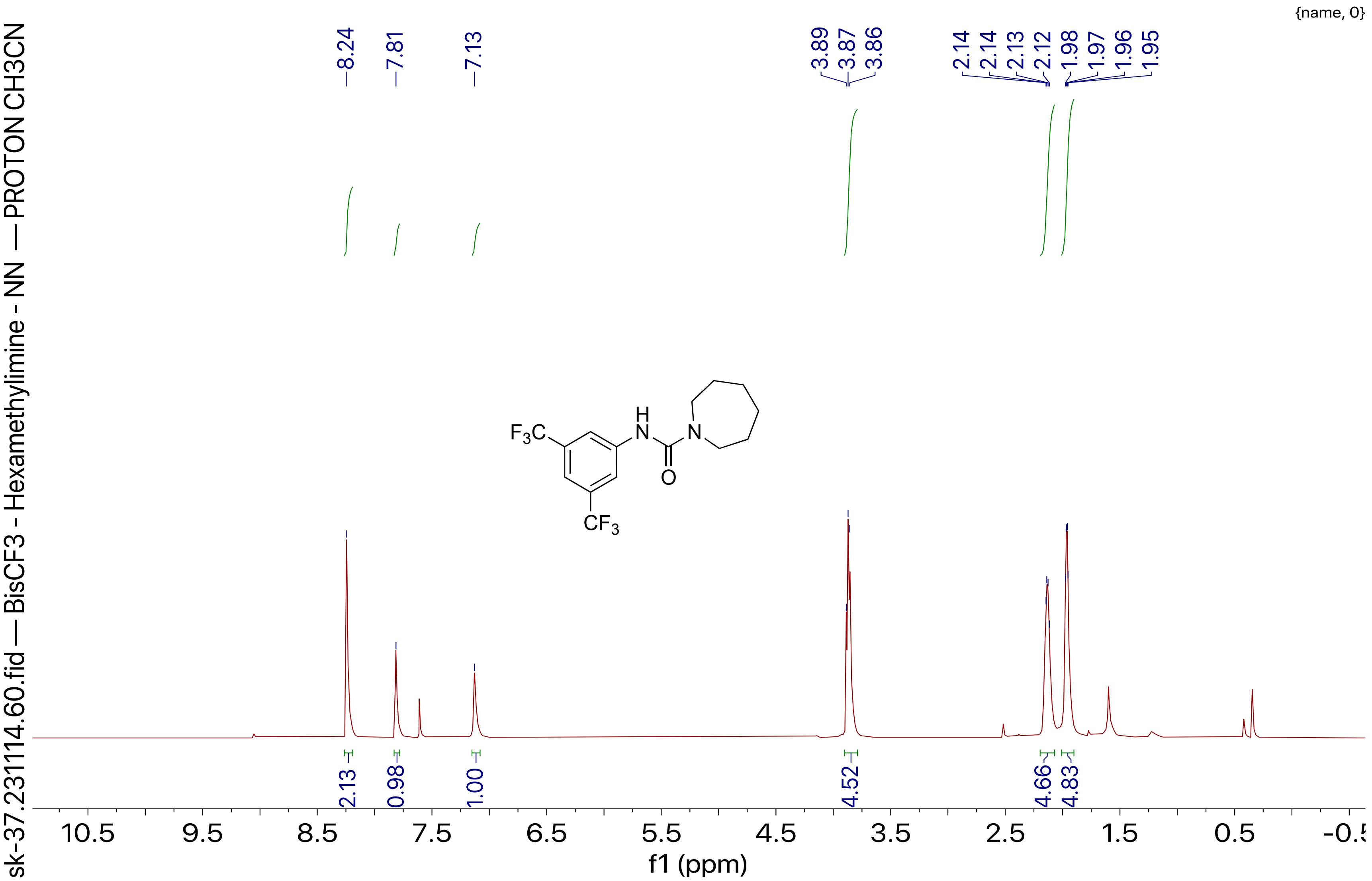

^{1}H NMR spectra of **5bb’** (400 MHz, RT, $CDCl_3$)

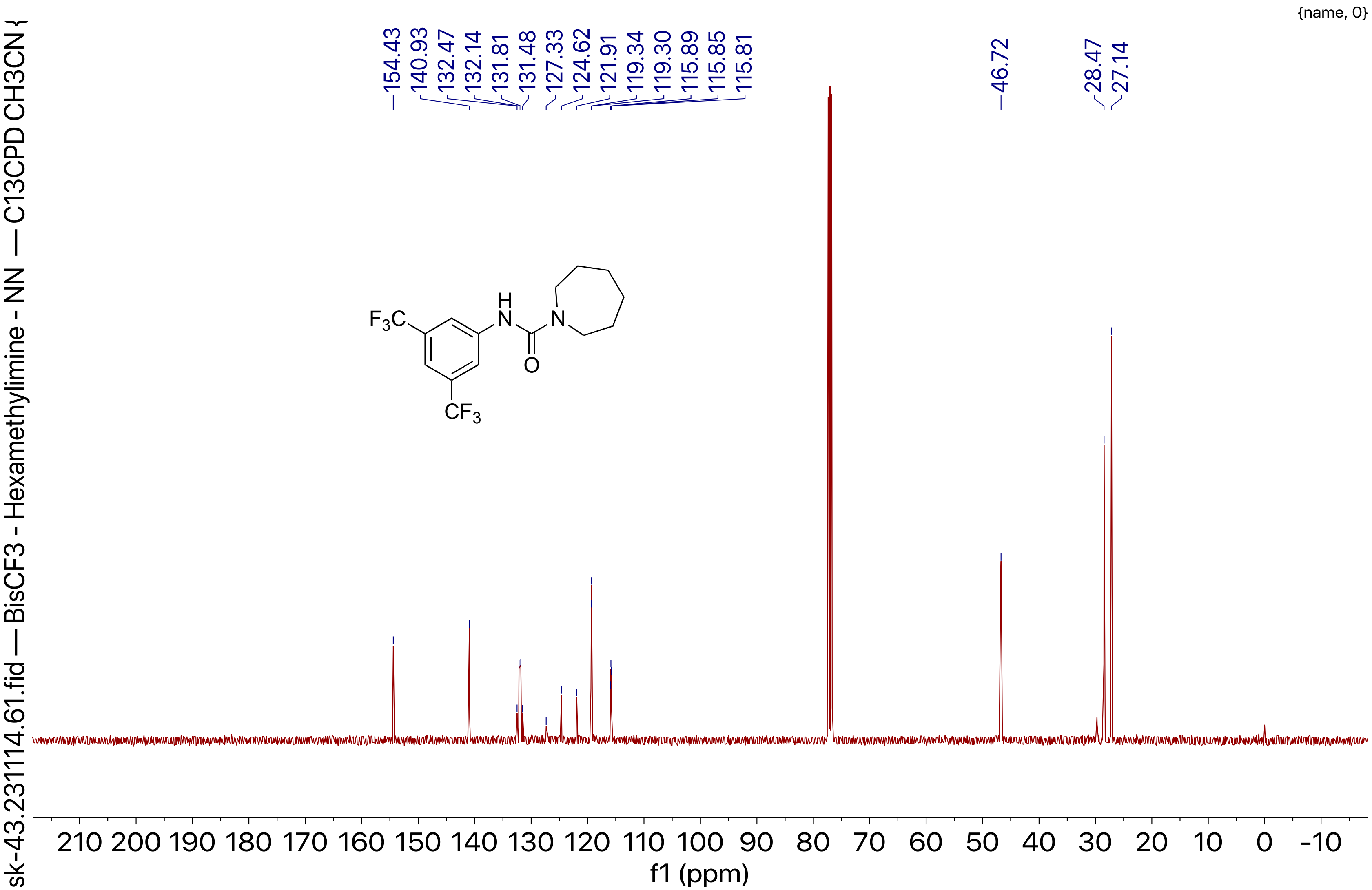

^{13}C NMR spectra of **5bb’** (101 MHz, RT, $CDCl_3$)

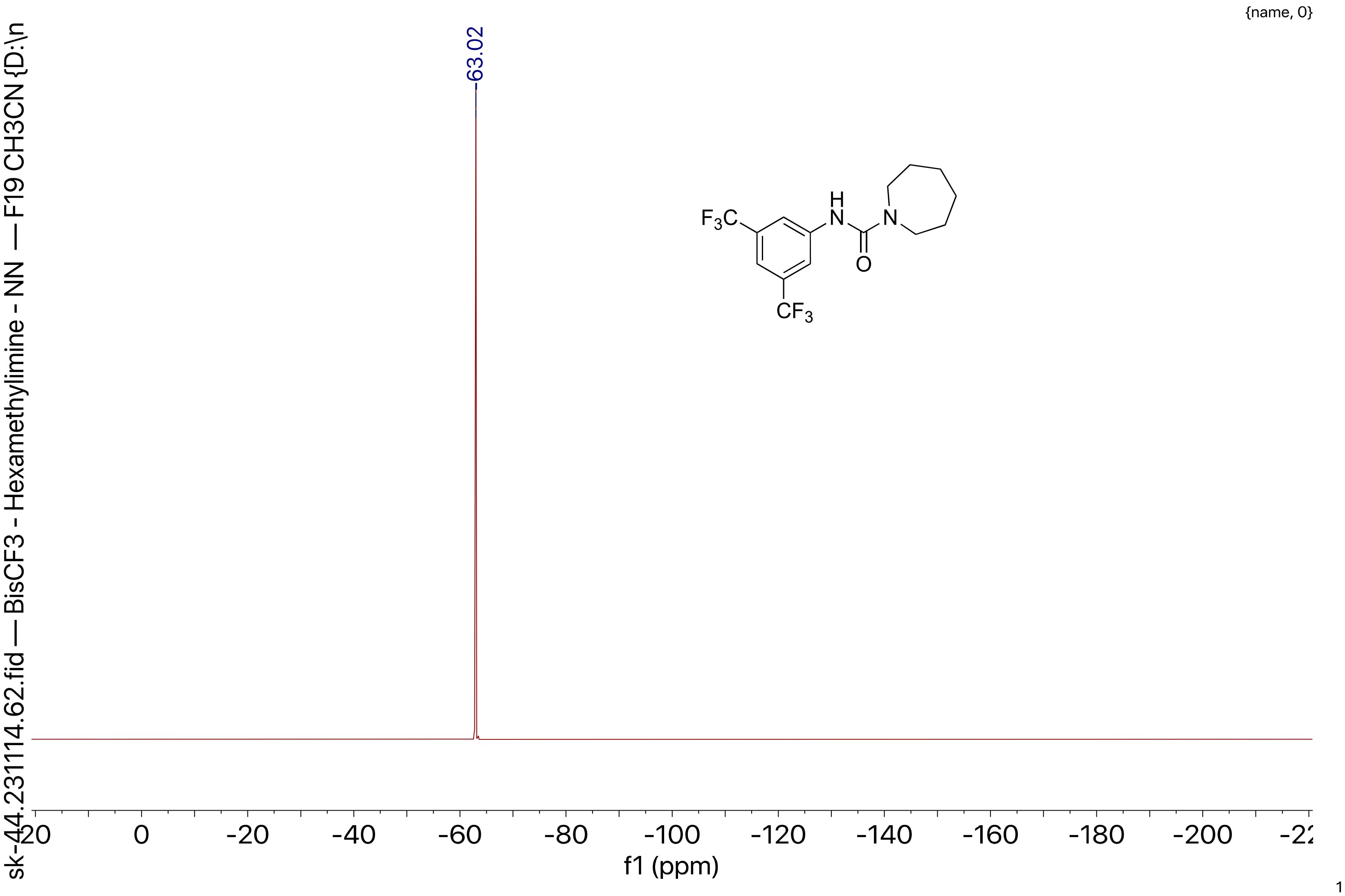

^{9}F NMR spectra of **5bb’** (376 MHz, RT, $CDCl_3$)

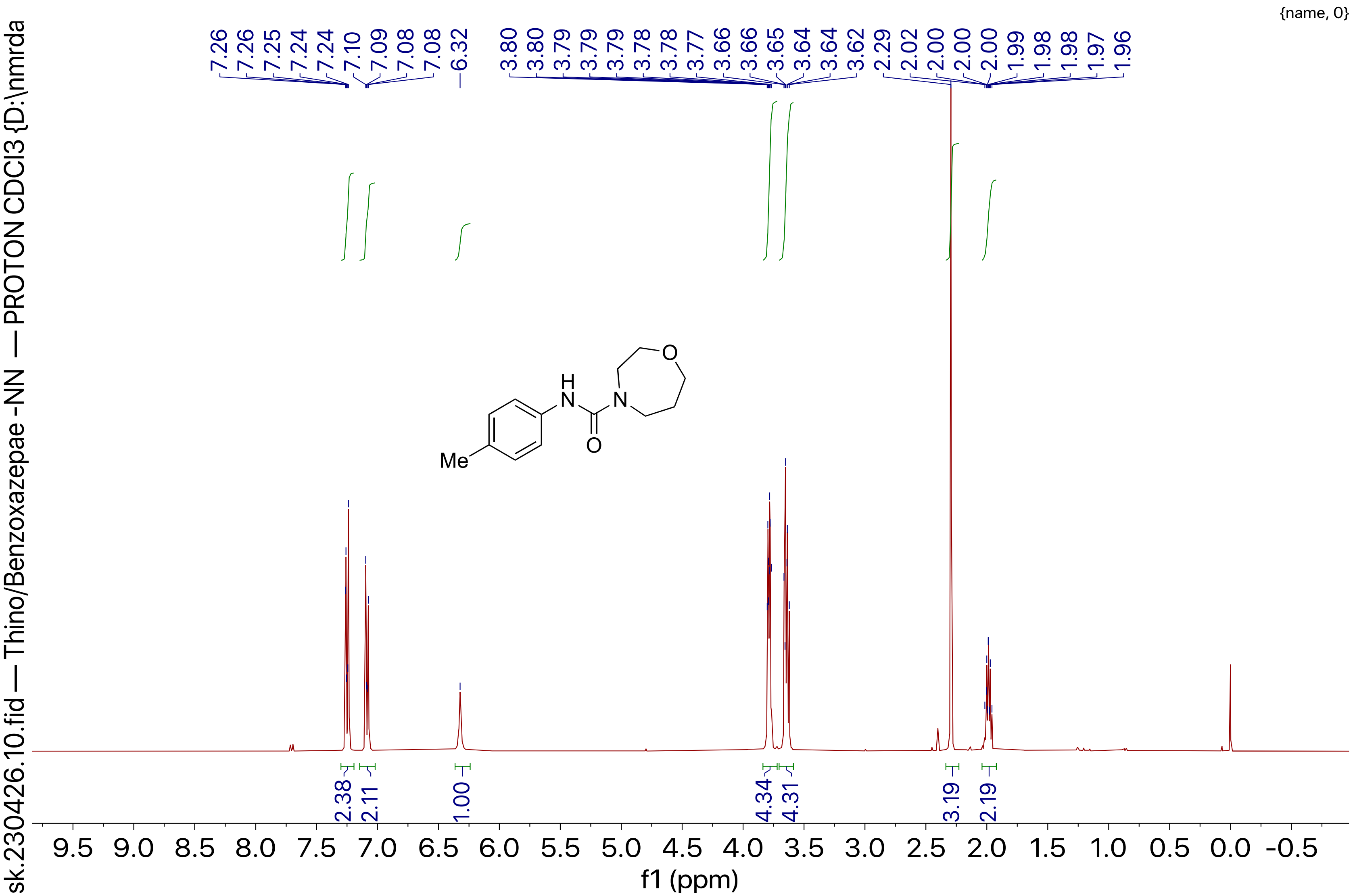

^{1}H NMR spectra of **5cc** (400 MHz, RT, $CDCl_3$)

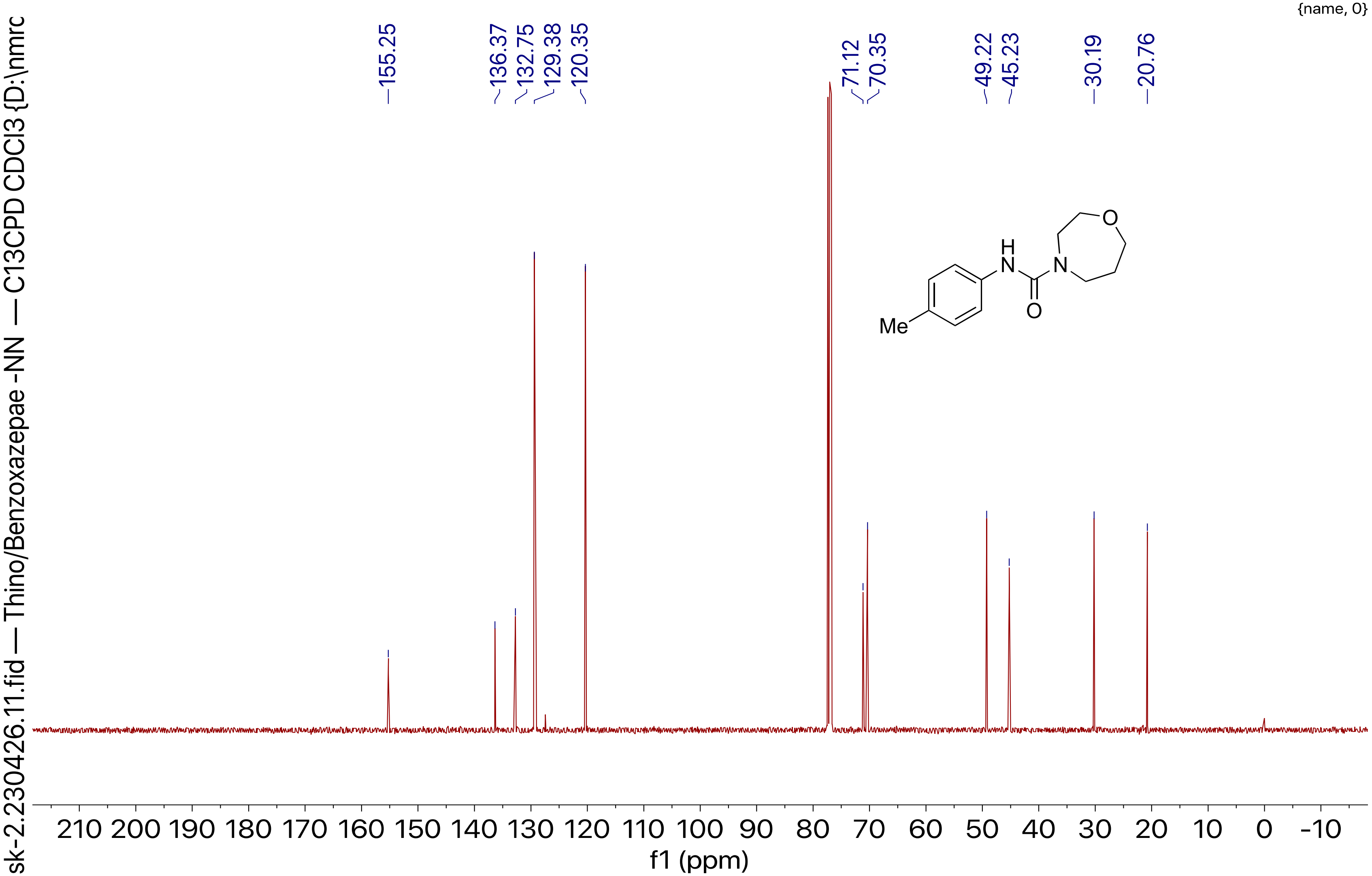

^{13}C NMR spectra of **5cc** (101 MHz, RT, $CDCl_3$)

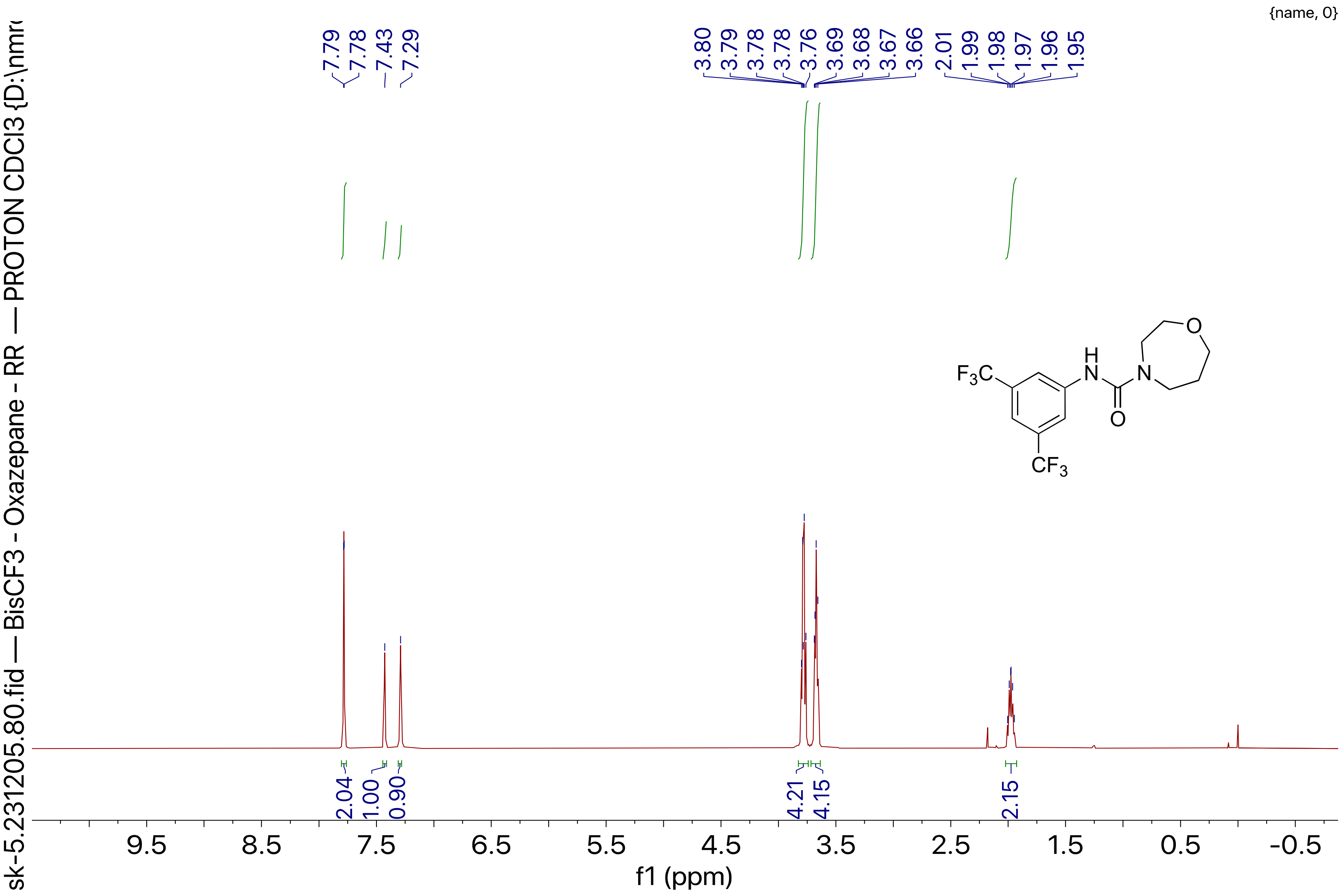

^{1}H NMR spectra of **5cc'** (400 MHz, RT, $CDCl_3$)

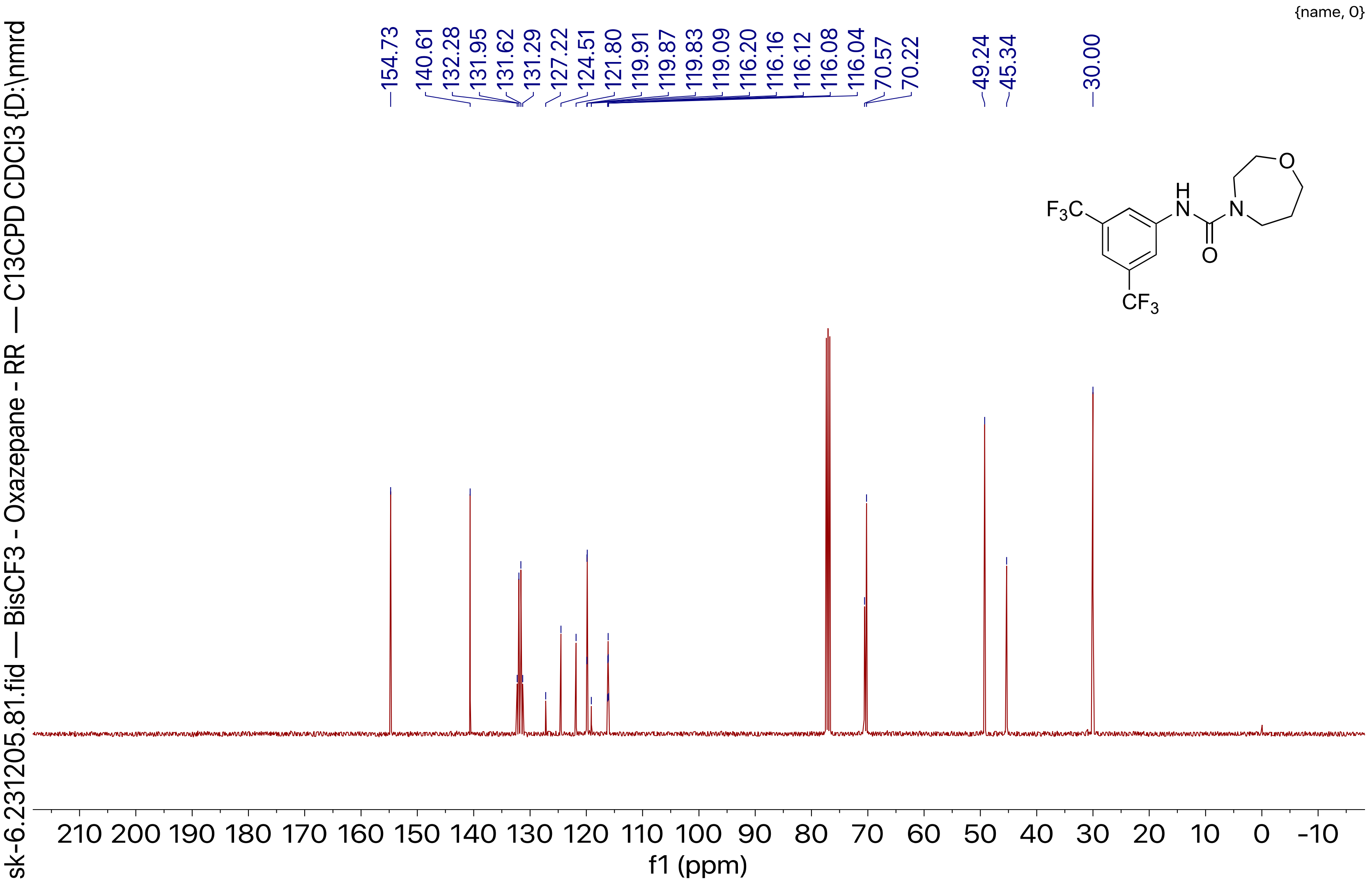

^{13}C NMR spectra of **5cc'** (101 MHz, RT, $CDCl_3$)

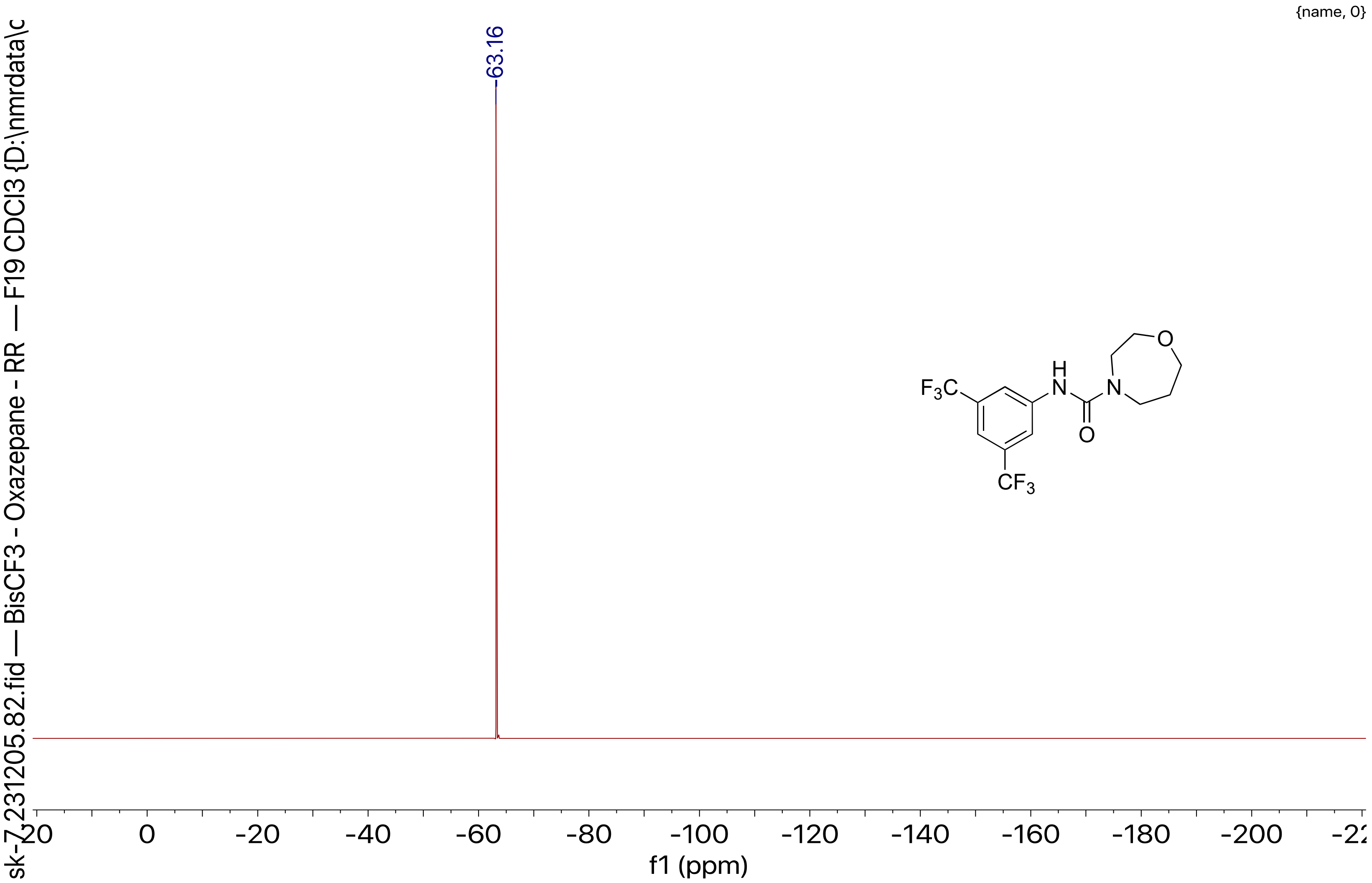

^{19}F NMR spectra of **5cc’** (376 MHz, RT, $CDCl_3$)

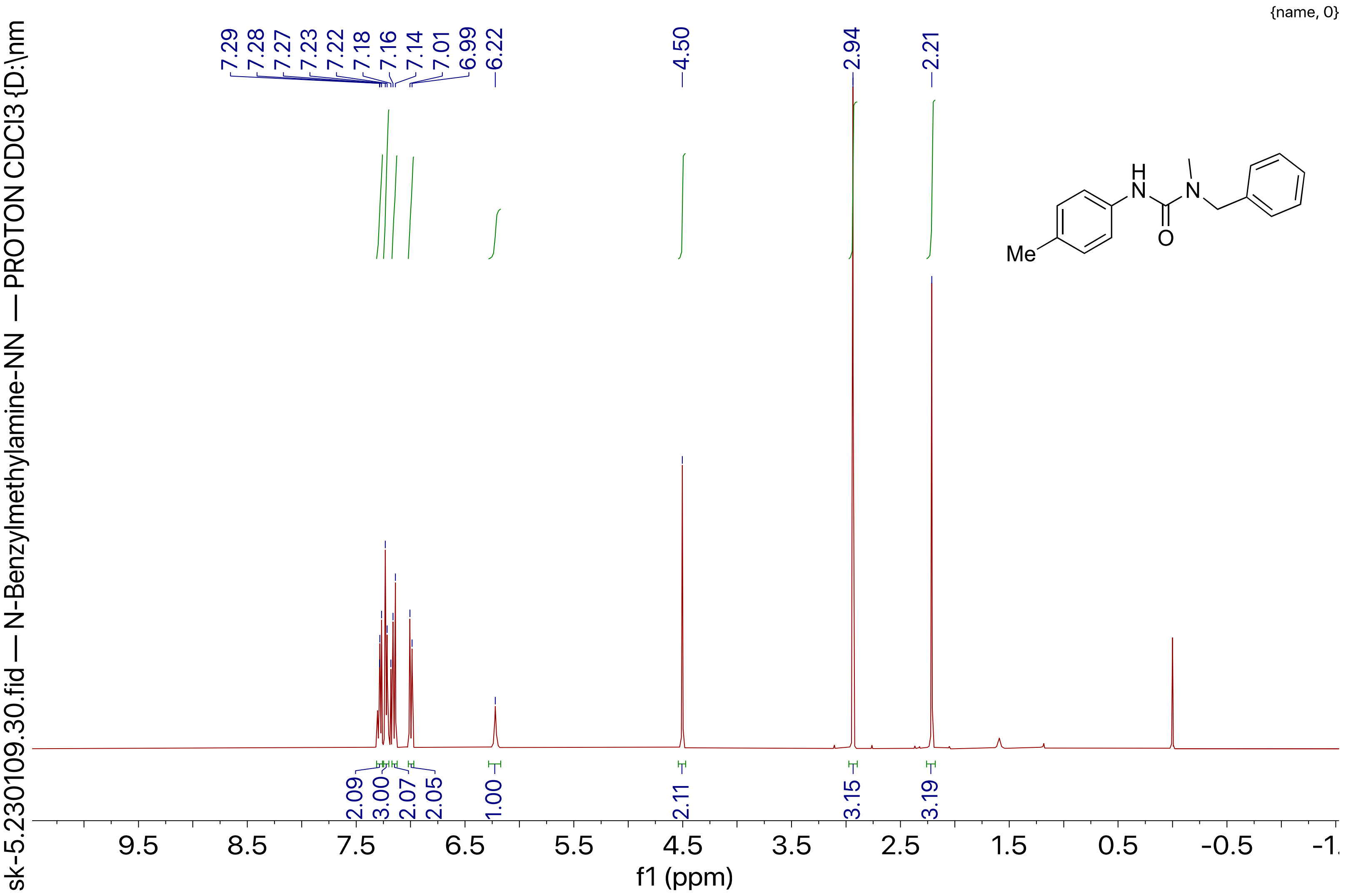

^{1}H NMR spectra of **5dd** (400 MHz, RT, $CDCl_3$)

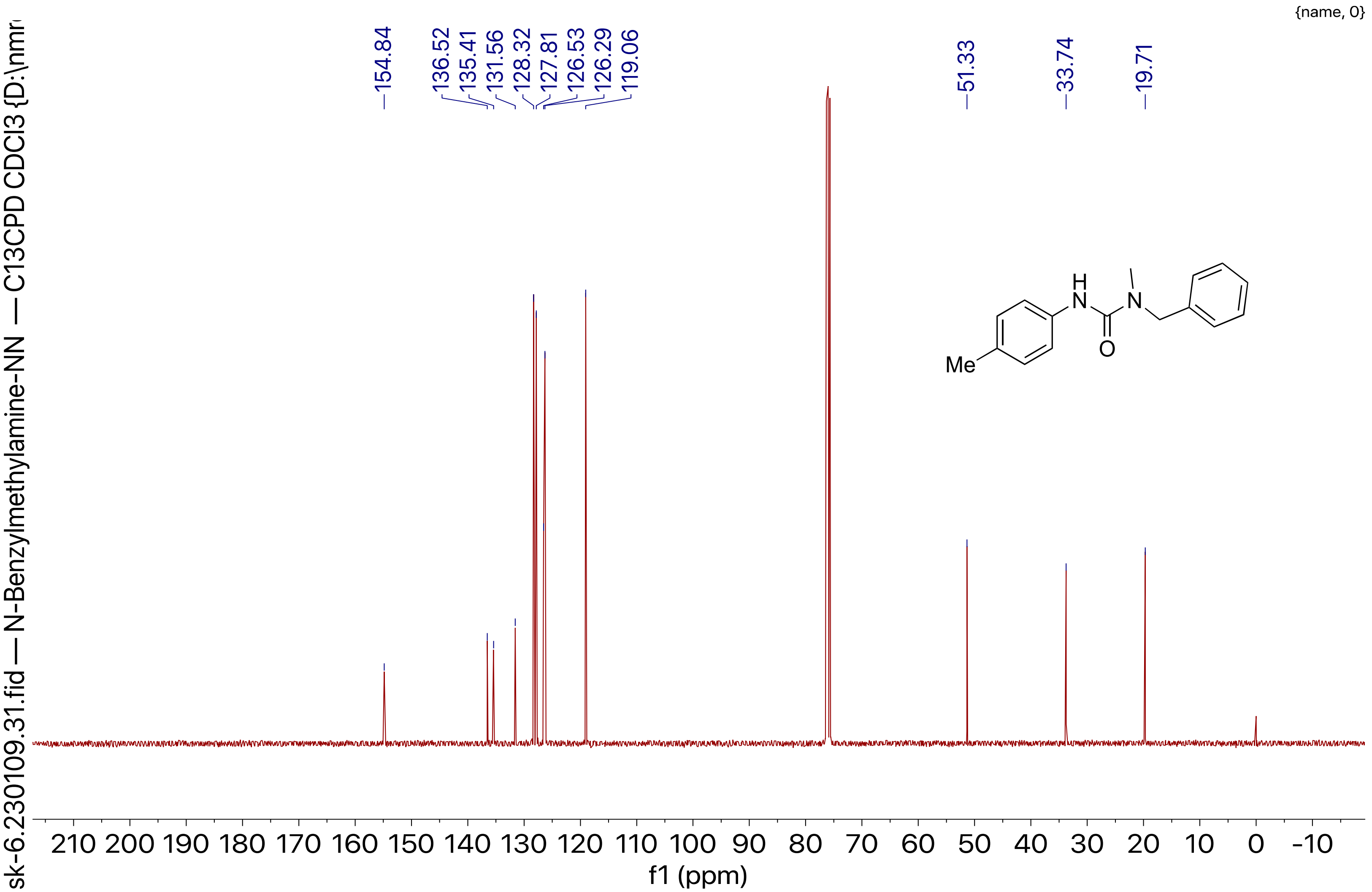

^{13}C NMR spectra of **5dd** (101 MHz, RT, $CDCl_3$)

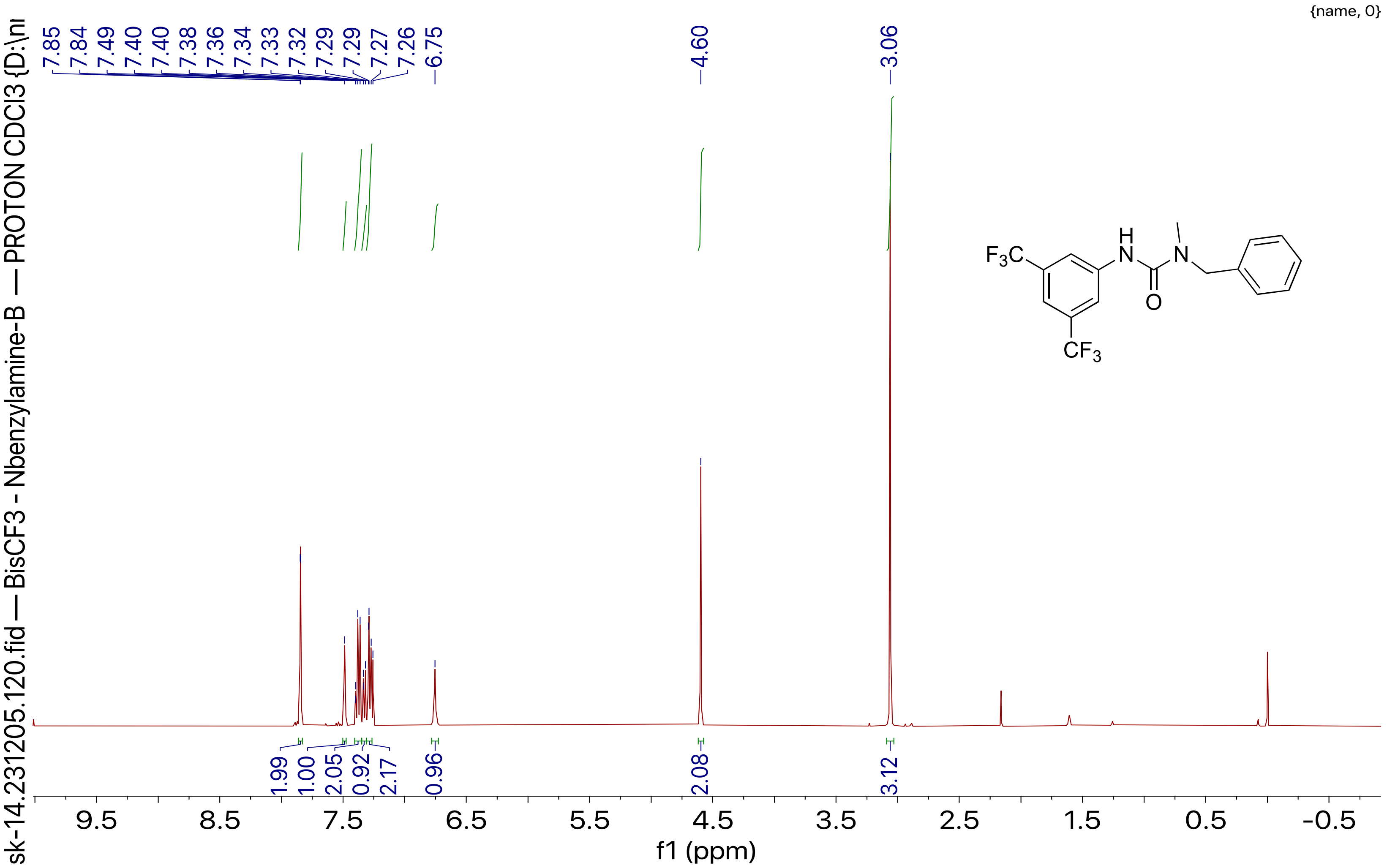

^{1}H NMR spectra of **5dd’** (400 MHz, RT, $CDCl_3$)

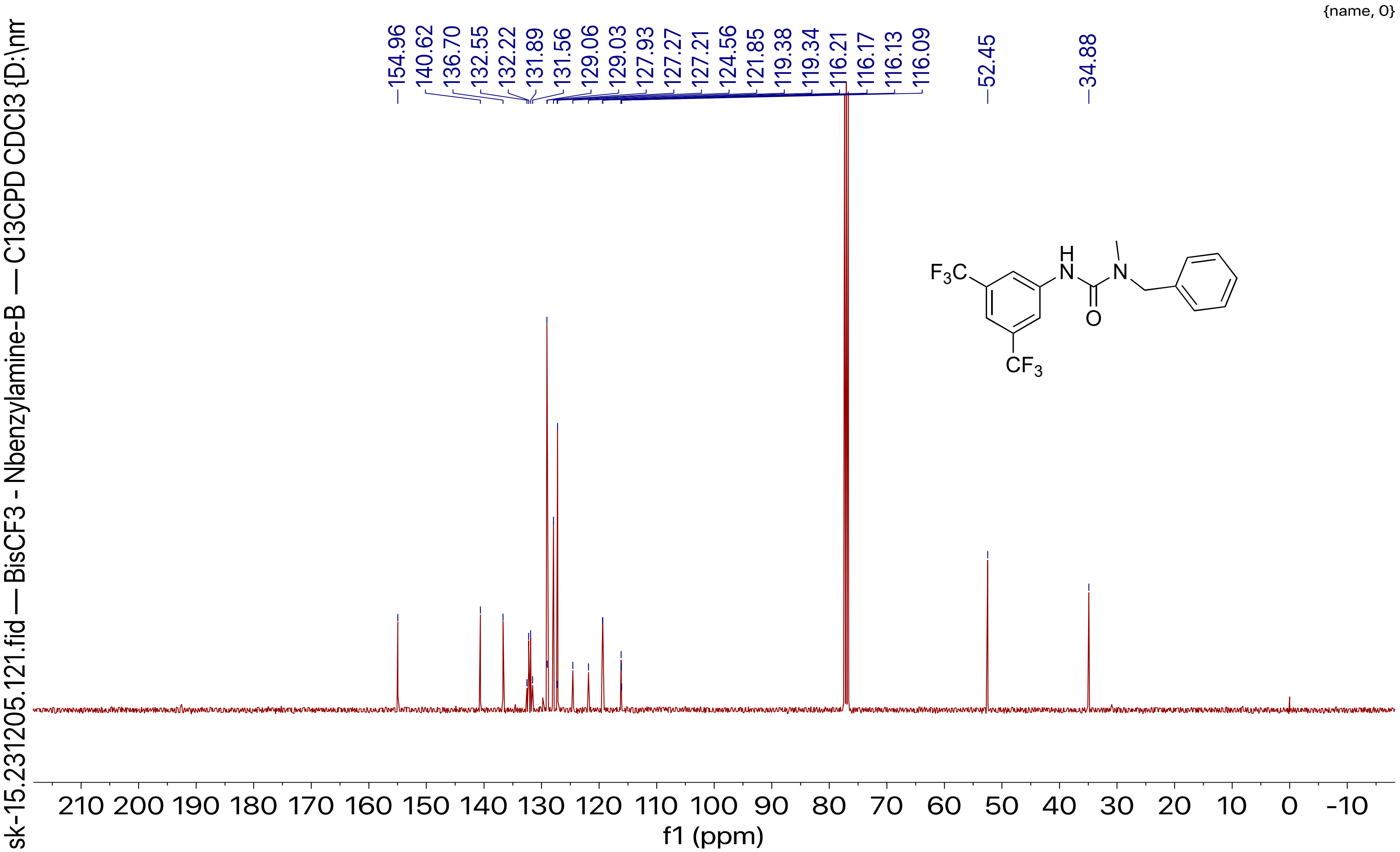

^{13}C NMR spectra of **5dd’** (101 MHz, RT, $CDCl_3$)

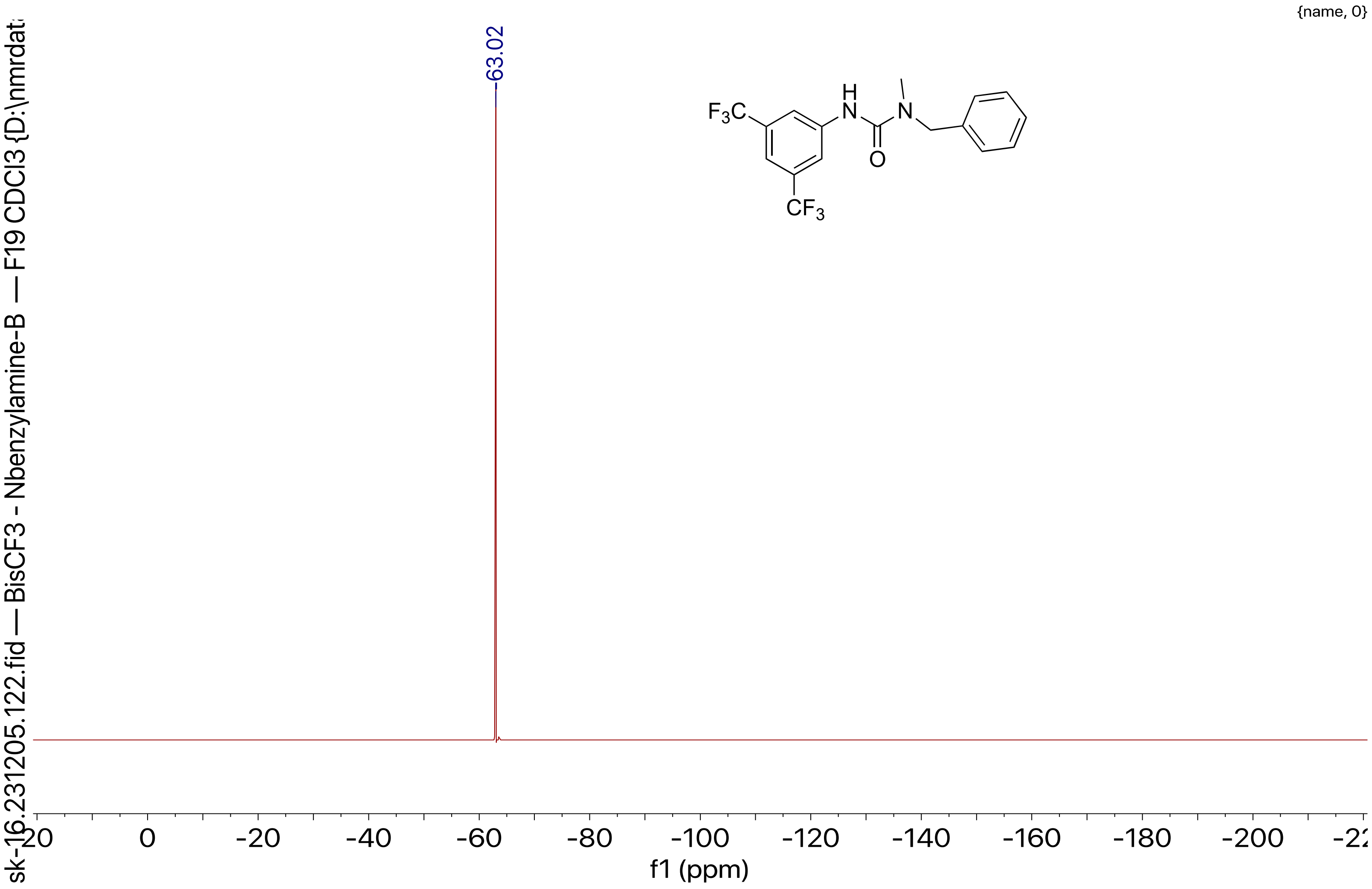

^{19}F NMR spectra of **5dd’** (376 MHz, RT, $CDCl_3$)

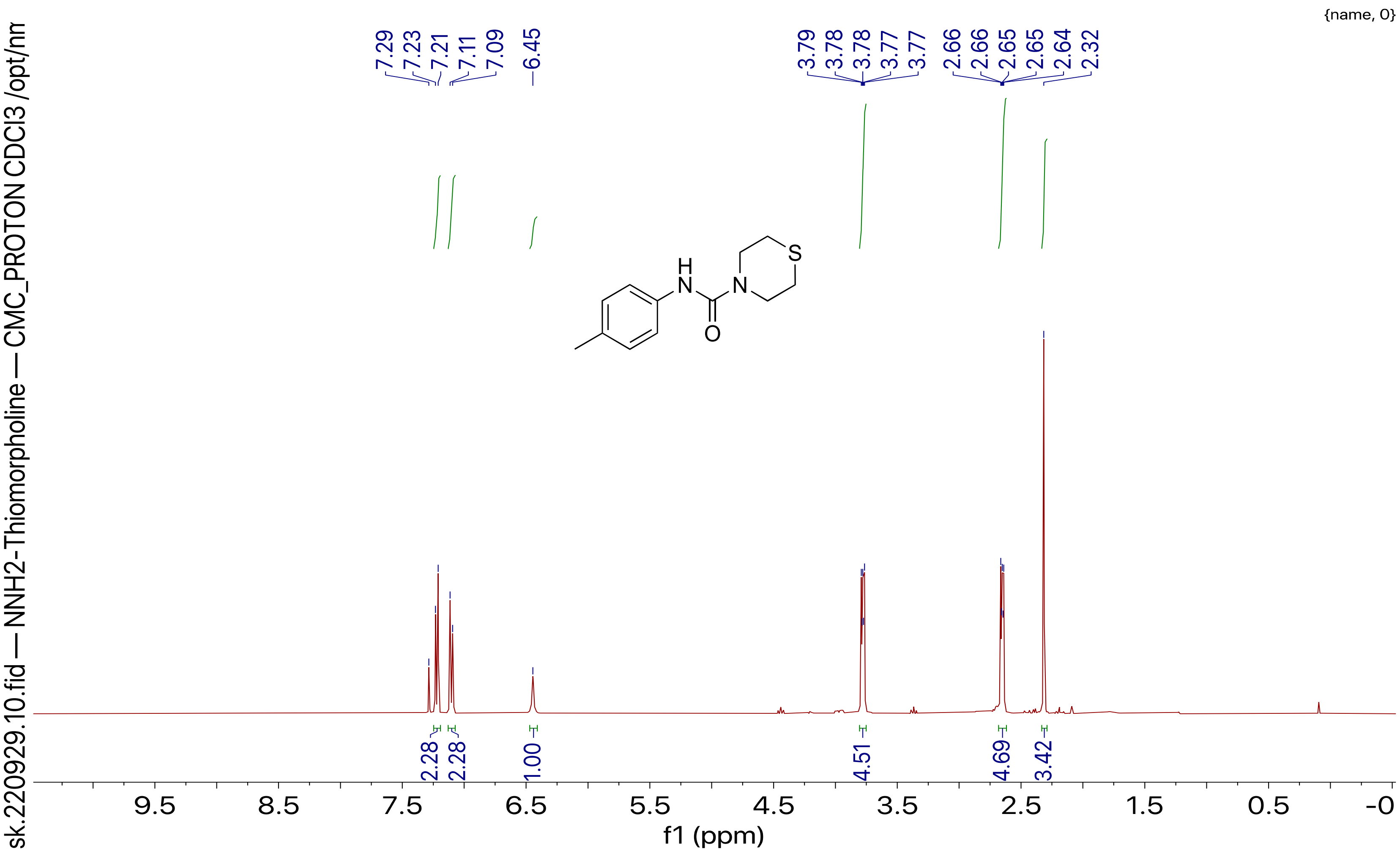

^{1}H NMR spectra of **7a** (400 MHz, RT, $CDCl_3$)

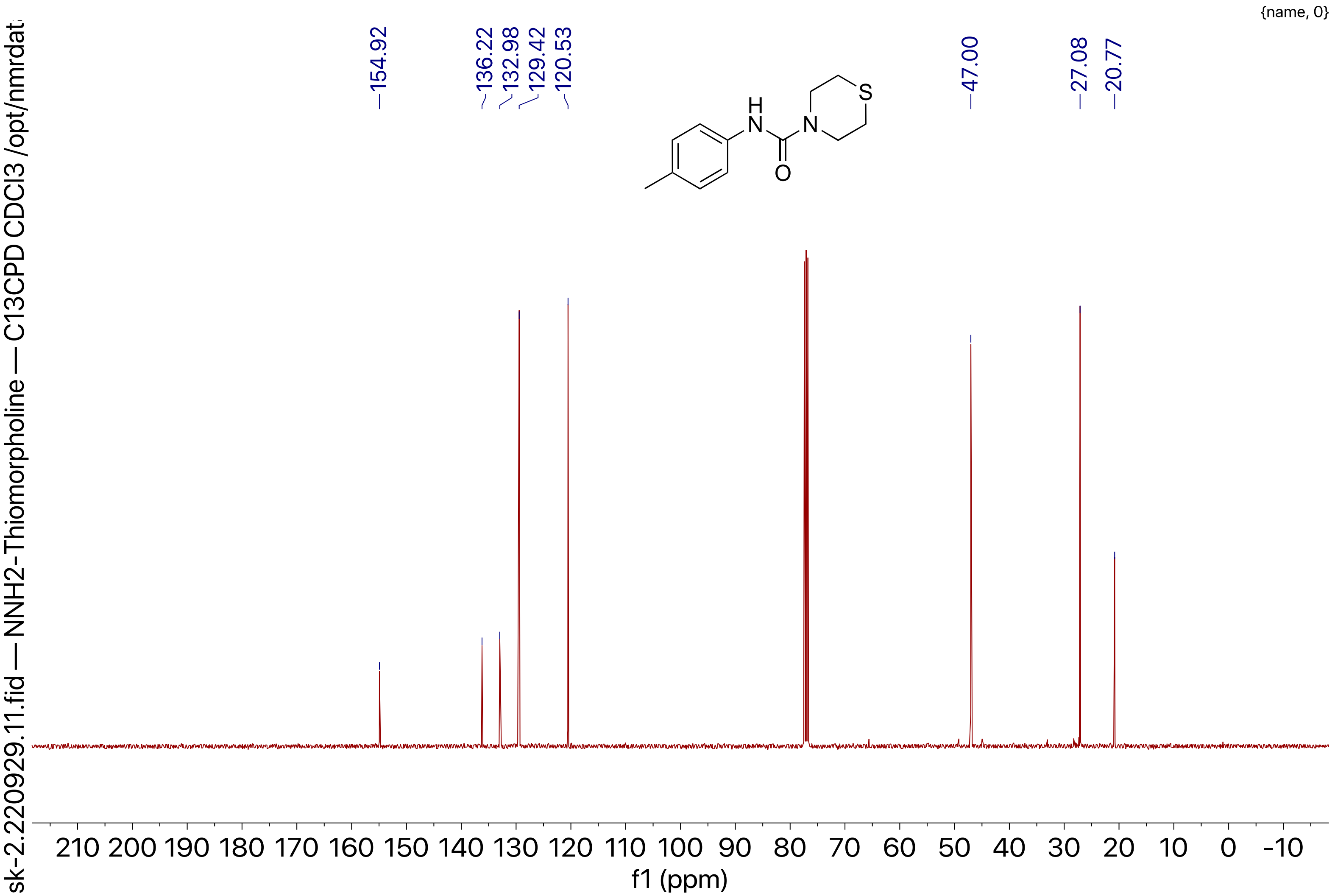

^{13}C NMR spectra of **7a** (101 MHz, RT, $CDCl_3$)

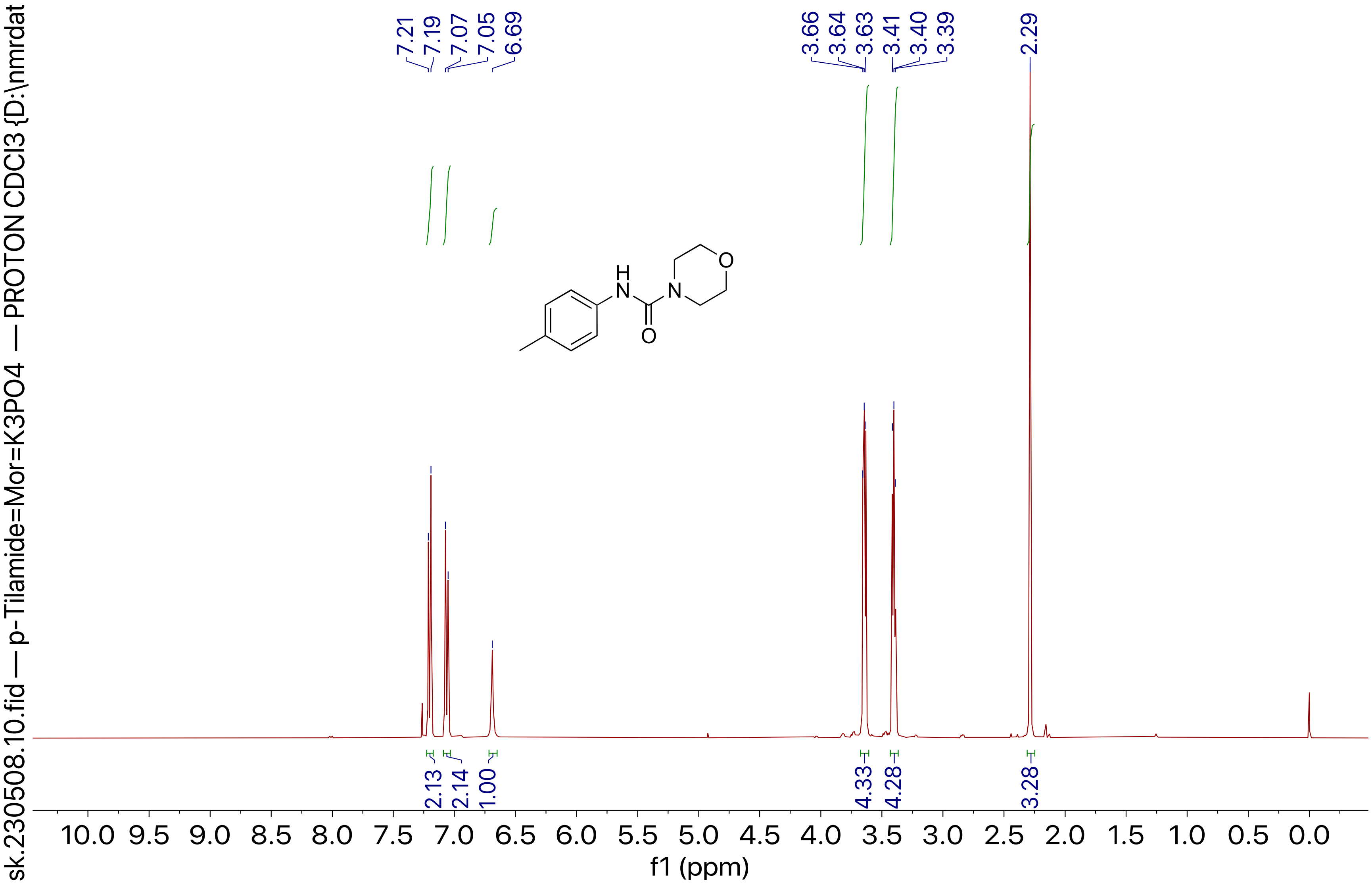

^{1}H NMR spectra of **7a’** (400 MHz, RT, $CDCl_3$)

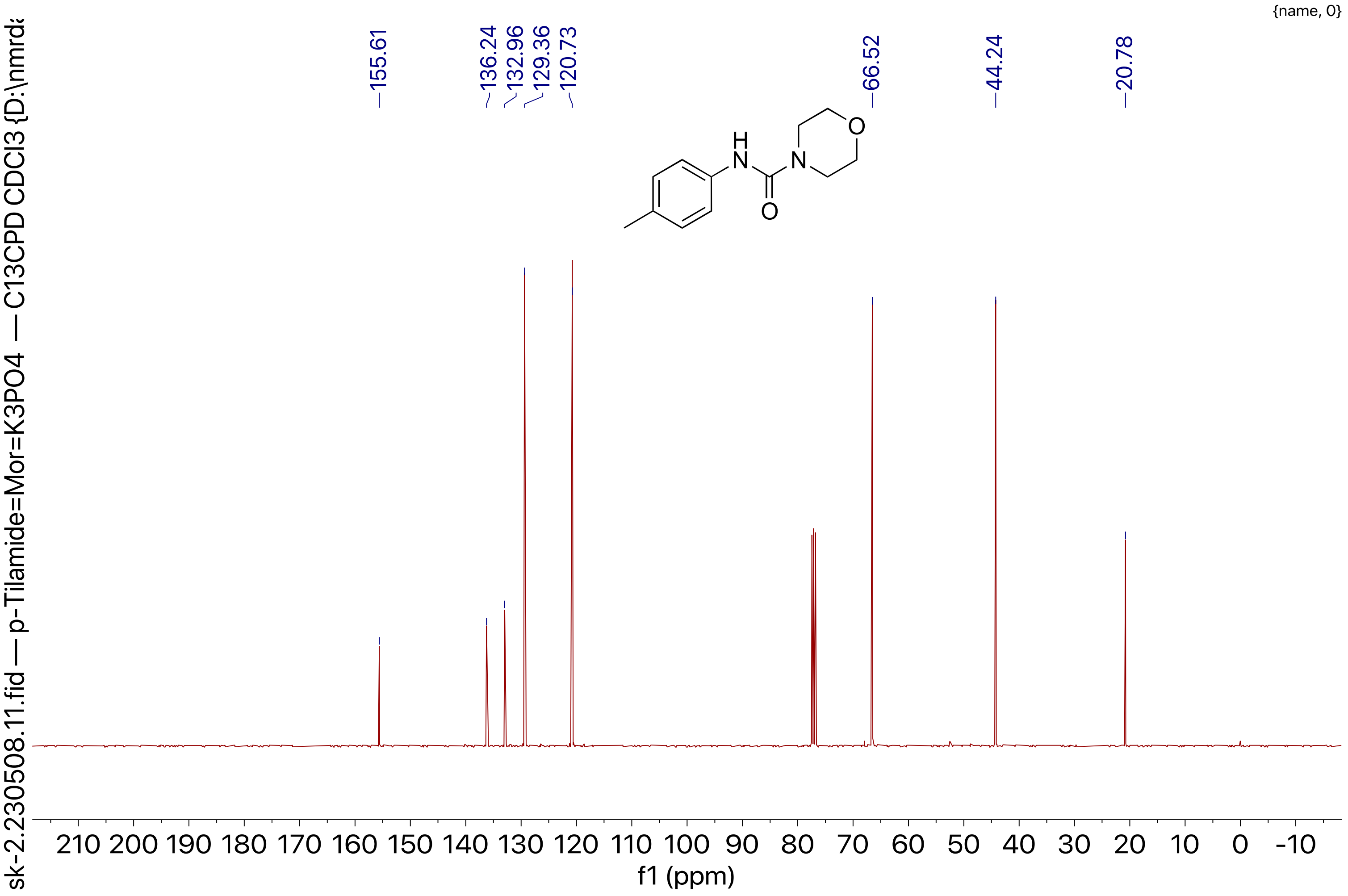

^{13}C NMR spectra of **7a'** (100 MHz, RT, $CDCl_3$)

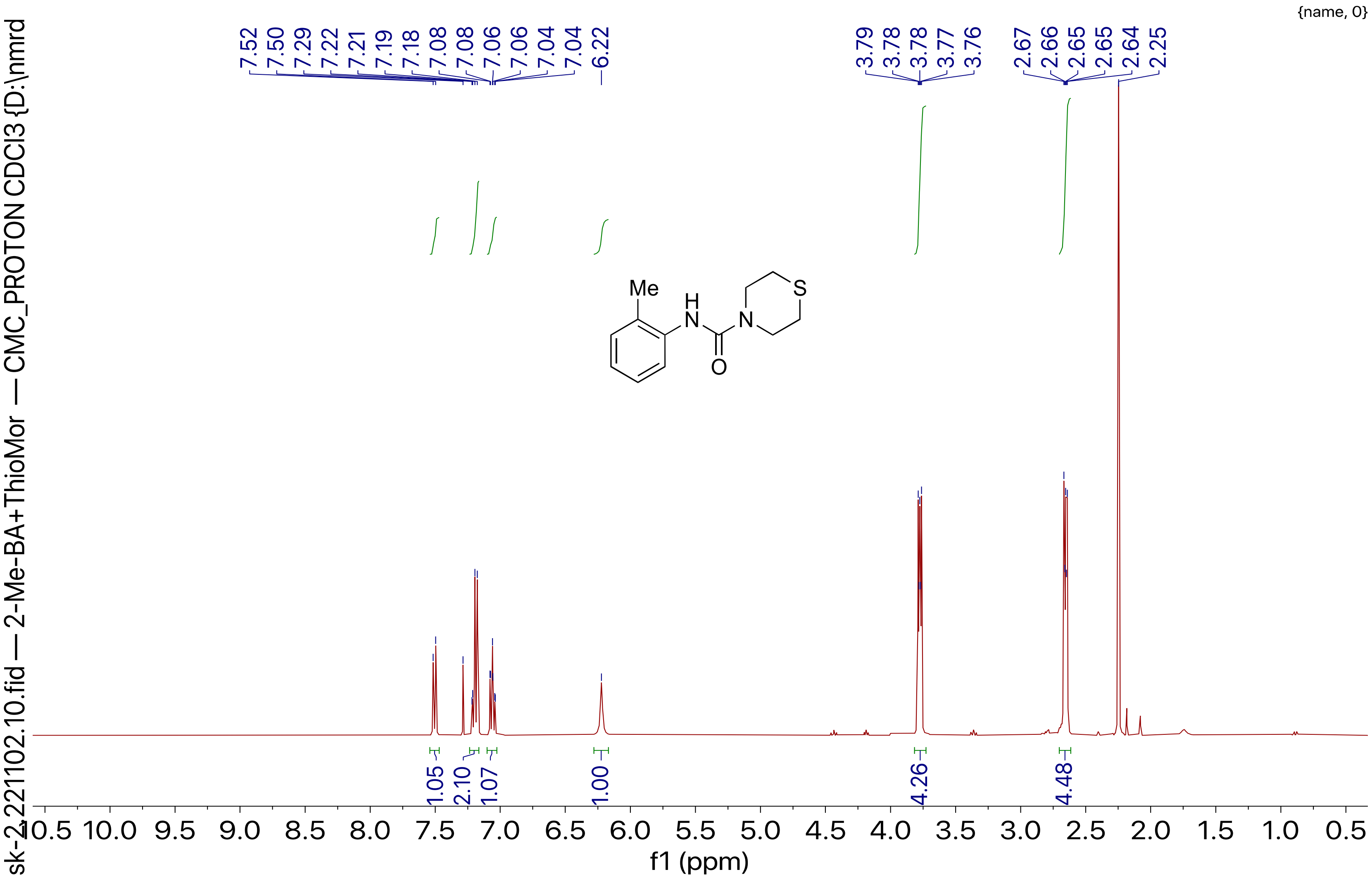

^{1}H NMR spectra of **7b** (400 MHz, RT, $CDCl_3$)

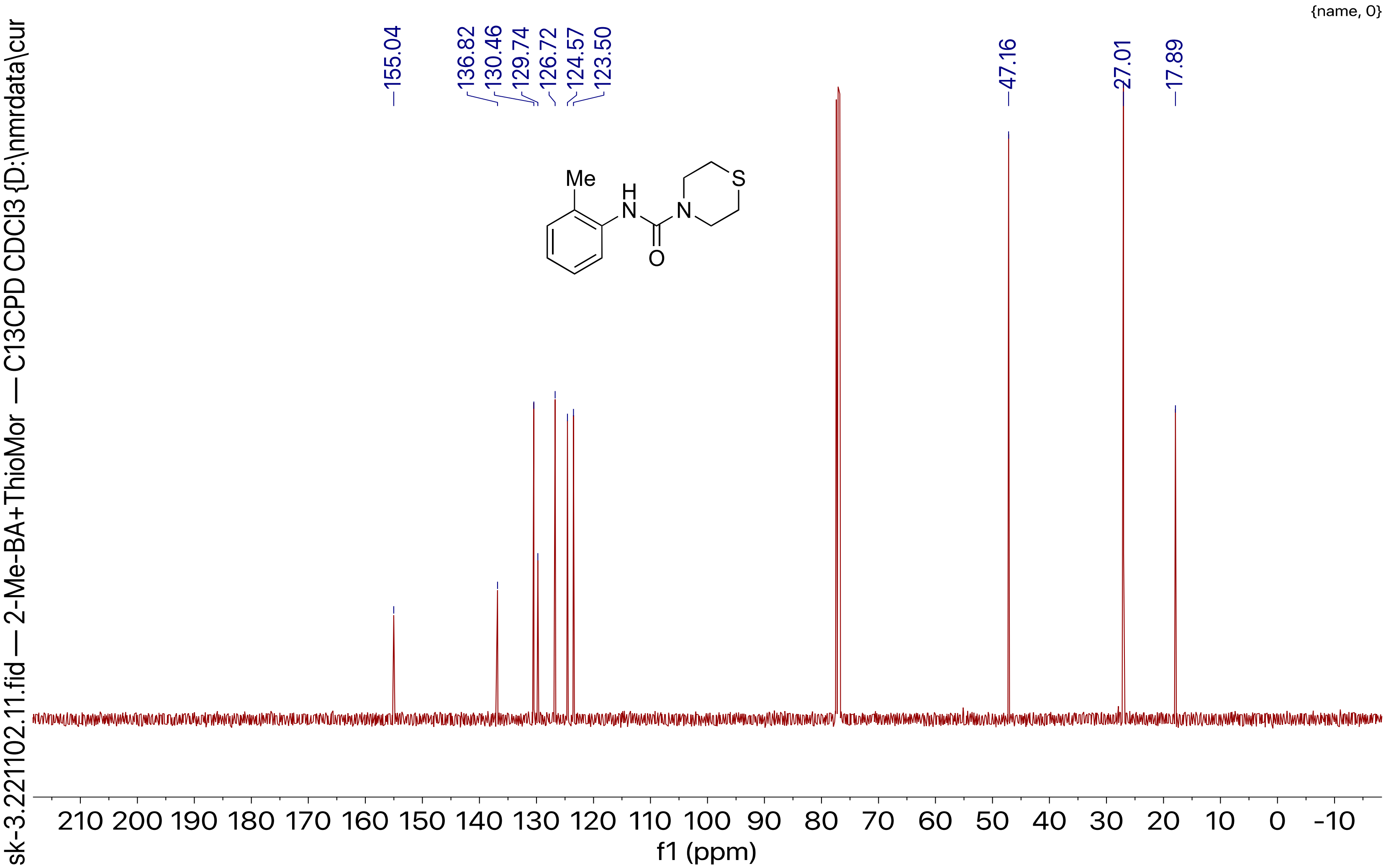

^{13}C NMR spectra of **7b** (101 MHz, RT, $CDCl_3$)

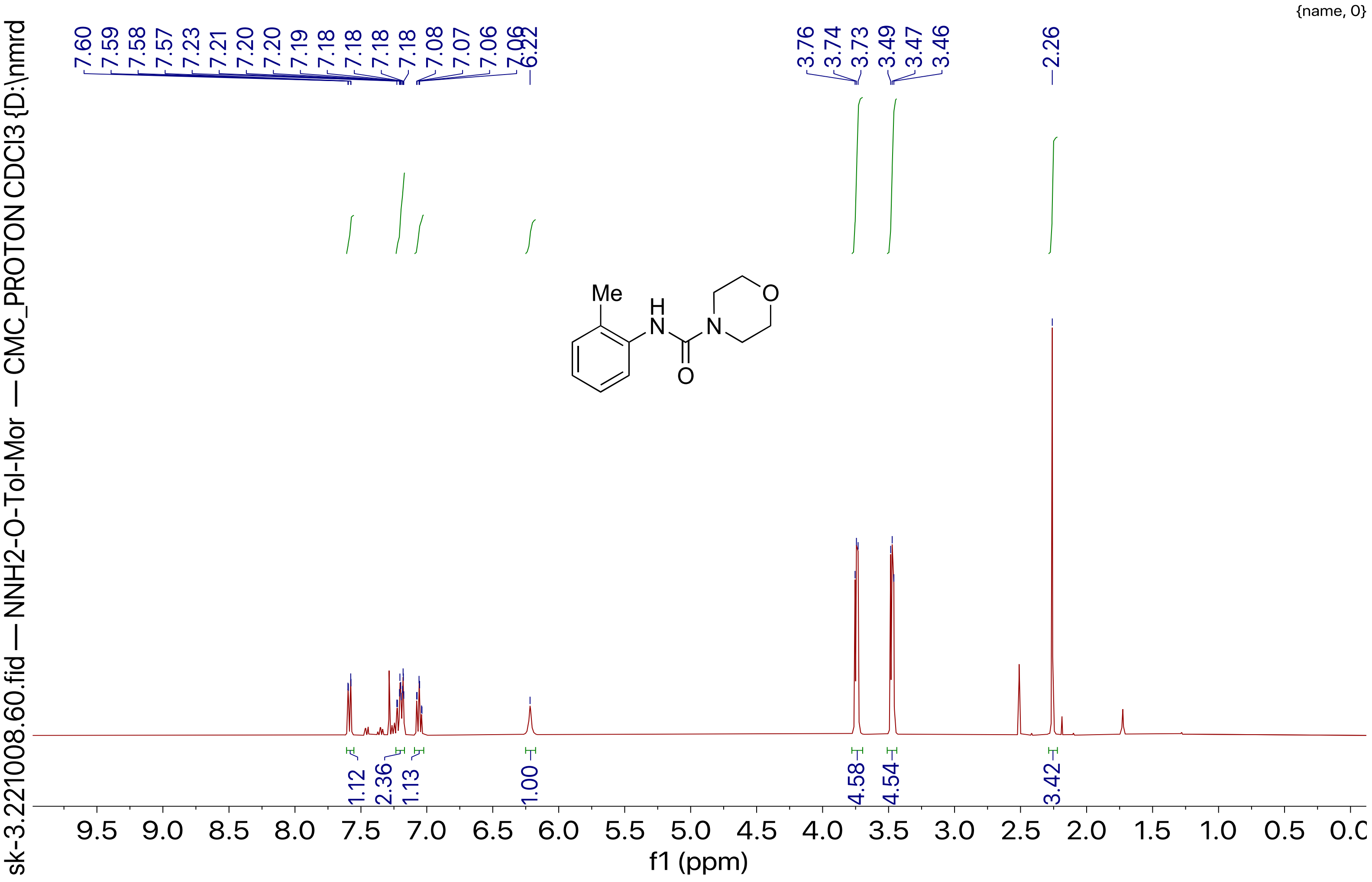

^{1}H NMR spectra of **7b'** (400 MHz, RT, $CDCl_3$)

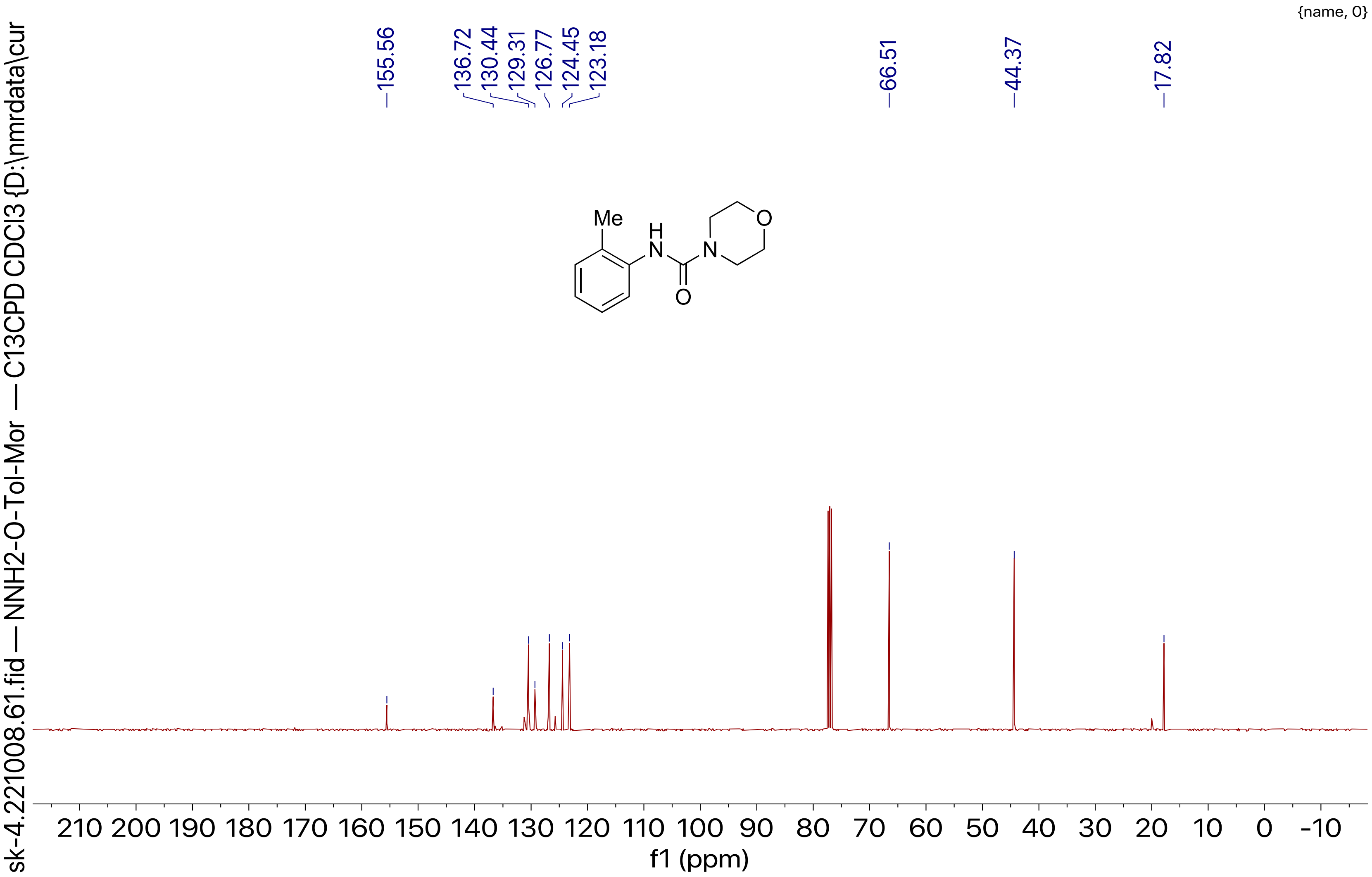

^{13}C NMR spectra of **7b’** (101 MHz, RT, $CDCl_3$)

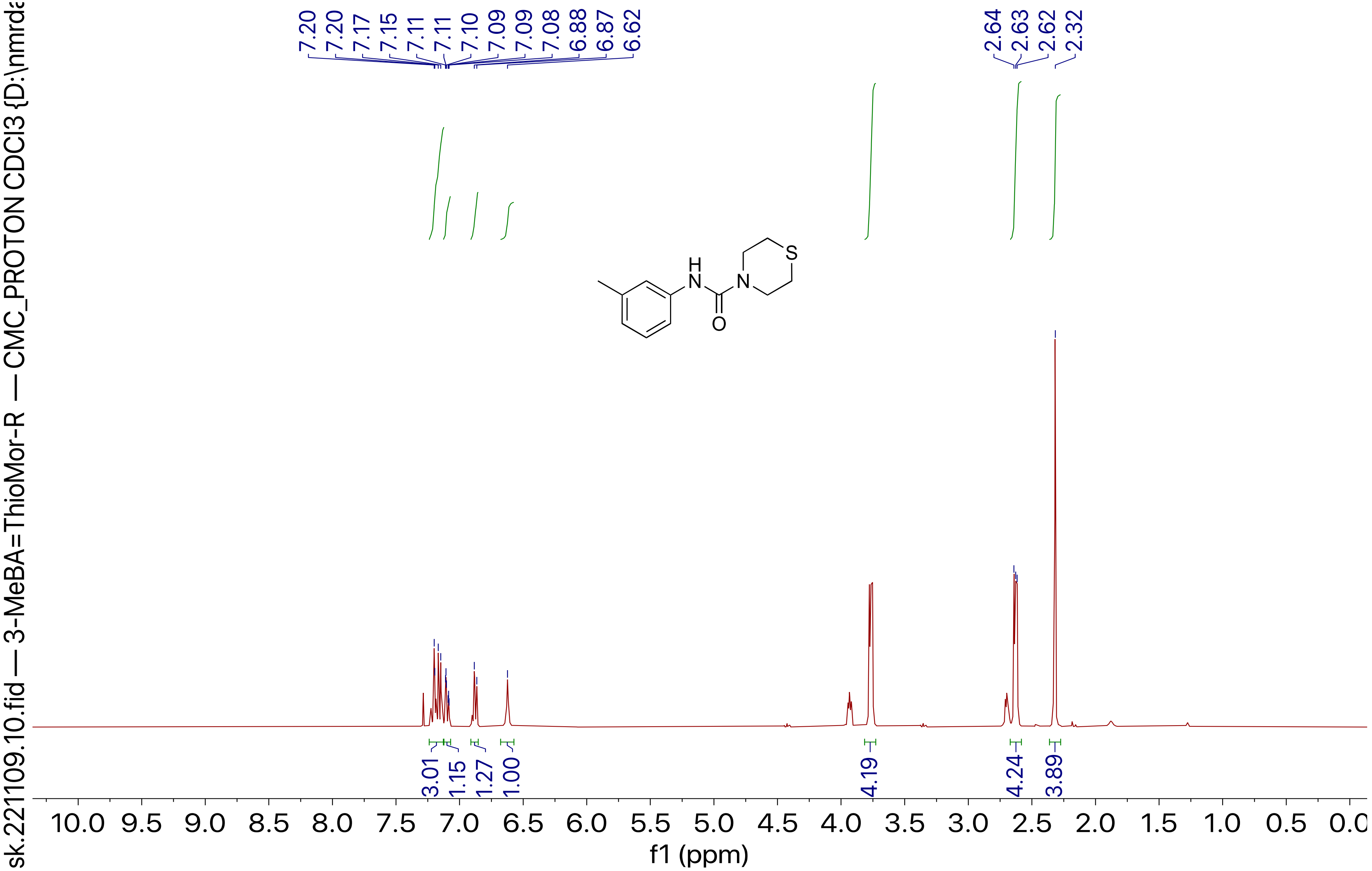

^{1}H NMR spectra of **7c** (400 MHz, RT, $CDCl_3$)

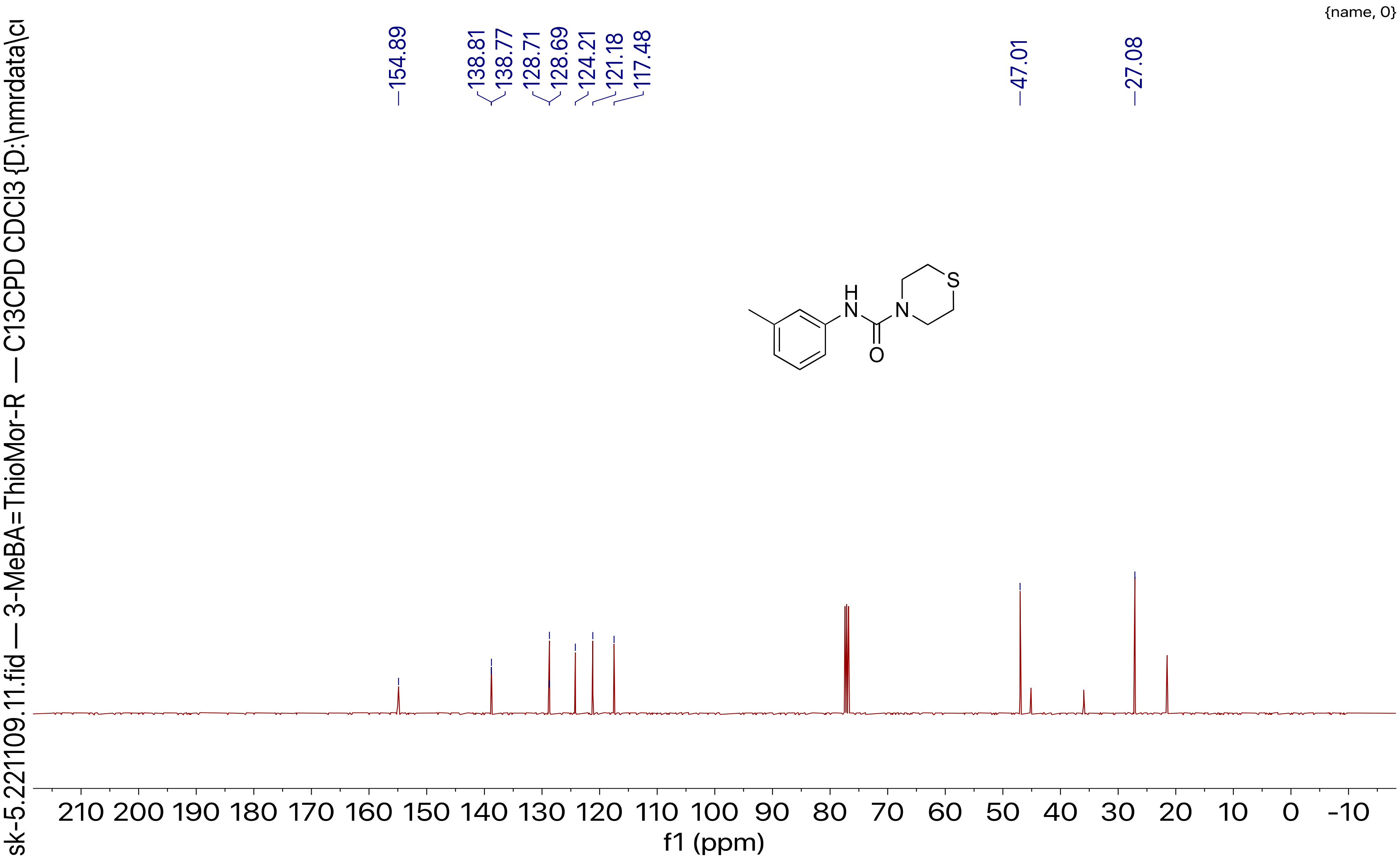

^{13}C NMR spectra of **7c** (100 MHz, RT, $CDCl_3$)

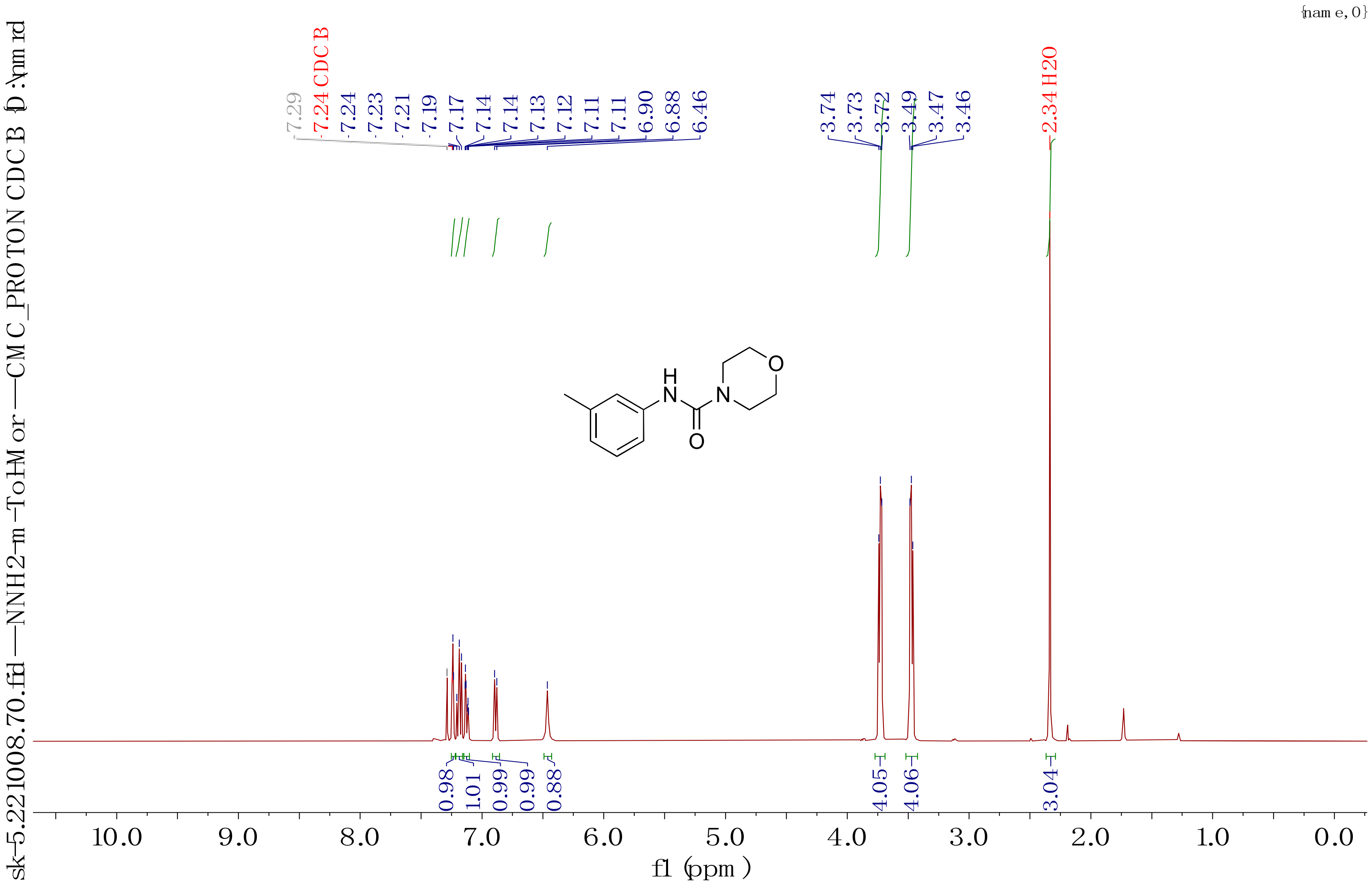

^{1}H NMR spectra of **7c'** (400 MHz, RT, $CDCl_3$)

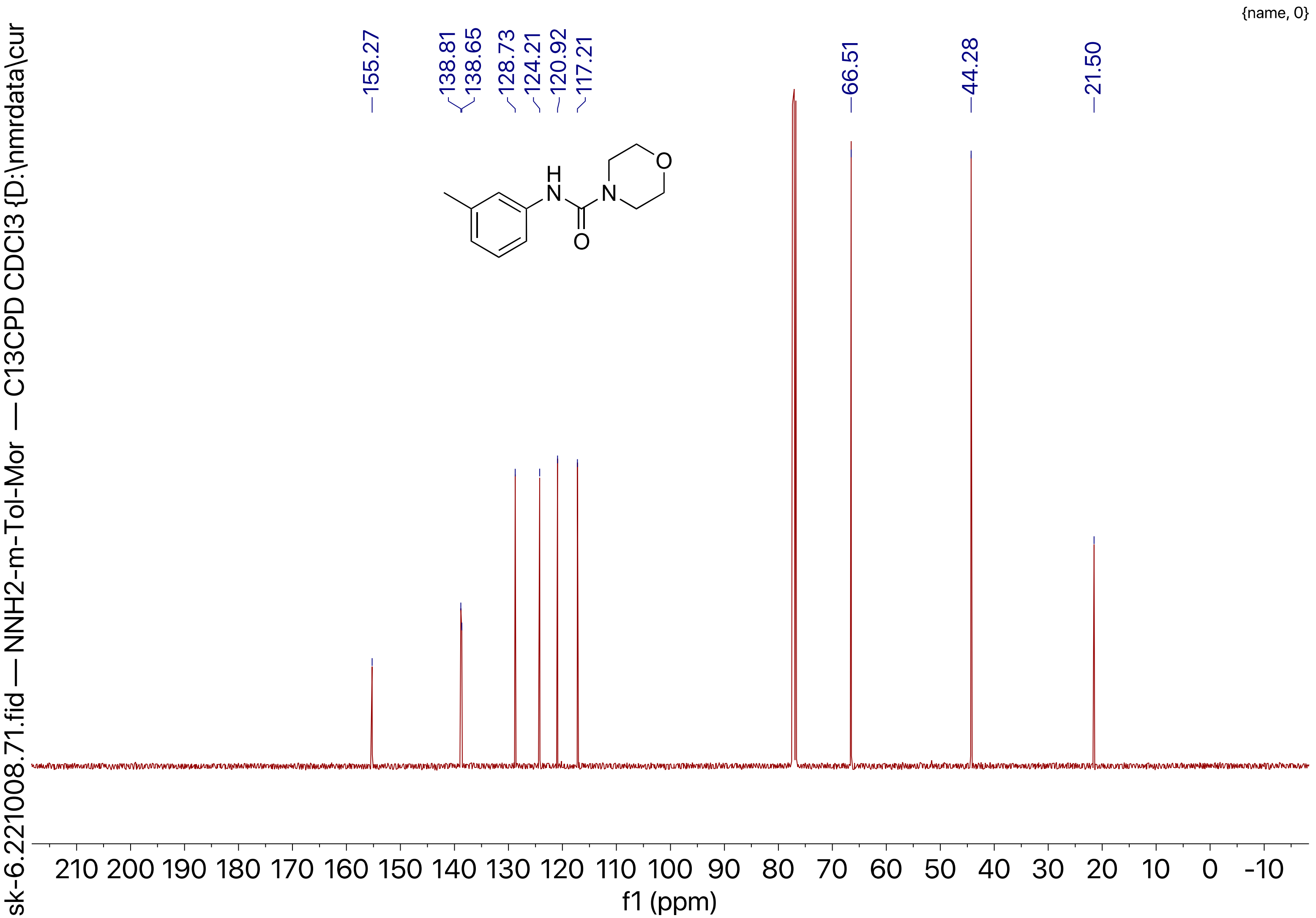

^{13}C NMR spectra of **7c’** (101 MHz, RT, $CDCl_3$)

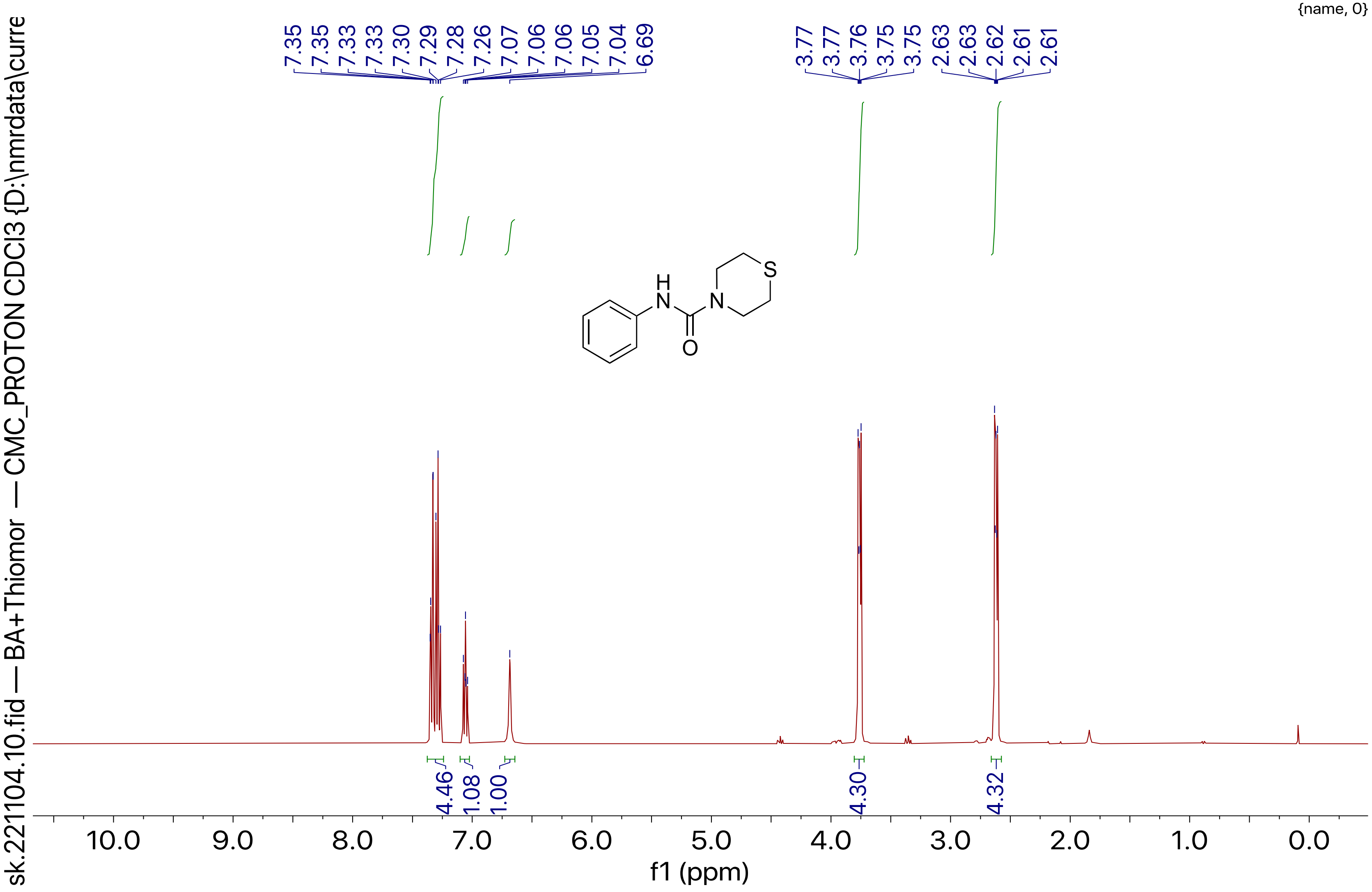

^{1}H NMR spectra of **7d** (400 MHz, RT, $CDCl_3$)

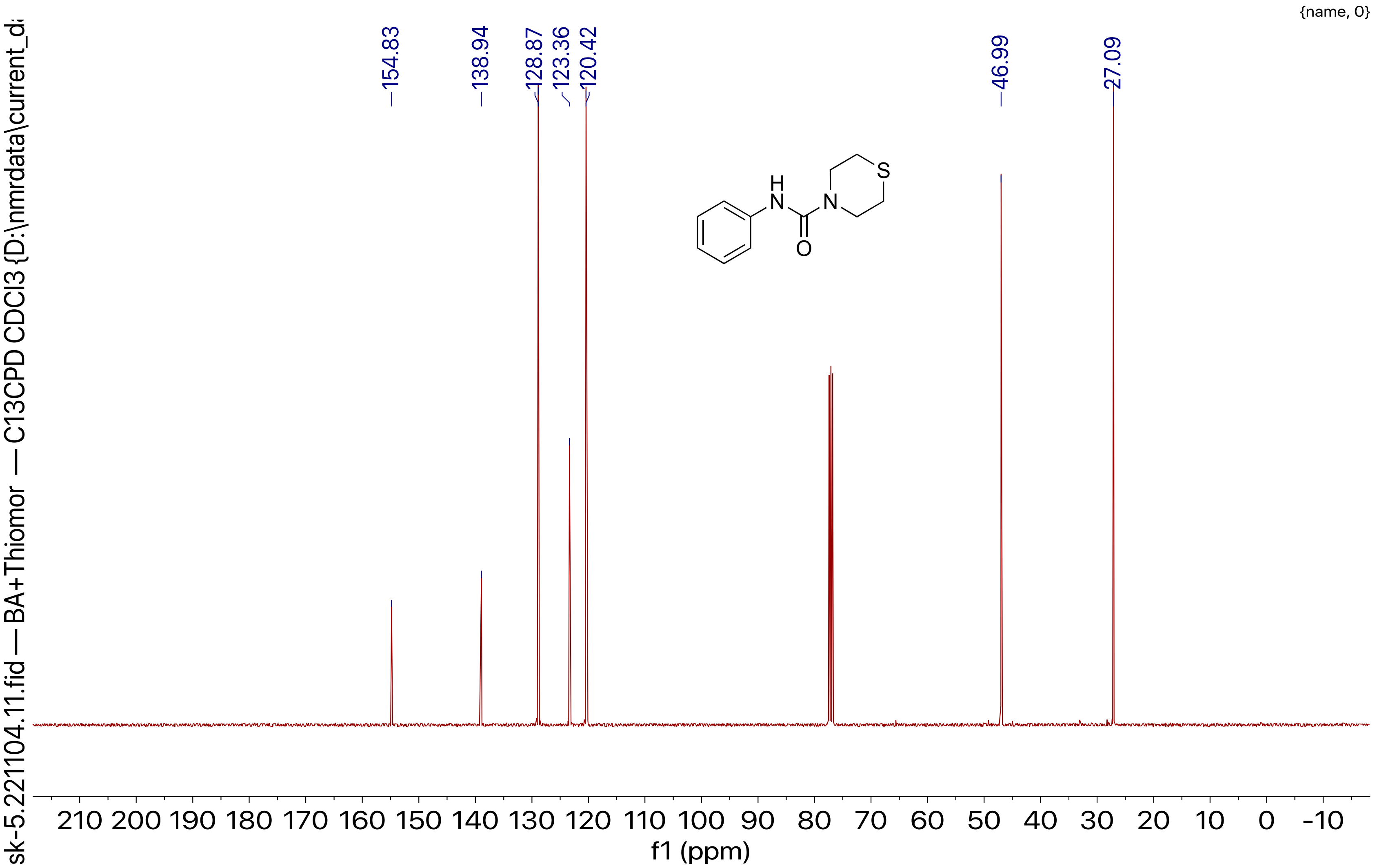

^{13}C NMR spectra of **7d** (100 MHz, RT, $CDCl_3$)

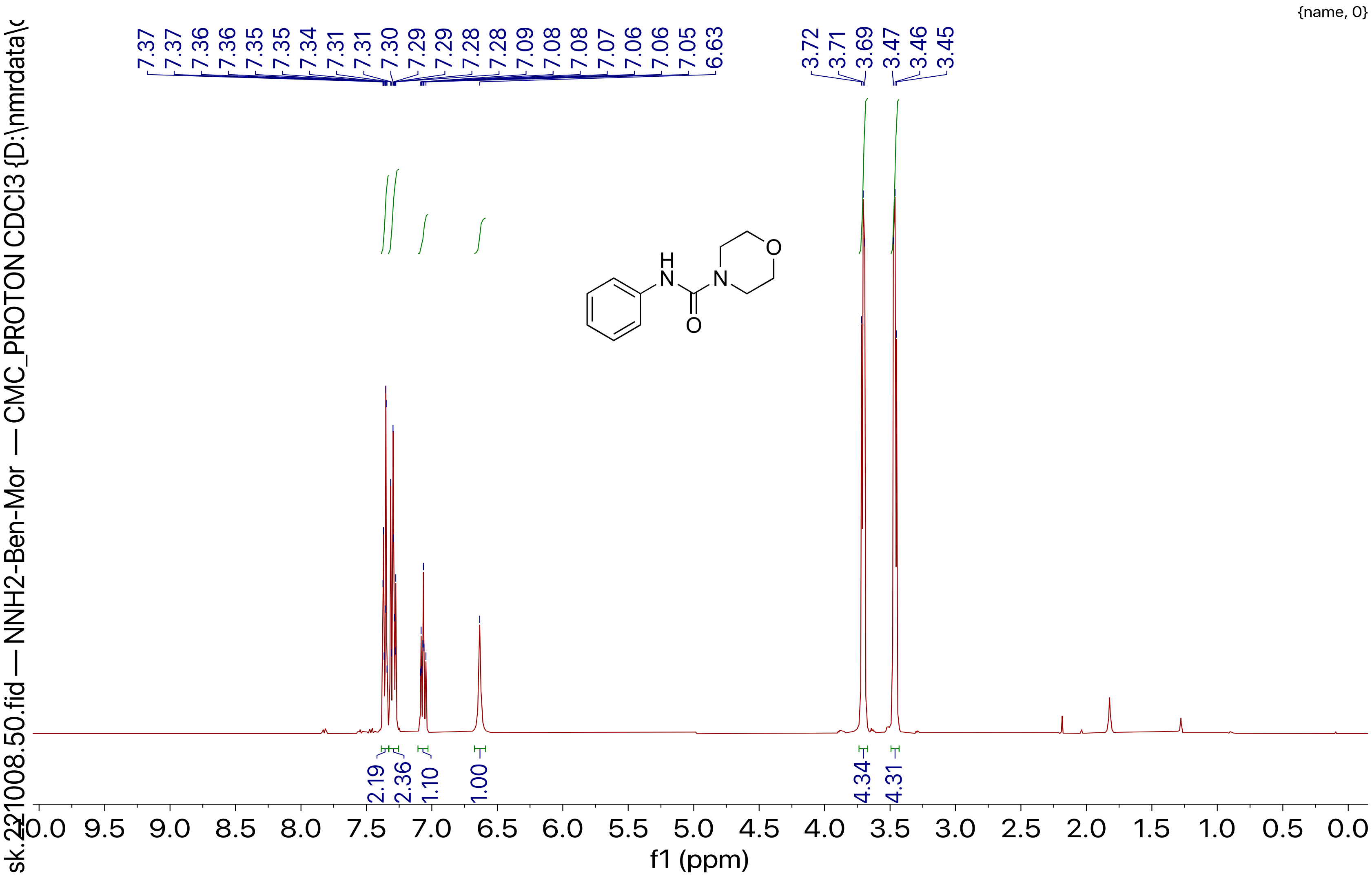

^{1}H NMR spectra of **7d'** (400 MHz, RT, $CDCl_3$)

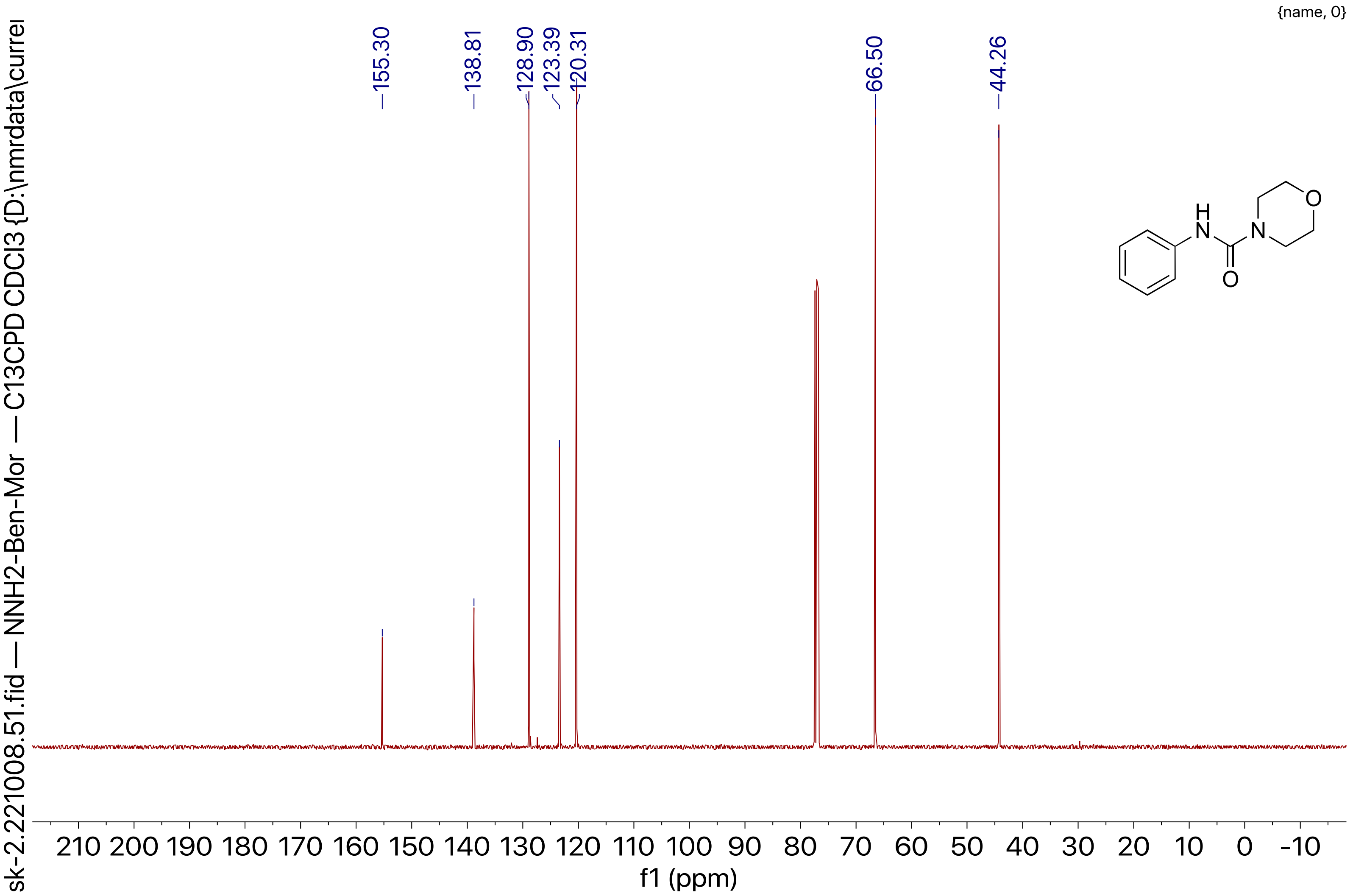

^{13}C NMR spectra of **7d’** (101 MHz, RT, $CDCl_3$)

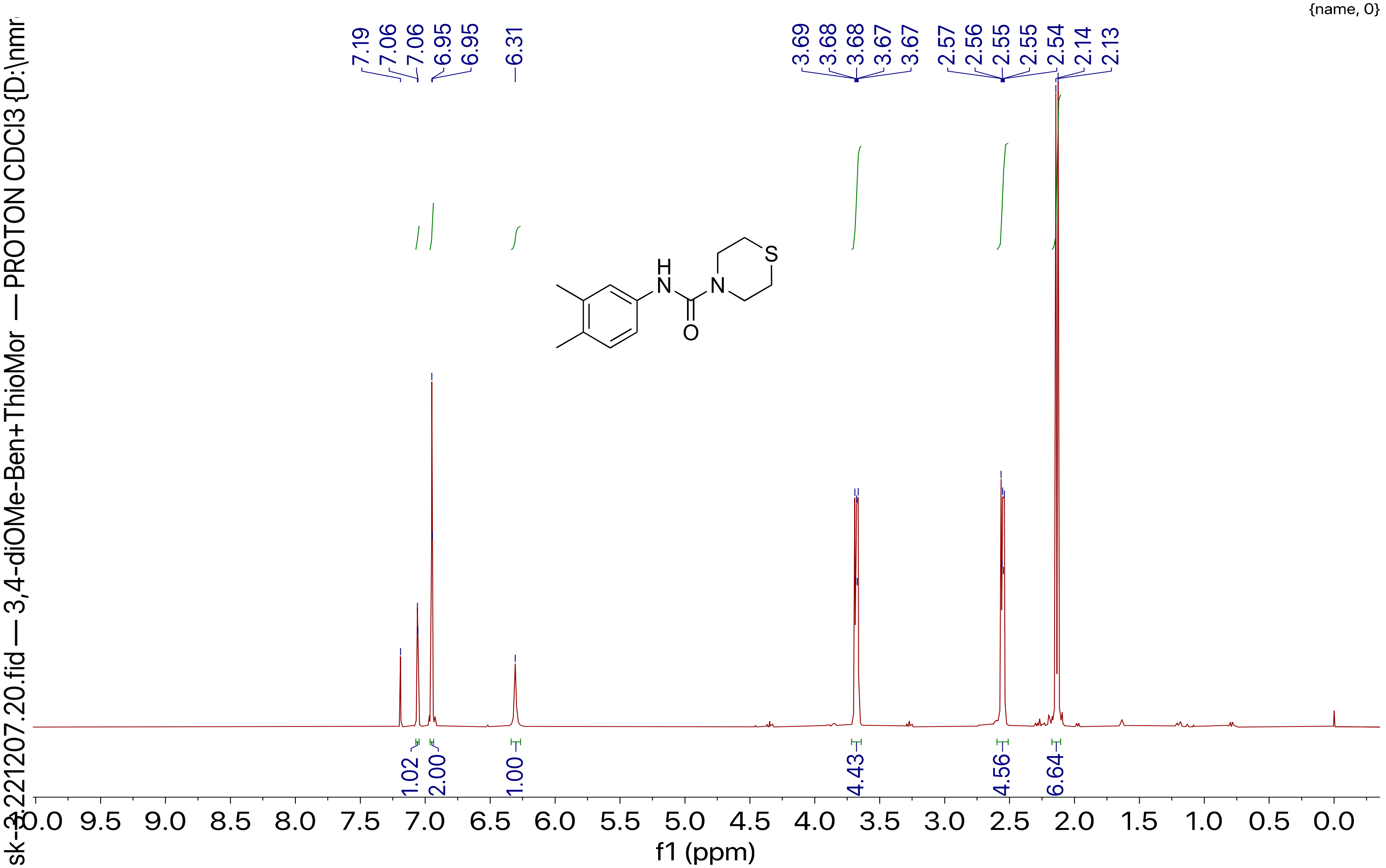

^{1}H NMR spectra of **7e** (400 MHz, RT, $CDCl_3$)

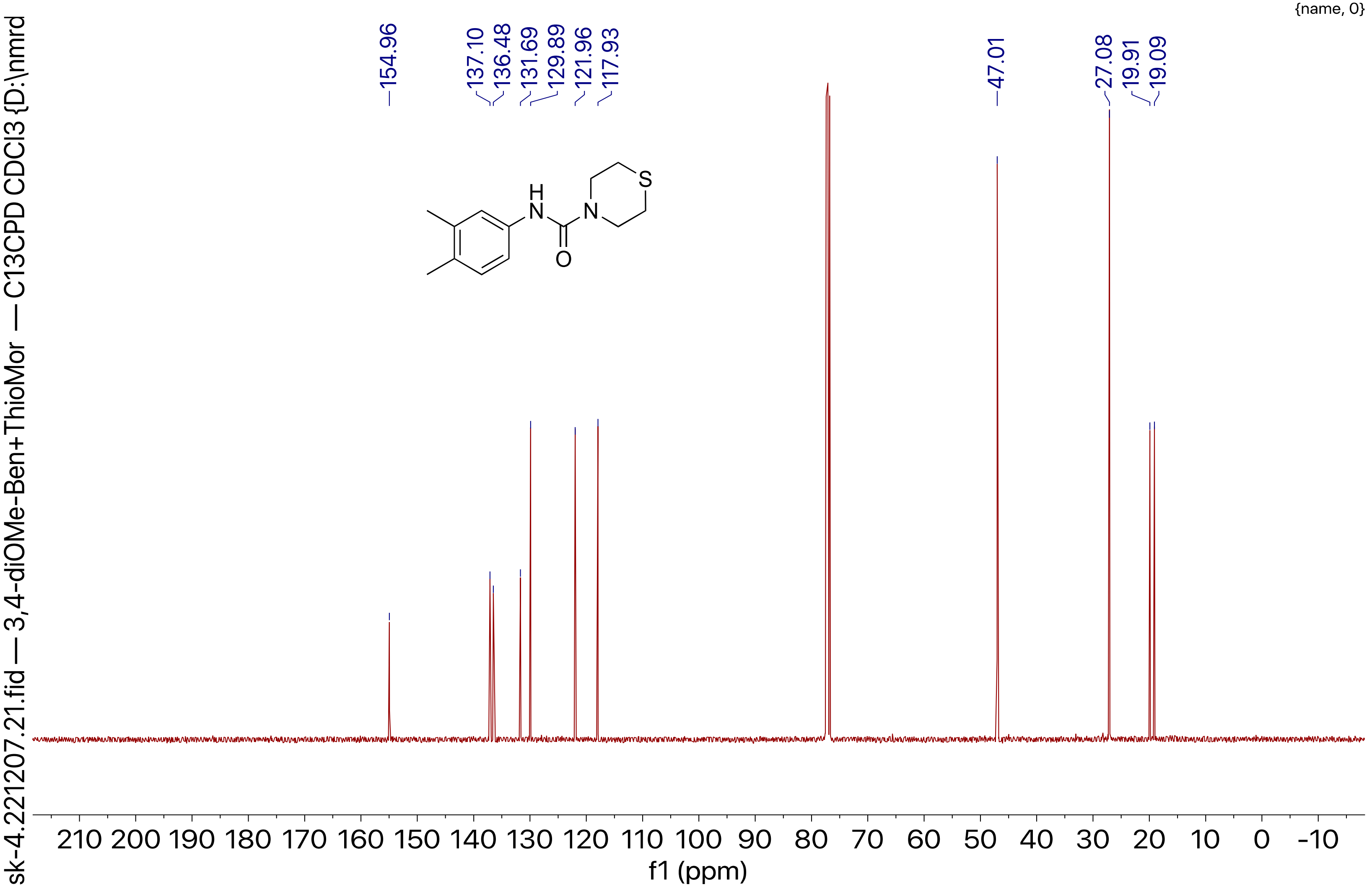

^{13}C NMR spectra of **7e** (101 MHz, RT, $CDCl_3$)

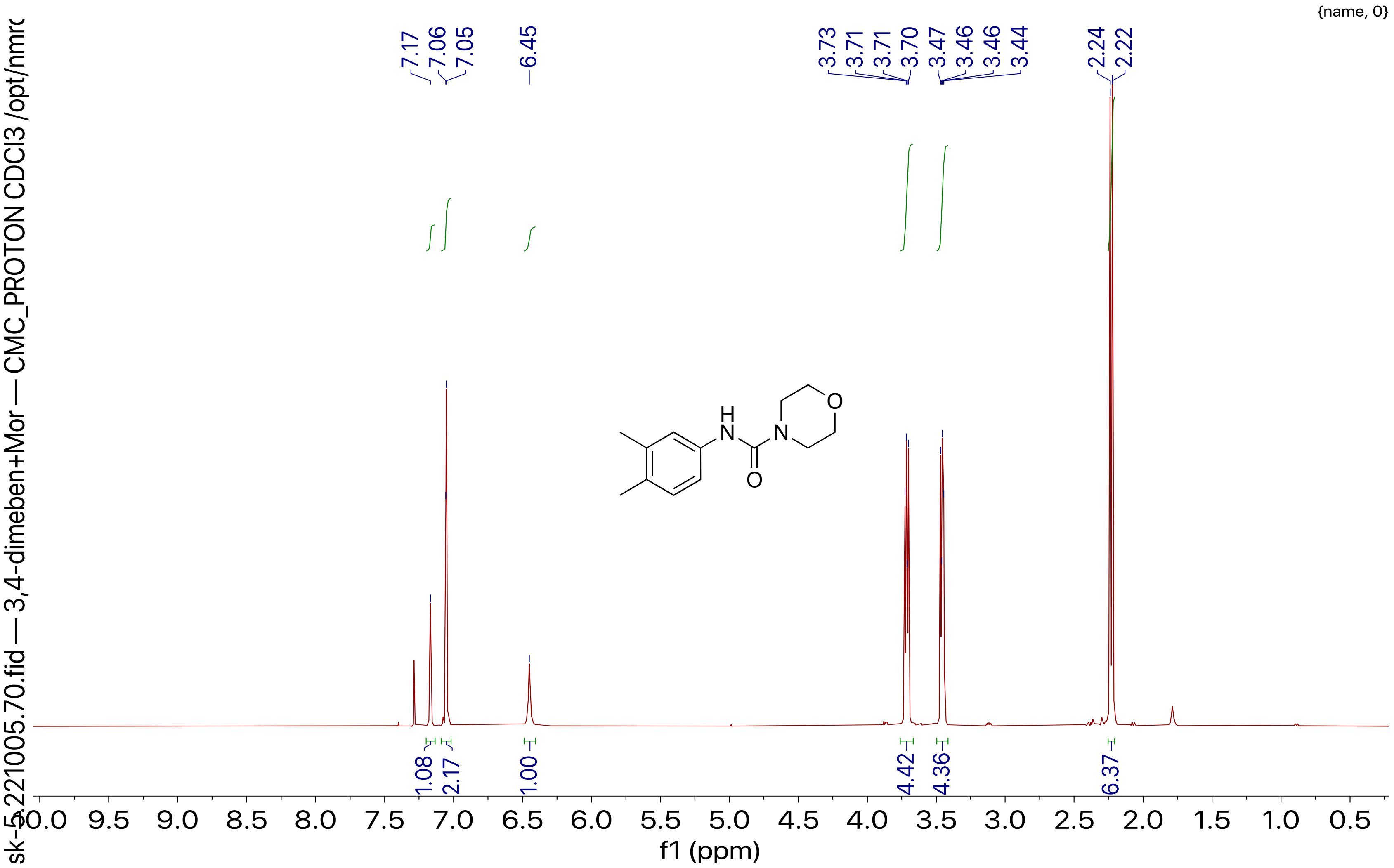

^{1}H NMR spectra of **7e’** (400 MHz, RT, $CDCl_3$)

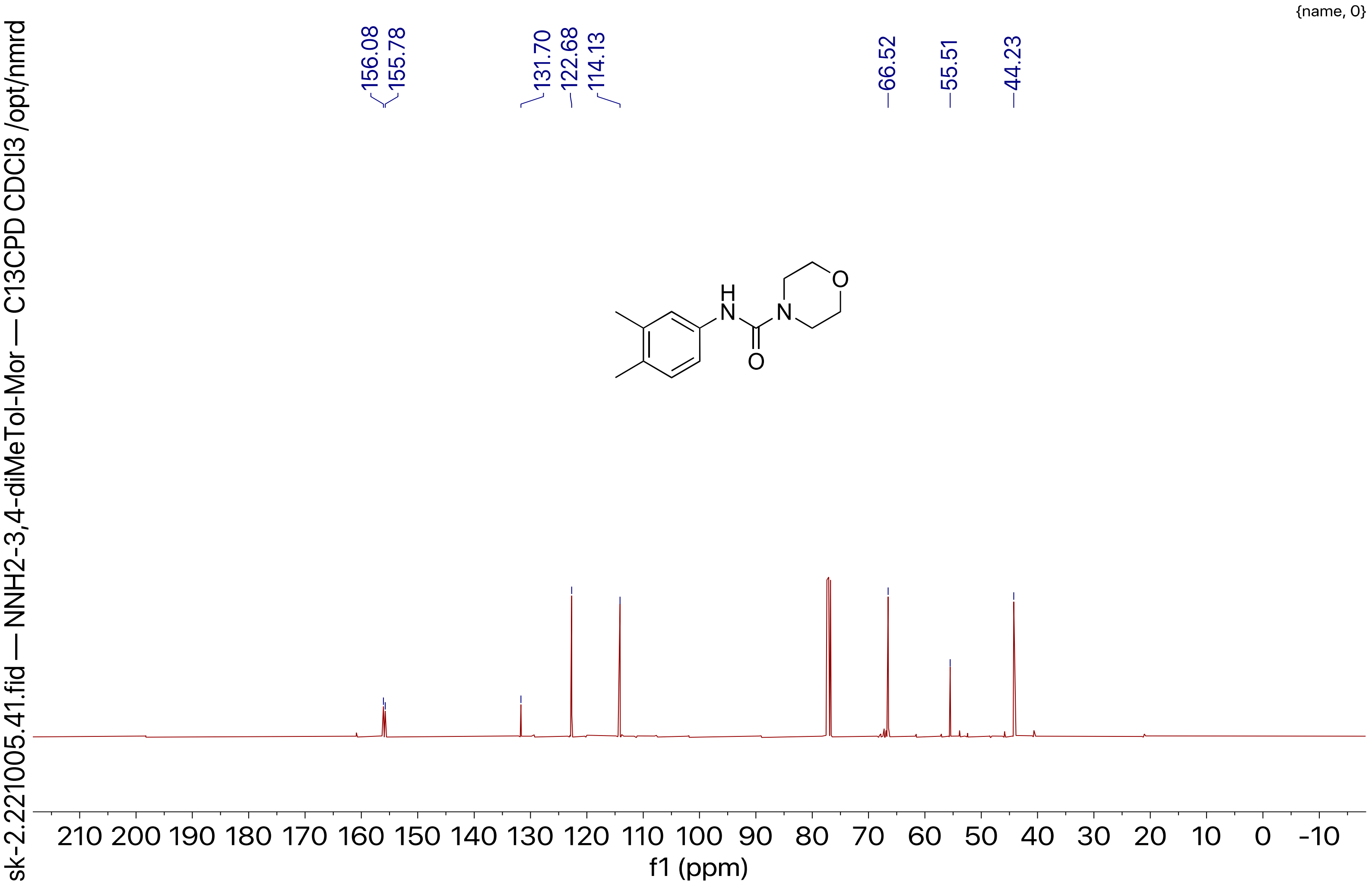

^{13}C NMR spectra of **7e’** (100 MHz, RT, $CDCl_3$)

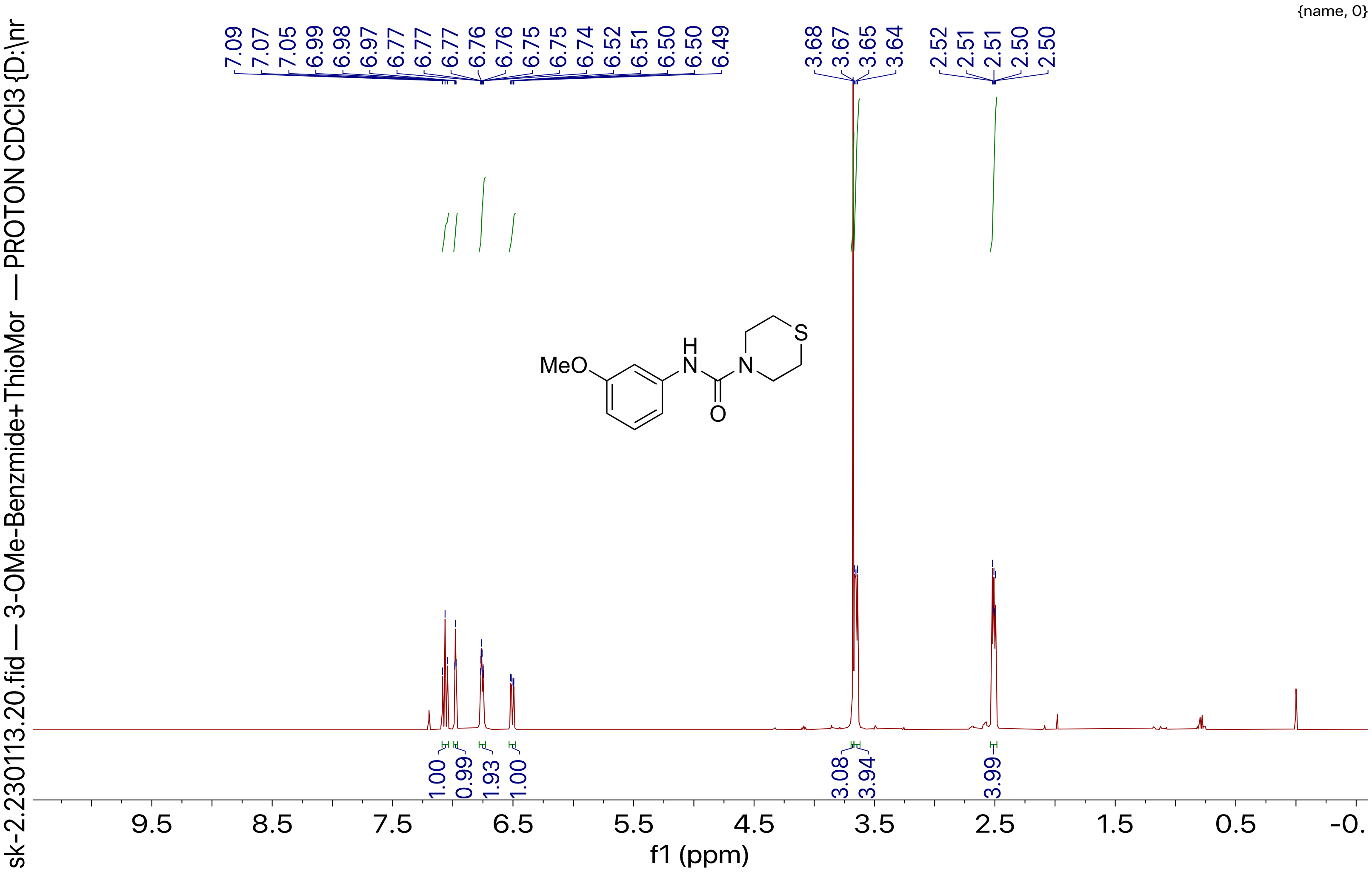

^{1}H NMR spectra of **7f** (400 MHz, RT, $CDCl_3$)

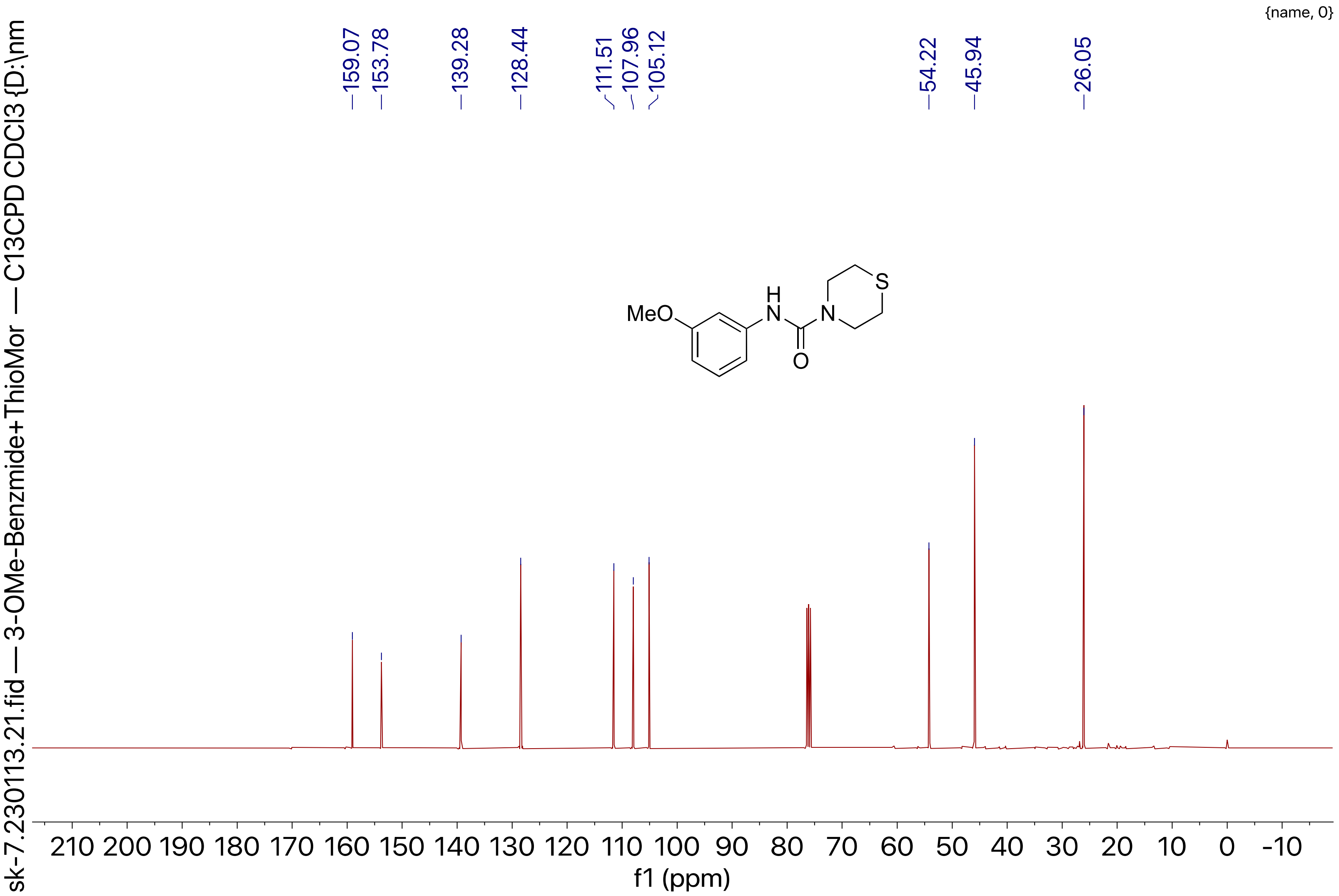

^{13}C NMR spectra of **7f** (101 MHz, RT, $CDCl_3$)

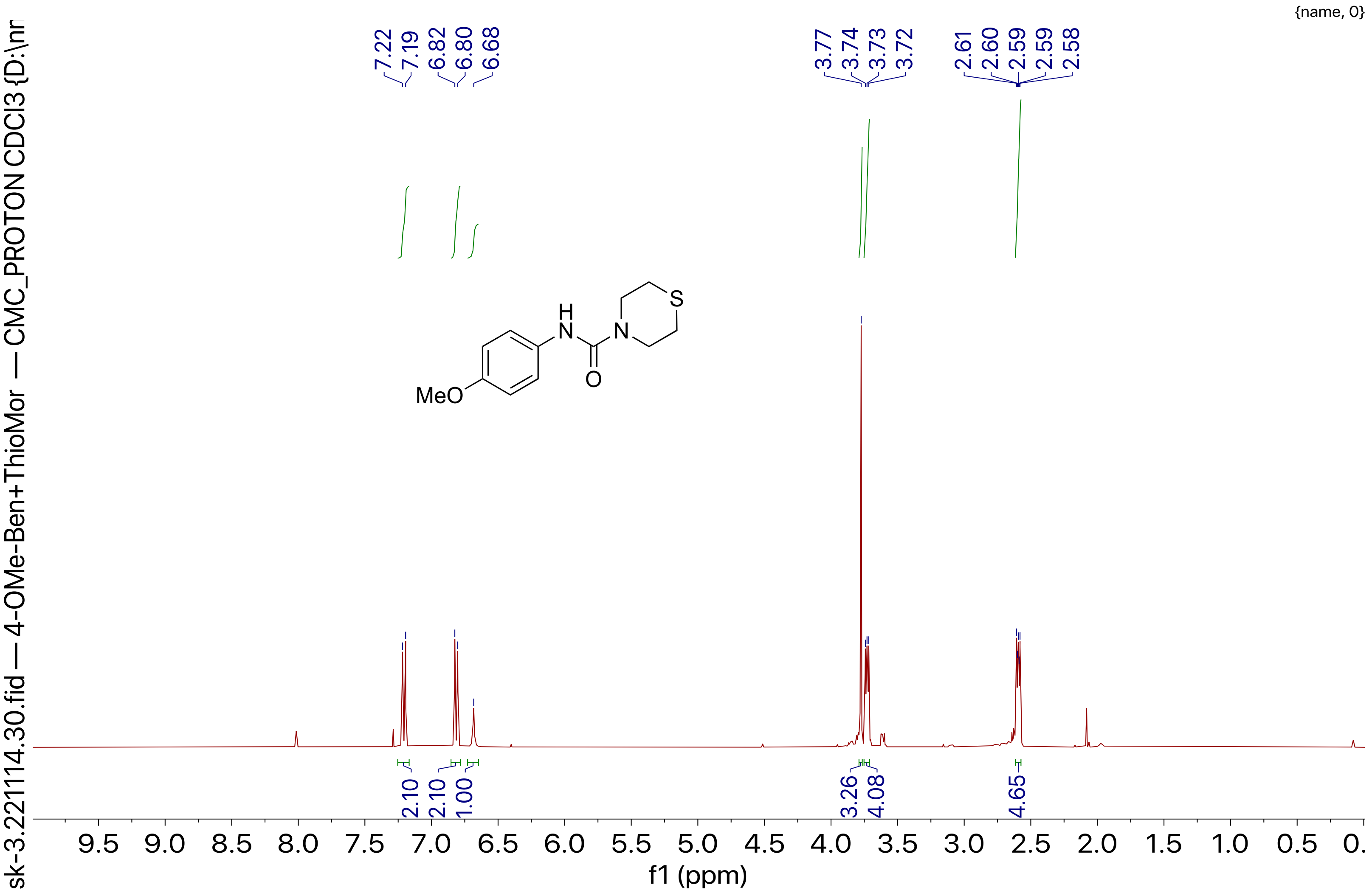

^{1}H NMR spectra of **7g** (400 MHz, RT, $CDCl_3$)

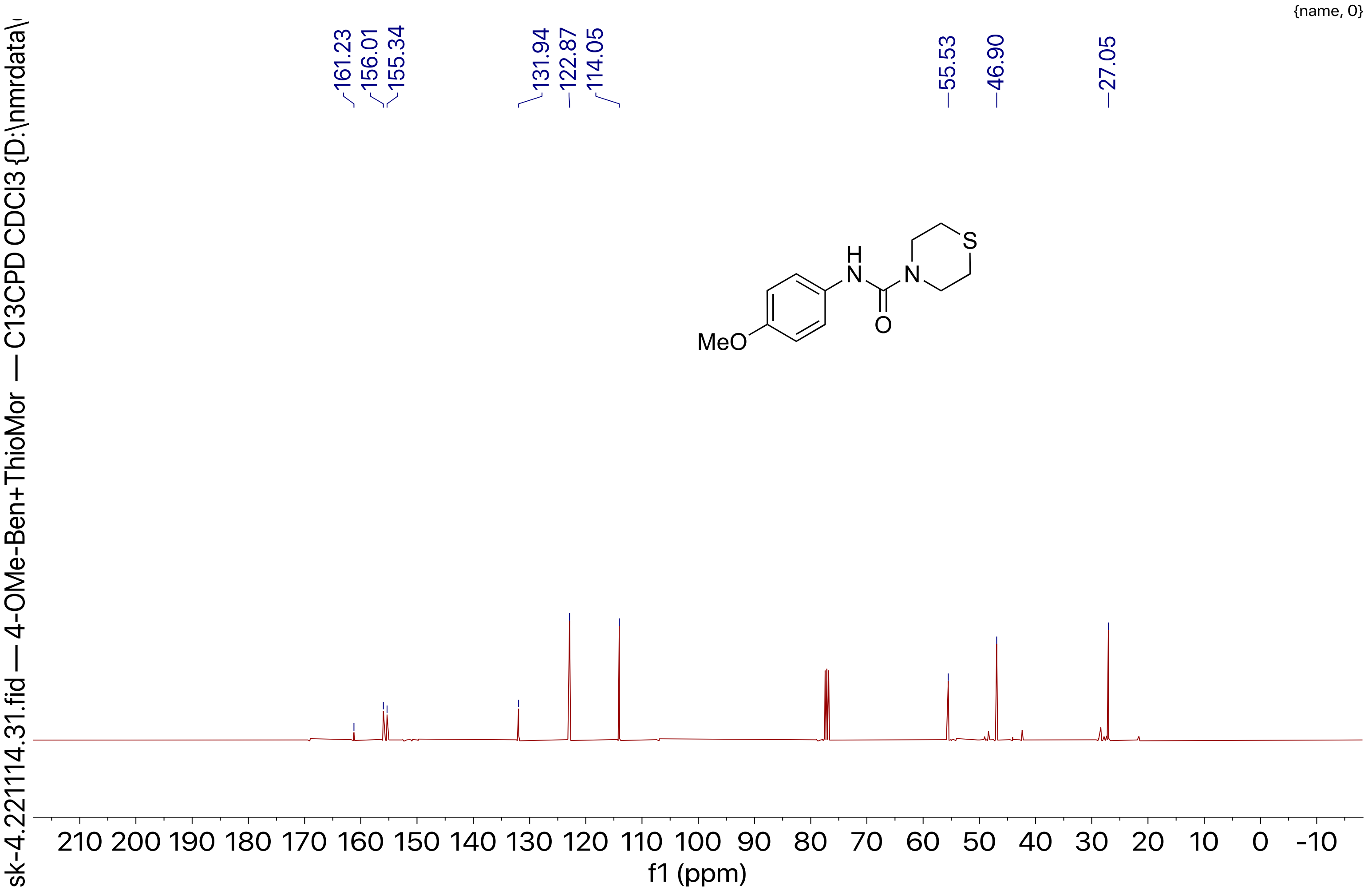

^{13}C NMR spectra of **7g** (101 MHz, RT, $CDCl_3$)

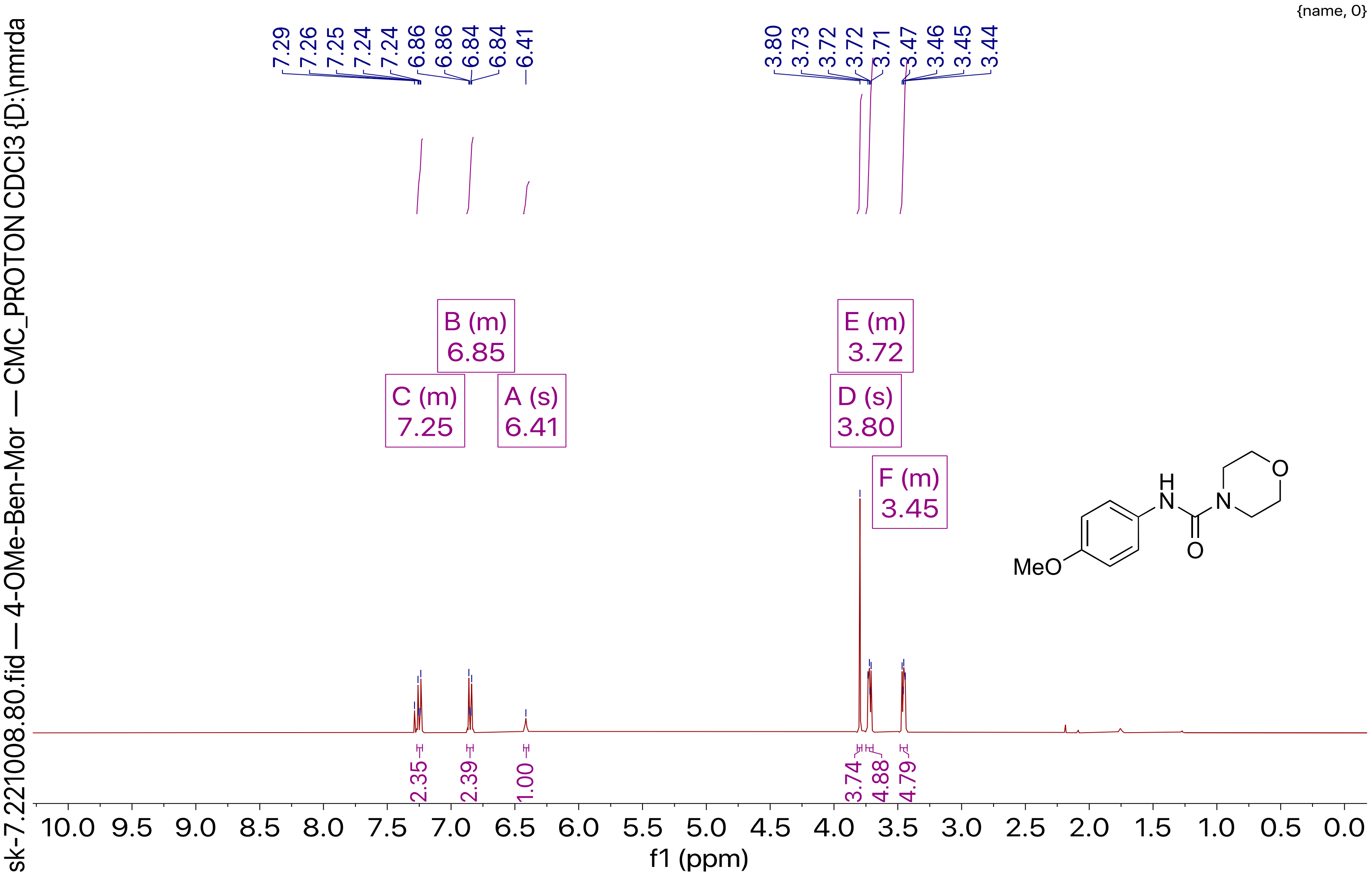

^{1}H NMR spectra of **7g’** (400 MHz, RT, $CDCl_3$)

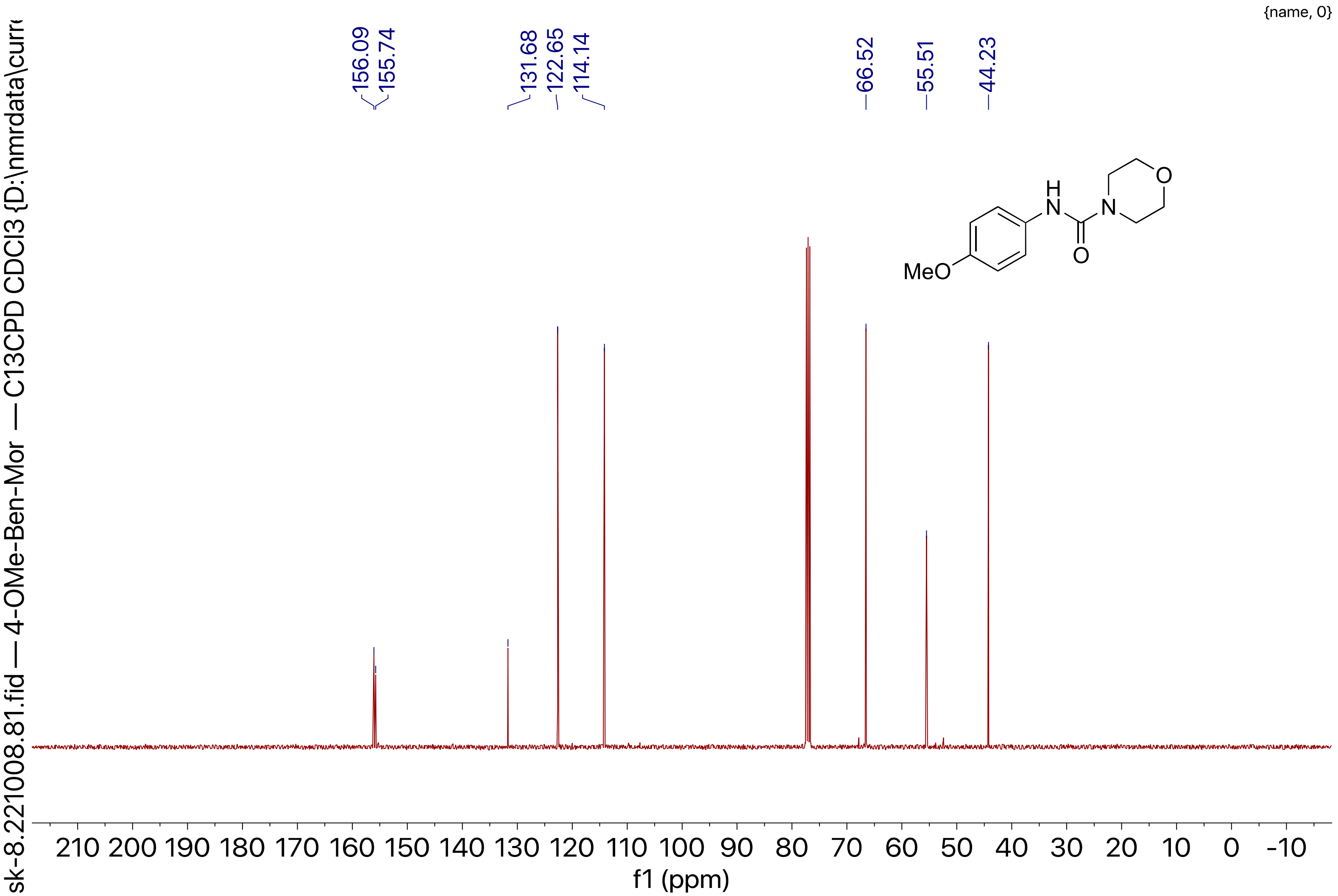

^{13}C NMR spectra of **7g’** (101 MHz, RT, $CDCl_3$)

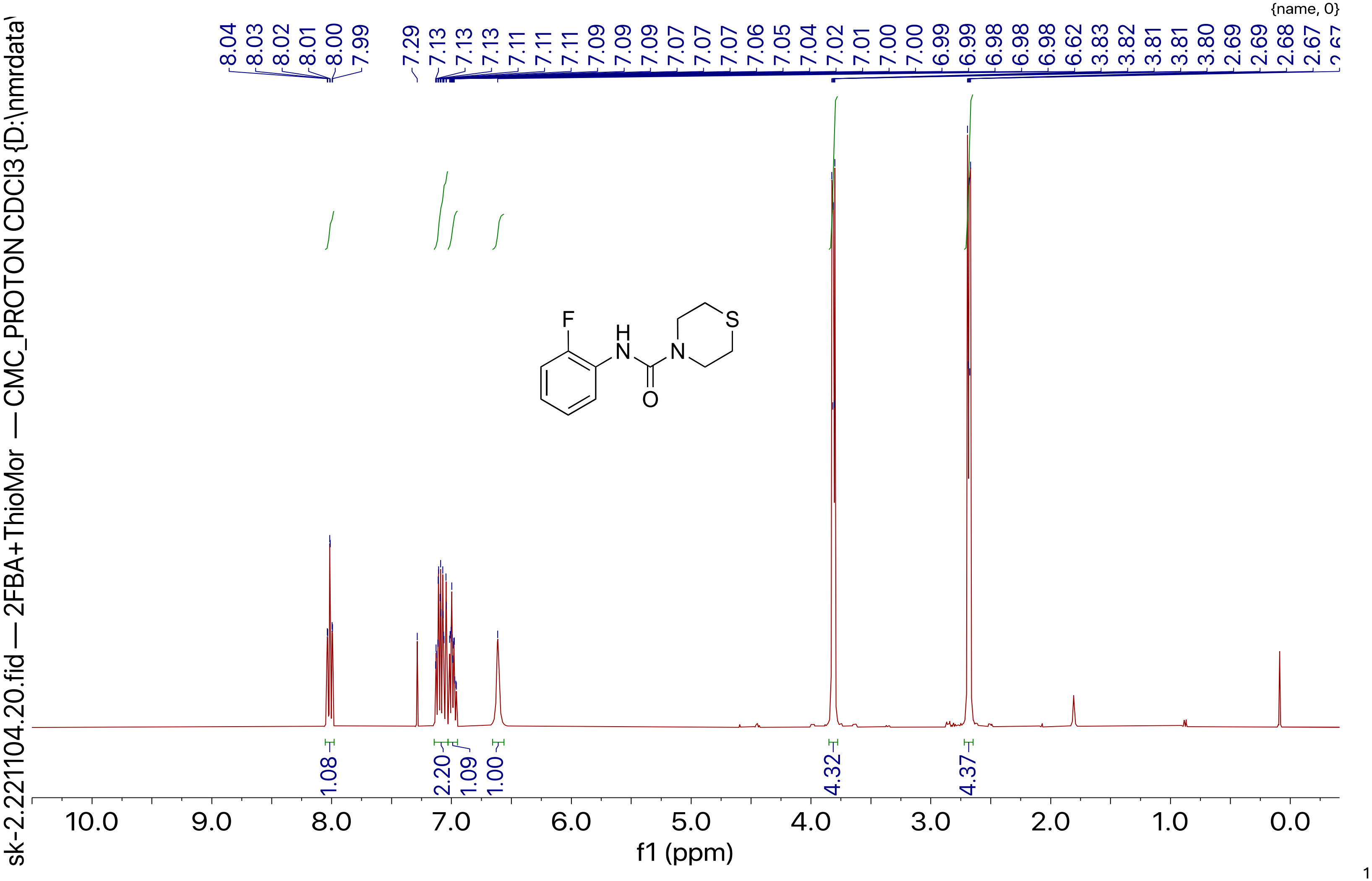

H NMR spectra of **7h** (400 MHz, RT, $CDCl_3$)

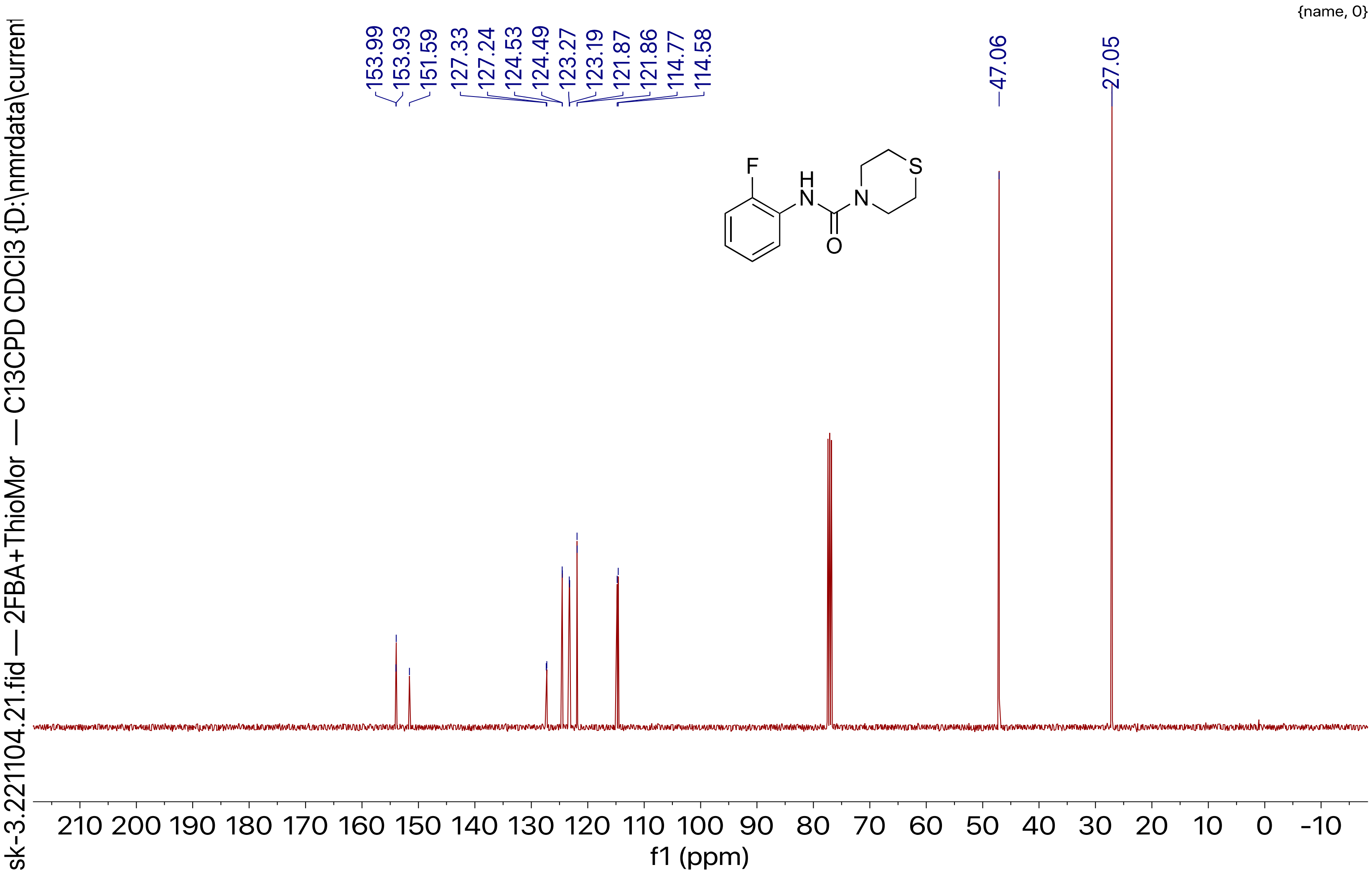

^{13}C NMR spectra of **7h** (100 MHz, RT, $CDCl_3$)

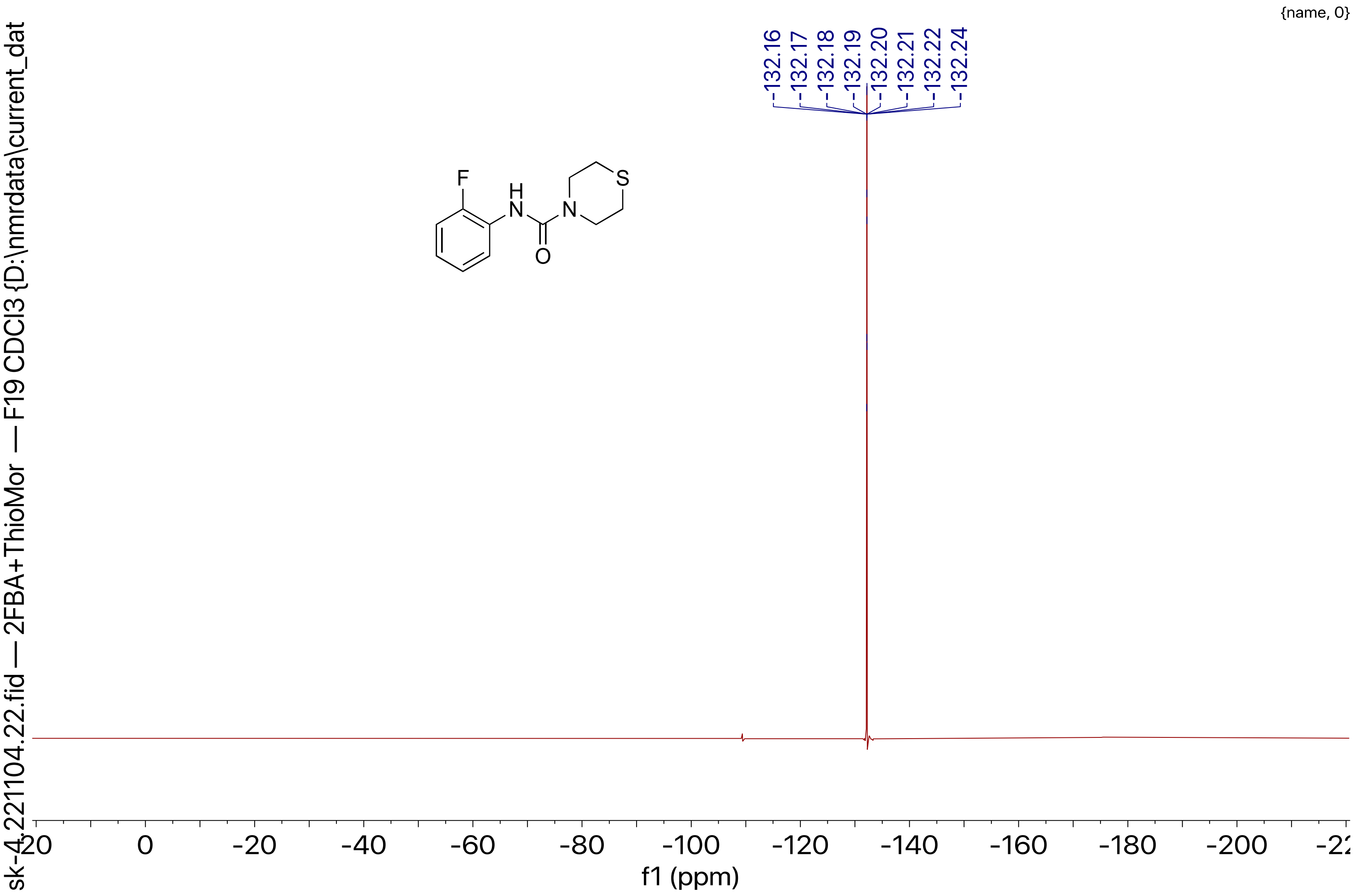

^{19}F NMR spectra of **7h** (376 MHz, RT, $CDCl_3$)

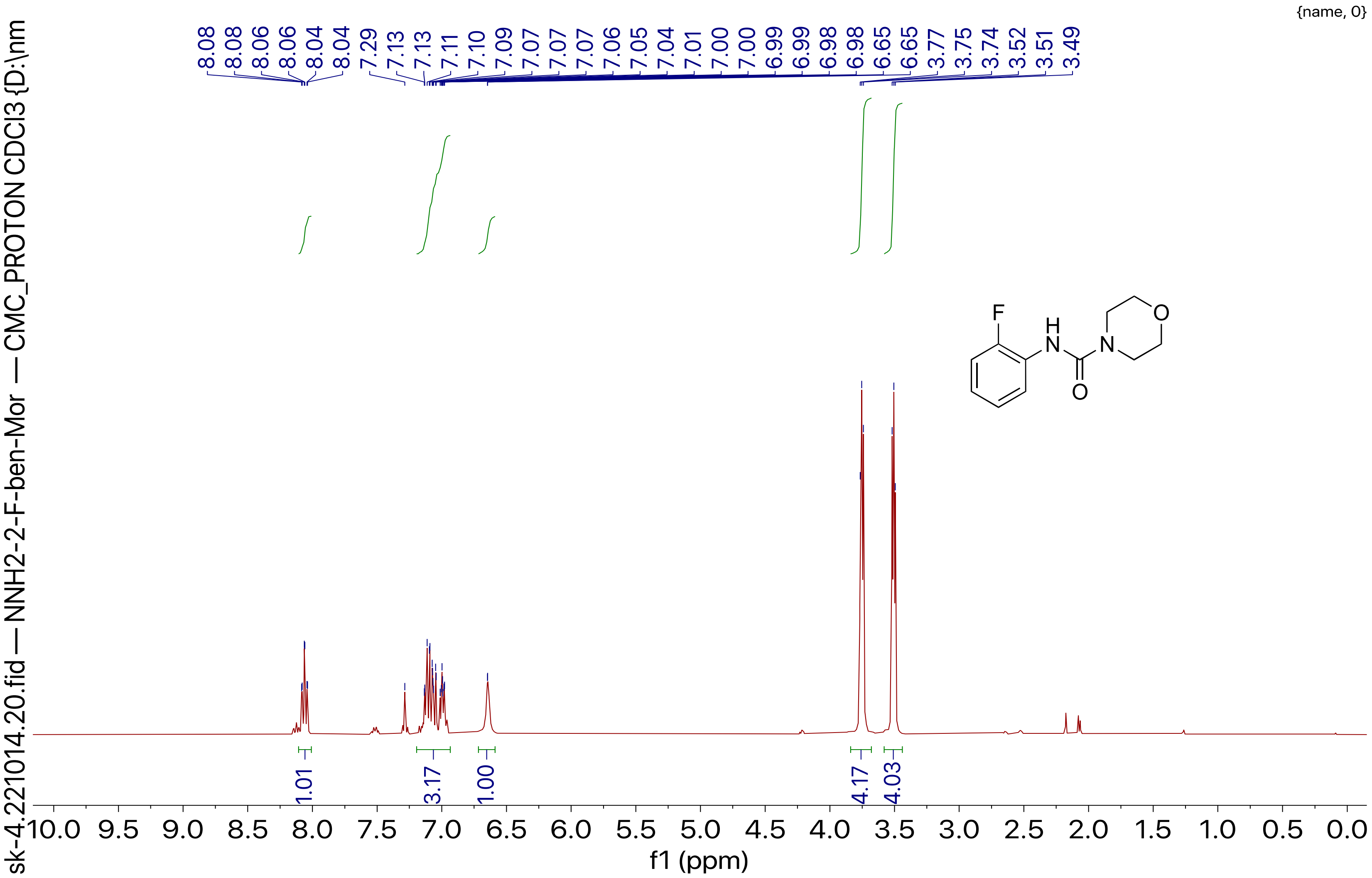

^{1}H NMR spectra of **7h’** (400 MHz, RT, $CDCl_3$)

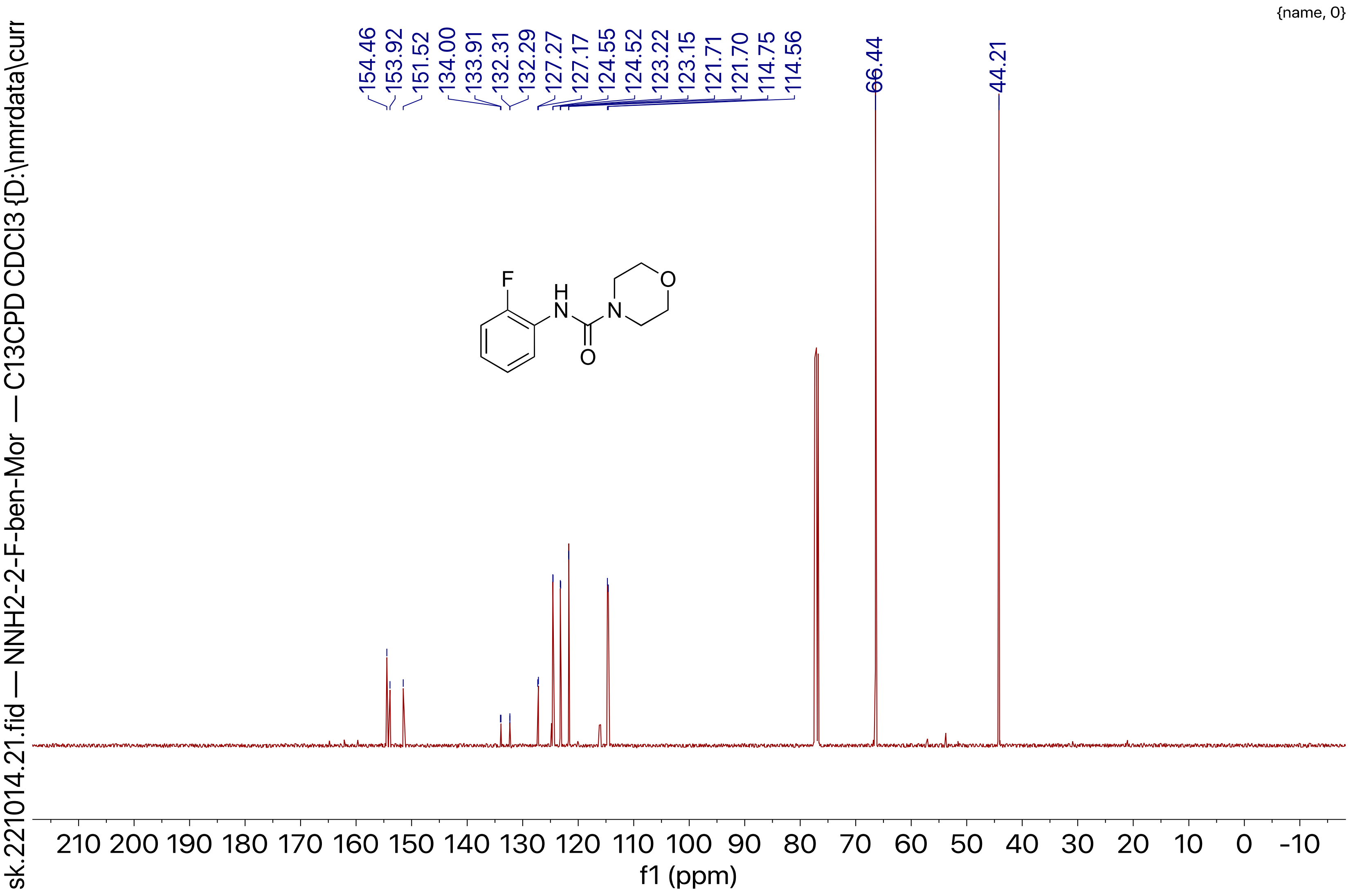

^{13}C NMR spectra of **7h'** (101 MHz, RT, $CDCl_3$)

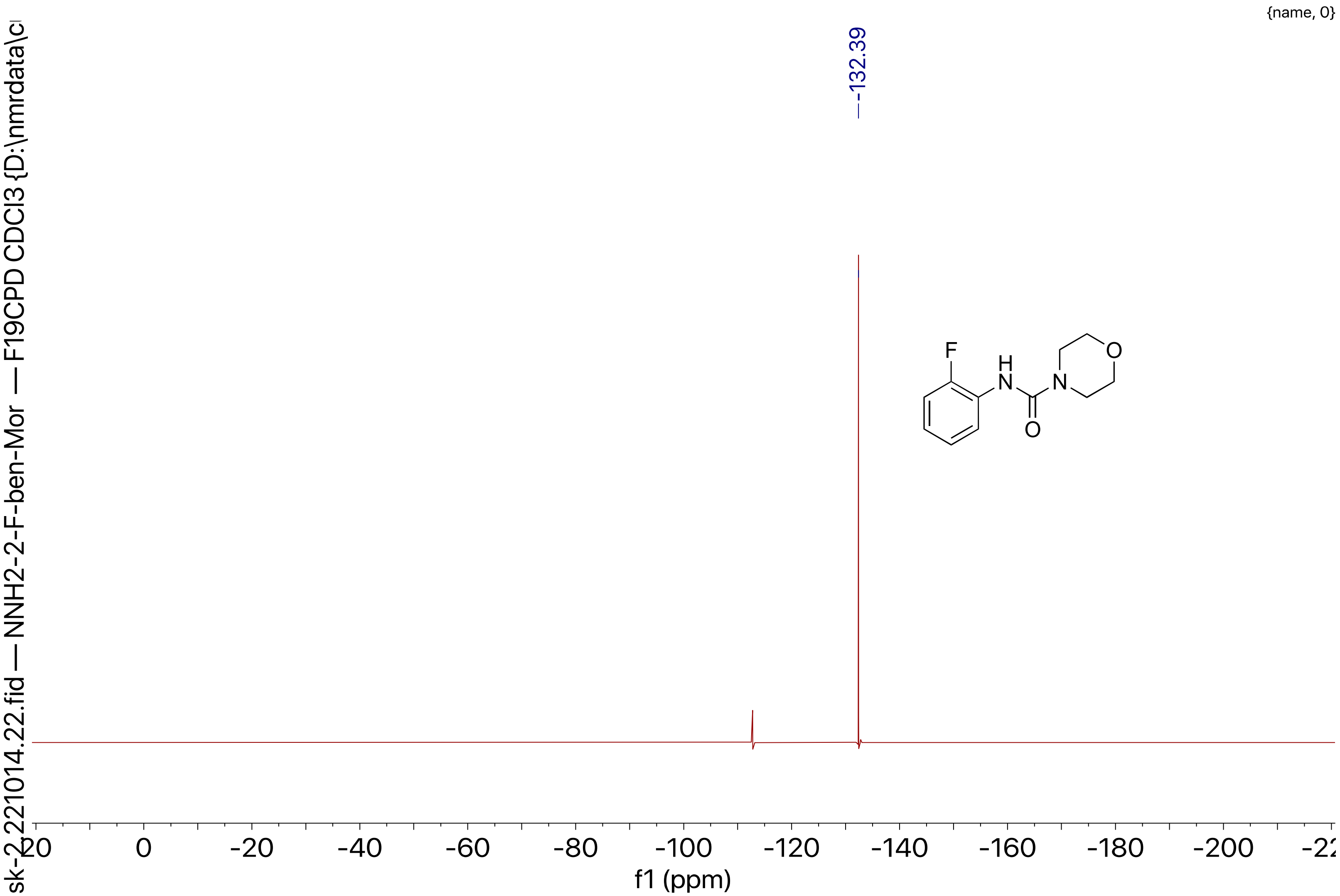

^{19}F NMR spectra of **7h’** (376 MHz, RT, CDCl$_3$)

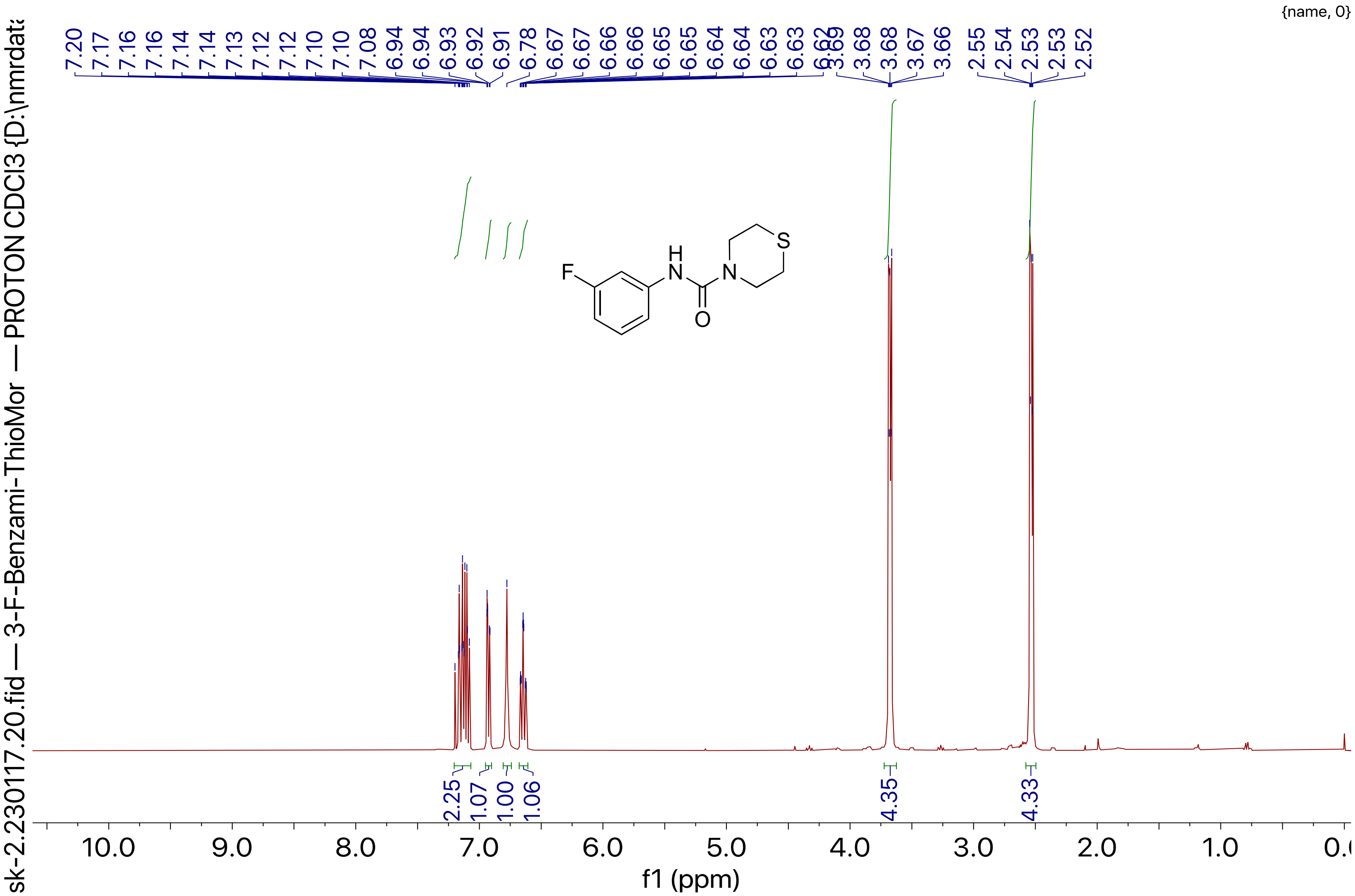

^{1}H NMR spectra of **7i** (400 MHz, RT, $CDCl_3$)

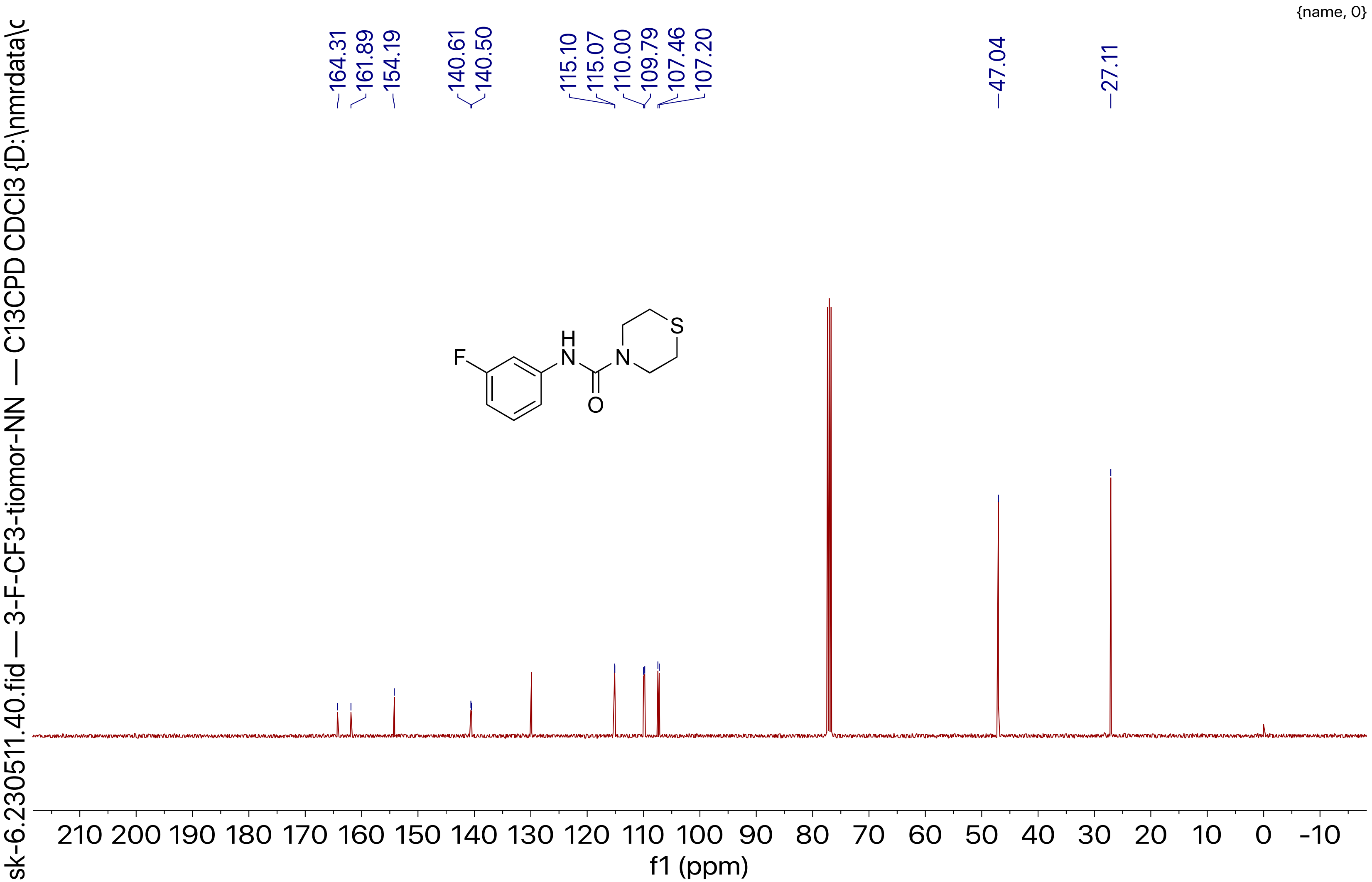

^{13}C NMR spectra of **7i** (400 MHz, RT, $CDCl_3$)

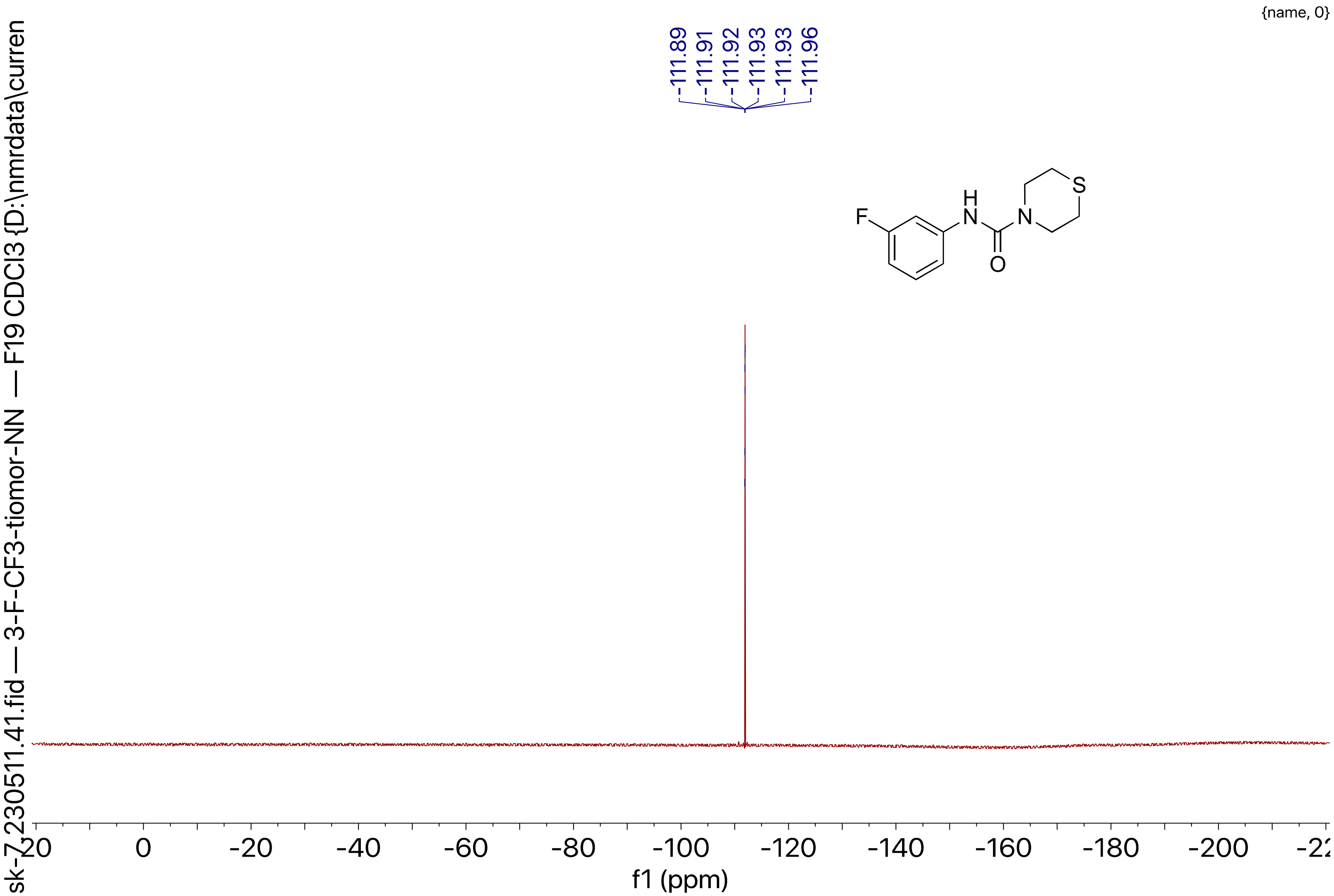

^{19}F NMR spectra of **7i** (376 MHz, RT, $CDCl_3$)

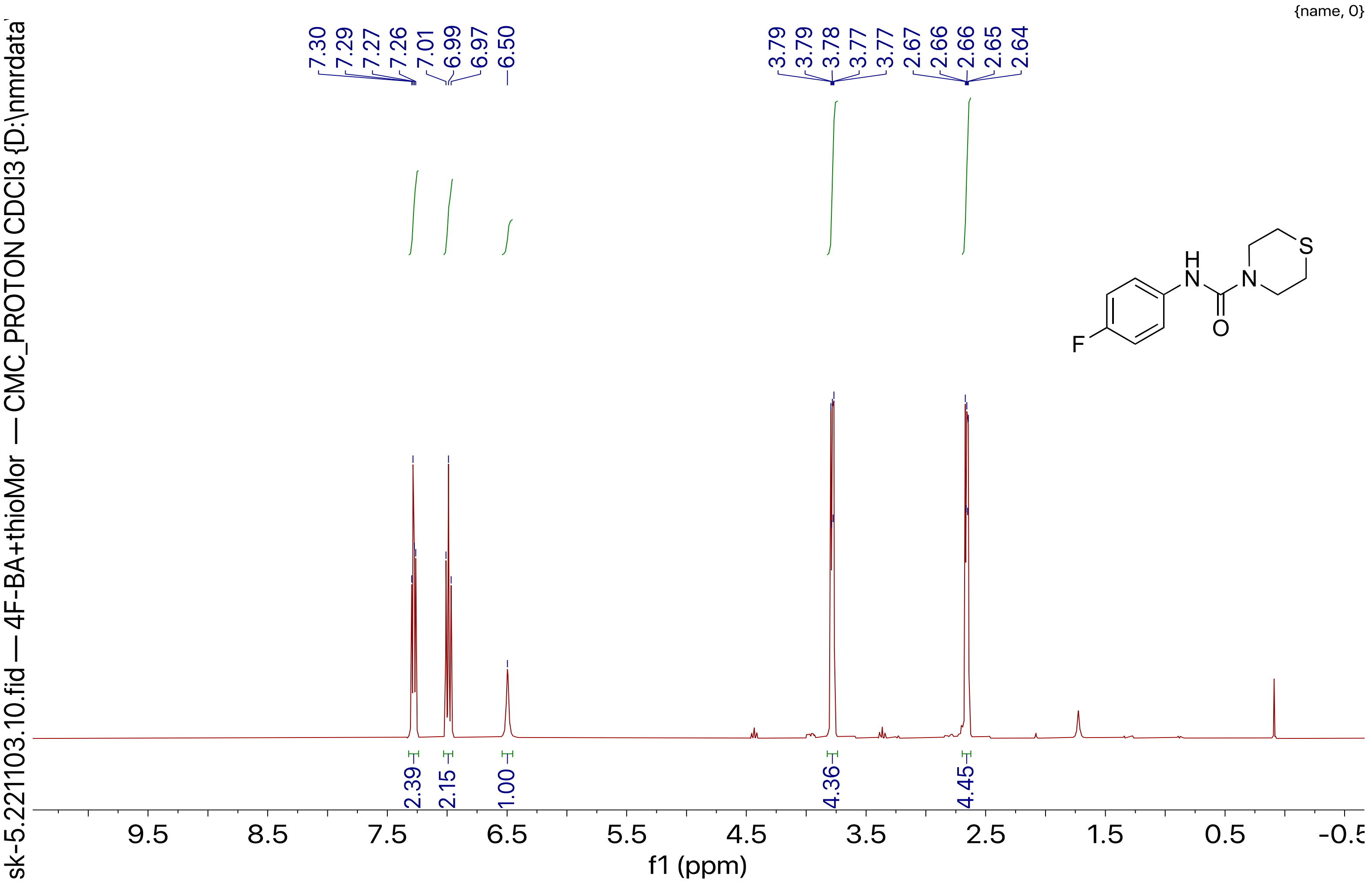

^{1}H NMR spectra of **7j** (400 MHz, RT, $CDCl_3$)

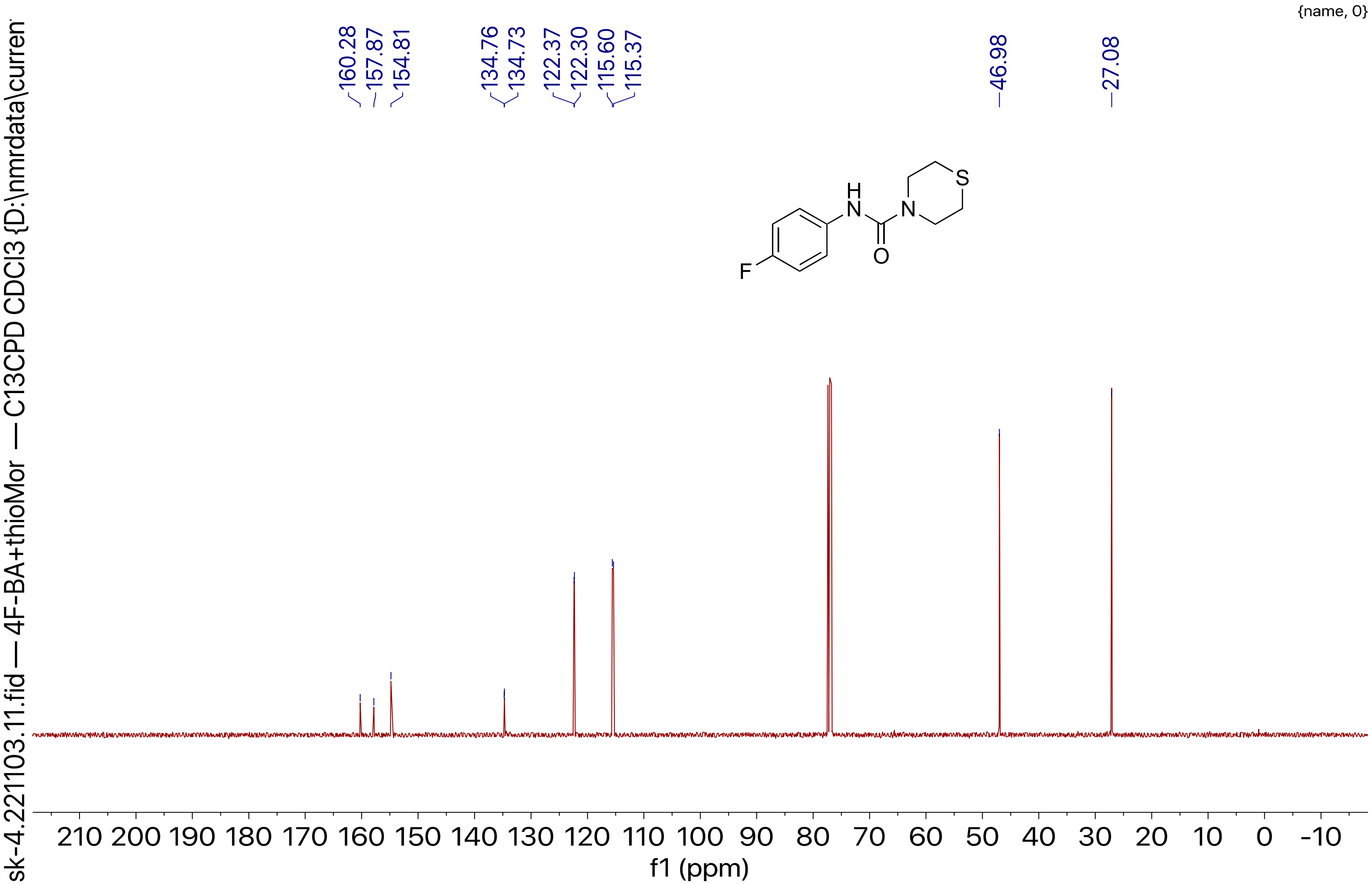

^{13}C NMR spectra of **7j** (101 MHz, RT, $CDCl_3$)

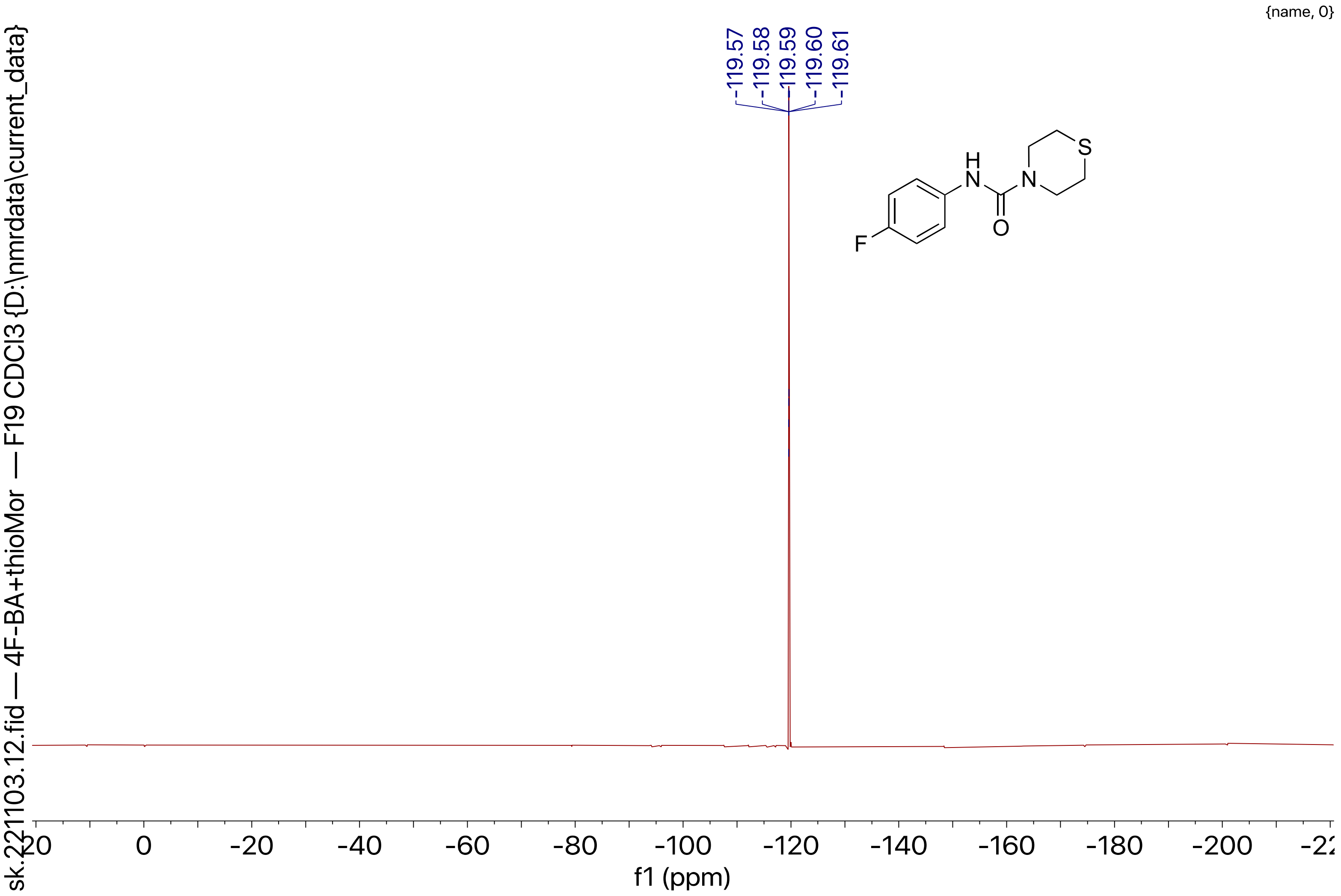

^{19}F NMR spectra of **7j** (376 MHz, RT, $CDCl_3$)

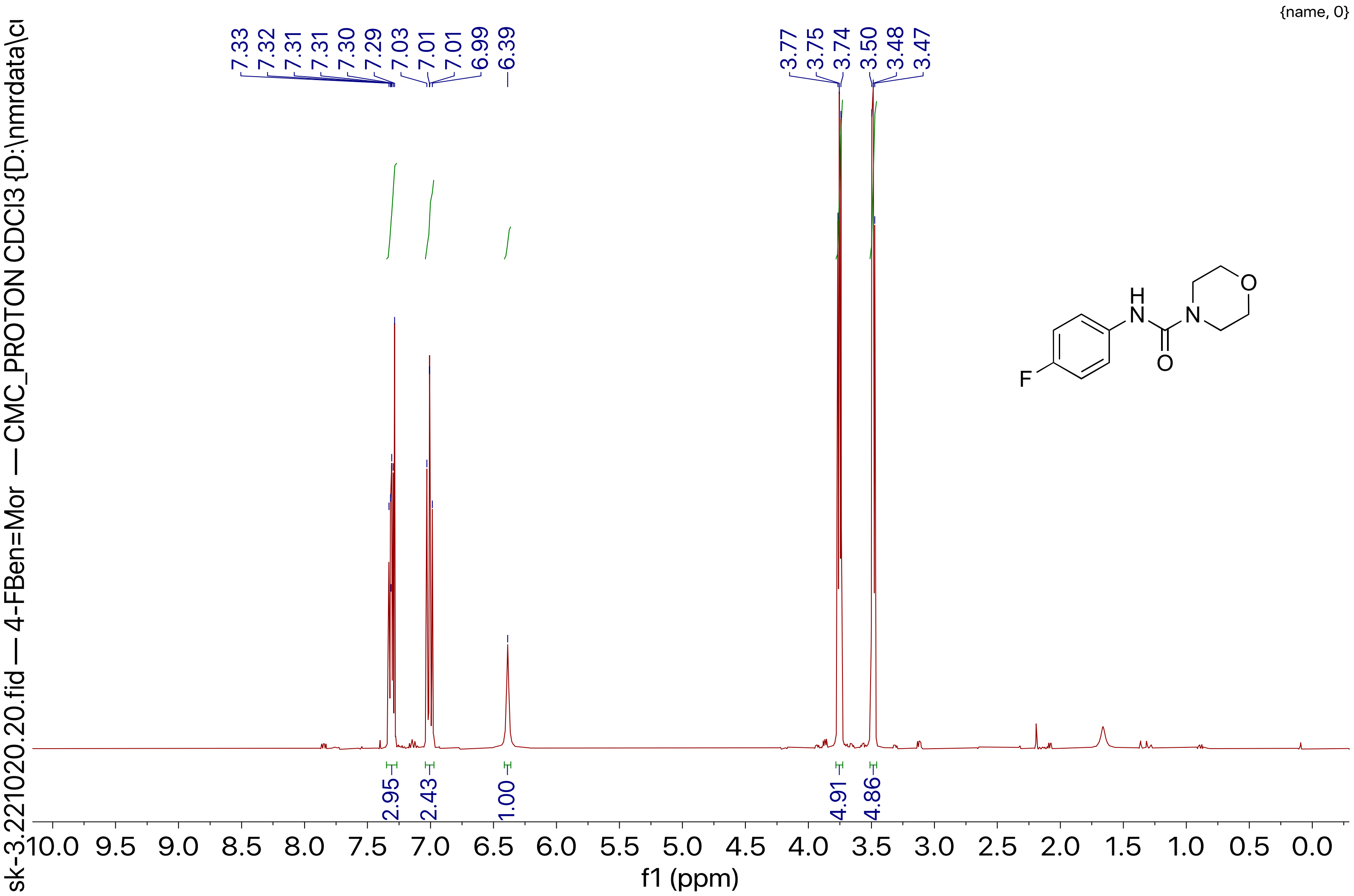

^{1}H NMR spectra of **7j’** (400 MHz, RT, $CDCl_3$)

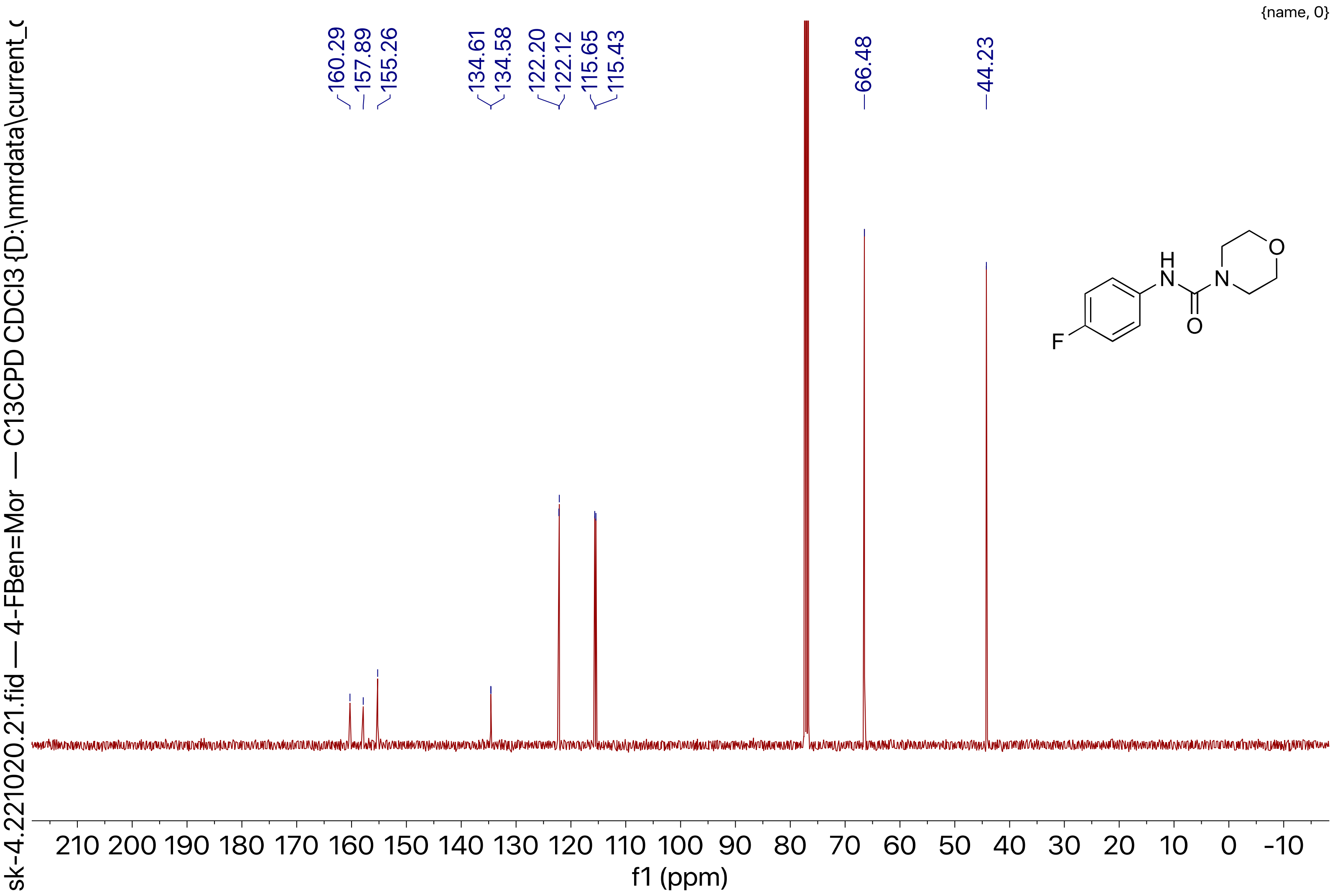

^{13}C NMR spectra of **7j'** (101 MHz, RT, $CDCl_3$)

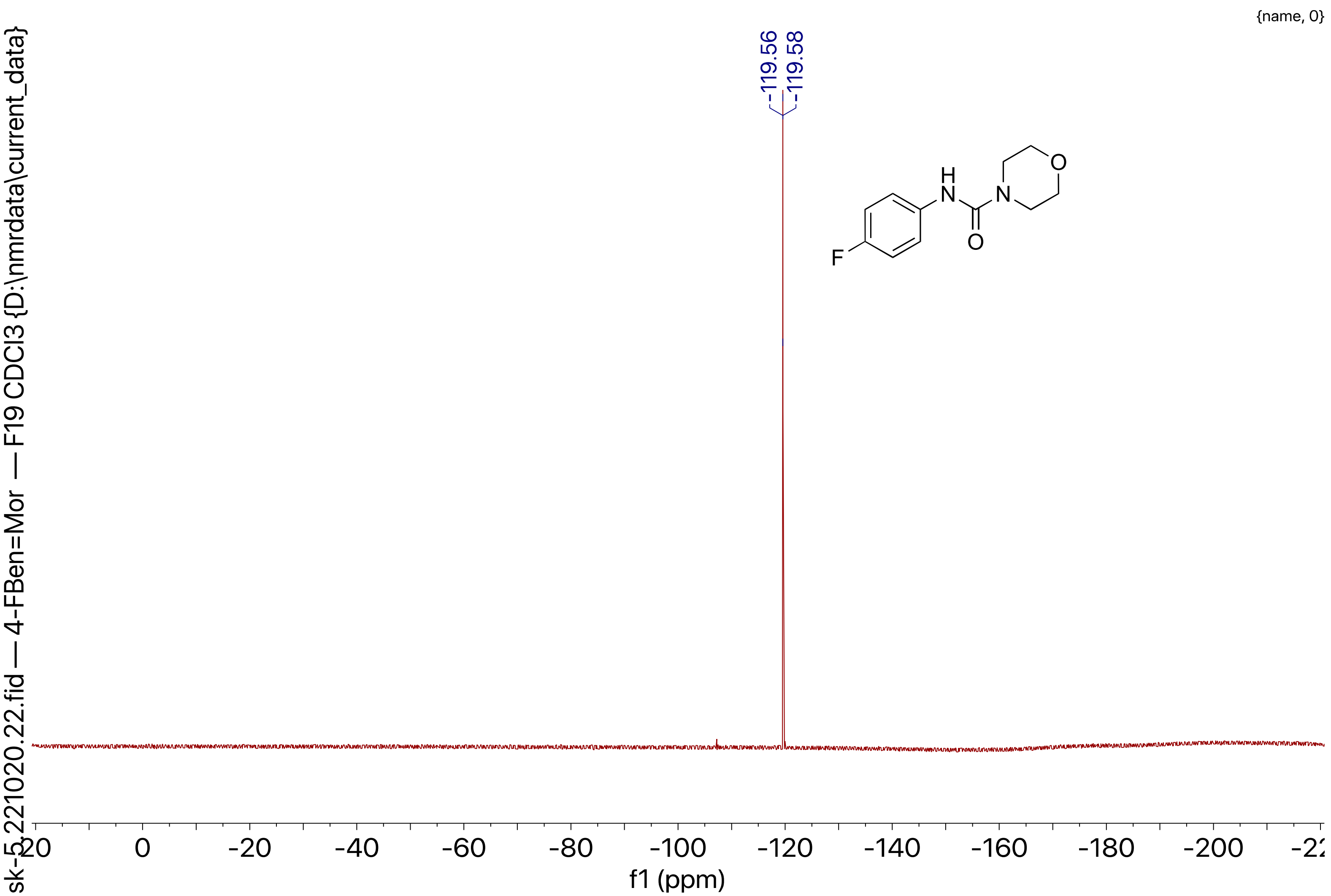

^{19}F NMR spectra of **7j’** (376 MHz, RT, $CDCl_3$)

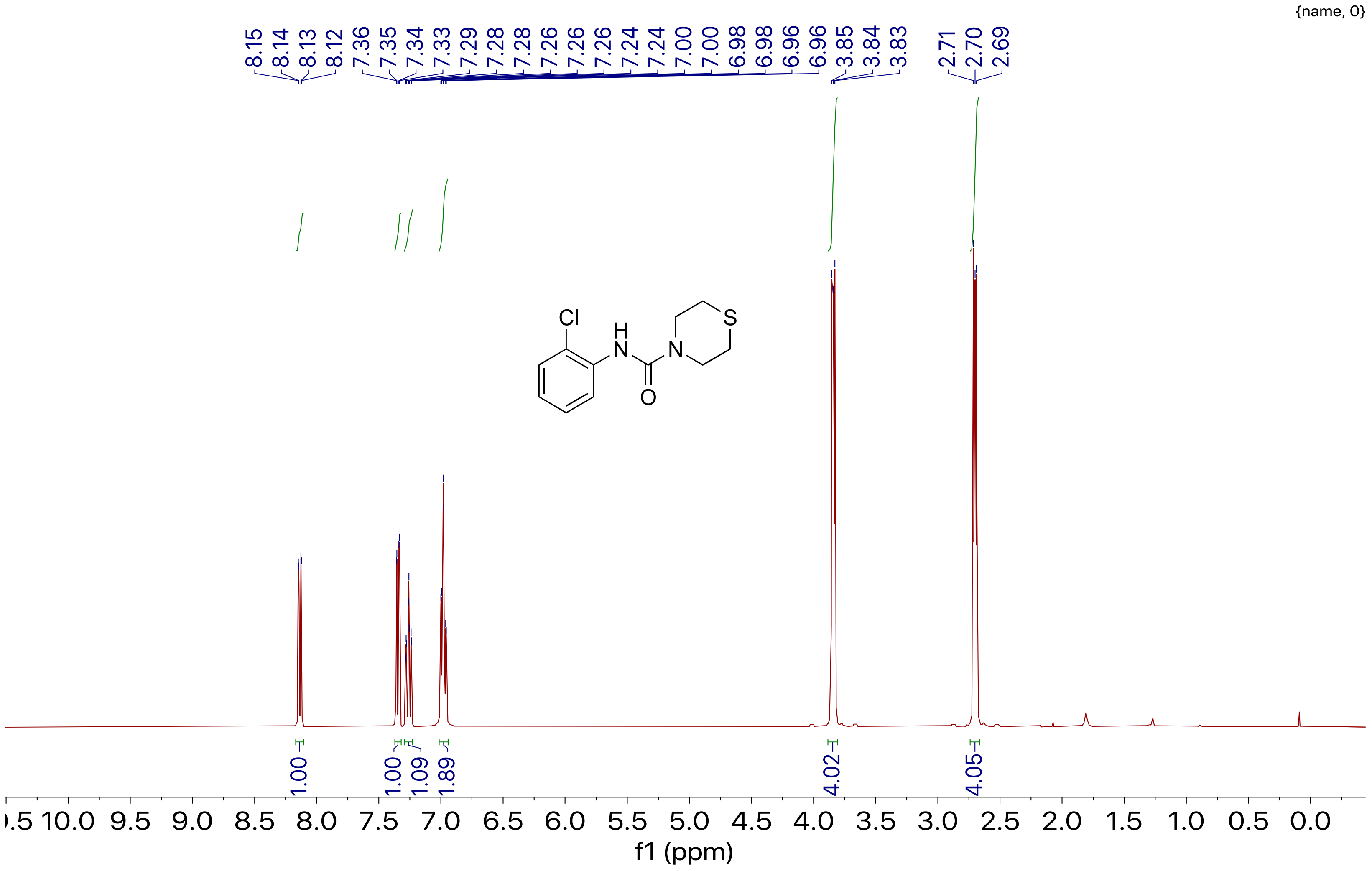

^{1}H NMR spectra of **7k** (400 MHz, RT, $CDCl_3$)

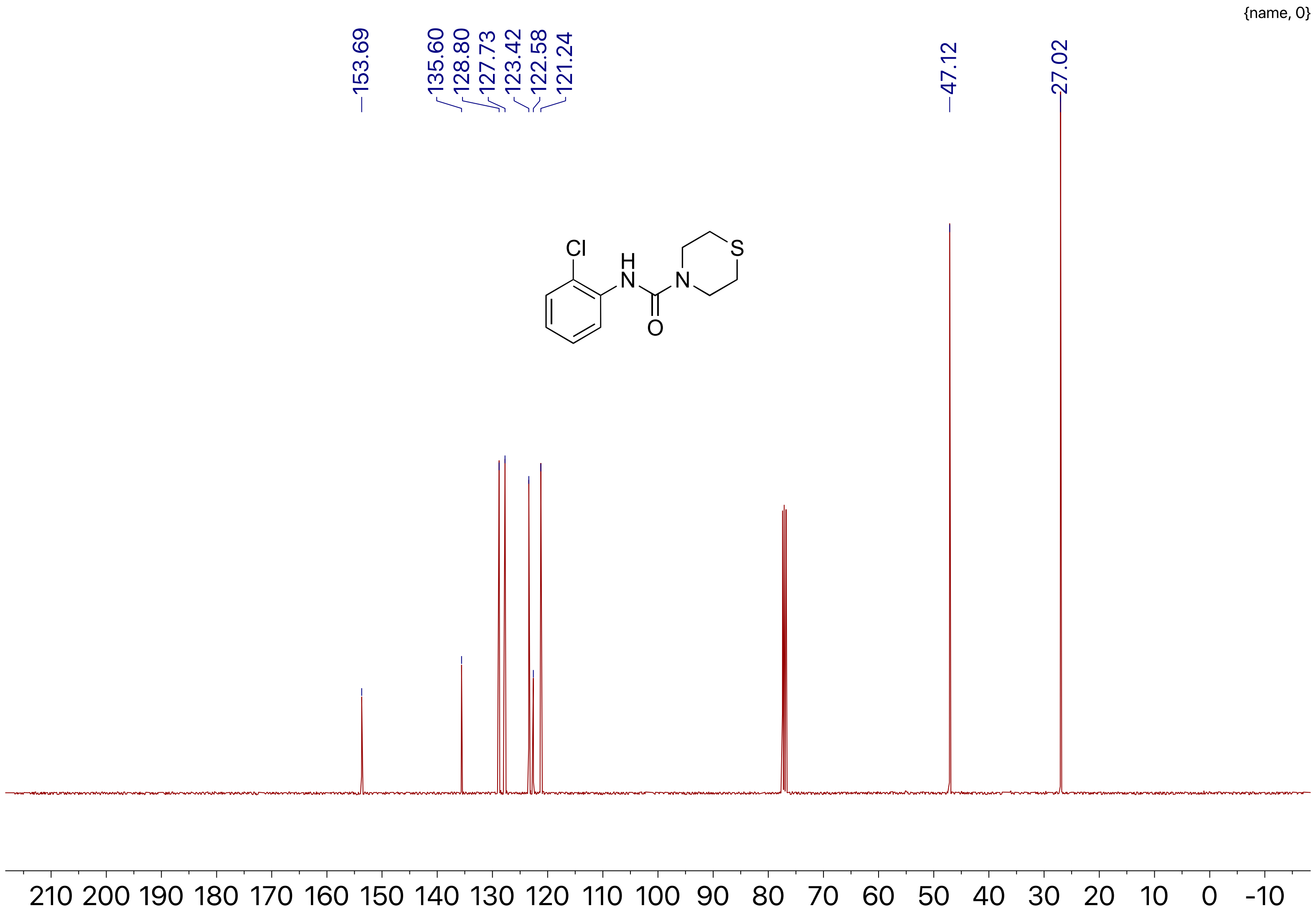

^{13}C NMR spectra of **7k** (101 MHz, RT, $CDCl_3$)

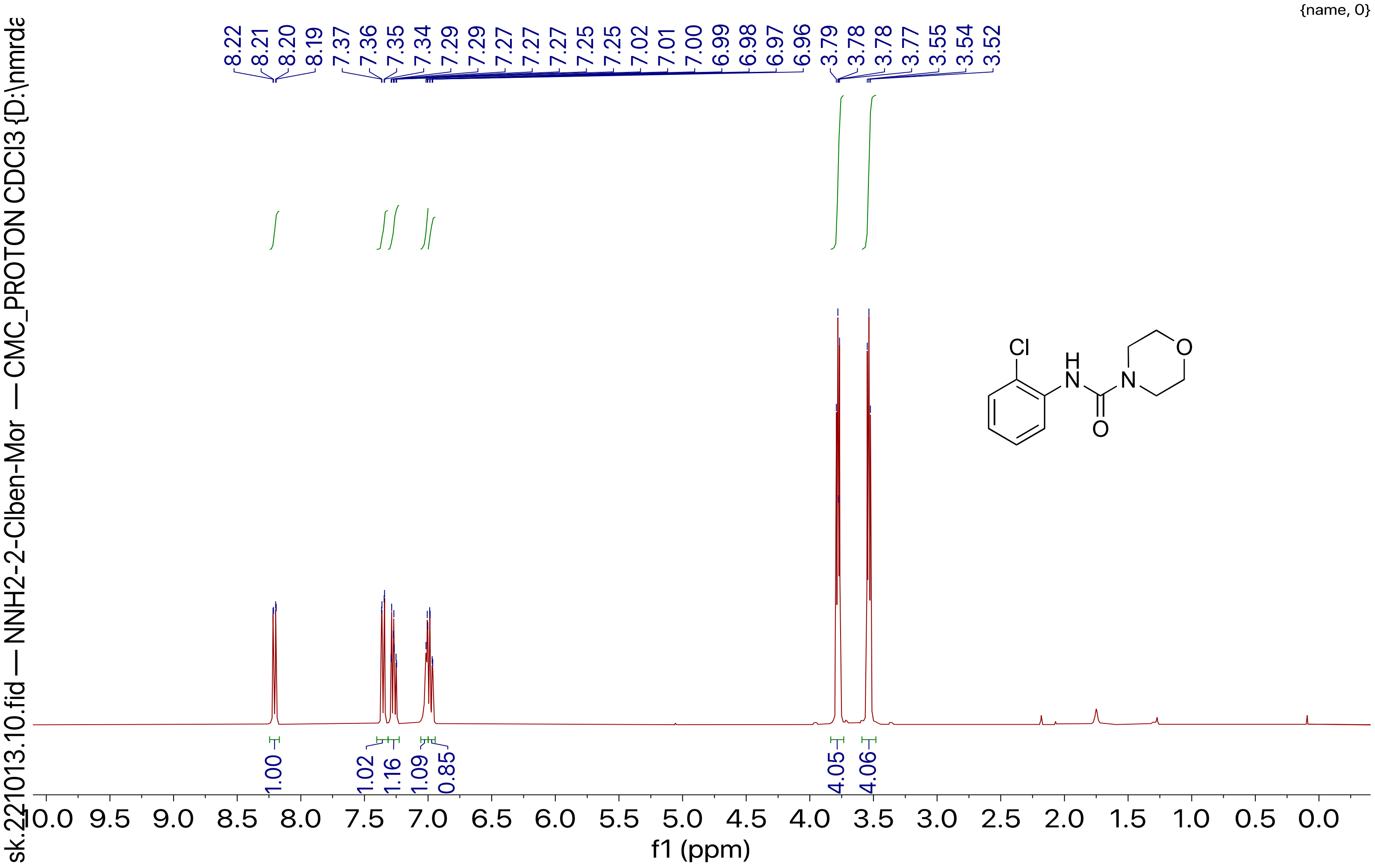

^{1}H NMR spectra of **7k’** (400 MHz, RT, $CDCl_3$)

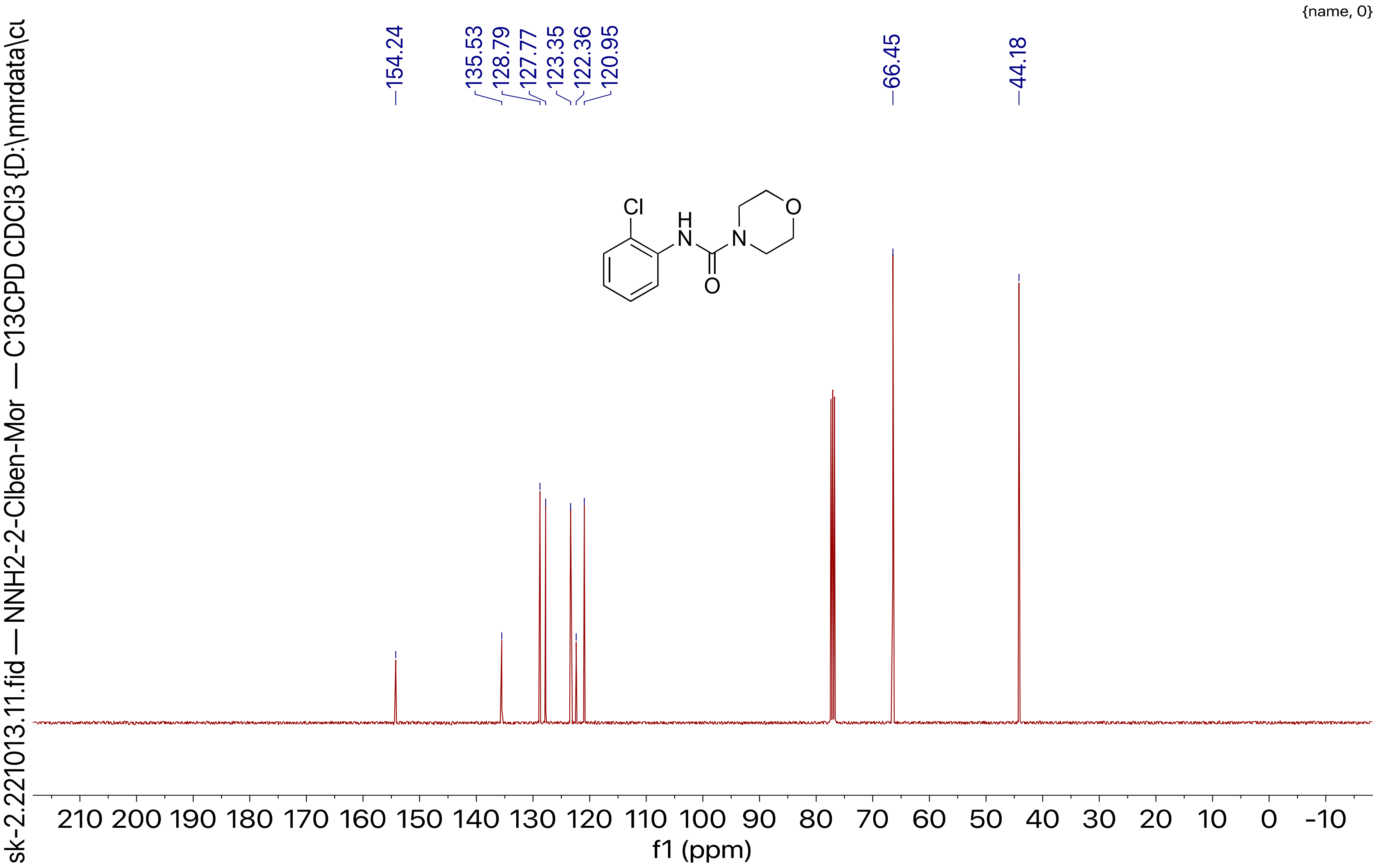

^{13}C NMR spectra of **7k'** (101 MHz, RT,$CDCl_3$)

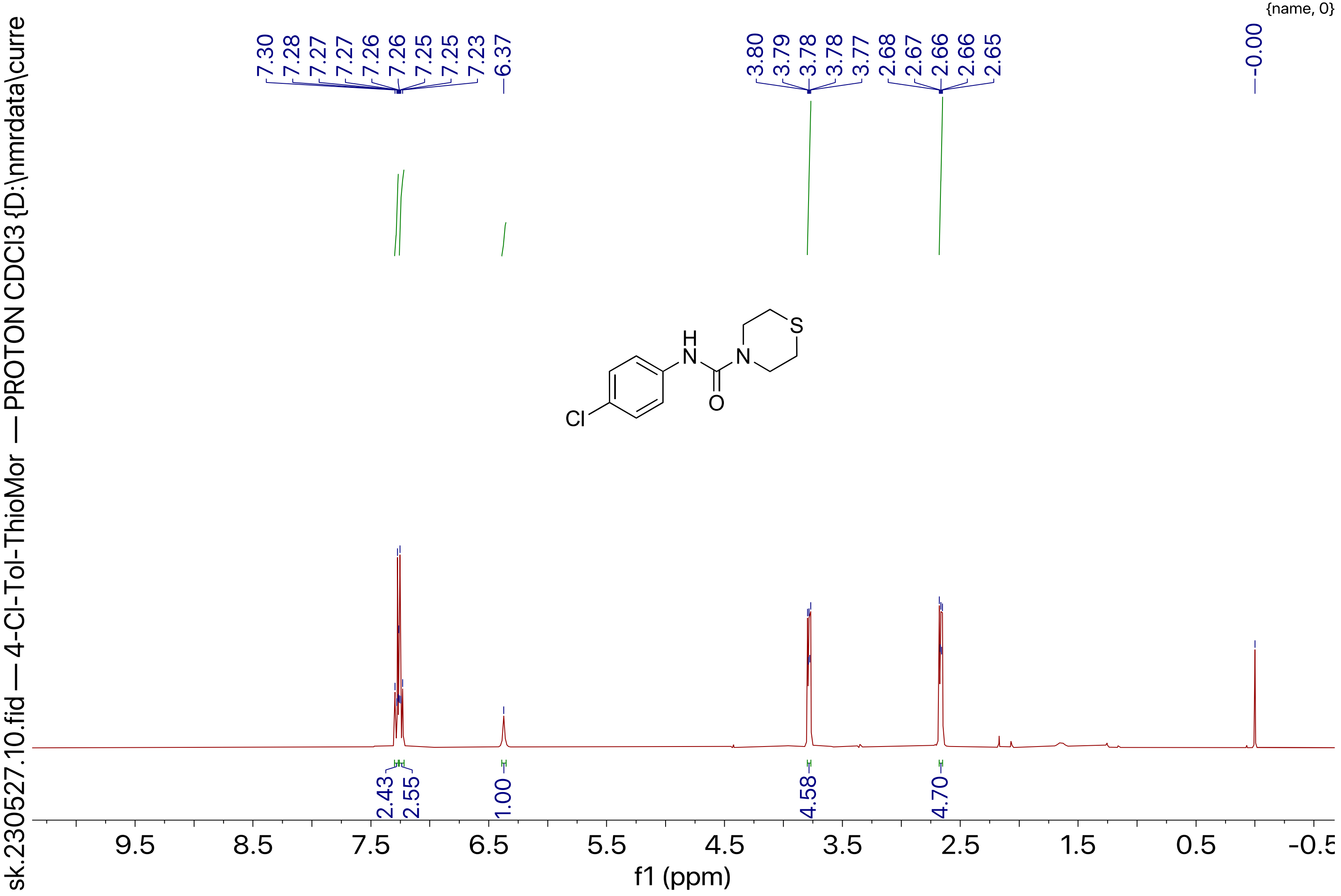

^{1}H NMR spectra of **7l** (400 MHz, RT, $CDCl_3$)

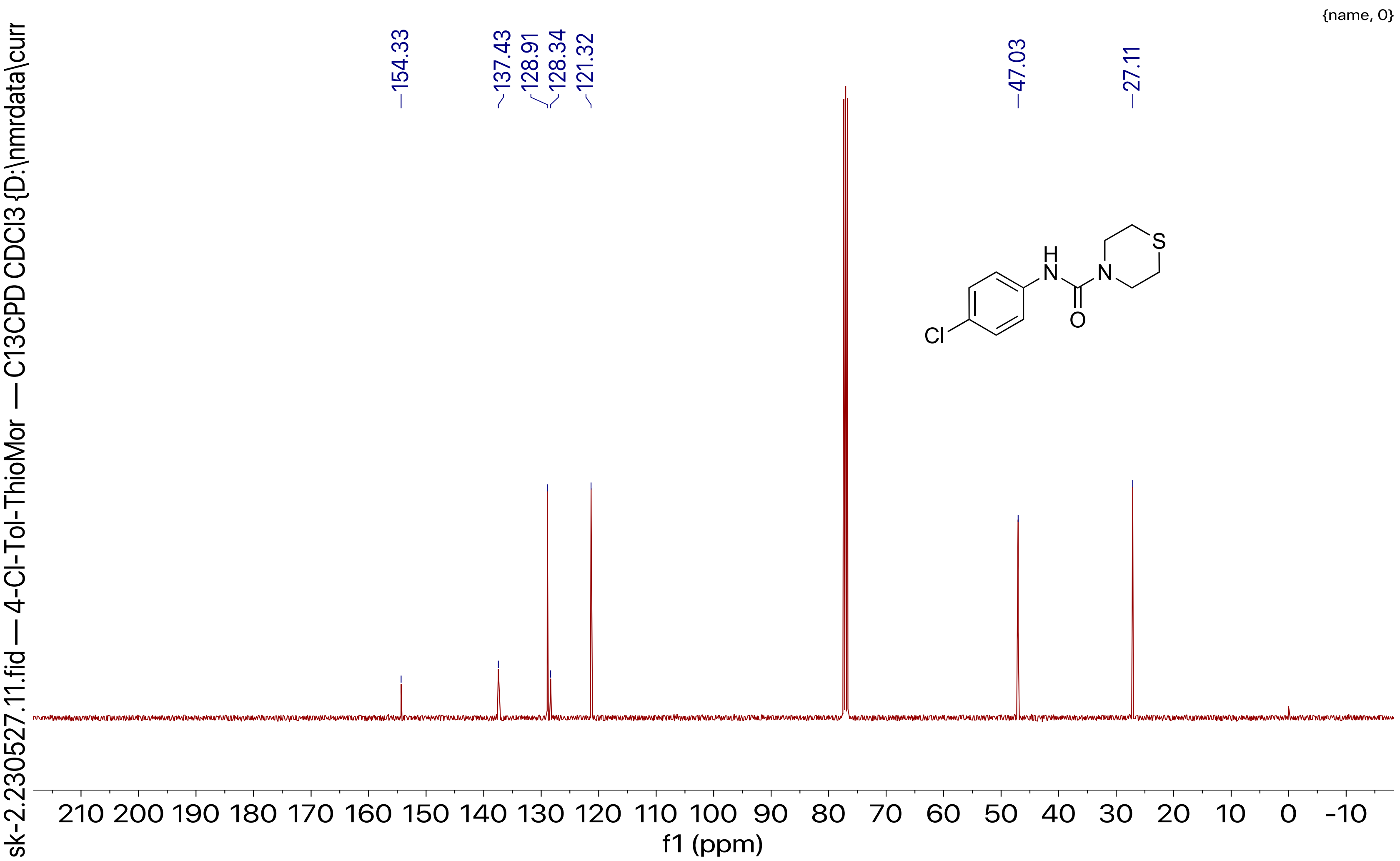

^{13}C NMR spectra of **7l** (101 MHz, RT, $CDCl_3$)

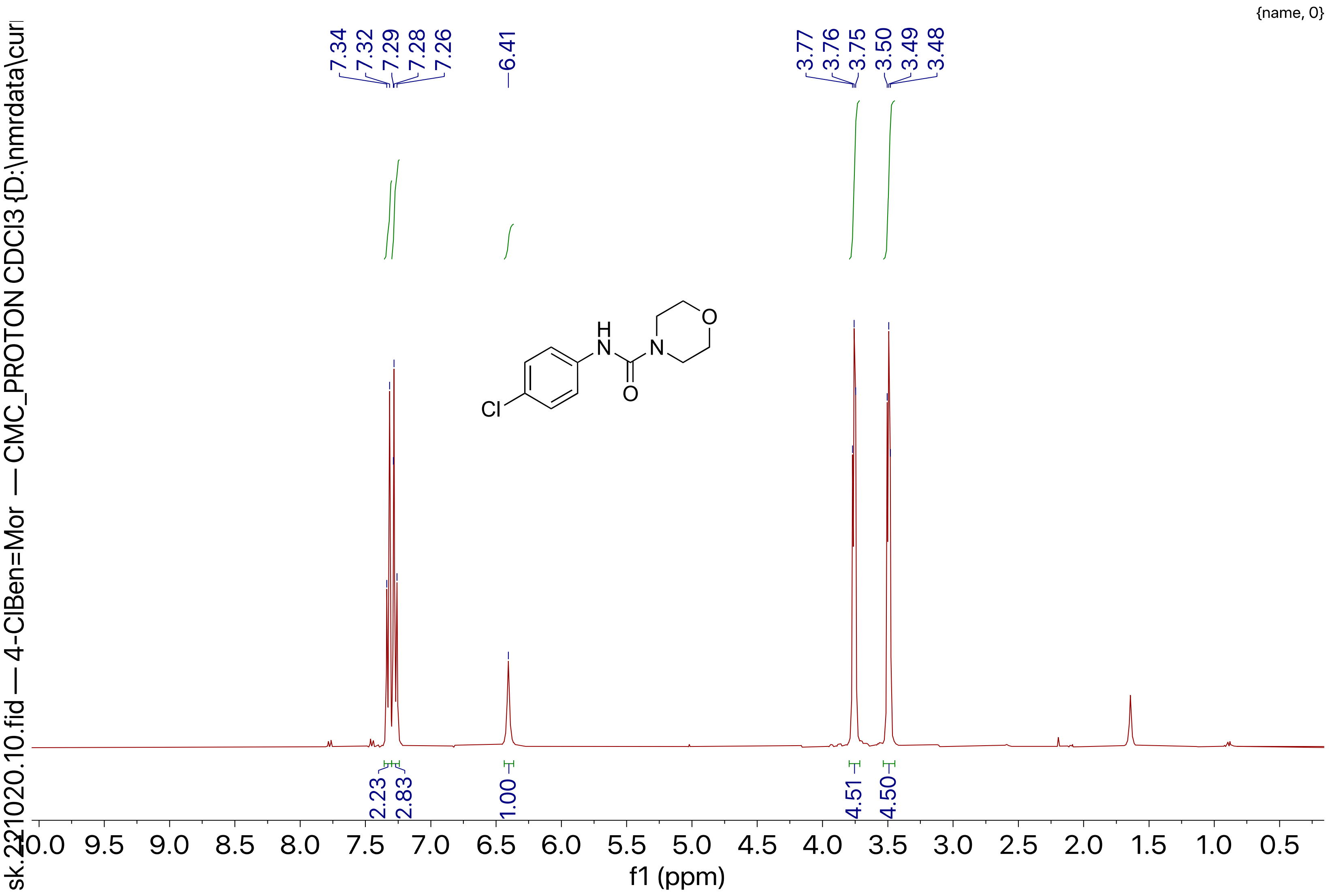

^{1}H NMR spectra of **7l'** (400 MHz, RT, $CDCl_3$)

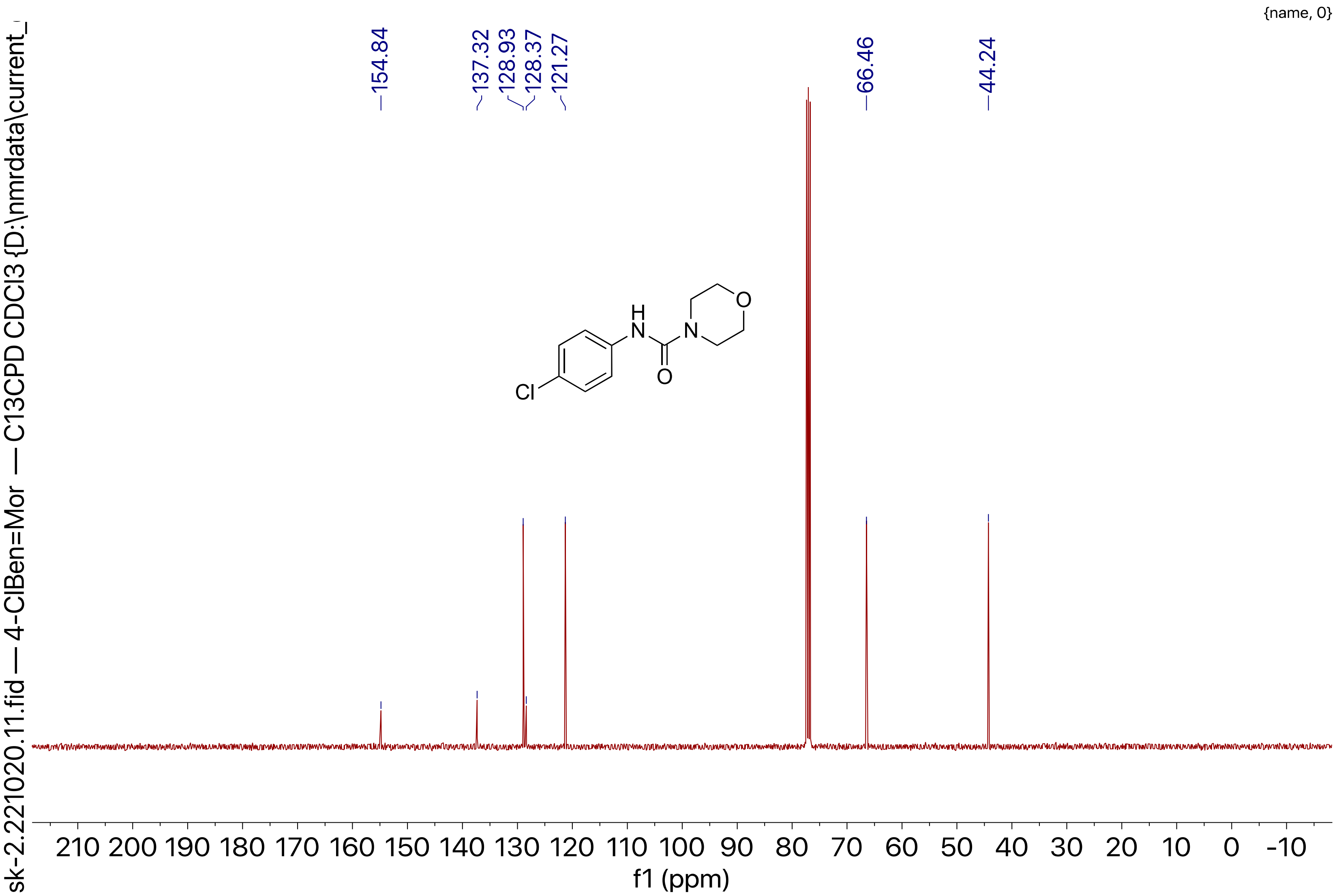

^{13}C NMR spectra of **7l’** (100 MHz, RT, $CDCl_3$)

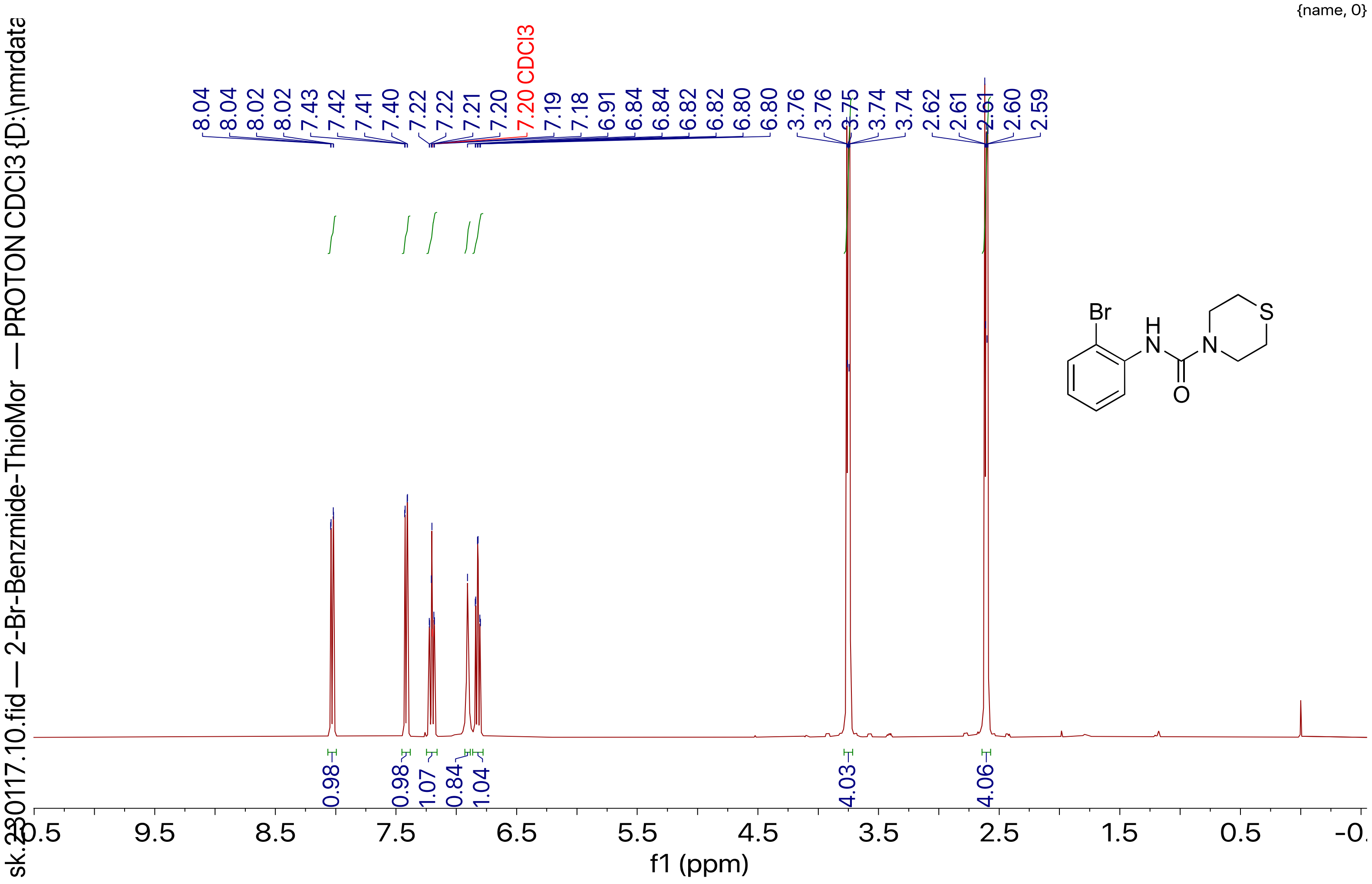

^{1}H NMR spectra of **5m** (400 MHz, RT, $CDCl_3$)

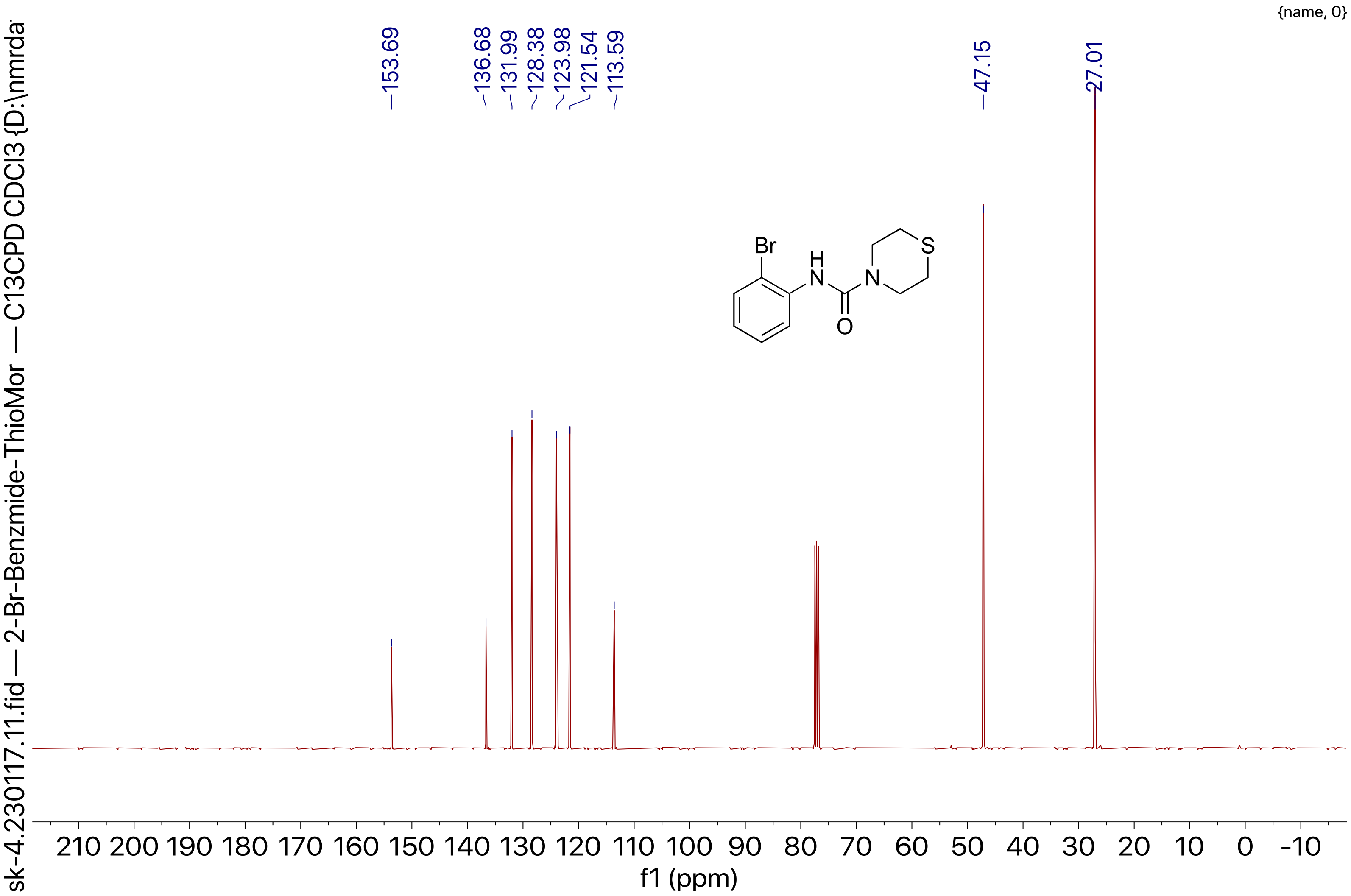

^{13}C NMR spectra of **5m** (101 MHz, RT, $CDCl_3$)

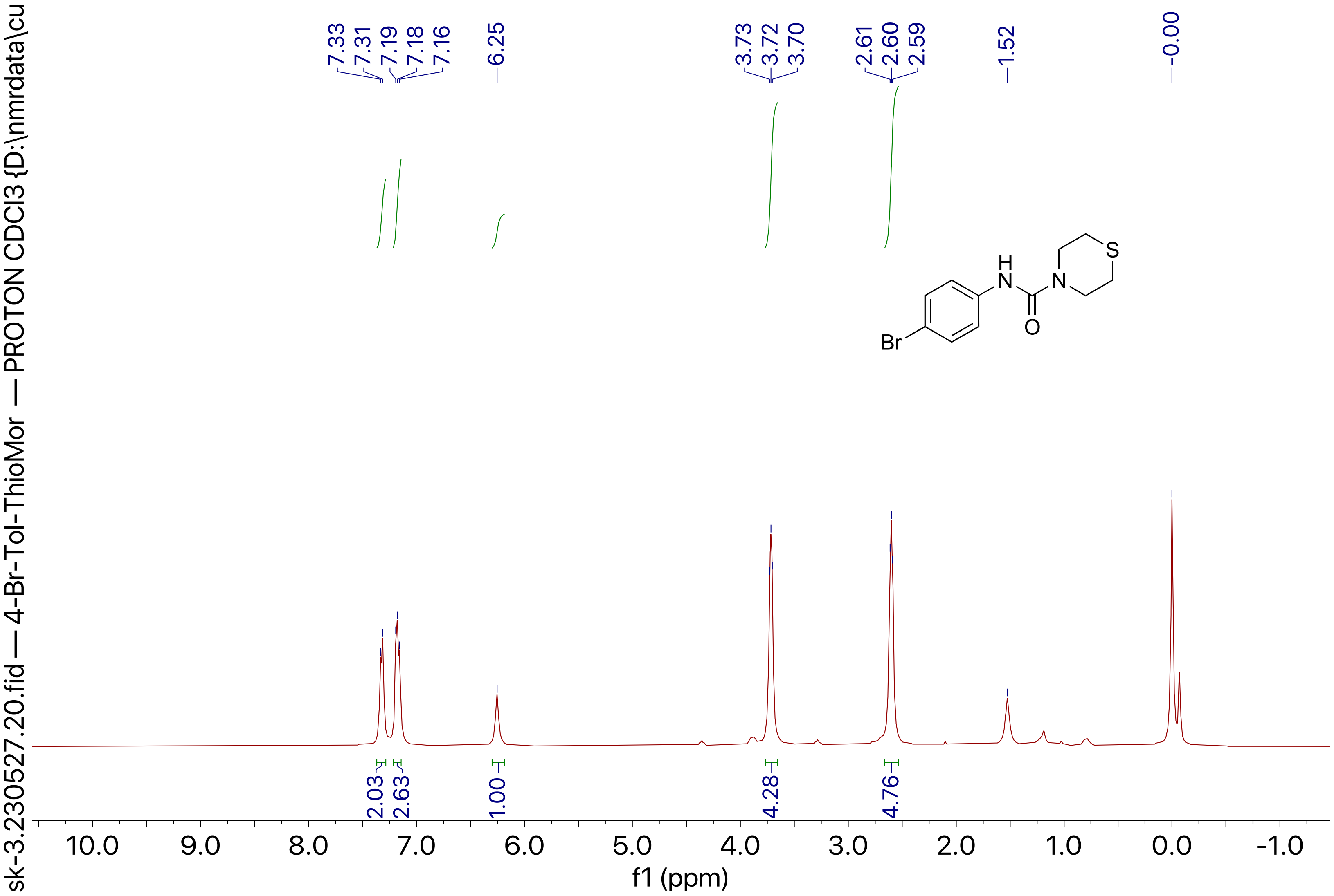

^{1}H NMR spectra of **7n** (400 MHz, RT, $CDCl_3$)

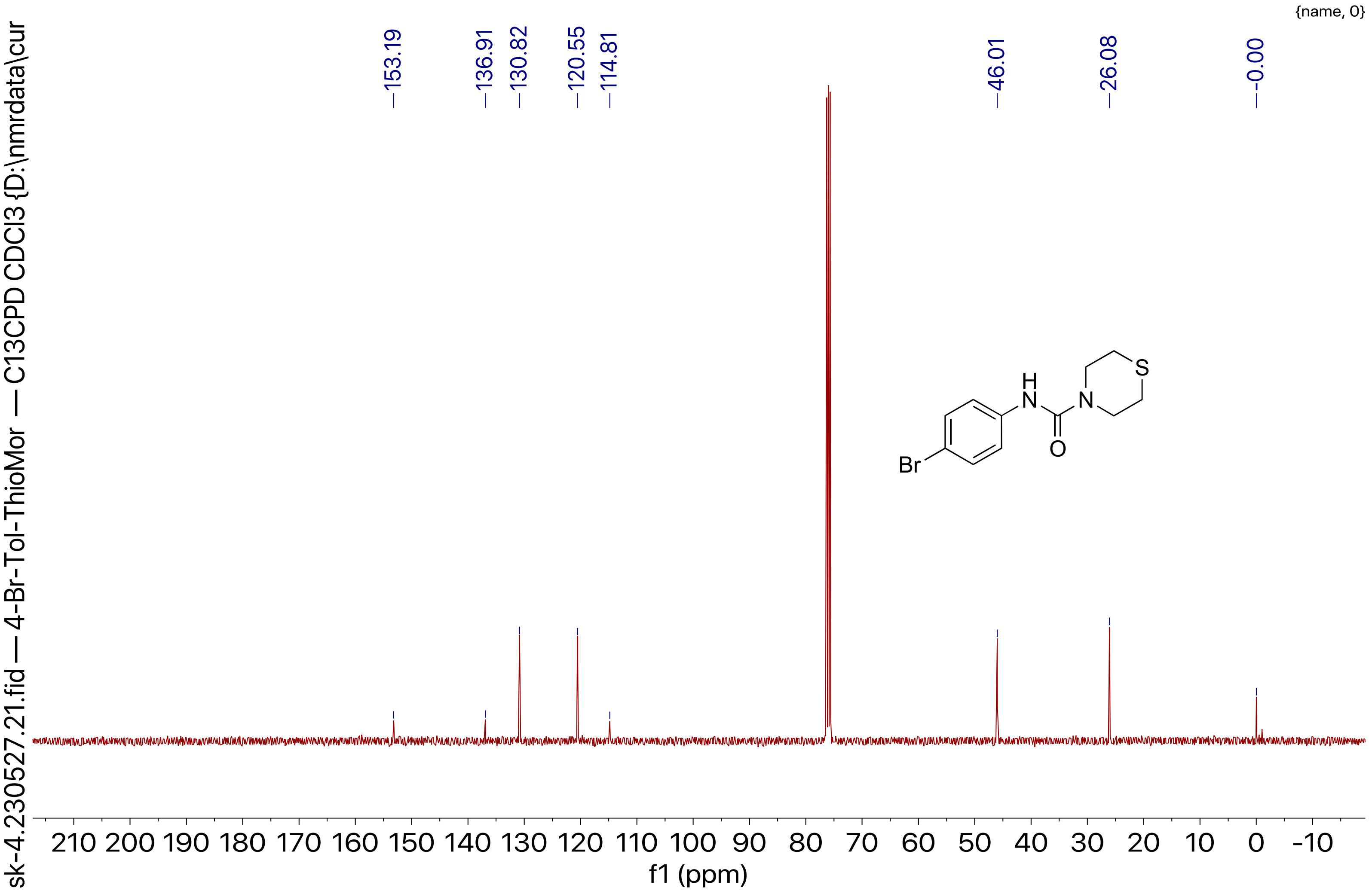

^{13}C NMR spectra of **7n** (101 MHz, RT, $CDCl_3$)

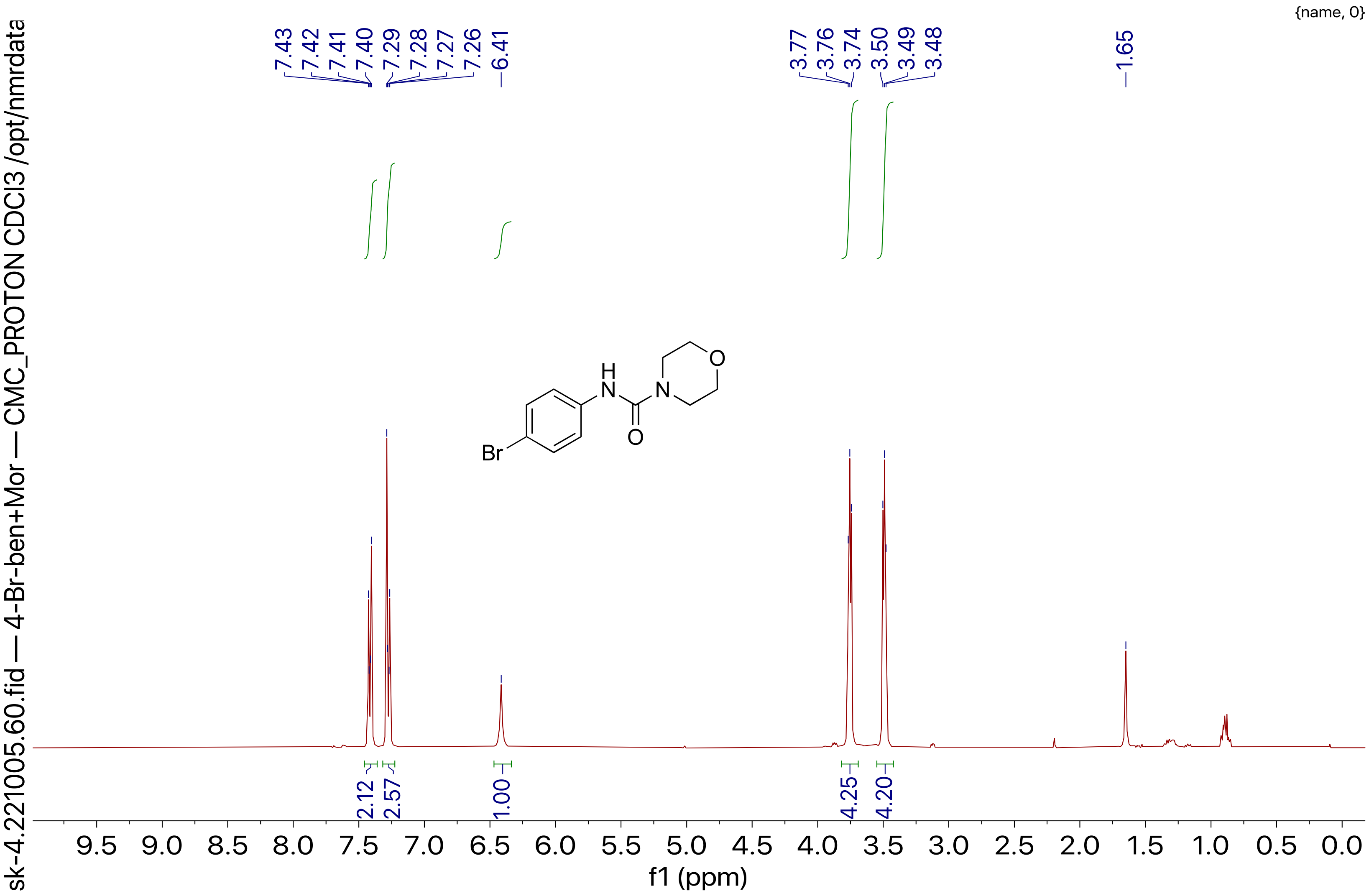

^{1}H NMR spectra of **7n’** (400 MHz, RT, $CDCl_3$)

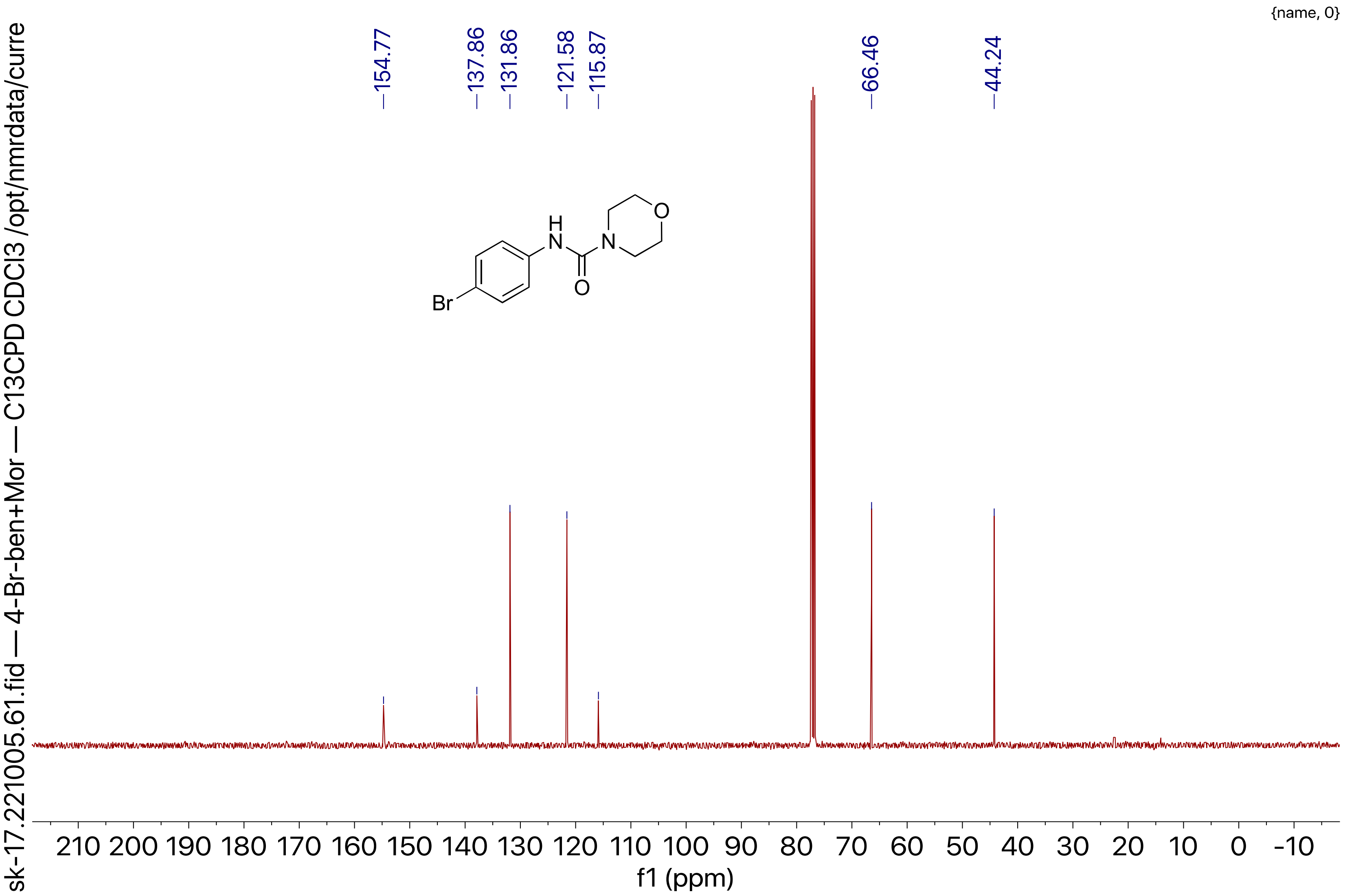

^{13}C NMR spectra of **7n’** (101 MHz, RT, $CDCl_3$)

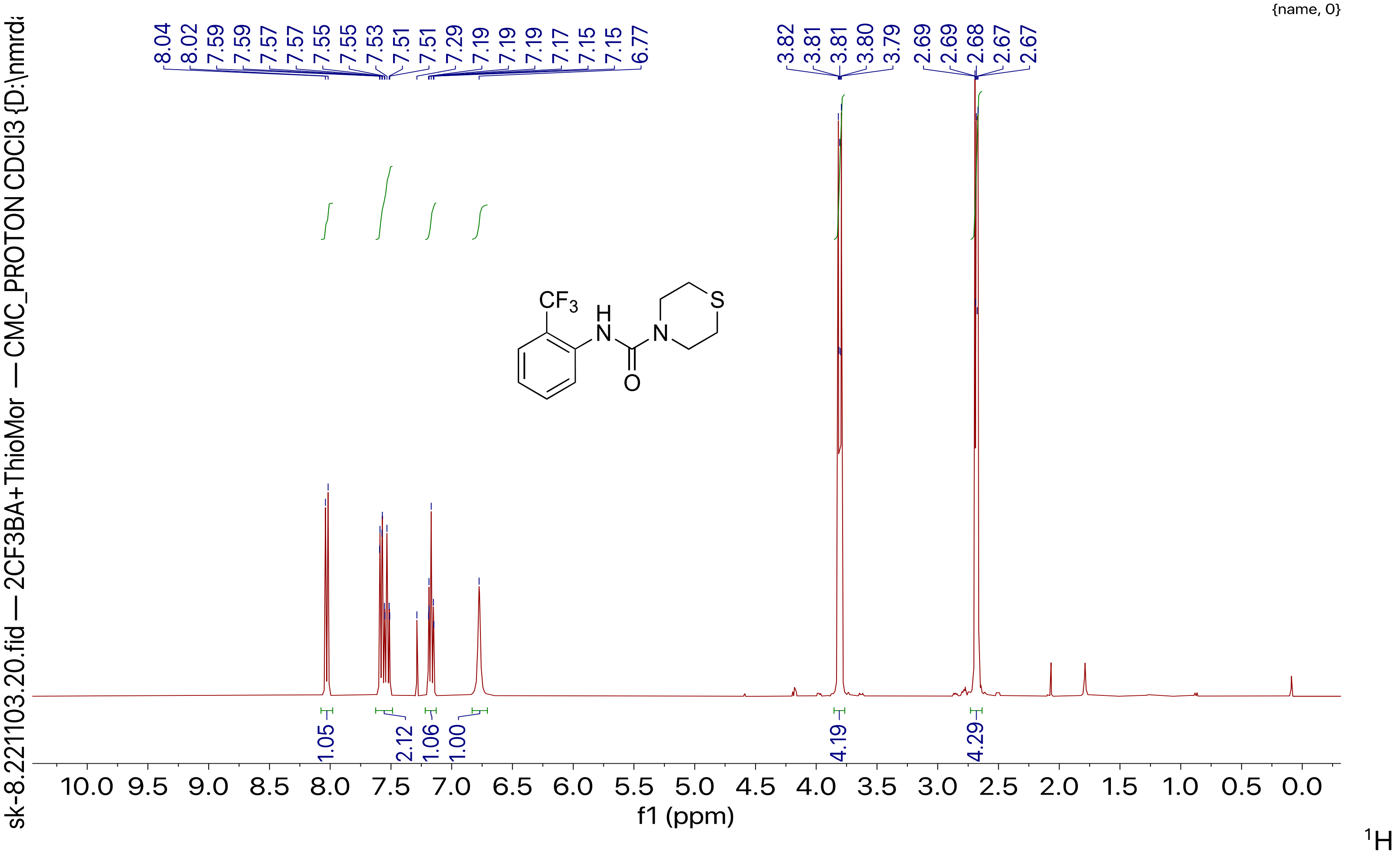

NMR spectra of **7o** (400 MHz, RT, $CDCl_3$)

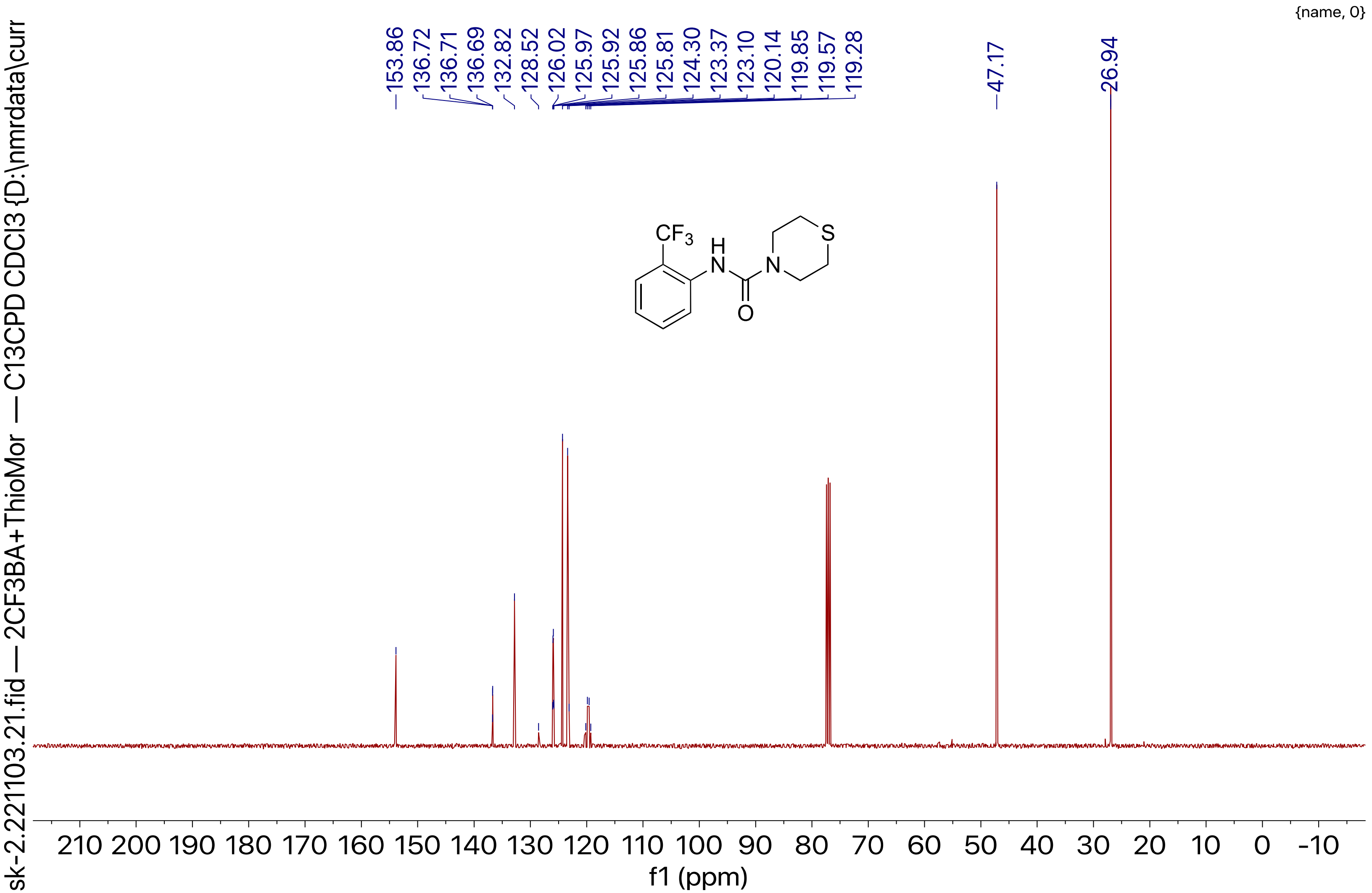

^{13}C NMR spectra of **7o** (100 MHz, RT, $CDCl_3$)

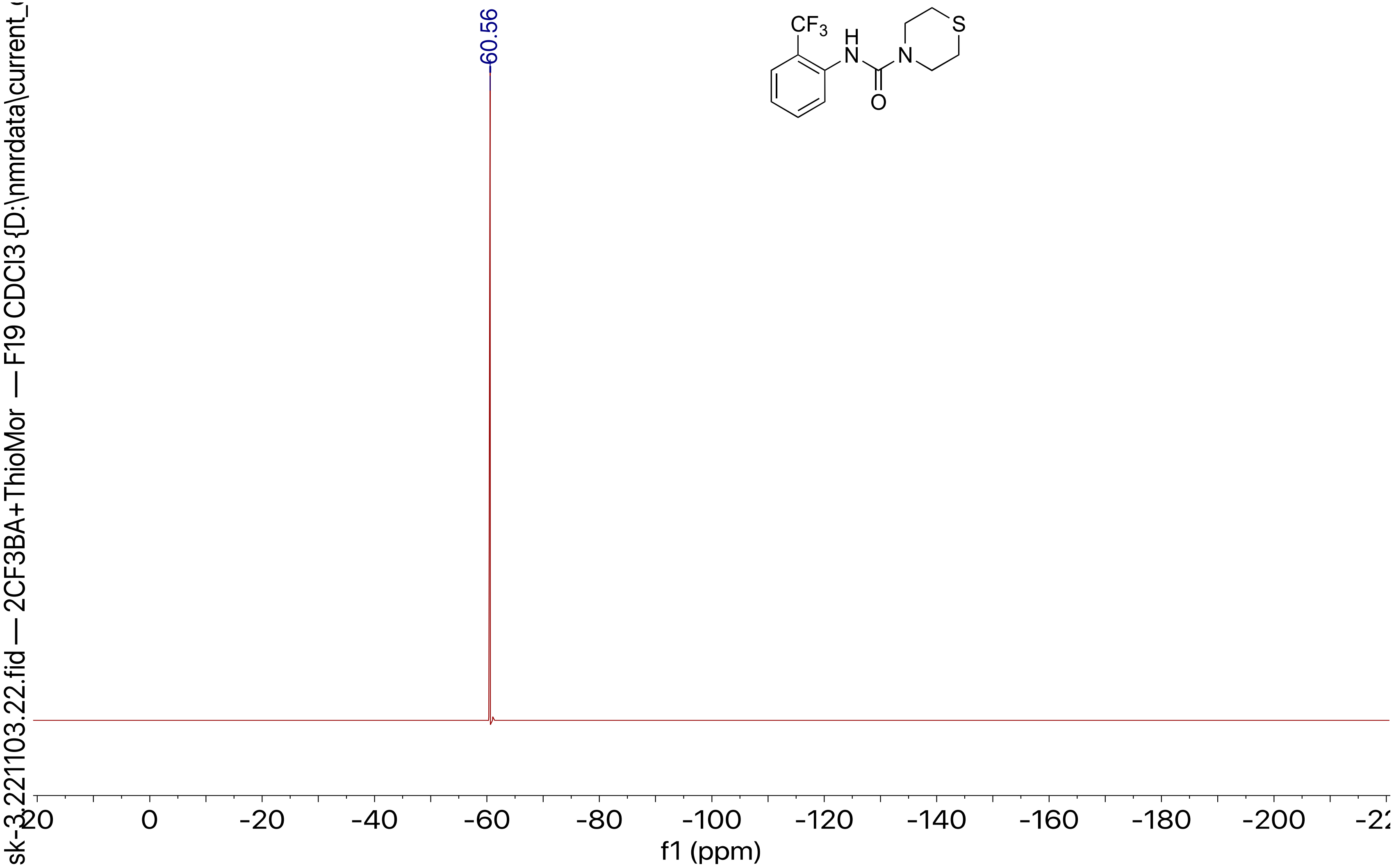

^{19}F NMR spectra of **7o** (376 MHz, RT, $CDCl_3$)

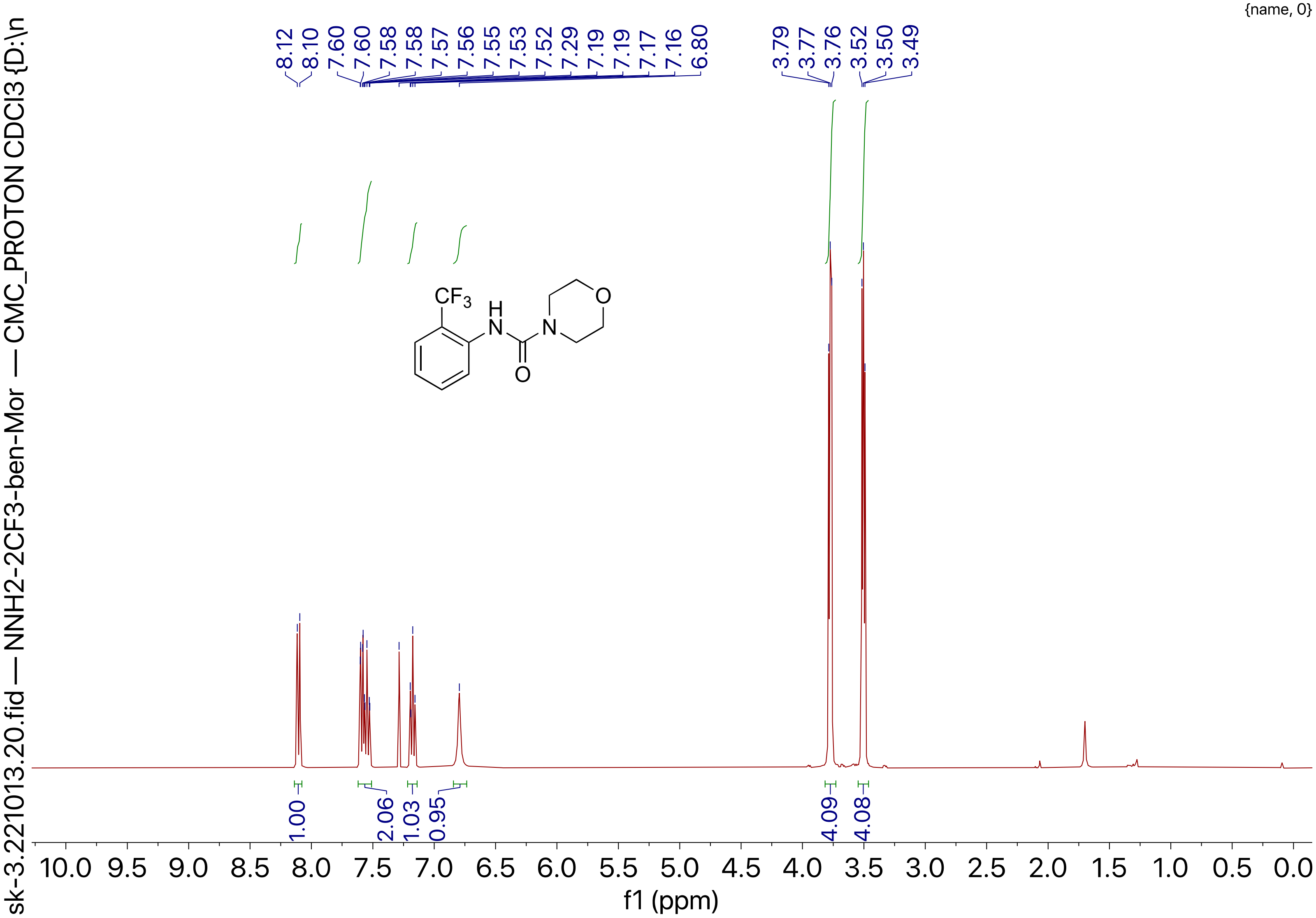

^{1}H NMR spectra of **7o’** (400 MHz, RT, $CDCl_3$)

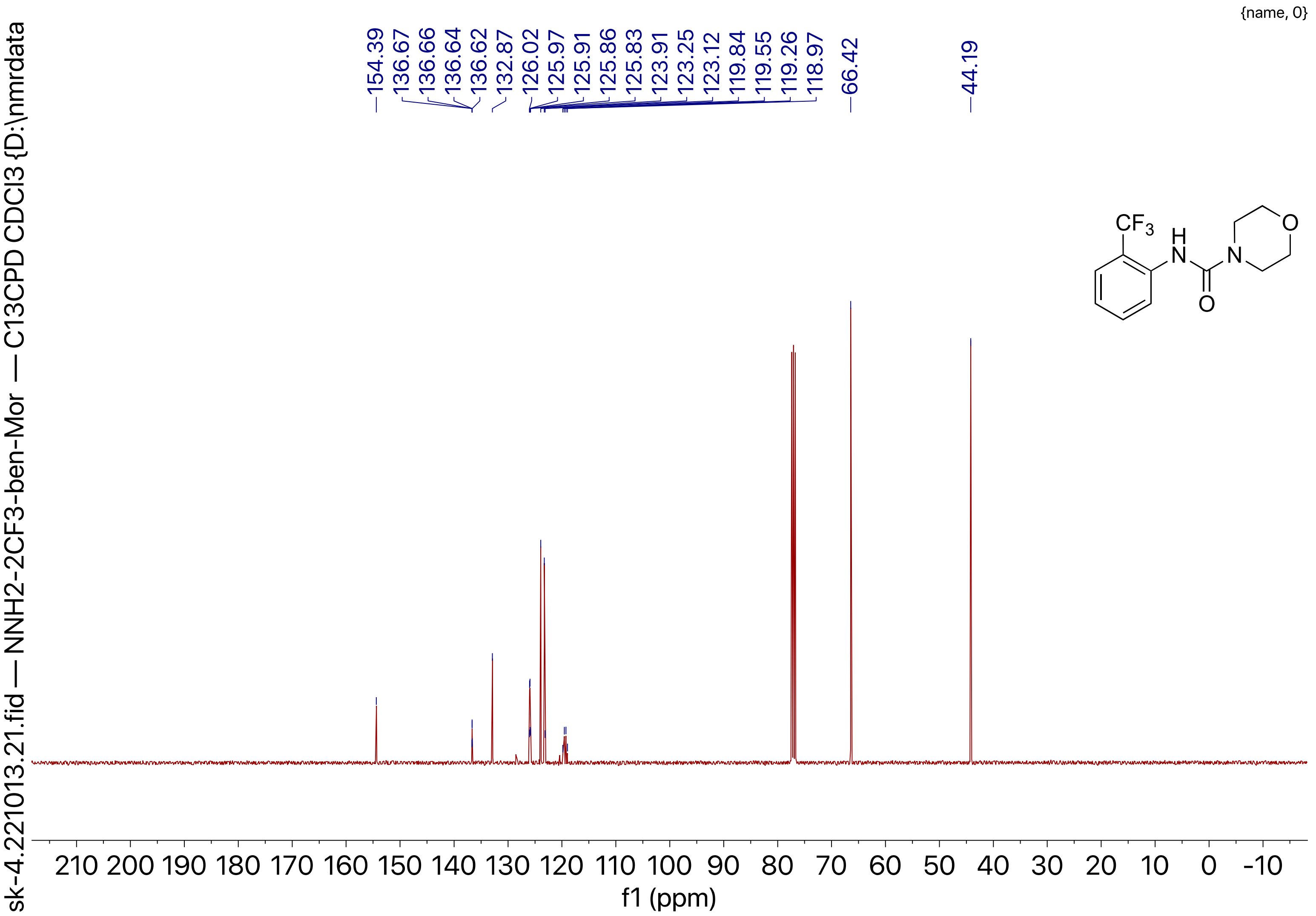

^{13}C NMR spectra of **7o'** (101 MHz, RT, $CDCl_3$)

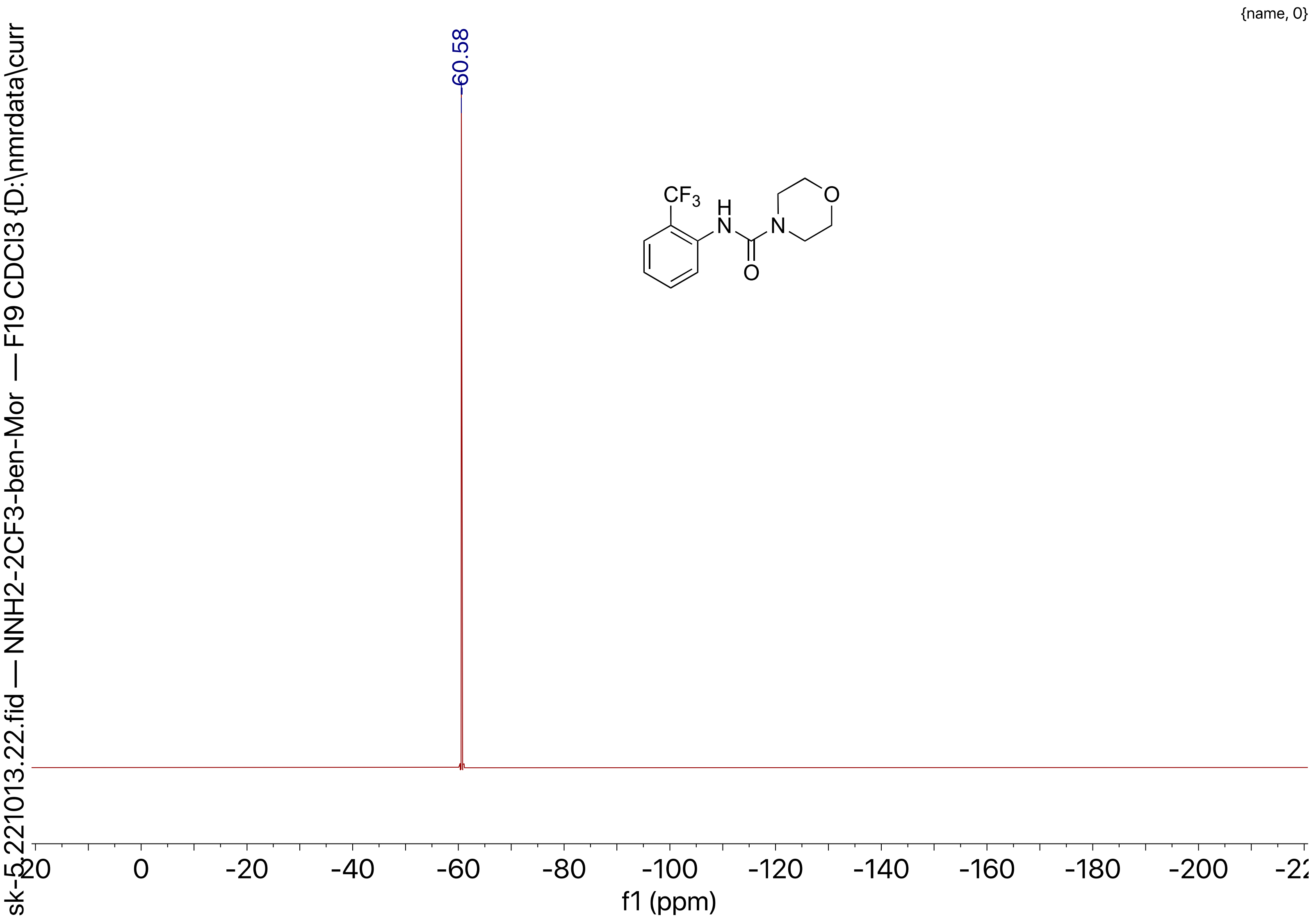

^{19}F NMR spectra of **7o'** (376 MHz, RT, $CDCl_3$)

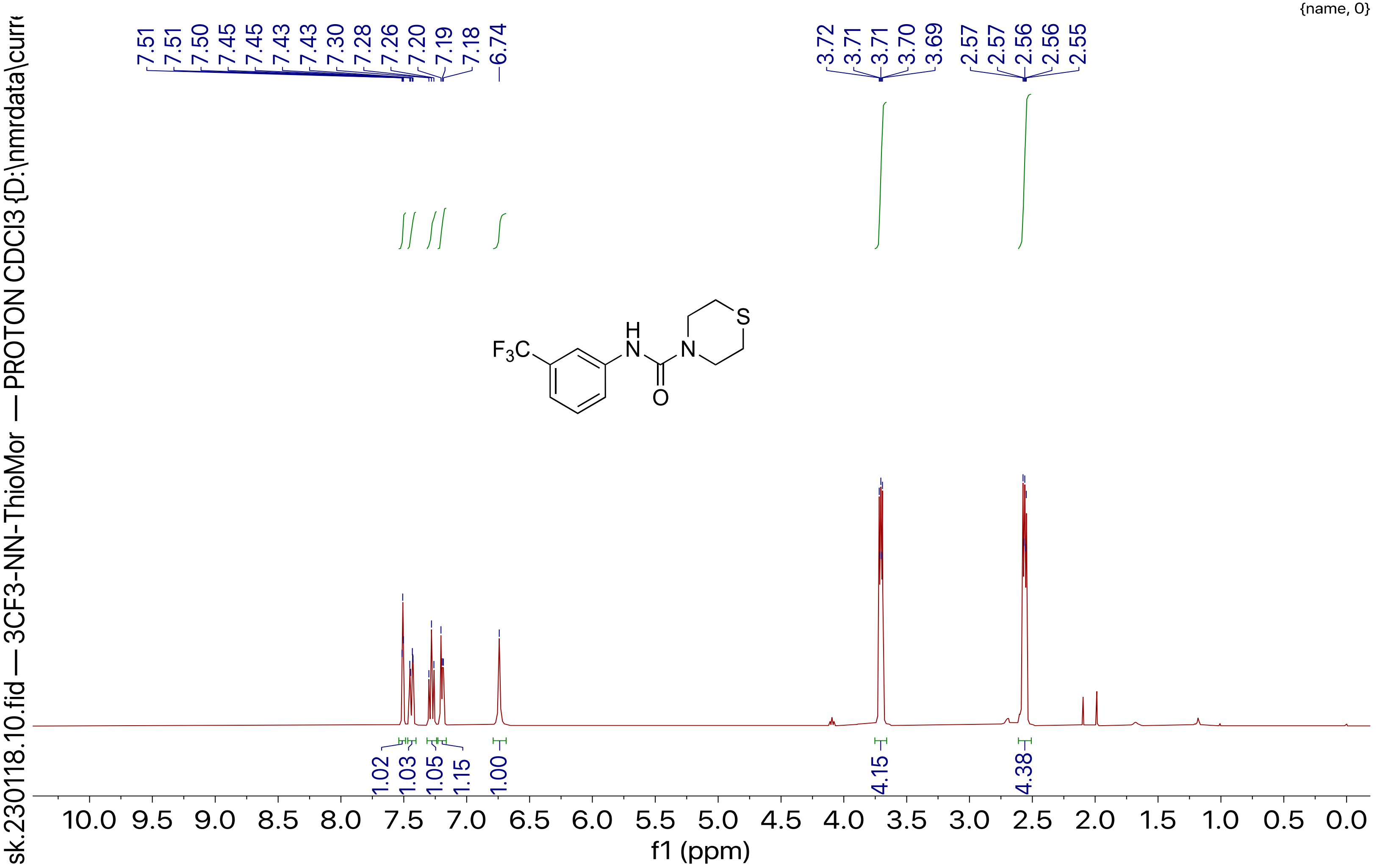

^{1}H NMR spectra of **7p** (400 MHz, RT, $CDCl_3$)

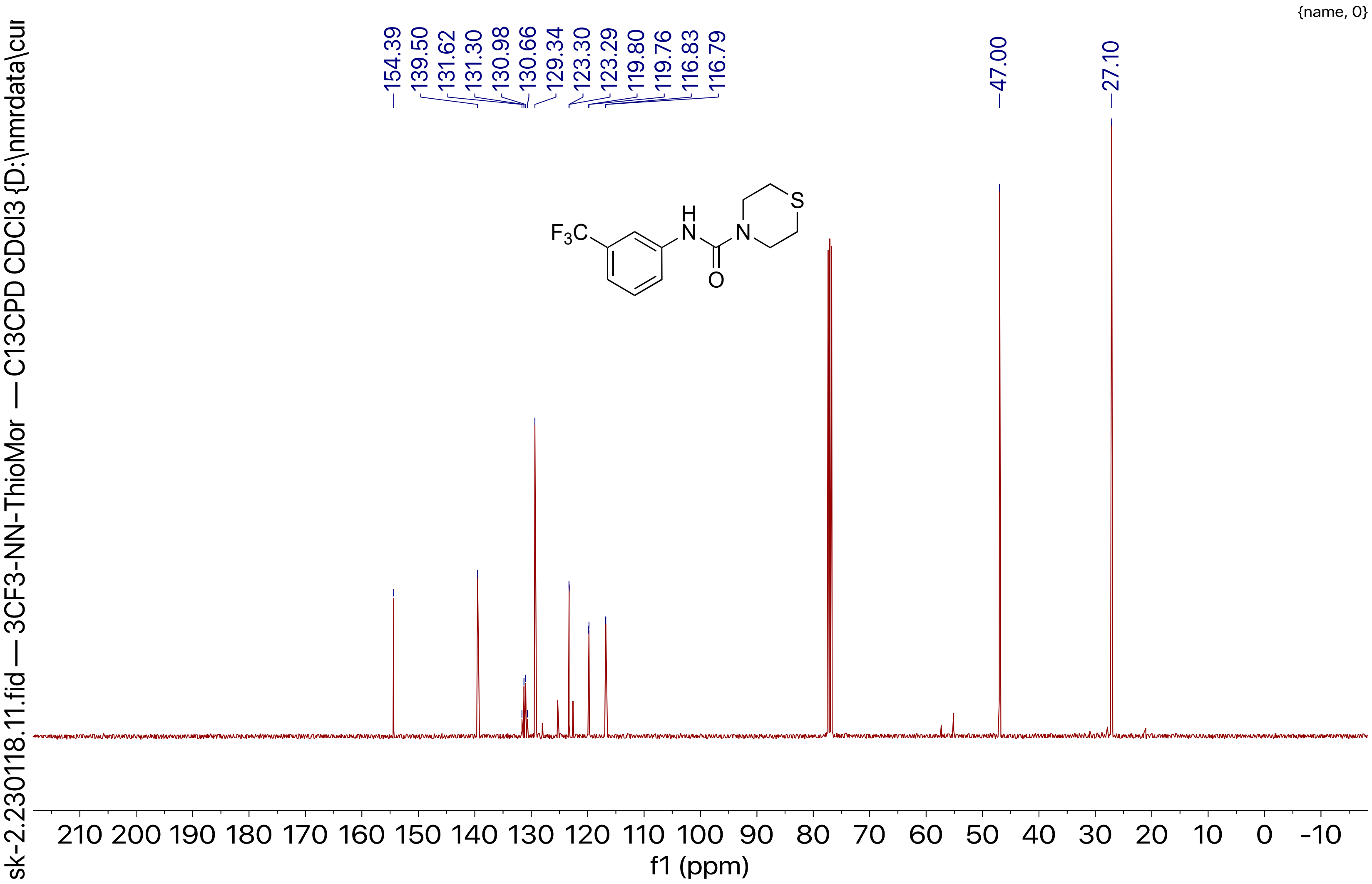

^{13}C NMR spectra of **7p** (101 MHz, RT, $CDCl_3$)

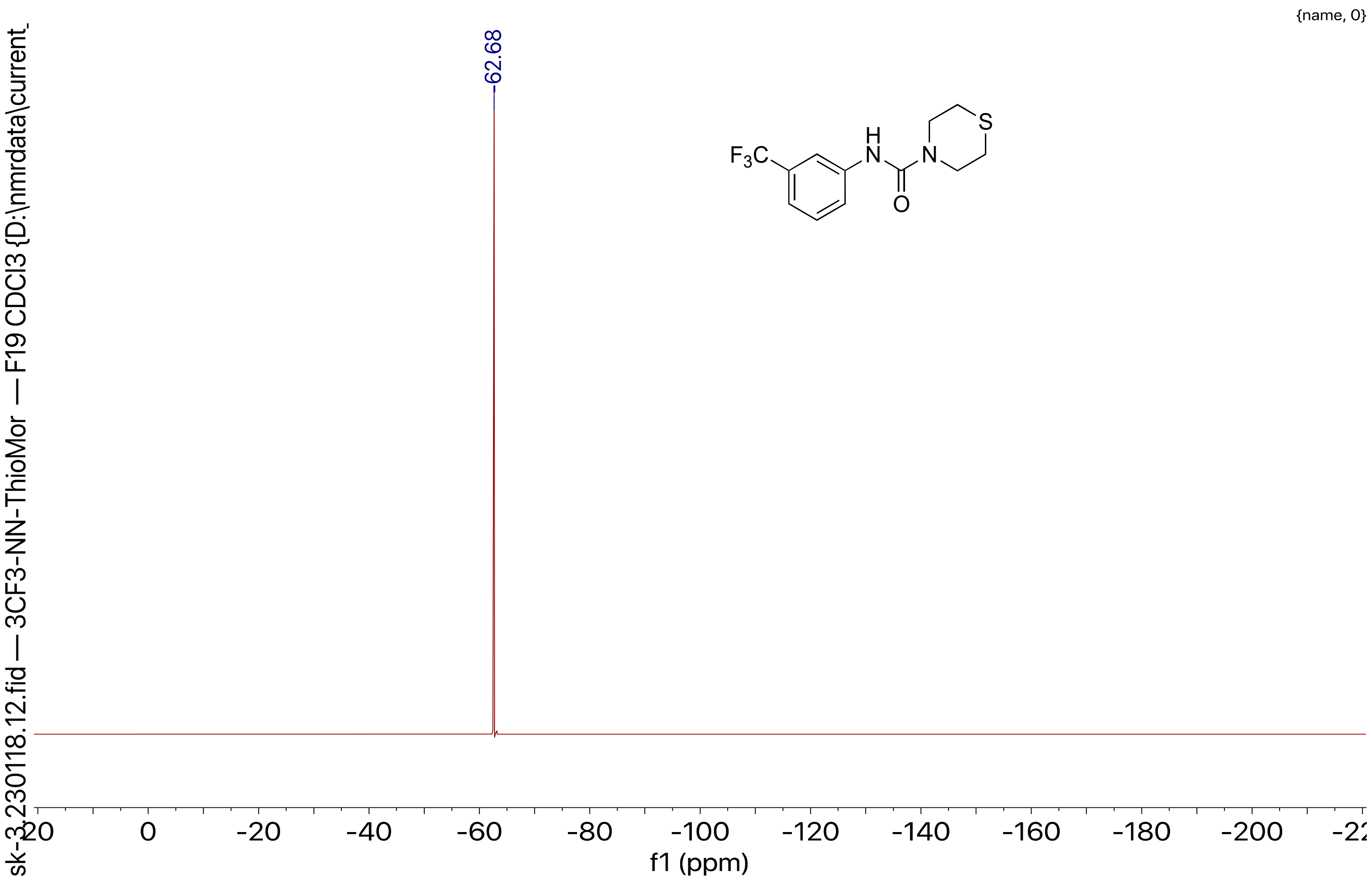

^{19}F NMR spectra of **7p** (376 MHz, RT, $CDCl_3$)

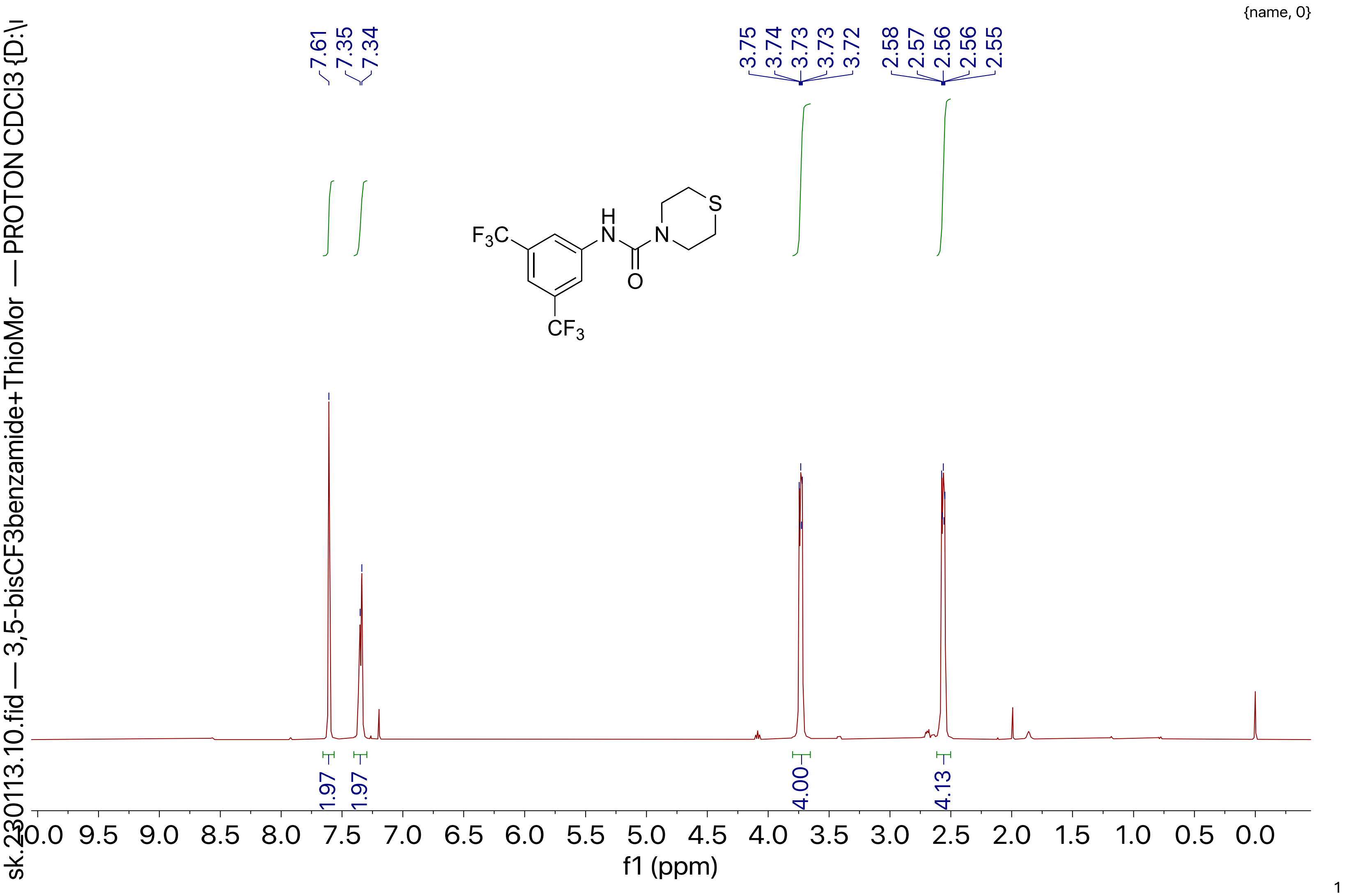

H NMR spectra of **7q** (400 MHz, RT, $CDCl_3$)

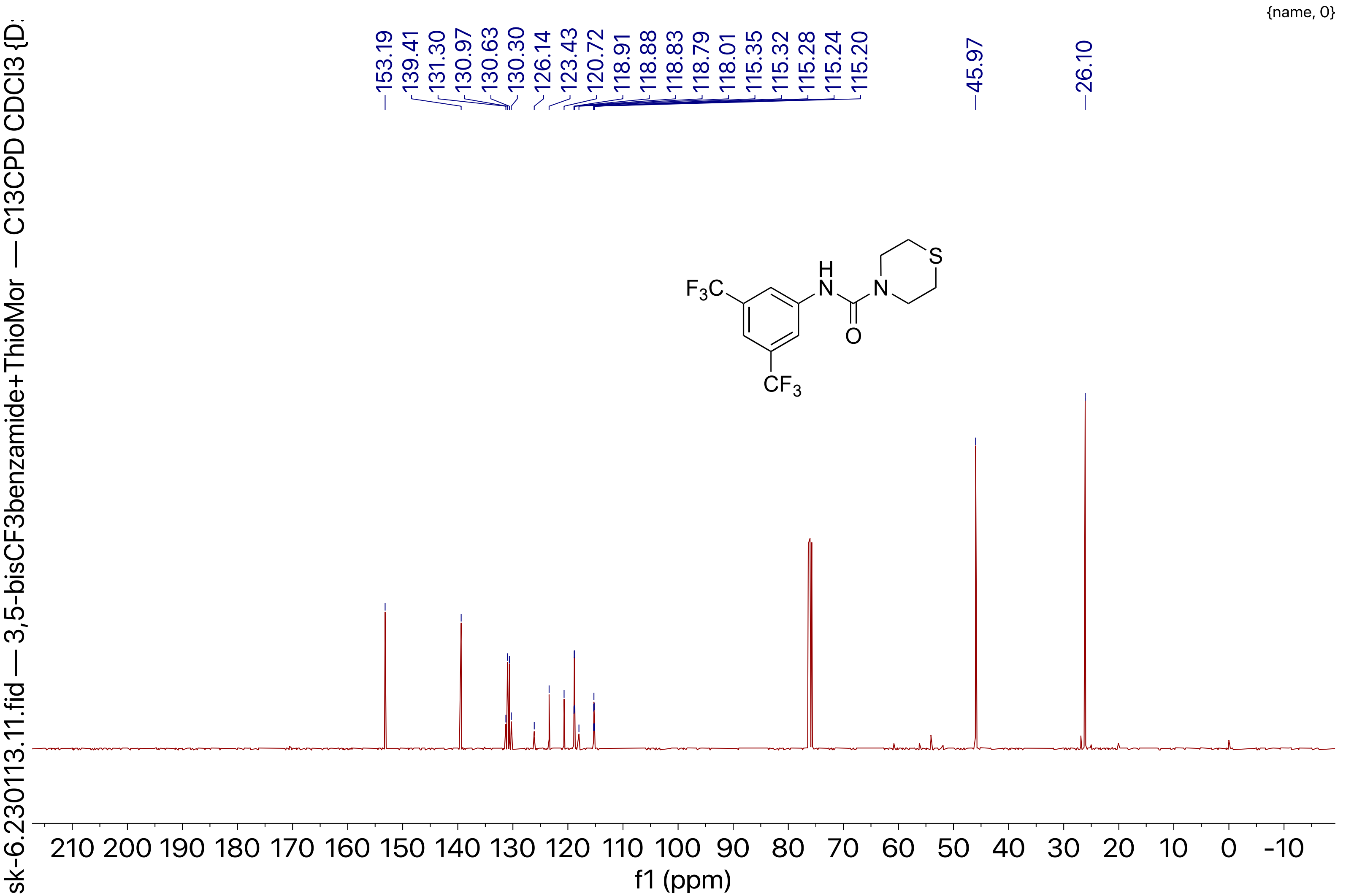

^{13}C NMR spectra of **7q** (101 MHz, RT, $CDCl_3$)

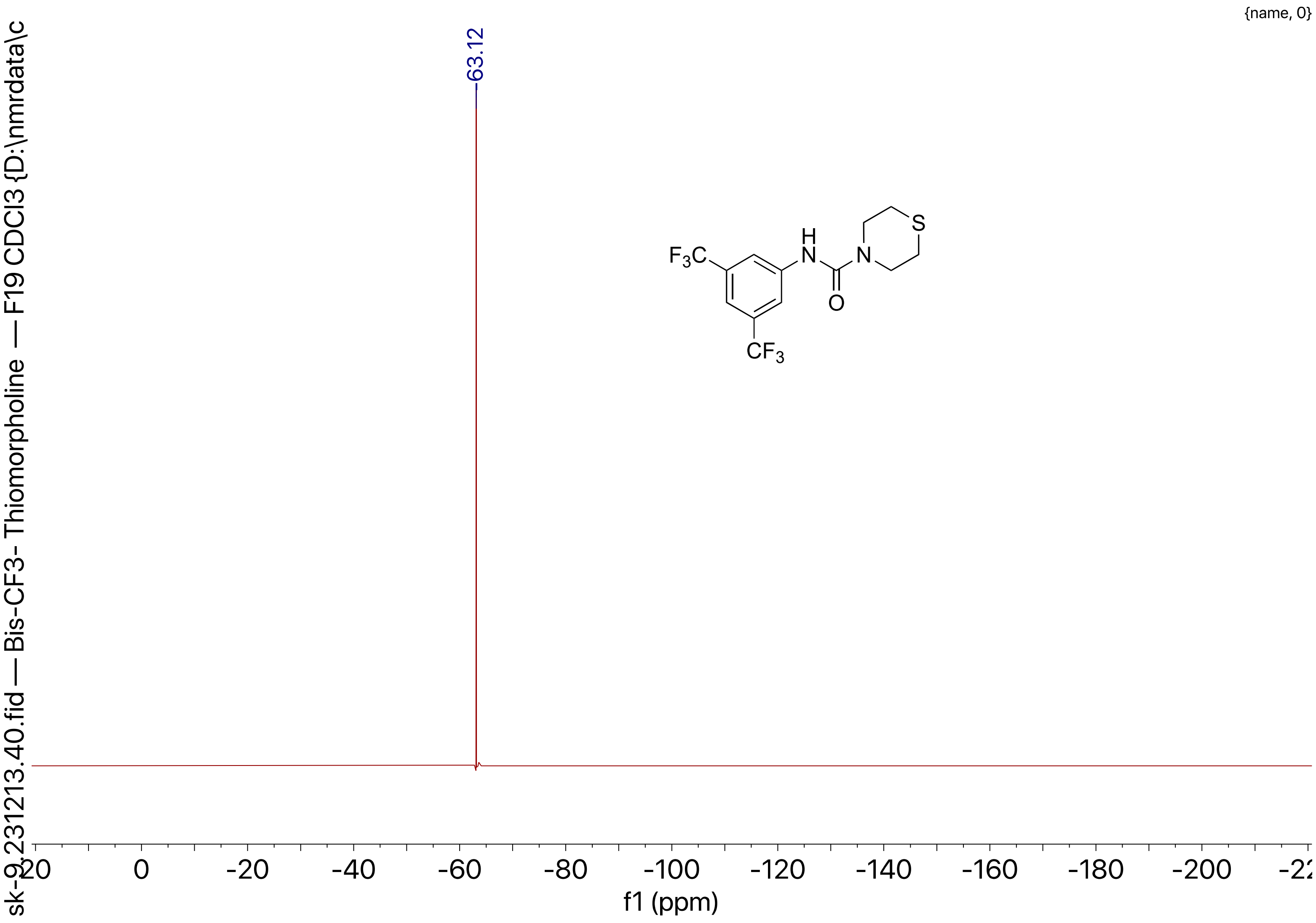

^{19}F NMR spectra of **7q** (376 MHz, RT, $CDCl_3$)

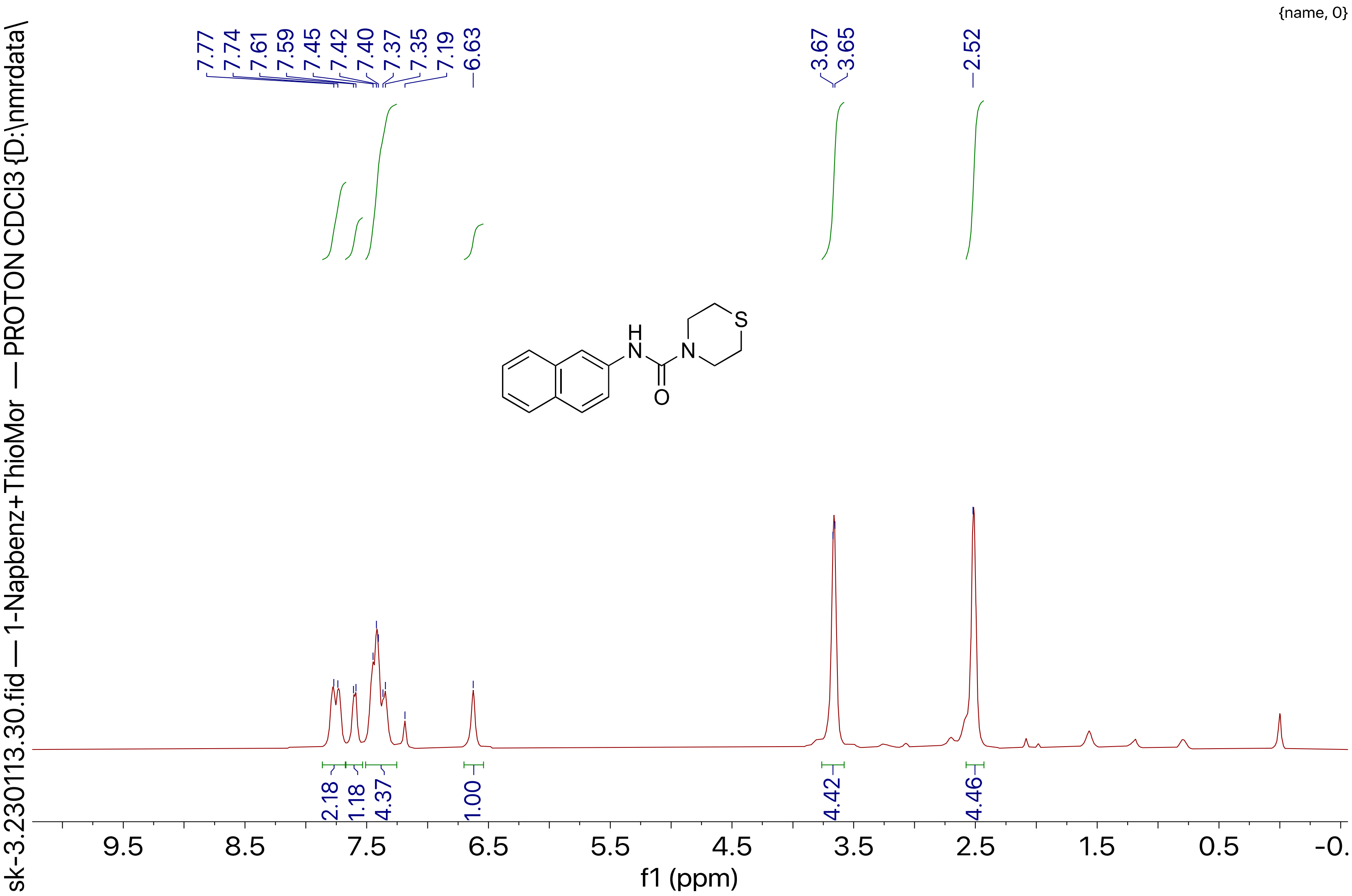

^{1}H NMR spectra of **7r** (400 MHz, RT, $CDCl_3$)

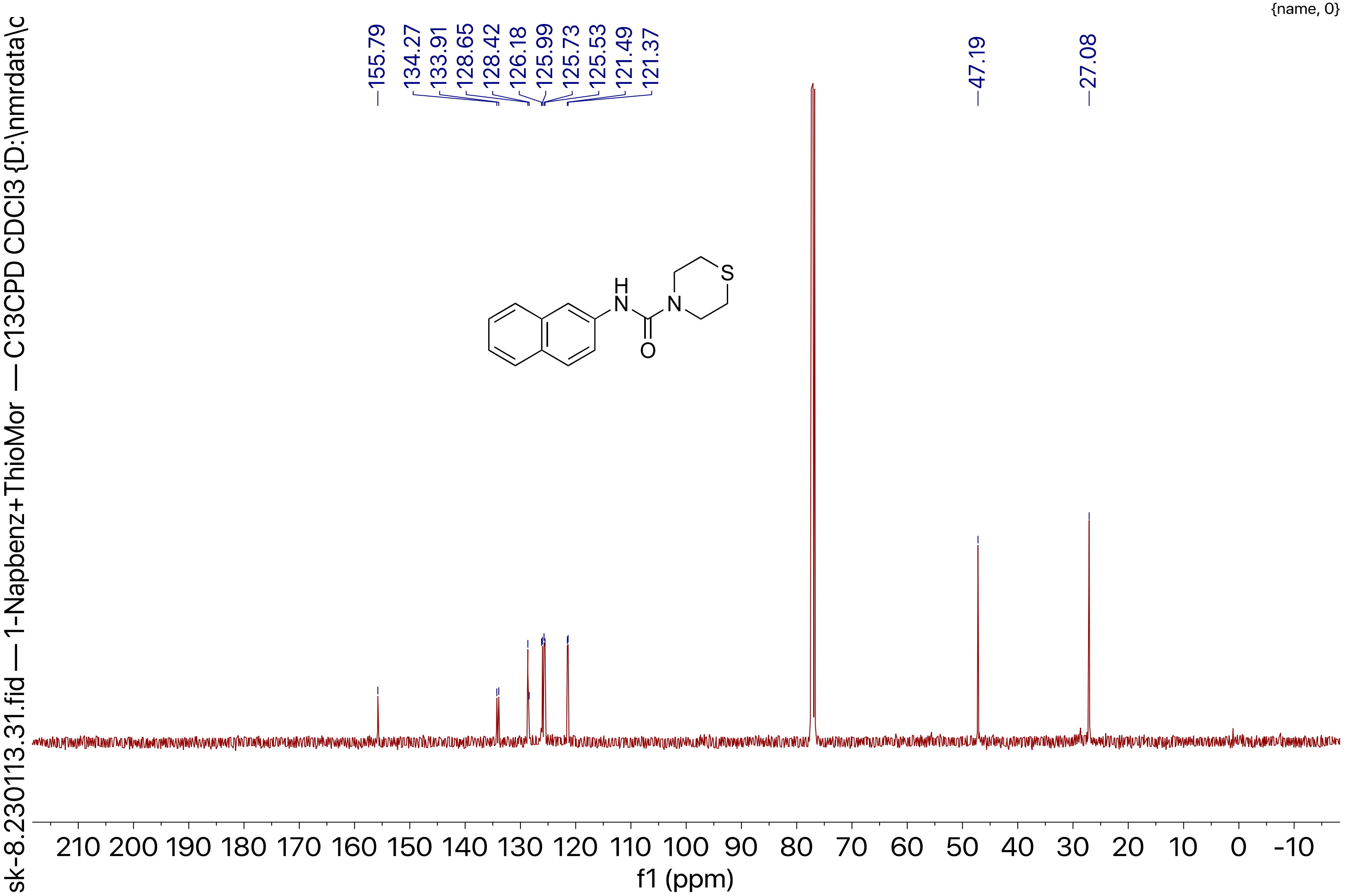

^{13}C NMR spectra of **7r** (101 MHz, RT, $CDCl_3$)

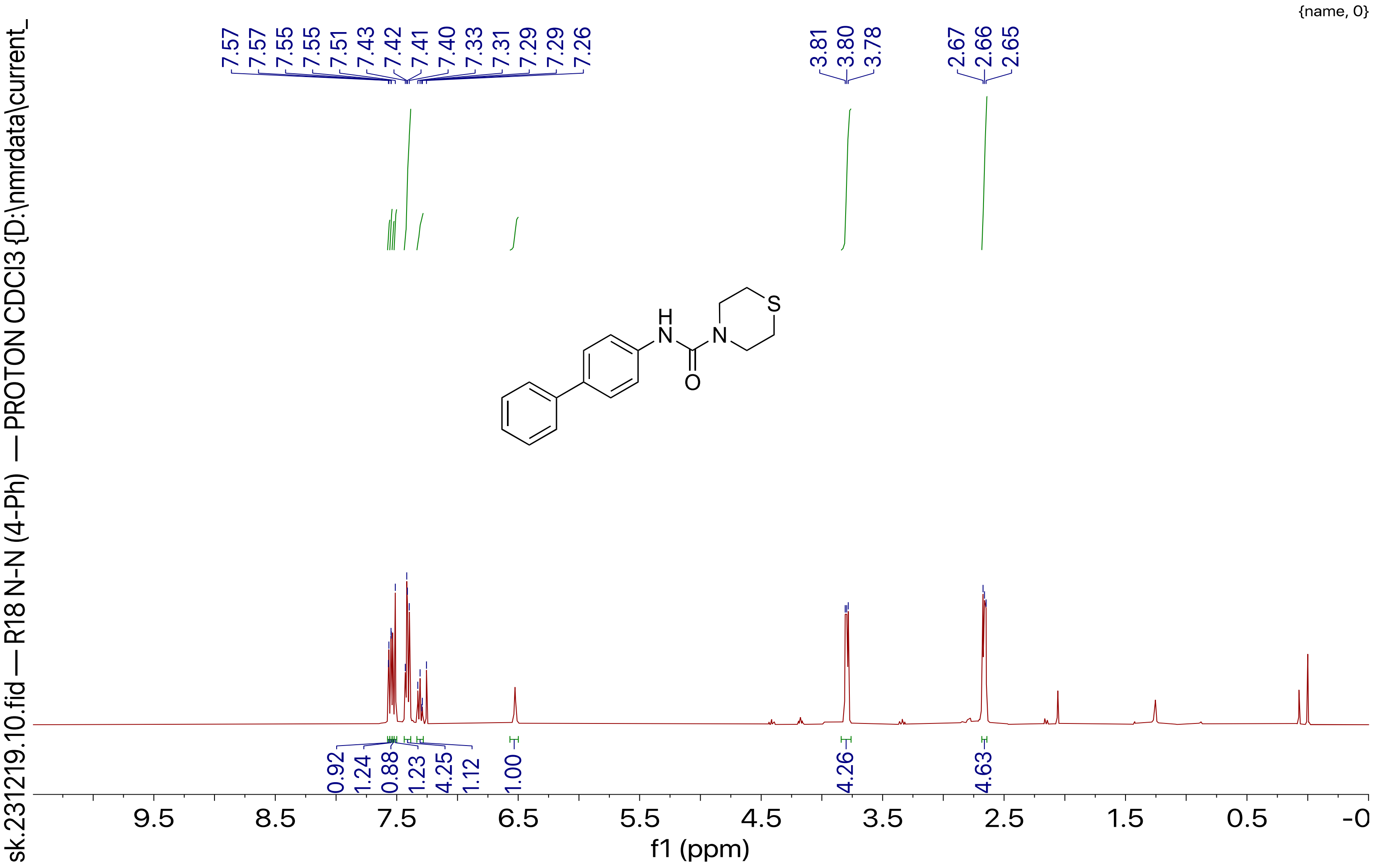

^{1}H NMR spectra of **7s** (400 MHz, RT, $CDCl_3$)

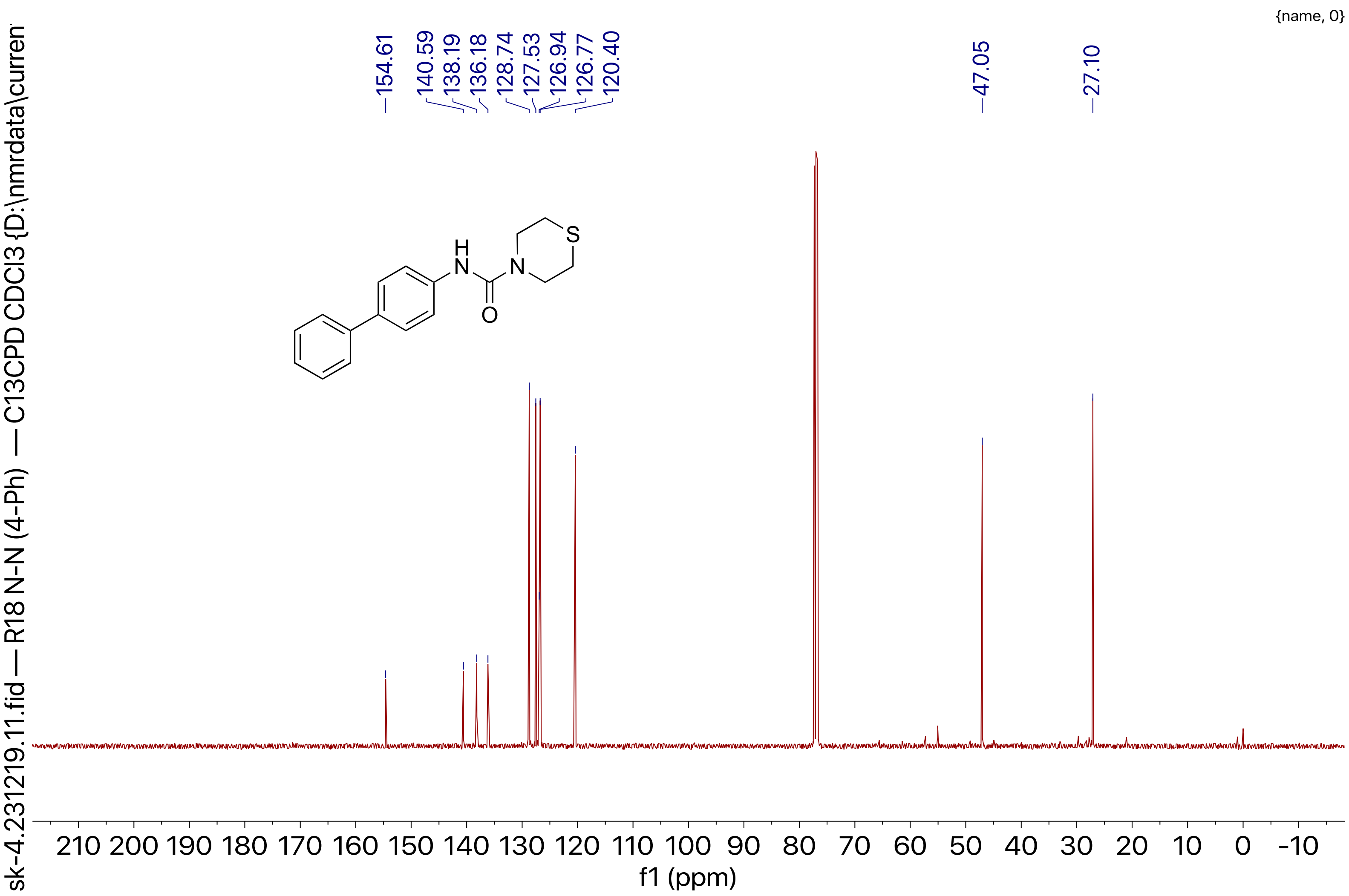

^{13}C NMR spectra of **7s** (101 MHz, RT, $CDCl_3$)

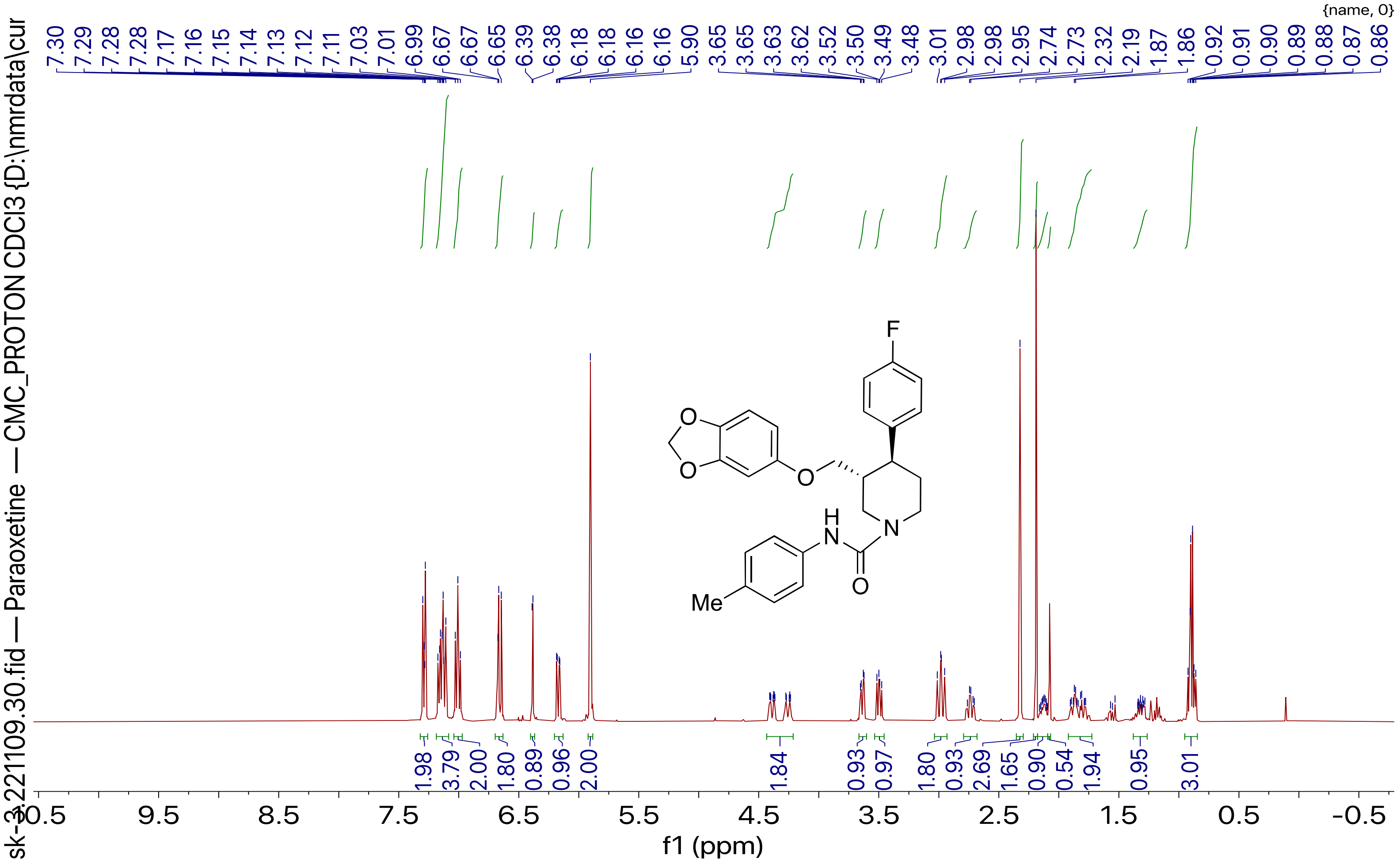

^{1}H NMR spectra of **9a** (400 MHz, RT, $CDCl_3$)

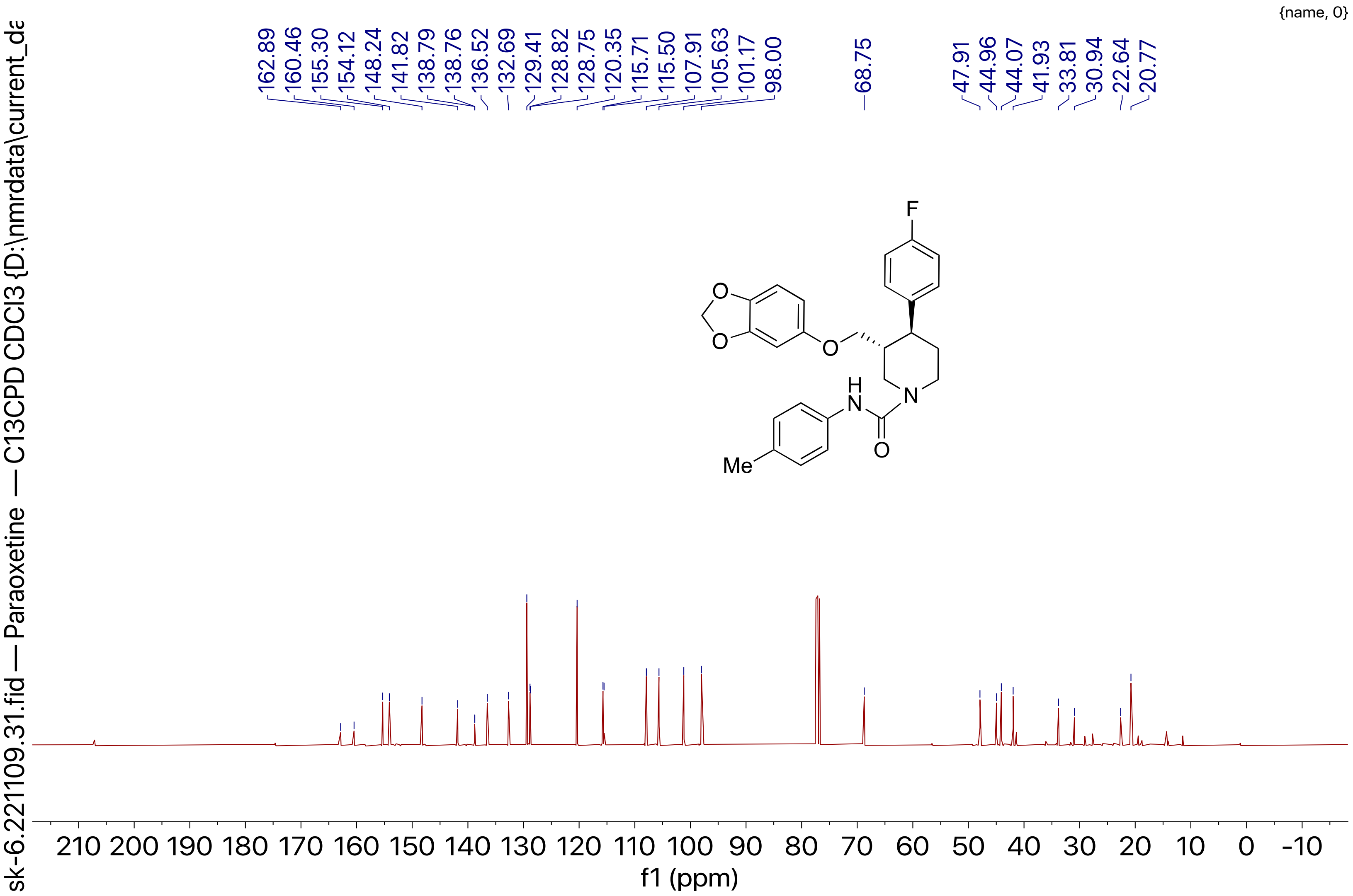

^{13}C NMR spectra of **9a** (101 MHz, RT, $CDCl_3$)

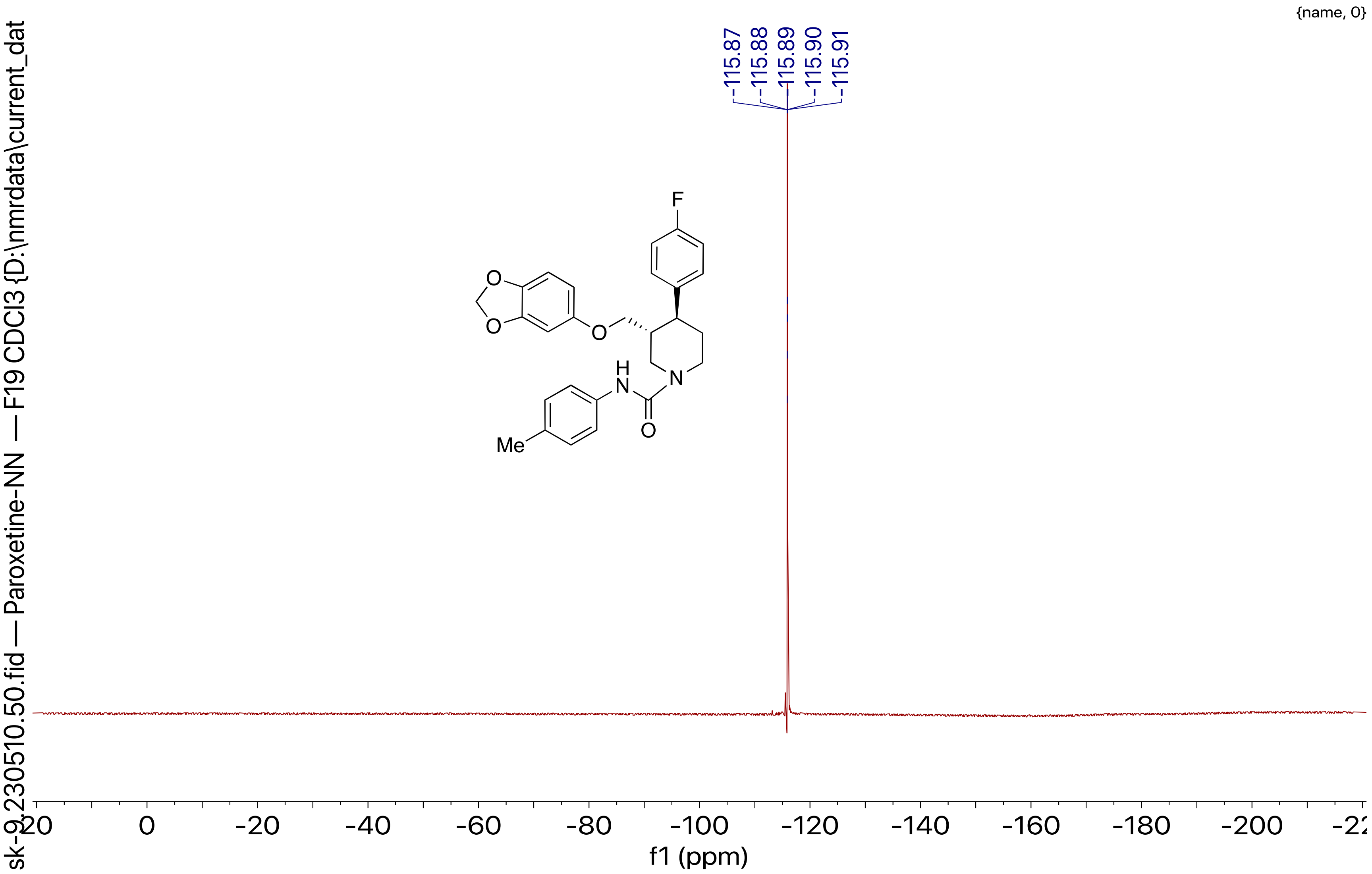

^{19}F NMR spectra of **9a** (376 MHz, RT, $CDCl_3$)

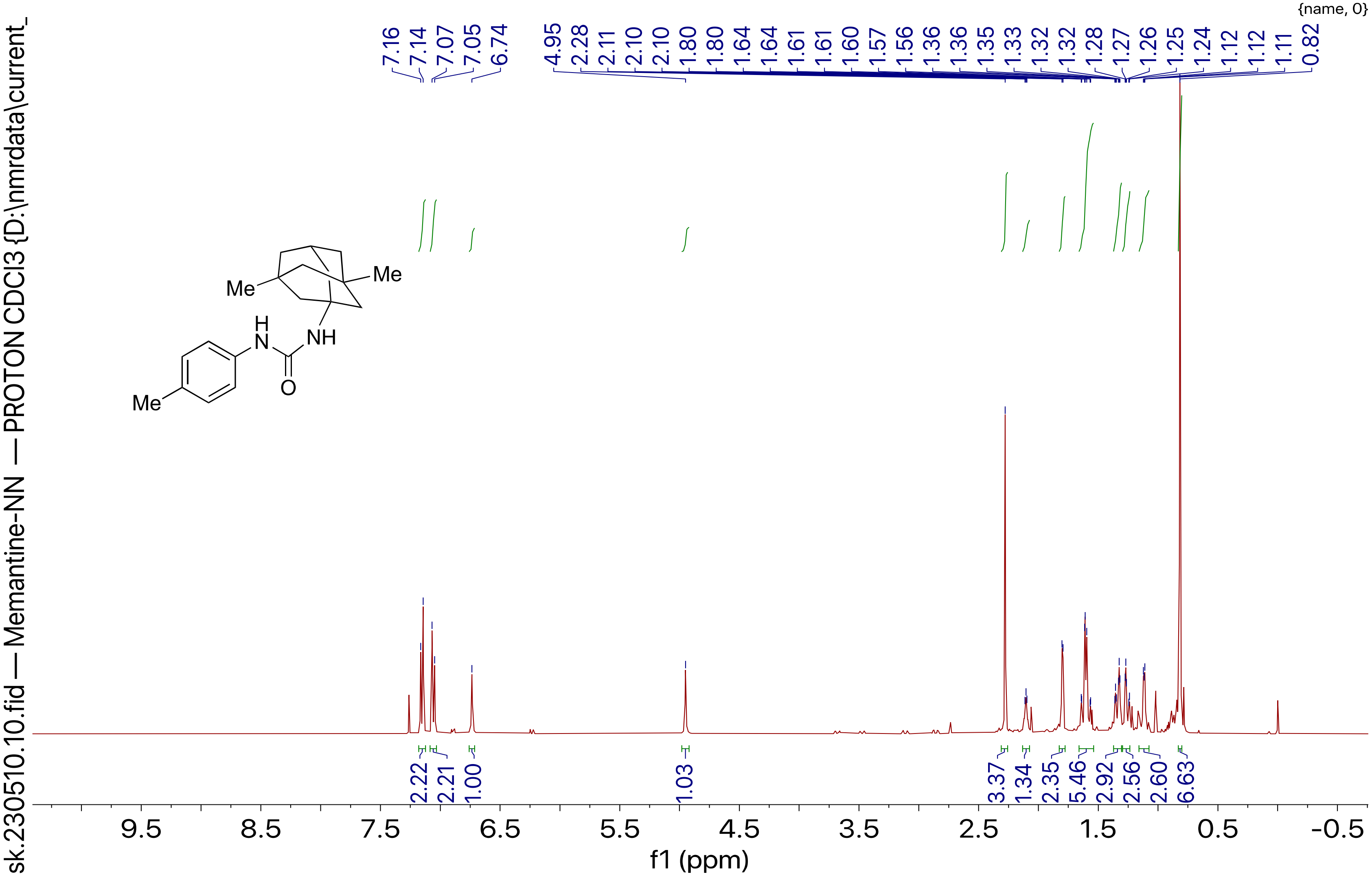

^{1}H NMR spectra of **9b** (400 MHz, RT, $CDCl_3$)

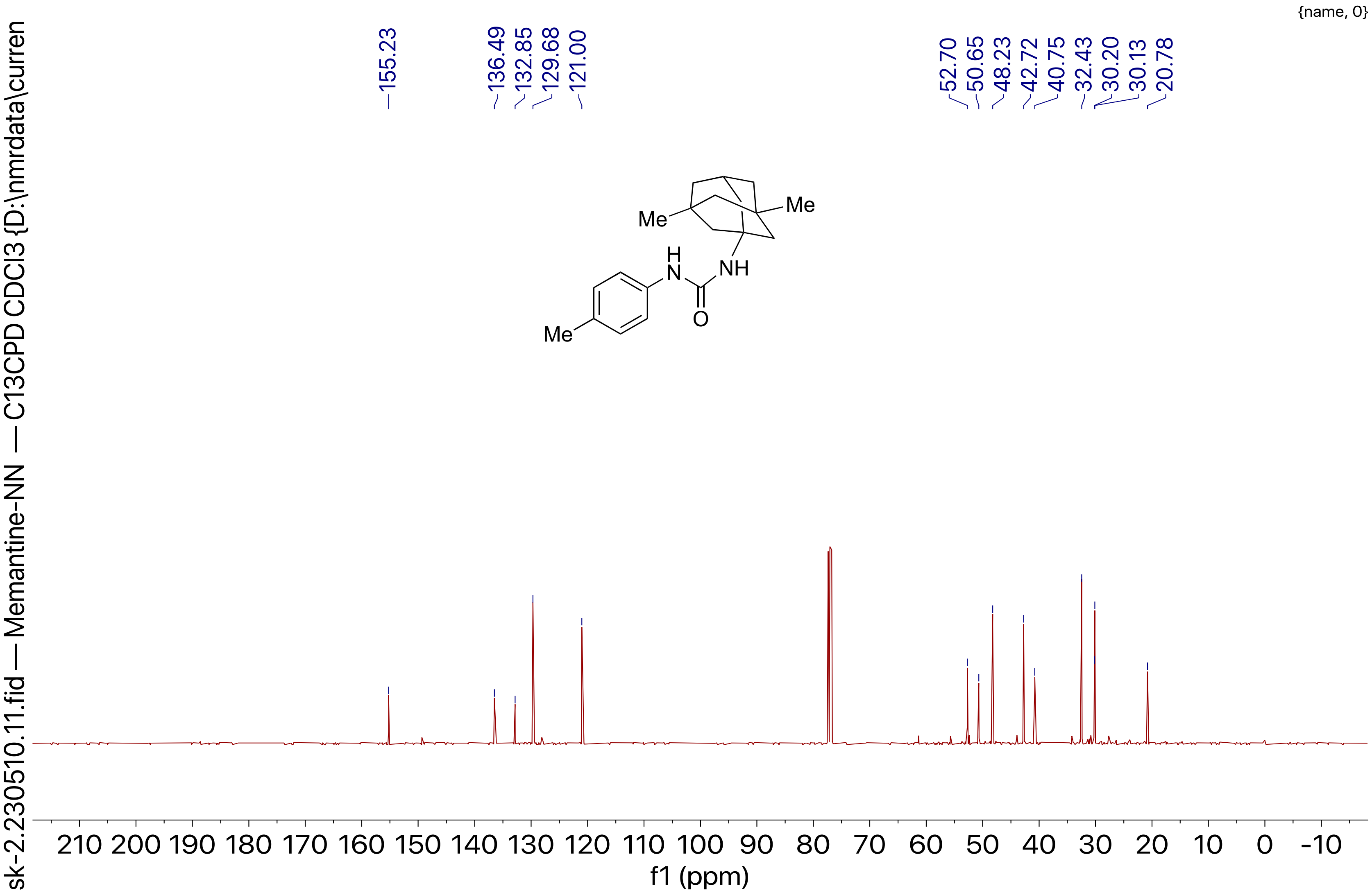
sk-2.230510.11.fid — Memantine-NN — C13CPD CDCl3 {D:\nmrdata\curren
{name, 0}
155.23
136.49
132.85
129.68
121.00
52.70
50.65
48.23
42.72
40.75
32.43
30.20
30.13
20.78
Me
Me
H
N
NH
O
Me
210 200 190 180 170 160 150 140 130 120 110 100 90 80 70 60 50 40 30 20 10 0 -10
f1 (ppm)

^{13}C NMR spectra of **9b** (101 MHz, RT, $CDCl_3$)

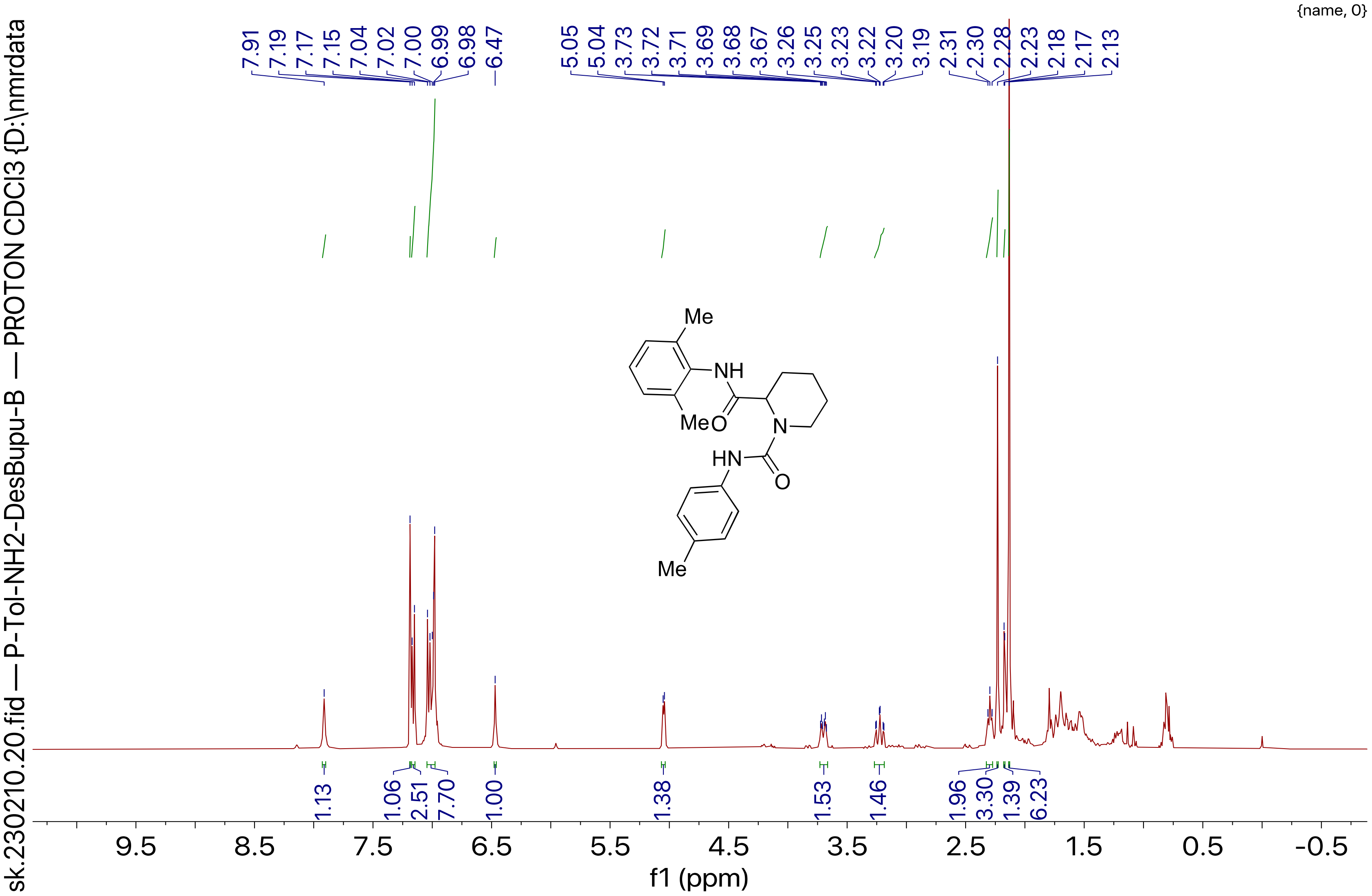

^{1}H NMR spectra of **9c** (400 MHz, RT, $CDCl_3$)

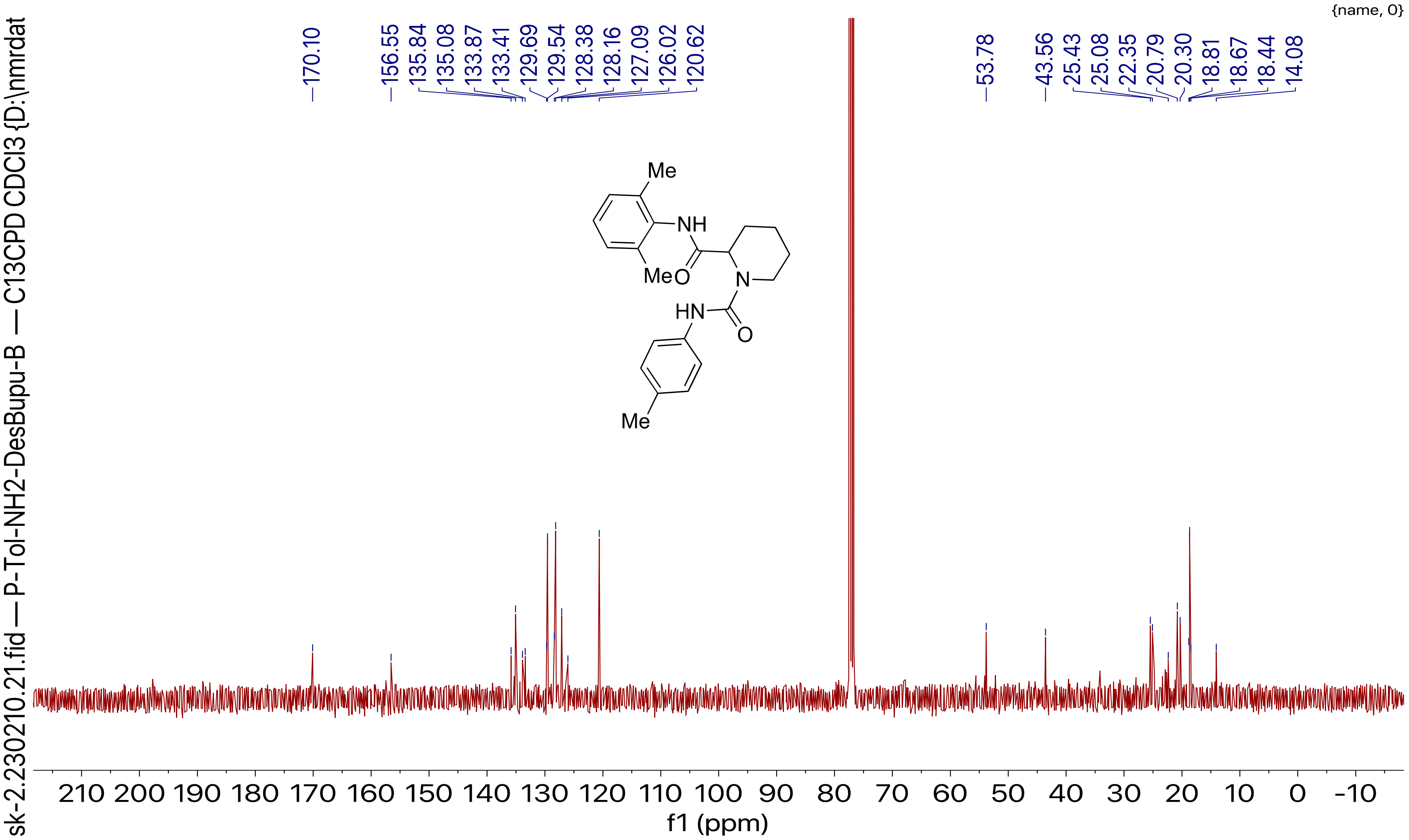

^{13}C NMR spectra of **9c** (101 MHz, RT, $CDCl_3$)

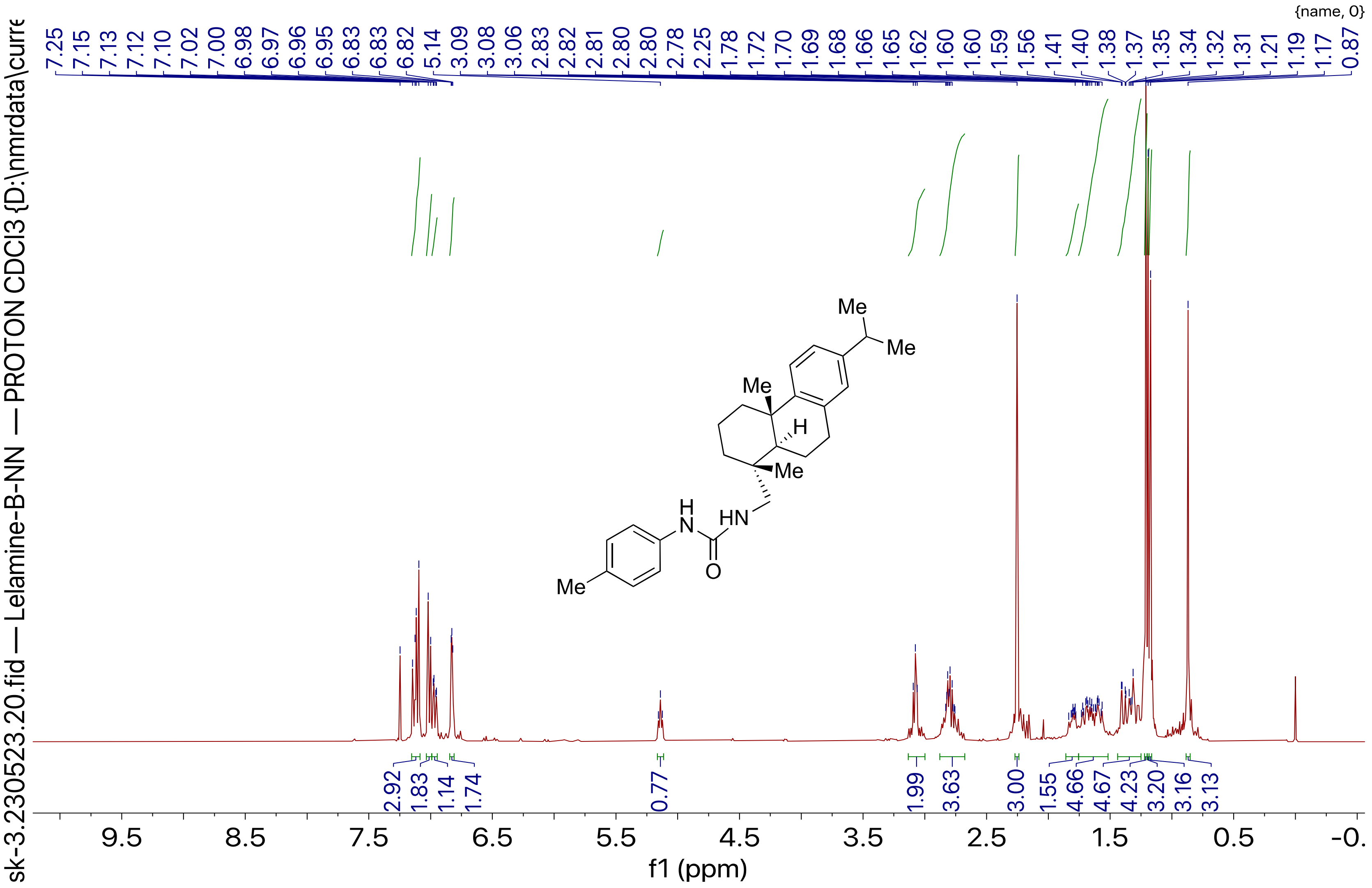

^{1}H NMR spectra of **9d** (400 MHz, RT, $CDCl_3$)

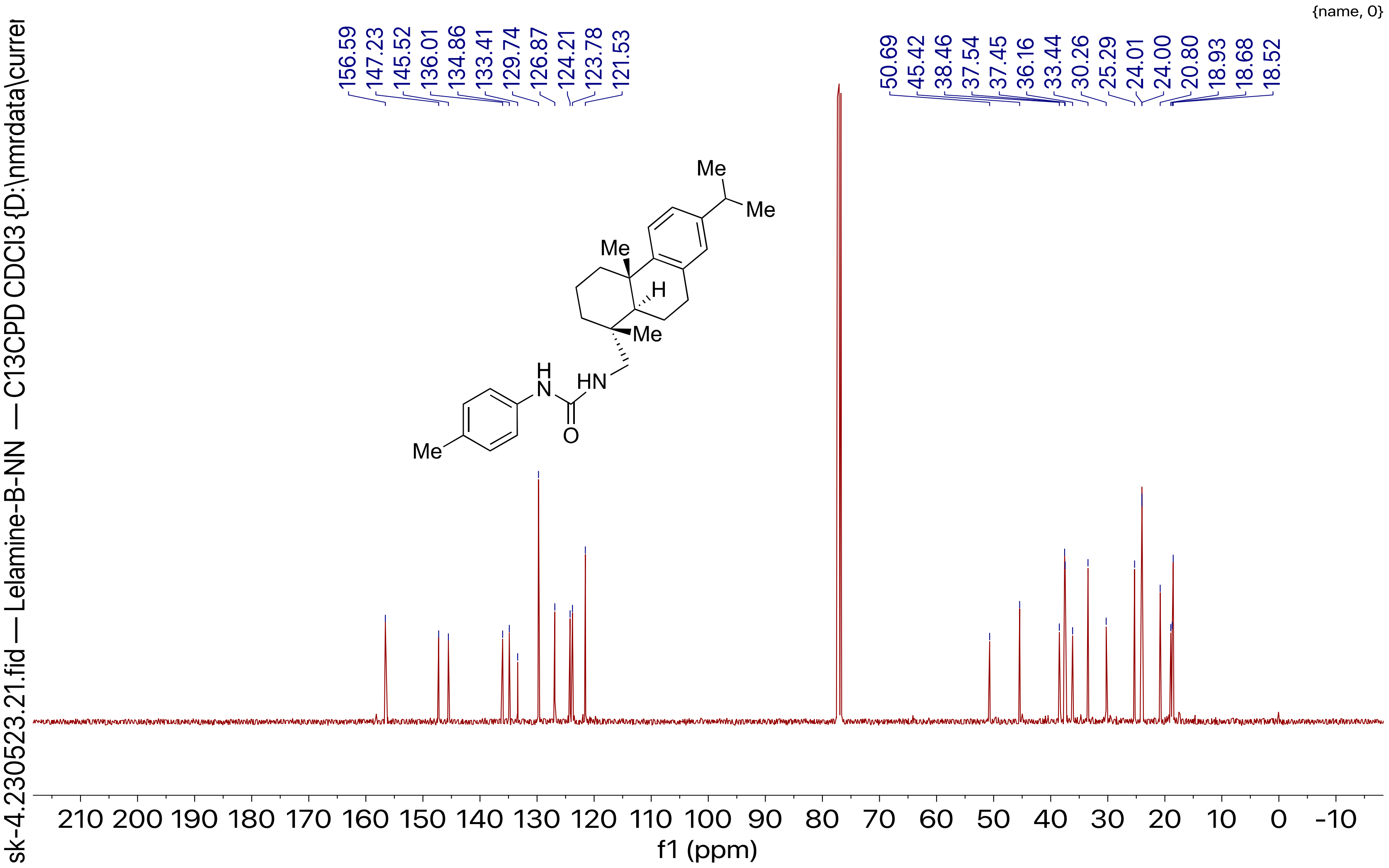

^{13}C NMR spectra of **9d** (101 MHz, RT, $CDCl_3$)

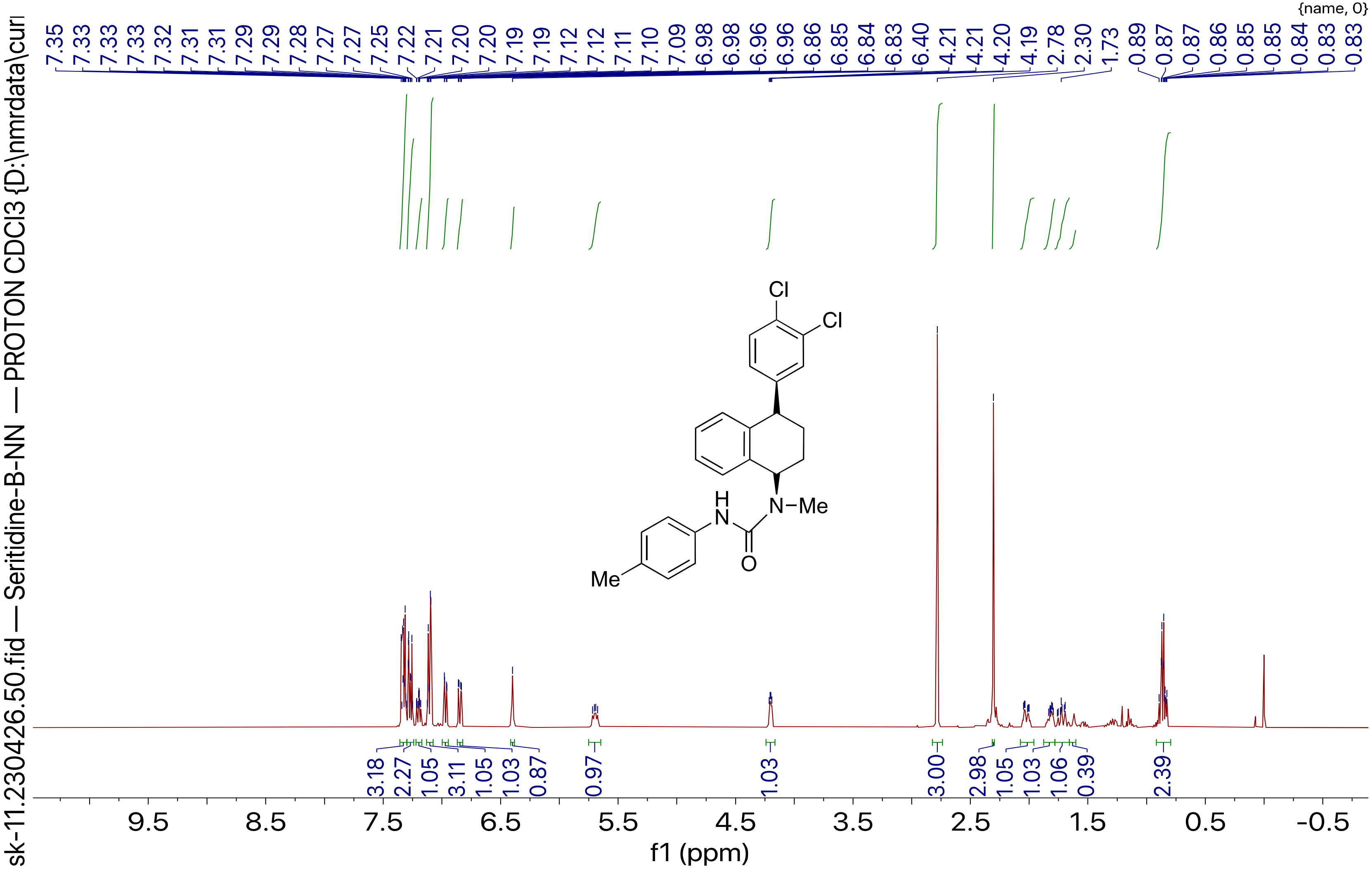

^{1}H NMR spectra of **9e** (400 MHz, RT, $CDCl_3$)

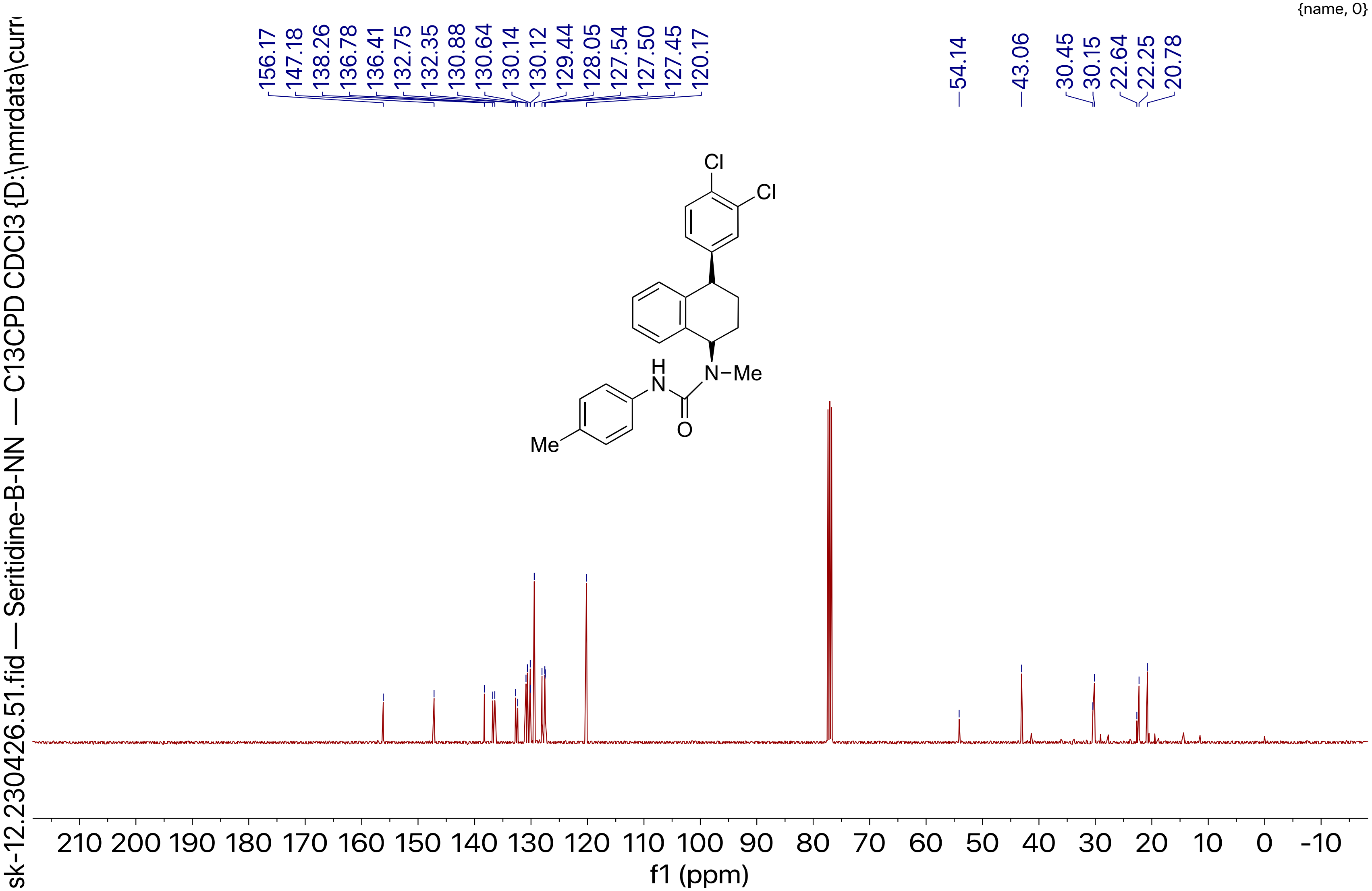

^{13}C NMR spectra of **9e** (101 MHz, RT, $CDCl_3$)

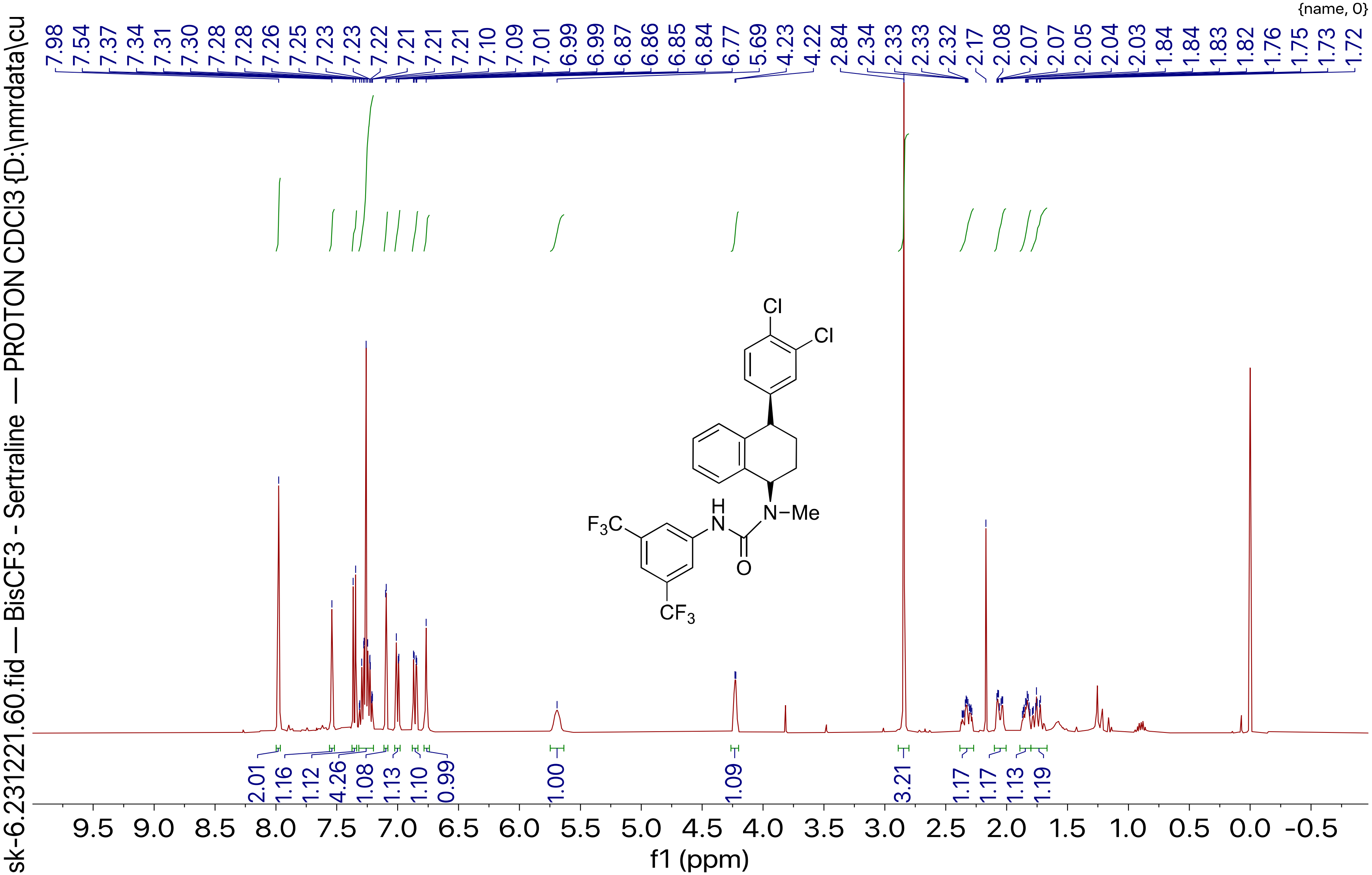

^{1}H NMR spectra of **9e’** (400 MHz, RT, $CDCl_3$)

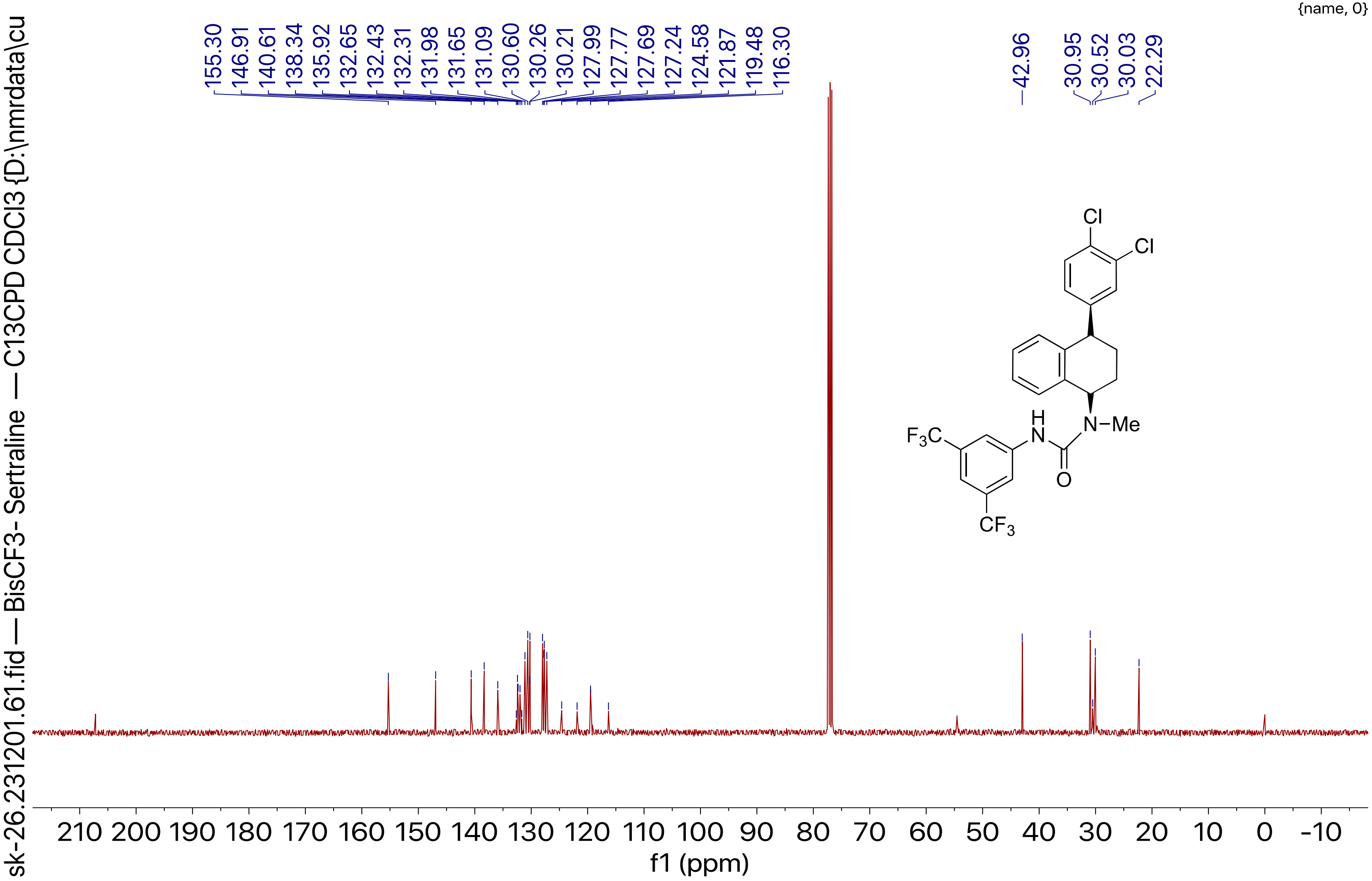

^{13}C NMR spectra of **9e’** (400 MHz, $CDCl_3$ RT)

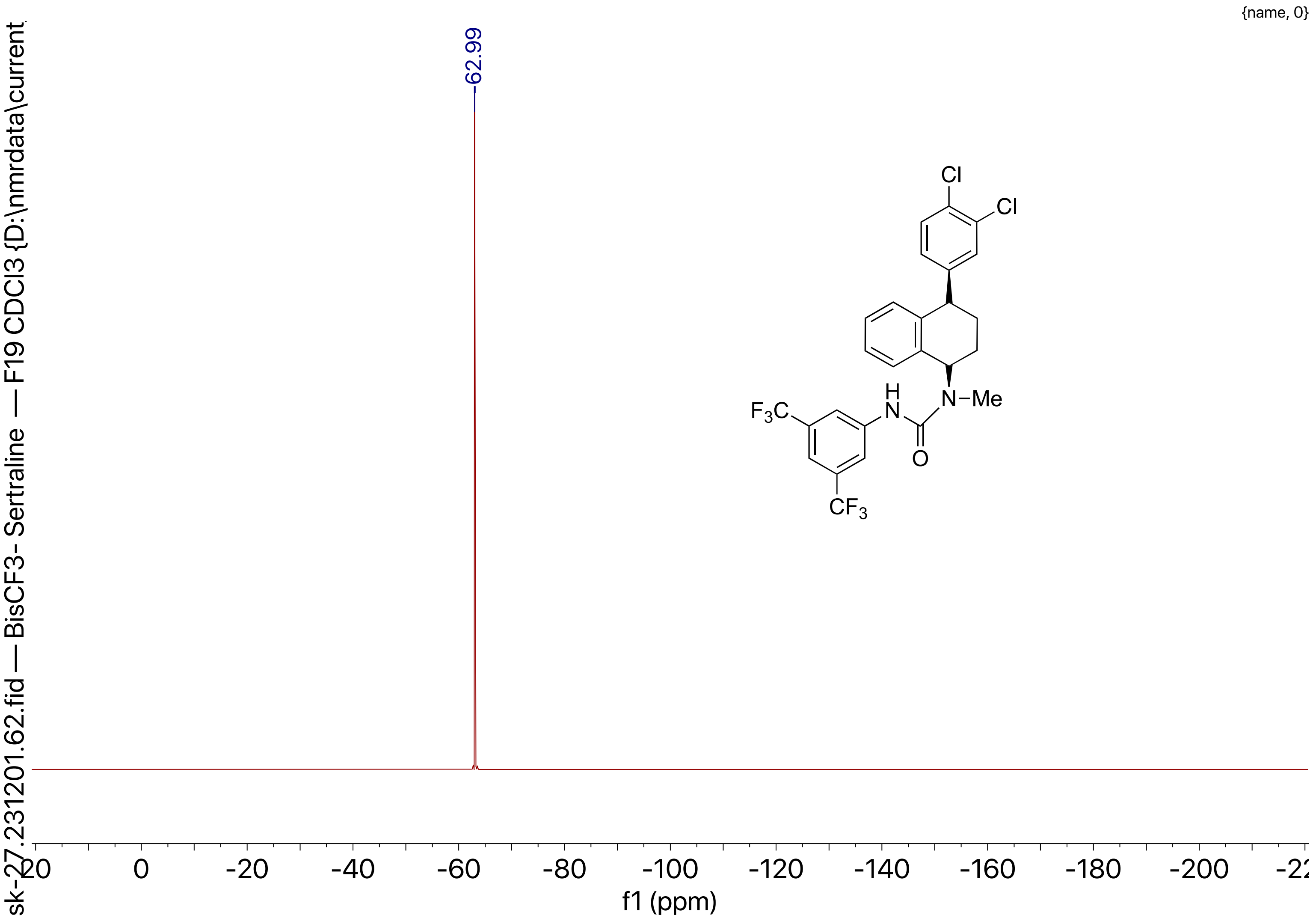

^{19}F NMR spectra of **9e’** (376 MHz, $CDCl_3$ RT)

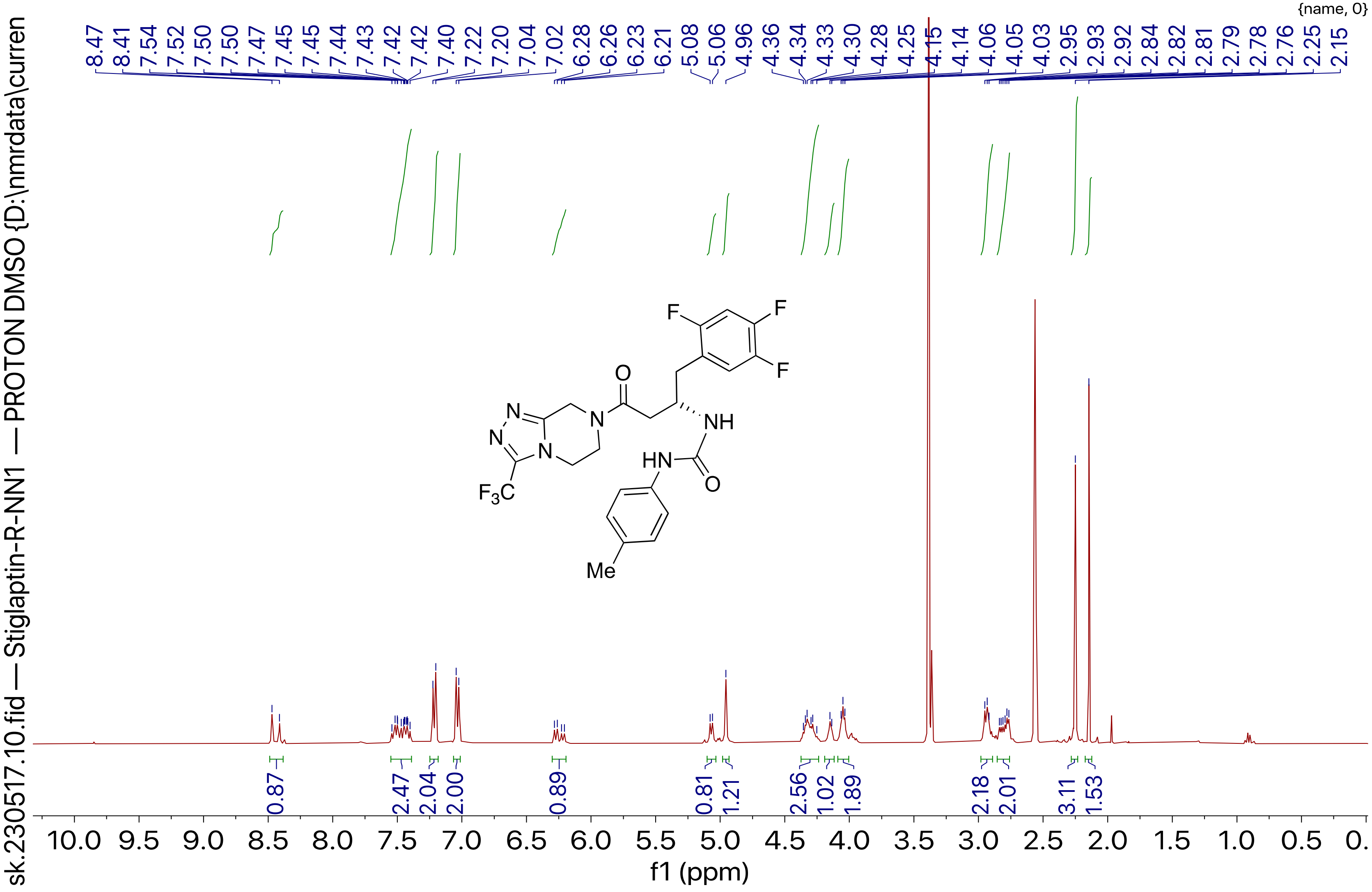

^{1}H NMR spectra of **9f** (101 MHz, RT, DMSO-d_6)

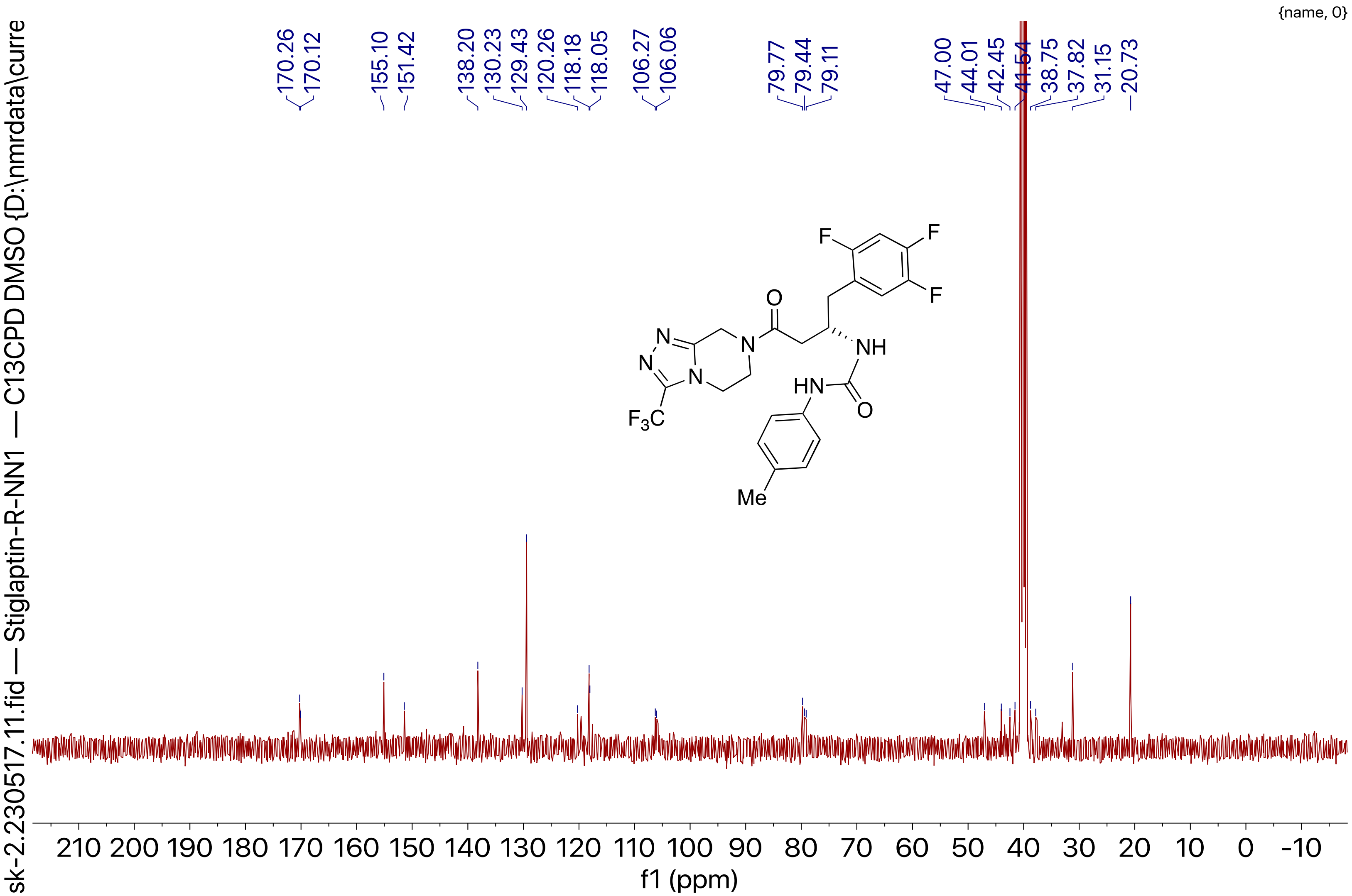

^{13}C NMR spectra of **9f** (101 MHz, RT, DMSO-d_6)

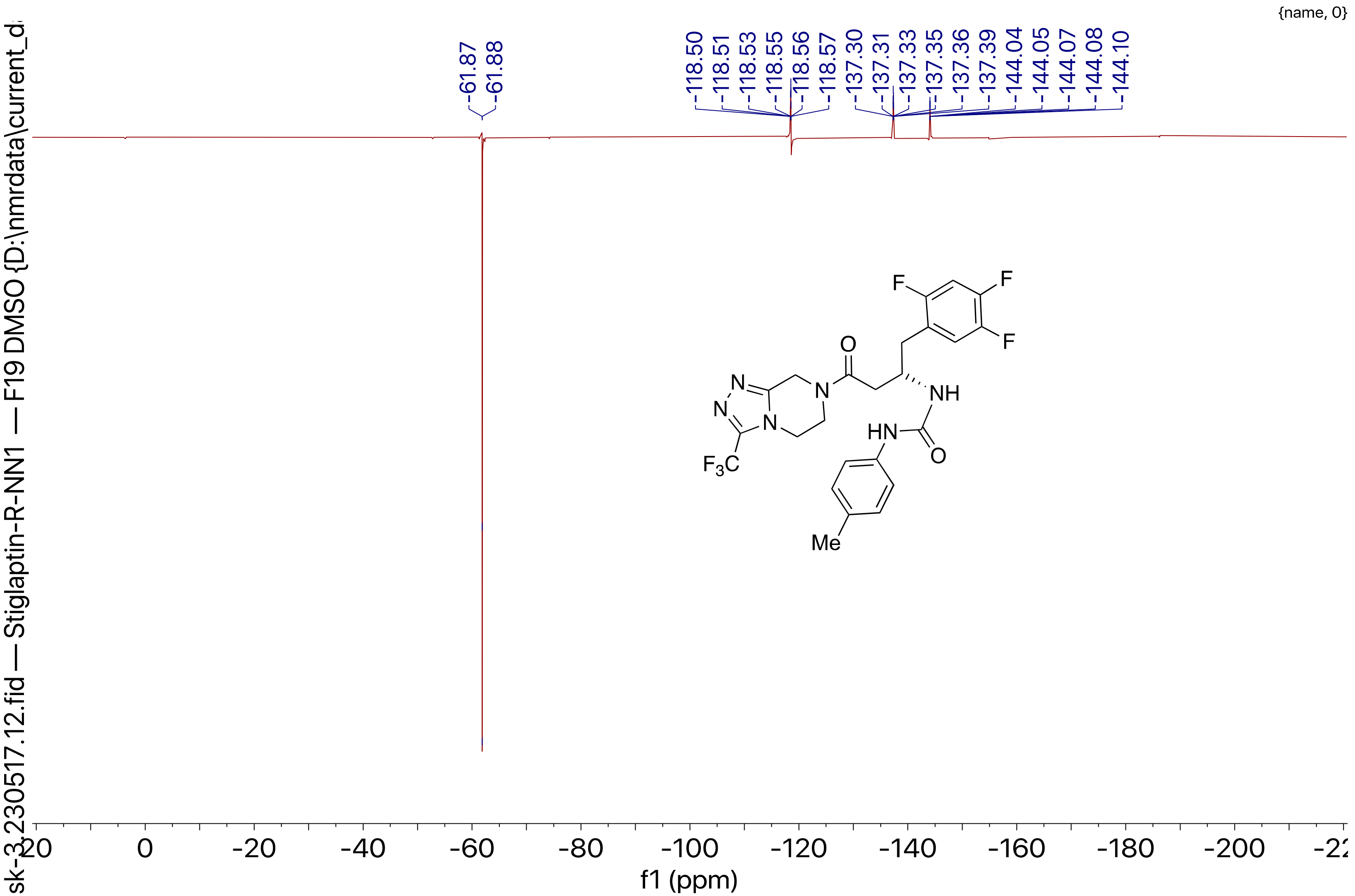

^{19}F NMR spectra of **9f** (376 MHz, RT, DMSO-d_6)

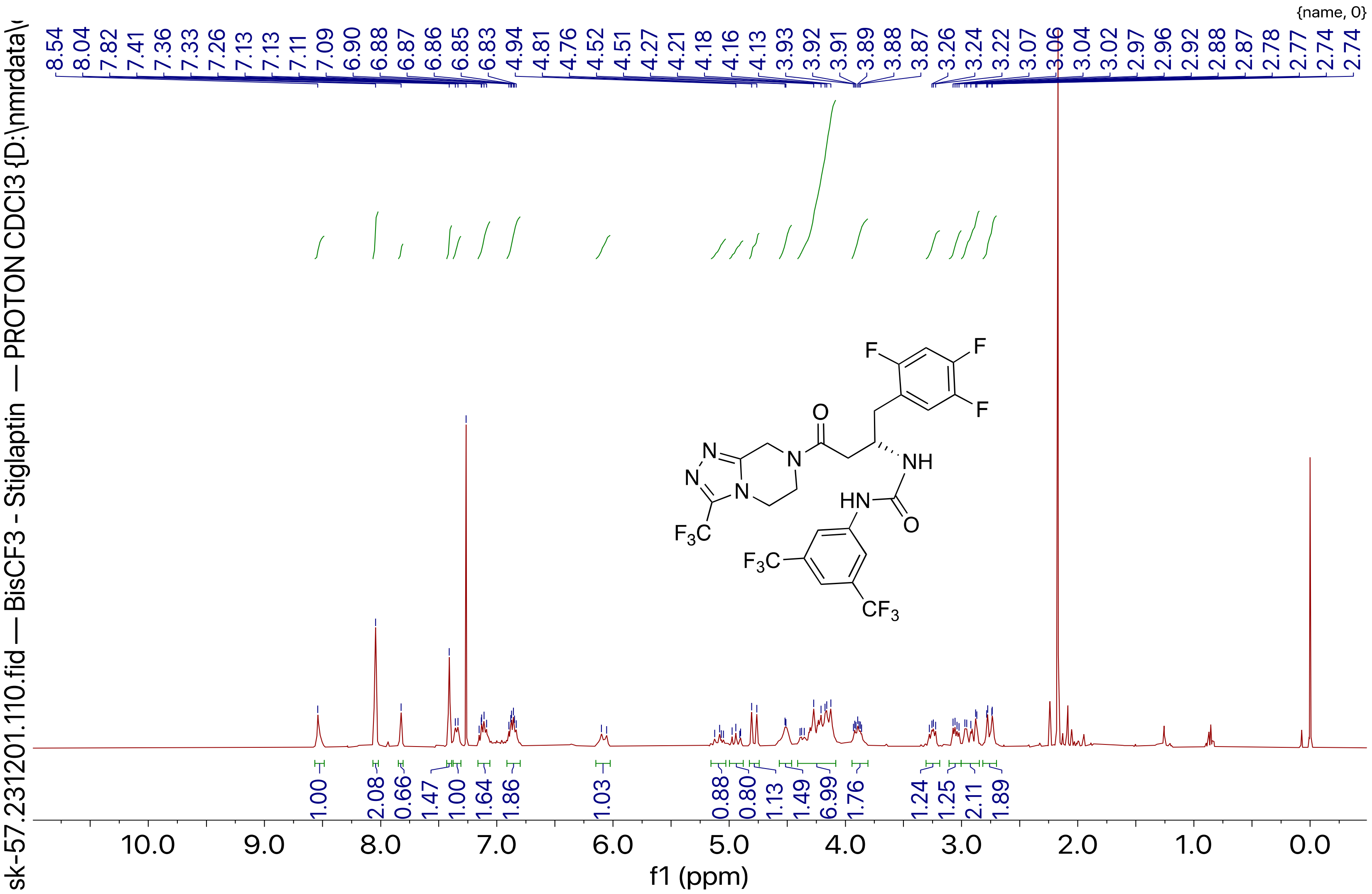

^{1}H NMR spectra of **9f'** (376 MHz, RT, CDCl$_3$)

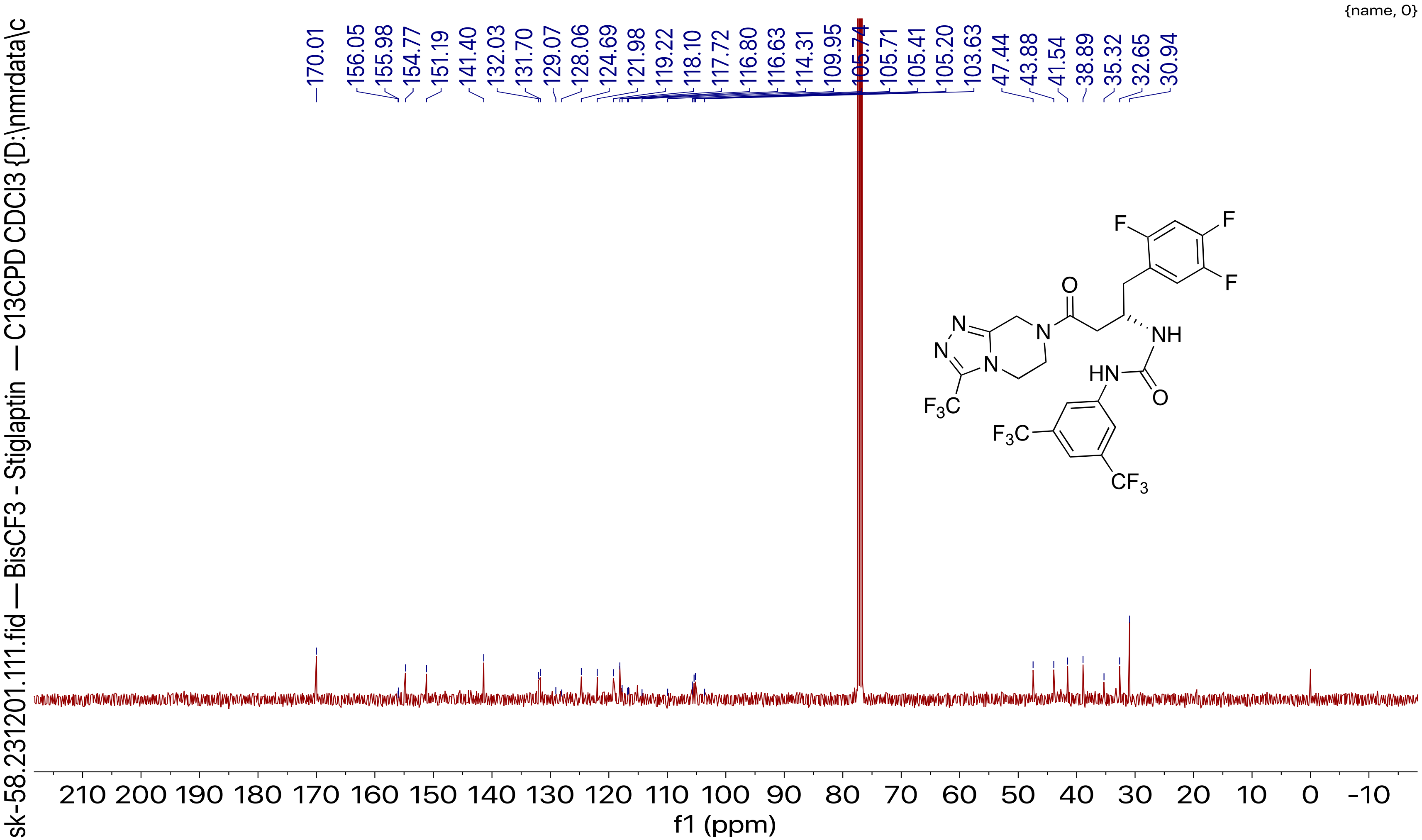

^{13}C NMR spectra of **9f'** (101 MHz, $CDCl_3$, RT)

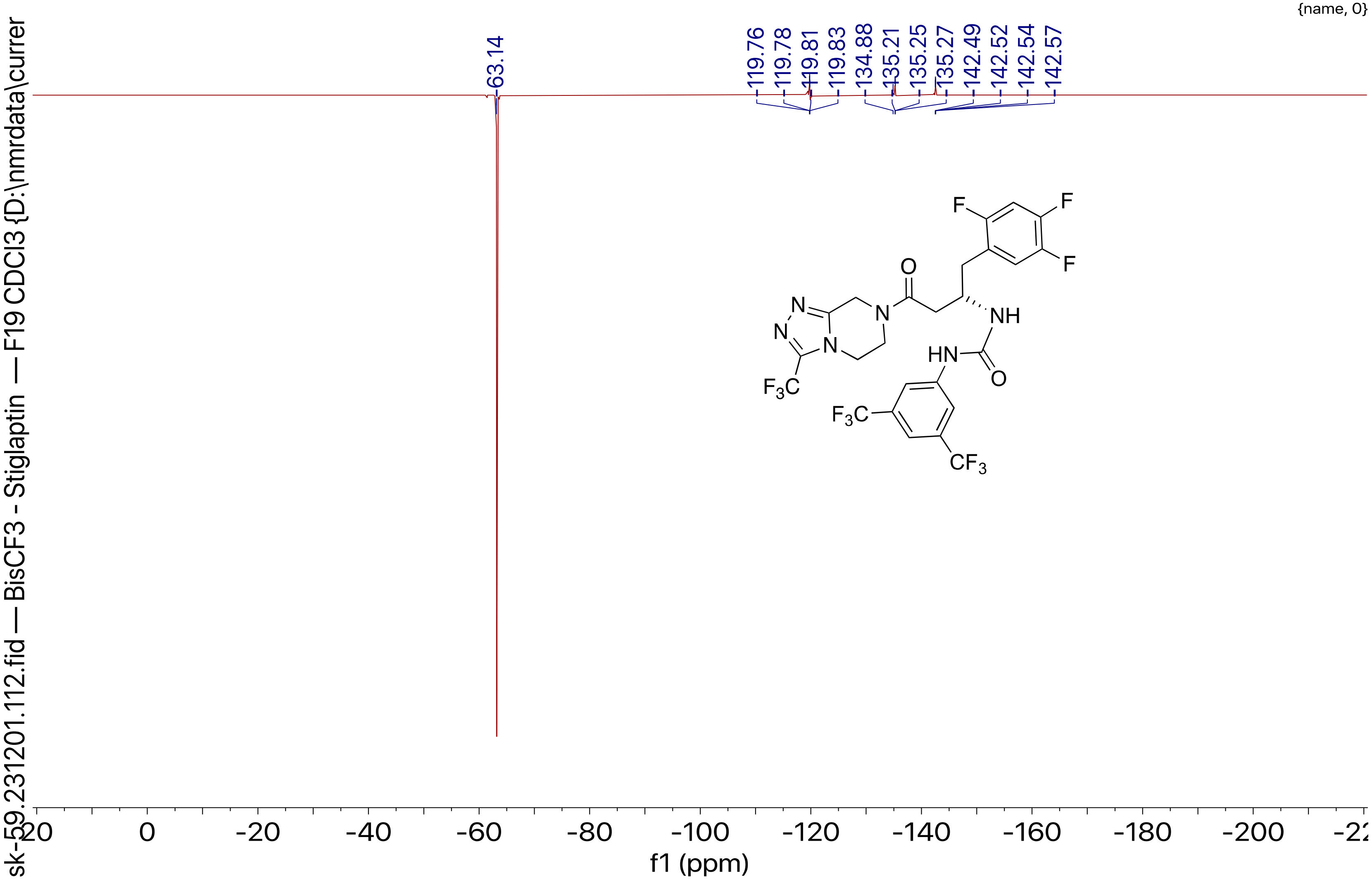

^{19}F NMR spectra of **9f'** (376 MHz, $CDCl_3$, RT)

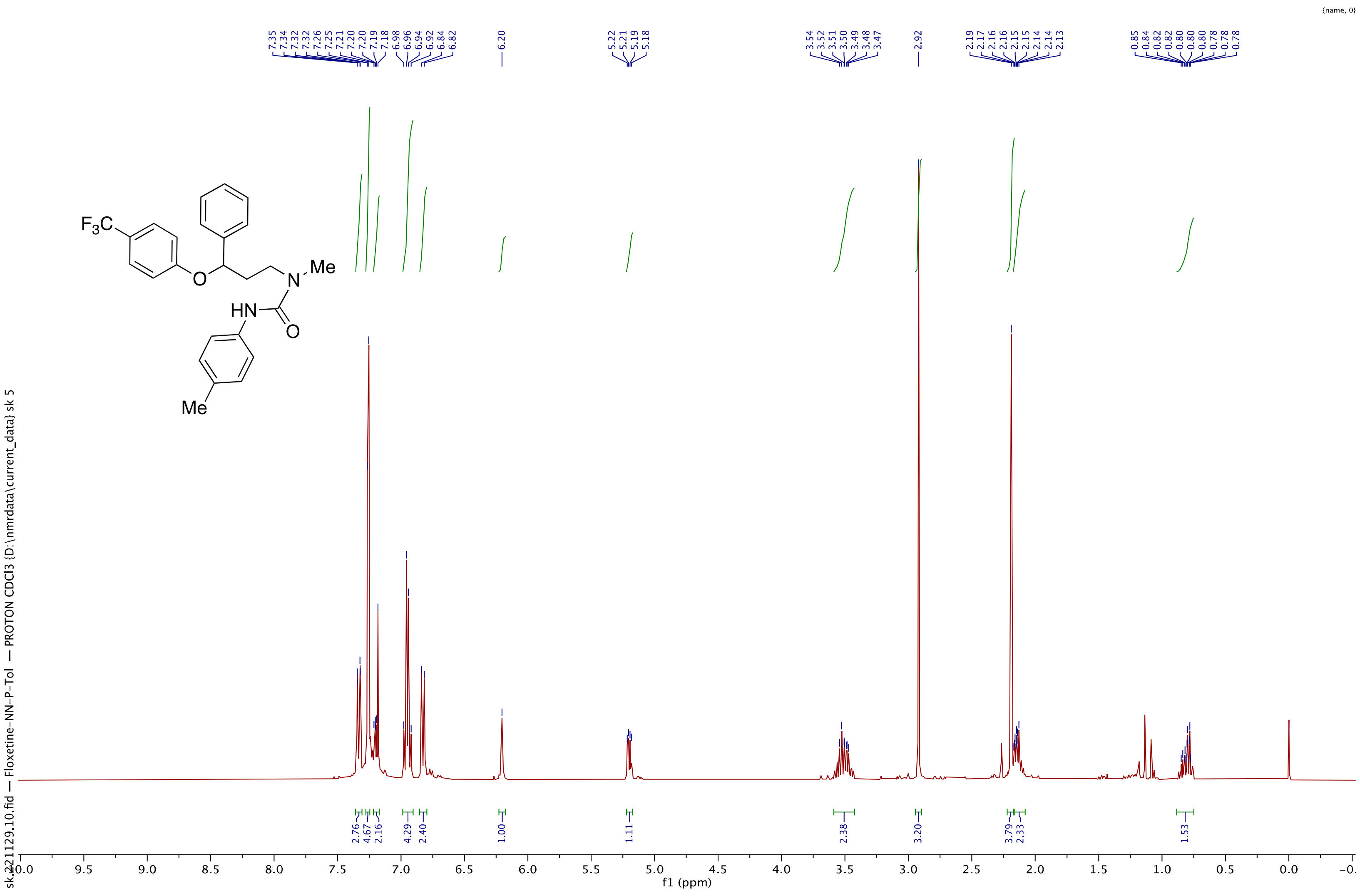

^{1}H NMR spectra of **9g** (400 MHz, RT, $CDCl_3$)

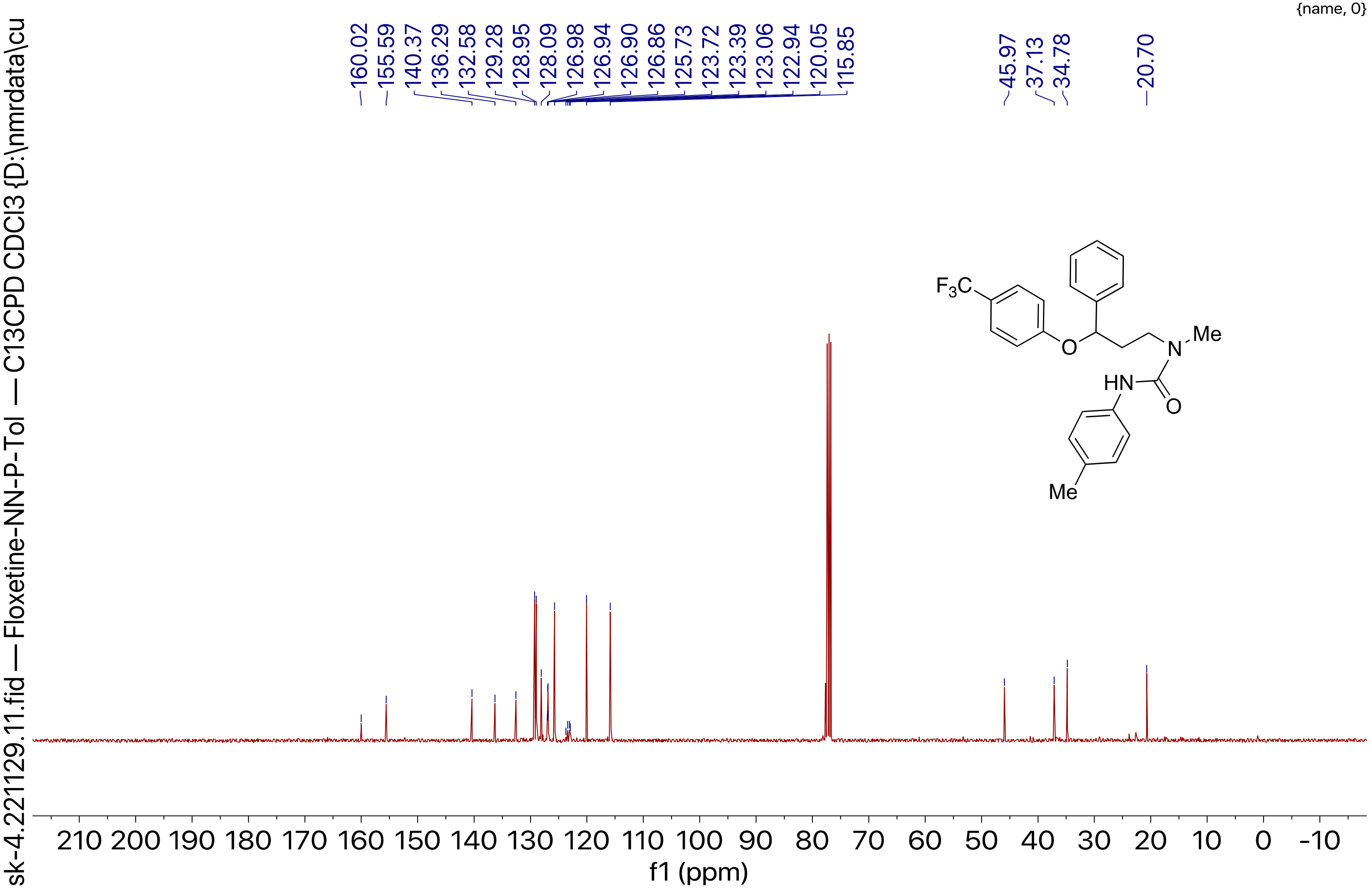

^{13}C NMR spectra of **9g** (101 MHz, RT, $CDCl_3$)

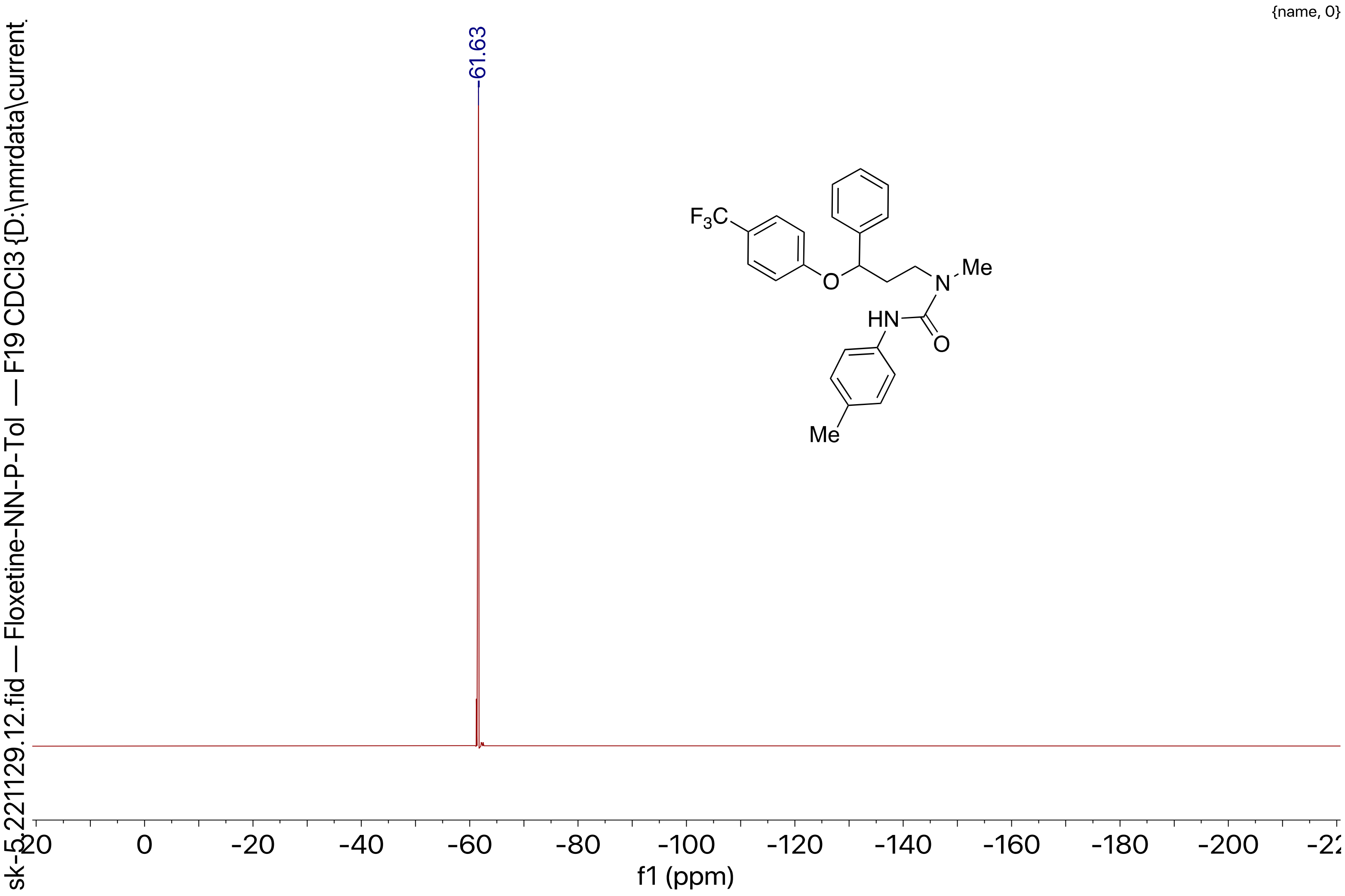

^{19}F NMR spectra of **9g** (376 MHz, RT, $CDCl_3$)

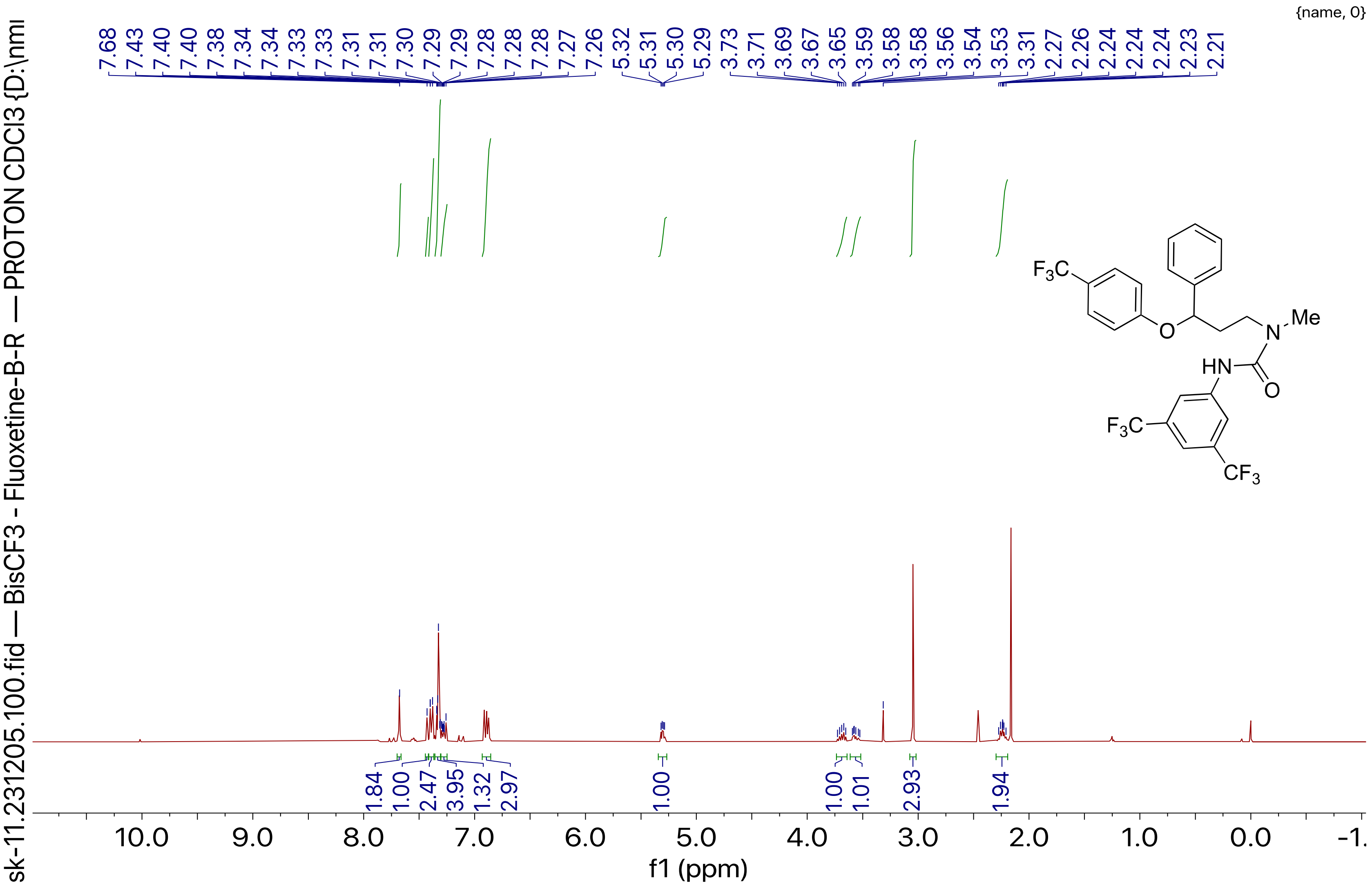

^{1}H NMR spectra of **9g'** (400 MHz, RT, $CDCl_3$)

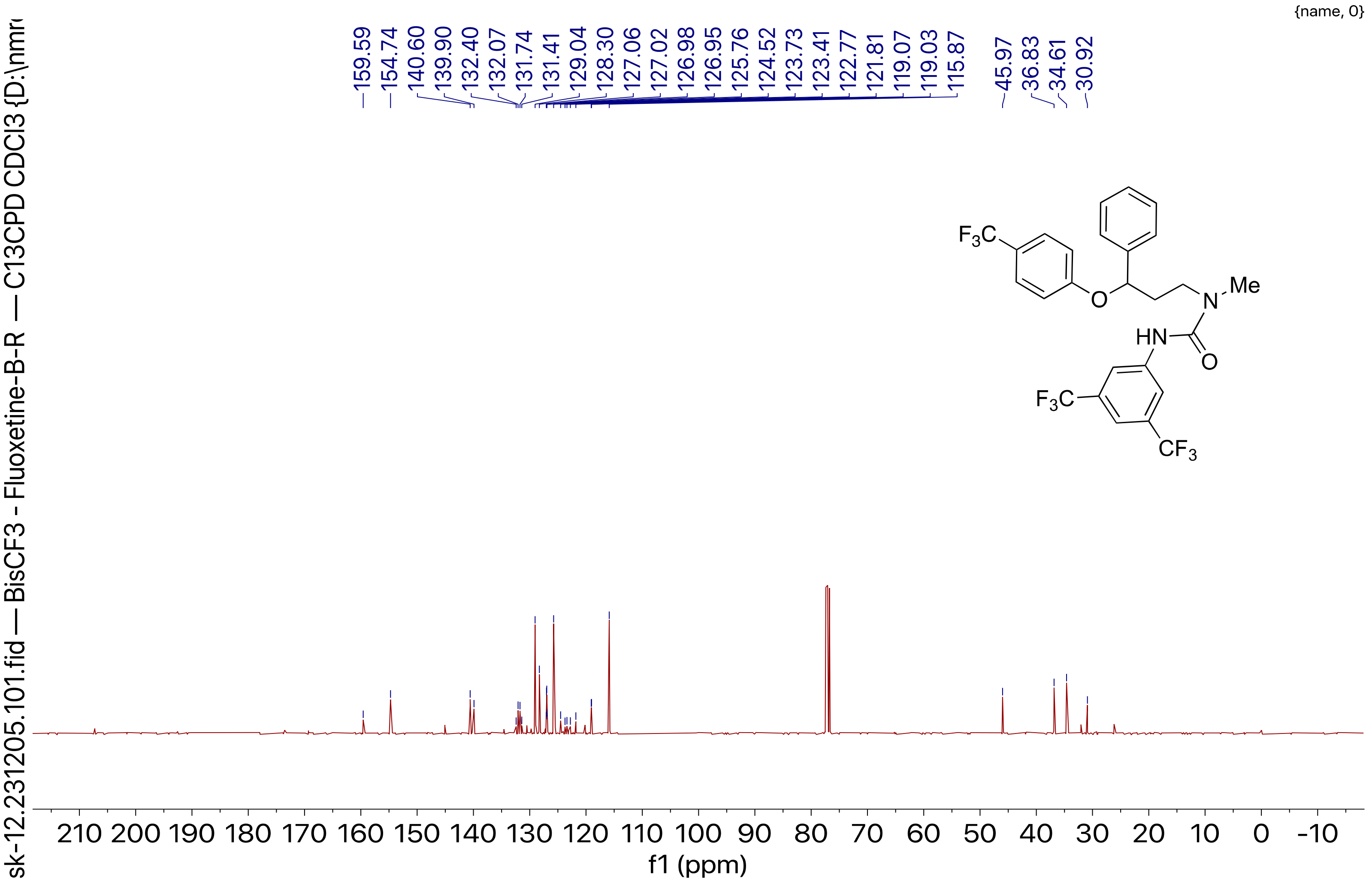

^{13}C NMR spectra of **9g’** (101 MHz, RT, $CDCl_3$)

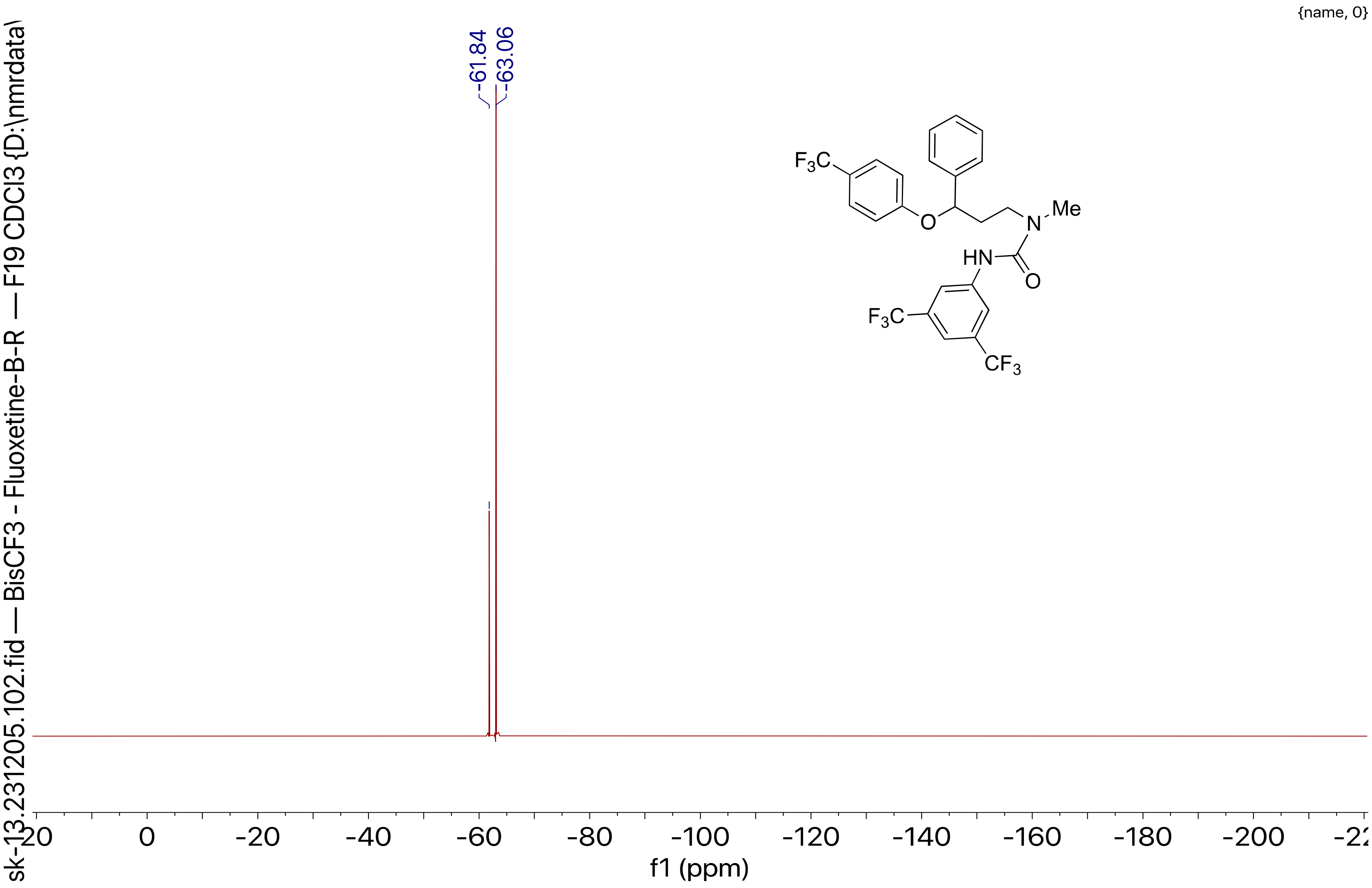

^{19}F NMR spectra of **9g’** (376 MHz, RT, $CDCl_3$)

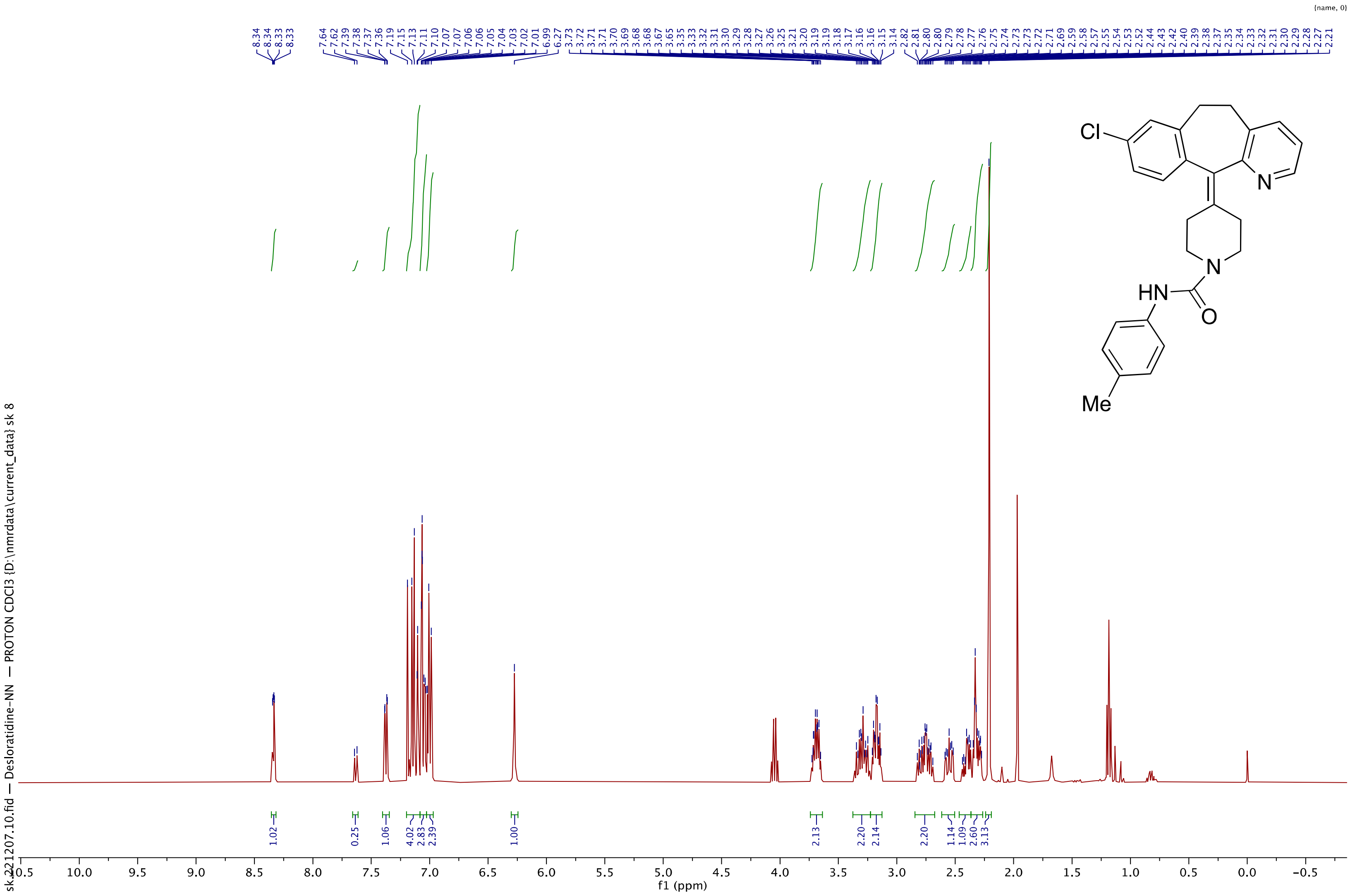

^{1}H NMR spectra of **9h** (400 MHz, RT, $CDCl_3$)

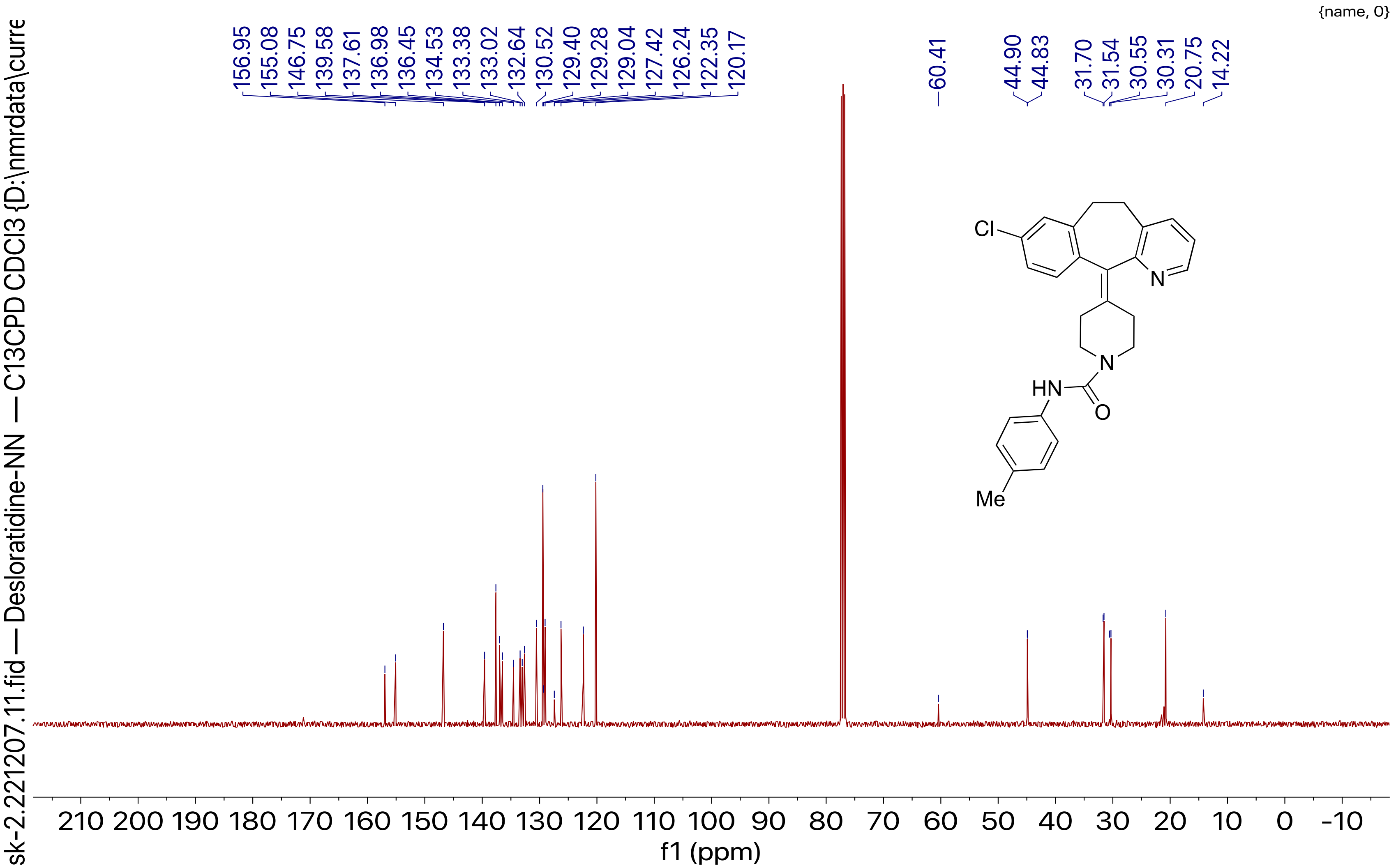

^{13}C NMR spectra of **9h** (101 MHz, RT, $CDCl_3$)

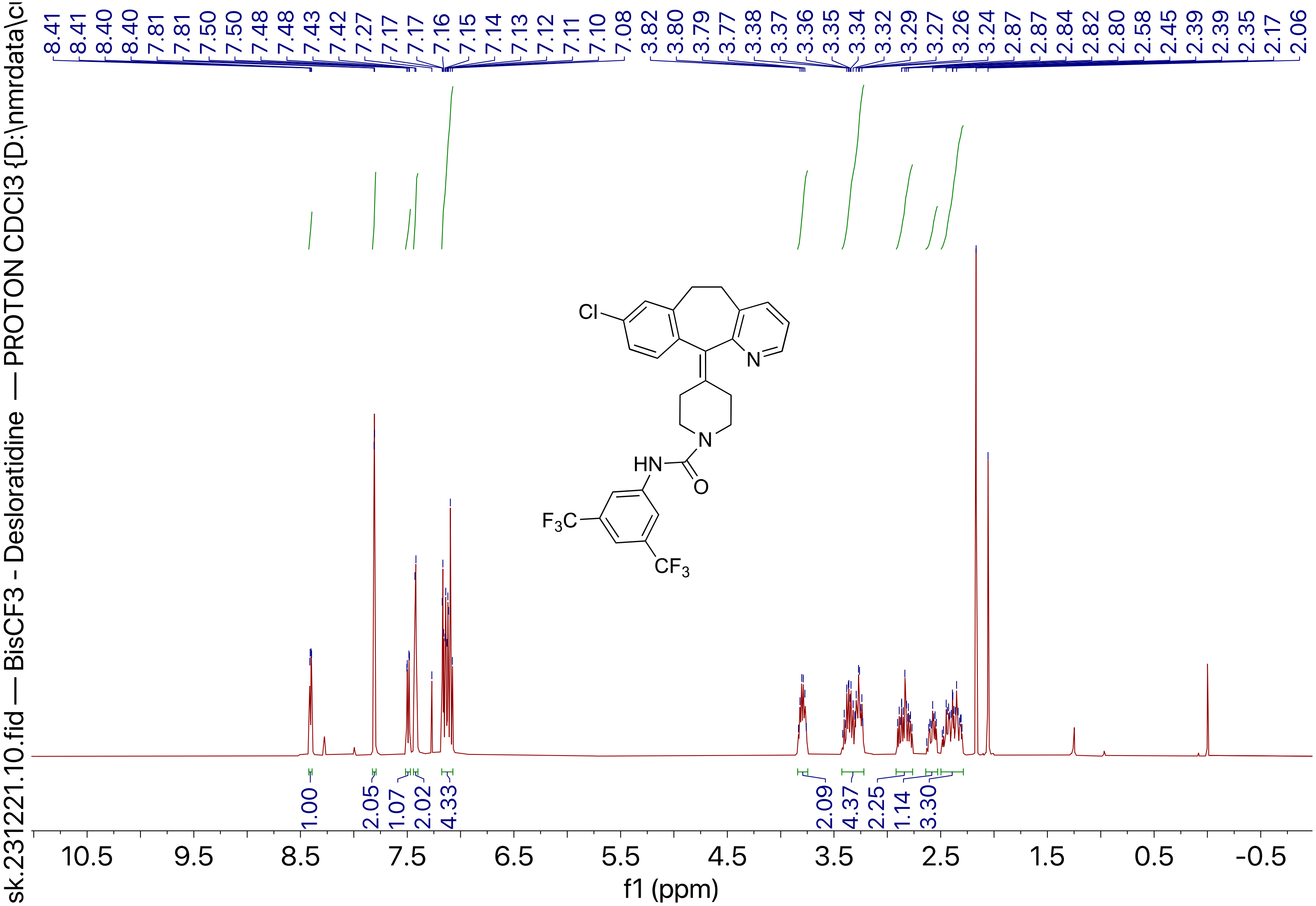

^{1}H NMR spectra of **9h’** (400 MHz, RT, $CDCl_3$)

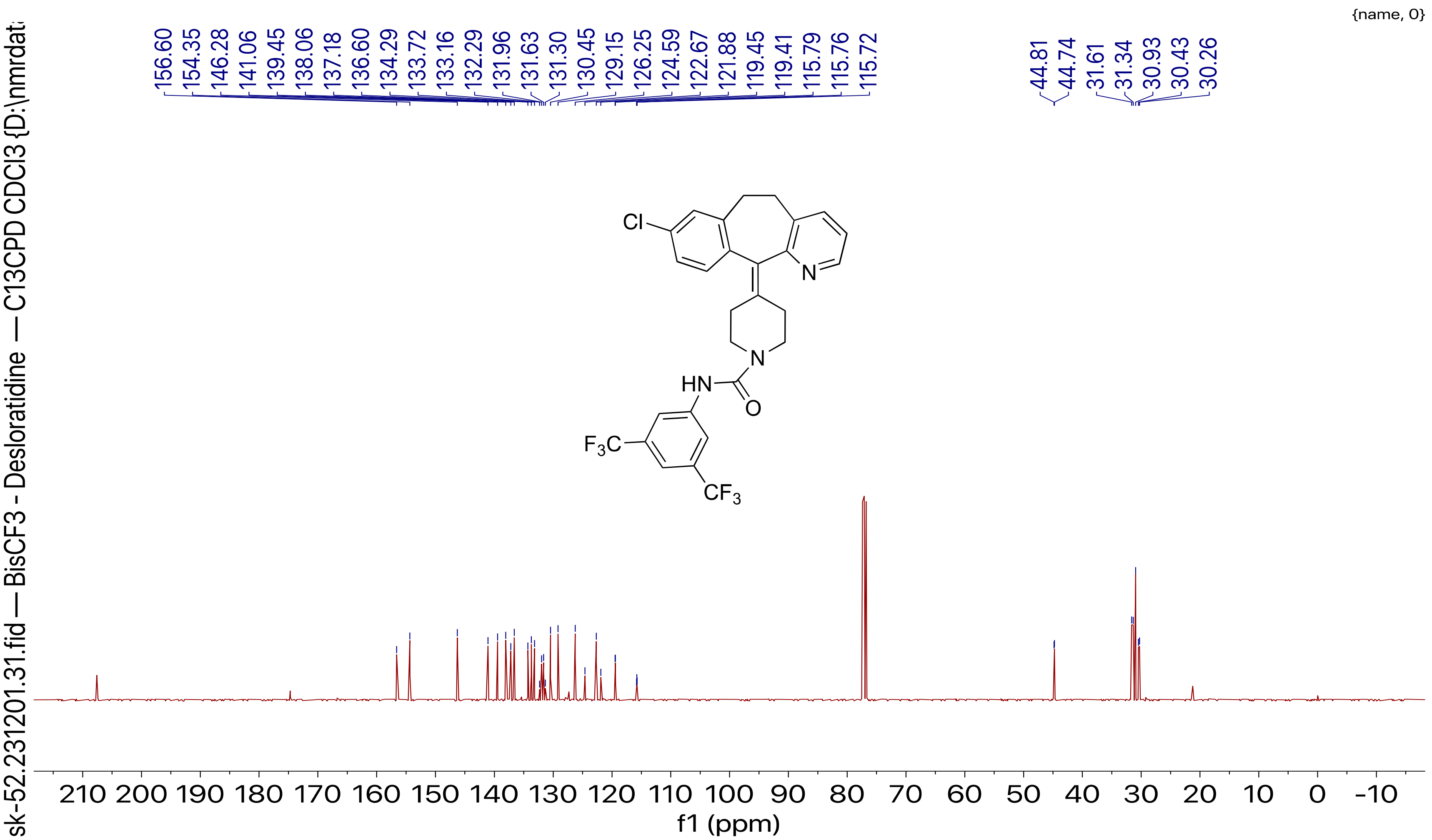

^{13}C NMR spectra of **9h'** (101 MHz, $CDCl_3$, RT)

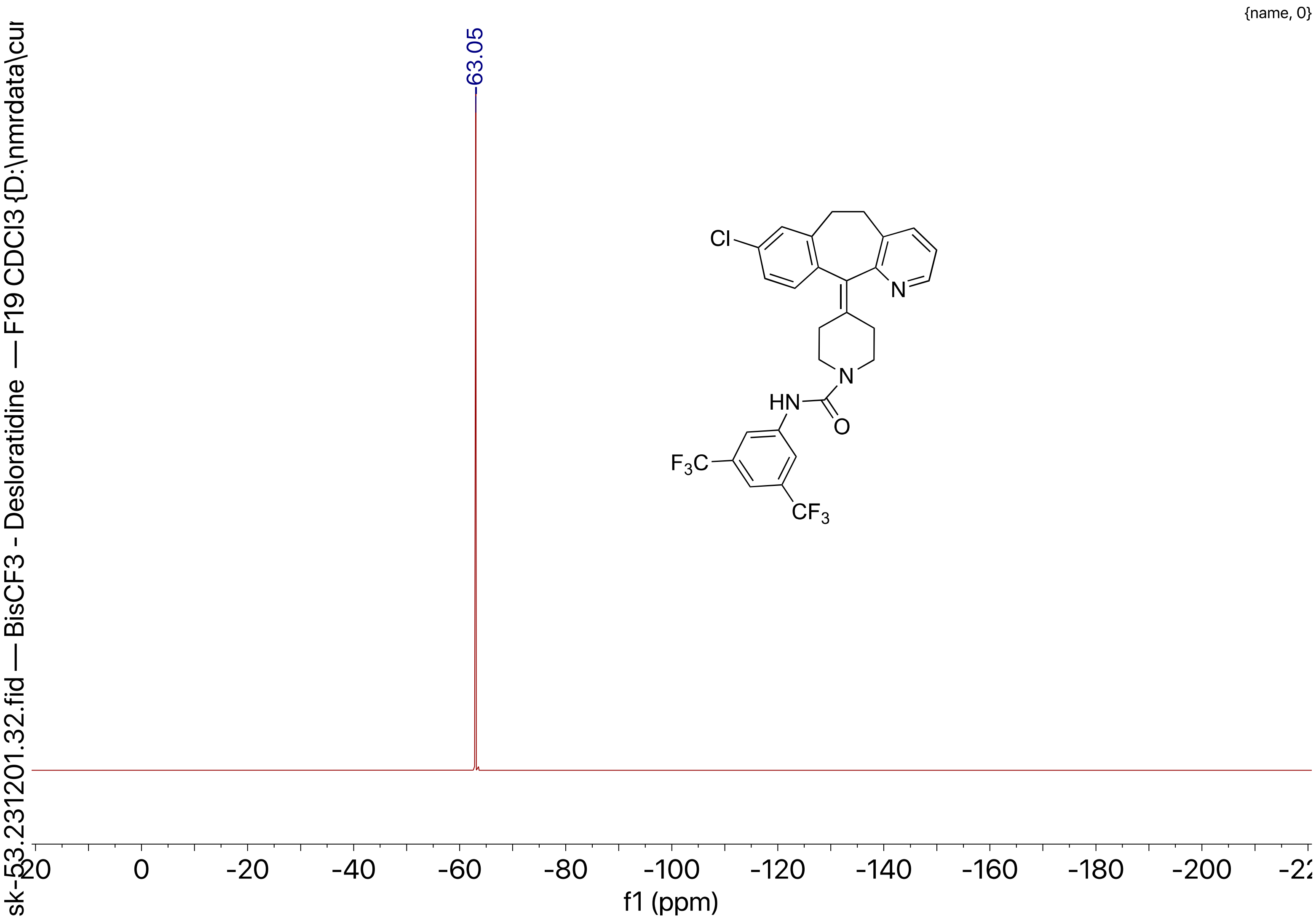

^{19}F NMR spectra of **9h'** (376 MHz, $CDCl_3$, RT)

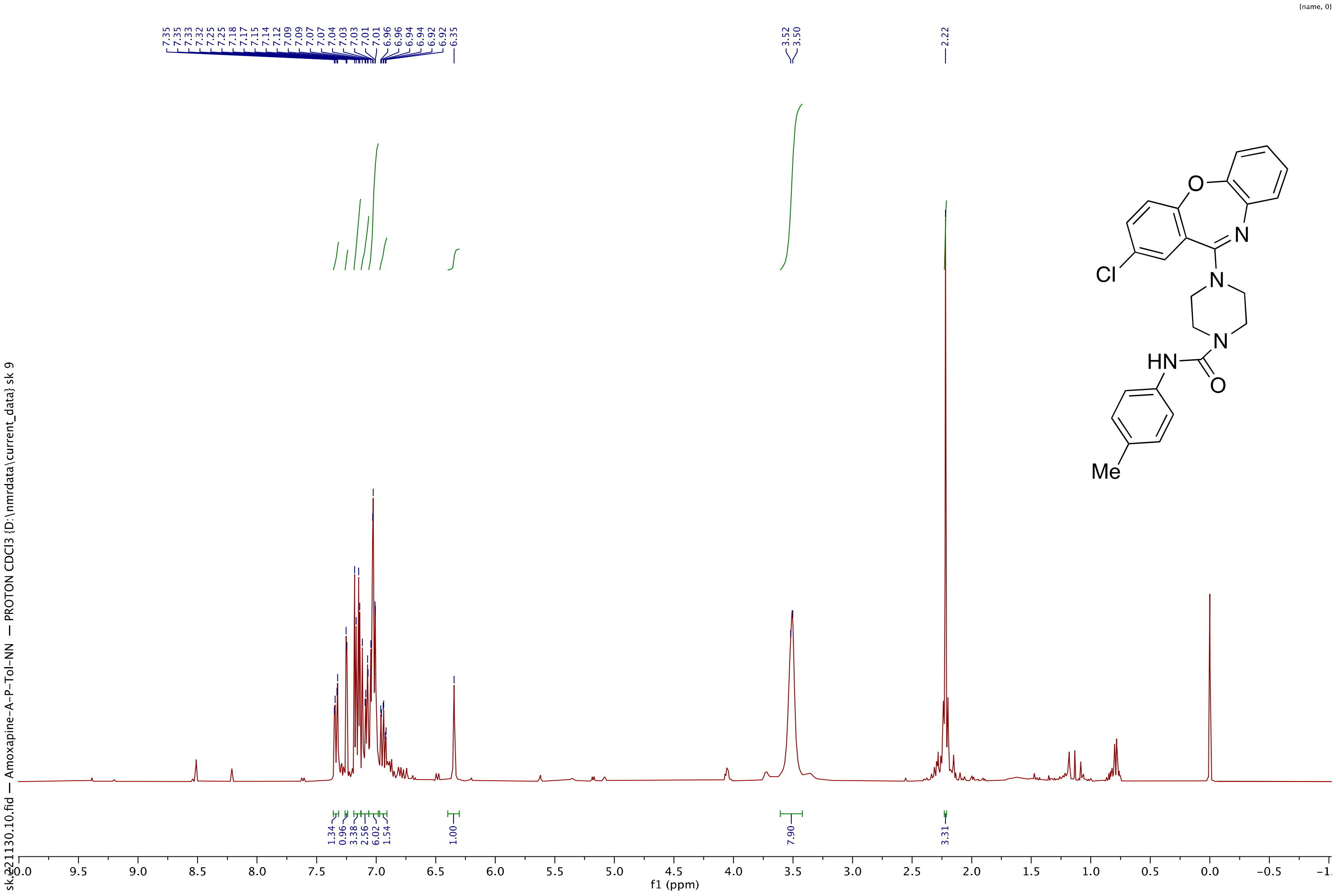

^{1}H NMR spectra of **9i** (400 MHz, RT, $CDCl_3$)

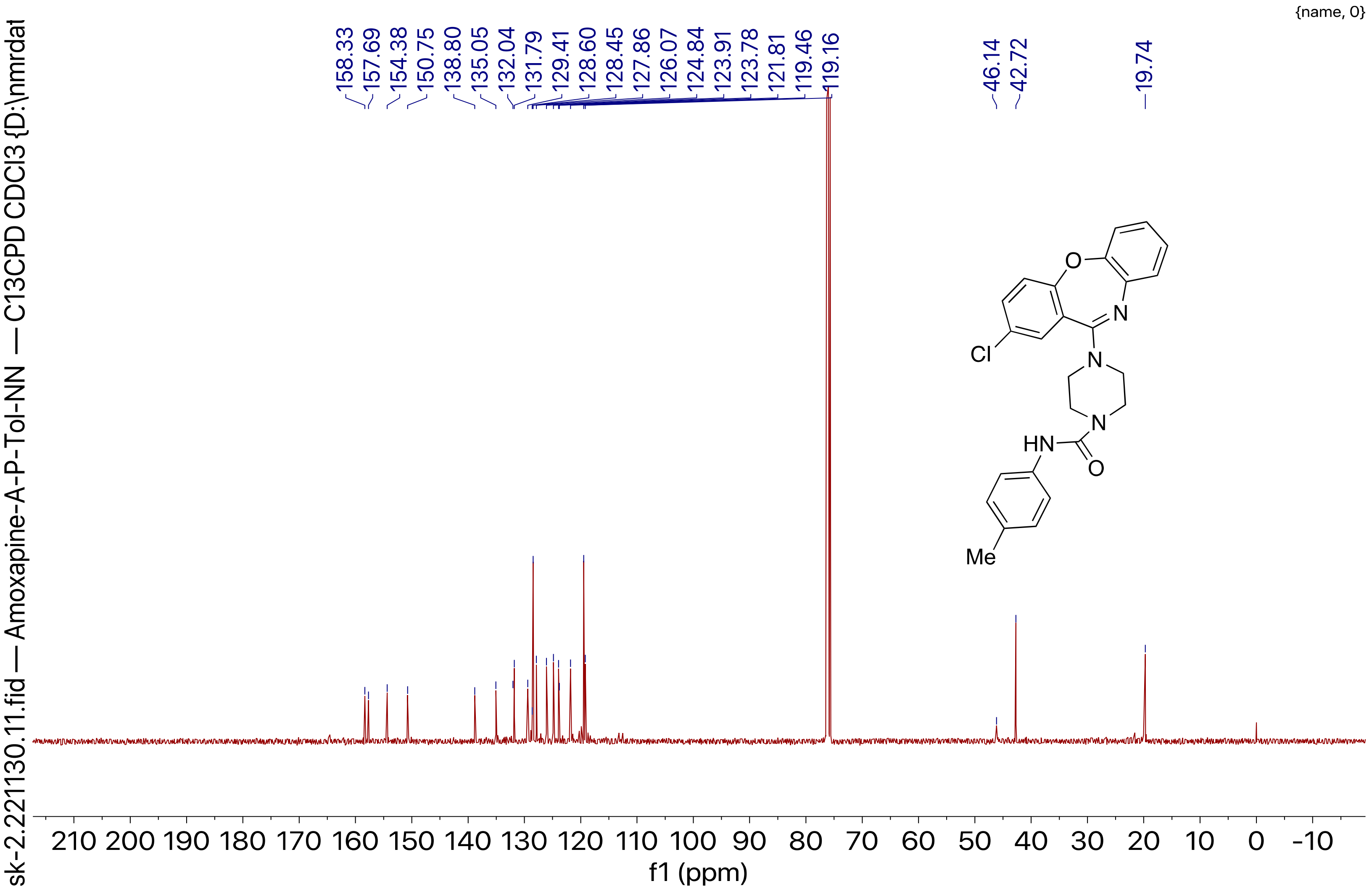

^{13}C NMR spectra of **9i** (101 MHz, RT, $CDCl_3$)

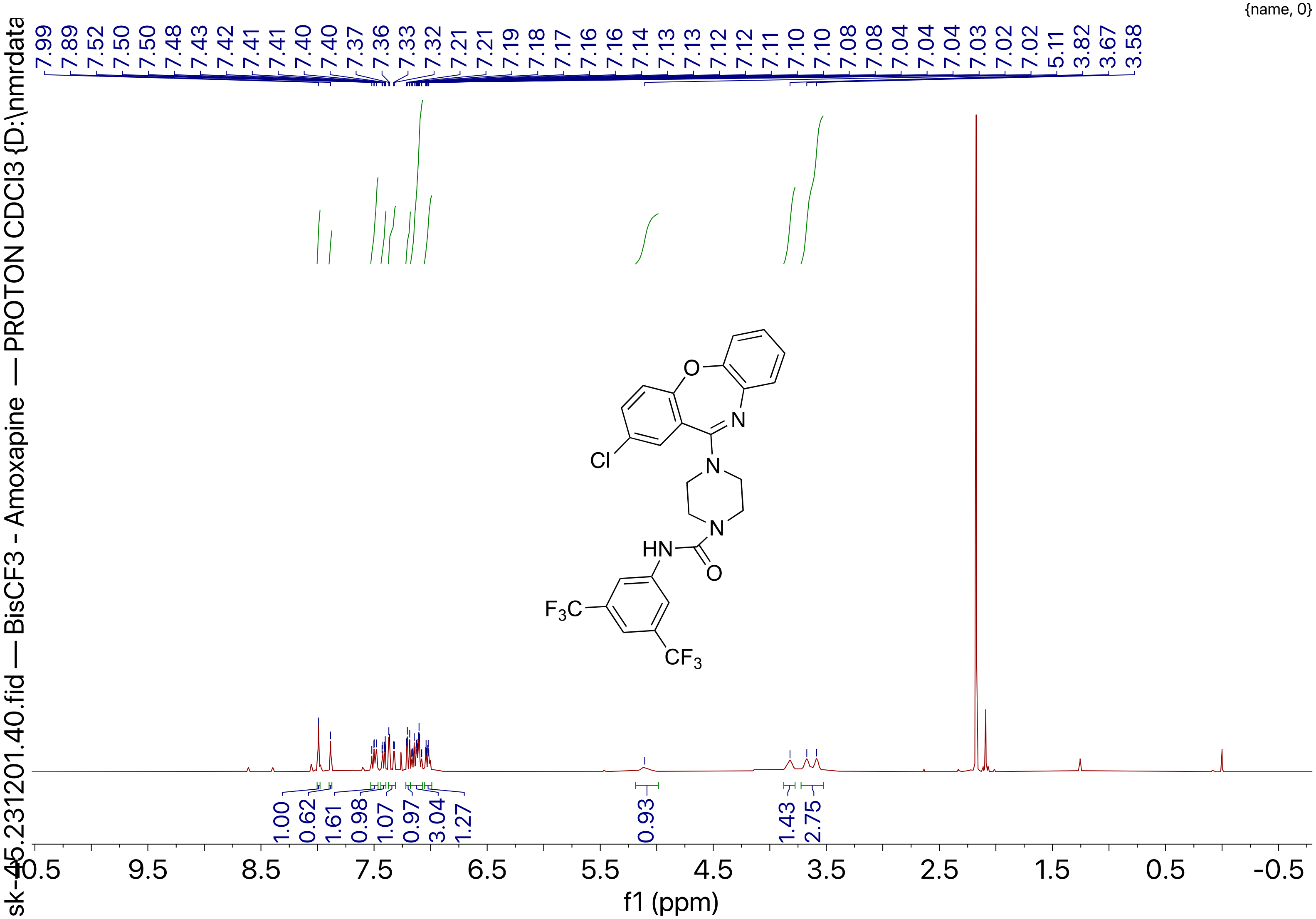

^{1}H NMR spectra of **9i'** (400 MHz, RT, $CDCl_3$)

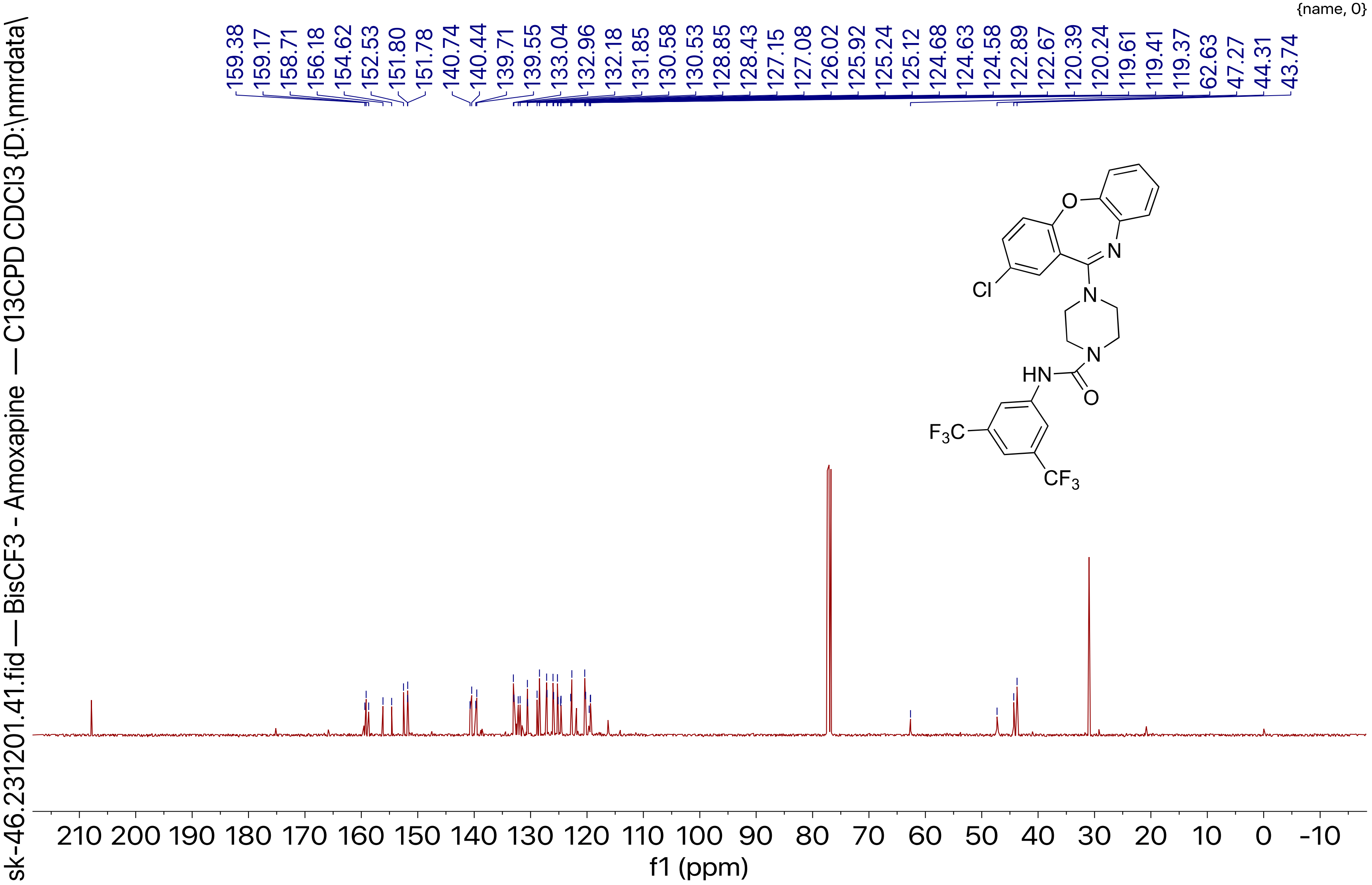

^{13}C NMR spectra of **9i’** (101 MHz, $CDCl_3$, RT)

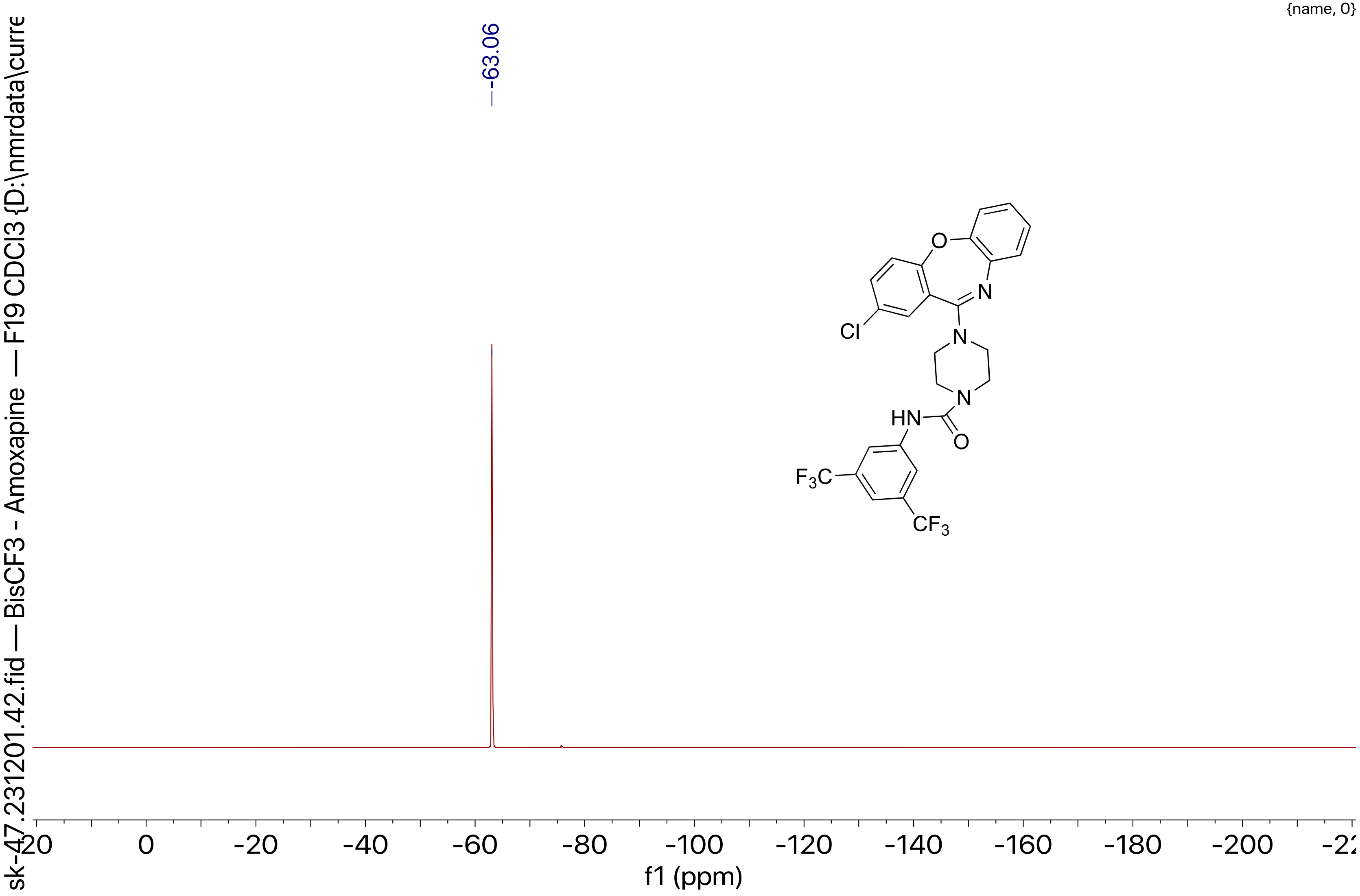

^{19}F NMR spectra of **9i’** (376 MHz, RT, $CDCl_3$)

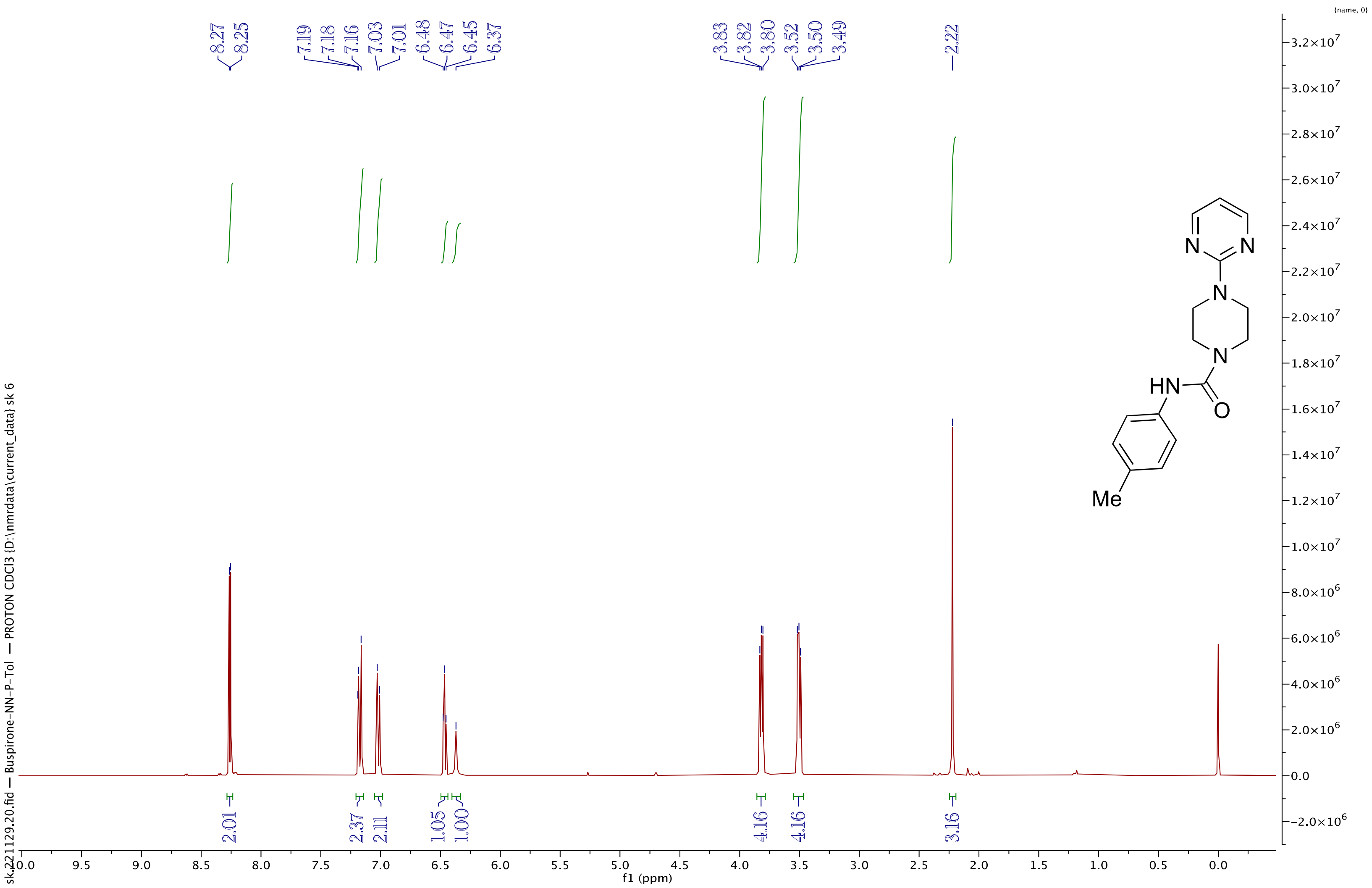

^{1}H NMR spectra of **9j** (400 MHz, RT, $CDCl_3$)

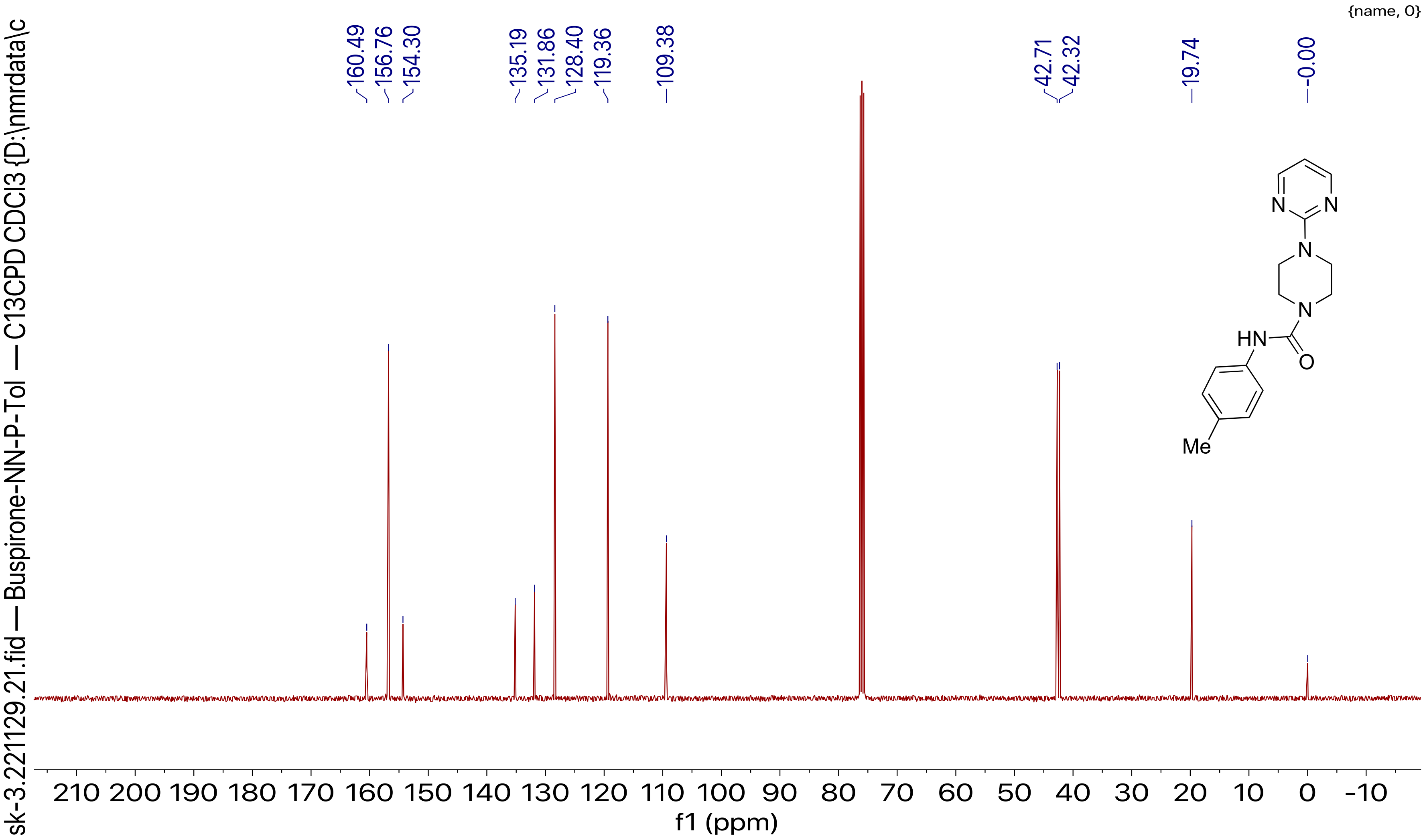

^{13}C NMR spectra of **9j** (101 MHz, RT, $CDCl_3$)

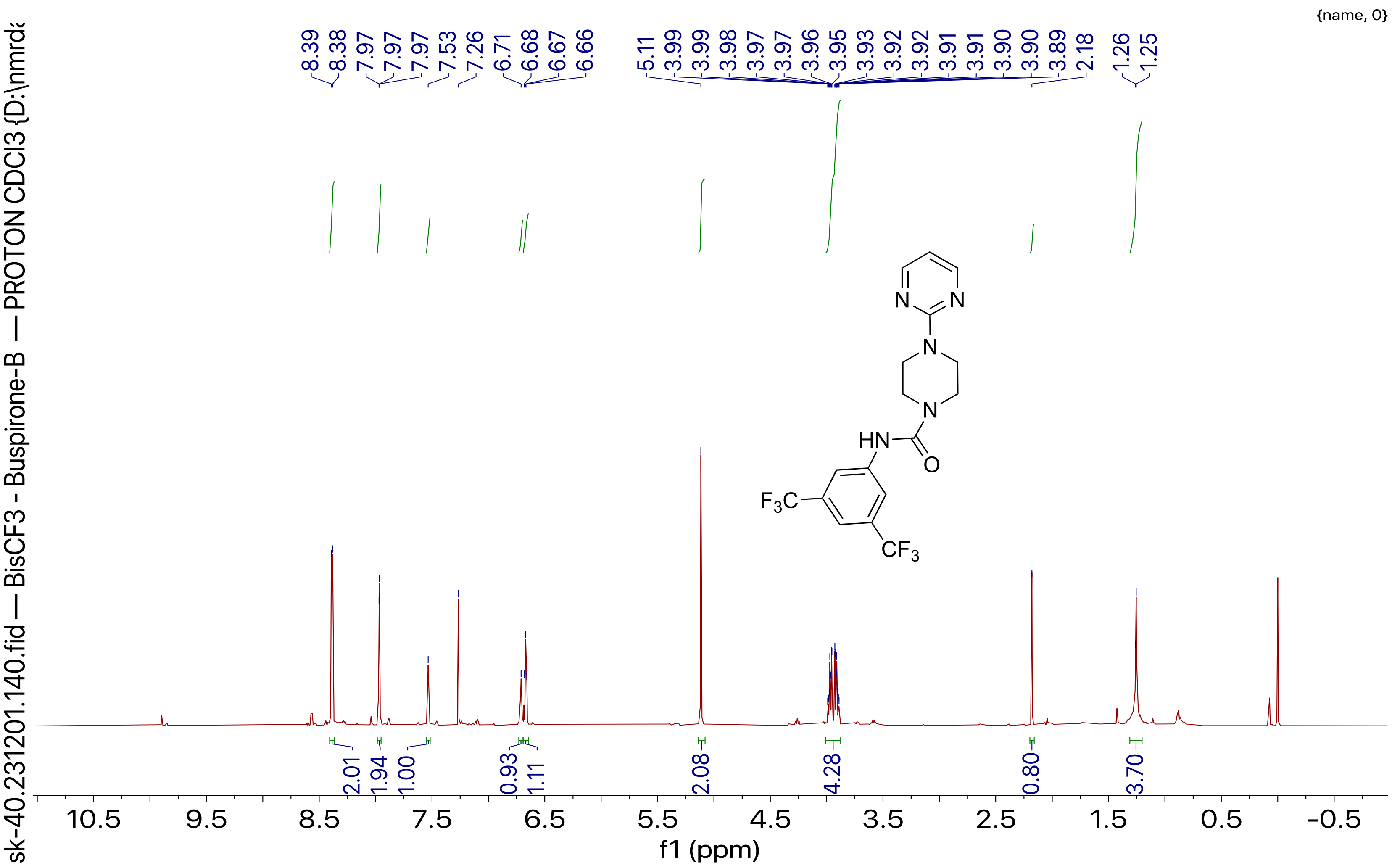

^{1}H NMR spectra of **9j’** (400 MHz, RT, $CDCl_3$)

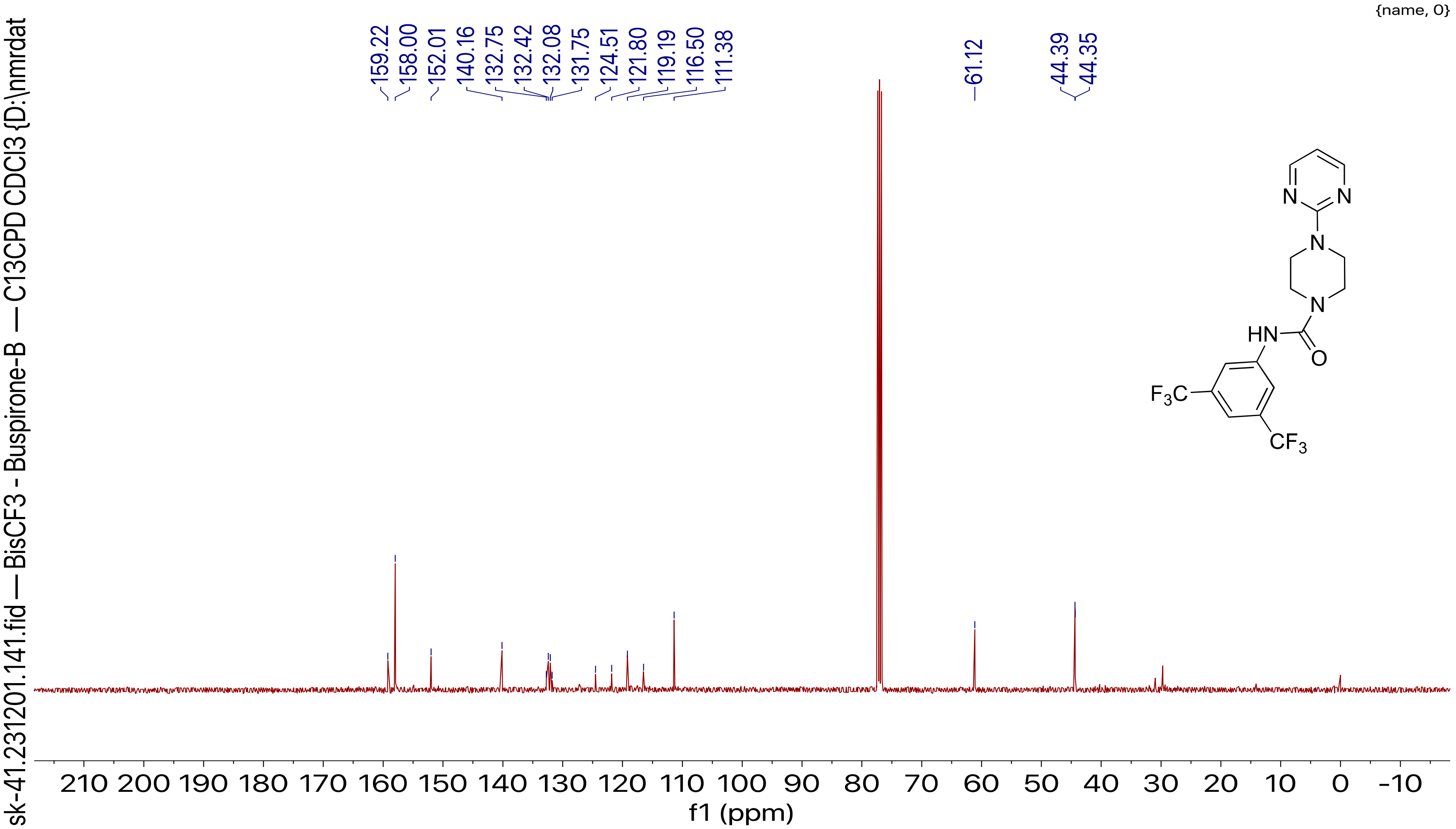

^{13}C NMR spectra of **9j’** (101 MHz, $CDCl_3$, RT)

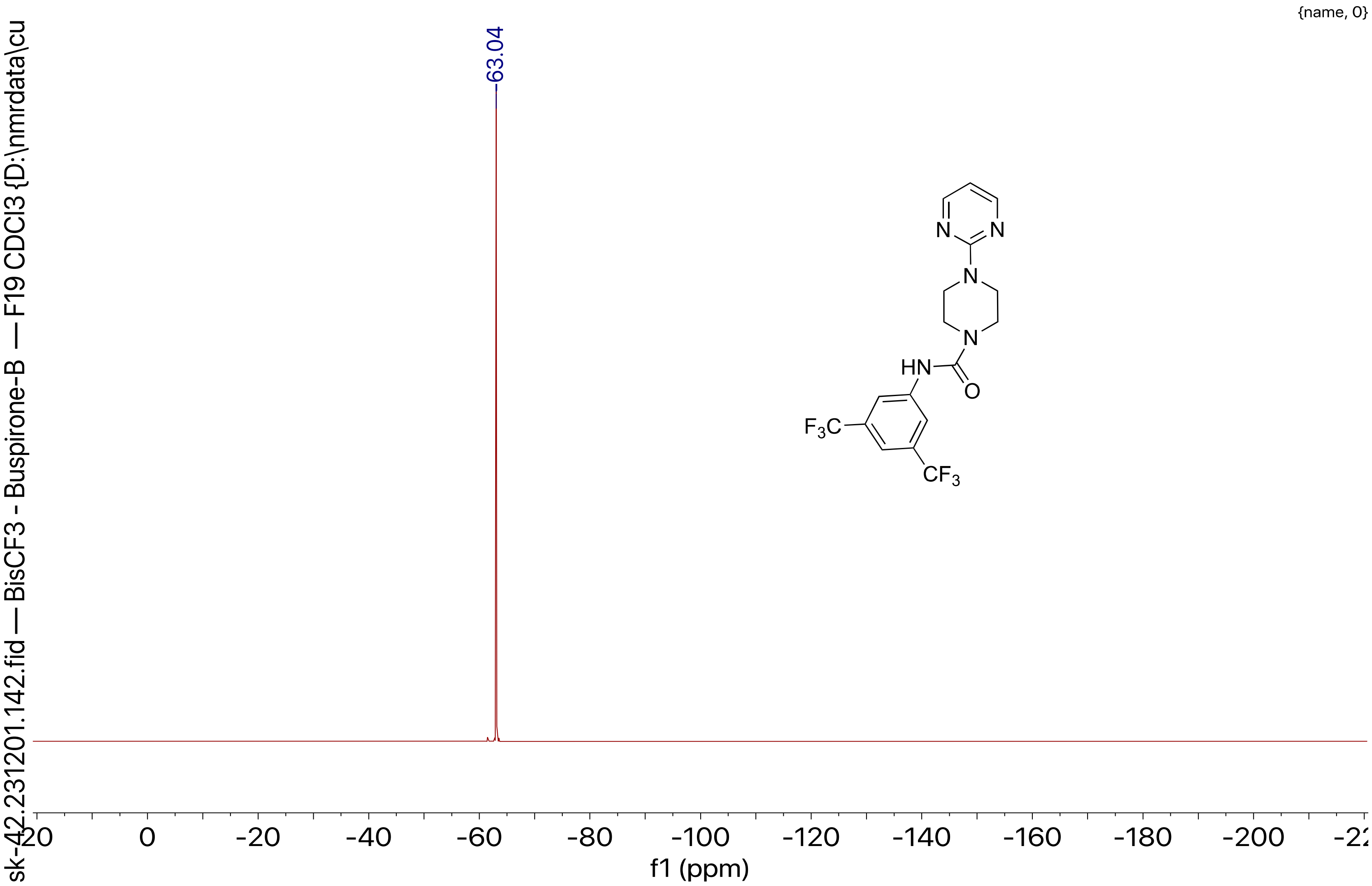

^{19}F NMR spectra of **9j’** (376 MHz, RT, $CDCl_3$)

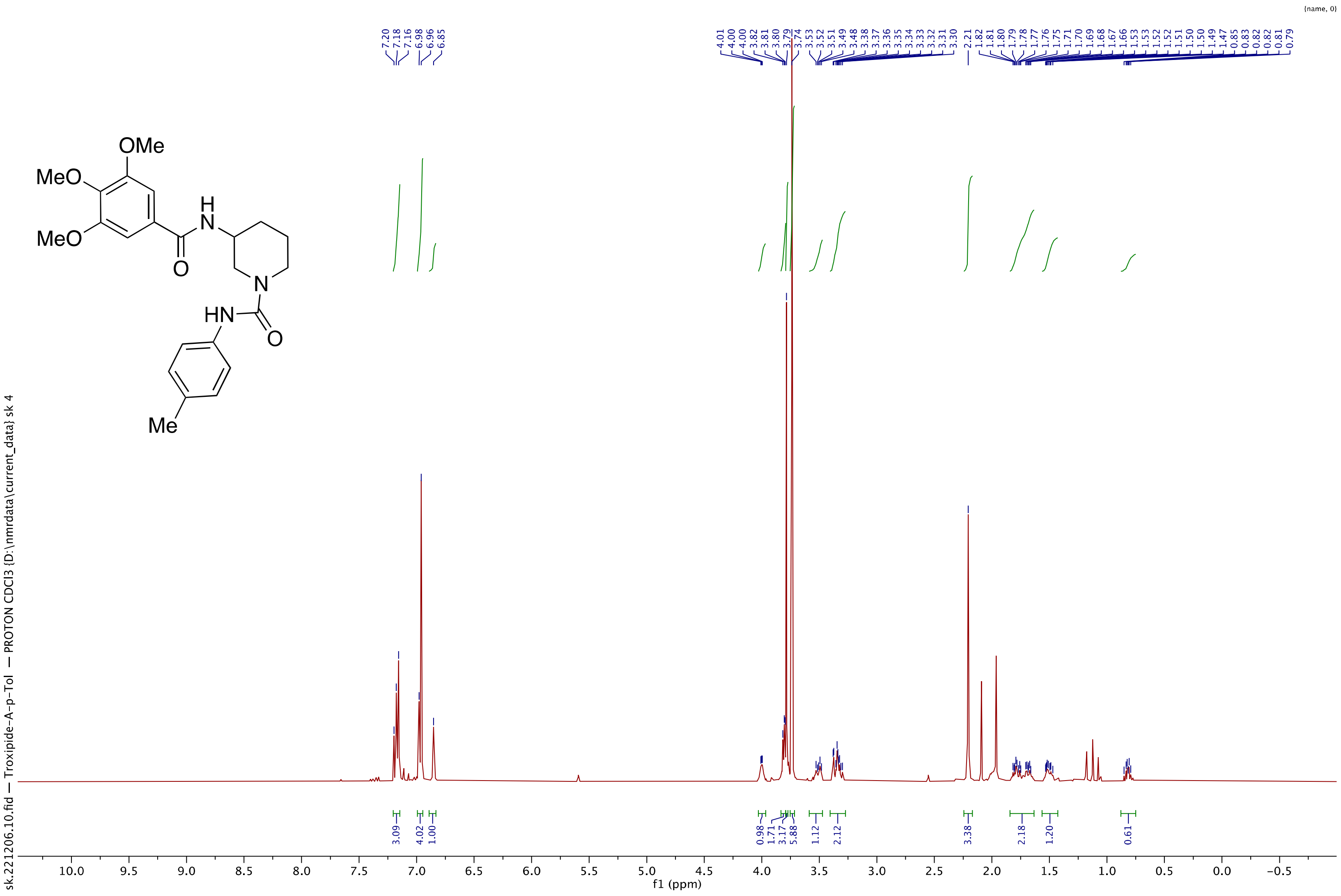

^{1}H NMR spectra of **9k** (400 MHz, RT, $CDCl_3$)

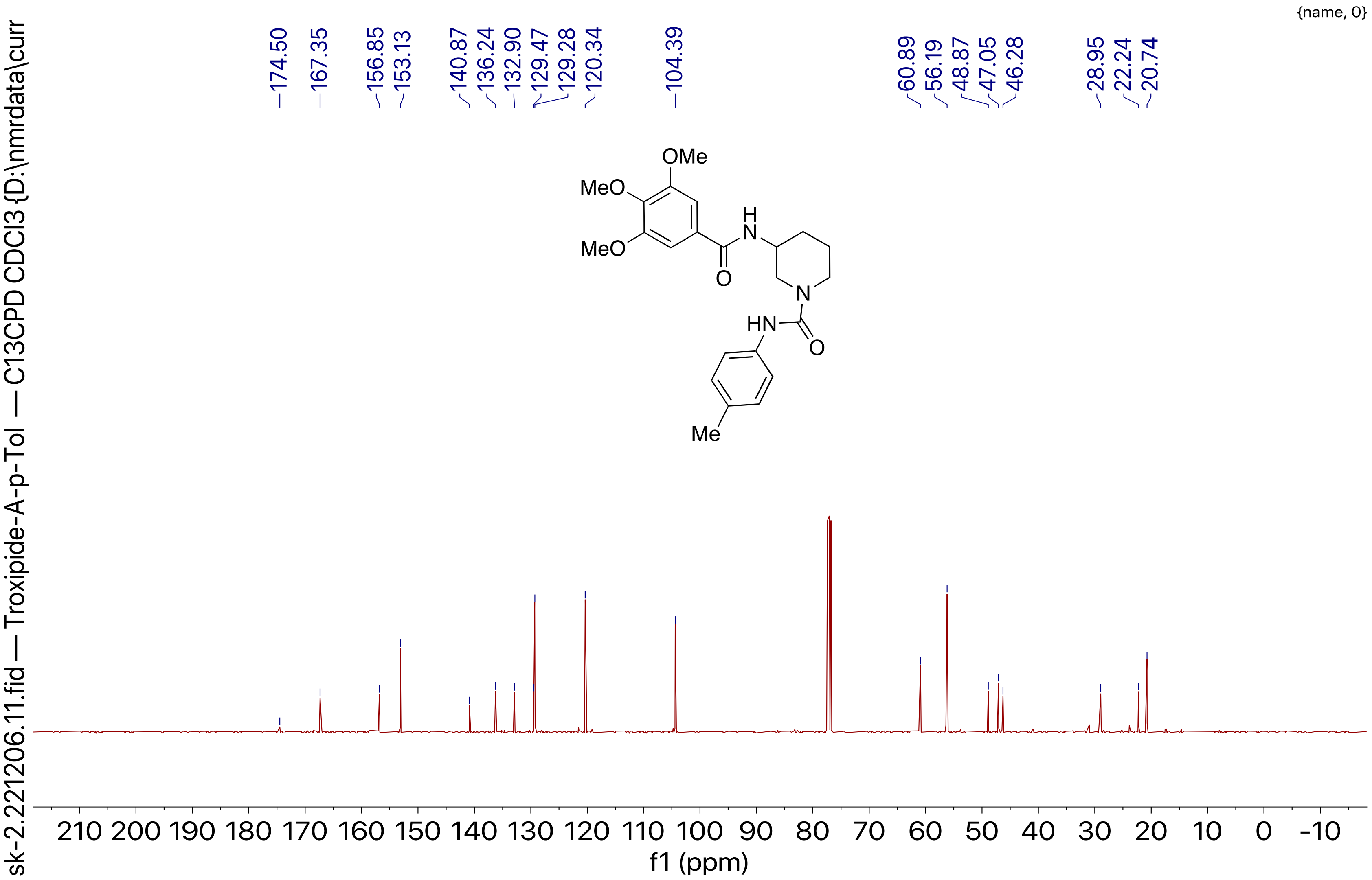

^{13}C NMR spectra of **9k** (100 MHz, RT, $CDCl_3$)

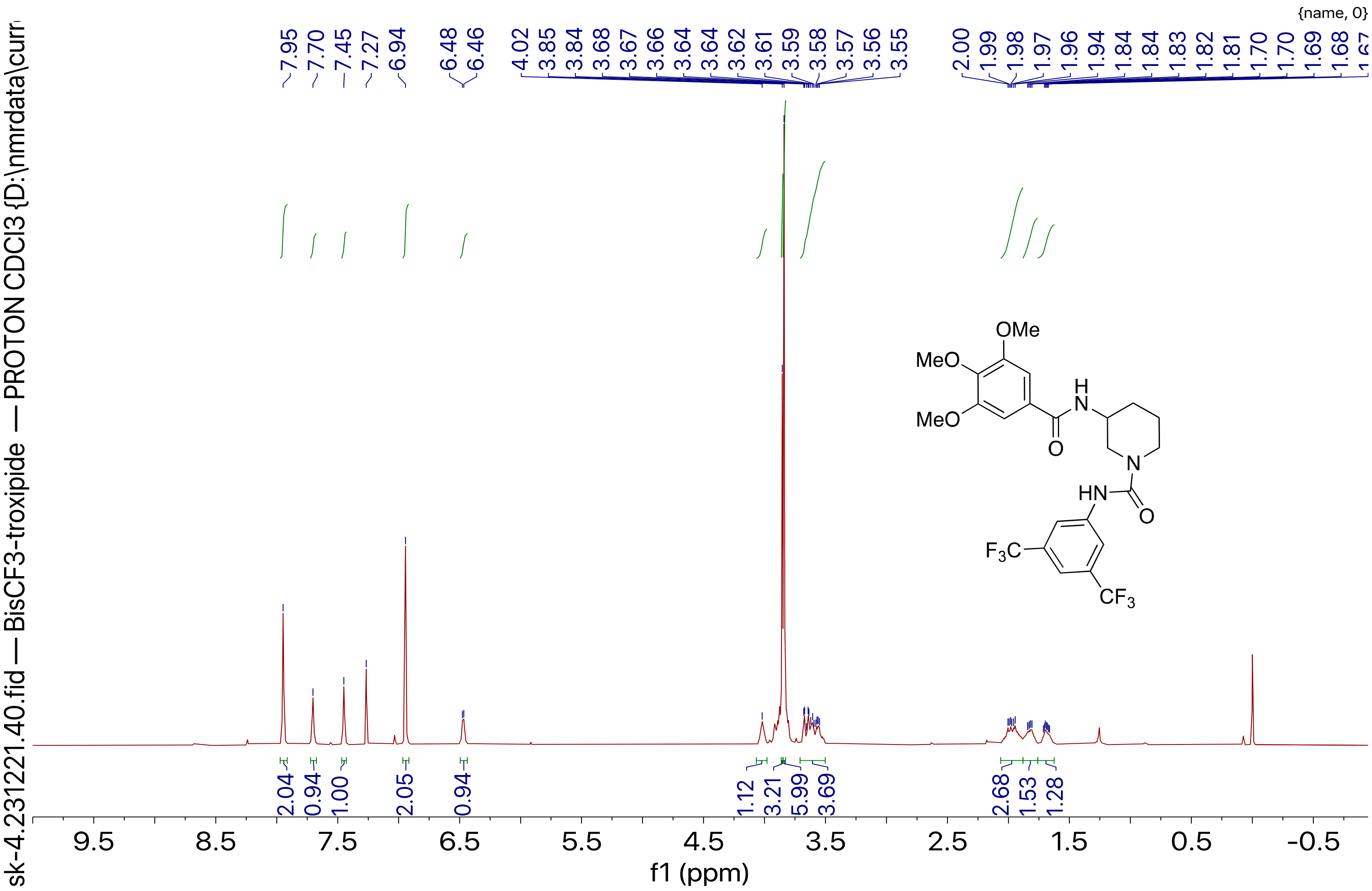

^{1}H NMR spectra of **9k'** (400 MHz, RT, $CDCl_3$)

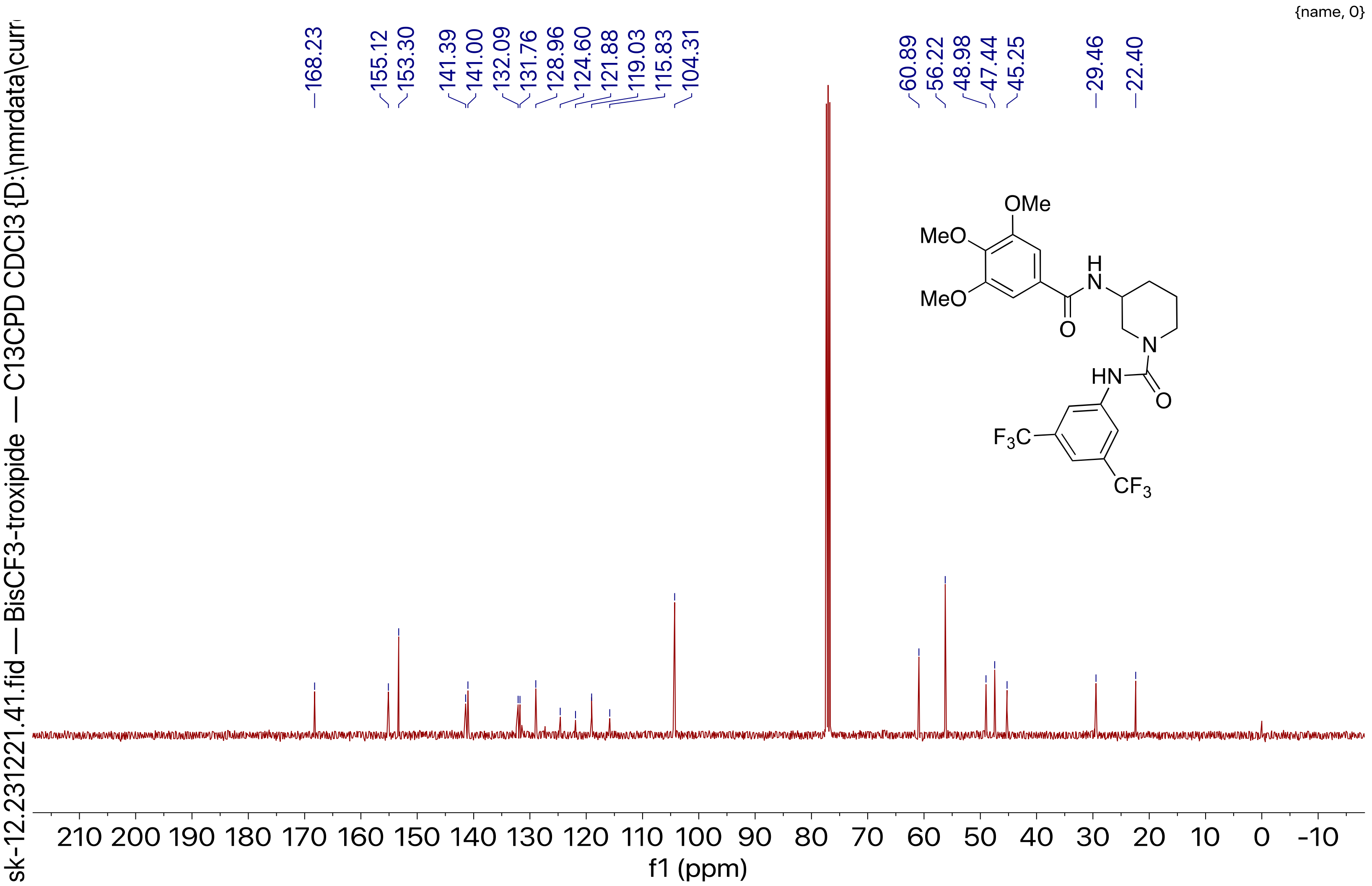

^{13}C NMR spectra of **9k’** (101 MHz, RT, $CDCl_3$)

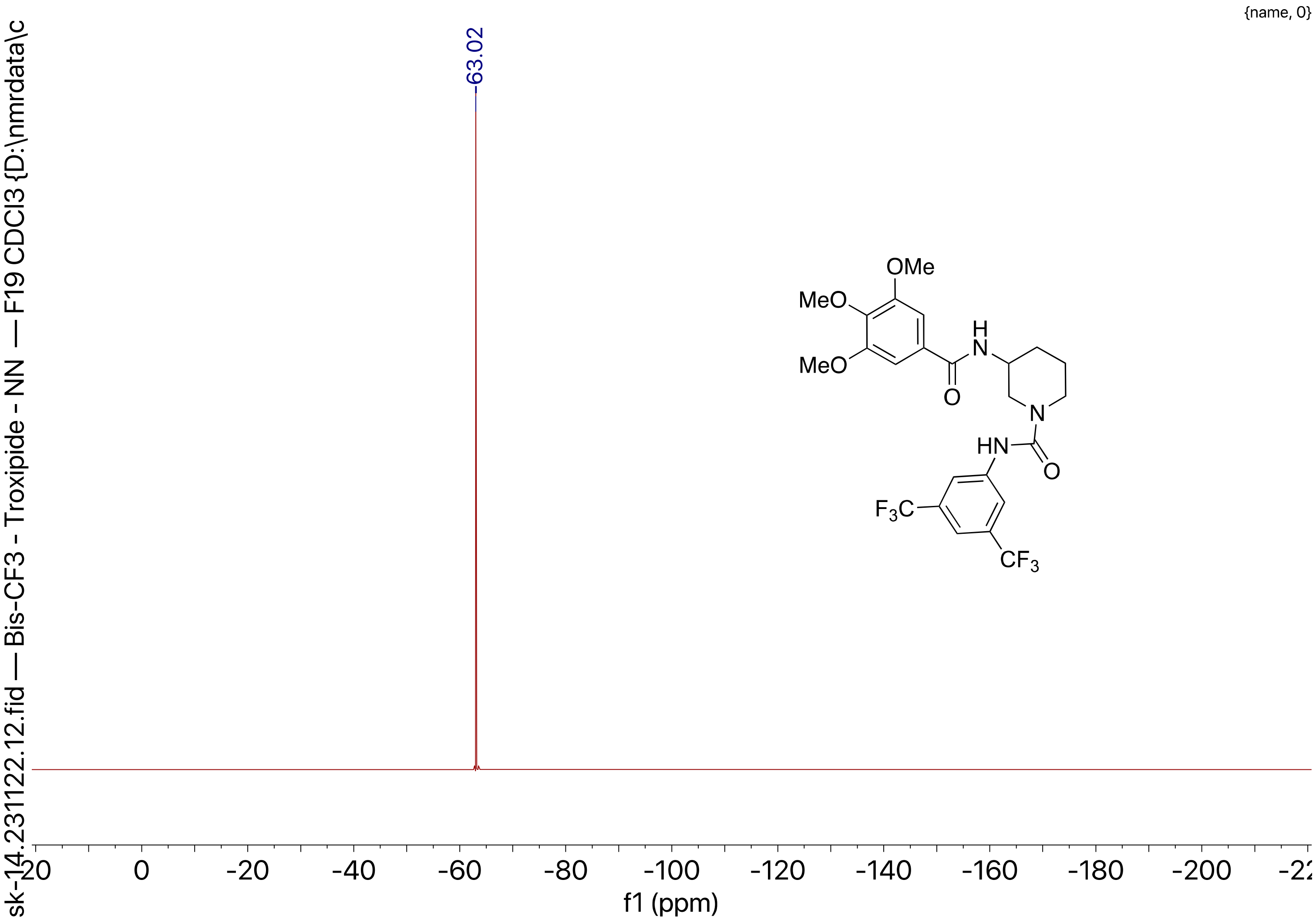

^{19}F NMR spectra of **9k’** (376 MHz, RT, $CDCl_3$)

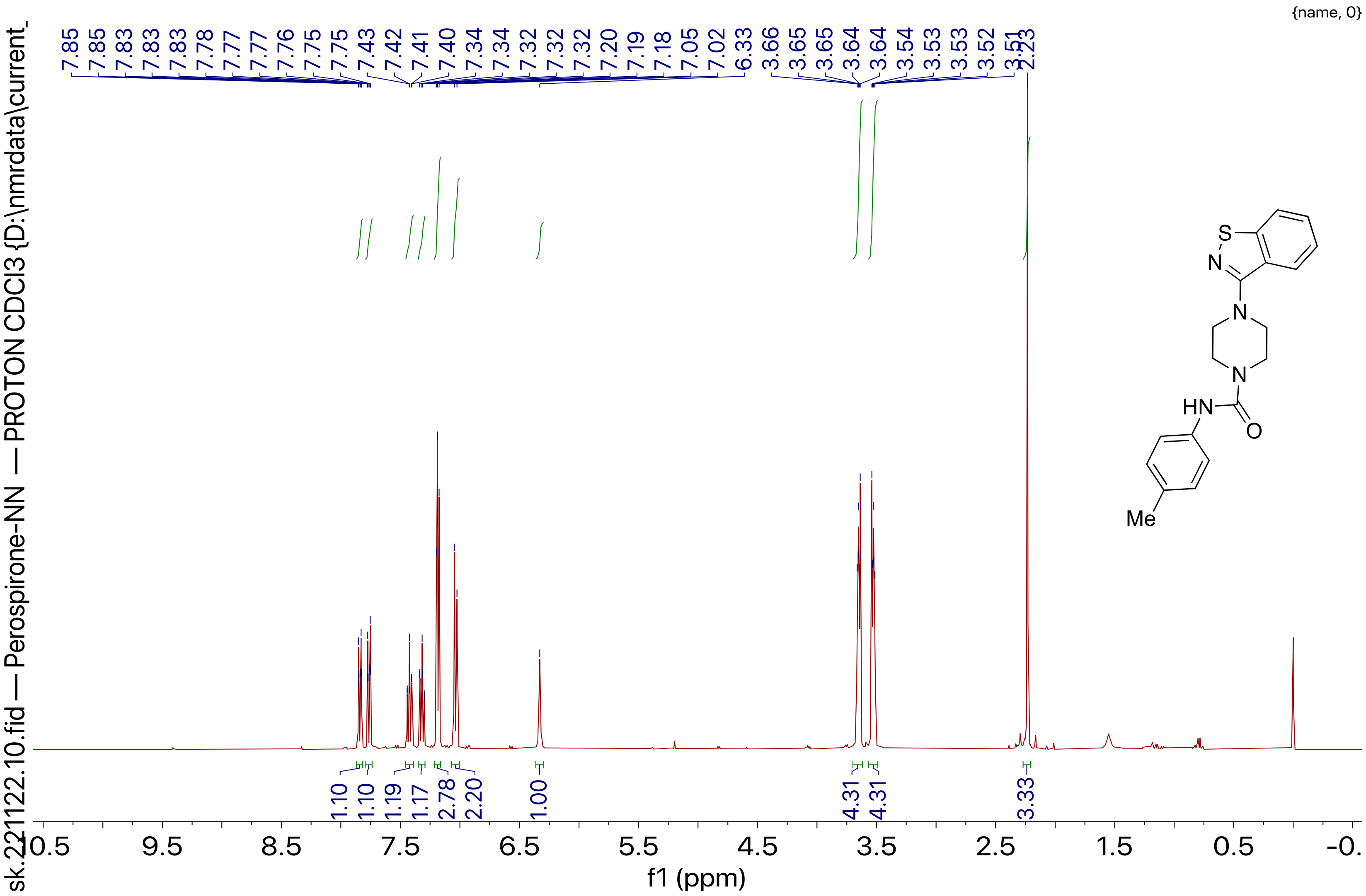

^{1}H NMR spectra of **9l** (400 MHz, RT, $CDCl_3$)

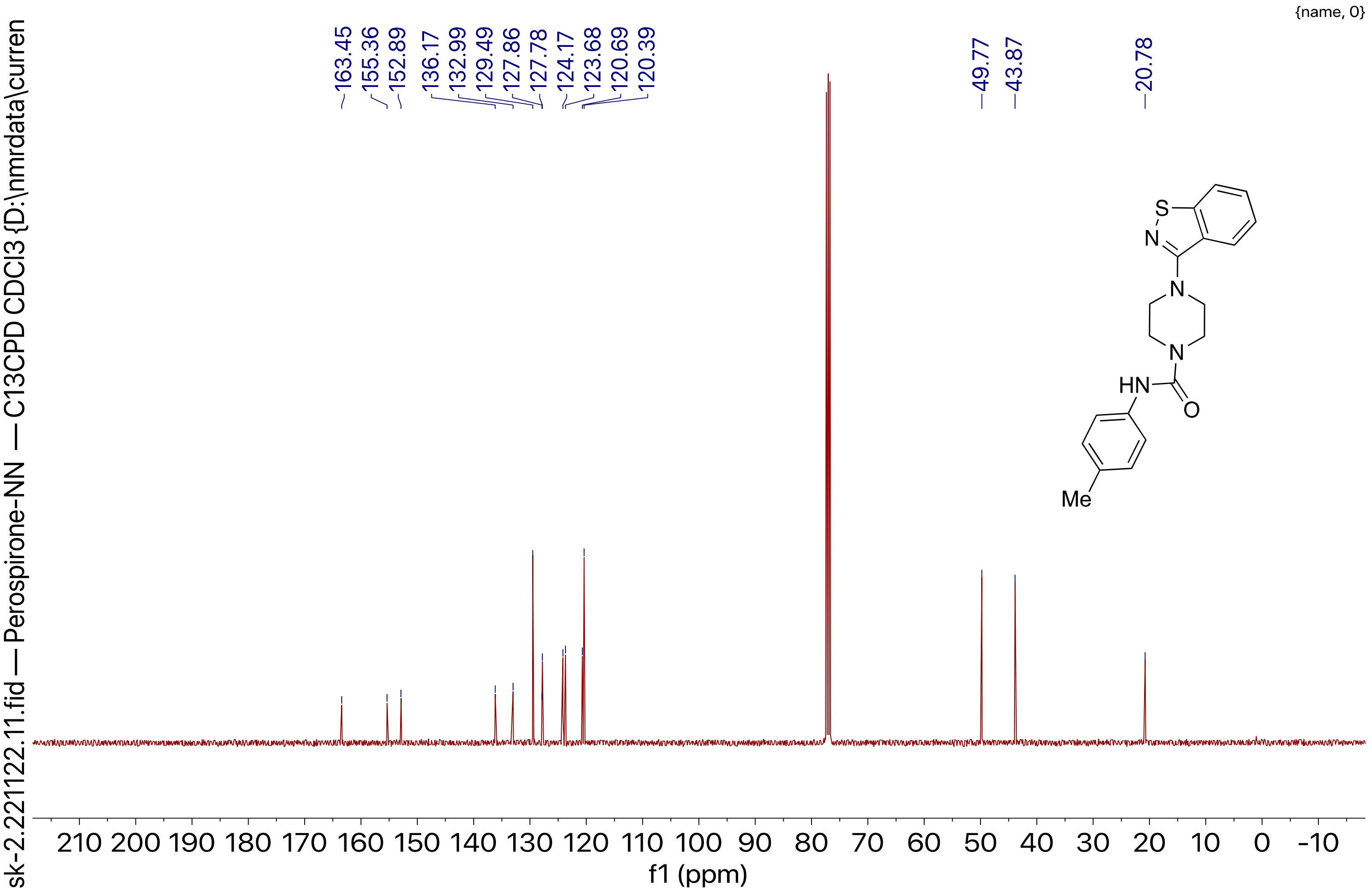

^{13}C NMR spectra of **9l** (101 MHz, RT, $CDCl_3$)

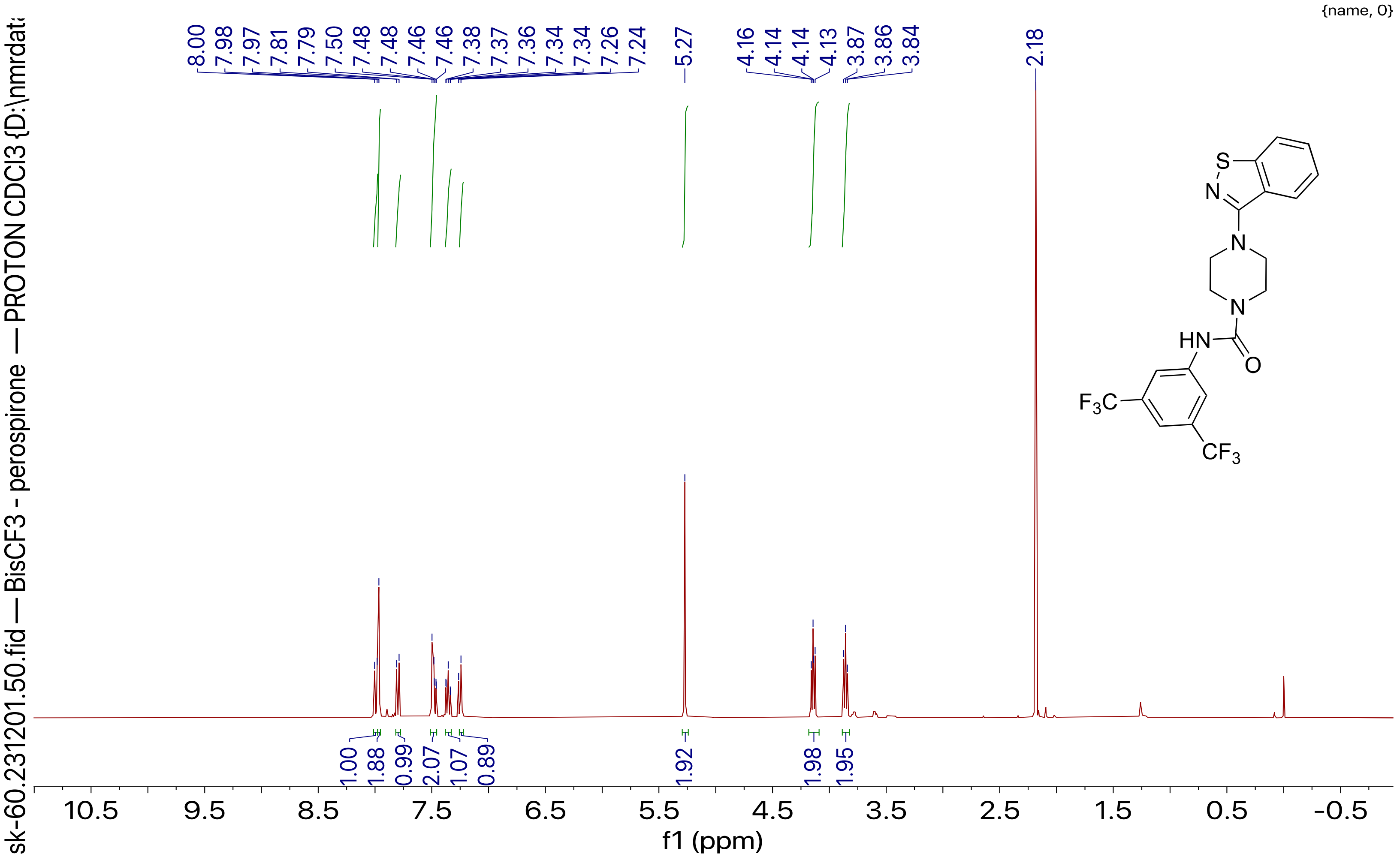

^{1}H NMR spectra of **9l'** (400 MHz, RT, $CDCl_3$)

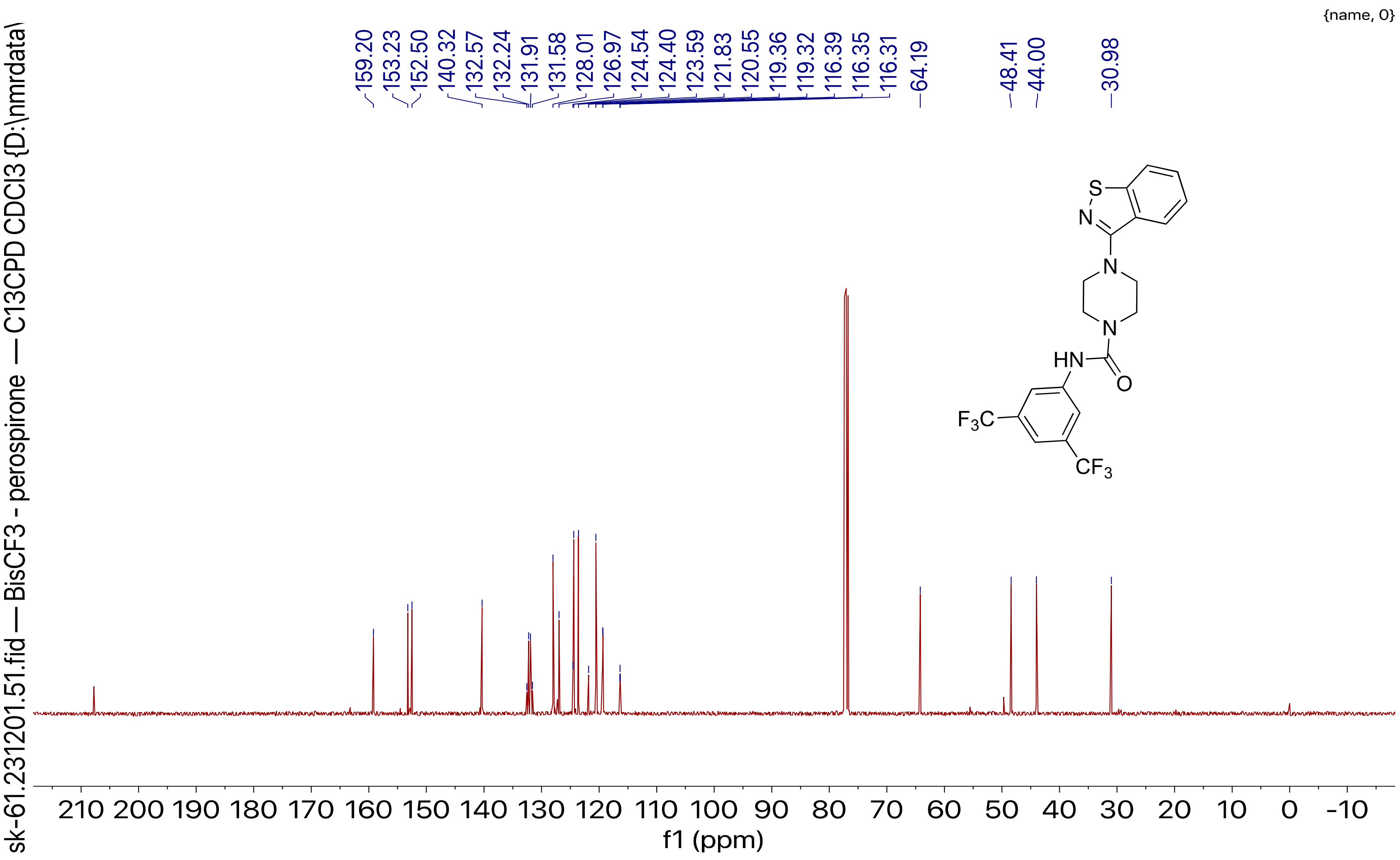

^{13}C NMR spectra of **9l’** (101 MHz, RT, $CDCl_3$)

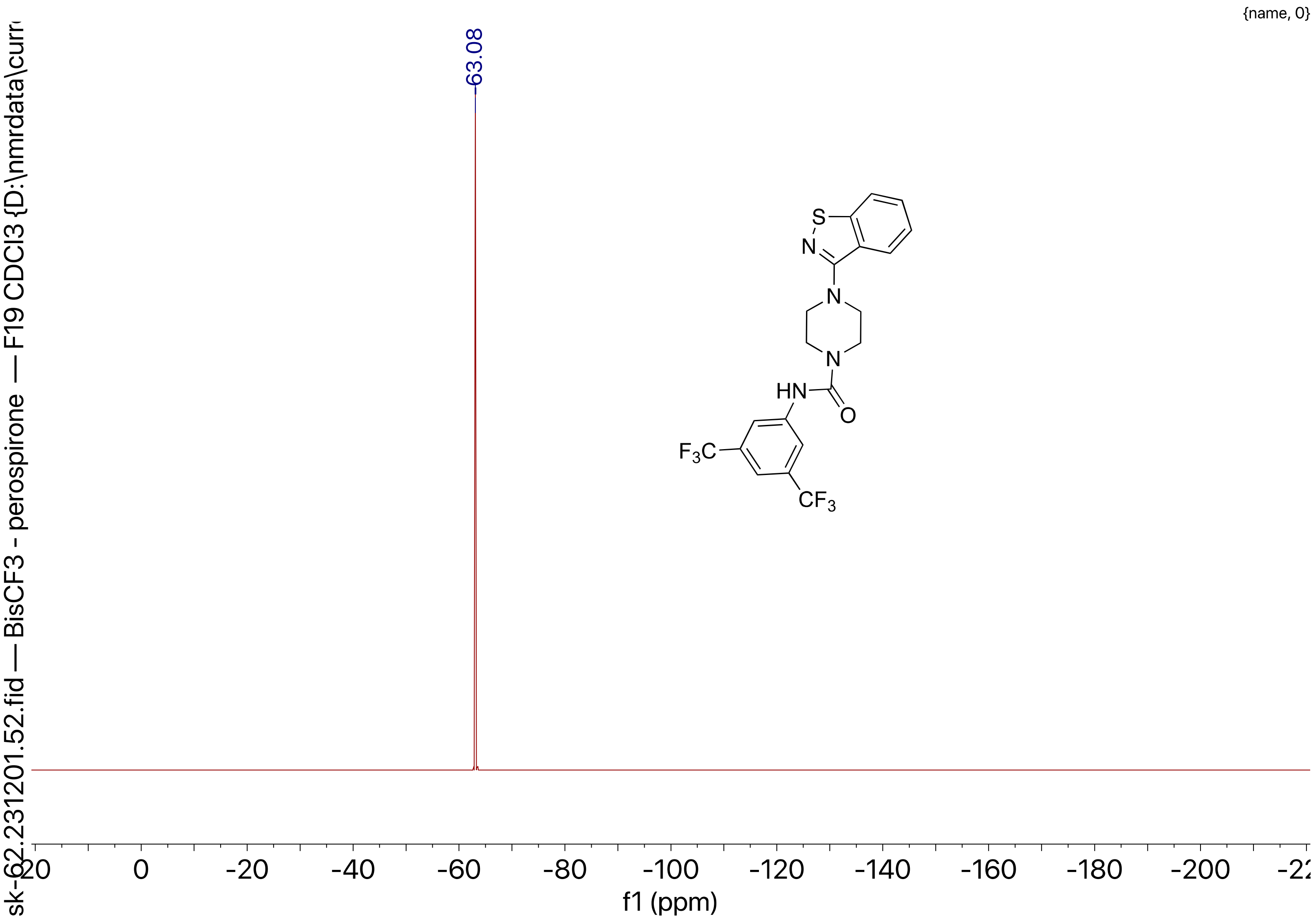

^{19}F NMR spectra of **9l’** (101 MHz, RT, $CDCl_3$)

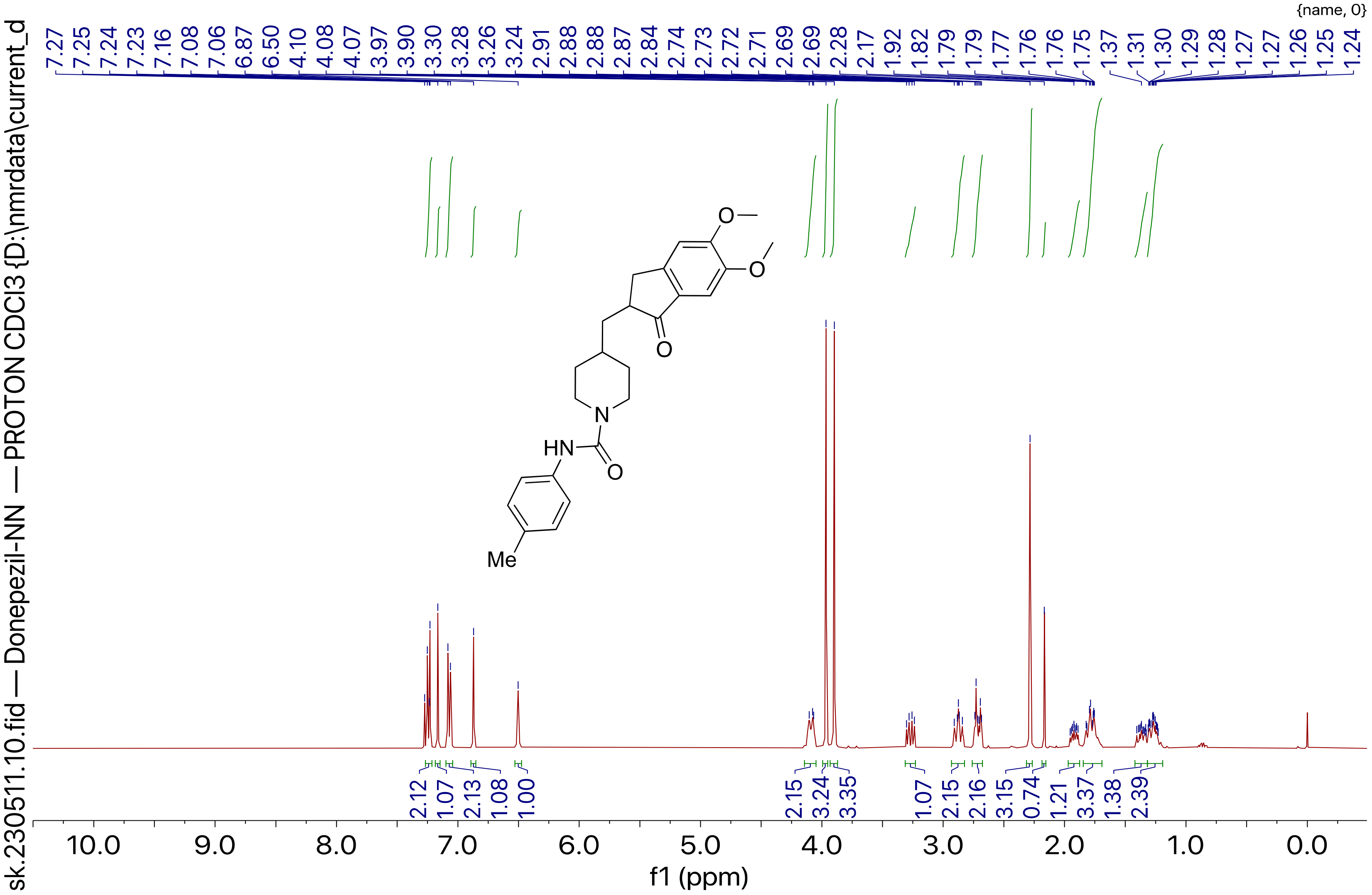

^{1}H NMR spectra of **9m** (400 MHz, RT, $CDCl_3$)

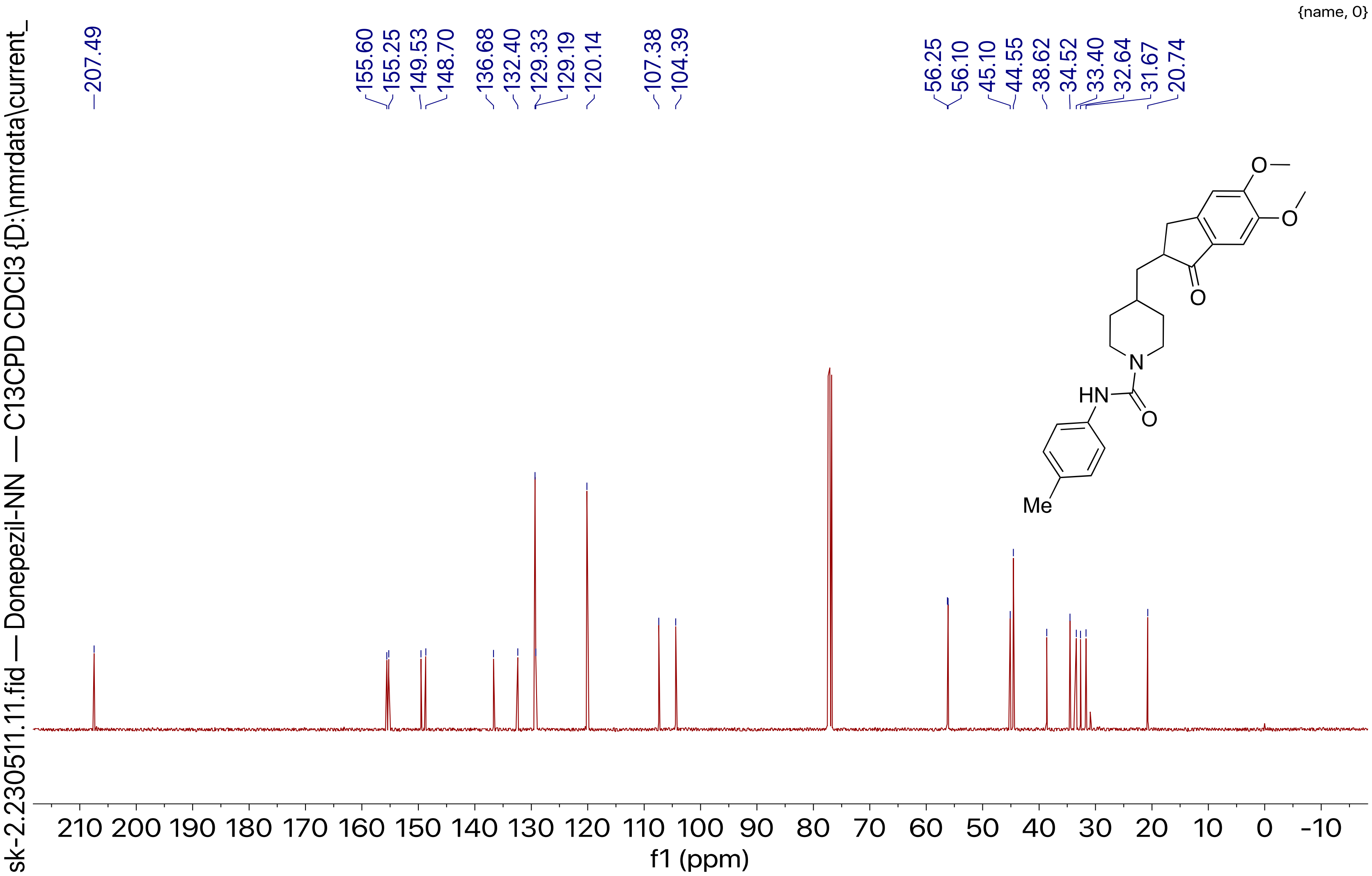

^{13}C NMR spectra of **9m** (101 MHz, RT, $CDCl_3$)

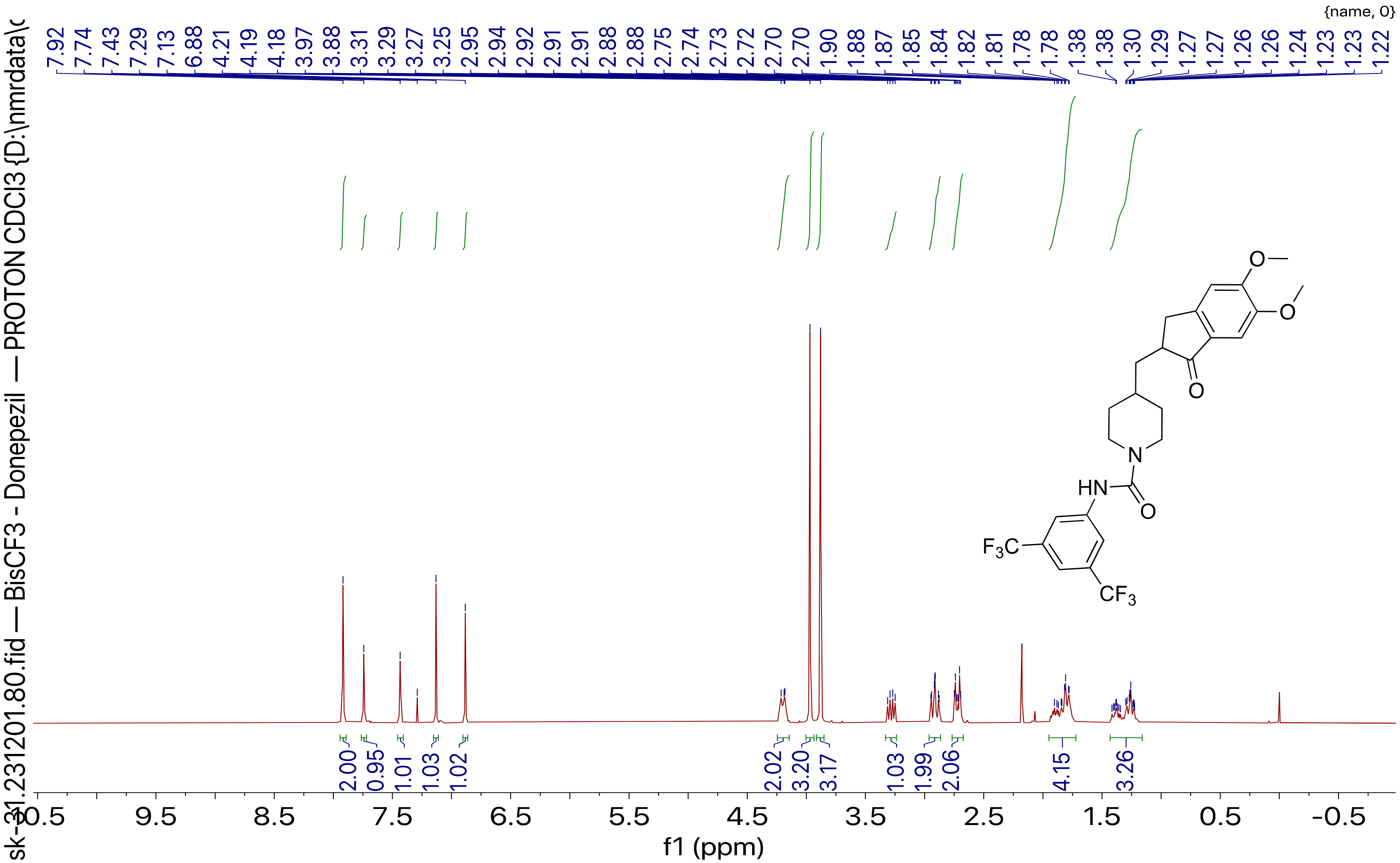

^{1}H NMR spectra of **9m’** (101 MHz, RT, $CDCl_3$)

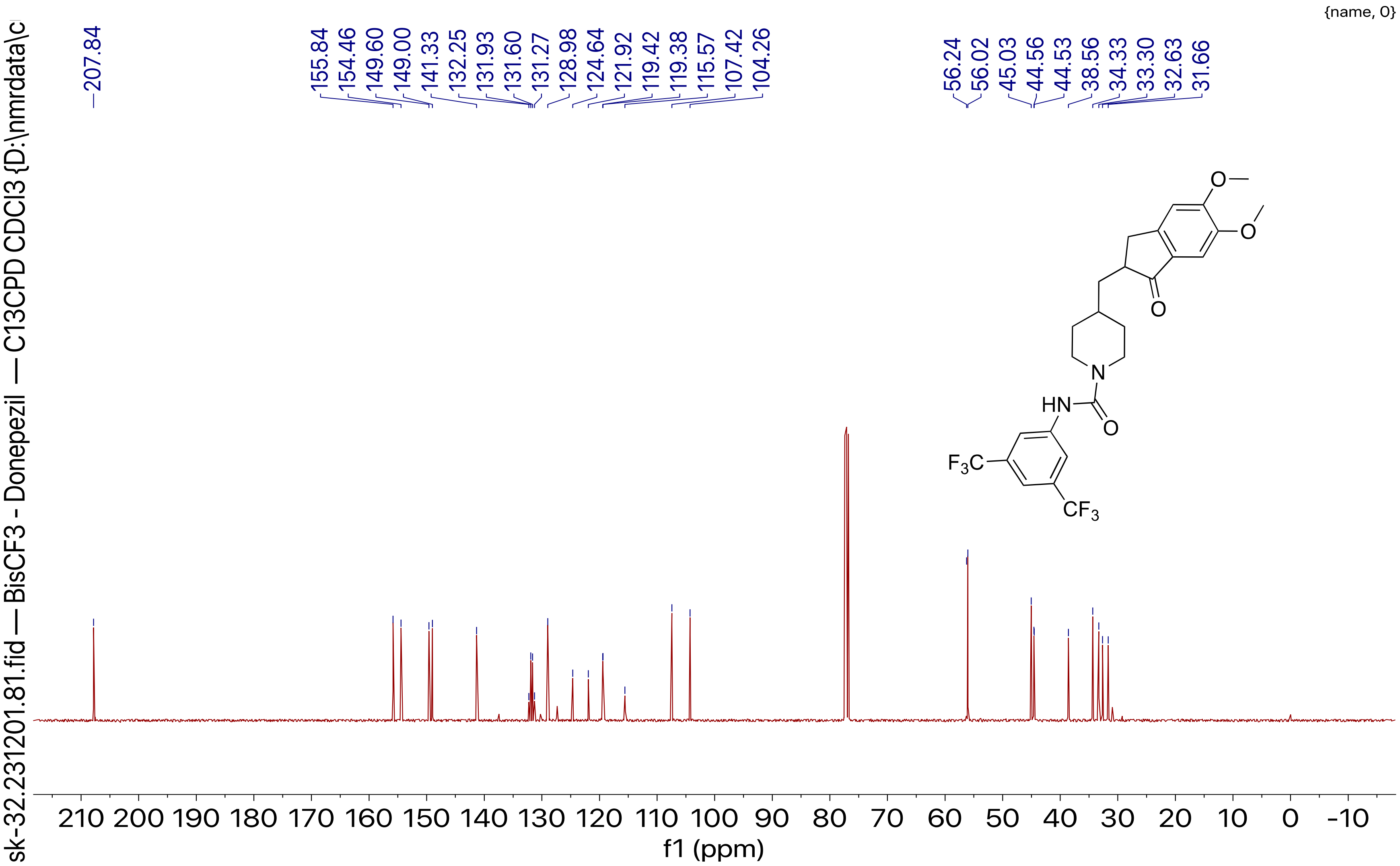

^{13}C NMR spectra of **9m’** (101 MHz, $CDCl_3$, RT)

{name, 0}

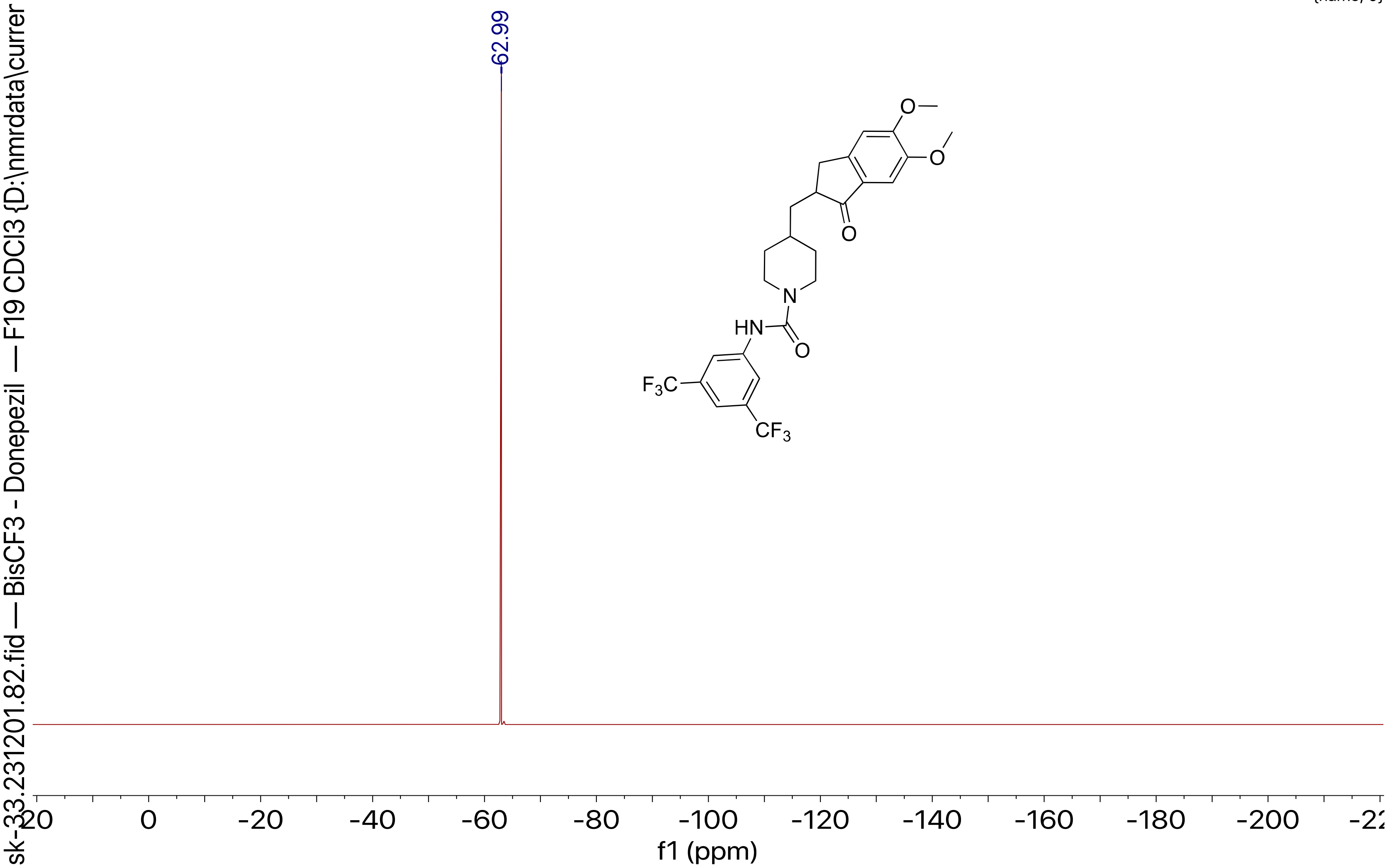

^{19}F NMR spectra of **9m’** (376 MHz, $CDCl_3$, RT)

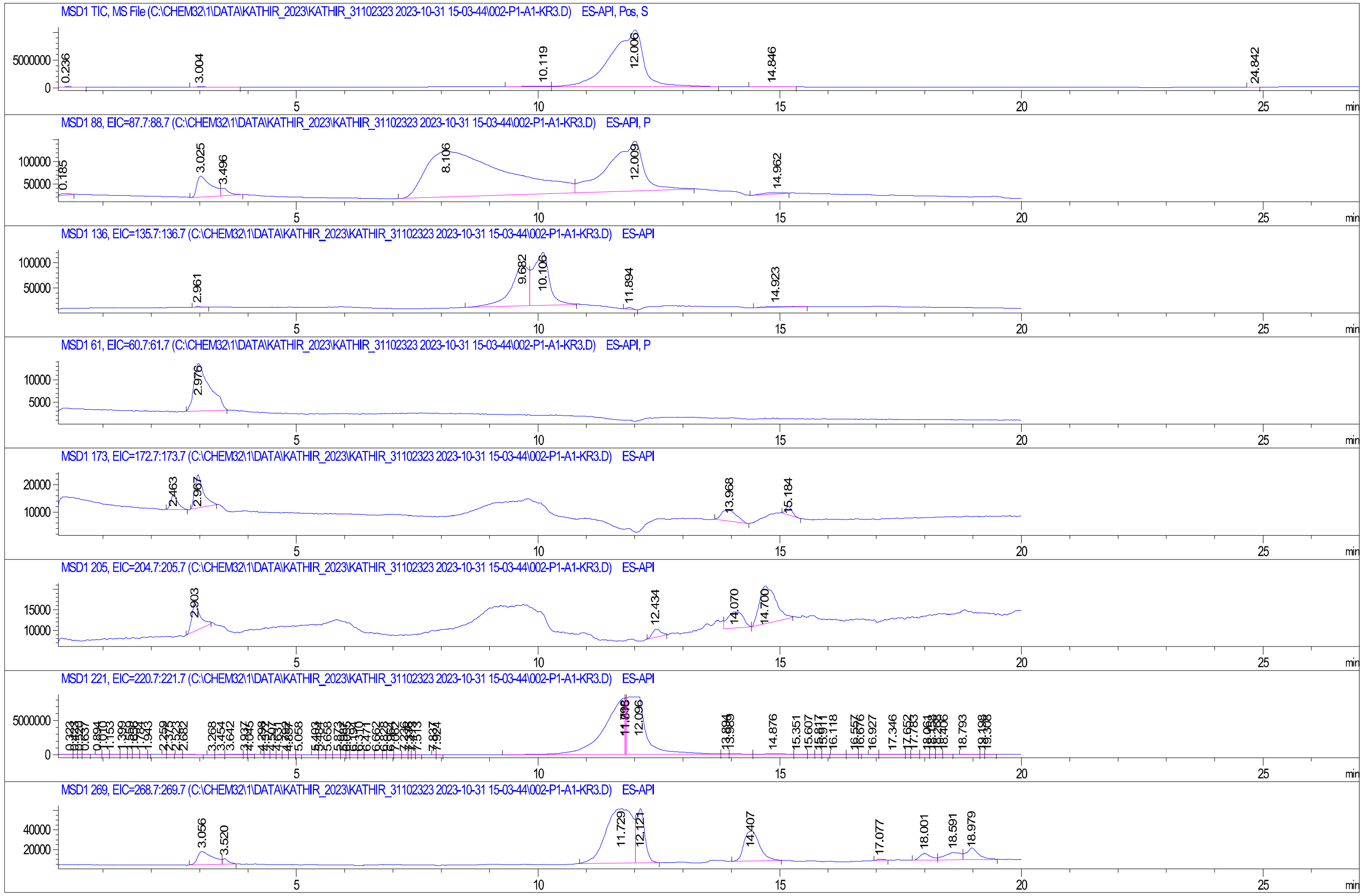

LC-MS spectra of crude reaction mixture to confirm AcOH released from reaction

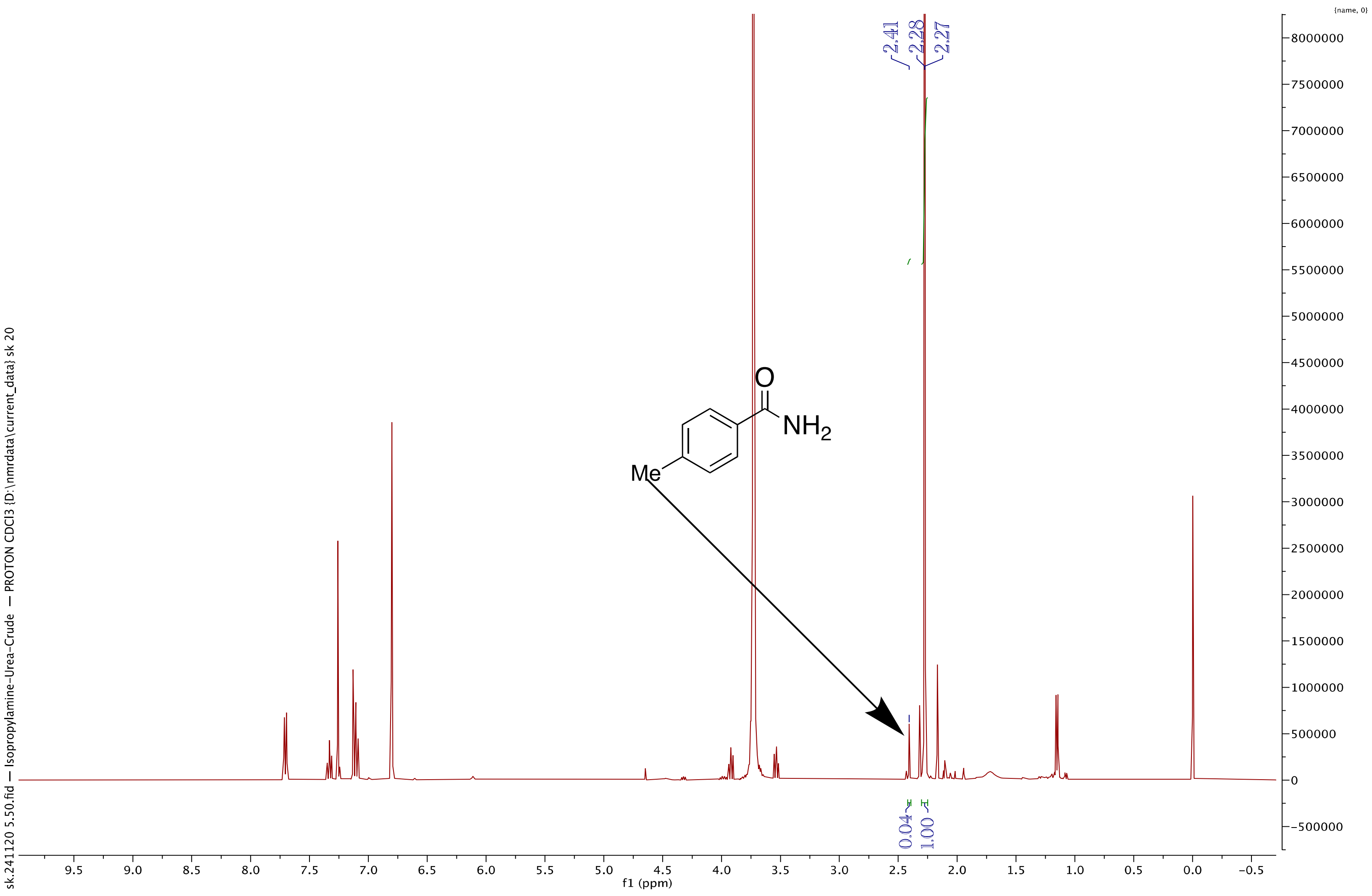

Crude yield analysis of unreacted amide in the reaction between **1a** and **2a** using mesitylene as internal standard
